# From Genomes to Phenotypes: Traitar, the Microbial Trait Analyzer

**DOI:** 10.1128/mSystems.00101-16

**Published:** 2016-12-27

**Authors:** Aaron Weimann, Kyra Mooren, Jeremy Frank, Phillip B. Pope, Andreas Bremges, Alice C. McHardy

**Affiliations:** a Computational Biology of Infection Research, Helmholtz Center for Infection Research, Braunschweig, Germany; b German Center for Infection Research (DZIF), Partner Site Hannover-Braunschweig, Braunschweig, Germany; c Department for Algorithmic Bioinformatics, Heinrich Heine University, Düsseldorf, Germany; d Department of Chemistry, Biotechnology and Food Science, Norwegian University of Life Sciences, Ås, Norway; University of Trento

**Keywords:** ancestral trait reconstruction, genotype-phenotype inference, metagenomics, microbial traits, phenotypes, phyletic patterns, single-cell genomics, support vector machines

## Abstract

The number of sequenced genomes is growing exponentially, profoundly shifting the bottleneck from data generation to genome interpretation. Traits are often used to characterize and distinguish bacteria and are likely a driving factor in microbial community composition, yet little is known about the traits of most microbes. We describe Traitar, the microbial trait analyzer, which is a fully automated software package for deriving phenotypes from a genome sequence. Traitar provides phenotype classifiers to predict 67 traits related to the use of various substrates as carbon and energy sources, oxygen requirement, morphology, antibiotic susceptibility, proteolysis, and enzymatic activities. Furthermore, it suggests protein families associated with the presence of particular phenotypes. Our method uses L1-regularized L2-loss support vector machines for phenotype assignments based on phyletic patterns of protein families and their evolutionary histories across a diverse set of microbial species. We demonstrate reliable phenotype assignment for Traitar to bacterial genomes from 572 species of eight phyla, also based on incomplete single-cell genomes and simulated draft genomes. We also showcase its application in metagenomics by verifying and complementing a manual metabolic reconstruction of two novel *Clostridiales* species based on draft genomes recovered from commercial biogas reactors. Traitar is available at https://github.com/hzi-bifo/traitar.

**IMPORTANCE** Bacteria are ubiquitous in our ecosystem and have a major impact on human health, e.g., by supporting digestion in the human gut. Bacterial communities can also aid in biotechnological processes such as wastewater treatment or decontamination of polluted soils. Diverse bacteria contribute with their unique capabilities to the functioning of such ecosystems, but lab experiments to investigate those capabilities are labor-intensive. Major advances in sequencing techniques open up the opportunity to study bacteria by their genome sequences. For this purpose, we have developed Traitar, software that predicts traits of bacteria on the basis of their genomes. It is applicable to studies with tens or hundreds of bacterial genomes. Traitar may help researchers in microbiology to pinpoint the traits of interest, reducing the amount of wet lab work required.

## Abstract (precis)

Bacteria are ubiquitous in our ecosystem and have a major impact on human health, e.g., by supporting digestion in the human gut. Bacterial communities can also aid in biotechnological processes such as wastewater treatment or decontamination of polluted soils. Diverse bacteria contribute with their unique capabilities to the functioning of such ecosystems, but lab experiments to investigate those capabilities are labor-intensive. Major advances in sequencing techniques open up the opportunity to study bacteria by their genome sequences. For this purpose, we have developed Traitar, software that predicts traits of bacteria on the basis of their genomes. It is applicable to studies with tens or hundreds of bacterial genomes. Traitar may help researchers in microbiology to pinpoint the traits of interest, reducing the amount of wet lab work required.

## INTRODUCTION

Microbes are often characterized and distinguished by their traits, for instance, in *Bergey’s Manual of Systematic Bacteriology* (1). A trait or phenotype can vary in complexity; for example, it can refer to the degradation of a specific substrate or the activity of an enzyme inferred in a lab assay, the respiratory mode of an organism, the reaction to Gram staining, or antibiotic resistances. Traits are also likely driving factors in microbial community composition (2). Microbial community members with various metabolic capabilities can aid in wastewater treatment, bioremediation of soils, and promotion of plant growth (3–5); in the cow rumen microbiota, bacterial cellulose degraders influence the ability to process plant biomass material (6). In the tammar wallaby foregut microbiome, the dominant bacterial species is implicated in the lower methane emissions produced by wallabies than by ruminants (7).

In addition to the exponential growth of available sequenced microbial genome isolates, metagenome and single-cell genome sequencing further contributes to the increasing number of available genomes. For the recovery of genomes from metagenomes (GFMs), computational methods based on, e.g., differential read coverage and k-mer usage were developed (8–13) that allow the recovery of genomes without the need to obtain microbial isolates in pure culture (6, 14). In addition, single-cell genomics provides another culture-independent analysis technique and also allows genome recovery, although often fragmented, for less abundant taxa in microbial communities (15, 16). Together, these developments profoundly shift the analytical bottleneck from data generation to interpretation.

The genotype-phenotype relationships for some microbial traits have been well studied. For instance, bacterial motility is attributed to the proteins of the flagellar apparatus (17). We have recently shown that delineating such relationships from microbial genomes and accompanying phenotype information with statistical learning methods enables the accurate prediction of the plant biomass degradation phenotype and the *de novo* discovery of both known and novel protein families that are relevant for the realization of the plant biomass degradation phenotype (18, 19). However, a fully automated software framework for prediction of a broad range of traits from only the genome sequence is currently missing. Additionally, horizontal gene transfer, a common phenomenon across bacterial genomes, has not been utilized to improve trait prediction so far. Traits with their causative genes may be transferred from one bacterium to another (20, 21) (e.g., for antibiotic resistances [22]), and the vertically transferred part of a bacterial genome might be unrelated to the traits under investigation (2, 23, 24).

**FIG 1:**
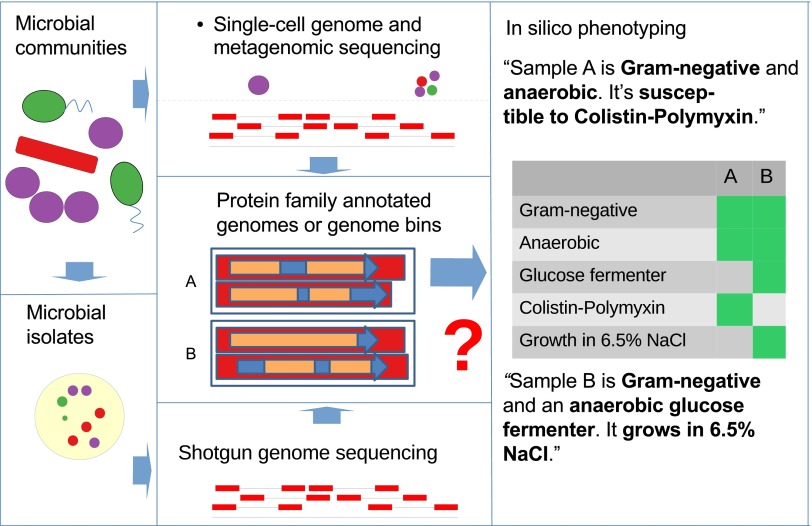
Traitar can be used to phenotype microbial community members on the basis of genomes recovered from single-cell sequencing or (metagenomic) environmental shotgun sequencing data or of microbial isolates. Traitar provides classification models based on protein family annotation for a wide variety of different phenotypes related to the use of various substrates as source of carbon and energy for growth, oxygen requirement, morphology, antibiotic susceptibility, and enzymatic activity.

Here we present Traitar, the microbial trait analyzer, an easy-to-use, fully automated software framework for the accurate prediction of currently 67 phenotypes directly from a genome sequence (Fig. 1). We used phenotype data from the microbiology section of the Global Infectious Disease and Epidemiology Online Network (GIDEON)—a resource dedicated to the diagnosis, treatment, and teaching of infectious diseases and microbiology (25)—for training phenotype classification models on the protein family annotation of a large number of sequenced genomes of microbial isolates (predominantly bacterial pathogens). We investigated the effect of incorporating ancestral protein family gains and losses into the model inference on classification performance to allow consideration of horizontal gene transfer events in the inference of phenotype-related protein families and phenotype classification. We rigorously tested the performance of our software in cross-validation experiments, on further test data sets and for different taxonomic ranks. To test Traitar’s applicability beyond the bacteria represented in GIDEON, we subsequently applied it to several hundred bacteria described in *Bergey’s Manual of Systematic Bacteriology* (1). We used Traitar to phenotype bacterial single amplified genomes (SAGs) and simulated incomplete genomes to investigate its potential for the phenotyping of microbial samples with incomplete genome sequences. We characterized two novel *Clostridiales* species of a biogas reactor community with Traitar on the basis of their genomes recovered with metagenomics. This verified and complemented a manual metabolic reconstruction. As Traitar furthermore suggests protein families associated with the presence of a particular phenotype, we discuss the protein families Traitar identified for several phenotypes, namely, for motility, nitrate-to-nitrite conversion, and l-arabinose fermentation.

Traitar is implemented in Python 2.7. It is freely available under the open-source GPL 3.0 license at https://github.com/hzi-bifo/traitar and as a Docker container at https://hub.docker.com/r/aweimann/traitar. A Traitar web service can be accessed at https://research.bifo.helmholtz-hzi.de/traitar.

## RESULTS

### The Traitar software.

We begin with a description of the Traitar software and phenotype classifiers. Traitar predicts the presence or absence of a phenotype, i.e., assigns a phenotype label, for 67 microbial traits to every input sequence sample (Table 1; see Table S1 in the supplemental material). For each of these traits, Traitar furthermore suggests candidate protein families associated with its realization, which can be subjects of experimental follow-up studies.

10.1128/mSystems.00101-16.1 Table S1  Detailed information on the 67 phenotypes used in this study. Download Table S1, PDF file, 0.5 MB. Copyright © 2016 Weimann et al. 2016 Weimann et al. This content is distributed under the terms of the Creative Commons Attribution 4.0 International license.

**TABLE 1:** The 67 traits available in Traitar for phenotyping (we grouped each of these phenotypes into a microbiological or biochemical category)

Phenotype^a^	Category^b^
Alkaline phosphatase	Enzyme
Beta-hemolysis	
Coagulase production	
Lipase	
Nitrate-to-nitrite conversion	
Nitrite to gas	
Pyrrolidonyl-β-naphthylamide	
Bile susceptible	Growth
Colistin-polymyxin susceptible	
DNase	
Growth at 42°C	
Growth in 6.5% NaCl	
Growth in KCN	
Growth on MacConkey agar	
Growth on ordinary blood agar	
Mucate utilization	
Arginine dihydrolase	Growth, amino acid
Indole	
Lysine decarboxylase	
Ornithine decarboxylase	
Acetate utilization	Growth, carboxylic acid
Citrate	
Malonate	
Tartrate utilization	
Gas from glucose	Growth, glucose
Glucose fermenter	
Glucose oxidizer	
Methyl red	
Voges-Proskauer	
Cellobiose	Growth, sugar
d-Mannitol	
d-Mannose	
d-Sorbitol	
d-Xylose	
Esculin hydrolysis	
Glycerol	
Lactose	
l-Arabinose	
l-Rhamnose	
Maltose	
Melibiose	
*myo*-Inositol	
ONPG^c^ (β-galactosidase)	
Raffinose	
Salicin	
Starch hydrolysis	
Sucrose	
Trehalose	
Urea hydrolysis	
Bacillus or coccobacillus	Morphology
Coccus	
Coccus—clusters or groups predominate	
Coccus—pairs or chains predominate	
Gram negative	
Gram positive	
Motile	
Spore formation	
Yellow pigment	
Aerobe	Oxygen
Anaerobe	
Capnophilic	
Facultative	
Catalase	Oxygen, enzyme
Oxidase	
Hydrogen sulfide	Product
Casein hydrolysis	Proteolysis
Gelatin hydrolysis	

a GIDEON phenotypes with at least 10 presence and 10 absence labels.

b Phenotypes assigned to microbiological/biochemical categories.

c ONPG, *o*-nitrophenyl-β-d-galactopyranoside.

For phenotype prediction, Traitar uses one of two different classification models. We trained the first classifier—the phypat classifier—on the protein and phenotype presence and absence labels from 234 bacterial species (see phenotype models in Materials and Methods). The 2nd classifier—the phypat+PGL classifier—was trained by using the same data and additionally information on evolutionary protein family and phenotype gains and losses. The latter were determined by using maximum-likelihood inference of their ancestral character states on the species phylogeny (see ancestral protein family and phenotype gains and losses in Materials and Methods).

**FIG 2:**
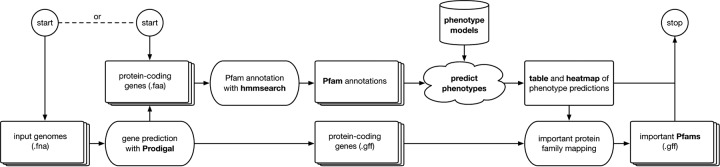
Work flow of Traitar. Input to the software can be genome sequence samples in nucleotide or amino acid FASTA format. Traitar predicts phenotypes on the basis of precomputed classification models and provides graphic and tabular output. In the case of nucleotide sequence input, the protein families that are important for the phenotype predictions will be further mapped to the predicted protein-coding genes.

The input to Traitar is either a nucleotide sequence FASTA file for every sample, which is run through gene prediction software, or a protein sequence FASTA file. Traitar then annotates the proteins with protein families. Subsequently, it predicts the presence or absence of each of the 67 traits for every input sequence. Note that Traitar does not require a phylogenetic tree for the input samples. Finally, it associates the predicted phenotypes with the protein families that contributed to these predictions (Fig. 2). A parallel execution of Traitar is supported by GNU parallel (26). The Traitar annotation procedure and the training of the phenotype models are described in more detail below (see Traitar software in Materials and Methods).

### Evaluation.

**FIG 3:**
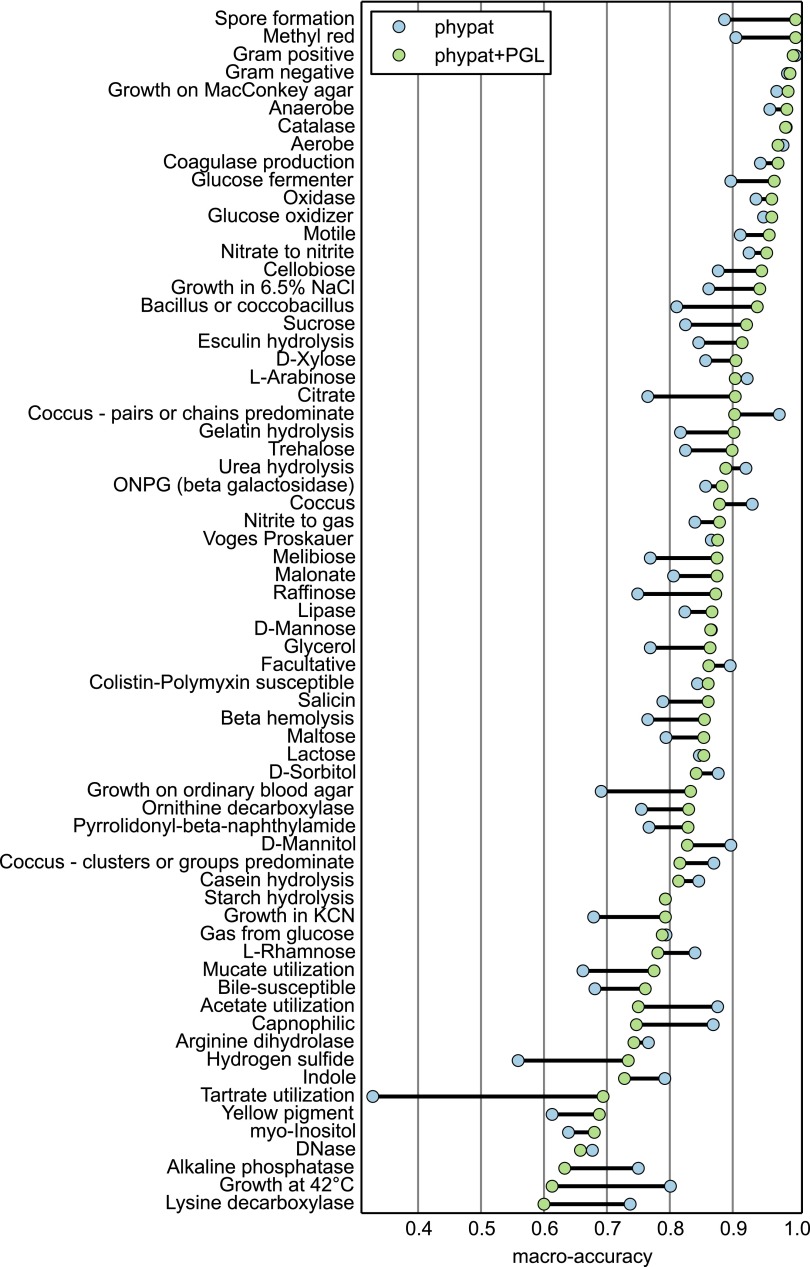
Macroaccuracy for each phenotype of the Traitar phypat and phypat+PGL phenotype classifiers determined in nested cross-validation of 234 bacterial species described in GIDEON (see evaluation metrics in Materials and Methods; Table 1; see Table S1 in the supplemental material).

We evaluated the two Traitar classifiers by using 10-fold nested cross-validation of 234 bacterial species found in GIDEON (GIDEON I). The macroaccuracy (the accuracy balanced over all phenotypes) determined for the 67 GIDEON phenotypes was 82.6% for the phypat classifier and 85.5% for the phypat+PGL classifier; the accuracy (fraction of correct assignments averaged over all of the samples tested) for phypat was 88.1%, in comparison to 89.8% for phypat+PGL (see evaluation metrics in Materials and Methods; Table 2). Notably, Traitar classified 53 phenotypes with >80% macroaccuracy and 26 phenotypes with at least 90% macroaccuracy with one of the two classifiers (Fig. 3; see Table S2 in the supplemental material). Phenotypes that could be predicted with very high confidence included the outcome of a methyl red test, spore formation, oxygen requirement (i.e., anaerobe and aerobe), and growth on MacConkey agar or catalase. Some phenotypes proved to be difficult to predict (60 to 70% macroaccuracy), which included DNase, *myo*-inositol, yellow pigment, and tartrate utilization, regardless of which classifier was used. This might be caused by the relatively small number (<20) of positive (phenotype present) examples that were available.

10.1128/mSystems.00101-16.2 Table S2  Macroaccuracy of the phypat and phypat+PGL classifiers obtained in cross-validation experiments for the 67 GIDEON phenotypes. Download Table S2, PDF file, 0.1 MB. Copyright © 2016 Weimann et al. 2016 Weimann et al. This content is distributed under the terms of the Creative Commons Attribution 4.0 International license.

**TABLE 2:** Evaluation of the Traitar phypat and phypat+PGL phenotype classifiers and a consensus vote of both classifiers for 234 bacteria described in GIDEON in a 10-fold nested cross-validation by using different evaluation measures^a^

Data set (no. of bacteria) and classifier	Macroaccuracy	Accuracy	Recall phenotype	
			Positive	Negative
GIDEON I (234)				
Phypat	82.6	88.1	86.1	91.4
Phypat+PGL	**85.5**	**89.8**	**87.8**	90.9
Consensus	83.0	88.8	82.2	**95.4**
GIDEON II (42)				
Phypat	85.3	87.5	84.9	90.2
Phypat+PGL	**86.7**	**87.9**	**86.3**	89.7
Consensus	85.7	87.2	80.8	**93.7**
*Bergey’s Manual of Systematic Bacteriology* (296)				
Phypat	NA^b^	**72.9**	**74.6**	71.2
Phypat+PGL	NA^b^	72.4	74	70.8
Consensus	NA^b^	**72.9**	66.6	**79.2**

a See evaluation metrics in Materials and Methods. Subsequently, we tested another 42 bacteria from GIDEON and 296 bacteria described in *Bergey’s Manual of Systematic Bacteriology* for an independent performance assessment of the two classifiers. Bold values depict the best performance obtained across the Phypat, Phypat+PGL, and consensus classifiers for each measure.

b Only the overall accuracy is reported, as insufficient phenotype labels (fewer than five with negative and positive labels, respectively) were available for several phenotypes, to enable a comparable macroaccuracy calculation to the other data sets (see Table S1 in the supplemental material).

For an independent assessment of Traitar’s classification performance, we next tested Traitar with 42 bacterial species that had phenotype information available in GIDEON (GIDEON II) but were not used for learning the phenotype models (see annotation in the Traitar software). For calculation of macroaccuracy, we considered only phenotypes represented by at least five phenotype-positive and five phenotype-negative bacteria. On these data, Traitar predicted the phenotypes with a macroaccuracy of 85.3% with the phypat classifier and 86.7% with the phypat+PGL classifier and accuracies of 87.5% and 87.9%, respectively (Table 2). To investigate the performance of Traitar for bacterial genomes from a different data source, we next determined from two volumes of *Bergey’s Manual of Systematic Bacteriology*, namely, the *Proteobacteria* and the *Firmicutes*, the phenotypes of further sequenced bacteria that were not in our GIDEON I and II data sets (see Tables S1 and S4 in the supplemental material). In total, we thus identified phenotypes for another 296 sequenced bacterial species (see annotation in the Traitar software). Also for these bacteria, Traitar performed well but was less reliable than before, with accuracies of 72.9% for the phypat classifier and 72.1% for the phypat+PGL classifier (Table 2). This is likely due to the taxonomic differences among the bacteria listed in GIDEON and *Bergey’s Manual of Systematic Bacteriology* and also because most of the bacteria in *Bergey’s Manual of Systematic Bacteriology* have only draft genomes available for phenotyping.

When combining the predictions of the phypat and phypat+PGL classifiers into a consensus vote, Traitar assigns phenotypes more reliably, while predicting fewer phenotype labels than the individual classifiers (Table 2). Depending on the use case, Traitar can be used with performance characterized by different tradeoffs between the recall of the phenotype-positive and phenotype-negative classes.

### Performance per taxon at different ranks of the taxonomy.

**FIG 4:**
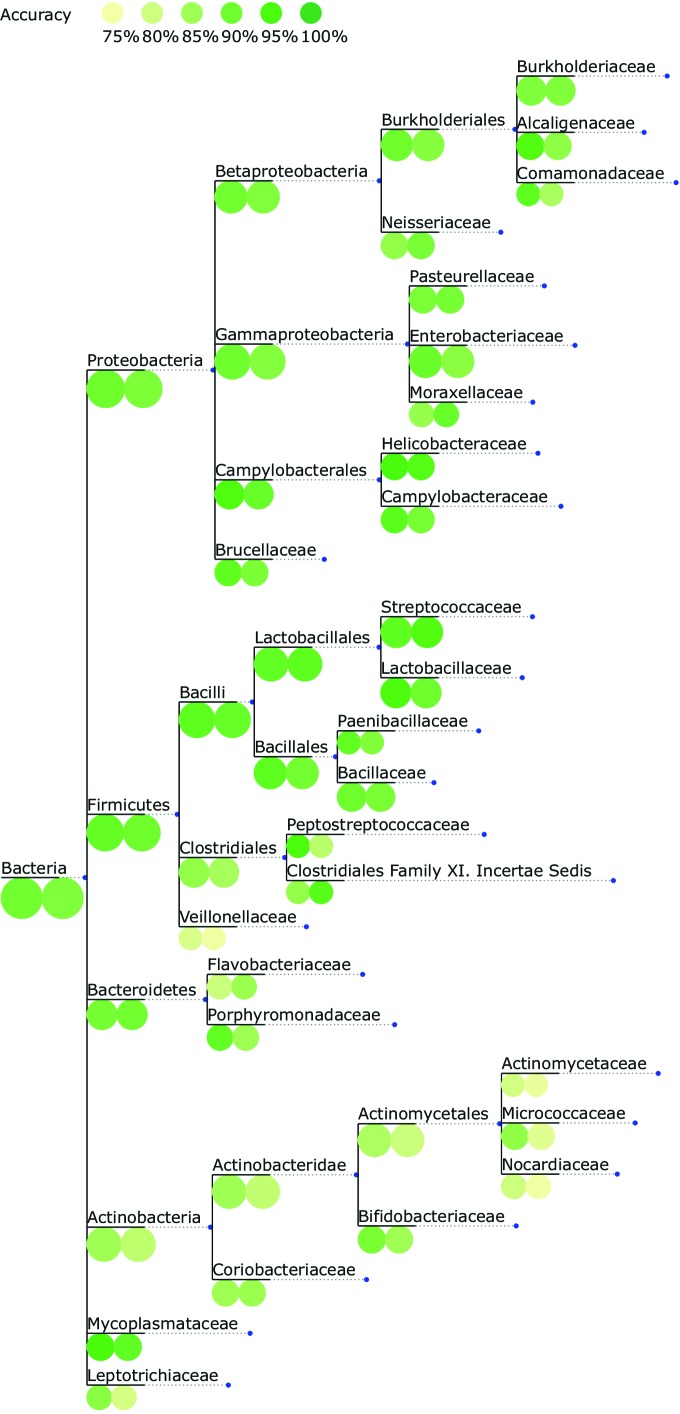
Classification accuracy for each taxon at different ranks of the NCBI taxonomy. For better visualization of names for the internal nodes, the taxon names are displayed on branches leading to the respective taxon node in the tree. The nested cross-validation accuracy obtained with Traitar for 234 bacterial species described in GIDEON was projected onto the NCBI taxonomy down to the family level. Colored circles at the tree nodes depict the performance of the phypat+PGL classifier (left-hand circles) and the phypat classifier (right-hand circles). The size of the circles reflects the number of species per taxon.

We investigated the performance of Traitar across the part of the bacterial tree of life represented in our data set. For this purpose, we evaluated the nested cross-validation performance of the phypat and phypat+PGL classifiers at different ranks of the National Center for Biotechnology Information (NCBI) taxonomy. For a given GIDEON taxon, we pooled all of the bacterial species that are descendants of this taxon. Figure 4 shows the accuracy estimates projected on the NCBI taxonomy from the domain level down to individual families. Notably, the accuracy of the phypat+PGL (phypat) classifier for the phyla covered by at least five bacterial species showed low variance and was high across all of the phyla, i.e., 84% (81%) for *Actinobacteria*, >90% (89%) for *Bacteroidetes*, 89% (90%) for *Proteobacteria*, 91% (90%) for *Firmicutes*, and 91% (86%) for *Tenericutes*.

### Phenotyping of incomplete genomes.

GFMs or SAGs are often incomplete, and thus we analyzed the effect of missing genome assembly parts on the performance of Traitar. Rinke et al. used a single-cell sequencing approach to analyze poorly characterized parts of the bacterial and archaeal tree of life, the so-called microbial dark matter (16). They pooled 20 SAGs from the “*Candidatus* Cloacimonetes” phylum, formerly known as WWE1, to generate joint—more complete—genome assemblies that had at least a genome-wide average nucleotide identity of 97% and belonged to a single 16S rRNA gene-based operational taxonomic unit, namely, “*Candidatus* Cloacamonas acidaminovorans” (27).

**FIG 5:**
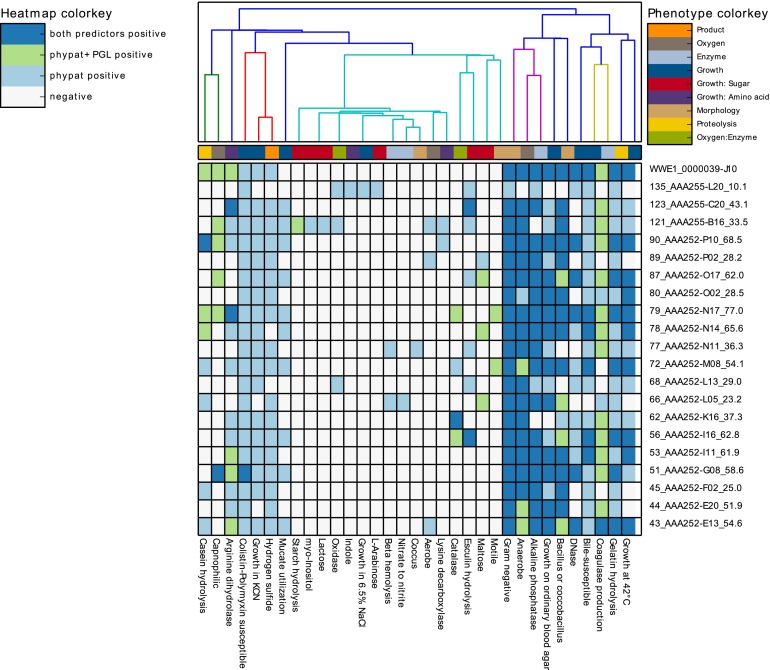
Single-cell phenotyping with Traitar. We used 20 genome assemblies with various degrees of completeness from single cells of the “*Candidatus* Cloacimonetes” phylum and a joint assembly for phenotyping with Traitar. Shown is a heat map of assembly samples versus phenotypes, which is the standard visualization for phenotype predictions in Traitar. The origin of the phenotype’s prediction (Traitar phypat and/or phypat+PGL classifier) determines the color of the heat map entries. The sample labels have their genome completeness estimates as suffixes. The colors of the dendrogram indicate similar phenotype distributions across samples, as determined by a hierarchical clustering with SciPy (http://docs.scipy.org/doc/scipy/reference/cluster.hierarchy.html).

According to our predictions based on the joint assembly of the single-cell genomes, “*Candidatus* Cloacamonas acidaminovorans” is Gram negative and is adapted to an anaerobic lifestyle, which agrees with the description of Rinke et al. (Fig. 5). Traitar further predicted arginine dihydrolase activity, which is in line with the characterization of the species as an amino acid degrader (16). Remarkably, the prediction of a bacillus or coccobacillus shape agrees with the results of Limam et al. (28), who used a WWE1-specific probe and characterized the samples by fluorescence *in situ* hybridization. They furthermore reported that members of the “*Candidatus* Cloacimonetes” phylum are implicated in the anaerobic digestion of cellulose primarily in early hydrolysis, which is in line with the very limited carbohydrate degradation spectrum found by Traitar.

**FIG 6:**
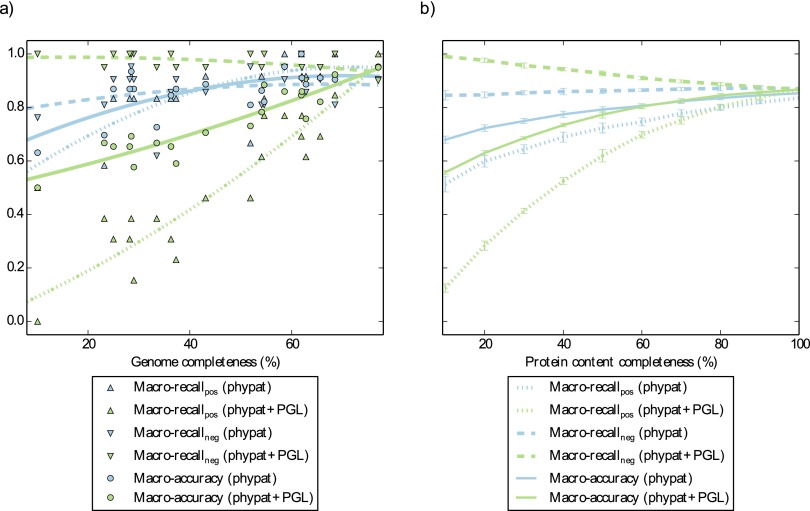
Phenotyping of simulated draft genomes and single-cell genomes. In panel a, we used 20 genome assemblies with various degrees of completeness from single cells of the “*Candidatus* Cloacimonetes” phylum and a joint assembly for phenotyping with the Traitar phypat and phypat+PGL classifiers. Shown is the performance of the phenotype prediction versus the genome completeness of the single cells with respect to the joint assembly. In panel b, we simulated draft genomes on the basis of an independent test set of 42 microbial (pan)genomes. The coding sequences of these genomes were downsampled (10 replications per sampling point), and the resulting simulated draft genomes were used for phenotyping with the Traitar phypat and phypat+PGL classifiers. We plotted various performance estimates (mean center values and standard deviation error bars are shown) against protein content completeness.

Subsequently, we compared the predicted phenotypes for the SAGs to the predictions for the joint assembly. The phypat classifier recalled more of the phenotype predictions of the joint assembly based on the SAGs than the phypat+PGL classifier. However, the phypat+PGL classifier made fewer false-positive predictions (Fig. 6a).

In the next experiment, we inferred phenotypes based on simulated GFMs by subsampling from the coding sequences of each of the 42 bacterial genomes (GIDEON II). Starting with the complete set of coding sequences, we randomly deleted genes from the genomes. For the draft genomes obtained with different degrees of completeness, we reran the Traitar classification and computed the accuracy measures as before. We observed that the average fraction of phenotypes identified (macrorecall for the positive class) of the phypat+PGL classifier dropped more quickly with more missing coding sequences than that of the phypat classifier (Fig. 6b). However, at the same time, the recall of the negative class of the phypat+PGL classifier improved with a decreasing number of coding sequences, meaning that fewer but more reliable predictions were made.

Overall, the tradeoffs in the recall of the phenotype-positive and phenotype-negative classes of the two classifiers resulted in a similar overall macroaccuracy across the range of tested genome completeness. Thus, depending on the intended use, a particular classifier can be chosen. We expect that the reliable predictions inferred with the phypat+PGL classifier and the more abundant but less reliable predictions made with the phypat classifier will complement one another in different use cases for partial genomes recovered from metagenomic data.

By analyzing the protein families with assigned weights and the bias terms of the two classifiers, we found the phypat+PGL classifier to base its predictions primarily on the presence of protein families that were typical for the phenotypes. In contrast, the phypat classifier also took typically absent protein families from phenotype-positive genomes into account in its decision. More technically, the positive weights in models of the phypat classifier are balanced out by negative weights, whereas for the phypat+PGL classifier, they are balanced out by the bias term. By downweighting the bias term for the phypat+PGL classifier by the protein content completeness, we could show that the accuracy of the phypat classifier could be exceeded by the phypat+PGL classifier, regardless of the protein content completeness (data not shown). However, this requires knowledge of the protein content completeness for each genomic sample, which could be indirectly estimated by using methods such as checkM (29).

### Traitar as a resource for gene target discovery.

**FIG 7:**
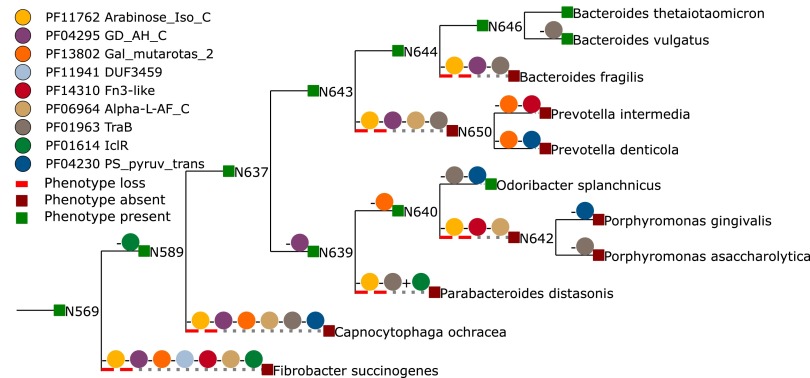
Phenotype gain and loss dynamics match protein family dynamics. Shown are the phenotype-protein family gain and loss dynamics for families identified as important by Traitar for the L-arabinose phenotype. Signed colored circles along the tree branches depict protein family gains (+) or losses (−). Taxon nodes are colored according to their inferred (ancestral) phenotype state.

In addition to phenotype assignment, Traitar suggests the protein families relevant for the assignment of a phenotype (see majority feature selection in Materials and Methods, Table 3). Here, as an example, we demonstrate this capability for three phenotypes that are already well studied, namely, motile, nitrate-to-nitrite conversion, and l-arabinose metabolism. These phenotypes each represent one of the phenotype categories morphology, enzymatic activity, and growth on sugar. In general, we observed that the protein families important for classification can be seen to be gained and lost jointly with the respective phenotypes within the microbial phylogeny (Fig. 7).

**TABLE 3:** The most relevant Pfam families for the classification of three important phenotypes, nitrate-to-nitrite conversion, motility, and l-arabinose^a^

Accession no.	Phenotype	Pfam description	Remark
PF13677	Motile	Membrane MotB of proton-channel complex MotA/MotB	Flagellar protein
PF03963	Motile	Flagellar hook capping proteinN-terminal region	Flagellar protein
PF02561	Motile	Flagellar FliS protein	Flagellar protein
PF02050	Motile	Flagellar FliJ protein	Flagellar protein
PF07559	Motile	Flagellar basal body protein FlaE	Flagellar protein
PF13682	Motile	Chemoreceptor zinc-binding domain	Chemotaxis related
PF03350	Motile	Uncharacterized protein family, UPF0114	
PF05226	Motile	CHASE2 domain	Chemotaxis related
PF07194	Motile	P2 response regulator binding domain	Chemotaxis related
PF04982	Motile	HPP family	
PF03927	Nitrate-to-nitrite conversion	NapD protein	Involved in Nar formation
PF13247	Nitrate-to-nitrite conversion	4Fe-4S dicluster domain	Iron-sulfur cluster center of beta subunit of Nar
PF03892	Nitrate-to-nitrite conversion	Nitrate reductase cytochrome *c*-type subunit (NapB)	Periplasmic Nap subunit
PF02613	Nitrate-to-nitrite conversion	Nitrate reductase delta subunit	Nap subunit
PF01127	Nitrate-to-nitrite conversion	Succinate dehydrogenase/fumarate reductase transmembrane subunit	
PF01292	Nitrate-to-nitrite conversion	Prokaryotic cytochrome *b*_561_	
PF03459	Nitrate-to-nitrite conversion	TOBE domain	
PF03824	Nitrate-to-nitrite conversion	High-affinity nickel transport protein	
PF04879	Nitrate-to-nitrite conversion	Molybdopterin oxidoreductase Fe_4_S_4_ domain	Bound to alpha subunit of Nar
PF02665	Nitrate-to-nitrite conversion	Nitrate reductase gamma subunit	Nar subunit
PF11762	l-Arabinose	l-Arabinose isomerase C-terminal domain	Catalyzes first reaction in l-arabinose metabolism
PF04295	l-Arabinose	d-Galactarate dehydratase/altronate hydrolase, C terminus	
PF13802	l-Arabinose	Galactose mutarotase-like	
PF11941	l-Arabinose	Domain of unknown function (DUF3459)	
PF14310	l-Arabinose	Fibronectin type III-like domain	
PF06964	l-Arabinose	α-l-ArabinofuranosidaseC terminus	Acts on l-arabinose side chains in pectins
PF01963	l-Arabinose	TraB family	
PF01614	l-Arabinose	Bacterial transcriptional regulator	
PF06276	l-Arabinose	Ferric iron reductase FhuF-like transporter	
PF04230	l-Arabinose	Polysaccharide pyruvyl transferase	

a We ranked the Pfam families with positive weights in the Traitar SVM classifiers by the correlation of the Pfam families with the respective phenotype labels across 234 bacteria described in GIDEON. Shown are the 10 highest ranking Pfam families along with their descriptions and a description of their phenotype-related function, where we found one.

Among the selected Pfam families that are important for classifying the motility phenotype were proteins of the flagellar apparatus and chemotaxis-related proteins (Table 3). Motility allows bacteria to colonize their preferred environmental niches. Genetically, it is attributed mainly to the flagellum, which is a molecular motor, and is closely related to chemotaxis, a process that lets bacteria sense chemicals in their surroundings. Motility also plays a role in bacterial pathogenicity, as it enables bacteria to establish and maintain an infection. For example, pathogens can use flagella to adhere to their host and have been reported to be less virulent if they lack flagella (30). Of the 48 flagellar proteins described in reference 31, 4 (FliS, MotB, FlgD, and FliJ) were sufficient for accurate classification of the motility phenotype and were selected by our classifier, as well as FlaE, which was not included in this collection. FliS (accession no. PF02561) is a known export chaperone that inhibits early polymerization of the flagellar filament FliC in the cytosol (32). MotB (PF13677), part of the membrane proton-channel complex, acts as the stator of the bacterial flagellar motor (33). Traitar also identified further protein families related to chemotaxis, such as CZB (PF13682), a family of chemoreceptor zinc-binding domains found in many bacterial signal transduction proteins involved in chemotaxis and motility (34), and the P2 response regulator-binding domain (PF07194). The latter is connected to the chemotaxis kinase CheA and is thought to enhance the phosphorylation signal of the signaling complex (35).

Nitrogen reduction in nitrate-to-nitrite conversion is an important step of the nitrogen cycle and has a major impact on agriculture and public health. Two types of nitrate reductases are found in bacteria, the membrane-bound Nar and periplasmic Nap nitrate reductases (36), both of which we found to be relevant for the classification of the phenotype. We identified all of the subunits of the Nar complex as being relevant for the nitrate-to-nitrite conversion phenotype (i.e., the gamma and delta subunits [PF02665, PF02613]), as well as Fer4_11 (PF13247), which is in the iron-sulfur center of the beta subunit of Nar. The delta subunit is involved in the assembly of the Nar complex and is essential for its stability but probably is not directly part of it (37). Traitar also identified the molybdopterin oxidoreductase Fe_4_S_4_ domain (PF04879), which is bound to the alpha subunit of the nitrate reductase complex (37). Traitor furthermore suggested as relevant NapB (PF03892), which is a subunit of the periplasmic Nap protein, and NapD (PF03927), which is an uncharacterized protein implicated in Nap formation (36).

l-Arabinose is major constituent of plant polysaccharides that is located, for instance, in pectin side chains and is an important microbial carbon source (38). Traitar identified the l-arabinose isomerase C-terminal domain (PF11762), which catalyzes the first step in l-arabinose metabolism—the conversion of l-arabinose into l-ribulose (39), as being important for realizing l-arabinose metabolism (Fig. 7). It furthermore suggested the C-terminal domain of α-l-arabinofuranosidase (PF06964), which cleaves nonreducing terminal α-l-arabinofuranosidic linkages in l-arabinose-containing polysaccharides (40) and is also part of the well-studied l-arabinose operon of *Escherichia coli* (39).

### Phenotyping of biogas reactor population genomes.

We used Traitar to phenotype two novel *Clostridiales* species (unClos_1, unFirm_1) on the basis of their genomic information reconstructed from metagenome samples. These were taken from a commercial biogas reactor operating with municipal waste (41). The genomes of unClos_1 and unFirm_1 were estimated to be 91 and 60% complete, respectively, on the basis of contigs of ≥5 kb. Traitar predicted unClos_1 to utilize a broader spectrum of carbohydrates than unFirm_1 (Table 4). We cross-referenced our predictions with a metabolic reconstruction conducted by Frank et al. (64). We considered all phenotype predictions that Traitar inferred with either the phypat or the phypat+PGL classifier. The manual reconstruction and predictions inferred with Traitar agreed to a great extent (Table 4). Traitar recalled 87.5% (6/7) of the phenotypes inferred via the metabolic reconstruction and also agreed to 81.8% (9/11) on the absent phenotypes. Notable exceptions were that Traitar found only a weak signal for d-xylose utilization. A weak signal means that only a minority of the classifiers in the voting committee assigned these samples to the phenotype-positive class (see phenotype models in Materials and Methods). However, the metabolic reconstruction was also inconclusive with respect to xylose fermentation. Furthermore, Traitar found only a weak signal for glucose fermentation by unFirm_1. While genomic analysis of unFirm_1 revealed the Embden-Meyerhof-Parnas (EMP) pathway, which would suggest glucose fermentation, gene-centric and metaproteomic analyses of this phylotype indicated that the EMP pathway was probably employed in an anabolic direction (gluconeogenesis); therefore, unFirm_1 is also unlikely to ferment d-mannose. This suggests that unFirm_1 is unlikely to ferment sugars and instead metabolizes acetate (also predicted by Traitar; Table 4) via a syntrophic interaction with hydrogen-utilizing methanogens.

**TABLE 4:**
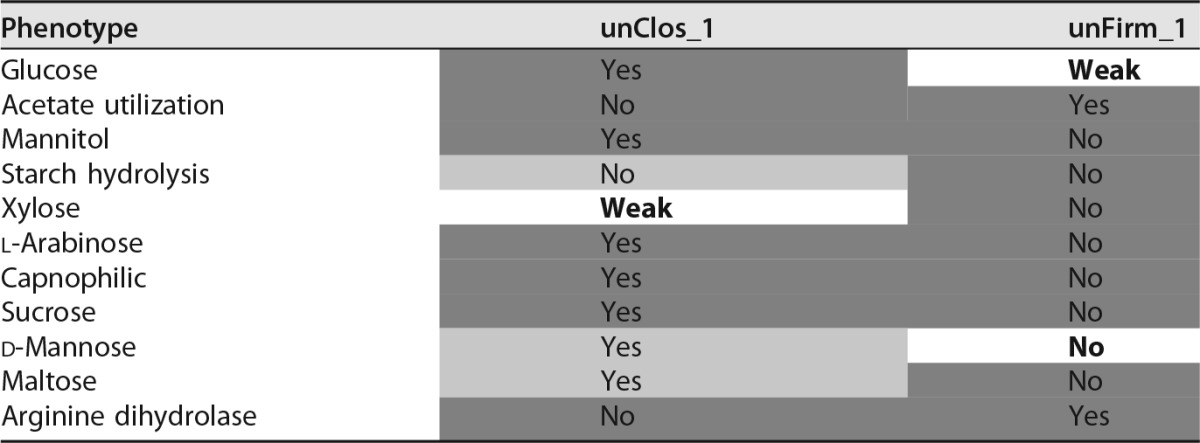
Phenotype predictions for two novel *Clostridiales* species with genomes reconstructed from a commercial biogas reactor metagenome^a^

a Traitar output (yes, no, weak) was cross-referenced with phenotypes manually reconstructed on the basis of Kyoto Encyclopedia of Genes and Genomes orthology annotation (64), which are primarily the fermentation phenotypes of various sugars. We considered all of the phenotype predictions that Traitar inferred with either the phypat or the phypat+PGL classifier. A weak prediction means that only a minority of the classifiers in the Traitar voting committee assigned this sample to the phenotype-positive class (Traitar phenotype). Entries shaded light gray show a difference between the prediction and the reconstruction, whereas dark gray denotes an overlap; bold (no shading) is inconclusive.

Traitar predicted further phenotypes for both species that were not targeted by the manual reconstruction. One of these predictions was an anaerobic lifestyle, which is likely to be accurate, as the genomes were isolated from an anaerobic bioreactor environment. It also predicted them to be Gram positive, which is probably correct, as the Gram-positive sortase protein family can be found in both genomes. This is a Gram positivity biomarker (42). Furthermore, all *Firmicutes* known so far are Gram positive (1). Additionally, Traitar assigned motile and spore formation to unFirm_1 on the basis of the presence of several flagellar proteins (i.e., FlaE, FliM, MotB, FliS, and FliJ) and the sporulation proteins CoatF and YunB.

## DISCUSSION

We have developed Traitar, a software framework for predicting phenotypes from the protein family profiles of bacterial genomes. Traitar provides a quick and fully automated way of assigning 67 different phenotypes to bacteria on the basis of the protein family contents of their genomes.

Microbial trait prediction from phyletic patterns has been proposed in previous studies for a limited number of phenotypes (18, 19, 43–46). To our knowledge, the only currently available software for microbial genotype-phenotype inference is PICA, which is based on learning associations of clusters of orthologous genes (47) with traits (45). Recently, PICA was extended by Feldbauer et al. for predicting 11 traits overall, optimized for large data sets, and tested on incomplete genomes (46). Of the 67 phenotypes that Traitar predicts, 60 are entirely novel. It furthermore includes different prediction modes, one based on phyletic patterns, one additionally including a statistical model of protein family evolution for its predictions. An initial prototype of the Traitar methodology was originally developed for prediction of the plant biomass phenotype, with excellent classification performance observed and providing suggestions of candidate domains for experimental verification (18). The methodology has since been adapted to the use of GIDEON and inclusion of phylogenetic signals, which is why the plant biomass predictor is not included in the Traitar release. This shows that, principally, given suitable training data, also very complex phenotypes can be learned and predicted with this methodology.

Traitar also suggests associations between phenotypes and protein families. For three traits, we showed that several of these associations are to known key families of establishment of a particular trait. Furthermore, candidate families were suggested that might be relevant for particular traits and serve as targets for experimental studies. Some of the phenotypes annotated in GIDEON are specific for the human habitat (such as coagulase production or growth on ordinary blood agar), and the genetic underpinnings learned by Traitar could be interesting to study for infection disease research.

In cross-validation experiments with phenotype data from the GIDEON database, we showed that the Traitar phypat classifier has high accuracy in phenotyping bacterial samples. Consideration of ancestral protein family gains and losses in the classification, which is implemented in the Traitar phypat+PGL classifier, improves the accuracy compared to prediction from phyletic patterns only, both for individual phenotypes and overall. Barker et al. were the first to note the phylogenetic dependence of genomic samples and how this can lead to biased conclusions (24). MacDonald et al. selected protein families on the basis of correlations with a phenotype and corrected for the taxonomy (45). Here we accounted for the evolutionary history of the phenotype and the protein families in the classifier training itself to automatically improve phenotype assignment. We additionally demonstrated the reliability of the performance estimates by phenotyping, with similar accuracy, an independent test data set with bacteria described in GIDEON that we did not use in the cross-validation. Traitar also reliably phenotyped a large and heterogenic collection of bacteria that we extracted from *Bergey’s Manual of Systematic Bacteriology*—mostly with only draft genomes available. We did not observe any bias toward specific taxa in GIDEON, but some of the phenotypes might be realized with different protein families in taxa that are less well represented, as indicated by the around 15 to 20% less reliable phenotyping results for bacteria described in *Bergey’s Manual of Systematic Bacteriology*. We expect that the accuracy of the phenotype classification models already available in Traitar will further improve as more data become available and can be incorporated into its training.

We found that Traitar can provide reliable insights into the metabolic capabilities of microbial community members even from partial genomes, which are very common for genomes recovered from single cells or metagenomes. One obvious limitation being for incomplete genomes, the absence of a phenotype prediction may be due to the absence of the relevant protein families from the input genomes. The analysis of both the SAGs and simulated genomes led us to the same conclusions, i.e., that the phypat classifier is more suitable for exploratory analysis, as it assigned more phenotypes to incomplete genomes at the price of more false-positive predictions. In contrast, the phypat+PGL classifier assigned fewer phenotypes but also made fewer false assignments. At the moment, genotype-phenotype inference with Traitar only takes into account the presence and absence of protein families of the bacteria analyzed. This information can be readily computed from the genomic and metagenomic data. Future research could focus also on the integration of other omics data to allow even more accurate phenotype assignments. Additionally, expert knowledge of the biochemical pathways that are used in manual metabolic reconstructions, for example, could be integrated as prior knowledge into the model in future studies.

For the phenotyping of novel microbial species, generating a detailed (manual) metabolic reconstruction such as the one by Frank et al. (64) is time-intensive. Furthermore, such reconstructions are usually focused on specific pathways and are dependent on the research question. This is not an option for studies with tens or hundreds of genomes, which are becoming more and more common in microbiology (6, 14, 16). Traitar thus is likely to be particularly helpful for multigenome studies. It furthermore may pick up on things outside the original research focus and could serve as a seed or a first-pass method for a detailed metabolic reconstruction in future studies.

## MATERIALS AND METHODS

### The Traitar software.

In this section, we first describe the Traitar annotation procedure. We proceed with the genome and phenotype data used for the training of Traitar phenotype models; afterward, we explain the training and illustrate how we considered ancestral protein family gains and losses in the models. Finally, we specify the requirements for running the Traitar software.

### Annotation.

In the case of nucleotide DNA sequence input, Traitar uses Prodigal (48) for gene prediction prior to Pfam family annotation. The amino acid sequences are then annotated in Traitar with protein families (Pfams) from the Pfam database (version 27.0) (49) by using the hmmsearch command of HMMER 3.0 (50).

Each Pfam family has a hand-curated threshold for the bit score, which is set in such a way that no false positive is included (51). A fixed threshold of 25 is then applied to the bit score (the log-odds score), and all Pfam domain hits with an *E* value above 10^−2^ are discarded. The resulting Pfam family counts (phyletic patterns) are turned into presence or absence values, as we found this representation to yield favorable classification performance (18).

### Genome and phenotype data.

We obtained our phenotype data from the GIDEON database (25). In GIDEON, a bacterium is labeled either as phenotype positive, phenotype negative, or strain specific. In the latter case, we discarded this phenotype label. The GIDEON traits can be grouped into the categories such as the use of various substrates as sources of carbon and energy for growth, oxygen requirement, morphology, antibiotic susceptibility, and enzymatic activity (Table 1; see Table S1 in the supplemental material). We considered only phenotypes that were available in GIDEON for at least 20 bacteria, with a minimum of 10 bacteria annotated as positive (phenotype presence) and 10 as negative (phenotype absence) for a given phenotype to enable a robust and reliable analysis of the respective phenotypes. Furthermore, for inclusion in the analysis, we required each bacterial sample to have (i) at least one annotated phenotype, (ii) at least one sequenced strain, and (iii) a representative in the sequenced tree of life (sTOL).

In total, we extracted 234 species-level bacterial samples with 67 phenotypes with sufficient total, positive, and negative labels from GIDEON (GIDEON I). GIDEON associates these bacteria with 9,305 individual phenotype labels, 2,971 being positive and 6,334 negative (see Tables S1 and S3 in the supplemental material). GIDEON species that had at least one sequenced strain available but were not part of the sTOL were set aside for a later independent assessment of classification accuracy. In total, this additional data set comprised a further 42 unique species with 58 corresponding sequenced bacterial strains (GIDEON II; see Tables S1 and S4). We obtained 1,836 additional phenotype labels for these bacteria, consisting of 574 positive and 1,262 negative ones. We searched the *Firmicutes* and *Proteobacteria* volumes of *Bergey’s Manual of Systematic Bacteriology* specifically for further bacteria not represented so far in the GIDEON data sets (1). In total, we obtained phenotype data from *Bergey’s Manual of Systematic Bacteriology* for 206 *Firmicutes* and 90 *Proteobacteria* with a total of 1,152 positive labels and 1,376 negative labels (see Tables S1 and S5). As in GIDEON, in *Bergey’s Manual of Systematic Bacteriology*, the phenotype information is usually given on the species level.

10.1128/mSystems.00101-16.3 Table S3  Mapping of bacterial strains to 234 species described in GIDEON with links to the NCBI databases. Download Table S3, PDF file, 0.2 MB. Copyright © 2016 Weimann et al. 2016 Weimann et al. This content is distributed under the terms of the Creative Commons Attribution 4.0 International license.

10.1128/mSystems.00101-16.4 Table S4  Mapping of bacterial strains to 42 species in GIDEON with links to the NCBI databases. Download Table S4, PDF file, 0.1 MB. Copyright © 2016 Weimann et al. 2016 Weimann et al. This content is distributed under the terms of the Creative Commons Attribution 4.0 International license.

10.1128/mSystems.00101-16.5 Table S5  Mapping of bacterial strains to 296 species described in *Bergey’s Manual of Systematic Bacteriology*. Download Table S5, PDF file, 0.5 MB. Copyright © 2016 Weimann et al. 2016 Weimann et al. This content is distributed under the terms of the Creative Commons Attribution 4.0 International license.

We downloaded the coding sequences of all of the complete bacterial genomes that were available via the NCBI FTP server at ftp://ftp.ncbi.nlm.nih.gov/genomes/ as of 11 May 2014 and genomes available from the PATRIC database as of September 2015 (52). These were annotated with Traitar. For bacteria with more than one sequenced strain available, we chose the union of the Pfam family annotation of the single genomes to represent the pangenome Pfam family annotation, as in reference 53.

### Phenotype models.

We represented each phenotype from the set of GIDEON phenotypes across all genomes as a vector *yp* and solved a binary classification problem by using the matrix of Pfam phyletic patterns *XP* across all genomes as input features and *yp a*s the binary target variable (see Fig. S1 in the supplemental material). For classification, we relied on support vector machines (SVMs), which are a well-established machine learning method (54). Specifically, we used a linear L1-regularized L2-loss SVM for classification as implemented in the LIBLINEAR library (55). For many data sets, linear SVMs achieve accuracy comparable to that of SVMs with a nonlinear kernel but allow faster training. The weight vector of the separating hyperplane provides a direct link to the Pfam families that are relevant for the classification. L1 regularization enables feature selection, which is useful when applied to highly correlated and high-dimensional data sets such as those used in this study (56). We used the interface to LIBLINEAR implemented in scikit-learn (57). For classification of unseen data points—genomes without available phenotype labels supplied by the user—Traitar uses a voting committee of five SVMs with the best single cross-validation accuracy (see cross-validation below). Traitar then assigns each unseen data point to the majority class (phenotype presence or absence class) of the voting committee.

10.1128/mSystems.00101-16.7 Figure S1  Schematic overview of Traitar phenotype model training. (a) The phenotype and Pfam protein family phyletic patterns correspond to gain events on a star-shaped phylogenetic tree. Alternatively, we reconstructed the ancestral Pfam family and phenotype gain and loss events on the sTOL. (b) We trained an SVM classifier either on the phyletic patterns and on the ancestral gain and loss events or solely on the phyletic patterns. (c) In this way, we inferred classification models for all available phenotypes. Download Figure S1, PDF file, 0.1 MB. Copyright © 2016 Weimann et al. 2016 Weimann et al. This content is distributed under the terms of the Creative Commons Attribution 4.0 International license.

### Ancestral protein family and phenotype gains and losses.

We constructed an extended classification problem by including ancestral protein family gains and losses, as well as the ancestral phenotype gains and losses in our analysis, as implemented in GLOOME (58). Barker et al. report that common methods for inferring functional links between genes that do not take the phylogeny into account suffer from high rates of false positives (24). Here, we jointly derived the classification models from the observable phyletic patterns and phenotype labels, and from phylogenetically unbiased ancestral protein family and phenotype gains and losses, which we inferred via a maximum-likelihood approach from the observable phyletic patterns on a phylogenetic tree, showing the relationships among the samples (see Fig. S1 in the supplemental material). Ancestral character state evolution in GLOOME is modeled via a continuous-time Markov process with exponential waiting times. The gain and loss rates are sampled from two independent gamma distributions (59).

GLOOME needs a binary phylogenetic tree with branch lengths as the input. The taxonomy of the NCBI and other taxonomies are not suitable because they provide no branch length information. We used the sTOL (60), which is bifurcating and was inferred by a maximum-likelihood approach based on unbiased sampling of structural protein domains from whole genomes of all sequenced organisms (61). We employed GLOOME with standard settings to infer posterior probabilities for the phenotype and Pfam family gains and losses from the Pfam phyletic patterns of all of the NCBI bacteria represented in the sTOL and the GIDEON phenotypes. Each GIDEON phenotype *p* is available for a varying number of bacteria. Therefore, for each phenotype, we pruned the sTOL to those bacteria that were present in the NCBI database and had a label for the respective phenotype in GIDEON. The posterior probabilities of ancestral Pfam gains and losses were then mapped onto this GIDEON phenotype-specific tree (Gps-sTOL; see Fig. S2 in the supplemental material).

10.1128/mSystems.00101-16.8 Figure S2  sTOL and Gps-sTOL correspondence. The phenotype label for sample B is not available. Consequently, only branches *b*_4_, *b*_5_, and *b*_6_ are also found in the Gps-sTOL. The posterior probabilities for a Pfam gain or loss are the same for *b*_4_, *b*_5_, and *b*_6_ in both trees. Branches *b*_1_ and *b*_3_ (blue) are collapsed into a single branch. The posterior probability for a gain on branch *b*_1,3_, *g*_*b*_1,3__, is computed from the posterior probability for a Pfam gain for *b*_1_ and *b*_3_, *g*_*b*_1__ and *g*_*b*_3__, as follows: *g*_*b*_1,3__ = *g*_*b*_1__ + (1 − *g*_*b*_1__)·*g*_*b*_3__. Branch *b*_2_ (red) in the sTOL does not have an analog in the Gps-sTOL. Download Figure S2, PDF file, 0.01 MB. Copyright © 2016 Weimann et al. 2016 Weimann et al. This content is distributed under the terms of the Creative Commons Attribution 4.0 International license.

Let *B* be the set of all branches in the sTOL and *P* be the set of all Pfam families. We then denote the posterior probability *g*_*ij*_ of an event *a* for a Pfam family *pf* to be a gain event on branch *b* in the sTOL computed with GLOOME as

$$g_{ij}=P\left(a=gain\;|\;i=b,j=pf\right)\forall i\in B,\forall j\in P$$

and the posterior probability of *a* to be a loss event for a Pfam family *p* on branch *b* as

$$l_{ij}=P\left(a=loss\;|\;i=b,j=pf\right)\forall i\in B,\forall j\in P$$

We established a mapping *f*:*B*ʹ→*B* between the branches of the sTOL *B* and the set of branches *B*ʹ of the Gps-sTOL (see Fig. S2 in the supplemental material). This was achieved by traversing the tree from the leaves to the root.

There are two different scenarios for branch *b*ʹ in *B*ʹ to map to the branches in *B*.

(i) Branch *b*ʹ in the Gps-sTOL derives from single branch *b* in the sTOL as follows: *f*(*b*ʹ) = {*b*}. The posterior probability of a Pfam gain inferred in the Gps-sTOL on branch *b*ʹ consequently is the same as that on branch *b* in the sTOL: $g_{b'j}=g_{bj}\forall j\epsilon P$.

(ii) Branch *b*ʹ in the Gps-sTOL derives from *m* branches *b*_1_........, *b*_*m*_ in the sTOL as follows: *f*(*b*ʹ) = {*b*_1_,........,*b*_*m*_**}** (see Fig. S2). In this case, we iteratively calculated the posterior probabilities for at least one Pfam gain *g*ʹ on branch *b*ʹ from the posterior probabilities for a gain $g{'}_{b_{1}j}$. From the posterior probabilities *g*_1_.......,*g*_*m*_ of a gain on branches *b*_1_.......,*b*_*m*_ with the help of *h*,

$$\begin{array}{lll} h_{1} & = & \;g_{b_{1}j}\\ h_{n\;+\;1}\; & = & \;(1-h_{n})\cdot g_{b_{n\;+\;1}j}\\ g{'}_{b_{1}j} & = & {\;h}_{m}\forall j\in P. \end{array}$$

Inferring the Gps-sTOL Pfam posterior loss probabilities (*l*ʹ_*ij*_) from the sTOL posterior Pfam loss probabilities is analogous to deriving the gain probabilities. The posterior probability for a phenotype (*p*) to be gained (*g*ʹ_*ip*_) or lost (*l*ʹ_*ip*_) can be directly defined for the Gps-sTOL in the same way as for the Pfam gain and loss probabilities.

For classification, we did not distinguish between phenotype or Pfam gains or losses, assuming that the same set of protein families gained with a phenotype will also be lost with the phenotype. This assumption simplified the classification problem. Specifically, we proceeded in the following way.

(i) We computed the joint probability *x*ʹ_*ij*_ of a Pfam family gain or loss on branch *b*ʹ and the joint probability *y*_*j*_ of a phenotype gain or loss on branch *b*ʹ:

$$\begin{array}{lll} x_{ij} & \;= & g\prime {}_{ij}\;l\prime {}_{ij}+\left(1-g\prime {}_{ij}\right)\cdot l\prime {}_{ij}+\left(1-l\prime {}_{ij}\right)\cdot g\prime {}_{ij}\forall i\in B\prime ,\forall j\in P\\ & \;= & g\prime {}_{ij}+\left(1-g\prime {}_{ij}\right)\cdot l\prime {}_{ij} \end{array}$$

$$y_{i}={g\prime }_{ip}+\left(1-g_{ip}\prime \right)\cdot {l\prime }_{ip}\forall i\in B\prime .$$

(ii) Let *x*_*i*_ be a vector representing the probabilities *x*ʹ_*ij*_ for all Pfam families j∈P on branch *b*_*i*_. We discarded any samples (*x*_*i*_, *y*_*i*_) that had a probability for a phenotype gain or loss (*y*_*i*_) above the reporting threshold of GLOOME but below a threshold (*t*). We set the threshold *t* to 0.5.

This defines the matrix *X* and the vector *y* as follows:

$$\left(X,y)=\{(x_{i,}y_{\text{i},})\;|\;y_{i}=0\vee y_{i}\geq t,i\in B\prime \right\}$$

By this means, we avoided presenting the classifier with samples corresponding to uncertain phenotype gain or loss events and used only confident labels in the subsequent classifier training instead.

(iii) We inferred discrete phenotype labels *y*ʹ by applying this threshold *t* to the joint probability *y*_*i*_ for a phenotype gain or loss to set up a well-defined classification problem with a binary target variable. Whenever the probability for a phenotype to be gained or lost on a specific branch was larger than *t*, the event was considered to have happened as follows:

$$y\prime =\{\begin{array}{l} 1,if\;y_{i}\geq t\\ 0,otherwise \end{array}\forall i\in B\prime$$

(iv) Finally, we formulated a joint binary classification problem for each target phenotype *yp* and the corresponding gain and loss events *y*ʹ the phyletic patterns *XP*, and the Pfam gain and loss events *X*, which we solved again with a linear L1-regularized L2-loss SVM. We applied this procedure for all of the GIDEON phenotypes under investigation.

### Software requirements.

Traitar can be run on a standard laptop with Linux/Unix. The run time (wall clock time) for annotating and phenotyping a typical microbial genome with 3 Mbp is 9 min (3 min/Mbp) on an Intel Core i5-2410M dual-core processor with 2.30 GHz, requiring only a few megabytes of memory.

### Cross-validation.

We employed cross-validation to assess the performance of the classifiers individually for each phenotype. For a given phenotype, we divided the bacterial samples that were annotated with that phenotype into 10 folds. Each fold was selected once for testing the model, which was trained on the remaining folds. The optimal regularization parameter *C* needed to be determined independently in each step of the cross-validation; therefore, we employed a further inner cross-validation by using the following range of values for the parameter *C*: 10^−3^,* *10^−2^ ⋅ 0.7,* *10^−2^ ⋅ 0.5,* *10^−2^ ⋅ 0.2, * *10^−2^ ⋅ 0.1,......,1. In other words, for each fold kept out for testing in the outer cross-validation, we determined the value of the parameter *C* that gave the best accuracy in an additional 10-fold cross-validation on the remaining folds. This value was then used to train the SVM model in the current outer cross-validation step. Whenever we proceeded to a new cross-validation fold, we recomputed the ancestral character state reconstruction of the phenotype with only the training samples included (see ancestral protein family and phenotype gains and losses above). This procedure is known as nested cross-validation (62).

The bacterial samples in the training folds imply a Gps-sTOL in each step of the inner and outer cross-validation without the samples in the test fold. We used the same procedure as before to map the Pfam gains and losses inferred previously on the Gps-sTOL onto the tree defined by the current cross-validation training folds. Importantly, the test error is only estimated on the observed phenotype labels rather than on the inferred phenotype gains and losses.

### Evaluation metrics.

We used evaluation metrics from multilabel classification theory for performance evaluation (63). We determined the performance for the individual phenotype-positive and the phenotype-negative classes based on the confusion matrix of true-positive (*TP*), true-negative (*TN*), false-negative (*FN*), and false-positive (*FP*) samples from their binary classification equivalents by averaging over all *n* phenotypes. We utilized two different accuracy measures to assess multiclass classification performance (i.e., the accuracy pooled over all classification decisions and the macroaccuracy). Macroaccuracy represents an average over the accuracy of the individual binary classification problems, and we computed this from the macrorecall of the phenotype-positive and phenotype-negative classes as follows:

$${Macrorecall}_{Pos}=\left(\overset{n}{\underset{i\;=\;1}{\sum }}\frac{TP_{i}}{TP_{i}+FN_{i}}\right)/n$$

$$Macrorecall_{Neg}=\left(\overset{n}{\underset{i\;=\;1}{\sum }}\frac{TN_{i}}{FP_{i}+TN_{i}}\right)/n$$

$$Macroaccuracy=\left(Macrorecall_{Pos}+Macrorecall_{Neg}\right)/2$$

However, if there are only few available labels for some phenotypes, the variance of the macroaccuracy will be high and this measure cannot be reliably computed anymore; it cannot be computed at all if no labels are available. The accuracy only assesses the overall classification performance without consideration of the information about specific phenotypes. Large classes dominate small classes (63).

$$Recall_{Pos}=\frac{\sum_{i\;=\;1}^{n}TP_{i}}{\sum_{i\;=\;1}^{n}TP_{i}+\sum_{i\;=\;1}^{n}FN_{i}}$$

$$Recall_{Neg}=\frac{\sum_{i\;=\;1}^{n}TN_{i}}{\sum_{i\;=\;1}^{n}TN_{i}+\sum_{i\;=\;1}^{n}FP_{i}}$$

$$Accuracy=\left(Recall_{Pos}+Recall_{Neg}\right)/2$$

### Majority feature selection.

The weights in linear SVMs can be directly linked to features that are relevant for the classification. We identified the most important protein families used as features from the voting committee of SVMs consisting of the five most accurate models, which were also used to classify new samples. If the majority, which is at least three predictors, included a positive value for a given protein family, we added this feature to the list of important features. We further ranked these protein family features by their correlation with the phenotype by using Pearson’s correlation coefficient (see Table S6 in the supplemental material).

10.1128/mSystems.00101-16.6 Table S6  Pfam rankings by their correlation with each of the 67 phenotypes for both classifiers using Pearson’s correlation coefficient. Download Table S6, PDF file, 1.5 MB. Copyright © 2016 Weimann et al. 2016 Weimann et al. This content is distributed under the terms of the Creative Commons Attribution 4.0 International license.
