# Supplementary material for: From Genomes to Phenotypes: Traitar, the Microbial Trait Analyzer
Source: mSystems. 2016 Dec 27;1(6):e00101-16. doi: 10.1128/mSystems.00101-16 (PMC5192078; doi:10.1128/mSystems.00101-16)
Supplement: Table S1 [file sys006162072st1.pdf]

Supplementary table S1 Detailed information on the 67 phenotypes used in this study

Phenotype(a)	Test type(b)	Test description(c)	GIDEON I+(e)	GIDEON I-(d)	GIDEON I total(f)	GIDEON II+(h)	GIDEON II-(g)	GIDEON II total(i)	Bergey's+(k)	Bergey's-(l)	Bergey's total(m)
Acetate utilization	General test	A variety of commercial kits are satisfactory. Includes late reactions for gram-positive and non-fermentative gram-negative rods	27	19	46	5	2	7	7	10	17
Aerobe	Basic test	Organisms which grow only in the presence of air.	64	167	231	7	35	42	25	0	25
Alkaline phosphatase	General test	Most kits utilize p-nitrophenyl phosphate as substrate. Insure thorough washing if phosphate buffers are employed	30	15	45	7	3	10	12	21	33
Anaerobe	Basic test	Organisms which do not grow in the presence of oxygen	46	169	215	14	27	41	16	11	27
Arginine dihydrolase	General test	For most organisms, Moeller medium interpreted with control after 18 hours (or longer for gram-positive and non-fermentative gram-negative rods)	46	88	134	8	15	23	35	55	90
Bacillus or coccobacillus	Basic test	Bacilli predominate	160	48	208	28	12	40	0	0	0
Beta hemolysis	General test	Sheep blood	18	134	152	4	17	21	4	5	9

Bile-susceptible	General test	Media and bile concentrations vary according to the species tested; Presumpto Plates useful for anaerobes	16	35	51	2	7	9	0	0	0
Capnophilic	General test	Exogenous carbon dioxide (5 to 7%) must be present for growth; Gas Pak or cylinder gas are preferred	22	175	197	2	33	35	0	0	0
Casein hydrolysis	General test	Standard skim-milk/nutrient agar halo test; Presumpto Plates useful for anaerobes	13	47	60	2	7	9	19	19	38
Catalase	Basic test	Perform on young colonies (up to 24 hours) using 3% hydrogen peroxide (alternative technique for Mycobacteria); Presumpto Plates useful for anaerobes	123	91	214	18	20	38	34	32	66
Cellobiose	Fermentation or acidification	Commercial phenol red techniques usually acceptable; Andrades or more sensitive indicators necessary for organisms with more subtle pH changes. Includes late reactions for gram positive & non-fermentative gram negative rods	33	63	96	4	7	11	42	50	92

Citrate	General test	Simmons citrate medium using a light inoculum (avoid stabbing the agar). Includes late reactions for gram-positive and non-fermentative gram-negative rods	35	81	116	11	16	27	16	19	35
Coagulase production	General test	Standard or commercial slide plasma tests acceptable; tube tests may help differentiate Staphylococcus aureus from other taxa	30	45	75	3	7	10	2	3	5
Coccus	Basic test	Cocci predominate.	44	170	214	12	28	40	0	0	0
Coccus - clusters or groups predominate	Basic test	The predominant forms are cocci, in clusters or irregular groups.	12	204	216	2	39	41	0	0	0
Coccus - pairs or chains predominate	Basic test	The predominant forms are cocci in chains or pairs.	28	185	213	10	30	40	0	0	0
Colistin-Polymyxin susceptible	General test	Standard disk diffusion technique; recommended media and disk potency may vary for specific taxa	38	55	93	3	11	14	0	0	0

D-Mannitol	Fermentation or acidification	Commercial phenol red techniques usually acceptable; Andrades or more sensitive indicators necessary for organisms with more subtle pH changes. Includes late reactions for gram positive & non-fermentative gram negative rods	53	128	181	12	19	31	49	58	107
D-Mannose	Fermentation or acidification	Commercial phenol red techniques usually acceptable; Andrades or more sensitive indicators necessary for organisms with more subtle pH changes. Includes late reactions for gram positive & non-fermentative gram negative rods	85	60	145	14	12	26	70	20	90
DNase	General test	Standard commercial agar tests; Presumpto Plates useful for anaerobes	15	71	86	2	9	11	2	0	2

D-Sorbitol	Fermentation or acidification	Commercial phenol red techniques usually acceptable; Andrades or more sensitive indicators necessary for organisms with more subtle pH changes. Includes late reactions for gram positive & non-fermentative gram negative rods	18	127	145	5	24	29	18	60	78
D-Xylose	Fermentation or acidification	Commercial phenol red techniques usually acceptable; Andrades or more sensitive indicators necessary for organisms with more subtle pH changes. Includes late reactions for gram positive & non-fermentative gram negative rods	44	118	162	10	22	32	32	77	109
Esculin hydrolysis	General test	For most organisms, Moeller medium interpreted with control after 18 hours (or longer for gram-positive and non-fermentative gram-negative rods)	52	93	145	17	12	29	49	31	80
Facultative	Basic test	Organisms which grow both in the presence and absence of air.	97	130	227	19	23	42	0	0	0

Gas from glucose	General test	Gas produced from D-glucose; Durham tube or gas bubbles noted in commercial kits	22	106	128	3	15	18	4	6	10
Gelatin hydrolysis	General test	Commercial or self prepared (or X-ray film) tests interpreted after 24-48 hours against control at lowered temperature; Presumpto Plates useful for anaerobes	30	111	141	3	19	22	30	31	61
Glucose fermenter	Basic test	Commercial phenol red techniques are generally acceptable; Andrades or more sensitive indicators necessary for organisms producing more subtle pH changes; specific acidification techniques applied for Neisseria; Presumpto Plates useful for anaerobes	131	78	209	26	9	35	0	0	0
Glucose oxidizer	Basic test	Hugh and Leifson method; in most cases 'positive' indicates nonfermentative organism which oxidizes glucose	22	192	214	2	38	40	0	0	0

Glycerol	Fermentation or acidification	Commercial phenol red techniques usually acceptable; Andrades or more sensitive indicators necessary for organisms with more subtle pH changes. Includes late reactions for gram positive & non-fermentative gram negative rods	24	86	110	8	14	22	28	31	59
Gram negative	Basic test	Gram-negative forms predominate	117	110	227	17	24	41	0	0	0
Gram positive	Basic test	Gram-positive forms predominate	100	127	227	23	18	41	14	8	22
Growth at 42 degrees C	General test	Media vary according to the species tested	43	27	70	10	2	12	0	4	4
Growth in 6.5% NaCl	General test	Media vary according to the species tested	29	67	96	6	9	15	0	0	0
Growth in KCN	General test	Commercial kits based on 1:13,000 KCN are suggested	10	14	24	2	0	2	18	10	28
Growth on MacConkey agar	Basic test	Visible growth within 48 hours; or within 7 days for gram positive and non-fermentative gram negative rods	55	165	220	7	31	38	0	2	2
Growth on ordinary blood agar	Basic test	Visible growth on sheep blood agar within 48 hours	211	18	229	39	3	42	0	0	0

Hydrogen sulfide	General test	TSI for enterobacteriaceae and most other species; Presumpto Plates useful for anaerobes. Includes late appearance of hydrogen sulfide for gram-positive and non-fermentative gram-negative rods	13	116	129	4	16	20	0	2	2
Indole	General test	A spot test is acceptable for most organisms; overnight testing with a paper strip is helpful in confirming negative reactions; Presumpto Plates useful for anaerobes	20	145	165	2	24	26	18	47	65
Lactose	Fermentation or acidification	Commercial phenol red techniques usually acceptable; Andrades or more sensitive indicators necessary for organisms with more subtle pH changes. Includes late reactions for gram positive & non-fermentative gram negative rods	61	104	165	11	17	28	48	55	103

L-Arabinose	Fermentation or acidification	Commercial phenol red techniques usually acceptable; Andrades or more sensitive indicators necessary for organisms with more subtle pH changes. Includes late reactions for gram positive & non-fermentative gram negative rods	44	114	158	8	23	31	24	84	108
Lipase	General test	Standard egg yolk agar test; Presumpto Plates useful for anaerobes	20	76	96	2	13	15	5	9	14
L-Rhamnose	Fermentation or acidification	Commercial phenol red techniques usually acceptable; Andrades or more sensitive indicators necessary for organisms with more subtle pH changes. Includes late reactions for gram positive & non-fermentative gram negative rods	20	109	129	2	24	26	10	43	53
Lysine decarboxylase	General test	For most organisms, Moeller medium interpreted with control after 18 hours (or longer for gram-positive and non-fermentative gram-negative rods)	11	63	74	4	5	9	6	20	26

Malonate	General test	Standard test based on maintenance of alkaline pH (bromthymol blue) in the presence of glucose and malonate; commercial kits are acceptable	11	29	40	1	4	5	0	0	0
Maltose	Fermentation or acidification	Commercial phenol red techniques usually acceptable; Andrades or more sensitive indicators necessary for organisms with more subtle pH changes. Includes late reactions for gram positive & non-fermentative gram negative rods	105	66	171	18	9	27	86	32	118
Melibiose	Fermentation or acidification	Commercial phenol red techniques usually acceptable; Andrades or more sensitive indicators necessary for organisms with more subtle pH changes. Includes late reactions for gram positive & non-fermentative gram negative rods	25	88	113	9	18	27	18	45	63
Methyl red	General test	Commercial or self-prepared media are generally acceptable	23	29	52	2	5	7	5	7	12

Motile	General test	Standard hanging drop on fresh broth isolates for most purposes; perform at 22 to 25 degrees C if Listeria suspected	70	144	214	11	26	37	60	32	92
Mucate utilization	General test	A variety of commercial kits are satisfactory. Includes late reactions for gram-positive and non-fermentative gram-negative rods	11	23	34	2	6	8	3	8	11
myo-Inositol	Fermentation or acidification	Commercial phenol red techniques usually acceptable; Andrades or more sensitive indicators necessary for organisms with more subtle pH changes. Includes late reactions for gram positive & non-fermentative gram negative rods	10	107	117	5	16	21	3	29	32
Nitrate to nitrite	General test	Commercial and self-prepared media are acceptable; alternative techniques used for mycobacteria	75	92	167	8	19	27	31	43	74
Nitrite to gas	General test	Standard zinc dust test applied to 'nitrate-negative' organisms	14	76	90	1	11	12	0	0	0

ONPG (beta galactosidase)	General test	Commercial kits are generally satisfactory; suggest a heavy inoculum in buffered medium; yellow pigmented organisms may not be suitable for testing	45	70	115	8	9	17	4	11	15
Ornithine decarboxylase	General test	For most organisms, Moeller medium interpreted with control after 18 hours (or longer for gram-positive and non-fermentative gram-negative rods)	17	67	84	2	5	7	13	23	36
Oxidase	Basic test	Paper strip test from appropriate media	56	130	186	5	24	29	19	19	38
Pyrrolidonyl-beta-naphthylamide	General test	L-pyrrolidonyl-beta-naphthylamide - PYR (or pyrrolidonyl arylamidase - PYRA) - reagents commercially available; read color within 10 seconds (2 minutes for Carr-Scarborough reagent)	23	56	79	5	10	15	0	0	0

Raffinose	Fermentation or acidification	Commercial phenol red techniques usually acceptable; Andrades or more sensitive indicators necessary for organisms with more subtle pH changes. Includes late reactions for gram positive & non-fermentative gram negative rods	30	107	137	10	17	27	23	66	89
Salicin	Fermentation or acidification	Commercial phenol red techniques usually acceptable; Andrades or more sensitive indicators necessary for organisms with more subtle pH changes. Includes late reactions for gram positive & non-fermentative gram negative rods	41	93	134	9	12	21	28	24	52
Spore formation	Basic test	Note that spores may only appear in vitro, and may not be seen in clinical material	18	216	234	2	40	42	3	2	5
Starch hydrolysis	General test	Standard starch hydrolysis or Mueller-Hilton agar tests developed with iodine solutions; Presumpto Plates useful for anaerobes	27	65	92	5	15	20	38	44	82

Sucrose	Fermentation or acidification	Commercial phenol red techniques usually acceptable; Andrades or more sensitive indicators necessary for organisms with more subtle pH changes. Includes late reactions for gram positive & non-fermentative gram negative rods	84	93	177	20	11	31	85	44	129
Tartrate utilization	General test	A variety of commercial kits are satisfactory. Includes late reactions for gram-positive and non-fermentative gram-negative rods	10	18	28	2	3	5	4	3	7
Trehalose	Fermentation or acidification	Commercial phenol red techniques usually acceptable; Andrades or more sensitive indicators necessary for organisms with more subtle pH changes. Includes late reactions for gram positive & non-fermentative gram negative rods	75	63	138	14	15	29	66	42	108

Urea hydrolysis	General test	Christensen agar testing for most taxa; other techniques for mycobacteria, ureaplasma and other organisms as recommended. Includes late reactions for gram-positive and non-fermentative gram-negative rods	28	127	155	7	22	29	15	56	71
Voges Proskauer	General test	Commercial or self-prepared media are generally acceptable; the test is most reliable when performed on cultures no older than three days	28	68	96	8	8	16	10	19	29
Yellow pigment	General test	Yellow pigment noted on sheep blood or other primary isolation agar. Includes late appearance of pigment for gram-positive and non-fermentative gram-negative rods	13	161	174	1	28	29	1	4	5

(a) GIDEON phenotypes
(b) Type of test required for the phenotype determination in the wet lab according to GIDEON
(c) Remarks on wet lab test for determination of the phenotype according to GIDEON
(d) Number of phenotype-positive bacteria in the GIDEON I dataset
(e) Number of phenotype-negative bacteria in the GIDEON I dataset
(f) Total number of bacteria with phenotype labels in the GIDEON I dataset
(g) Number of phenotype-positive bacteria in the GIDEON II dataset
(h) Number of phenotype-negative bacteria in the GIDEON II dataset
(i) Total number of bacteria with phenotype labels in the GIDEON II dataset

(j) Number of phenotype-positive bacteria in the Bergey dataset
(k) Number of phenotype-negative bacteria in the Bergey dataset
(l) Total number of bacteria with phenotype labels in the Bergey dataset
