# Supplementary material for: From Genomes to Phenotypes: Traitar, the Microbial Trait Analyzer
Source: mSystems. 2016 Dec 27;1(6):e00101-16. doi: 10.1128/mSystems.00101-16 (PMC5192078; doi:10.1128/mSystems.00101-16)
Supplement: Table S2 [file sys006162072st2.pdf]

Supplementary Table S2 Macro-accuracy of the phypat and phypat+PGL classifiers obtained in cross-validation experiments for the 67 GIDEON phenotypes

Phenotype(a)	phypat+PGL(b)	phypat(c)
Spore formation	1	0.887
Methyl red	1	0.905
Gram positive	0.996	1
Gram negative	0.991	0.987
Growth on MacConkey agar	0.988	0.97
Anaerobe	0.986	0.959
Catalase	0.984	0.985
Aerobe	0.972	0.98
Coccus - pairs or chains predominate	0.903	0.974
Coagulase production	0.972	0.944
Glucose fermenter	0.966	0.897
Glucose oxidizer	0.962	0.949
Oxidase	0.962	0.937
Motile	0.958	0.912
Nitrate to nitrite	0.954	0.926
Cellobiose	0.946	0.877
Growth in 6.5% NaCl	0.943	0.862
Bacillus or coccobacillus	0.939	0.811
Coccus	0.879	0.931
L-Arabinose	0.904	0.923
Sucrose	0.922	0.825
Urea hydrolysis	0.889	0.921
Esculin hydrolysis	0.915	0.846
D-Xylose	0.905	0.857
Citrate	0.904	0.765
Gelatin hydrolysis	0.902	0.817
Glycerol	0.864	0.769
Trehalose	0.899	0.825
D-Mannitol	0.828	0.897
Facultative	0.862	0.896

ONPG (beta galactosidase)	0.883	0.857
Nitrite to gas	0.879	0.84
D-Sorbitol	0.842	0.877
Voges Proskauer	0.876	0.866
Acetate utilization	0.75	0.876
Malonate	0.875	0.806
Melibiose	0.875	0.769
Raffinose	0.873	0.749
Coccus - clusters or groups predominate	0.816	0.87
Capnophilic	0.747	0.869
Lipase	0.867	0.824
D-Mannose	0.865	0.866
Salicin	0.861	0.789
Colistin-Polymyxin susceptible	0.861	0.844
Beta hemolysis	0.855	0.765
Lactose	0.854	0.847
Maltose	0.854	0.794
Casein hydrolysis	0.814	0.846
L-Rhamnose	0.781	0.84
Growth on ordinary blood agar	0.833	0.691
Ornithine decarboxylase	0.83	0.755
Pyrrolidonyl-beta-naphthylamide	0.829	0.767
Growth at 42 degrees C	0.613	0.801
Gas from glucose	0.788	0.794
Starch hydrolysis	0.793	0.793
Growth in KCN	0.793	0.679
Indole	0.728	0.792
Lysine decarboxylase	0.6	0.737
Mucate utilization	0.775	0.662
Arginine dihydrolase	0.743	0.766
Bile-susceptible	0.761	0.681
Alkaline phosphatase	0.633	0.75
Hydrogen sulfide	0.734	0.559

Tartrate utilization	0.694	0.328
Yellow pigment	0.688	0.613
myo-Inositol	0.68	0.639
DNase	0.658	0.677

(a) Phenotypes sorted by the maximal macro-accuracy determined from a 10-fold nested cross-validation from 234 bacteria described in the Global Infectious Disease and Epidemiology Online Network

(b) Macro accuracy for the phypat+PGL classifier

(c) Macro accuracy for the phypat classifier
