# Supplementary material for: From Genomes to Phenotypes: Traitar, the Microbial Trait Analyzer
Source: mSystems. 2016 Dec 27;1(6):e00101-16. doi: 10.1128/mSystems.00101-16 (PMC5192078; doi:10.1128/mSystems.00101-16)
Supplement: Table S3 [file sys006162072st3.pdf]

Supplementary Table S3 Mapping of bacterial strains to 234 species described in the Global Infectious Disease and Epidemiology Online Network with links to the National Center for Biotechnology Information (NCBI) databases

Strain(a)	Species(b)	Bioproject id(c)	NCBI taxonomy id(d)
Acholeplasma laidlawii PG-8A	Acholeplasma laidlawii	19259	441768
Achromobacter xylosoxidans A8	Achromobacter xylosoxidans	762376	59899
Acidaminococcus fermentans DSM 20731	Acidaminococcus fermentans	591001	43471
Acidaminococcus intestini RyC-MR95	Acidaminococcus intestini	568816	74445
Acidovorax avenae subsp. avenae ATCC 19860	Acidovorax avenae	643561	42497
Acinetobacter baumannii 1656-2	Acinetobacter baumannii	400667	58731
Acinetobacter baumannii AB0057	Acinetobacter baumannii	405416	58765
Acinetobacter baumannii AB307-0294	Acinetobacter baumannii	480119	59083
Acinetobacter baumannii ACICU	Acinetobacter baumannii	497978	158685
Acinetobacter baumannii ATCC 17978	Acinetobacter baumannii	509170	61601
Acinetobacter baumannii AYE	Acinetobacter baumannii	509173	61637
Acinetobacter baumannii MDR-TJ	Acinetobacter baumannii	557600	59271
Acinetobacter baumannii MDR-ZJ06	Acinetobacter baumannii	696749	158677
Acinetobacter baumannii SDF	Acinetobacter baumannii	889738	162739
Acinetobacter baumannii TCDC-AB0715	Acinetobacter baumannii	980514	158679
Acinetobacter calcoaceticus PHEA-2	Acinetobacter calcoaceticus	871585	83123
Aerococcus urinae ACS-120-V-Col10a	Aerococcus urinae	866775	64757
Aeromonas hydrophila subsp. hydrophila ATCC 7966	Aeromonas hydrophila	380703	58617
Aeromonas salmonicida subsp. salmonicida A449	Aeromonas salmonicida	382245	58631
Aggregatibacter actinomycetemcomitans ANH9381	Aggregatibacter actinomycetemcomitans	694569	46989
Aggregatibacter actinomycetemcomitans D7S-1	Aggregatibacter actinomycetemcomitans	754507	80743
Aggregatibacter aphrophilus NJ8700	Aggregatibacter aphrophilus	634176	59407
Anaerococcus prevotii DSM 20548	Anaerococcus prevotii	525919	59219
Arcanobacterium haemolyticum DSM 20595	Arcanobacterium haemolyticum	644284	49489
Arcobacter butzleri ED-1	Arcobacter butzleri	367737	58557
Arcobacter butzleri RM4018	Arcobacter butzleri	944546	158699
Arthrobacter aurescens TC1	Arthrobacter aurescens	290340	58109
Atopobium parvulum DSM 20469	Atopobium parvulum	521095	59195
Bacillus anthracis str. A0248	Bacillus anthracis	198094	57909
Bacillus anthracis str. Ames	Bacillus anthracis	260799	58091
Bacillus anthracis str. 'Ames Ancestor'	Bacillus anthracis	261594	58083
Bacillus anthracis str. CDC 684	Bacillus anthracis	568206	59303

Bacillus anthracis str. H9401	Bacillus anthracis	592021	59385
Bacillus anthracis str. Sterne	Bacillus anthracis	768494	162021
Bacillus cereus 03BB102	Bacillus cereus	222523	57673
Bacillus cereus AH187	Bacillus cereus	226900	57975
Bacillus cereus AH820	Bacillus cereus	288681	58103
Bacillus cereus ATCC 10987	Bacillus cereus	334406	82815
Bacillus cereus ATCC 14579	Bacillus cereus	347495	83611
Bacillus cereus B4264	Bacillus cereus	361100	58529
Bacillus cereus biovar anthracis str. CI	Bacillus cereus	405531	58759
Bacillus cereus E33L	Bacillus cereus	405532	58757
Bacillus cereus F837/76	Bacillus cereus	405534	58753
Bacillus cereus G9842	Bacillus cereus	405535	58751
Bacillus cereus NC7401	Bacillus cereus	572264	59299
Bacillus cereus Q1	Bacillus cereus	637380	50615
Bacillus coagulans 2-6	Bacillus coagulans	345219	54335
Bacillus coagulans 36D1	Bacillus coagulans	941639	68053
Bacillus licheniformis DSM 13 = ATCC 14580	Bacillus licheniformis	279010	58097
Bacillus megaterium DSM 319	Bacillus megaterium	1006007	159841
Bacillus megaterium WSH-002	Bacillus megaterium	592022	48371
Bacillus pumilus SAFR-032	Bacillus pumilus	315750	59017
Bacillus subtilis BSn5	Bacillus subtilis	1052585	73967
Bacillus subtilis subsp. spizizenii str. W23	Bacillus subtilis	1052588	158879
Bacillus subtilis subsp. spizizenii TU-B-10	Bacillus subtilis	224308	57675
Bacillus subtilis subsp. subtilis str. 168	Bacillus subtilis	655816	51879
Bacillus subtilis subsp. subtilis str. RO-NN-1	Bacillus subtilis	936156	62463
Bacillus thuringiensis BMB171	Bacillus thuringiensis	281309	58089
Bacillus thuringiensis serovar chinensis CT-43	Bacillus thuringiensis	412694	58795
Bacillus thuringiensis serovar finitimus YBT-020	Bacillus thuringiensis	541229	158151
Bacillus thuringiensis serovar konkukian str. 97-27	Bacillus thuringiensis	714359	49135
Bacillus thuringiensis str. Al Hakam	Bacillus thuringiensis	930170	158875
Bacteroides fragilis 638R	Bacteroides fragilis	272559	57639
Bacteroides fragilis NCTC 9343	Bacteroides fragilis	295405	58195
Bacteroides fragilis YCH46	Bacteroides fragilis	862962	84217
Bacteroides thetaiotaomicron VPI-5482	Bacteroides thetaiotaomicron	226186	62913
Bacteroides vulgatus ATCC 8482	Bacteroides vulgatus	435590	58253
Bartonella bacilliformis KC583	Bartonella bacilliformis	360095	58533
Bartonella clarridgeiae 73	Bartonella clarridgeiae	696125	62131

Bartonella grahamii as4aup	Bartonella grahamii	634504	59405
Bartonella henselae str. Houston-1	Bartonella henselae	283166	57745
Bartonella quintana str. Toulouse	Bartonella quintana	283165	57635
Bifidobacterium adolescentis ATCC 15703	Bifidobacterium adolescentis	367928	58559
Bifidobacterium bifidum BGN4	Bifidobacterium bifidum	484020	167988
Bifidobacterium bifidum PRL2010	Bifidobacterium bifidum	702459	59883
Bifidobacterium bifidum S17	Bifidobacterium bifidum	883062	59545
Bifidobacterium breve ACS-071-V-Sch8b	Bifidobacterium breve	866777	158863
Bifidobacterium dentium Bd1	Bifidobacterium dentium	401473	43091
Bifidobacterium longum DJO10A	Bifidobacterium longum	1035817	158861
Bifidobacterium longum NCC2705	Bifidobacterium longum	205913	58833
Bifidobacterium longum subsp. infantis 157F	Bifidobacterium longum	206672	57939
Bifidobacterium longum subsp. infantis ATCC 15697 = JCM 1222	Bifidobacterium longum	391904	159865
Bifidobacterium longum subsp. longum BBMN68	Bifidobacterium longum	565040	62693
Bifidobacterium longum subsp. longum JCM 1217	Bifidobacterium longum	565042	62695
Bifidobacterium longum subsp. longum JDM301	Bifidobacterium longum	759350	49131
Bifidobacterium longum subsp. longum KACC 91563	Bifidobacterium longum	890402	60163
Bordetella avium 197N	Bordetella avium	360910	61563
Bordetella bronchiseptica RB50	Bordetella bronchiseptica	257310	57613
Bordetella parapertussis 12822	Bordetella parapertussis	257311	57615
Bordetella pertussis CS	Bordetella pertussis	1017264	158859
Bordetella pertussis Tohama I	Bordetella pertussis	257313	57617
Bordetella petrii DSM 12804	Bordetella petrii	340100	61631
Brachyspira pilosicoli 95/1000	Brachyspira pilosicoli	759914	50609
Brevibacillus brevis NBRC 100599	Brevibacillus brevis	358681	59175
Brucella abortus A13334	Brucella abortus	1104320	83615
Brucella abortus bv. 1 str. 9-941	Brucella abortus	262698	58019
Brucella abortus S19	Brucella abortus	430066	58873
Brucella canis ATCC 23365	Brucella canis	1104321	83613
Brucella canis HSK A52141	Brucella canis	483179	59009
Brucella melitensis ATCC 23457	Brucella melitensis	1029825	158853
Brucella melitensis biovar Abortus 2308	Brucella melitensis	224914	57735
Brucella melitensis bv. 1 str. 16M	Brucella melitensis	359391	62937
Brucella melitensis M28	Brucella melitensis	546272	59241
Brucella melitensis M5-90	Brucella melitensis	703352	158855
Brucella melitensis NI	Brucella melitensis	941967	158857
Brucella suis 1330	Brucella suis	1112912	83617

Brucella suis ATCC 23445	Brucella suis	204722	159871
Brucella suis VBI22	Brucella suis	470137	59015
Burkholderia ambifaria AMMD	Burkholderia ambifaria	339670	58303
Burkholderia ambifaria MC40-6	Burkholderia ambifaria	398577	58701
Burkholderia cenocepacia AU 1054	Burkholderia cenocepacia	216591	57953
Burkholderia cenocepacia HI2424	Burkholderia cenocepacia	331271	58371
Burkholderia cenocepacia J2315	Burkholderia cenocepacia	331272	58369
Burkholderia cenocepacia MC0-3	Burkholderia cenocepacia	406425	58769
Burkholderia gladioli BSR3	Burkholderia gladioli	999541	66301
Burkholderia mallei ATCC 23344	Burkholderia mallei	243160	57725
Burkholderia mallei NCTC 10229	Burkholderia mallei	320388	58387
Burkholderia mallei NCTC 10247	Burkholderia mallei	320389	58385
Burkholderia mallei SAVP1	Burkholderia mallei	412022	58383
Burkholderia multivorans ATCC 17616	Burkholderia multivorans	395019	58697
Burkholderia pseudomallei 1026b	Burkholderia pseudomallei	272560	57733
Burkholderia pseudomallei 1106a	Burkholderia pseudomallei	320372	58391
Burkholderia pseudomallei 1710b	Burkholderia pseudomallei	320373	58389
Burkholderia pseudomallei 668	Burkholderia pseudomallei	357348	58515
Burkholderia pseudomallei K96243	Burkholderia pseudomallei	884204	162511
Burkholderia thailandensis E264	Burkholderia thailandensis	271848	58081
Burkholderia vietnamiensis G4	Burkholderia vietnamiensis	269482	58075
Campylobacter concisus 13826	Campylobacter concisus	360104	58667
Campylobacter curvus 525.92	Campylobacter curvus	360105	58669
Campylobacter hominis ATCC BAA-381	Campylobacter hominis	360107	58981
Campylobacter lari RM2100	Campylobacter lari	306263	58115
Capnocytophaga canimorsus Cc5	Capnocytophaga canimorsus	860228	70727
Capnocytophaga ochracea DSM 7271	Capnocytophaga ochracea	521097	59197
Chromobacterium violaceum ATCC 12472	Chromobacterium violaceum	243365	58001
Citrobacter koseri ATCC BAA-895	Citrobacter koseri	290338	58143
Citrobacter rodentium ICC168	Citrobacter rodentium	637910	43089
Clostridium beijerinckii NCIMB 8052	Clostridium beijerinckii	290402	58137
Clostridium botulinum A2 str. Kyoto	Clostridium botulinum	413999	61579
Clostridium botulinum A3 str. Loch Maree	Clostridium botulinum	441770	58927
Clostridium botulinum A str. ATCC 19397	Clostridium botulinum	441771	58931
Clostridium botulinum A str. ATCC 3502	Clostridium botulinum	441772	58929
Clostridium botulinum A str. Hall	Clostridium botulinum	498213	59147
Clostridium botulinum B1 str. Okra	Clostridium botulinum	498214	59149

Clostridium botulinum Ba4 str. 657	Clostridium botulinum	508765	59159
Clostridium botulinum BKT015925	Clostridium botulinum	508767	59157
Clostridium botulinum B str. Eklund 17B	Clostridium botulinum	515621	59173
Clostridium botulinum E3 str. Alaska E43	Clostridium botulinum	536232	59229
Clostridium botulinum F str. 230613	Clostridium botulinum	758678	159513
Clostridium botulinum F str. Langeland	Clostridium botulinum	929506	66203
Clostridium botulinum H04402 065	Clostridium botulinum	941968	162091
Clostridium difficile 630	Clostridium difficile	272563	57679
Clostridium difficile BI1	Clostridium difficile	645462	41017
Clostridium difficile CD196	Clostridium difficile	645463	40921
Clostridium difficile R20291	Clostridium difficile	699034	158363
Clostridium perfringens ATCC 13124	Clostridium perfringens	195102	57681
Clostridium perfringens SM101	Clostridium perfringens	195103	57901
Clostridium perfringens str. 13	Clostridium perfringens	289380	58117
Clostridium tetani E88	Clostridium tetani	212717	57683
Comamonas testosteroni CNB-2	Comamonas testosteroni	688245	62961
Corynebacterium aurimucosum ATCC 700975	Corynebacterium aurimucosum	548476	59409
Corynebacterium diphtheriae 241	Corynebacterium diphtheriae	257309	57691
Corynebacterium diphtheriae 31A	Corynebacterium diphtheriae	698962	84309
Corynebacterium diphtheriae BH8	Corynebacterium diphtheriae	698963	84313
Corynebacterium diphtheriae C7 (beta)	Corynebacterium diphtheriae	698964	84303
Corynebacterium diphtheriae CDCE 8392	Corynebacterium diphtheriae	698965	84295
Corynebacterium diphtheriae HC01	Corynebacterium diphtheriae	698966	83607
Corynebacterium diphtheriae HC02	Corynebacterium diphtheriae	698967	84297
Corynebacterium diphtheriae HC03	Corynebacterium diphtheriae	698968	84317
Corynebacterium diphtheriae HC04	Corynebacterium diphtheriae	698969	84299
Corynebacterium diphtheriae INCA 402	Corynebacterium diphtheriae	698970	84301
Corynebacterium diphtheriae NCTC 13129	Corynebacterium diphtheriae	698971	84305
Corynebacterium diphtheriae PW8	Corynebacterium diphtheriae	698972	83605
Corynebacterium diphtheriae VA01	Corynebacterium diphtheriae	698973	84311
Corynebacterium jeikeium K411	Corynebacterium jeikeium	306537	58399
Corynebacterium kroppenstedtii DSM 44385	Corynebacterium kroppenstedtii	645127	59411
Corynebacterium pseudotuberculosis 1002	Corynebacterium pseudotuberculosis	1074485	89381
Corynebacterium pseudotuberculosis 1/06-A	Corynebacterium pseudotuberculosis	1087451	162167
Corynebacterium pseudotuberculosis 258	Corynebacterium pseudotuberculosis	1087452	83609
Corynebacterium pseudotuberculosis 267	Corynebacterium pseudotuberculosis	1087453	159669
Corynebacterium pseudotuberculosis 31	Corynebacterium pseudotuberculosis	1087454	159665

Corynebacterium pseudotuberculosis 316	Corynebacterium pseudotuberculosis	1089446	162175
Corynebacterium pseudotuberculosis 3/99-5	Corynebacterium pseudotuberculosis	1117942	157909
Corynebacterium pseudotuberculosis 42/02-A	Corynebacterium pseudotuberculosis	1161911	168258
Corynebacterium pseudotuberculosis C231	Corynebacterium pseudotuberculosis	1168865	167260
Corynebacterium pseudotuberculosis CIP 52.97	Corynebacterium pseudotuberculosis	679896	159677
Corynebacterium pseudotuberculosis Cp162	Corynebacterium pseudotuberculosis	681645	159675
Corynebacterium pseudotuberculosis FRC41	Corynebacterium pseudotuberculosis	765874	50585
Corynebacterium pseudotuberculosis I19	Corynebacterium pseudotuberculosis	889513	159673
Corynebacterium pseudotuberculosis P54B96	Corynebacterium pseudotuberculosis	935298	159671
Corynebacterium pseudotuberculosis PAT10	Corynebacterium pseudotuberculosis	935697	159667
Corynebacterium resistens DSM 45100	Corynebacterium resistens	662755	50555
Corynebacterium ulcerans 0102	Corynebacterium ulcerans	945711	159659
Corynebacterium ulcerans 809	Corynebacterium ulcerans	945712	68291
Corynebacterium ulcerans BR-AD22	Corynebacterium ulcerans	996634	169879
Corynebacterium urealyticum DSM 7109	Corynebacterium urealyticum	504474	61639
Cronobacter sakazakii ATCC BAA-894	Cronobacter sakazakii	1138308	167045
Cronobacter sakazakii ES15	Cronobacter sakazakii	290339	58145
Cronobacter turicensis z3032	Cronobacter turicensis	693216	40821
Cryptobacterium curtum DSM 15641	Cryptobacterium curtum	469378	59041
Cupriavidus metallidurans CH34	Cupriavidus metallidurans	266264	57815
Desulfovibrio desulfuricans ND132	Desulfovibrio desulfuricans	525146	59213
Desulfovibrio desulfuricans subsp. desulfuricans str. ATCC 27774	Desulfovibrio desulfuricans	641491	63159
Desulfovibrio vulgaris DP4	Desulfovibrio vulgaris	391774	58679
Desulfovibrio vulgaris RCH1	Desulfovibrio vulgaris	573059	161961
Desulfovibrio vulgaris str. Hildenborough	Desulfovibrio vulgaris	882	57645
Desulfovibrio vulgaris str. 'Miyazaki F'	Desulfovibrio vulgaris	883	59089
Dichelobacter nodosus VCS1703A	Dichelobacter nodosus	246195	57643
Edwardsiella tarda EIB202	Edwardsiella tarda	498217	41819
Edwardsiella tarda FL6-60	Edwardsiella tarda	718251	159657
Eggerthella lenta DSM 2243	Eggerthella lenta	479437	59079
Enterobacter aerogenes KCTC 2190	Enterobacter aerogenes	1028307	68103
Enterobacter asburiae LF7a	Enterobacter asburiae	640513	72793
Enterococcus faecalis D32	Enterococcus faecalis	1206105	171261
Enterococcus faecalis OG1RF	Enterococcus faecalis	226185	57669
Enterococcus faecalis V583	Enterococcus faecalis	474186	54927
Enterococcus faecium Aus0004	Enterococcus faecium	1155766	87025
Enterococcus faecium DO	Enterococcus faecium	333849	55353

Enterococcus hirae ATCC 9790	Enterococcus hirae	768486	70619
Escherichia coli 042	Escherichia coli	1033813	162139
Escherichia coli 536	Escherichia coli	1048689	162153
Escherichia coli 55989	Escherichia coli	1072459	162115
Escherichia coli ABU 83972	Escherichia coli	155864	57831
Escherichia coli APEC O1	Escherichia coli	199310	57915
Escherichia coli ATCC 8739	Escherichia coli	216592	161985
Escherichia coli BL21(DE3)	Escherichia coli	316385	58979
Escherichia coli 'BL21-Gold(DE3)pLysS AG'	Escherichia coli	316401	161993
Escherichia coli B str. REL606	Escherichia coli	316407	161931
Escherichia coli BW2952	Escherichia coli	331111	58395
Escherichia coli CFT073	Escherichia coli	331112	58393
Escherichia coli DH1	Escherichia coli	362663	58531
Escherichia coli E24377A	Escherichia coli	364106	58541
Escherichia coli ED1a	Escherichia coli	386585	57781
Escherichia coli ETEC H10407	Escherichia coli	405955	58623
Escherichia coli HS	Escherichia coli	409438	59425
Escherichia coli IAI1	Escherichia coli	413997	58803
Escherichia coli IAI39	Escherichia coli	431946	161939
Escherichia coli IHE3034	Escherichia coli	439855	58919
Escherichia coli KO11FL	Escherichia coli	444450	59091
Escherichia coli LF82	Escherichia coli	469008	161947
Escherichia coli NA114	Escherichia coli	481805	58783
Escherichia coli O103:H2 str. 12009	Escherichia coli	511145	57779
Escherichia coli O111:H- str. 11128	Escherichia coli	536056	161951
Escherichia coli O127:H6 str. E2348/69	Escherichia coli	544404	59235
Escherichia coli O157:H7 str. EC4115	Escherichia coli	566546	162011
Escherichia coli O157:H7 str. EDL933	Escherichia coli	573235	41021
Escherichia coli O157:H7 str. Sakai	Escherichia coli	574521	59343
Escherichia coli O157:H7 str. TW14359	Escherichia coli	585034	59377
Escherichia coli O26:H11 str. 11368	Escherichia coli	585035	62979
Escherichia coli O55:H7 str. CB9615	Escherichia coli	585055	59383
Escherichia coli O55:H7 str. RM12579	Escherichia coli	585056	62981
Escherichia coli O7:K1 str. CE10	Escherichia coli	585057	59381
Escherichia coli O83:H1 str. NRG 857C	Escherichia coli	585395	41013
Escherichia coli P12b	Escherichia coli	585396	41023
Escherichia coli S88	Escherichia coli	585397	59379

Escherichia coli SE11	Escherichia coli	591946	161965
Escherichia coli SE15	Escherichia coli	595495	162099
Escherichia coli SMS-3-5	Escherichia coli	595496	59391
Escherichia coli str. 'clone D i14'	Escherichia coli	655817	161975
Escherichia coli str. 'clone D i2'	Escherichia coli	685038	161987
Escherichia coli str. K-12 substr. DH10B	Escherichia coli	696406	161991
Escherichia coli str. K-12 substr. MG1655	Escherichia coli	701177	46655
Escherichia coli str. K-12 substr. W3110	Escherichia coli	714962	162007
Escherichia coli UM146	Escherichia coli	741093	163995
Escherichia coli UMN026	Escherichia coli	866768	59245
Escherichia coli UMNK88	Escherichia coli	869729	162043
Escherichia coli UTI89	Escherichia coli	885275	162049
Escherichia coli W	Escherichia coli	885276	162047
Escherichia coli Xuzhou21	Escherichia coli	910348	162061
Escherichia fergusonii ATCC 35469	Escherichia fergusonii	585054	59375
Eubacterium eligens ATCC 27750	Eubacterium eligens	515620	59171
Eubacterium limosum KIST612	Eubacterium limosum	903814	59777
Eubacterium rectale ATCC 33656	Eubacterium rectale	515619	59169
Fibrobacter succinogenes subsp. succinogenes S85	Fibrobacter succinogenes	59374	161919
Filifactor alocis ATCC 35896	Filifactor alocis	546269	46625
Finegoldia magna ATCC 29328	Finegoldia magna	334413	58867
Francisella philomiragia subsp. philomiragia ATCC 25017	Francisella philomiragia	484022	59105
Francisella tularensis subsp. holarctica FTNF002-00	Francisella tularensis	1001534	89373
Francisella tularensis subsp. holarctica LVS	Francisella tularensis	1001542	89379
Francisella tularensis subsp. holarctica OSU18	Francisella tularensis	177416	57589
Francisella tularensis subsp. mediasiatica FSC147	Francisella tularensis	376619	58595
Francisella tularensis subsp. tularensis FSC198	Francisella tularensis	393011	58687
Francisella tularensis subsp. tularensis NE061598	Francisella tularensis	393115	58693
Francisella tularensis subsp. tularensis SCHU S4	Francisella tularensis	418136	58811
Francisella tularensis subsp. tularensis TI0902	Francisella tularensis	441952	58939
Francisella tularensis subsp. tularensis TIGB03	Francisella tularensis	458234	58999
Francisella tularensis subsp. tularensis WY96-3418	Francisella tularensis	510831	161973
Fusobacterium nucleatum subsp. nucleatum ATCC 25586	Fusobacterium nucleatum	190304	57885
Gardnerella vaginalis 409-05	Gardnerella vaginalis	1009464	162045
Gardnerella vaginalis ATCC 14019	Gardnerella vaginalis	525284	55487
Gardnerella vaginalis HMP9231	Gardnerella vaginalis	553190	43211
Gordonia bronchialis DSM 43247	Gordonia bronchialis	526226	41403

Gordonia polyisoprenivorans VH2	Gordonia polyisoprenivorans	1112204	86651
Haemophilus ducreyi 35000HP	Haemophilus ducreyi	233412	57625
Haemophilus influenzae 10810	Haemophilus influenzae	262727	161921
Haemophilus influenzae 86-028NP	Haemophilus influenzae	262728	161923
Haemophilus influenzae F3031	Haemophilus influenzae	281310	58093
Haemophilus influenzae F3047	Haemophilus influenzae	374930	58591
Haemophilus influenzae PittEE	Haemophilus influenzae	374931	58593
Haemophilus influenzae PittGG	Haemophilus influenzae	71421	57771
Haemophilus influenzae R2846	Haemophilus influenzae	862964	86647
Haemophilus influenzae R2866	Haemophilus influenzae	866630	62123
Haemophilus influenzae Rd KW20	Haemophilus influenzae	935897	62097
Haemophilus parainfluenzae T3T1	Haemophilus parainfluenzae	862965	72801
Helicobacter bizzozeronii CIII-1	Helicobacter bizzozeronii	1002804	68141
Helicobacter cinaedi PAGU611	Helicobacter cinaedi	1172562	162219
Helicobacter felis ATCC 49179	Helicobacter felis	936155	61409
Helicobacter pylori 2017	Helicobacter pylori	102608	161139
Helicobacter pylori 2018	Helicobacter pylori	1055527	158157
Helicobacter pylori 26695	Helicobacter pylori	1055528	159611
Helicobacter pylori 35A	Helicobacter pylori	1055529	161157
Helicobacter pylori 51	Helicobacter pylori	1055530	159615
Helicobacter pylori 83	Helicobacter pylori	1127122	165869
Helicobacter pylori 908	Helicobacter pylori	1163739	162205
Helicobacter pylori B38	Helicobacter pylori	1163740	162207
Helicobacter pylori B8	Helicobacter pylori	1163741	162209
Helicobacter pylori Cuz20	Helicobacter pylori	1163742	162211
Helicobacter pylori ELS37	Helicobacter pylori	1163743	162213
Helicobacter pylori F16	Helicobacter pylori	290847	161925
Helicobacter pylori F30	Helicobacter pylori	357544	58517
Helicobacter pylori F32	Helicobacter pylori	512562	59165
Helicobacter pylori F57	Helicobacter pylori	563041	59305
Helicobacter pylori G27	Helicobacter pylori	570508	59327
Helicobacter pylori Gambia94/24	Helicobacter pylori	585535	49903
Helicobacter pylori HPAG1	Helicobacter pylori	585538	161153
Helicobacter pylori HUP-B14	Helicobacter pylori	592205	59415
Helicobacter pylori India7	Helicobacter pylori	637913	159639
Helicobacter pylori J99	Helicobacter pylori	693745	49873
Helicobacter pylori Lithuania75	Helicobacter pylori	765962	53541

Helicobacter pylori P12	Helicobacter pylori	765963	53539
Helicobacter pylori PeCan18	Helicobacter pylori	765964	159987
Helicobacter pylori PeCan4	Helicobacter pylori	794851	159467
Helicobacter pylori Puno120	Helicobacter pylori	85962	178201
Helicobacter pylori Puno135	Helicobacter pylori	85963	57789
Helicobacter pylori Sat464	Helicobacter pylori	866344	161145
Helicobacter pylori Shi112	Helicobacter pylori	866345	159991
Helicobacter pylori Shi169	Helicobacter pylori	866346	161143
Helicobacter pylori Shi417	Helicobacter pylori	869727	159985
Helicobacter pylori Shi470	Helicobacter pylori	907237	159491
Helicobacter pylori SJM180	Helicobacter pylori	907238	161149
Helicobacter pylori SNT49	Helicobacter pylori	907239	159989
Helicobacter pylori SouthAfrica7	Helicobacter pylori	907240	159493
Helicobacter pylori v225d	Helicobacter pylori	985080	161159
Helicobacter pylori XZ274	Helicobacter pylori	985081	161151
Klebsiella oxytoca E718	Klebsiella oxytoca	1006551	83159
Klebsiella oxytoca KCTC 1686	Klebsiella oxytoca	1191061	170256
Klebsiella variicola At-22	Klebsiella variicola	640131	42113
Kocuria rhizophila DC2201	Kocuria rhizophila	378753	59099
Kytococcus sedentarius DSM 20547	Kytococcus sedentarius	478801	59071
Lactobacillus acidophilus 30SC	Lactobacillus acidophilus	272621	57685
Lactobacillus acidophilus NCFM	Lactobacillus acidophilus	891391	63605
Lactobacillus brevis ATCC 367	Lactobacillus brevis	387344	57989
Lactobacillus buchneri NRRL B-30929	Lactobacillus buchneri	511437	66205
Lactobacillus casei ATCC 334	Lactobacillus casei	321967	57985
Lactobacillus casei BD-II	Lactobacillus casei	498216	50673
Lactobacillus casei BL23	Lactobacillus casei	543734	59237
Lactobacillus casei LC2W	Lactobacillus casei	998820	162119
Lactobacillus casei str. Zhang	Lactobacillus casei	999378	162121
Lactobacillus crispatus ST1	Lactobacillus crispatus	748671	48359
Lactobacillus fermentum CECT 5716	Lactobacillus fermentum	334390	58865
Lactobacillus fermentum IFO 3956	Lactobacillus fermentum	712938	162003
Lactobacillus gasseri ATCC 33323	Lactobacillus gasseri	324831	57687
Lactobacillus johnsonii DPC 6026	Lactobacillus johnsonii	257314	58029
Lactobacillus johnsonii FI9785	Lactobacillus johnsonii	633699	41735
Lactobacillus johnsonii NCC 533	Lactobacillus johnsonii	909954	162057
Lactobacillus plantarum JDM1	Lactobacillus plantarum	220668	62911

Lactobacillus plantarum subsp. plantarum ST-III	Lactobacillus plantarum	644042	59361
Lactobacillus plantarum WCFS1	Lactobacillus plantarum	889932	53537
Lactobacillus reuteri DSM 20016	Lactobacillus reuteri	491077	55357
Lactobacillus reuteri JCM 1112	Lactobacillus reuteri	557433	58875
Lactobacillus reuteri SD2112	Lactobacillus reuteri	557436	58471
Lactobacillus rhamnosus ATCC 8530	Lactobacillus rhamnosus	1088720	162169
Lactobacillus rhamnosus GG	Lactobacillus rhamnosus	568703	161983
Lactobacillus rhamnosus Lc 705	Lactobacillus rhamnosus	568704	59315
Lactobacillus salivarius CECT 5713	Lactobacillus salivarius	362948	58233
Lactobacillus salivarius UCC118	Lactobacillus salivarius	712961	162005
Lactococcus garvieae ATCC 49156	Lactococcus garvieae	420889	73413
Lactococcus garvieae Lg2	Lactococcus garvieae	420890	161935
Laribacter hongkongensis HLHK9	Laribacter hongkongensis	557598	59265
Legionella longbeachae NSW150	Legionella longbeachae	661367	46099
Legionella pneumophila 2300/99 Alcoy	Legionella pneumophila	272624	57609
Legionella pneumophila str. Corby	Legionella pneumophila	297245	58209
Legionella pneumophila str. Lens	Legionella pneumophila	297246	58211
Legionella pneumophila str. Paris	Legionella pneumophila	400673	58733
Legionella pneumophila subsp. pneumophila ATCC 43290	Legionella pneumophila	423212	48801
Legionella pneumophila subsp. pneumophila str. Philadelphia 1	Legionella pneumophila	933093	86885
Leptospira interrogans serovar Copenhageni str. Fiocruz L1-130	Leptospira interrogans	189518	57881
Leptospira interrogans serovar Lai str. 56601	Leptospira interrogans	267671	58065
Leptospira interrogans serovar Lai str. IPAV	Leptospira interrogans	573825	161957
Leptotrichia buccalis C-1013-b	Leptotrichia buccalis	523794	59211
Leuconostoc citreum KM20	Leuconostoc citreum	349519	58481
Leuconostoc mesenteroides subsp. mesenteroides ATCC 8293	Leuconostoc mesenteroides	1107880	84337
Leuconostoc mesenteroides subsp. mesenteroides J18	Leuconostoc mesenteroides	203120	57919
Listeria innocua Clip11262	Listeria innocua	272626	61567
Listeria monocytogenes 07PF0776	Listeria monocytogenes	1030009	162131
Listeria monocytogenes 08-5578	Listeria monocytogenes	1126011	162185
Listeria monocytogenes 08-5923	Listeria monocytogenes	169963	61583
Listeria monocytogenes 10403S	Listeria monocytogenes	265669	57689
Listeria monocytogenes EGD-e	Listeria monocytogenes	393126	54441
Listeria monocytogenes Finland 1998	Listeria monocytogenes	393127	54443
Listeria monocytogenes FSL R2-561	Listeria monocytogenes	393130	54459
Listeria monocytogenes HCC23	Listeria monocytogenes	393133	54461
Listeria monocytogenes J0161	Listeria monocytogenes	552536	59203

Listeria monocytogenes M7	Listeria monocytogenes	568819	59317
Listeria monocytogenes serotype 4b str. CLIP 80459	Listeria monocytogenes	637381	43727
Listeria monocytogenes serotype 4b str. F2365	Listeria monocytogenes	653938	43671
Listeria seeligeri serovar 1/2b str. SLCC3954	Listeria seeligeri	683837	46215
Listeria welshimeri serovar 6b str. SLCC5334	Listeria welshimeri	386043	61605
Lysinibacillus sphaericus C3-41	Lysinibacillus sphaericus	444177	58945
Micrococcus luteus NCTC 2665	Micrococcus luteus	465515	59033
Mobiluncus curtisii ATCC 43063	Mobiluncus curtisii	548479	49695
Moraxella catarrhalis RH4	Moraxella catarrhalis	749219	48809
Mycobacterium leprae Br4923	Mycobacterium leprae	272631	57697
Mycobacterium leprae TN	Mycobacterium leprae	561304	59293
Mycoplasma fermentans JER	Mycoplasma fermentans	637387	53543
Mycoplasma fermentans M64	Mycoplasma fermentans	943945	62099
Mycoplasma genitalium G37	Mycoplasma genitalium	243273	57707
Mycoplasma penetrans HF-2	Mycoplasma penetrans	272633	57729
Mycoplasma pneumoniae 309	Mycoplasma pneumoniae	1112856	85495
Mycoplasma pneumoniae FH	Mycoplasma pneumoniae	272634	57709
Mycoplasma pneumoniae M129	Mycoplasma pneumoniae	722438	162027
Neisseria gonorrhoeae FA 1090	Neisseria gonorrhoeae	242231	57611
Neisseria gonorrhoeae NCCP11945	Neisseria gonorrhoeae	521006	59191
Neisseria gonorrhoeae TCDC-NG08107	Neisseria gonorrhoeae	940296	161097
Neisseria lactamica 020-06	Neisseria lactamica	489653	60851
Neisseria meningitidis 053442	Neisseria meningitidis	122586	57817
Neisseria meningitidis 8013	Neisseria meningitidis	122587	57819
Neisseria meningitidis alpha14	Neisseria meningitidis	272831	57825
Neisseria meningitidis alpha710	Neisseria meningitidis	374833	58587
Neisseria meningitidis FAM18	Neisseria meningitidis	604162	161967
Neisseria meningitidis G2136	Neisseria meningitidis	630588	161971
Neisseria meningitidis H44/76	Neisseria meningitidis	662598	61649
Neisseria meningitidis M01-240149	Neisseria meningitidis	909420	162083
Neisseria meningitidis M01-240355	Neisseria meningitidis	935588	162075
Neisseria meningitidis M04-240196	Neisseria meningitidis	935589	162077
Neisseria meningitidis MC58	Neisseria meningitidis	935591	162079
Neisseria meningitidis NZ-05/33	Neisseria meningitidis	935593	162081
Neisseria meningitidis WUE 2594	Neisseria meningitidis	935599	162085
Neisseria meningitidis Z2491	Neisseria meningitidis	942513	162093
Nocardia farcinica IFM 10152	Nocardia farcinica	247156	58203

Nocardiopsis dassonvillei subsp. dassonvillei DSM 43111	Nocardiopsis dassonvillei	446468	49483
Ochrobactrum anthropi ATCC 49188	Ochrobactrum anthropi	439375	58921
Odoribacter splanchnicus DSM 220712	Odoribacter splanchnicus	709991	63397
Olsenella uli DSM 7084	Olsenella uli	633147	51367
Paenibacillus polymyxa E681	Paenibacillus polymyxa	1052684	162159
Paenibacillus polymyxa M1	Paenibacillus polymyxa	349520	53477
Paenibacillus polymyxa SC2	Paenibacillus polymyxa	886882	59583
Parabacteroides distasonis ATCC 8503	Parabacteroides distasonis	435591	58301
Pasteurella multocida 36950	Pasteurella multocida	1075089	86887
Pasteurella multocida subsp. multocida str. 3480	Pasteurella multocida	1132496	156881
Pasteurella multocida subsp. multocida str. HN06	Pasteurella multocida	272843	57627
Pasteurella multocida subsp. multocida str. Pm70	Pasteurella multocida	584721	161955
Pediococcus pentosaceus ATCC 25745	Pediococcus pentosaceus	278197	57981
Porphyromonas asaccharolytica DSM 20707	Porphyromonas asaccharolytica	879243	66603
Porphyromonas gingivalis ATCC 33277	Porphyromonas gingivalis	1030843	67407
Porphyromonas gingivalis TDC60	Porphyromonas gingivalis	242619	57641
Porphyromonas gingivalis W83	Porphyromonas gingivalis	431947	58879
Prevotella denticola F0289	Prevotella denticola	767031	65091
Prevotella intermedia 17	Prevotella intermedia	246198	163151
Prevotella melaninogenica ATCC 25845	Prevotella melaninogenica	553174	51377
Propionibacterium acnes 266	Propionibacterium acnes	1031709	162137
Propionibacterium acnes 6609	Propionibacterium acnes	1091045	162177
Propionibacterium acnes ATCC 11828	Propionibacterium acnes	1114966	80745
Propionibacterium acnes KPA171202	Propionibacterium acnes	1114967	80735
Propionibacterium acnes SK137	Propionibacterium acnes	1114969	80733
Propionibacterium acnes TypeIA2 P.acn17	Propionibacterium acnes	267747	58101
Propionibacterium acnes TypeIA2 P.acn31	Propionibacterium acnes	553199	48071
Propionibacterium acnes TypeIA2 P.acn33	Propionibacterium acnes	909952	162059
Proteus mirabilis HI4320	Proteus mirabilis	529507	61599
Providencia stuartii MRSN 2154	Providencia stuartii	1157951	162193
Pseudomonas aeruginosa DK2	Pseudomonas aeruginosa	1089456	162173
Pseudomonas aeruginosa LESB58	Pseudomonas aeruginosa	1093787	168996
Pseudomonas aeruginosa M18	Pseudomonas aeruginosa	208963	57977
Pseudomonas aeruginosa NCGM2.S1	Pseudomonas aeruginosa	208964	57945
Pseudomonas aeruginosa PA7	Pseudomonas aeruginosa	381754	58627
Pseudomonas aeruginosa PAO1	Pseudomonas aeruginosa	557722	59275
Pseudomonas aeruginosa UCBPP-PA14	Pseudomonas aeruginosa	941193	162089

Pseudomonas fluorescens A506	Pseudomonas fluorescens	1037911	165185
Pseudomonas fluorescens F113	Pseudomonas fluorescens	1114970	87037
Pseudomonas fluorescens Pf0-1	Pseudomonas fluorescens	205922	57591
Pseudomonas fluorescens SBW25	Pseudomonas fluorescens	216595	158693
Pseudomonas mendocina NK-01	Pseudomonas mendocina	1001585	66299
Pseudomonas mendocina ymp	Pseudomonas mendocina	399739	58723
Pseudomonas putida BIRD-1	Pseudomonas putida	1042876	68747
Pseudomonas putida DOT-T1E	Pseudomonas putida	1196325	171260
Pseudomonas putida F1	Pseudomonas putida	160488	57843
Pseudomonas putida GB-1	Pseudomonas putida	231023	167583
Pseudomonas putida KT2440	Pseudomonas putida	351746	58355
Pseudomonas putida ND6	Pseudomonas putida	390235	58651
Pseudomonas putida S16	Pseudomonas putida	76869	58735
Pseudomonas putida W619	Pseudomonas putida	931281	162055
Pseudomonas stutzeri A1501	Pseudomonas stutzeri	1123519	170940
Pseudomonas stutzeri ATCC 17588 = LMG 11199	Pseudomonas stutzeri	1196835	168379
Pseudomonas stutzeri CCUG 29243	Pseudomonas stutzeri	379731	58641
Pseudomonas stutzeri DSM 10701	Pseudomonas stutzeri	96563	68749
Pseudomonas stutzeri DSM 4166	Pseudomonas stutzeri	996285	162113
Rahnella aquatilis CIP 78.65 = ATCC 33071	Rahnella aquatilis	1151116	158049
Rahnella aquatilis HX2	Rahnella aquatilis	745277	86855
Ralstonia pickettii 12D	Ralstonia pickettii	402626	58737
Ralstonia pickettii 12J	Ralstonia pickettii	428406	58859
Rhodococcus equi 103S	Rhodococcus equi	685727	60171
Rhodococcus erythropolis PR4	Rhodococcus erythropolis	234621	59019
Roseburia hominis A2-183	Roseburia hominis	585394	73419
Rothia dentocariosa ATCC 17931	Rothia dentocariosa	762948	49331
Rothia mucilaginosa DY-18	Rothia mucilaginosa	680646	43093
Salmonella bongori NCTC 12419	Salmonella bongori	218493	70155
Sebaldella termitidis ATCC 33386	Sebaldella termitidis	526218	41865
Selenomonas sputigena ATCC 35185	Selenomonas sputigena	546271	55329
Serratia plymuthica AS9	Serratia plymuthica	768492	67313
Shigella boydii CDC 3083-94	Shigella boydii	300268	58215
Shigella boydii Sb227	Shigella boydii	344609	58415
Shigella dysenteriae Sd197	Shigella dysenteriae	300267	58213
Shigella flexneri 2002017	Shigella flexneri	198214	62907
Shigella flexneri 2a str. 2457T	Shigella flexneri	198215	57991

Shigella flexneri 2a str. 301	Shigella flexneri	373384	58583
Shigella flexneri 5 str. 8401	Shigella flexneri	591020	159233
Shigella sonnei Ss046	Shigella sonnei	300269	58217
Slackia heliotrinireducens DSM 20476	Slackia heliotrinireducens	471855	59051
Staphylococcus aureus 04-02981	Staphylococcus aureus	1006543	159861
Staphylococcus aureus RF122	Staphylococcus aureus	1028799	88071
Staphylococcus aureus subsp. aureus 11819-97	Staphylococcus aureus	1074252	162163
Staphylococcus aureus subsp. aureus 71193	Staphylococcus aureus	1118959	88065
Staphylococcus aureus subsp. aureus COL	Staphylococcus aureus	1123523	159981
Staphylococcus aureus subsp. aureus ECT-R 2	Staphylococcus aureus	1155084	162141
Staphylococcus aureus subsp. aureus ED133	Staphylococcus aureus	158878	57835
Staphylococcus aureus subsp. aureus ED98	Staphylococcus aureus	158879	57837
Staphylococcus aureus subsp. aureus HO 5096 0412	Staphylococcus aureus	196620	57903
Staphylococcus aureus subsp. aureus JH1	Staphylococcus aureus	273036	57661
Staphylococcus aureus subsp. aureus JH9	Staphylococcus aureus	282458	57839
Staphylococcus aureus subsp. aureus JKD6159	Staphylococcus aureus	282459	57841
Staphylococcus aureus subsp. aureus LGA251	Staphylococcus aureus	359786	58455
Staphylococcus aureus subsp. aureus M013	Staphylococcus aureus	359787	58457
Staphylococcus aureus subsp. aureus MRSA252	Staphylococcus aureus	418127	58817
Staphylococcus aureus subsp. aureus MSHR1132	Staphylococcus aureus	426430	58839
Staphylococcus aureus subsp. aureus MSSA476	Staphylococcus aureus	451515	58555
Staphylococcus aureus subsp. aureus Mu3	Staphylococcus aureus	451516	58925
Staphylococcus aureus subsp. aureus Mu50	Staphylococcus aureus	546342	159855
Staphylococcus aureus subsp. aureus MW2	Staphylococcus aureus	548473	159859
Staphylococcus aureus subsp. aureus N315	Staphylococcus aureus	663951	159241
Staphylococcus aureus subsp. aureus NCTC 8325	Staphylococcus aureus	681288	41455
Staphylococcus aureus subsp. aureus str. JKD6008	Staphylococcus aureus	685039	159689
Staphylococcus aureus subsp. aureus str. Newman	Staphylococcus aureus	703339	161969
Staphylococcus aureus subsp. aureus T0131	Staphylococcus aureus	869816	159691
Staphylococcus aureus subsp. aureus TCH60	Staphylococcus aureus	889933	159389
Staphylococcus aureus subsp. aureus TW20	Staphylococcus aureus	93061	57795
Staphylococcus aureus subsp. aureus USA300_FPR3757	Staphylococcus aureus	93062	57797
Staphylococcus aureus subsp. aureus USA300_TCH1516	Staphylococcus aureus	985002	89393
Staphylococcus aureus subsp. aureus VC40	Staphylococcus aureus	985006	159391
Staphylococcus carnosus subsp. carnosus TM300	Staphylococcus carnosus	396513	59401
Staphylococcus epidermidis ATCC 12228	Staphylococcus epidermidis	176279	57663
Staphylococcus epidermidis RP62A	Staphylococcus epidermidis	176280	57861

Staphylococcus haemolyticus JCSC1435	Staphylococcus haemolyticus	279808	62919
Staphylococcus lugdunensis HKU09-01	Staphylococcus lugdunensis	1034809	162143
Staphylococcus lugdunensis N920143	Staphylococcus lugdunensis	698737	46233
Staphylococcus pseudintermedius ED99	Staphylococcus pseudintermedius	937773	62125
Staphylococcus pseudintermedius HKU10-03	Staphylococcus pseudintermedius	984892	162109
Staphylococcus saprophyticus subsp. saprophyticus ATCC 15305	Staphylococcus saprophyticus	342451	58411
Stenotrophomonas maltophilia D457	Stenotrophomonas maltophilia	1163399	162199
Stenotrophomonas maltophilia JV3	Stenotrophomonas maltophilia	391008	58657
Stenotrophomonas maltophilia K279a	Stenotrophomonas maltophilia	522373	61647
Stenotrophomonas maltophilia R551-3	Stenotrophomonas maltophilia	868597	72473
Streptobacillus moniliformis DSM 12112	Streptobacillus moniliformis	519441	41863
Streptococcus agalactiae 2603V/R	Streptococcus agalactiae	205921	57935
Streptococcus agalactiae A909	Streptococcus agalactiae	208435	57943
Streptococcus agalactiae NEM316	Streptococcus agalactiae	211110	61585
Streptococcus dysgalactiae subsp. equisimilis ATCC 12394	Streptococcus dysgalactiae	486410	59103
Streptococcus dysgalactiae subsp. equisimilis GGS_124	Streptococcus dysgalactiae	663954	161979
Streptococcus equi subsp. equi 4047	Streptococcus equi	40041	59261
Streptococcus equi subsp. zooepidemicus	Streptococcus equi	552526	59263
Streptococcus equi subsp. zooepidemicus MGCS10565	Streptococcus equi	553482	59259
Streptococcus gallolyticus subsp. gallolyticus ATCC 43143	Streptococcus gallolyticus	637909	46061
Streptococcus gallolyticus subsp. gallolyticus ATCC BAA-2069	Streptococcus gallolyticus	981539	162103
Streptococcus gallolyticus UCN34	Streptococcus gallolyticus	990317	63617
Streptococcus gordonii str. Challis substr. CH1	Streptococcus gordonii	467705	57667
Streptococcus infantarius subsp. infantarius CJ18	Streptococcus infantarius	1069533	87033
Streptococcus intermedius JTH08	Streptococcus intermedius	591365	168614
Streptococcus mitis B6	Streptococcus mitis	365659	46097
Streptococcus oralis Uo5	Streptococcus oralis	927666	65449
Streptococcus pasteurianus ATCC 43144	Streptococcus pasteurianus	981540	68019
Streptococcus pneumoniae 670-6B	Streptococcus pneumoniae	1130804	162191
Streptococcus pneumoniae 70585	Streptococcus pneumoniae	170187	57857
Streptococcus pneumoniae AP200	Streptococcus pneumoniae	171101	57859
Streptococcus pneumoniae ATCC 700669	Streptococcus pneumoniae	189423	52533
Streptococcus pneumoniae CGSP14	Streptococcus pneumoniae	373153	58581
Streptococcus pneumoniae D39	Streptococcus pneumoniae	487213	59119
Streptococcus pneumoniae G54	Streptococcus pneumoniae	487214	59117
Streptococcus pneumoniae Hungary19A-6	Streptococcus pneumoniae	488221	59125
Streptococcus pneumoniae INV104	Streptococcus pneumoniae	488222	59121

Streptococcus pneumoniae INV200	Streptococcus pneumoniae	488223	59123
Streptococcus pneumoniae JJA	Streptococcus pneumoniae	512566	59167
Streptococcus pneumoniae OXC141	Streptococcus pneumoniae	516950	59181
Streptococcus pneumoniae P1031	Streptococcus pneumoniae	525381	49735
Streptococcus pneumoniae R6	Streptococcus pneumoniae	561276	59287
Streptococcus pneumoniae ST556	Streptococcus pneumoniae	574093	52453
Streptococcus pneumoniae Taiwan19F-14	Streptococcus pneumoniae	869215	162037
Streptococcus pneumoniae TCH8431/19A	Streptococcus pneumoniae	869216	162035
Streptococcus pneumoniae TIGR4	Streptococcus pneumoniae	869269	162039
Streptococcus pseudopneumoniae IS7493	Streptococcus pseudopneumoniae	1054460	71153
Streptococcus pyogenes Alab49	Streptococcus pyogenes	1010840	158061
Streptococcus pyogenes M1 GAS	Streptococcus pyogenes	160490	57845
Streptococcus pyogenes MGAS10270	Streptococcus pyogenes	160491	57847
Streptococcus pyogenes MGAS10394	Streptococcus pyogenes	186103	57871
Streptococcus pyogenes MGAS10750	Streptococcus pyogenes	193567	57895
Streptococcus pyogenes MGAS15252	Streptococcus pyogenes	198466	57911
Streptococcus pyogenes MGAS1882	Streptococcus pyogenes	286636	58105
Streptococcus pyogenes MGAS2096	Streptococcus pyogenes	293653	58337
Streptococcus pyogenes MGAS315	Streptococcus pyogenes	319701	58335
Streptococcus pyogenes MGAS5005	Streptococcus pyogenes	370551	58569
Streptococcus pyogenes MGAS6180	Streptococcus pyogenes	370552	58571
Streptococcus pyogenes MGAS8232	Streptococcus pyogenes	370553	58573
Streptococcus pyogenes MGAS9429	Streptococcus pyogenes	370554	58575
Streptococcus pyogenes NZ131	Streptococcus pyogenes	471876	59035
Streptococcus pyogenes SSI-1	Streptococcus pyogenes	487215	162171
Streptococcus pyogenes str. Manfredo	Streptococcus pyogenes	798300	158037
Streptococcus salivarius 57.I	Streptococcus salivarius	1046629	162151
Streptococcus salivarius JIM8777	Streptococcus salivarius	347253	162145
Streptococcus uberis 0140J	Streptococcus uberis	218495	57959
Streptomyces griseus subsp. griseus NBRC 13350	Streptomyces griseus	455632	58983
Treponema denticola ATCC 35405	Treponema denticola	243275	57583
Treponema pallidum subsp. pallidum DAL-1	Treponema pallidum	243276	208669
Treponema pallidum subsp. pallidum SS14	Treponema pallidum	455434	58977
Treponema pallidum subsp. pallidum str. Chicago	Treponema pallidum	491078	87069
Treponema pallidum subsp. pallidum str. Nichols	Treponema pallidum	491079	87051
Treponema pallidum subsp. pertenue str. CDC2	Treponema pallidum	491080	87067
Treponema pallidum subsp. pertenue str. Gauthier	Treponema pallidum	491081	87065

Treponema pallidum subsp. pertenue str. SamoaD	Treponema pallidum	666714	159543
Tropheryma whipplei str. Twist	Tropheryma whipplei	203267	57705
Tropheryma whipplei TW08/27	Tropheryma whipplei	218496	57961
Tsukamurella paurometabola DSM 20162	Tsukamurella paurometabola	521096	48829
Ureaplasma parvum serovar 3 str. ATCC 27815	Ureaplasma parvum	273119	57711
Ureaplasma parvum serovar 3 str. ATCC 700970	Ureaplasma parvum	505682	58887
Ureaplasma urealyticum serovar 10 str. ATCC 33699	Ureaplasma urealyticum	565575	59011
Veillonella parvula DSM 2008	Veillonella parvula	479436	41927
Vibrio cholerae IEC224	Vibrio cholerae	1134456	89389
Vibrio cholerae LMA3984-4	Vibrio cholerae	243277	57623
Vibrio cholerae M66-2	Vibrio cholerae	345073	159869
Vibrio cholerae MJ-1236	Vibrio cholerae	579112	59355
Vibrio cholerae O1 biovar El Tor str. N16961	Vibrio cholerae	593588	59387
Vibrio cholerae O1 str. 2010EL-1786	Vibrio cholerae	914149	78933
Vibrio cholerae O395	Vibrio cholerae	935297	159541
Vibrio furnissii NCTC 11218	Vibrio furnissii	903510	82347
Vibrio parahaemolyticus RIMD 2210633	Vibrio parahaemolyticus	223926	57969
Vibrio vulnificus CMCP6	Vibrio vulnificus	196600	58007
Vibrio vulnificus MO6-24/O	Vibrio vulnificus	216895	62909
Vibrio vulnificus YJ016	Vibrio vulnificus	914127	62243
Weeksella virosa DSM 16922	Weeksella virosa	865938	63627
Wolinella succinogenes DSM 1740	Wolinella succinogenes	273121	61591
Yersinia enterocolitica subsp. enterocolitica 8081	Yersinia enterocolitica	393305	57741
Yersinia enterocolitica subsp. palearctica 105.5R(r)	Yersinia enterocolitica	930944	162069
Yersinia enterocolitica subsp. palearctica Y11	Yersinia enterocolitica	994476	63663
Yersinia pestis A1122	Yersinia pestis	1035377	158119
Yersinia pestis Angola	Yersinia pestis	187410	57875
Yersinia pestis Antiqua	Yersinia pestis	214092	57621
Yersinia pestis biovar Medievalis str. Harbin 35	Yersinia pestis	229193	58037
Yersinia pestis biovar Microtus str. 91001	Yersinia pestis	349746	58485
Yersinia pestis CO92	Yersinia pestis	360102	58607
Yersinia pestis D106004	Yersinia pestis	377628	58609
Yersinia pestis D182038	Yersinia pestis	386656	58619
Yersinia pestis KIM10+	Yersinia pestis	547048	158537
Yersinia pestis Nepal516	Yersinia pestis	637382	158071
Yersinia pestis Pestoides F	Yersinia pestis	637385	158073
Yersinia pestis Z176003	Yersinia pestis	637386	47317

Yersinia pseudotuberculosis IP 31758	Yersinia pseudotuberculosis	273123	58157
Yersinia pseudotuberculosis IP 32953	Yersinia pseudotuberculosis	349747	58487
Yersinia pseudotuberculosis PB1/+	Yersinia pseudotuberculosis	502800	59151
Yersinia pseudotuberculosis YPIII	Yersinia pseudotuberculosis	502801	59153

(a) Strain designation
(b) Species name
(c) NCBI bioproject id of the sequencing project
(d) NCBI taxonomy strain id
