# Supplementary material for: From Genomes to Phenotypes: Traitar, the Microbial Trait Analyzer
Source: mSystems. 2016 Dec 27;1(6):e00101-16. doi: 10.1128/mSystems.00101-16 (PMC5192078; doi:10.1128/mSystems.00101-16)
Supplement: Table S4 [file sys006162072st4.pdf]

Supplementary Table S4 Mapping of bacterial strains to 42 species in the Global Infectious Disease and Epidemiology Online Network with links to the National Center for Biotechnology Information (NCBI) databases

Strain(a)	Species(b)	NCBI Bioproject id(c)	NCBI taxonomy id(d)
Actinobacillus suis H91-0380	Actinobacillus suis	176363	696748
Adlercreutzia equolifaciens DSM 19450	Adlercreutzia equolifaciens	223286	1384484
Alistipes shahii WAL 8301	Alistipes shahii	197175	717959
Bacillus infantis NRRL B-14911	Bacillus infantis	222804	1367477
Bacteroides xylanisolvens XB1A	Bacteroides xylanisolvens	197168	657309
Burkholderia cepacia GG4	Burkholderia cepacia	173858	1009846
Butyrivibrio fibrisolvens	Butyrivibrio fibrisolvens	197155	831
Campylobacter coli 15-537360	Campylobacter coli	226113	1358410
Campylobacter coli CVM N29710	Campylobacter coli	219322	1273173
Corynebacterium argentoratense DSM 44202	Corynebacterium argentoratense	217419	1348662
Enterococcus casseliflavus EC20	Enterococcus casseliflavus	55693	565655
Enterococcus mundtii QU 25	Enterococcus mundtii	229420	1300150
Erysipelothrix rhusiopathiae	Erysipelothrix rhusiopathiae	68021	1648
Erysipelothrix rhusiopathiae SY1027	Erysipelothrix rhusiopathiae	206518	1313290
Eubacterium siraeum	Eubacterium siraeum	197160	39492
Eubacterium siraeum V10Sc8a	Eubacterium siraeum	197178	717961
Faecalibacterium prausnitzii	Faecalibacterium prausnitzii	197157	853
Faecalibacterium prausnitzii L2-6	Faecalibacterium prausnitzii	197183	718252
Fretibacterium fastidiosum	Fretibacterium fastidiosum	197182	651822
Gordonibacter pamelaeae 7-10-1-b	Gordonibacter pamelaeae	197167	657308
Lactobacillus paracasei subsp. paracasei 8700:2	Lactobacillus paracasei	55295	537973
Listeria ivanovii	Listeria ivanovii	73473	1638
Mannheimia haemolytica D153	Mannheimia haemolytica	212303	1261126
Mannheimia haemolytica D171	Mannheimia haemolytica	212304	1311759
Mannheimia haemolytica D174	Mannheimia haemolytica	212305	1311760
Mannheimia haemolytica M42548	Mannheimia haemolytica	198769	1316932
Mannheimia haemolytica USDA-ARS-USMARC-183	Mannheimia haemolytica	195458	1249531
Mannheimia haemolytica USDA-ARS-USMARC-185	Mannheimia haemolytica	195457	1249526
Mannheimia haemolytica USMARC_2286	Mannheimia haemolytica	213228	1366053
Megasphaera elsdenii	Megasphaera elsdenii	71135	907

Mycoplasma hominis	Mycoplasma hominis	41875	2098
Nocardia brasiliensis ATCC 700358	Nocardia brasiliensis	86913	1133849
Nocardia cyriacigeorgica	Nocardia cyriacigeorgica	89395	135487
Pandoraea pnomenusa 3kgm	Pandoraea pnomenusa	229878	1416914
Photorhabdus asymbiotica subsp. asymbiotica ATCC 43949	Photorhabdus asymbiotica	59243	553480
Prevotella dentalis DSM 3688	Prevotella dentalis	184818	908937
Propionibacterium avidum 44067	Propionibacterium avidum	197361	1170318
Pseudomonas monteilii SB3078	Pseudomonas monteilii	232252	1435044
Pseudomonas monteilii SB3101	Pseudomonas monteilii	232253	1435058
Raoultella ornithinolytica B6	Raoultella ornithinolytica	198431	1286170
Roseburia intestinalis	Roseburia intestinalis	197164	166486
Roseburia intestinalis XB6B4	Roseburia intestinalis	197179	718255
Ruminococcus bromii	Ruminococcus bromii	197158	40518
Ruminococcus champanellensis 18P13	Ruminococcus champanellensis	197169	213810
Serratia marcescens FGI94	Serratia marcescens	185180	1249634
Serratia marcescens WW4	Serratia marcescens	188478	435998
Staphylococcus pasteuri SP1	Staphylococcus pasteuri	226267	1276282
Staphylococcus warneri SG1	Staphylococcus warneri	187059	1194526
Streptococcus anginosus C1051	Streptococcus anginosus	218003	862970
Streptococcus anginosus C238	Streptococcus anginosus	218004	862971
Streptococcus constellatus subsp. pharyngis C1050	Streptococcus constellatus	218002	862969
Streptococcus constellatus subsp. pharyngis C232	Streptococcus constellatus	217998	696216
Streptococcus constellatus subsp. pharyngis C818	Streptococcus constellatus	218001	862968
Streptococcus iniae SF1	Streptococcus iniae	206041	1318633
Streptococcus lutetiensis 033	Streptococcus lutetiensis	213397	1076934
Streptococcus oligofermentans AS 1.3089	Streptococcus oligofermentans	201429	1302863
Streptomyces albus J1074	Streptomyces albus	196849	457425
Vibrio alginolyticus NBRC 15630 = ATCC 17749	Vibrio alginolyticus	199933	1219076

(a) Strain designation

(b) Species name

(c) NCBI bioproject id of the sequencing project

(d) NCBI taxonomy strain id
