# Supplementary material for: From Genomes to Phenotypes: Traitar, the Microbial Trait Analyzer
Source: mSystems. 2016 Dec 27;1(6):e00101-16. doi: 10.1128/mSystems.00101-16 (PMC5192078; doi:10.1128/mSystems.00101-16)
Supplement: Table S5 [file sys006162072st5.pdf]

Supplementary Table S5 Mapping of bacterial strains to 296 species described in Bergey's Manual of Systematic Bacteriology

Strain(a)	Species(b)	NCBI taxonomy id(d)
[Bacteroides] pectinophilus ATCC 43243	[Bacteroides] pectinophilus	483218.5
[Clostridium] mangenotii LM2	[Clostridium] mangenotii	1392497.3
[Clostridium] mangenotii TR	[Clostridium] mangenotii	1408823.3
[Eubacterium] cylindroides ATCC 27803	Faecalitalea cylindroides	649755.3
[Eubacterium] cylindroides T2-87	Faecalitalea cylindroides	717960.3
Abiotrophia defectiva ATCC 49176	Abiotrophia defectiva	592010.4
Acetobacterium woodii DSM 1030	Acetobacterium woodii	931626.3
Acidithiobacillus caldus ATCC 51756	Acidithiobacillus caldus	637389.3
Acidithiobacillus caldus SM-1	Acidithiobacillus caldus	990288.8
Acidithiobacillus ferrooxidans ATCC 23270	Acidithiobacillus ferrooxidans	243159.4
Acidithiobacillus ferrooxidans ATCC 53993	Acidithiobacillus ferrooxidans	380394.4
Acidithiobacillus thiooxidans	Acidithiobacillus thiooxidans	930.4
Acidithiobacillus thiooxidans A01	Acidithiobacillus thiooxidans	1432062.4
Acidithiobacillus thiooxidans ATCC 19377	Acidithiobacillus thiooxidans	637390.5
Aerococcus viridans ATCC 11563	Aerococcus viridans	655812.3
Aerococcus viridans LL1	Aerococcus viridans	1175629.3
Alkalibacillus haloalkaliphilus C5	Alkalibacillus haloalkaliphilus	1193119.3
Alkaliphilus transvaalensis ATCC 700919	Alkaliphilus transvaalensis	1408422.3
Amphibacillus xylanus NBRC 15112	Amphibacillus xylanus	698758.3
Anaerococcus hydrogenalis ACS-025-V-Sch4	Anaerococcus hydrogenalis	879306.3
Anaerococcus hydrogenalis DSM 7454	Anaerococcus hydrogenalis	561177.4
Anaerococcus lactolyticus ATCC 51172	Anaerococcus lactolyticus	525254.4
Anaerococcus lactolyticus S7-1-13	Anaerococcus lactolyticus	1284686.3
Anaerococcus tetradius ATCC 35098	Anaerococcus tetradius	525255.3
Anaerococcus vaginalis ATCC 51170	Anaerococcus vaginalis	655811.4
Anaerostipes hadrus DSM 3319	Anaerostipes hadrus	649757.3
Anaerotruncus colihominis DSM 17241	Anaerotruncus colihominis	445972.6
Aneurinibacillus aneurinilyticus ATCC 12856	Aneurinibacillus aneurinilyticus	649747.3
Aneurinibacillus terranovensis DSM 18919	Aneurinibacillus terranovensis	1121002.4
Anoxybacillus ayderensis AB04	Anoxybacillus ayderensis	265546.4
Anoxybacillus flavithermus	Anoxybacillus flavithermus	33934.3
Anoxybacillus flavithermus AK1	Anoxybacillus flavithermus	1297581.3
Anoxybacillus flavithermus NBRC 109594	Anoxybacillus flavithermus	1315967.3
Anoxybacillus flavithermus TNO-09.006	Anoxybacillus flavithermus	1267580.3
Anoxybacillus flavithermus WK1	Anoxybacillus flavithermus	491915.6
Anoxybacillus gonensis G2	Anoxybacillus gonensis	198467.4
Anoxybacillus kamchatkensis G10	Anoxybacillus kamchatkensis	1212546.3
Anoxybacillus tepidamans PS2	Anoxybacillus tepidamans	1382358.3
Azotobacter chroococcum NCIMB 8003	Azotobacter chroococcum	1328314.4
Azotobacter vinelandii CA	Azotobacter vinelandii	1283330.3
Azotobacter vinelandii CA6	Azotobacter vinelandii	1283331.3
Azotobacter vinelandii DJ	Azotobacter vinelandii	322710.5
Bacillus alcalophilus ATCC 27647	Bacillus alcalophilus	1218173.3
Bacillus halodurans C-125	Bacillus halodurans	272558.8
Bacillus methanolicus MGA3	Bacillus methanolicus	796606.3
Bacillus mycoides ATCC 6462	Bacillus mycoides	1405.1
Bacillus mycoides DSM 2048	Bacillus mycoides	526997.3

Bacillus mycoides Rock1-4	Bacillus mycoides	526998.3
Bacillus mycoides Rock3-17	Bacillus mycoides	526999.3
Bacillus pseudofirmus OF4	Bacillus pseudofirmus	398511.4
Bacillus pseudomycoides DSM 12442	Bacillus pseudomycoides	527000.3
Bacillus selenitireducens MLS10	[Bacillus] selenitireducens	439292.5
Bacillus thermoamylovorans	Bacillus thermoamylovorans	35841.3
Bacillus weihenstephanensis	Bacillus weihenstephanensis	86662.6
Bacillus weihenstephanensis FSL H7-687	Bacillus weihenstephanensis	1227358.4
Bacillus weihenstephanensis FSL R5-860	Bacillus weihenstephanensis	1227359.4
Bacillus weihenstephanensis KBAB4	Bacillus weihenstephanensis	315730.11
Bacillus weihenstephanensis NBRC 101238 = DSM 11821	Bacillus weihenstephanensis	1220585.4
Blautia hansenii DSM 20583	Blautia hansenii	537007.6
Blautia producta ATCC 27340	Blautia producta	1121114.4
Blautia producta ER3	Blautia producta	33035.4
Blautia schinkii DSM 10518	Blautia schinkii	1410649.3
Brevibacillus agri 5-2	Brevibacillus agri	1444307.3
Brevibacillus agri BAB-2500	Brevibacillus agri	1246477.3
Brevibacillus borstelensis 3096-7	Brevibacillus borstelensis	1444309.3
Brevibacillus borstelensis AK1	Brevibacillus borstelensis	1300222.3
Brevibacillus borstelensis cifa_chp40	Brevibacillus borstelensis	1429889.3
Brevibacillus borstelensis LChuR05	Brevibacillus borstelensis	45462.4
Brevibacillus laterosporus	Brevibacillus laterosporus	1465.13
Brevibacillus laterosporus DSM 25	Brevibacillus laterosporus	1121121.3
Brevibacillus laterosporus GI-9	Brevibacillus laterosporus	1118154.3
Brevibacillus laterosporus LMG 15441	Brevibacillus laterosporus	1042163.3
Brevibacillus laterosporus PE36	Brevibacillus laterosporus	1399144.3
Brevibacillus thermoruber 423	Brevibacillus thermoruber	1346613.3
Brevibacillus thermoruber PM1	Brevibacillus thermoruber	1382302.3
Bulleidia extructa W1219	Bulleidia extructa	679192.3
Buttiauxella agrestis	Buttiauxella agrestis	82977.3
Buttiauxella agrestis ATCC 33320	Buttiauxella agrestis	1006004.4
Butyrivibrio fibrisolvens 16/4	Butyrivibrio fibrisolvens	657324.3
Butyrivibrio fibrisolvens AB2020	Butyrivibrio fibrisolvens	1280697.3
Butyrivibrio fibrisolvens FE2007	Butyrivibrio fibrisolvens	1280700.3
Butyrivibrio fibrisolvens MD2001	Butyrivibrio fibrisolvens	1280703.3
Butyrivibrio fibrisolvens ND3005	Butyrivibrio fibrisolvens	1280696.3
Butyrivibrio fibrisolvens WTE3004	Butyrivibrio fibrisolvens	1280699.3
Butyrivibrio fibrisolvens YRB2005	Butyrivibrio fibrisolvens	1280687.3
Caldibacillus debilis DSM 16016	Caldibacillus debilis	1121917.3
Cardiobacterium hominis ATCC 15826	Cardiobacterium hominis	638300.3
Carnobacterium maltaromaticum ATCC 35586	Carnobacterium maltaromaticum	1087479.3
Carnobacterium maltaromaticum LMA28	Carnobacterium maltaromaticum	1234679.3
Chromohalobacter israelensis 6768	Chromohalobacter israelensis	141390.3
Chromohalobacter salexigens DSM 3043	Chromohalobacter salexigens	290398.11
Citrobacter amalonaticus	Citrobacter amalonaticus	35703.8
Citrobacter braakii GTA-CB04	Citrobacter braakii	57706.1
Citrobacter farmeri GTC 1319	Citrobacter farmeri	1114922.3
Citrobacter freundii 4_7_47CFAA	Citrobacter freundii	742730.3
Citrobacter freundii ATCC 8090 = MTCC 1658	Citrobacter freundii	1006003.3
Citrobacter freundii CFNIH1	Citrobacter freundii	1333848.3

Citrobacter freundii GTC 09479	Citrobacter freundii	1288347.3
Citrobacter freundii GTC 09629	Citrobacter freundii	1297584.3
Citrobacter freundii MGH 56	Citrobacter freundii	1439318.3
Citrobacter freundii NBRC 12681	Citrobacter freundii	1114920.3
Citrobacter freundii RLS1	Citrobacter freundii	1454056.3
Citrobacter freundii str. ballerup 7851/39	Citrobacter freundii	670484.3
Citrobacter freundii UCI 31	Citrobacter freundii	1400136.3
Citrobacter freundii UCI 32	Citrobacter freundii	1400137.3
Citrobacter sedlakii NBRC 105722	Citrobacter sedlakii	1218086.3
Citrobacter werkmanii NBRC 105721	Citrobacter werkmanii	1114921.3
Citrobacter youngae ATCC 29220	Citrobacter youngae	500640.5
Clostridium acidurici 9a	Gottschalkia acidurici	1128398.3
Clostridium cellulosi CS-4-4	[Clostridium] cellulosi	1367212.3
Clostridium leptum DSM 753	[Clostridium] leptum	428125.8
Clostridium methylpentosum DSM 5476	[Clostridium] methylpentosum	537013.3
Clostridium orbiscindens 1_3_50AFAA	Flavonifractor plautii	742738.3
Clostridium sporosphaeroides DSM 1294	[Clostridium] sporosphaeroides	1121334.3
Clostridium sticklandii DSM 519	[Clostridium] sticklandii	499177.3
Clostridium thermocellum AD2	Ruminiclostridium thermocellum	1138384.3
Clostridium thermocellum ATCC 27405	Ruminiclostridium thermocellum	203119.11
Clostridium thermocellum BC1	Ruminiclostridium thermocellum	1349417.3
Clostridium thermocellum DSM 1313	Ruminiclostridium thermocellum	637887.3
Clostridium thermocellum DSM 2360	Ruminiclostridium thermocellum	572545.3
Clostridium thermocellum JW20	Ruminiclostridium thermocellum	492476.4
Clostridium thermocellum YS	Ruminiclostridium thermocellum	1094188.3
Colwellia psychrerythraea 34H	Colwellia psychrerythraea	167879.5
Colwellia psychrerythraea GAB14E	Colwellia psychrerythraea	28229.3
Desulfitobacterium dehalogenans ATCC 51507	Desulfitobacterium dehalogenans	756499.4
Desulfitobacterium hafniense DCB-2	Desulfitobacterium hafniense	272564.6
Desulfitobacterium hafniense DP7	Desulfitobacterium hafniense	537010.4
Desulfitobacterium hafniense PCP-1	Desulfitobacterium hafniense	1090321.3
Desulfitobacterium hafniense TCP-A	Desulfitobacterium hafniense	872024.4
Desulfitobacterium hafniense Y51	Desulfitobacterium hafniense	138119.41
Desulfitobacterium metallireducens DSM 15288	Desulfitobacterium metallireducens	871968.4
Dickeya chrysanthemi M074	Dickeya chrysanthemi	556.28
Dickeya chrysanthemi NCPPB 3533	Dickeya chrysanthemi	1224148.3
Dickeya chrysanthemi NCPPB 402	Dickeya chrysanthemi	1223569.3
Dickeya chrysanthemi NCPPB 516	Dickeya chrysanthemi	1223571.3
Dolosigranulum pigrum ATCC 51524	Dolosigranulum pigrum	883103.3
Dorea formicigenerans 4_6_53AFAA	Dorea formicigenerans	742765.5
Dorea formicigenerans ATCC 27755	Dorea formicigenerans	411461.4
Dorea longicatena AGR2136	Dorea longicatena	1280698.3
Dorea longicatena DSM 13814	Dorea longicatena	411462.6
Enterobacter cancerogenus ATCC 35316	Enterobacter cancerogenus	500639.8
Enterobacter cancerogenus M004	Enterobacter cancerogenus	69218.3
Enterobacter cloacae ATCC 13047	Enterobacter cloacae	550.124
Enterobacter cloacae BIDMC 66	Enterobacter cloacae	1439324.3
Enterobacter cloacae BIDMC 67	Enterobacter cloacae	1439325.3
Enterobacter cloacae BIDMC 8	Enterobacter cloacae	1329846.6
Enterobacter cloacae BWH 31	Enterobacter cloacae	1329845.3

Enterobacter cloacae BWH 43	Enterobacter cloacae	1439328.3
Enterobacter cloacae CHS 79	Enterobacter cloacae	1439326.3
Enterobacter cloacae EC_38VIM1	Enterobacter cloacae	1334630.3
Enterobacter cloacae ECNIH2	Enterobacter cloacae	1333850.3
Enterobacter cloacae ECNIH3	Enterobacter cloacae	1333851.3
Enterobacter cloacae ECR091	Enterobacter cloacae	1333849.3
Enterobacter cloacae EcWSU1	Enterobacter cloacae	1045856.3
Enterobacter cloacae IIT-BT 08	Enterobacter cloacae	1070842.3
Enterobacter cloacae ISC8	Enterobacter cloacae	1432556.3
Enterobacter cloacae JD6301	Enterobacter cloacae	1399774.3
Enterobacter cloacae JD8715	Enterobacter cloacae	1399775.3
Enterobacter cloacae MGH 53	Enterobacter cloacae	1439329.3
Enterobacter cloacae MGH 54	Enterobacter cloacae	1439330.3
Enterobacter cloacae MR2	Enterobacter cloacae	1312879.3
Enterobacter cloacae MRSN 11489	Enterobacter cloacae	1410032.3
Enterobacter cloacae P101	Enterobacter cloacae	1354030.3
Enterobacter cloacae S611	Enterobacter cloacae	1399146.3
Enterobacter cloacae str. Hanford	Enterobacter cloacae	1340854.3
Enterobacter cloacae subsp. cloacae 08XA1	Enterobacter cloacae	1203195.3
Enterobacter cloacae subsp. cloacae ATCC 13047	Enterobacter cloacae	716541.4
Enterobacter cloacae subsp. cloacae ENHKU01	Enterobacter cloacae	1211025.3
Enterobacter cloacae subsp. cloacae GS1	Enterobacter cloacae	1177927.3
Enterobacter cloacae subsp. cloacae NCTC 9394	Enterobacter cloacae	718254.4
Enterobacter cloacae subsp. cloacae SY-70	Enterobacter cloacae	1449089.4
Enterobacter cloacae subsp. dissolvens SDM	Enterobacter cloacae	1104326.3
Enterobacter cloacae UCI 23	Enterobacter cloacae	1400146.3
Enterobacter cloacae UCI 24	Enterobacter cloacae	1400147.3
Enterobacter cloacae UCI 29	Enterobacter cloacae	1400148.3
Enterobacter cloacae UCI 30	Enterobacter cloacae	1400149.3
Enterobacter cloacae UCI 35	Enterobacter cloacae	1400150.3
Enterobacter cloacae UCI 36	Enterobacter cloacae	1400151.3
Enterobacter cloacae UCI 39	Enterobacter cloacae	1400152.3
Enterobacter cloacae UCI 49	Enterobacter cloacae	1400154.3
Enterobacter cloacae UCI 50	Enterobacter cloacae	1400155.3
Enterobacter cloacae UCICRE 11	Enterobacter cloacae	1329855.3
Enterobacter cloacae UCICRE 12	Enterobacter cloacae	1329856.3
Enterobacter cloacae UCICRE 3	Enterobacter cloacae	1329852.3
Enterobacter cloacae UCICRE 5	Enterobacter cloacae	1329853.3
Enterobacter cloacae UCICRE 9	Enterobacter cloacae	1329854.3
Erwinia amylovora 01SFR-BO	Erwinia amylovora	1255306.3
Erwinia amylovora ACW56400	Erwinia amylovora	1027397.3
Erwinia amylovora ATCC 49946	Erwinia amylovora	716540.3
Erwinia amylovora CFBP 1232	Erwinia amylovora	1255307.3
Erwinia amylovora CFBP 2585	Erwinia amylovora	1255305.3
Erwinia amylovora CFBP1430	Erwinia amylovora	665029.3
Erwinia amylovora Ea266	Erwinia amylovora	1255304.3
Erwinia amylovora Ea356	Erwinia amylovora	1255303.3
Erwinia amylovora Ea644	Erwinia amylovora	1255309.3
Erwinia amylovora MR1	Erwinia amylovora	1255310.3
Erwinia amylovora NBRC 12687	Erwinia amylovora	1219359.3

Erwinia amylovora UPN527	Erwinia amylovora	1255308.3
Erwinia billingiae Eb661	Erwinia billingiae	634500.5
Erwinia mallotivora	Erwinia mallotivora	69222.5
Erwinia tracheiphila PSU-1	Erwinia tracheiphila	1044999.3
Erysipelothrix rhusiopathiae ATCC 19414	Erysipelothrix rhusiopathiae	525280.3
Erysipelothrix rhusiopathiae SY1027	Erysipelothrix rhusiopathiae	1313290.3
Escherichia hermannii NBRC 105704	Escherichia hermannii	1115512.3
Escherichia vulneris NBRC 102420	Escherichia vulneris	1115515.3
Eubacterium acidaminophilum DSM 3953	Eubacterium acidaminophilum	1286171.3
Eubacterium biforme DSM 3989	Holdemanella biformis	518637.5
Eubacterium brachy ATCC 33089	Eubacterium brachy	1321814.3
Eubacterium desmolans ATCC 43058	Eubacterium desmolans	1408437.3
Eubacterium dolichum DSM 3991	[Eubacterium] dolichum	428127.7
Eubacterium hallii DSM 3353	[Eubacterium] hallii	411469.3
Eubacterium infirmum F0142	[Eubacterium] infirmum	883109.3
Eubacterium nodatum ATCC 33099	[Eubacterium] nodatum	1161902.3
Eubacterium plexicaudatum ASF492	Eubacterium plexicaudatum	1235802.3
Eubacterium ramulus ATCC 29099	Eubacterium ramulus	1256908.3
Eubacterium saphenum ATCC 49989	Eubacterium saphenum	592031.3
Eubacterium siraeum 70/3	[Eubacterium] siraeum	657319.3
Eubacterium siraeum DSM 15702	[Eubacterium] siraeum	428128.7
Eubacterium siraeum V10Sc8a	[Eubacterium] siraeum	717961.3
Eubacterium sulci ATCC 35585	[Eubacterium] sulci	888727.3
Eubacterium ventriosum ATCC 27560	Eubacterium ventriosum	411463.4
Eubacterium xylanophilum ATCC 35991	Eubacterium xylanophilum	1336241.3
Exiguobacterium acetylicum DSM 20416	Exiguobacterium acetylicum	1397697.3
Exiguobacterium antarcticum B7	Exiguobacterium antarcticum	1087448.3
Exiguobacterium antarcticum DSM 14480	Exiguobacterium antarcticum	1397700.3
Exiguobacterium aurantiacum DSM 6208	Exiguobacterium aurantiacum	1397694.4
Facklamia hominis ACS-120-V-Sch10	Facklamia hominis	883110.3
Facklamia hominis CCUG 36813	Facklamia hominis	883111.3
Facklamia ignava CCUG 37419	Facklamia ignava	883112.3
Facklamia languida CCUG 37842	Facklamia languida	883113.3
Facklamia sourekii ATCC 700629	Facklamia sourekii	1408438.3
Faecalibacterium cf. prausnitzii KLE1255	Faecalibacterium prausnitzii	748224.3
Faecalibacterium prausnitzii A2-165	Faecalibacterium prausnitzii	411483.3
Faecalibacterium prausnitzii L2-6	Faecalibacterium prausnitzii	718252.3
Faecalibacterium prausnitzii M21/2	Faecalibacterium prausnitzii	411485.1
Faecalibacterium prausnitzii SL3/3	Faecalibacterium prausnitzii	657322.3
Flavonifractor plautii ATCC 29863	Flavonifractor plautii	411475.3
Gallibacterium anatis 10672-6	Gallibacterium anatis	1396515.3
Gallibacterium anatis 12656/12	Gallibacterium anatis	1195244.3
Gallibacterium anatis 23T10	Gallibacterium anatis	750.1
Gallibacterium anatis 4895	Gallibacterium anatis	1396510.3
Gallibacterium anatis 7990	Gallibacterium anatis	1396511.3
Gallibacterium anatis CCM5995	Gallibacterium anatis	1396513.3
Gallibacterium anatis DSM 16844 = F 149	Gallibacterium anatis	1121910.3
Gallibacterium anatis IPDH697-78	Gallibacterium anatis	1396514.3
Gallibacterium anatis str. Avicor	Gallibacterium anatis	1396512.3
Gallibacterium anatis UMN179	Gallibacterium anatis	1005058.3

Gemella bergeriae ATCC 700627	Gemella bergeri	1321820.3
Gemella cuniculi DSM 15828	Gemella cuniculi	1121914.3
Gemella haemolysans ATCC 10379	Gemella haemolysans	546270.5
Gemella haemolysans M341	Gemella haemolysans	562981.3
Gemella sanguinis ATCC 700632	Gemella sanguinis	1408440.3
Gemella sanguinis M325	Gemella sanguinis	562983.3
Geobacillus caldoxylosilyticus CIC9	Geobacillus caldoxylosilyticus	1234664.3
Geobacillus caldoxylosilyticus NBRC 107762	Geobacillus caldoxylosilyticus	1220594.3
Geobacillus kaustophilus GBlys	Geobacillus kaustophilus	1337888.4
Geobacillus kaustophilus HTA426	Geobacillus kaustophilus	235909.7
Geobacillus kaustophilus NBRC 102445	Geobacillus kaustophilus	1220595.3
Geobacillus stearothermophilus ATCC 7953	Geobacillus stearothermophilus	937593.4
Geobacillus subterraneus PSS2	Geobacillus subterraneus	1382357.3
Geobacillus thermocatenulatus GS-1	Geobacillus thermocatenulatus	1444308.3
Geobacillus thermodenitrificans DSM 465	Geobacillus thermodenitrificans	1413215.3
Geobacillus thermodenitrificans NG80-2	Geobacillus thermodenitrificans	420246.7
Geobacillus thermoglucosidans TNO-09.020	Geobacillus thermoglucosidasius	1136178.3
Geobacillus thermoglucosidasius C56-YS93	Geobacillus thermoglucosidasius	634956.3
Geobacillus thermoglucosidasius NBRC 107763	Geobacillus thermoglucosidasius	1223501.3
Geobacillus thermoleovorans B23	Geobacillus thermoleovorans	1406857.3
Geobacillus thermoleovorans CCB_US3_UF5	Geobacillus thermoleovorans	1111068.3
Geobacillus vulcani PSS1	Geobacillus vulcani	1382315.3
Gracilibacillus boraciitolerans JCM 21714	Gracilibacillus boraciitolerans	1298598.3
Granulicatella adiacens ATCC 49175	Granulicatella adiacens	638301.3
Granulicatella elegans ATCC 700633	Granulicatella elegans	626369.3
Halobacillus halophilus DSM 2266	Halobacillus halophilus	866895.3
Halorhodospira halochloris str. A	Halorhodospira halochloris	1354791.3
Halorhodospira halophila SL1	Halorhodospira halophila	349124.8
Halothermothrix orenii H 168	Halothermothrix orenii	373903.5
Halothiobacillus neapolitanus c2	Halothiobacillus neapolitanus	555778.5
Holdemania filiformis DSM 12042	Holdemania filiformis	545696.5
Hydrogenovibrio marinus	Hydrogenovibrio marinus	28885.3
Hydrogenovibrio marinus DSM 11271	Hydrogenovibrio marinus	1123513.3
Jeotgalibacillus campisalis SF-57	Jeotgalibacillus campisalis	220754.4
Jeotgalicoccus psychrophilus DSM 19085	Jeotgalicoccus psychrophilus	1122129.3
Kyrpidia tusciae DSM 2912	Kyrpidia tusciae	562970.4
Lachnoanaerobaculum saburreum DSM 3986	Lachnoanaerobaculum saburreum	887325.3
Lachnoanaerobaculum saburreum F0468	Lachnoanaerobaculum saburreum	1095750.3
Lelliottia amnigena CHS 78	Lelliottia amnigena	1439331.3
Leuconostoc argentinum KCTC 3773	Leuconostoc lactis	886872.3
Leuconostoc carnosum JB16	Leuconostoc carnosum	1229758.3
Leuconostoc carnosum KCTC 3525	Leuconostoc carnosum	1046593.3
Leuconostoc fallax KCTC 3537	Leuconostoc fallax	907931.3
Leuconostoc lactis	Leuconostoc lactis	1246.4
Leuconostoc lactis KCTC 3528 = DSM 20202	Leuconostoc lactis	935294.3
Leuconostoc pseudomesenteroides 1159	Leuconostoc pseudomesenteroides	1339246.3
Leuconostoc pseudomesenteroides 4882	Leuconostoc pseudomesenteroides	1154757.4
Leuconostoc pseudomesenteroides KCTC 3652	Leuconostoc pseudomesenteroides	935295.4
Leuconostoc pseudomesenteroides PS12	Leuconostoc pseudomesenteroides	1339247.3
Listeria grayi DSM 20601	Listeria grayi	525367.9

Listeria grayi FSL F6-1183	Listeria grayi	1265827.4
Listeria ivanovii FSL F6-596	Listeria ivanovii	702454.3
Listeria ivanovii subsp. ivanovii PAM 55	Listeria ivanovii	1638.4
Listeria ivanovii subsp. ivanovii WSLC 3010	Listeria ivanovii	202751.3
Listeria ivanovii subsp. londoniensis WSLC 30167	Listeria ivanovii	202752.6
Listeria ivanovii WSLC3009	Listeria ivanovii	1457190.3
Lonsdalea quercina subsp. quercina	Lonsdalea quercina	1082705.1
Luteimonas mephitis DSM 12574	Luteimonas mephitis	1122183.3
Lysinibacillus fusiformis	Lysinibacillus fusiformis	28031.4
Lysinibacillus fusiformis H1k	Lysinibacillus fusiformis	1416755.3
Lysinibacillus fusiformis ZB2	Lysinibacillus fusiformis	1231627.3
Lysinibacillus fusiformis ZC1	Lysinibacillus fusiformis	714961.3
Lysinibacillus odysseyi 34hs-1 = NBRC 100172	Lysinibacillus odysseyi	1220589.3
Lysobacter antibioticus	Lysobacter antibioticus	84531.4
Lysobacter antibioticus HS124	Lysobacter antibioticus	1286308.3
Macrococcus caseolyticus JCSC5402	Macrococcus caseolyticus	458233.11
Mannheimia granulomatis DSM 19156	Mannheimia granulomatis	1122190.3
Mannheimia haemolytica D153	Mannheimia haemolytica	1261126.6
Mannheimia haemolytica D171	Mannheimia haemolytica	1311759.4
Mannheimia haemolytica D174	Mannheimia haemolytica	1311760.4
Mannheimia haemolytica D193	Mannheimia haemolytica	1329904.3
Mannheimia haemolytica D35	Mannheimia haemolytica	1329905.3
Mannheimia haemolytica D38	Mannheimia haemolytica	1329906.3
Mannheimia haemolytica M42548	Mannheimia haemolytica	1316932.3
Mannheimia haemolytica MhBrain2012	Mannheimia haemolytica	1329902.3
Mannheimia haemolytica MhSwine2000	Mannheimia haemolytica	1329903.3
Mannheimia haemolytica PHL213	Mannheimia haemolytica	272629.3
Mannheimia haemolytica serotype 6 str. H23	Mannheimia haemolytica	1261125.3
Mannheimia haemolytica serotype A1/A6 str. PKL10	Mannheimia haemolytica	1450449.3
Mannheimia haemolytica serotype A2 str. BOVINE	Mannheimia haemolytica	669262.3
Mannheimia haemolytica serotype A2 str. OVINE	Mannheimia haemolytica	669261.3
Mannheimia haemolytica USDA-ARS-USMARC-183	Mannheimia haemolytica	1249531.3
Mannheimia haemolytica USDA-ARS-USMARC-185	Mannheimia haemolytica	1249526.3
Mannheimia haemolytica USMARC_2286	Mannheimia haemolytica	1366053.4
Mannheimia varigena USDA-ARS-USMARC-1261	Mannheimia varigena	1432056.3
Mannheimia varigena USDA-ARS-USMARC-1296	Mannheimia varigena	1433287.3
Mannheimia varigena USDA-ARS-USMARC-1312	Mannheimia varigena	1434214.3
Mannheimia varigena USDA-ARS-USMARC-1388	Mannheimia varigena	1434215.3
Marinococcus halotolerans DSM 16375	Marinococcus halotolerans	1122203.4
Marinomonas mediterranea MMB-1	Marinomonas mediterranea	717774.3
Megasphaera elsdenii 24-50	Megasphaera elsdenii	907.5
Megasphaera elsdenii DSM 20460	Megasphaera elsdenii	907.4
Megasphaera elsdenii T81	Megasphaera elsdenii	1410663.3
Methylobacter luteus IMV-B-3098	Methylobacter luteus	1095552.3
Methylobacter marinus A45	Methylobacter marinus	674036.3
Methylobacter whittenburyi	Methylobacter whittenburyi	39770.3
Methylococcus capsulatus str. Bath	Methylococcus capsulatus	243233.7
Methylococcus capsulatus str. Texas = ATCC 19069	Methylococcus capsulatus	1224744.3
Methylomicrobium agile	Methylomicrobium agile	39774.3
Methylomicrobium album BG8	Methylomicrobium album	686340.3

Methylomonas methanica MC09	Methylomonas methanica	857087.3
Mitsuokella jalaludinii DSM 13811	Mitsuokella jalaludinii	1410665.3
Mitsuokella multacida DSM 20544	Mitsuokella multacida	500635.8
Moorella thermoacetica ATCC 39073	Moorella thermoacetica	264732.11
Moorella thermoacetica Y72	Moorella thermoacetica	1325331.3
Oenococcus kitaharae DSM 17330	Oenococcus kitaharae	1045004.4
Oenococcus oeni ATCC BAA-1163	Oenococcus oeni	379360.3
Oenococcus oeni AWRIB202	Oenococcus oeni	1160703.3
Oenococcus oeni AWRIB304	Oenococcus oeni	1160702.3
Oenococcus oeni AWRIB318	Oenococcus oeni	1167631.3
Oenococcus oeni AWRIB418	Oenococcus oeni	1206769.3
Oenococcus oeni AWRIB419	Oenococcus oeni	1206770.3
Oenococcus oeni AWRIB422	Oenococcus oeni	1206771.3
Oenococcus oeni AWRIB429	Oenococcus oeni	655225.3
Oenococcus oeni AWRIB548	Oenococcus oeni	1206772.3
Oenococcus oeni AWRIB553	Oenococcus oeni	1206773.3
Oenococcus oeni AWRIB568	Oenococcus oeni	1206774.3
Oenococcus oeni AWRIB576	Oenococcus oeni	1206775.3
Oenococcus oeni DSM 20252 = AWRIB129	Oenococcus oeni	1122618.3
Oenococcus oeni PSU-1	Oenococcus oeni	203123.7
Oenococcus oeni X2L	Oenococcus oeni	1335618.3
Orenia marismortui DSM 5156	Orenia marismortui	926561.3
Paenibacillus panacisoli DSM 21345	Paenibacillus panacisoli	1122922.3
Paenibacillus pasadenensis DSM 19293	Paenibacillus pasadenensis	1122923.3
Paenibacillus peoriae KCTC 3763	Paenibacillus peoriae	1087481.3
Paenibacillus popilliae ATCC 14706	Paenibacillus popilliae	1212764.3
Paenibacillus sanguinis 2301083 = DSM 16941	Paenibacillus sanguinis	1122925.3
Paenibacillus stellifer DSM 14472	Paenibacillus stellifer	169760.4
Paenibacillus terrae HPL-003	Paenibacillus terrae	985665.3
Paenibacillus wynnii DSM 18334	Paenibacillus wynnii	268407.5
Parvimonas micra A293	Parvimonas micra	1408286.3
Parvimonas micra ATCC 33270	Parvimonas micra	411465.1
Parvimonas micra KCOM 1535; ChDC B708	Parvimonas micra	33033.4
Pasteurella dagmatis ATCC 43325	Pasteurella dagmatis	667128.3
Pectobacterium carotovorum M022	Pectobacterium carotovorum	554.6
Pectobacterium carotovorum subsp. brasiliense	Pectobacterium carotovorum	180957.1
Pectobacterium carotovorum subsp. brasiliensis PBR1692	Pectobacterium carotovorum	558269.5
Pectobacterium carotovorum subsp. carotovorum	Pectobacterium carotovorum	555.14
Pectobacterium carotovorum subsp. carotovorum PC1	Pectobacterium carotovorum	561230.3
Pectobacterium carotovorum subsp. carotovorum PCC21	Pectobacterium carotovorum	1218933.3
Pectobacterium carotovorum subsp. odoriferum	Pectobacterium carotovorum	78398.4
Pediococcus acidilactici 7_4	Pediococcus acidilactici	563194.3
Pediococcus acidilactici AGR20	Pediococcus acidilactici	1384067.3
Pediococcus acidilactici D3	Pediococcus acidilactici	1306952.3
Pediococcus acidilactici DSM 20284	Pediococcus acidilactici	862514.3
Pediococcus acidilactici MA18/5M	Pediococcus acidilactici	1080365.4
Pediococcus claussenii ATCC BAA-344	Pediococcus claussenii	701521.8
Peptoniphilus harei ACS-146-V-Sch2b	Peptoniphilus harei	908338.3
Peptoniphilus indolicus ATCC 29427	Peptoniphilus indolicus	997350.3
Peptoniphilus lacrimalis 315-B	Peptoniphilus lacrimalis	596330.3

Peptoniphilus lacrimalis DNF00528	Peptoniphilus lacrimalis	1401070.3
Peptoniphilus lacrimalis DSM 7455	Peptoniphilus lacrimalis	1122949.3
Peptostreptococcus anaerobius 653-L	Peptostreptococcus anaerobius	596329.3
Peptostreptococcus anaerobius VPI 4330	Peptostreptococcus anaerobius	1035196.3
Photobacterium angustum S14	Photobacterium angustum	314292.23
Photobacterium leiognathi lrivu.4.1	Photobacterium leiognathi	1248232.3
Photobacterium leiognathi subsp. mandapamensis svers.1.1.	Photobacterium leiognathi	1001530.3
Photobacterium phosphoreum ANT220	Photobacterium phosphoreum	1454202.3
Photobacterium profundum 3TCK	Photobacterium profundum	314280.5
Photobacterium profundum SS9	Photobacterium profundum	298386.8
Planococcus antarcticus DSM 14505	Planococcus antarcticus	1185653.3
Pluralibacter gergoviae FB2	Pluralibacter gergoviae	61647.1
Pontibacillus chungwhensis BH030062	Pontibacillus chungwhensis	1385513.3
Pontibacillus marinus BH030004 = DSM 16465	Pontibacillus marinus	1385511.3
Proteus penneri ATCC 35198	Proteus penneri	471881.3
Providencia alcalifaciens 205/92	Providencia alcalifaciens	1256988.3
Providencia alcalifaciens Ban1	Providencia alcalifaciens	663916.4
Providencia alcalifaciens Dmel2	Providencia alcalifaciens	1141661.3
Providencia alcalifaciens DSM 30120	Providencia alcalifaciens	520999.6
Providencia alcalifaciens F90-2004	Providencia alcalifaciens	1256987.3
Providencia alcalifaciens PAL-1	Providencia alcalifaciens	1256991.3
Providencia alcalifaciens PAL-2	Providencia alcalifaciens	1256992.3
Providencia alcalifaciens PAL-3	Providencia alcalifaciens	1256993.3
Providencia alcalifaciens R90-1475	Providencia alcalifaciens	1256989.3
Providencia alcalifaciens RIMD 1656011	Providencia alcalifaciens	1256990.3
Providencia rettgeri CCBH11880	Providencia rettgeri	587.17
Providencia rettgeri Dmel1	Providencia rettgeri	1141663.3
Providencia rettgeri DSM 1131	Providencia rettgeri	521000.6
Providencia rustigianii DSM 4541	Providencia rustigianii	500637.6
Pseudoalteromonas citrea	Pseudoalteromonas citrea	43655.3
Pseudoalteromonas citrea NCIMB 1889	Pseudoalteromonas citrea	1117314.3
Pseudoalteromonas luteoviolacea 2ta16	Pseudoalteromonas luteoviolacea	1353533.3
Pseudoalteromonas luteoviolacea B = ATCC 29581	Pseudoalteromonas luteoviolacea	1268239.3
Pseudoalteromonas luteoviolacea HI1	Pseudoalteromonas luteoviolacea	43657.9
Pseudoalteromonas piscicida ATCC 15057	Pseudoalteromonas piscicida	1279016.3
Pseudoalteromonas piscicida JCM 20779	Pseudoalteromonas piscicida	1117317.3
Pseudoalteromonas rubra ATCC 29570	Pseudoalteromonas rubra	1117318.14
Pseudobacteroides cellulosolvens ATCC 35603 = DSM 2933	Pseudobacteroides cellulosolvens	398512.4
Pseudobutyrivibrio ruminis AD2017	Pseudobutyrivibrio ruminis	1280694.3
Pseudobutyrivibrio ruminis CF1b	Pseudobutyrivibrio ruminis	1280688.3
Pseudobutyrivibrio ruminis HUN009	Pseudobutyrivibrio ruminis	1458469.3
Pseudoflavonifractor capillosus ATCC 29799	Pseudoflavonifractor capillosus	411467.6
Pseudomonas agarici NCPPB 2289	Pseudomonas agarici	690598.6
Pseudomonas alcaligenes MRY13-0052	Pseudomonas alcaligenes	1405803.3
Pseudomonas alcaligenes NBRC 14159	Pseudomonas alcaligenes	1215092.3
Pseudomonas alcaligenes OT 69	Pseudomonas alcaligenes	1333854.3
Pseudomonas cichorii JBC1	Pseudomonas cichorii	1441629.3
Pseudomonas corrugata CFBP 5454	Pseudomonas corrugata	1316927.4
Pseudomonas luteola XLDN4-9	Pseudomonas luteola	1207076.3
Pseudomonas oryzihabitans NBRC 102199	Pseudomonas oryzihabitans	1215113.3

Pseudomonas pseudoalcaligenes AD6	Pseudomonas pseudoalcaligenes	1453503.3
Pseudomonas pseudoalcaligenes CECT 5344	Pseudomonas pseudoalcaligenes	1182590.4
Pseudomonas pseudoalcaligenes KF707	Pseudomonas pseudoalcaligenes	1149133.6
Pseudomonas tolaasii 6264	Pseudomonas tolaasii	1161101.3
Pseudomonas tolaasii NCPPB 2192	Pseudomonas tolaasii	564423.7
Pseudomonas tolaasii PMS117	Pseudomonas tolaasii	1030145.6
Psychromonas arctica DSM 14288	Psychromonas arctica	1123036.3
Ruminococcus albus 7	Ruminococcus albus	697329.1
Ruminococcus albus 8	Ruminococcus albus	246199.4
Ruminococcus albus AD2013	Ruminococcus albus	1384065.3
Ruminococcus albus SY3	Ruminococcus albus	1341156.4
Ruminococcus bromii L2-63	Ruminococcus bromii	657321.5
Ruminococcus callidus ATCC 27760	Ruminococcus callidus	411473.3
Ruminococcus flavefaciens 007c	Ruminococcus flavefaciens	1341157.4
Ruminococcus flavefaciens 17	Ruminococcus flavefaciens	1030842.4
Ruminococcus flavefaciens AE3010	Ruminococcus flavefaciens	1384066.3
Ruminococcus flavefaciens ATCC 19208	Ruminococcus flavefaciens	1336236.3
Ruminococcus flavefaciens FD-1	Ruminococcus flavefaciens	641112.4
Ruminococcus flavefaciens MA2007	Ruminococcus flavefaciens	1410670.3
Ruminococcus flavefaciens MC2020	Ruminococcus flavefaciens	1410671.3
Ruminococcus flavefaciens ND2009	Ruminococcus flavefaciens	1410672.3
Ruminococcus gnavus AGR2154	[Ruminococcus] gnavus	1384063.4
Ruminococcus gnavus ATCC 29149	[Ruminococcus] gnavus	411470.6
Ruminococcus gnavus CC55_001C	[Ruminococcus] gnavus	1073375.3
Ruminococcus lactaris ATCC 29176	Ruminococcus lactaris	471875.6
Ruminococcus lactaris CC59_002D	Ruminococcus lactaris	1073376.3
Ruminococcus obeum A2-162	[Ruminococcus] obeum	657314.3
Ruminococcus obeum ATCC 29174	[Ruminococcus] obeum	411459.7
Ruminococcus torques ATCC 27756	[Ruminococcus] torques	411460.6
Ruminococcus torques L2-14	[Ruminococcus] torques	657313.3
Selenomonas artemidis DSM 19719	Selenomonas artemidis	1123249.3
Selenomonas artemidis F0399	Selenomonas artemidis	749551.3
Selenomonas flueggei ATCC 43531	Selenomonas flueggei	638302.3
Selenomonas infelix ATCC 43532	Selenomonas infelix	679201.3
Selenomonas noxia ATCC 43541	Selenomonas noxia	585503.3
Selenomonas noxia F0398	Selenomonas noxia	702437.3
Selenomonas ruminantium AB3002	Selenomonas ruminantium	1392502.3
Selenomonas ruminantium AC2024	Selenomonas ruminantium	1392501.3
Selenomonas ruminantium subsp. ruminantium ATCC 12561	Selenomonas ruminantium	1280706.4
Serratia fonticola AU-AP2C	Serratia fonticola	1332071.4
Serratia fonticola AU-P3(3)	Serratia fonticola	1332070.3
Serratia fonticola LMG 7882	Serratia fonticola	1378072.3
Serratia fonticola RB-25 [PRJNA232952]	Serratia fonticola	1441930.3
Serratia fonticola UTAD54	Serratia fonticola	1379259.4
Shewanella algae JCM 21037	Shewanella algae	1236544.3
Shewanella amazonensis SB2B	Shewanella amazonensis	326297.1
Shewanella baltica BA175	Shewanella baltica	693974.3
Shewanella baltica OS117	Shewanella baltica	693970.3
Shewanella baltica OS155	Shewanella baltica	325240.15
Shewanella baltica OS183	Shewanella baltica	693971.4

Shewanella baltica OS185	Shewanella baltica	402882.13
Shewanella baltica OS195	Shewanella baltica	399599.8
Shewanella baltica OS223	Shewanella baltica	407976.7
Shewanella baltica OS625	Shewanella baltica	693972.3
Shewanella baltica OS678	Shewanella baltica	693973.6
Shewanella colwelliana ATCC 39565	Shewanella colwelliana	1336240.3
Shewanella frigidimarina NCIMB 400	Shewanella frigidimarina	318167.14
Shewanella putrefaciens 200	Shewanella putrefaciens	399804.5
Shewanella putrefaciens CN-32	Shewanella putrefaciens	319224.16
Shewanella putrefaciens HRCR-6	Shewanella putrefaciens	1305841.3
Shewanella putrefaciens JCM 20190	Shewanella putrefaciens	1236543.3
Shewanella woodyi ATCC 51908	Shewanella woodyi	392500.6
Shimwellia blattae DSM 4481 = NBRC 105725	Shimwellia blattae	630626.3
Shuttleworthia satelles DSM 14600	Shuttleworthia satelles	626523.3
Solibacillus silvestris StLB046	Solibacillus silvestris	1002809.3
Solobacterium moorei DSM 22971	Solobacterium moorei	1123263.3
Solobacterium moorei F0204	Solobacterium moorei	706433.3
Sporolactobacillus inulinus CASD	Sporolactobacillus inulinus	1069536.3
Sporolactobacillus laevolacticus DSM 442	Sporolactobacillus laevolacticus	1395513.3
Sporolactobacillus terrae DSM 11697	Sporolactobacillus terrae	1444306.3
Sporolactobacillus terrae HKM-1	Sporolactobacillus terrae	1449983.3
Sporomusa ovata DSM 2662	Sporomusa ovata	1123288.3
Staphylococcus arlettae CVD059	Staphylococcus arlettae	1212545.3
Staphylococcus caprae C87	Staphylococcus capitis	435838.3
Staphylococcus chromogenes MU 970	Staphylococcus chromogenes	1220551.3
Staphylococcus delphini 8086	Staphylococcus delphini	1141105.7
Staphylococcus epidermidis M23864:W1	Staphylococcus caprae	525378.3
Staphylococcus hyicus ATCC 11249	Staphylococcus hyicus	1284.6
Staphylococcus intermedius NCTC 11048	Staphylococcus intermedius	1141106.7
Streptococcus anginosus 1_2_62CV	Streptococcus anginosus	742820.3
Streptococcus anginosus 1505	Streptococcus anginosus	1163301.3
Streptococcus anginosus C1051	Streptococcus anginosus	862970.3
Streptococcus anginosus C238	Streptococcus anginosus	862971.3
Streptococcus anginosus DORA_7	Streptococcus anginosus	1403946.3
Streptococcus anginosus F0211	Streptococcus anginosus	706437.3
Streptococcus anginosus SA1	Streptococcus anginosus	1328.12
Streptococcus anginosus SK1138	Streptococcus anginosus	1161422.3
Streptococcus anginosus SK52 = DSM 20563	Streptococcus anginosus	1000570.3
Streptococcus anginosus subsp. whileyi CCUG 39159	Streptococcus anginosus	1095729.3
Streptococcus anginosus subsp. whileyi MAS624	Streptococcus anginosus	1353243.3
Streptococcus anginosus T5	Streptococcus anginosus	1163302.3
Streptococcus bovis ATCC 700338	Streptococcus equinus	864569.5
Streptococcus bovis B315	Streptococcus equinus	1280690.3
Streptococcus bovis SN033	Streptococcus equinus	1280704.3
Streptococcus canis FSL Z3-227	Streptococcus canis	482234.3
Streptococcus criceti HS-6	Streptococcus criceti	873449.3
Streptococcus devriesei DSM 19639	Streptococcus devriesei	1123300.3
Streptococcus didelphis DSM 15616	Streptococcus didelphis	1123301.3
Streptococcus entericus DSM 14446	Streptococcus entericus	1123302.3
Streptococcus equinus	Streptococcus equinus	1335.4

Streptococcus equinus 2B	Streptococcus equinus	1410675.5
Streptococcus equinus ATCC 33317	Streptococcus equinus	1210006.5
Streptococcus equinus ATCC 9812	Streptococcus equinus	525379.3
Streptococcus equinus JB1	Streptococcus equinus	1294274.5
Streptococcus ferus DSM 20646	Streptococcus ferus	1123303.3
Streptococcus hyovaginalis DSM 12219	Streptococcus hyovaginalis	1123305.3
Streptococcus infantis ATCC 700779	Streptococcus infantis	889204.3
Streptococcus infantis SK1076	Streptococcus infantis	1005705.3
Streptococcus infantis SK1302	Streptococcus infantis	871237.3
Streptococcus infantis SK970	Streptococcus infantis	1035189.4
Streptococcus infantis X	Streptococcus infantis	997830.4
Streptococcus iniae	Streptococcus iniae	1346.13
Streptococcus iniae 9117	Streptococcus iniae	386894.6
Streptococcus iniae IUSA1	Streptococcus iniae	1273539.3
Streptococcus iniae KCTC 11634BP	Streptococcus iniae	1260129.3
Streptococcus iniae SF1	Streptococcus iniae	1318633.3
Streptococcus lutetiensis 033	Streptococcus lutetiensis	1076934.5
Streptococcus macacae NCTC 11558	Streptococcus macacae	764298.3
Streptococcus minor DSM 17118	Streptococcus minor	1123309.3
Streptococcus mutans 11A1	Streptococcus mutans	857155.3
Streptococcus mutans 11SSST2	Streptococcus mutans	857147.3
Streptococcus mutans 11VS1	Streptococcus mutans	857143.3
Streptococcus mutans 14D	Streptococcus mutans	857113.3
Streptococcus mutans 15JP3	Streptococcus mutans	857152.3
Streptococcus mutans 15VF2	Streptococcus mutans	857145.3
Streptococcus mutans 1ID3	Streptococcus mutans	857154.3
Streptococcus mutans 1SM1	Streptococcus mutans	857151.3
Streptococcus mutans 21	Streptococcus mutans	857112.3
Streptococcus mutans 24	Streptococcus mutans	857107.3
Streptococcus mutans 2ST1	Streptococcus mutans	857148.3
Streptococcus mutans 2VS1	Streptococcus mutans	857144.3
Streptococcus mutans 3SN1	Streptococcus mutans	857149.3
Streptococcus mutans 4SM1	Streptococcus mutans	857150.3
Streptococcus mutans 4VF1	Streptococcus mutans	857146.3
Streptococcus mutans 5DC8	Streptococcus mutans	1257037.3
Streptococcus mutans 5SM3	Streptococcus mutans	857142.3
Streptococcus mutans 66-2A	Streptococcus mutans	857111.3
Streptococcus mutans 8ID3	Streptococcus mutans	857153.3
Streptococcus mutans A19	Streptococcus mutans	857136.3
Streptococcus mutans A9	Streptococcus mutans	857139.3
Streptococcus mutans AC4446	Streptococcus mutans	1257040.3
Streptococcus mutans ATCC 25175	Streptococcus mutans	1257041.3
Streptococcus mutans B	Streptococcus mutans	857110.3
Streptococcus mutans B04Sm5	Streptococcus mutans	1225197.3
Streptococcus mutans B05Sm11	Streptococcus mutans	1225187.3
Streptococcus mutans B06Sm2	Streptococcus mutans	1225199.3
Streptococcus mutans B07Sm2	Streptococcus mutans	1225192.3
Streptococcus mutans B082SM-A	Streptococcus mutans	1225198.3
Streptococcus mutans B084SM-A	Streptococcus mutans	1225190.3
Streptococcus mutans B09Sm1	Streptococcus mutans	1225193.3

Streptococcus mutans B102SM-B	Streptococcus mutans	1225195.3
Streptococcus mutans B107SM-B	Streptococcus mutans	1225191.3
Streptococcus mutans B111SM-A	Streptococcus mutans	1225203.3
Streptococcus mutans B112SM-A	Streptococcus mutans	1225196.3
Streptococcus mutans B114SM-A	Streptococcus mutans	1225204.3
Streptococcus mutans B115SM-A	Streptococcus mutans	1225205.3
Streptococcus mutans B12Sm1	Streptococcus mutans	1225189.3
Streptococcus mutans B13Sm1	Streptococcus mutans	1225188.3
Streptococcus mutans B23Sm1	Streptococcus mutans	1225202.3
Streptococcus mutans B24Sm2	Streptococcus mutans	1225194.3
Streptococcus mutans B85SM-B	Streptococcus mutans	1225200.3
Streptococcus mutans B88SM-A	Streptococcus mutans	1225201.3
Streptococcus mutans DSM 20523	Streptococcus mutans	1123310.3
Streptococcus mutans G123	Streptococcus mutans	857134.3
Streptococcus mutans GS-5	Streptococcus mutans	1198676.3
Streptococcus mutans KK21	Streptococcus mutans	1257038.3
Streptococcus mutans KK23	Streptococcus mutans	1257039.3
Streptococcus mutans M21	Streptococcus mutans	857133.3
Streptococcus mutans M230	Streptococcus mutans	857100.3
Streptococcus mutans M2A	Streptococcus mutans	857126.3
Streptococcus mutans N29	Streptococcus mutans	857138.3
Streptococcus mutans N3209	Streptococcus mutans	857125.3
Streptococcus mutans N34	Streptococcus mutans	857131.3
Streptococcus mutans N66	Streptococcus mutans	857124.3
Streptococcus mutans NCTC 11060	Streptococcus mutans	1257042.3
Streptococcus mutans NFSM1	Streptococcus mutans	857130.3
Streptococcus mutans NFSM2	Streptococcus mutans	857141.3
Streptococcus mutans NLML1	Streptococcus mutans	857114.3
Streptococcus mutans NLML4	Streptococcus mutans	857129.3
Streptococcus mutans NLML5	Streptococcus mutans	857128.3
Streptococcus mutans NLML8	Streptococcus mutans	857115.3
Streptococcus mutans NLML9	Streptococcus mutans	857127.3
Streptococcus mutans NMT4863	Streptococcus mutans	857137.3
Streptococcus mutans NN2025	Streptococcus mutans	511691.3
Streptococcus mutans NV1996	Streptococcus mutans	857123.3
Streptococcus mutans NVAB	Streptococcus mutans	857140.4
Streptococcus mutans OMZ175	Streptococcus mutans	857099.3
Streptococcus mutans PKUSS-HG01	Streptococcus mutans	1403829.3
Streptococcus mutans PKUSS-LG01	Streptococcus mutans	1404260.3
Streptococcus mutans R221	Streptococcus mutans	857101.3
Streptococcus mutans S1B	Streptococcus mutans	857105.3
Streptococcus mutans SA38	Streptococcus mutans	857104.3
Streptococcus mutans SA41	Streptococcus mutans	857103.3
Streptococcus mutans SF1	Streptococcus mutans	857121.3
Streptococcus mutans SF12	Streptococcus mutans	857102.3
Streptococcus mutans SF14	Streptococcus mutans	857120.3
Streptococcus mutans SM1	Streptococcus mutans	857108.3
Streptococcus mutans SM4	Streptococcus mutans	857109.4
Streptococcus mutans SM6	Streptococcus mutans	857119.3
Streptococcus mutans ST1	Streptococcus mutans	857118.3

Streptococcus mutans ST6	Streptococcus mutans	857117.3
Streptococcus mutans str. B16 P Sm1	Streptococcus mutans	1225186.3
Streptococcus mutans T4	Streptococcus mutans	857132.3
Streptococcus mutans TCI-101	Streptococcus mutans	1074113.3
Streptococcus mutans TCI-109	Streptococcus mutans	1074114.3
Streptococcus mutans TCI-11	Streptococcus mutans	1074095.3
Streptococcus mutans TCI-110	Streptococcus mutans	1074115.3
Streptococcus mutans TCI-116	Streptococcus mutans	1074116.3
Streptococcus mutans TCI-120	Streptococcus mutans	1074118.3
Streptococcus mutans TCI-123	Streptococcus mutans	1074119.3
Streptococcus mutans TCI-125	Streptococcus mutans	1074120.3
Streptococcus mutans TCI-138	Streptococcus mutans	1074121.3
Streptococcus mutans TCI-143	Streptococcus mutans	1074122.3
Streptococcus mutans TCI-145	Streptococcus mutans	1074123.3
Streptococcus mutans TCI-146	Streptococcus mutans	1074124.3
Streptococcus mutans TCI-148	Streptococcus mutans	1074125.3
Streptococcus mutans TCI-149	Streptococcus mutans	1074126.3
Streptococcus mutans TCI-151	Streptococcus mutans	1074127.3
Streptococcus mutans TCI-152	Streptococcus mutans	1074128.3
Streptococcus mutans TCI-153	Streptococcus mutans	1074129.3
Streptococcus mutans TCI-154	Streptococcus mutans	1074130.3
Streptococcus mutans TCI-162	Streptococcus mutans	1074134.3
Streptococcus mutans TCI-163	Streptococcus mutans	1074135.3
Streptococcus mutans TCI-164	Streptococcus mutans	1074136.3
Streptococcus mutans TCI-169	Streptococcus mutans	1074137.3
Streptococcus mutans TCI-170	Streptococcus mutans	1074138.3
Streptococcus mutans TCI-173	Streptococcus mutans	1074140.3
Streptococcus mutans TCI-176	Streptococcus mutans	1074143.3
Streptococcus mutans TCI-177	Streptococcus mutans	1074144.3
Streptococcus mutans TCI-179	Streptococcus mutans	1074146.3
Streptococcus mutans TCI-187	Streptococcus mutans	1074148.3
Streptococcus mutans TCI-191	Streptococcus mutans	1074149.3
Streptococcus mutans TCI-196	Streptococcus mutans	1074151.3
Streptococcus mutans TCI-201	Streptococcus mutans	1074153.3
Streptococcus mutans TCI-202	Streptococcus mutans	1074154.3
Streptococcus mutans TCI-204	Streptococcus mutans	1074155.3
Streptococcus mutans TCI-210	Streptococcus mutans	1074156.3
Streptococcus mutans TCI-212	Streptococcus mutans	1074157.3
Streptococcus mutans TCI-218	Streptococcus mutans	1074159.3
Streptococcus mutans TCI-219	Streptococcus mutans	1074160.3
Streptococcus mutans TCI-220	Streptococcus mutans	1074161.3
Streptococcus mutans TCI-222	Streptococcus mutans	1074162.3
Streptococcus mutans TCI-223	Streptococcus mutans	1074163.3
Streptococcus mutans TCI-224	Streptococcus mutans	1074164.3
Streptococcus mutans TCI-227	Streptococcus mutans	1074165.3
Streptococcus mutans TCI-228	Streptococcus mutans	1074166.3
Streptococcus mutans TCI-234	Streptococcus mutans	1074167.3
Streptococcus mutans TCI-239	Streptococcus mutans	1074168.3
Streptococcus mutans TCI-242	Streptococcus mutans	1074169.3
Streptococcus mutans TCI-243	Streptococcus mutans	1074170.3

Streptococcus mutans TCI-244	Streptococcus mutans	1074171.3
Streptococcus mutans TCI-249	Streptococcus mutans	1074173.3
Streptococcus mutans TCI-256	Streptococcus mutans	1074175.3
Streptococcus mutans TCI-260	Streptococcus mutans	1074176.3
Streptococcus mutans TCI-264	Streptococcus mutans	1074177.3
Streptococcus mutans TCI-267	Streptococcus mutans	1074178.3
Streptococcus mutans TCI-268	Streptococcus mutans	1074179.3
Streptococcus mutans TCI-278	Streptococcus mutans	1074180.3
Streptococcus mutans TCI-279	Streptococcus mutans	1074181.3
Streptococcus mutans TCI-280	Streptococcus mutans	1074182.3
Streptococcus mutans TCI-289	Streptococcus mutans	1074183.3
Streptococcus mutans TCI-292	Streptococcus mutans	1074184.3
Streptococcus mutans TCI-294	Streptococcus mutans	1074185.3
Streptococcus mutans TCI-298	Streptococcus mutans	1074186.3
Streptococcus mutans TCI-30	Streptococcus mutans	1074190.3
Streptococcus mutans TCI-399	Streptococcus mutans	1074092.3
Streptococcus mutans TCI-400	Streptococcus mutans	1074093.3
Streptococcus mutans TCI-51	Streptococcus mutans	1074100.3
Streptococcus mutans TCI-62	Streptococcus mutans	1074101.3
Streptococcus mutans TCI-70	Streptococcus mutans	1074102.3
Streptococcus mutans TCI-75	Streptococcus mutans	1074104.3
Streptococcus mutans TCI-78	Streptococcus mutans	1074105.3
Streptococcus mutans TCI-82	Streptococcus mutans	1074106.3
Streptococcus mutans TCI-85	Streptococcus mutans	1074107.3
Streptococcus mutans TCI-86	Streptococcus mutans	1074108.3
Streptococcus mutans TCI-92	Streptococcus mutans	1074109.3
Streptococcus mutans TCI-96	Streptococcus mutans	1074111.3
Streptococcus mutans TCI-99	Streptococcus mutans	1074112.3
Streptococcus mutans U138	Streptococcus mutans	857135.3
Streptococcus mutans U2A	Streptococcus mutans	857116.3
Streptococcus mutans U2B	Streptococcus mutans	857106.3
Streptococcus mutans UA159	Streptococcus mutans	210007.7
Streptococcus mutans UA159-FR	Streptococcus mutans	1437447.3
Streptococcus mutans W6	Streptococcus mutans	857122.3
Streptococcus oligofermentans AS 1.3089	Streptococcus oligofermentans	1302863.3
Streptococcus orisratti DSM 15617	Streptococcus orisratti	1123311.3
Streptococcus ovis DSM 16829	Streptococcus ovis	1123312.3
Streptococcus parasanguinis ATCC 15912	Streptococcus parasanguinis	760570.3
Streptococcus parasanguinis ATCC 903	Streptococcus parasanguinis	888048.3
Streptococcus parasanguinis CC87K	Streptococcus parasanguinis	1073372.3
Streptococcus parasanguinis F0405	Streptococcus parasanguinis	905067.3
Streptococcus parasanguinis F0449	Streptococcus parasanguinis	1095733.3
Streptococcus parasanguinis FW213	Streptococcus parasanguinis	1114965.3
Streptococcus parasanguinis SK236	Streptococcus parasanguinis	1035185.3
Streptococcus parauberis	Streptococcus parauberis	1348.3
Streptococcus parauberis KCTC 11537	Streptococcus parauberis	936154.3
Streptococcus parauberis KCTC 11980BP	Streptococcus parauberis	1260132.3
Streptococcus parauberis KRS-02083	Streptococcus parauberis	1207545.3
Streptococcus parauberis KRS-02109	Streptococcus parauberis	1207544.3
Streptococcus parauberis NCFD 2020	Streptococcus parauberis	873447.3

Streptococcus phocae C-4	Streptococcus phocae	1000562.3
Streptococcus porcinus str. Jelinkova 176	Streptococcus porcinus	873448.3
Streptococcus ratti FA-1 = DSM 20564	Streptococcus ratti	699248.3
Streptococcus sanguinis ATCC 29667	Streptococcus sanguinis	997356.4
Streptococcus sanguinis CC94A	Streptococcus sanguinis	1073373.3
Streptococcus sanguinis SK1	Streptococcus sanguinis	888807.3
Streptococcus sanguinis SK1056	Streptococcus sanguinis	888820.3
Streptococcus sanguinis SK1057	Streptococcus sanguinis	888821.3
Streptococcus sanguinis SK1058	Streptococcus sanguinis	888822.3
Streptococcus sanguinis SK1059	Streptococcus sanguinis	888823.3
Streptococcus sanguinis SK1087	Streptococcus sanguinis	888824.3
Streptococcus sanguinis SK115	Streptococcus sanguinis	888810.3
Streptococcus sanguinis SK150	Streptococcus sanguinis	888811.3
Streptococcus sanguinis SK160	Streptococcus sanguinis	888812.3
Streptococcus sanguinis SK330	Streptococcus sanguinis	888813.3
Streptococcus sanguinis SK340	Streptococcus sanguinis	888814.4
Streptococcus sanguinis SK353	Streptococcus sanguinis	888815.3
Streptococcus sanguinis SK355	Streptococcus sanguinis	888816.3
Streptococcus sanguinis SK36	Streptococcus sanguinis	388919.9
Streptococcus sanguinis SK405	Streptococcus sanguinis	888817.3
Streptococcus sanguinis SK408	Streptococcus sanguinis	888818.3
Streptococcus sanguinis SK49	Streptococcus sanguinis	888808.3
Streptococcus sanguinis SK678	Streptococcus sanguinis	888819.3
Streptococcus sanguinis SK72	Streptococcus sanguinis	888809.3
Streptococcus sanguinis VMC66	Streptococcus sanguinis	888825.3
Streptococcus sinensis HKU4	Streptococcus sinensis	176090.4
Streptococcus sobrinus DSM 20742	Streptococcus sobrinus	1123317.3
Streptococcus sobrinus TCI-107	Streptococcus sobrinus	1074066.3
Streptococcus sobrinus TCI-118	Streptococcus sobrinus	1074117.3
Streptococcus sobrinus TCI-119	Streptococcus sobrinus	1074067.3
Streptococcus sobrinus TCI-121	Streptococcus sobrinus	1074068.3
Streptococcus sobrinus TCI-124	Streptococcus sobrinus	1074069.3
Streptococcus sobrinus TCI-13	Streptococcus sobrinus	1074053.3
Streptococcus sobrinus TCI-157	Streptococcus sobrinus	1074132.3
Streptococcus sobrinus TCI-16	Streptococcus sobrinus	1074054.3
Streptococcus sobrinus TCI-160	Streptococcus sobrinus	1074133.3
Streptococcus sobrinus TCI-172	Streptococcus sobrinus	1074139.3
Streptococcus sobrinus TCI-175	Streptococcus sobrinus	1074142.3
Streptococcus sobrinus TCI-194	Streptococcus sobrinus	1074150.3
Streptococcus sobrinus TCI-2	Streptococcus sobrinus	1074094.3
Streptococcus sobrinus TCI-200	Streptococcus sobrinus	1074152.3
Streptococcus sobrinus TCI-215	Streptococcus sobrinus	1074158.3
Streptococcus sobrinus TCI-28	Streptococcus sobrinus	1074055.3
Streptococcus sobrinus TCI-336	Streptococcus sobrinus	1074189.3
Streptococcus sobrinus TCI-342	Streptococcus sobrinus	1074070.3
Streptococcus sobrinus TCI-345	Streptococcus sobrinus	1074071.4
Streptococcus sobrinus TCI-348	Streptococcus sobrinus	1074072.3
Streptococcus sobrinus TCI-349	Streptococcus sobrinus	1074073.3
Streptococcus sobrinus TCI-352	Streptococcus sobrinus	1074074.3
Streptococcus sobrinus TCI-355	Streptococcus sobrinus	1074076.3

Streptococcus sobrinus TCI-357	Streptococcus sobrinus	1074077.3
Streptococcus sobrinus TCI-363	Streptococcus sobrinus	1074078.3
Streptococcus sobrinus TCI-366	Streptococcus sobrinus	1074079.3
Streptococcus sobrinus TCI-367	Streptococcus sobrinus	1074080.3
Streptococcus sobrinus TCI-373	Streptococcus sobrinus	1074081.3
Streptococcus sobrinus TCI-374	Streptococcus sobrinus	1074082.3
Streptococcus sobrinus TCI-376	Streptococcus sobrinus	1074083.3
Streptococcus sobrinus TCI-377	Streptococcus sobrinus	1074084.3
Streptococcus sobrinus TCI-381	Streptococcus sobrinus	1074085.3
Streptococcus sobrinus TCI-384	Streptococcus sobrinus	1074086.3
Streptococcus sobrinus TCI-392	Streptococcus sobrinus	1074088.3
Streptococcus sobrinus TCI-395	Streptococcus sobrinus	1074089.3
Streptococcus sobrinus TCI-396	Streptococcus sobrinus	1074090.3
Streptococcus sobrinus TCI-50	Streptococcus sobrinus	1074056.3
Streptococcus sobrinus TCI-53	Streptococcus sobrinus	1074057.3
Streptococcus sobrinus TCI-54	Streptococcus sobrinus	1074058.3
Streptococcus sobrinus TCI-56	Streptococcus sobrinus	1074059.3
Streptococcus sobrinus TCI-61	Streptococcus sobrinus	1074060.3
Streptococcus sobrinus TCI-77	Streptococcus sobrinus	1074061.3
Streptococcus sobrinus TCI-79	Streptococcus sobrinus	1074062.3
Streptococcus sobrinus TCI-80	Streptococcus sobrinus	1074063.3
Streptococcus sobrinus TCI-89	Streptococcus sobrinus	1074064.3
Streptococcus sobrinus TCI-9	Streptococcus sobrinus	1074052.3
Streptococcus sobrinus TCI-98	Streptococcus sobrinus	1074065.3
Streptococcus sobrinus W1703	Streptococcus sobrinus	1227275.3
Streptococcus suis 05HAS68	Streptococcus suis	672190.3
Streptococcus suis 05ZYH33	Streptococcus suis	391295.8
Streptococcus suis 07SC3	Streptococcus suis	1214149.3
Streptococcus suis 10581	Streptococcus suis	1214158.3
Streptococcus suis 11538	Streptococcus suis	1214180.3
Streptococcus suis 11611	Streptococcus suis	1214148.3
Streptococcus suis 12814	Streptococcus suis	1214156.3
Streptococcus suis 13730	Streptococcus suis	1214159.3
Streptococcus suis 14636	Streptococcus suis	1214183.3
Streptococcus suis 14A	Streptococcus suis	1214167.3
Streptococcus suis 161_00P5	Streptococcus suis	1214150.3
Streptococcus suis 22083	Streptococcus suis	1214184.3
Streptococcus suis 2524	Streptococcus suis	1214181.3
Streptococcus suis 2651	Streptococcus suis	1214154.3
Streptococcus suis 2726	Streptococcus suis	1214161.3
Streptococcus suis 4417	Streptococcus suis	1214155.3
Streptococcus suis 4961	Streptococcus suis	1214176.3
Streptococcus suis 6407	Streptococcus suis	1214179.3
Streptococcus suis 8074	Streptococcus suis	1214182.3
Streptococcus suis 86-5192	Streptococcus suis	1214166.3
Streptococcus suis 8830	Streptococcus suis	1214157.3
Streptococcus suis 89/1591	Streptococcus suis	286604.5
Streptococcus suis 89-1591	Streptococcus suis	1214151.3
Streptococcus suis 89-2479	Streptococcus suis	1214169.3
Streptococcus suis 89-3576-3	Streptococcus suis	1214171.3

Streptococcus suis 89-4109-1	Streptococcus suis	1214172.3
Streptococcus suis 89-5259	Streptococcus suis	1214173.3
Streptococcus suis 92-1191	Streptococcus suis	1214175.3
Streptococcus suis 92-1400	Streptococcus suis	1214177.3
Streptococcus suis 92-4172	Streptococcus suis	1214178.3
Streptococcus suis 93A	Streptococcus suis	1214162.3
Streptococcus suis 98HAH33	Streptococcus suis	391296.8
Streptococcus suis A7	Streptococcus suis	993512.3
Streptococcus suis BM407	Streptococcus suis	568814.3
Streptococcus suis D12	Streptococcus suis	1004952.3
Streptococcus suis D9	Streptococcus suis	1005042.3
Streptococcus suis EA1832.92	Streptococcus suis	1321372.3
Streptococcus suis GZ1	Streptococcus suis	423211.3
Streptococcus suis JS14	Streptococcus suis	945704.3
Streptococcus suis NT77	Streptococcus suis	1214163.3
Streptococcus suis P1/7	Streptococcus suis	218494.6
Streptococcus suis R61	Streptococcus suis	996306.3
Streptococcus suis R735	Streptococcus suis	1214165.3
Streptococcus suis RC1	Streptococcus suis	1214152.3
Streptococcus suis S15	Streptococcus suis	1214160.3
Streptococcus suis S19	Streptococcus suis	1214164.3
Streptococcus suis S22	Streptococcus suis	1214168.3
Streptococcus suis S24	Streptococcus suis	1214170.3
Streptococcus suis S28	Streptococcus suis	1214174.3
Streptococcus suis S428	Streptococcus suis	1214153.3
Streptococcus suis S735	Streptococcus suis	1184252.3
Streptococcus suis SC070731	Streptococcus suis	1246365.4
Streptococcus suis SC84	Streptococcus suis	568813.3
Streptococcus suis SS12	Streptococcus suis	1005041.3
Streptococcus suis ST1	Streptococcus suis	1004951.3
Streptococcus suis ST3	Streptococcus suis	1007064.3
Streptococcus suis T15	Streptococcus suis	1340847.3
Streptococcus suis TL13	Streptococcus suis	1276647.3
Streptococcus suis YB51	Streptococcus suis	1380773.3
Streptococcus suis YS1	Streptococcus suis	1214185.3
Streptococcus suis YS10-2	Streptococcus suis	1214186.3
Streptococcus suis YS12	Streptococcus suis	1214187.3
Streptococcus suis YS14	Streptococcus suis	1214188.3
Streptococcus suis YS16	Streptococcus suis	1214189.3
Streptococcus suis YS17-2	Streptococcus suis	1214190.3
Streptococcus suis YS19-3	Streptococcus suis	1214191.3
Streptococcus suis YS21	Streptococcus suis	1214192.3
Streptococcus suis YS23-2	Streptococcus suis	1214193.3
Streptococcus suis YS24	Streptococcus suis	1214194.3
Streptococcus suis YS27-2	Streptococcus suis	1214195.3
Streptococcus suis YS3	Streptococcus suis	1214196.3
Streptococcus suis YS31	Streptococcus suis	1214197.3
Streptococcus suis YS34	Streptococcus suis	1214198.3
Streptococcus suis YS35	Streptococcus suis	1214199.3
Streptococcus suis YS39	Streptococcus suis	1214200.3

Streptococcus suis YS4	Streptococcus suis	1214201.3
Streptococcus suis YS43	Streptococcus suis	1214202.3
Streptococcus suis YS44	Streptococcus suis	1214203.3
Streptococcus suis YS46	Streptococcus suis	1214204.3
Streptococcus suis YS49	Streptococcus suis	1214205.3
Streptococcus suis YS50	Streptococcus suis	1214206.3
Streptococcus suis YS53	Streptococcus suis	1214207.3
Streptococcus suis YS54-2	Streptococcus suis	1214208.3
Streptococcus suis YS56	Streptococcus suis	1214209.3
Streptococcus suis YS57	Streptococcus suis	1214210.3
Streptococcus suis YS59	Streptococcus suis	1214211.3
Streptococcus suis YS6	Streptococcus suis	1214212.3
Streptococcus suis YS64	Streptococcus suis	1214213.3
Streptococcus suis YS66	Streptococcus suis	1214214.3
Streptococcus suis YS67	Streptococcus suis	1214215.3
Streptococcus suis YS7	Streptococcus suis	1214216.3
Streptococcus suis YS72	Streptococcus suis	1214217.3
Streptococcus suis YS74	Streptococcus suis	1214218.3
Streptococcus suis YS77	Streptococcus suis	1214219.3
Streptococcus thermophilus ASCC 1275	Streptococcus thermophilus	1408178.4
Streptococcus thermophilus CNCM I-1630	Streptococcus thermophilus	1042404.3
Streptococcus thermophilus CNRZ1066	Streptococcus thermophilus	299768.6
Streptococcus thermophilus DGCC7710	Streptococcus thermophilus	1268061.3
Streptococcus thermophilus JIM 8232	Streptococcus thermophilus	1051074.3
Streptococcus thermophilus LMD-9	Streptococcus thermophilus	322159.8
Streptococcus thermophilus LMG 18311	Streptococcus thermophilus	264199.4
Streptococcus thermophilus MN-ZLW-002	Streptococcus thermophilus	1187956.3
Streptococcus thermophilus MTCC 5460	Streptococcus thermophilus	1073569.3
Streptococcus thermophilus MTCC 5461	Streptococcus thermophilus	1073570.5
Streptococcus thermophilus ND03	Streptococcus thermophilus	767463.3
Streptococcus thoraltensis DSM 12221	Streptococcus thoraltensis	1123318.3
Streptococcus urinalis 2285-97	Streptococcus urinalis	764291.3
Streptococcus urinalis FB127-CNA-2	Streptococcus urinalis	883168.3
Syntrophothermus lipocalidus DSM 12680	Syntrophothermus lipocalidus	643648.3
Tetragenococcus muriaticus 3MR10-3	Tetragenococcus muriaticus	1302648.3
Tetragenococcus muriaticus DSM 15685	Tetragenococcus muriaticus	1123359.3
Tetragenococcus muriaticus PMC-11-5	Tetragenococcus muriaticus	1302649.3
Thiomicrospira chilensis DSM 12352	Thiomicrospira chilensis	1123515.3
Thiomicrospira crunogena XCL-2	Thiomicrospira crunogena	317025.9
Thiomicrospira kuenenii DSM 12350	Thiomicrospira kuenenii	1168067.3
Thiomicrospira pelophila DSM 1534	Thiomicrospira pelophila	1123517.3
Virgibacillus halodenitrificans	Virgibacillus halodenitrificans	1482.4
Virgibacillus halodenitrificans 1806	Virgibacillus halodenitrificans	1196028.3
Weissella cibaria KACC 11862	Weissella cibaria	911104.3
Weissella confusa LBAE C39-2	Weissella confusa	1127131.3
Weissella halotolerans DSM 20190	Weissella halotolerans	1123500.3
Weissella hellenica	Weissella hellenica	46256.5
Weissella koreensis KACC 15510	Weissella koreensis	1045854.4
Weissella koreensis KCTC 3621	Weissella koreensis	1123721.3
Weissella paramesenteroides ATCC 33313	Weissella paramesenteroides	585506.3

Xanthomonas albilineans	Xanthomonas albilineans	29447.3
Xanthomonas campestris JX	Xanthomonas campestris	1182783.3
Xanthomonas campestris LMCP11	Xanthomonas campestris	339.49
Xanthomonas campestris pv. arecae NCPPB 2649	Xanthomonas campestris	487849.3
Xanthomonas campestris pv. campestris str. 8004	Xanthomonas campestris	314565.5
Xanthomonas campestris pv. campestris str. ATCC 33913	Xanthomonas campestris	190485.4
Xanthomonas campestris pv. campestris str. B100	Xanthomonas campestris	509169.4
Xanthomonas campestris pv. campestris str. Xca5	Xanthomonas campestris	1211707.3
Xanthomonas campestris pv. cannabis NCPPB 2877	Xanthomonas campestris	92824.15
Xanthomonas campestris pv. musacearum 'Kenyan'	Xanthomonas campestris	1075759.3
Xanthomonas campestris pv. musacearum NCPPB 2005	Xanthomonas campestris	1094183.3
Xanthomonas campestris pv. musacearum NCPPB 4379	Xanthomonas campestris	1094184.3
Xanthomonas campestris pv. musacearum NCPPB 4380	Xanthomonas campestris	1094185.3
Xanthomonas campestris pv. musacearum NCPPB 4381	Xanthomonas campestris	559737.3
Xanthomonas campestris pv. musacearum NCPPB 4384	Xanthomonas campestris	1094186.4
Xanthomonas campestris pv. musacearum NCPPB 4392	Xanthomonas campestris	1184263.3
Xanthomonas campestris pv. musacearum NCPPB 4394	Xanthomonas campestris	1094187.3
Xanthomonas campestris pv. raphani 756C	Xanthomonas campestris	990315.4
Xanthomonas campestris pv. viticola LMG 965	Xanthomonas campestris	487899.3
Xanthomonas fragariae LMG 25863	Xanthomonas fragariae	1131451.6
Xanthomonas oryzae ATCC 35933	Xanthomonas oryzae	1313303.3
Xanthomonas oryzae pv. oryzae KACC 10331	Xanthomonas oryzae	291331.8
Xanthomonas oryzae pv. oryzae MAFF 311018	Xanthomonas oryzae	342109.8
Xanthomonas oryzae pv. oryzae NAI8	Xanthomonas oryzae	1423889.3
Xanthomonas oryzae pv. oryzae PXO99A	Xanthomonas oryzae	360094.4
Xanthomonas oryzae pv. oryzicola BLS256	Xanthomonas oryzae	383407.3
Xanthomonas oryzae pv. oryzicola MAI10	Xanthomonas oryzae	1423890.3
Xanthomonas oryzae X11-5A	Xanthomonas oryzae	1009853.4
Xanthomonas oryzae X8-1A	Xanthomonas oryzae	1009854.4
Xenorhabdus bovienii SS-2004	Xenorhabdus bovienii	406818.4
Xenorhabdus nematophila C2-3	Xenorhabdus nematophila	628.3
Xenorhabdus nematophila F1	Xenorhabdus nematophila	1306162.3
Xylella fastidiosa 32	Xylella fastidiosa	1214121.5
Xylella fastidiosa 6c	Xylella fastidiosa	1211847.5
Xylella fastidiosa 9a5c	Xylella fastidiosa	160492.11
Xylella fastidiosa ATCC 35879	Xylella fastidiosa	2371.35
Xylella fastidiosa Dixon	Xylella fastidiosa	155919.4
Xylella fastidiosa EB92.1	Xylella fastidiosa	945689.3
Xylella fastidiosa M12	Xylella fastidiosa	405440.5
Xylella fastidiosa M23	Xylella fastidiosa	405441.5
Xylella fastidiosa MUL0034	Xylella fastidiosa	1401256.4
Xylella fastidiosa Mul-MD	Xylella fastidiosa	1403344.3
Xylella fastidiosa PLS229	Xylella fastidiosa	1444770.3
Xylella fastidiosa subsp. fastidiosa GB514	Xylella fastidiosa	788929.3
Xylella fastidiosa subsp. multiplex ATCC 35871	Xylella fastidiosa	1267006.3
Xylella fastidiosa subsp. multiplex str. Red Oak 1	Xylella fastidiosa	1343737.3
Xylella fastidiosa subsp. sandyi Ann-1	Xylella fastidiosa	155920.4
Xylella fastidiosa Temecula1	Xylella fastidiosa	183190.5

(a) Strain designation

(b) Species name

(c) NCBI taxonomy strain id
