# Supplementary figures and images for: From Genomes to Phenotypes: Traitar, the Microbial Trait Analyzer
Source: mSystems. 2016 Dec 27;1(6):e00101-16. doi: 10.1128/mSystems.00101-16 (PMC5192078; doi:10.1128/mSystems.00101-16)

Supplementary Figure 1

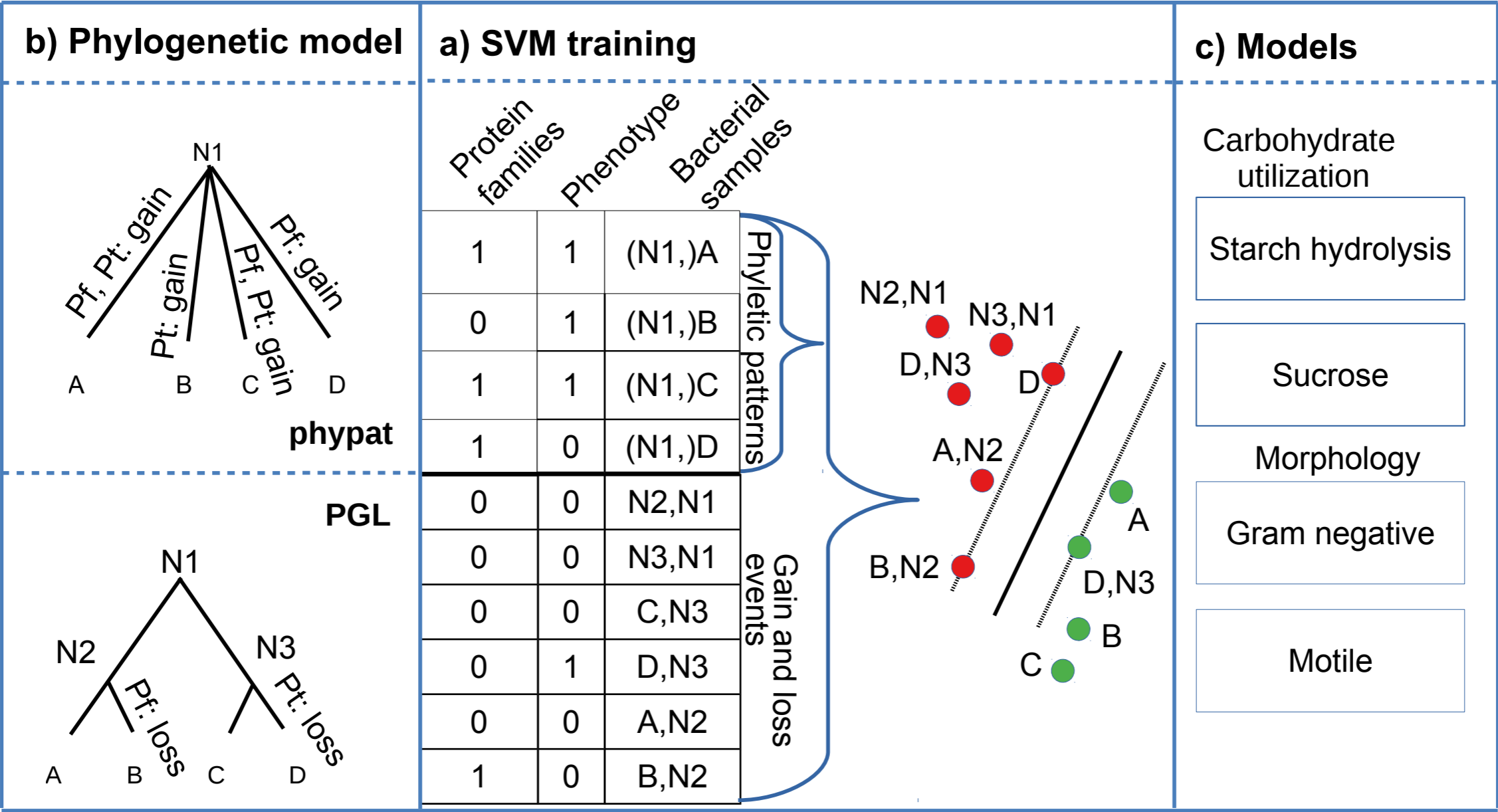

Supplement: Figure S1 [file sys006162072sf7.pdf]

Supplementary Figure 2

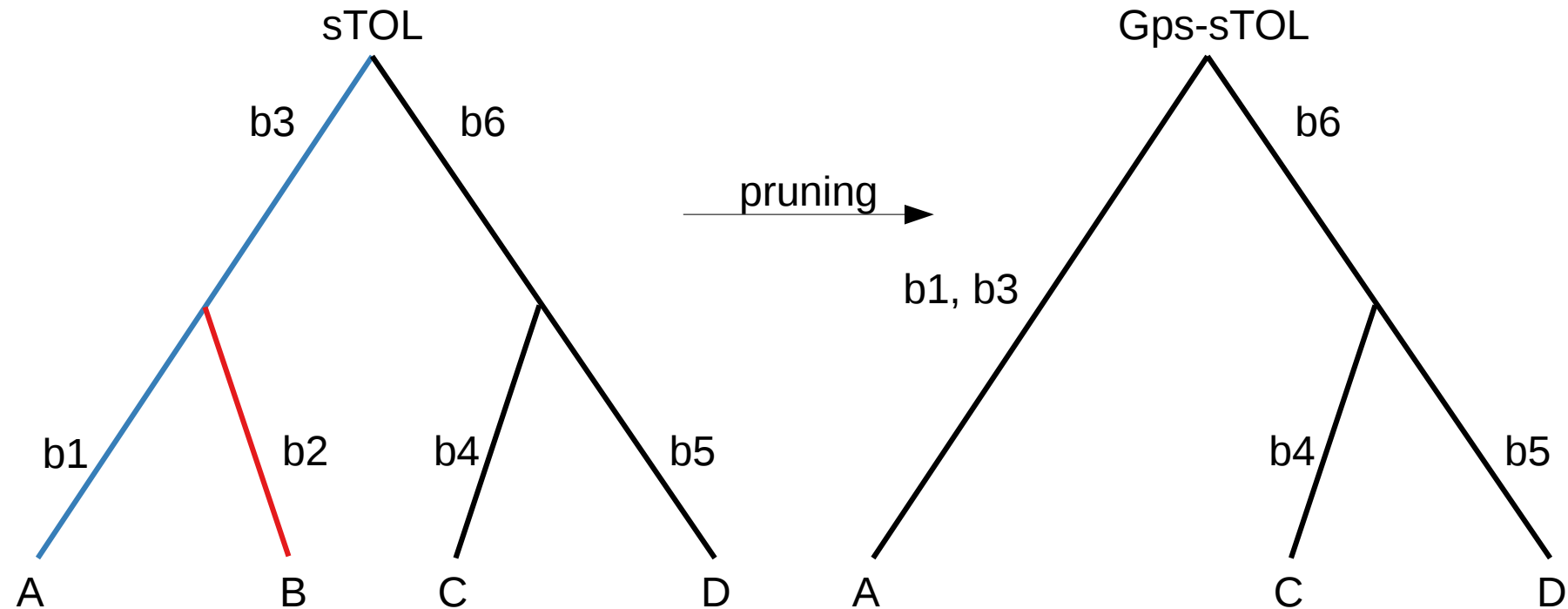

Supplement: Figure S2 [file sys006162072sf8.pdf]
