# Supplementary material for: From Genomes to Phenotypes: Traitar, the Microbial Trait Analyzer
Source: mSystems. 2016 Dec 27;1(6):e00101-16. doi: 10.1128/mSystems.00101-16 (PMC5192078; doi:10.1128/mSystems.00101-16)
Supplement: Table S6 [file sys006162072st6.pdf]

Phenotype: Alkaline phosphatase (Enzyme), Predictor: phypat

Pfam	class	Pfam_desc	cor
PF02872	+	5'-nucleotidase, C-terminal domain	0.589
PF04357	+	Family of unknown function (DUF490)	0.500
PF03767	+	HAD superfamily, subfamily IIIB (Acid phosphatase)	0.481
PF04228	+	Putative neutral zinc metallopeptidase	0.472
PF02817	+	e3 binding domain	0.426
PF00221	+	Aromatic amino acid lyase	0.414
PF13172	+	PepSY-associated TM helix	0.414
PF01175	+	Urocanase	0.385
PF01037	+	AsnC family	0.378
PF07463	+	NUMOD4 motif	0.329
PF01906	+	Putative heavy-metal-binding	0.328
PF01841	+	Transglutaminase-like superfamily	0.318
PF00723	+	Glycosyl hydrolases family 15	0.303
PF12837	+	4Fe-4S binding domain	0.253
PF13506	+	Glycosyl transferase family 21	0.251
PF05222	+	Alanine dehydrogenase/PNT, N-terminal domain	0.192
PF01501	+	Glycosyl transferase family 8	0.183
PF02350	+	UDP-N-acetylglucosamine 2-epimerase	0.094
PF09278	-	MerR, DNA binding	-0.158
PF05065	-	Phage capsid family	-0.189
PF00255	-	Glutathione peroxidase	-0.192
PF02541	-	Ppx/GppA phosphatase family	-0.200
PF13426	-	PAS domain	-0.251
PF13173	-	AAA domain	-0.259
PF13599	-	Pentapeptide repeats (9 copies)	-0.277
PF06953	-	Arsenical resistance operon trans-acting repressor ArsD	-0.288
PF01396	-	Topoisomerase DNA binding C4 zinc finger	-0.289
PF03596	-	Cadmium resistance transporter	-0.300
PF09954	-	Uncharacterized protein conserved in bacteria (DUF2188)	-0.302
PF02110	-	Hydroxyethylthiazole kinase family	-0.316
PF01680	-	SOR/SNZ family	-0.318
PF14690	-	zinc-finger of transposase IS204/IS1001/IS1096/IS1165	-0.324
PF03275	-	UDP-galactopyranose mutase	-0.324
PF01183	-	Glycosyl hydrolases family 25	-0.328
PF00209	-	Sodium:neurotransmitter symporter family	-0.328
PF12890	-	Dihydro-orotase-like	-0.348
PF02583	-	Metal-sensitive transcriptional repressor	-0.378
PF12974	-	ABC transporter, phosphonate, periplasmic substrate-binding protein	-0.378
PF01934	-	Protein of unknown function DUF86	-0.381
PF02503	-	Polyphosphate kinase middle domain	-0.385
PF13090	-	Polyphosphate kinase C-terminal domain	-0.385
PF13089	-	Polyphosphate kinase N-terminal domain	-0.385
PF05133	-	Phage portal protein, SPP1 Gp6-like	-0.385
PF12900	-	Pyridoxamine 5'-phosphate oxidase	-0.385
PF02686	-	Glu-tRNAGln amidotransferase C subunit	-0.402
PF13241	-	Putative NAD(P)-binding	-0.421

PF06050	-	2-hydroxyglutaryl-CoA dehydratase, D-component	-0.555
PF09989	-	CoA enzyme activase uncharacterised domain (DUF2229)	-0.555

Phenotype: Alkaline phosphatase (Enzyme), Predictor: phypat+PGL

Pfam	class	Pfam_desc	cor
PF02872	+	5'-nucleotidase, C-terminal domain	0.589
PF03767	+	HAD superfamily, subfamily IIIB (Acid phosphatase)	0.481
PF13172	+	PepSY-associated TM helix	0.414
PF01075	+	Glycosyltransferase family 9 (heptosyltransferase)	0.385
PF00485	+	Phosphoribulokinase / Uridine kinase family	0.354
PF13905	+	Thioredoxin-like	0.350
PF01738	+	Dienelactone hydrolase family	0.348
PF00756	+	Putative esterase	0.347
PF01906	+	Putative heavy-metal-binding	0.328
PF13519	+	von Willebrand factor type A domain	0.324
PF02353	+	Mycolic acid cyclopropane synthetase	0.320
PF01551	+	Peptidase family M23	0.320
PF00723	+	Glycosyl hydrolases family 15	0.303
PF08211	+	Cytidine and deoxycytidylate deaminase zinc-binding region	0.300
PF07819	+	PGAP1-like protein	0.289
PF10423	+	Bacterial AMP nucleoside phosphorylase N-terminus	0.277
PF13545	+	Crp-like helix-turn-helix domain	0.256
PF13280	+	WYL domain	0.256
PF01192	+	RNA polymerase Rpb6	0.256
PF04140	+	Isoprenylcysteine carboxyl methyltransferase (ICMT) family	0.253
PF13506	+	Glycosyl transferase family 21	0.251
PF12481	+	Aluminium induced protein	0.250
PF08363	+	Glucan-binding protein C	0.250
PF07786	+	Protein of unknown function (DUF1624)	0.250
PF01464	+	Transglycosylase SLT domain	0.238
PF03824	+	High-affinity nickel-transport protein	0.230
PF01597	+	Glycine cleavage H-protein	0.221
PF14399	+	NlpC/p60-like transpeptidase	0.221
PF12846	+	AAA-like domain	0.213
PF01262	+	Alanine dehydrogenase/PNT, C-terminal domain	0.192
PF13601	+	Winged helix DNA-binding domain	0.189
PF03848	+	Tellurite resistance protein TehB	0.189
PF14559	+	Tetratricopeptide repeat	0.189
PF13500	+	AAA domain	0.189
PF13185	+	GAF domain	0.189
PF13632	+	Glycosyl transferase family group 2	0.173
PF03949	+	Malic enzyme, NAD binding domain	0.173
PF02347	+	Glycine cleavage system P-protein	0.158
PF13515	+	Fusaric acid resistance protein-like	0.146
PF01730	+	UreF	0.139
PF05015	-	Plasmid maintenance system killer protein	0.107
PF00311	-	Phosphoenolpyruvate carboxylase	0.094
PF00515	+	Tetratricopeptide repeat	0.094

PF11361	+	Protein of unknown function (DUF3159)	0.082
PF03352	+	Methyladenine glycosylase	0.076
PF01361	+	Tautomerase enzyme	0.063
PF05016	+	Plasmid stabilisation system protein	0.063
PF00585	-	C-terminal regulatory domain of Threonine dehydratase	0.063
PF12669	+	Virus attachment protein p12 family	0.055
PF09992	+	Predicted periplasmic protein (DUF2233)	0.055
PF09643	+	YopX protein	0.038
PF08402	-	TOBE domain	0.034
PF03641	-	Possible lysine decarboxylase	0.034
PF05656	+	Protein of unknown function (DUF805)	0.033
PF12161	+	HsdM N-terminal domain	0.032
PF02913	-	FAD linked oxidases, C-terminal domain	-0.000
PF02498	-	BRO family, N-terminal domain	-0.000
PF09382	-	RQC domain	-0.000
PF02574	-	Homocysteine S-methyltransferase	-0.031
PF05495	-	CHY zinc finger	-0.069
PF02065	-	Melibiase	-0.069
PF01943	-	Polysaccharide biosynthesis protein	-0.082
PF07885	-	Ion channel	-0.094
PF13425	-	O-antigen ligase like membrane protein	-0.100
PF12146	-	Putative lysophospholipase	-0.100
PF06993	-	Protein of unknown function (DUF1304)	-0.107
PF06271	-	RDD family	-0.126
PF13558	-	Putative exonuclease SbcCD, C subunit	-0.126
PF12564	+	Type III restriction/modification enzyme methylation subunit	-0.139
PF01925	-	Sulfite exporter TauE/SafE	-0.139
PF00202	-	Aminotransferase class-III	-0.151
PF02378	-	Phosphotransferase system, EIIC	-0.170
PF01855	-	Pyruvate flavodoxin/ferredoxin oxidoreductase, thiamine diP-bdg	-0.189
PF12724	-	Flavodoxin domain	-0.192
PF13527	-	Acetyltransferase (GNAT) domain	-0.200
PF01297	-	Periplasmic solute binding protein family	-0.205
PF01261	-	Xylose isomerase-like TIM barrel	-0.213
PF09084	-	NMT1/THI5 like	-0.230
PF02597	-	ThiS family	-0.230
PF13426	-	PAS domain	-0.251
PF12459	-	D-Ala-teichoic acid biosynthesis protein	-0.256
PF13173	-	AAA domain	-0.259
PF06103	-	Bacterial protein of unknown function (DUF948)	-0.263
PF02581	-	Thiamine monophosphate synthase/TENI	-0.265
PF08502	-	LeuA allosteric (dimerisation) domain	-0.272
PF13710	-	ACT domain	-0.272
PF05738	-	Cna protein B-type domain	-0.283
PF02082	-	Transcriptional regulator	-0.289
PF01396	-	Topoisomerase DNA binding C4 zinc finger	-0.289
PF02275	-	Linear amide C-N hydrolases, choloylglycine hydrolase family	-0.302
PF06838	-	Methionine gamma-lyase	-0.302

PF08863	-	YolD-like protein	-0.302
PF02110	-	Hydroxyethylthiazole kinase family	-0.316
PF01680	-	SOR/SNZ family	-0.318
PF00180	-	Isocitrate/isopropylmalate dehydrogenase	-0.320
PF02535	-	ZIP Zinc transporter	-0.320
PF02361	-	Cobalt transport protein	-0.320
PF14690	-	zinc-finger of transposase IS204/IS1001/IS1096/IS1165	-0.324
PF00209	-	Sodium:neurotransmitter symporter family	-0.328
PF02659	-	Domain of unknown function DUF	-0.347
PF13089	-	Polyphosphate kinase N-terminal domain	-0.385
PF02503	-	Polyphosphate kinase middle domain	-0.385

Phenotype: Beta hemolysis (Enzyme), Predictor: phypat

Pfam	class	Pfam_desc	cor
PF05031	+	Iron Transport-associated domain	0.471
PF00217	+	ATP:guanido phosphotransferase, C-terminal catalytic domain	0.470
PF10960	+	Protein of unknown function (DUF2762)	0.449
PF06605	+	Prophage endopeptidase tail	0.444
PF13514	+	AAA domain	0.408
PF12363	+	Phage protein	0.405
PF05135	+	Phage gp6-like head-tail connector protein	0.387
PF14265	+	Domain of unknown function (DUF4355)	0.387
PF05738	+	Cna protein B-type domain	0.379
PF04235	+	Protein of unknown function (DUF418)	0.374
PF11193	+	Protein of unknown function (DUF2812)	0.374
PF04286	+	Protein of unknown function (DUF445)	0.372
PF02868	+	Thermolysin metallopeptidase, alpha-helical domain	0.370
PF01447	+	Thermolysin metallopeptidase, catalytic domain	0.370
PF04183	+	IucA / IucC family	0.362
PF01289	+	Thiol-activated cytolysin	0.339
PF02624	+	YcaO-like family	0.336
PF00874	+	PRD domain	0.331
PF13620	+	Carboxypeptidase regulatory-like domain	0.314
PF05521	+	Phage head-tail joining protein	0.312
PF06114	+	Domain of unknown function (DUF955)	0.312
PF03454	+	MoeA C-terminal region (domain IV)	0.310
PF03703	+	Bacterial PH domain	0.305
PF04066	+	Multiple resistance and pH regulation protein F (MrpF / PhaF)	0.298
PF02436	+	Conserved carboxylase domain	0.295
PF01928	+	CYTH domain	0.271
PF02690	+	Na+/Pi-cotransporter	0.259
PF12729	+	Four helix bundle sensory module for signal transduction	0.259
PF10340	+	Protein of unknown function (DUF2424)	0.251
PF05076	+	Suppressor of fused protein (SUFU)	0.246
PF12835	+	Integrase	0.243
PF01244	+	Membrane dipeptidase (Peptidase family M19)	0.222
PF03595	+	Voltage-dependent anion channel	0.202
PF01797	+	Transposase IS200 like	0.184

PF03616	+	Sodium/glutamate symporter	0.177
PF00821	+	Phosphoenolpyruvate carboxykinase	0.176
PF02614	+	Glucuronate isomerase	0.153
PF03786	+	D-mannonate dehydratase (UxuA)	0.145
PF02633	+	Creatinine amidohydrolase	0.133
PF01930	+	Domain of unknown function DUF83	0.131
PF13333	-	Integrase core domain	0.051
PF02926	-	THUMP domain	0.030
PF00239	-	Resolvase, N terminal domain	0.015
PF04138	-	GtrA-like protein	0.012
PF00145	-	C-5 cytosine-specific DNA methylase	-0.003
PF13183	-	4Fe-4S dicluster domain	-0.017
PF01396	-	Topoisomerase DNA binding C4 zinc finger	-0.018
PF04892	-	VanZ like family	-0.024
PF03601	-	Conserved hypothetical protein 698	-0.024
PF13483	-	Beta-lactamase superfamily domain	-0.030
PF04230	-	Polysaccharide pyruvyl transferase	-0.045
PF01965	-	DJ-1/PfpI family	-0.048
PF01548	-	Transposase	-0.051
PF02781	-	Glucose-6-phosphate dehydrogenase, C-terminal domain	-0.058
PF00479	-	Glucose-6-phosphate dehydrogenase, NAD binding domain	-0.058
PF00221	-	Aromatic amino acid lyase	-0.069
PF01566	-	Natural resistance-associated macrophage protein	-0.074
PF01717	-	Cobalamin-independent synthase, Catalytic domain	-0.079
PF01555	-	DNA methylase	-0.085
PF13191	-	AAA ATPase domain	-0.089
PF01161	-	Phosphatidylethanolamine-binding protein	-0.092
PF13950	-	UDP-glucose 4-epimerase C-term subunit	-0.104
PF06912	-	Protein of unknown function (DUF1275)	-0.108
PF05362	-	Lon protease (S16) C-terminal proteolytic domain	-0.114
PF12773	-	Double zinc ribbon	-0.127
PF02929	-	Beta galactosidase small chain	-0.135
PF05016	-	Plasmid stabilisation system protein	-0.152
PF02604	-	Antitoxin Phd_YefM, type II toxin-antitoxin system	-0.161
PF02706	-	Chain length determinant protein	-0.162
PF01343	-	Peptidase family S49	-0.177
PF13089	-	Polyphosphate kinase N-terminal domain	-0.182
PF06418	-	CTP synthase N-terminus	-0.187
PF02503	-	Polyphosphate kinase middle domain	-0.188
PF02272	-	DHHA1 domain	-0.194
PF13340	-	Putative transposase of IS4/5 family (DUF4096)	-0.197
PF00254	-	FKBP-type peptidyl-prolyl cis-trans isomerase	-0.222
PF14821	-	Threonine synthase N terminus	-0.227
PF04011	-	LemA family	-0.229
PF05973	-	Phage derived protein Gp49-like (DUF891)	-0.234

Phenotype: Beta hemolysis (Enzyme), Predictor: phypat+PGL

Pfam	class	Pfam_desc	cor

PF06605	+	Prophage endopeptidase tail	0.444
PF12363	+	Phage protein	0.405
PF05565	+	Siphovirus Gp157	0.400
PF13848	+	Thioredoxin-like domain	0.387
PF05135	+	Phage gp6-like head-tail connector protein	0.387
PF14265	+	Domain of unknown function (DUF4355)	0.387
PF11193	+	Protein of unknown function (DUF2812)	0.374
PF02868	+	Thermolysin metallopeptidase, alpha-helical domain	0.370
PF01447	+	Thermolysin metallopeptidase, catalytic domain	0.370
PF04183	+	IucA / IucC family	0.362
PF07194	+	P2 response regulator binding domain	0.357
PF11070	+	Protein of unknown function (DUF2871)	0.357
PF07299	+	Fibronectin-binding protein (FBP)	0.354
PF03243	+	Alkylmercury lyase	0.346
PF15542	+	Putative toxin 64	0.339
PF01289	+	Thiol-activated cytolysin	0.339
PF02624	+	YcaO-like family	0.336
PF04531	+	Bacteriophage holin	0.322
PF02679	+	(2R)-phospho-3-sulfolactate synthase (ComA)	0.321
PF08010	+	Bacteriophage protein GP30.3	0.315
PF08309	+	LVIVD repeat	0.315
PF13620	+	Carboxypeptidase regulatory-like domain	0.314
PF05521	+	Phage head-tail joining protein	0.312
PF04630	+	Phage major tail protein	0.307
PF13783	+	Domain of unknown function (DUF4177)	0.307
PF04466	+	Phage terminase large subunit	0.307
PF04066	+	Multiple resistance and pH regulation protein F (MrpF / PhaF)	0.298
PF02436	+	Conserved carboxylase domain	0.295
PF03142	+	Chitin synthase	0.292
PF06152	+	Phage minor capsid protein 2	0.291
PF08937	+	MTH538 TIR-like domain (DUF1863)	0.279
PF12811	+	Bax inhibitor 1 like	0.277
PF01905	+	CRISPR-associated negative auto-regulator DevR/Csa2	0.275
PF08817	+	WXG100 protein secretion system (Wss), protein YukD	0.272
PF13244	+	Domain of unknown function (DUF4040)	0.267
PF12686	+	Protein of unknown function (DUF3800)	0.266
PF13730	+	Helix-turn-helix domain	0.263
PF12729	+	Four helix bundle sensory module for signal transduction	0.259
PF00882	+	Zinc dependent phospholipase C	0.258
PF10340	+	Protein of unknown function (DUF2424)	0.251
PF05076	+	Suppressor of fused protein (SUFU)	0.246
PF00708	+	Acylphosphatase	0.246
PF12835	+	Integrase	0.243
PF03592	+	Terminase small subunit	0.236
PF09586	+	Bacterial membrane protein YfhO	0.220
PF02901	+	Pyruvate formate lyase	0.213
PF06634	+	Protein of unknown function (DUF1156)	0.211
PF13274	+	Protein of unknown function (DUF4065)	0.204

PF07694	-	5TMR of 5TMR-LYT	0.204
PF02652	+	L-lactate permease	0.187
PF01797	+	Transposase IS200 like	0.184
PF00821	+	Phosphoenolpyruvate carboxykinase	0.176
PF13826	+	Domain of unknown function (DUF4188)	0.161
PF07508	-	Recombinase	0.153
PF13539	+	D-alanyl-D-alanine carboxypeptidase	0.153
PF05448	+	Acetyl xylan esterase (AXE1)	0.149
PF11734	-	TilS substrate C-terminal domain	0.146
PF08031	+	Berberine and berberine like	0.140
PF02633	+	Creatinine amidohydrolase	0.133
PF01930	+	Domain of unknown function DUF83	0.131
PF08244	-	Glycosyl hydrolases family 32 C terminal	0.126
PF02744	+	Galactose-1-phosphate uridyl transferase, C-terminal domain	0.121
PF10593	+	Z1 domain	0.119
PF03441	+	FAD binding domain of DNA photolyase	0.113
PF00875	+	DNA photolyase	0.113
PF07669	-	Eco57I restriction-modification methylase	0.110
PF13452	+	N-terminal half of MaoC dehydratase	0.096
PF13484	-	4Fe-4S double cluster binding domain	0.091
PF13518	-	Helix-turn-helix domain	0.083
PF05164	-	Cell division protein ZapA	0.075
PF00899	-	ThiF family	0.074
PF07498	-	Rho termination factor, N-terminal domain	0.071
PF14690	+	zinc-finger of transposase IS204/IS1001/IS1096/IS1165	0.064
PF12161	+	HsdM N-terminal domain	0.064
PF13348	-	Tyrosine phosphatase family C-terminal region	0.060
PF13683	-	Integrase core domain	0.059
PF02498	-	BRO family, N-terminal domain	0.053
PF01458	-	Uncharacterized protein family (UPF0051)	0.049
PF06969	-	HemN C-terminal domain	0.047
PF01575	+	MaoC like domain	0.046
PF13309	+	HTH domain	0.041
PF08348	+	YheO-like PAS domain	0.041
PF13347	-	MFS/sugar transport protein	0.034
PF02086	+	D12 class N6 adenine-specific DNA methyltransferase	0.034
PF02926	-	THUMP domain	0.030
PF02475	-	Met-10+ like-protein	0.022
PF01841	-	Transglutaminase-like superfamily	0.019
PF03264	+	NapC/NirT cytochrome c family, N-terminal region	0.018
PF01935	-	Domain of unknown function DUF87	0.017
PF01402	+	Ribbon-helix-helix protein, copG family	0.012
PF06146	-	Phosphate-starvation-inducible E	0.005
PF13700	-	Domain of unknown function (DUF4158)	0.002
PF04290	+	Tripartite ATP-independent periplasmic transporters, DctQ component	0.001
PF01040	-	UbiA prenyltransferase family	-0.005
PF00925	-	GTP cyclohydrolase II	-0.007
PF14635	-	Helix-hairpin-helix motif	-0.008

PF00932	+	Lamin Tail Domain	-0.015
PF02335	+	Cytochrome c552	-0.017
PF04892	-	VanZ like family	-0.024
PF10412	-	Type IV secretion-system coupling protein DNA-binding domain	-0.036
PF13744	-	Helix-turn-helix domain	-0.041
PF13417	+	Glutathione S-transferase, N-terminal domain	-0.041
PF00071	+	Ras family	-0.042
PF04230	-	Polysaccharide pyruvyl transferase	-0.045
PF12568	-	Acetyltransferase (GNAT) domain	-0.046
PF00733	-	Asparagine synthase	-0.054
PF00561	-	alpha/beta hydrolase fold	-0.055
PF00999	-	Sodium/hydrogen exchanger family	-0.064
PF10881	-	Protein of unknown function (DUF2726)	-0.088
PF04383	-	KilA-N domain	-0.088
PF13596	-	PAS domain	-0.088
PF02590	-	Predicted SPOUT methyltransferase	-0.089
PF01112	-	Asparaginase	-0.094
PF13950	-	UDP-glucose 4-epimerase C-term subunit	-0.104
PF04488	-	Glycosyltransferase sugar-binding region containing DXD motif	-0.112
PF05704	-	Capsular polysaccharide synthesis protein	-0.112
PF03275	-	UDP-galactopyranose mutase	-0.127
PF13409	-	Glutathione S-transferase, N-terminal domain	-0.148
PF02604	-	Antitoxin Phd_YefM, type II toxin-antitoxin system	-0.161
PF01427	-	D-ala-D-ala dipeptidase	-0.163
PF13007	-	Transposase C of IS166 homeodomain	-0.166
PF12399	-	Branched-chain amino acid ATP-binding cassette transporter	-0.192
PF08238	-	Sel1 repeat	-0.221
PF14821	-	Threonine synthase N terminus	-0.227
PF04011	-	LemA family	-0.229

Phenotype: Coagulase production (Enzyme), Predictor: phypat

Pfam	class	Pfam_desc	cor
PF04203	+	Sortase family	0.805
PF01424	+	R3H domain	0.757
PF13083	+	KH domain	0.664
PF01883	+	Domain of unknown function DUF59	0.462
PF05154	+	TM2 domain	0.377
PF08478	+	POTRA domain, FtsQ-type	0.312
PF01636	+	Phosphotransferase enzyme family	0.302
PF02742	+	Iron dependent repressor, metal binding and dimerisation domain	0.294
PF13014	+	KH domain	0.262
PF00857	+	Isochorismatase family	0.218
PF02922	+	Carbohydrate-binding module 48 (Isoamylase N-terminal domain)	0.206
PF01842	+	ACT domain	0.194
PF07085	+	DRTGG domain	0.159
PF00923	+	Transaldolase	0.042
PF06969	+	HemN C-terminal domain	0.000
PF12146	+	Putative lysophospholipase	0.000

PF06559	+	2'-deoxycytidine 5'-triphosphate deaminase (DCD)	-0.056
PF02311	-	AraC-like ligand binding domain	-0.224
PF13185	-	GAF domain	-0.315
PF12682	-	Flavodoxin	-0.327
PF03169	-	OPT oligopeptide transporter protein	-0.359
PF13704	-	Glycosyl transferase family 2	-0.382
PF01051	-	Initiator Replication protein	-0.423
PF02547	-	Queuosine biosynthesis protein	-0.460
PF00325	-	Bacterial regulatory proteins, crp family	-0.491
PF01713	-	Smr domain	-0.508
PF02574	-	Homocysteine S-methyltransferase	-0.610
PF07732	-	Multicopper oxidase	-0.611
PF00697	-	N-(5'phosphoribosyl)anthranilate (PRA) isomerase	-0.635
PF13580	-	SIS domain	-0.637
PF13375	-	RnfC Barrel sandwich hybrid domain	-0.689
PF06508	-	Queuosine biosynthesis protein QueC	-0.692
PF02310	-	B12 binding domain	-0.709
PF12837	-	4Fe-4S binding domain	-0.709
PF02592	-	Uncharacterized ACR, YhhQ family COG1738	-0.719
PF00022	-	Actin	-0.724
PF04060	-	Putative Fe-S cluster	-0.744
PF01957	-	NfeD-like C-terminal, partner-binding	-0.757
PF06415	-	BPG-independent PGAM N-terminus (iPGM_N)	-0.784
PF04324	-	BFD-like [2Fe-2S] binding domain	-0.784
PF03116	-	NQR2, RnfD, RnfE family	-0.795
PF05683	-	Fumarase C-terminus	-0.827
PF05681	-	Fumarate hydratase (Fumerase)	-0.827
PF01075	-	Glycosyltransferase family 9 (heptosyltransferase)	-0.866

Phenotype: Coagulase production (Enzyme), Predictor: phypat+PGL

Pfam	class	Pfam_desc	cor
PF08353	+	Domain of unknown function (DUF1727)	0.805
PF02632	+	BioY family	0.719
PF08455	+	Bacterial SNF2 helicase associated	0.692
PF11772	+	DNA-directed RNA polymerase subunit beta	0.663
PF06018	+	CodY GAF-like domain	0.615
PF02797	+	Chalcone and stilbene synthases, C-terminal domain	0.612
PF08903	+	Domain of unknown function (DUF1846)	0.598
PF03051	+	Peptidase C1-like family	0.588
PF12368	+	Protein of unknown function (DUF3650)	0.587
PF06912	+	Protein of unknown function (DUF1275)	0.551
PF12732	+	YtxH-like protein	0.549
PF01883	+	Domain of unknown function DUF59	0.462
PF13305	+	WHG domain	0.408
PF12911	+	N-terminal TM domain of oligopeptide transport permease C	0.395
PF05154	+	TM2 domain	0.377
PF07536	+	HWE histidine kinase	0.377
PF07568	+	Histidine kinase	0.377

PF04020	+	Membrane protein of unknown function	0.377
PF13344	+	Haloacid dehalogenase-like hydrolase	0.369
PF00496	+	Bacterial extracellular solute-binding proteins, family 5 Middle	0.321
PF00232	+	Glycosyl hydrolase family 1	0.293
PF13244	+	Domain of unknown function (DUF4040)	0.291
PF04039	+	Domain related to MnhB subunit of Na+/H+ antiporter	0.291
PF13014	+	KH domain	0.262
PF13485	+	Peptidase MA superfamily	0.182
PF03725	+	3' exoribonuclease family, domain 2	0.167
PF03726	+	Polyribonucleotide nucleotidyltransferase, RNA binding domain	0.167
PF01138	+	3' exoribonuclease family, domain 1	0.167
PF11138	+	Protein of unknown function (DUF2911)	0.142
PF14693	+	Ribosomal protein TL5, C-terminal domain	0.099
PF01223	-	DNA/RNA non-specific endonuclease	0.033
PF09527	+	Putative F0F1-ATPase subunit (ATPase_gene1)	-0.031
PF03432	-	Relaxase/Mobilisation nuclease domain	-0.055
PF05708	-	Orthopoxvirus protein of unknown function (DUF830)	-0.111
PF13612	-	Transposase DDE domain	-0.225
PF04464	-	CDP-Glycerol:Poly(glycerophosphate) glycerophosphotransferase	-0.225
PF04909	-	Amidohydrolase	-0.243
PF01235	-	Sodium:alanine symporter family	-0.295
PF02627	-	Carboxymuconolactone decarboxylase family	-0.311
PF11870	-	Domain of unknown function (DUF3390)	-0.312
PF03169	-	OPT oligopeptide transporter protein	-0.359
PF13704	-	Glycosyl transferase family 2	-0.382
PF01638	-	HxlR-like helix-turn-helix	-0.401
PF01051	-	Initiator Replication protein	-0.423
PF02366	-	Dolichyl-phosphate-mannose-protein mannosyltransferase	-0.461
PF01855	-	Pyruvate flavodoxin/ferredoxin oxidoreductase, thiamine diP-bdg	-0.465
PF04284	-	Protein of unknown function (DUF441)	-0.481
PF13231	-	Dolichyl-phosphate-mannose-protein mannosyltransferase	-0.501
PF12974	-	ABC transporter, phosphonate, periplasmic substrate-binding protein	-0.523
PF07660	-	Secretin and TonB N terminus short domain	-0.526
PF03992	-	Antibiotic biosynthesis monooxygenase	-0.531
PF02445	-	Quinolinate synthetase A protein	-0.569
PF02086	-	D12 class N6 adenine-specific DNA methyltransferase	-0.578
PF02574	-	Homocysteine S-methyltransferase	-0.610
PF00301	-	Rubredoxin	-0.611
PF00697	-	N-(5'phosphoribosyl)anthranilate (PRA) isomerase	-0.635
PF13580	-	SIS domain	-0.637
PF02554	-	Carbon starvation protein CstA	-0.647
PF14697	-	4Fe-4S dicluster domain	-0.649
PF04333	-	VacJ like lipoprotein	-0.685
PF13375	-	RnfC Barrel sandwich hybrid domain	-0.689
PF00885	-	6,7-dimethyl-8-ribityllumazine synthase	-0.713
PF00677	-	Lumazine binding domain	-0.713
PF11638	-	DnaA N-terminal domain	-0.720
PF03572	-	Peptidase family S41	-0.723

PF08645	-	Polynucleotide kinase 3 phosphatase	-0.744
PF01957	-	NfeD-like C-terminal, partner-binding	-0.757
PF04324	-	BFD-like [2Fe-2S] binding domain	-0.784
PF06835	-	Lipopolysaccharide-assembly, LptC-related	-0.795
PF05683	-	Fumarase C-terminus	-0.827

Phenotype: Lipase (Enzyme), Predictor: phypat

Pfam	class	Pfam_desc	cor
PF14241	+	Domain of unknown function (DUF4341)	0.734
PF04209	+	homogentisate 1,2-dioxygenase	0.734
PF14696	+	Hydroxyphenylpyruvate dioxygenase, HPPD, N-terminal	0.711
PF05724	+	Thiopurine S-methyltransferase (TPMT)	0.697
PF04116	+	Fatty acid hydroxylase superfamily	0.684
PF04280	+	Tim44-like domain	0.636
PF06041	+	Bacterial protein of unknown function (DUF924)	0.627
PF07103	+	Protein of unknown function (DUF1365)	0.622
PF13745	+	HxxPF-repeated domain	0.621
PF03171	+	2OG-Fe(II) oxygenase superfamily	0.592
PF04191	+	Phospholipid methyltransferase	0.556
PF09413	+	Domain of unknown function (DUF2007)	0.542
PF08808	+	RES domain	0.535
PF11744	+	Aluminium activated malate transporter	0.528
PF06821	+	Serine hydrolase	0.517
PF10014	+	2OG-Fe dioxygenase	0.504
PF08449	+	UAA transporter family	0.503
PF03441	+	FAD binding domain of DNA photolyase	0.490
PF04072	+	Leucine carboxyl methyltransferase	0.478
PF03583	+	Secretory lipase	0.460
PF09588	+	YqaJ-like viral recombinase domain	0.433
PF00668	+	Condensation domain	0.432
PF11870	+	Domain of unknown function (DUF3390)	0.432
PF06056	+	Putative ATPase subunit of terminase (gpP-like)	0.380
PF00782	+	Dual specificity phosphatase, catalytic domain	0.374
PF00959	+	Phage lysozyme	0.318
PF13454	+	FAD-NAD(P)-binding	0.277
PF03174	+	Chitobiase/beta-hexosaminidase C-terminal domain	0.263
PF04586	+	Caudovirus prohead protease	0.223
PF01050	-	Mannose-6-phosphate isomerase	0.175
PF02541	-	Ppx/GppA phosphatase family	0.007
PF00627	-	UBA/TS-N domain	0.005
PF00291	-	Pyridoxal-phosphate dependent enzyme	-0.021
PF03807	-	NADP oxidoreductase coenzyme F420-dependent	-0.021
PF11794	-	4-hydroxyphenylacetate 3-hydroxylase N terminal	-0.036
PF03241	-	4-hydroxyphenylacetate 3-hydroxylase C terminal	-0.036
PF13189	-	Cytidylate kinase-like family	-0.039
PF13673	-	Acetyltransferase (GNAT) domain	-0.055
PF01661	-	Macro domain	-0.068
PF00465	-	Iron-containing alcohol dehydrogenase	-0.079

PF14821	-	Threonine synthase N terminus	-0.105
PF04393	-	Protein of unknown function (DUF535)	-0.150
PF07179	-	SseB protein N-terminal domain	-0.160
PF02452	-	PemK-like protein	-0.173
PF04296	-	Protein of unknown function (DUF448)	-0.195
PF06769	-	Plasmid encoded toxin Txe	-0.197
PF04760	-	Translation initiation factor IF-2, N-terminal region	-0.200
PF03288	-	Poxvirus D5 protein-like	-0.200
PF02677	-	Uncharacterized BCR, COG1636	-0.214
PF02742	-	Iron dependent repressor, metal binding and dimerisation domain	-0.227
PF12822	-	Protein of unknown function (DUF3816)	-0.235
PF13749	-	ATP-dependent DNA helicase recG C-terminal	-0.245
PF02361	-	Cobalt transport protein	-0.249
PF03786	-	D-mannonate dehydratase (UxuA)	-0.263
PF00692	-	dUTPase	-0.291
PF01867	-	CRISPR associated protein Cas1	-0.295
PF13173	-	AAA domain	-0.352
PF00703	-	Glycosyl hydrolases family 2	-0.359
PF02929	-	Beta galactosidase small chain	-0.381
PF02837	-	Glycosyl hydrolases family 2, sugar binding domain	-0.384

Phenotype: Lipase (Enzyme), Predictor: phypat+PGL

Pfam	class	Pfam_desc	cor
PF14241	+	Domain of unknown function (DUF4341)	0.734
PF05724	+	Thiopurine S-methyltransferase (TPMT)	0.697
PF13661	+	2OG-Fe(II) oxygenase superfamily	0.665
PF04377	+	Arginine-tRNA-protein transferase, C terminus	0.636
PF04376	+	Arginine-tRNA-protein transferase, N terminus	0.636
PF12973	+	ChrR Cupin-like domain	0.627
PF13745	+	HxxPF-repeated domain	0.621
PF00850	+	Histone deacetylase domain	0.572
PF04191	+	Phospholipid methyltransferase	0.556
PF09413	+	Domain of unknown function (DUF2007)	0.542
PF03413	+	Peptidase propeptide and YPEB domain	0.540
PF11744	+	Aluminium activated malate transporter	0.528
PF09917	+	Uncharacterized protein conserved in bacteria (DUF2147)	0.504
PF08449	+	UAA transporter family	0.503
PF05922	+	Peptidase inhibitor I9	0.497
PF04072	+	Leucine carboxyl methyltransferase	0.478
PF03583	+	Secretory lipase	0.460
PF04214	+	Protein of unknown function, DUF	0.453
PF12787	+	EcsC protein family	0.453
PF00080	+	Copper/zinc superoxide dismutase (SODC)	0.439
PF00668	+	Condensation domain	0.432
PF02805	+	Metal binding domain of Ada	0.424
PF06056	+	Putative ATPase subunit of terminase (gpP-like)	0.380
PF07287	+	Protein of unknown function (DUF1446)	0.369
PF11391	+	Protein of unknown function (DUF2798)	0.369

PF10719	+	Late competence development protein ComFB	0.363
PF02607	+	B12 binding domain	0.354
PF02965	+	Vitamin B12 dependent methionine synthase, activation domain	0.354
PF11140	+	Protein of unknown function (DUF2913)	0.349
PF12349	+	Sterol-sensing domain of SREBP cleavage-activation	0.329
PF00959	+	Phage lysozyme	0.318
PF07726	+	ATPase family associated with various cellular activities (AAA)	0.300
PF06213	+	Cobalamin biosynthesis protein CobT	0.291
PF12106	+	Colicin C terminal ribonuclease domain	0.284
PF13743	+	Thioredoxin	0.279
PF13454	+	FAD-NAD(P)-binding	0.277
PF00704	+	Glycosyl hydrolases family 18	0.264
PF05048	-	Periplasmic copper-binding protein (NosD)	0.252
PF05962	+	HutD	0.251
PF04892	+	VanZ like family	0.249
PF12870	+	Lumazine-binding domain	0.245
PF08840	+	BAAT / Acyl-CoA thioester hydrolase C terminal	0.222
PF14534	+	Domain of unknown function (DUF4440)	0.210
PF11480	+	Colicin-E5 Imm protein	0.200
PF01566	+	Natural resistance-associated macrophage protein	0.178
PF02016	-	LD-carboxypeptidase	0.154
PF01206	-	Sulfurtransferase TusA	0.150
PF06857	+	Malonate decarboxylase delta subunit (MdcD)	0.144
PF12834	+	Phage integrase, N-terminal	0.138
PF08020	+	Protein of unknown function (DUF1706)	0.133
PF03328	-	HpcH/HpaI aldolase/citrate lyase family	0.090
PF01925	-	Sulfite exporter TauE/SafE	0.079
PF14284	+	PcfJ-like protein	0.077
PF11299	+	Protein of unknown function (DUF3100)	0.077
PF10551	-	MULE transposase domain	0.068
PF04261	-	Dyp-type peroxidase family	0.060
PF13495	-	Phage integrase, N-terminal SAM-like domain	0.058
PF14701	+	glucanotransferase domain of human glycogen debranching enzyme	0.057
PF13682	+	Chemoreceptor zinc-binding domain	0.053
PF08485	-	Polysaccharide biosynthesis protein C-terminal	0.031
PF01298	+	Transferrin binding protein-like solute binding protein	0.021
PF13936	-	Helix-turn-helix domain	-0.011
PF06123	-	Inner membrane protein CreD	-0.012
PF01314	+	Aldehyde ferredoxin oxidoreductase, domains 2 & 3	-0.039
PF01637	-	Archaeal ATPase	-0.059
PF03773	-	Predicted permease	-0.060
PF07751	-	Abi-like protein	-0.079
PF09848	-	Uncharacterized conserved protein (DUF2075)	-0.101
PF13248	-	zinc-ribbon domain	-0.122
PF12738	-	twin BRCT domain	-0.124
PF01427	-	D-ala-D-ala dipeptidase	-0.135
PF02661	-	Fic/DOC family	-0.147
PF07179	-	SseB protein N-terminal domain	-0.160

PF05336	-	Domain of unknown function (DUF718)	-0.160
PF01487	-	Type I 3-dehydroquinase	-0.170
PF13597	-	Anaerobic ribonucleoside-triphosphate reductase	-0.184
PF06769	-	Plasmid encoded toxin Txe	-0.197
PF03881	-	Fructosamine kinase	-0.236
PF07944	-	Putative glycosyl hydrolase of unknown function (DUF1680)	-0.255
PF02614	-	Glucuronate isomerase	-0.263
PF10371	-	Domain of unknown function	-0.267
PF13173	-	AAA domain	-0.352

Phenotype: Nitrate to nitrite (Enzyme), Predictor: phypat

Pfam	class	Pfam_desc	cor
PF02613	+	Nitrate reductase delta subunit	0.820
PF13247	+	4Fe-4S dicluster domain	0.739
PF03459	+	TOBE domain	0.705
PF14711	+	Respiratory nitrate reductase beta C-terminal	0.693
PF02665	+	Nitrate reductase gamma subunit	0.688
PF06463	+	Molybdenum Cofactor Synthesis C	0.676
PF04879	+	Molybdopterin oxidoreductase Fe4S4 domain	0.660
PF00174	+	Oxidoreductase molybdopterin binding domain	0.659
PF03475	+	3-alpha domain	0.650
PF01389	+	OmpA-like transmembrane domain	0.614
PF01583	+	Adenylylsulphate kinase	0.576
PF03892	+	Nitrate reductase cytochrome c-type subunit (NapB)	0.576
PF04995	+	Heme exporter protein D (CcmD)	0.575
PF03927	+	NapD protein	0.559
PF00227	+	Proteasome subunit	0.541
PF09107	+	Elongation factor SelB, winged helix	0.530
PF03824	+	High-affinity nickel-transport protein	0.526
PF03605	+	Anaerobic c4-dicarboxylate membrane transporter	0.514
PF06779	+	Protein of unknown function (DUF1228)	0.502
PF01564	+	Spermine/spermidine synthase	0.465
PF00975	+	Thioesterase domain	0.463
PF00668	+	Condensation domain	0.442
PF13521	+	AAA domain	0.427
PF03788	+	LrgA family	0.415
PF12729	+	Four helix bundle sensory module for signal transduction	0.395
PF01730	+	UreF	0.370
PF04261	+	Dyp-type peroxidase family	0.365
PF03814	+	Potassium-transporting ATPase A subunit	0.331
PF06796	+	Periplasmic nitrate reductase protein NapE	0.308
PF13726	+	Na+-H+ antiporter family	0.304
PF08239	+	Bacterial SH3 domain	0.301
PF00282	+	Pyridoxal-dependent decarboxylase conserved domain	0.293
PF04860	+	Phage portal protein	0.274
PF01865	+	Protein of unknown function DUF47	0.213
PF14501	+	GHKL domain	0.168
PF01903	+	CbiX	0.128

PF02515	-	CoA-transferase family III	0.119
PF04120	-	Low affinity iron permease	0.104
PF13905	-	Thioredoxin-like	0.096
PF13518	-	Helix-turn-helix domain	0.087
PF13565	-	Homeodomain-like domain	0.077
PF13683	-	Integrase core domain	0.048
PF10410	-	DnaB-helicase binding domain of primase	0.043
PF03315	-	Serine dehydratase beta chain	0.031
PF03313	-	Serine dehydratase alpha chain	0.031
PF14535	-	AMP-binding enzyme C-terminal domain	0.024
PF09445	-	RNA cap guanine-N2 methyltransferase	0.016
PF03006	-	Haemolysin-III related	0.012
PF15611	-	EH_Signature domain	0.003
PF00766	-	Electron transfer flavoprotein FAD-binding domain	-0.000
PF12169	-	DNA polymerase III subunits gamma and tau domain III	-0.004
PF01769	-	Divalent cation transporter	-0.010
PF00078	-	Reverse transcriptase (RNA-dependent DNA polymerase)	-0.022
PF04548	-	AIG1 family	-0.026
PF01223	-	DNA/RNA non-specific endonuclease	-0.034
PF08937	+	MTH538 TIR-like domain (DUF1863)	-0.041
PF02464	-	Competence-damaged protein	-0.048
PF02381	-	MraZ protein	-0.056
PF13192	-	Thioredoxin domain	-0.065
PF13542	-	Helix-turn-helix domain of transposase family ISL3	-0.072
PF04024	-	PspC domain	-0.076
PF14529	-	Endonuclease-reverse transcriptase	-0.077
PF08463	-	EcoEI R protein C-terminal	-0.084
PF13588	-	Type I restriction enzyme R protein N terminus (HSDR_N)	-0.087
PF01055	-	Glycosyl hydrolases family 31	-0.090
PF01741	-	Large-conductance mechanosensitive channel, MscL	-0.111
PF07286	-	Protein of unknown function (DUF1445)	-0.115
PF02899	-	Phage integrase, N-terminal SAM-like domain	-0.122
PF02463	-	RecF/RecN/SMC N terminal domain	-0.124
PF03193	-	Protein of unknown function, DUF258	-0.127
PF01643	-	Acyl-ACP thioesterase	-0.138
PF01321	-	Creatinase/Prolidase N-terminal domain	-0.142
PF00498	-	FHA domain	-0.146
PF00327	-	Ribosomal protein L30p/L7e	-0.149
PF01988	-	VIT family	-0.157
PF06564	-	YhjQ protein	-0.159
PF00692	-	dUTPase	-0.178
PF08889	-	WbqC-like protein family	-0.185
PF00326	-	Prolyl oligopeptidase family	-0.197
PF03060	-	Nitronate monooxygenase	-0.205
PF13601	-	Winged helix DNA-binding domain	-0.221
PF13514	-	AAA domain	-0.222
PF01458	-	Uncharacterized protein family (UPF0051)	-0.225
PF13482	-	RNase_H superfamily	-0.248

PF02517	-	CAAX protease self-immunity	-0.266
PF06470	-	SMC proteins Flexible Hinge Domain	-0.290
PF00142	-	4Fe-4S iron sulfur cluster binding proteins, NifH/frxC family	-0.304
PF02632	-	BioY family	-0.330
PF02637	-	GatB domain	-0.331
PF02934	-	GatB/GatE catalytic domain	-0.331
PF06541	-	Protein of unknown function (DUF1113)	-0.405
PF04263	-	Thiamin pyrophosphokinase, catalytic domain	-0.512
PF03793	-	PASTA domain	-0.519

Phenotype: Nitrate to nitrite (Enzyme), Predictor: phypat+PGL

Pfam	class	Pfam_desc	cor
PF02613	+	Nitrate reductase delta subunit	0.820
PF13247	+	4Fe-4S dicluster domain	0.739
PF03459	+	TOBE domain	0.705
PF02665	+	Nitrate reductase gamma subunit	0.688
PF04879	+	Molybdopterin oxidoreductase Fe4S4 domain	0.660
PF01292	+	Prokaryotic cytochrome b561	0.649
PF03892	+	Nitrate reductase cytochrome c-type subunit (NapB)	0.576
PF03927	+	NapD protein	0.559
PF01127	+	Succinate dehydrogenase/Fumarate reductase transmembrane subunit	0.558
PF03824	+	High-affinity nickel-transport protein	0.526
PF13977	+	Bacterial transcriptional repressor	0.525
PF04324	+	BFD-like [2Fe-2S] binding domain	0.523
PF03738	+	Glutathionylspermidine synthase preATP-grasp	0.514
PF13806	+	Rieske-like [2Fe-2S] domain	0.501
PF02652	+	L-lactate permease	0.484
PF04717	+	Phage-related baseplate assembly protein	0.467
PF03349	+	Outer membrane protein transport protein (OMPP1/FadL/TodX)	0.466
PF02599	+	Global regulator protein family	0.465
PF00975	+	Thioesterase domain	0.463
PF03887	+	YfbU domain	0.452
PF02962	+	5-carboxymethyl-2-hydroxymuconate isomerase	0.452
PF00873	+	AcrB/AcrD/AcrF family	0.450
PF03264	+	NapC/NirT cytochrome c family, N-terminal region	0.443
PF06276	+	Ferric iron reductase FhuF-like transporter	0.443
PF06228	+	Haem utilisation ChuX/HutX	0.432
PF04172	+	LrgB-like family	0.424
PF00875	+	DNA photolyase	0.418
PF03441	+	FAD binding domain of DNA photolyase	0.418
PF01490	+	Transmembrane amino acid transporter protein	0.396
PF12729	+	Four helix bundle sensory module for signal transduction	0.395
PF01957	+	NfeD-like C-terminal, partner-binding	0.383
PF01730	+	UreF	0.370
PF04400	+	Protein of unknown function (DUF539)	0.368
PF01904	+	Protein of unknown function DUF72	0.366
PF00850	+	Histone deacetylase domain	0.353
PF05069	+	Phage virion morphogenesis family	0.343

PF02743	+	Cache domain	0.341
PF00449	+	Urease alpha-subunit, N-terminal domain	0.330
PF01037	+	AsnC family	0.325
PF05016	+	Plasmid stabilisation system protein	0.315
PF13483	+	Beta-lactamase superfamily domain	0.294
PF00282	+	Pyridoxal-dependent decarboxylase conserved domain	0.293
PF10442	+	FIST C domain	0.276
PF04860	+	Phage portal protein	0.274
PF12679	-	ABC-2 family transporter protein	0.268
PF00563	-	EAL domain	0.265
PF14248	+	Domain of unknown function (DUF4345)	0.264
PF06821	-	Serine hydrolase	0.264
PF07836	+	DmpG-like communication domain	0.264
PF09981	+	Uncharacterized protein conserved in bacteria (DUF2218)	0.264
PF01081	-	KDPG and KHG aldolase	0.262
PF05239	+	PRC-barrel domain	0.250
PF02342	+	TerD domain	0.248
PF11162	-	Protein of unknown function (DUF2946)	0.248
PF13407	+	Periplasmic binding protein domain	0.236
PF11932	+	Protein of unknown function (DUF3450)	0.229
PF13581	-	Histidine kinase-like ATPase domain	0.227
PF00532	+	Periplasmic binding proteins and sugar binding domain of LacI family	0.225
PF04963	-	Sigma-54 factor, core binding domain	0.223
PF08028	-	Acyl-CoA dehydrogenase, C-terminal domain	0.220
PF12837	-	4Fe-4S binding domain	0.220
PF11852	+	Domain of unknown function (DUF3372)	0.215
PF13577	-	SnoaL-like domain	0.203
PF10589	-	NADH-ubiquinone oxidoreductase-F iron-sulfur binding region	0.202
PF09997	-	Predicted membrane protein (DUF2238)	0.201
PF13447	+	Seven times multi-haem cytochrome CxxCH	0.195
PF00614	+	Phospholipase D Active site motif	0.193
PF04286	+	Protein of unknown function (DUF445)	0.180
PF07484	-	Phage Tail Collar Domain	0.179
PF00016	+	Ribulose bisphosphate carboxylase large chain, catalytic domain	0.178
PF05226	+	CHASE2 domain	0.169
PF14501	+	GHKL domain	0.168
PF01161	-	Phosphatidylethanolamine-binding protein	0.164
PF00441	-	Acyl-CoA dehydrogenase, C-terminal domain	0.164
PF14532	-	Sigma-54 interaction domain	0.164
PF01925	-	Sulfite exporter TauE/SafE	0.159
PF03609	+	PTS system sorbose-specific iic component	0.155
PF01850	-	PIN domain	0.150
PF04014	-	Antidote-toxin recognition MazE	0.148
PF13576	+	Pentapeptide repeats (9 copies)	0.142
PF13567	+	Domain of unknown function (DUF4131)	0.127
PF13276	-	HTH-like domain	0.125
PF14885	+	Hypothetical glycosyl hydrolase family 15	0.122
PF04773	-	FecR protein	0.116

PF10029	-	Predicted periplasmic protein (DUF2271)	0.112
PF13007	-	Transposase C of IS166 homeodomain	0.111
PF04268	+	Sarcosine oxidase, gamma subunit family	0.110
PF03009	-	Glycerophosphoryl diester phosphodiesterase family	0.109
PF08643	-	Fungal family of unknown function (DUF1776)	0.099
PF05717	-	IS66 Orf2 like protein	0.096
PF13384	-	Homeodomain-like domain	0.095
PF04230	-	Polysaccharide pyruvyl transferase	0.094
PF00255	-	Glutathione peroxidase	0.081
PF13011	+	leucine-zipper of insertion element IS481	0.078
PF01965	-	DJ-1/PfpI family	0.075
PF02664	+	S-Ribosylhomocysteinase (LuxS)	0.074
PF12728	-	Helix-turn-helix domain	0.067
PF00301	-	Rubredoxin	0.058
PF13768	-	von Willebrand factor type A domain	0.044
PF04471	-	Restriction endonuclease	0.033
PF03313	-	Serine dehydratase alpha chain	0.031
PF03315	-	Serine dehydratase beta chain	0.031
PF13087	-	AAA domain	0.031
PF06314	+	Acetoacetate decarboxylase (ADC)	0.029
PF02016	-	LD-carboxypeptidase	0.026
PF13575	+	Domain of unknown function (DUF4135)	0.026
PF02535	-	ZIP Zinc transporter	0.025
PF14535	-	AMP-binding enzyme C-terminal domain	0.024
PF13470	-	PIN domain	0.019
PF03306	+	Alpha-acetolactate decarboxylase	0.017
PF09445	-	RNA cap guanine-N2 methyltransferase	0.016
PF03006	-	Haemolysin-III related	0.012
PF01888	-	CbiD	0.007
PF08388	-	Group II intron, maturase-specific domain	0.005
PF01012	-	Electron transfer flavoprotein domain	0.002
PF03747	-	ADP-ribosylglycohydrolase	0.001
PF00766	-	Electron transfer flavoprotein FAD-binding domain	-0.000
PF13086	-	AAA domain	-0.014
PF13574	-	Metallo-peptidase family M12B Reprolysin-like	-0.020
PF13246	+	Putative hydrolase of sodium-potassium ATPase alpha subunit	-0.022
PF00128	-	Alpha amylase, catalytic domain	-0.024
PF02562	-	PhoH-like protein	-0.031
PF05057	-	Putative serine esterase (DUF676)	-0.036
PF13596	-	PAS domain	-0.038
PF08937	+	MTH538 TIR-like domain (DUF1863)	-0.041
PF07859	-	alpha/beta hydrolase fold	-0.042
PF10397	-	Adenylosuccinate lyase C-terminus	-0.042
PF02498	-	BRO family, N-terminal domain	-0.043
PF02700	+	Phosphoribosylformylglycinamidine (FGAM) synthase	-0.055
PF00239	-	Resolvase, N terminal domain	-0.063
PF07726	+	ATPase family associated with various cellular activities (AAA)	-0.068
PF08463	-	EcoEI R protein C-terminal	-0.084

PF00150	-	Cellulase (glycosyl hydrolase family 5)	-0.091
PF04221	-	RelB antitoxin	-0.110
PF06250	-	Protein of unknown function (DUF1016)	-0.116
PF13707	+	RloB-like protein	-0.125
PF00069	-	Protein kinase domain	-0.132
PF01174	-	SNO glutamine amidotransferase family	-0.133
PF07476	-	Methylaspartate ammonia-lyase C-terminus	-0.134
PF01321	-	Creatinase/Prolidase N-terminal domain	-0.142
PF02608	-	Basic membrane protein	-0.150
PF01988	-	VIT family	-0.157
PF00082	-	Subtilase family	-0.181
PF03703	-	Bacterial PH domain	-0.191
PF00326	-	Prolyl oligopeptidase family	-0.197
PF09424	-	Yqey-like protein	-0.211
PF02361	-	Cobalt transport protein	-0.214
PF01458	-	Uncharacterized protein family (UPF0051)	-0.225
PF04020	-	Membrane protein of unknown function	-0.249
PF02517	-	CAAX protease self-immunity	-0.266
PF13495	-	Phage integrase, N-terminal SAM-like domain	-0.290
PF00142	-	4Fe-4S iron sulfur cluster binding proteins, NifH/frxC family	-0.304
PF02632	-	BioY family	-0.330
PF05167	+	Uncharacterised ACR (DUF711)	-0.373

Phenotype: Nitrite to gas (Enzyme), Predictor: phypat

Pfam	class	Pfam_desc	cor
PF09832	+	Uncharacterized protein conserved in bacteria (DUF2059)	0.470
PF06627	+	Protein of unknown function (DUF1153)	0.470
PF05573	+	NosL	0.469
PF11953	+	Domain of unknown function (DUF3470)	0.424
PF04273	+	Putative phosphatase (DUF442)	0.403
PF13429	+	Tetratricopeptide repeat	0.374
PF15579	+	Immunity protein 32	0.368
PF14451	+	Mut7-C ubiquitin	0.333
PF04392	+	ABC transporter substrate binding protein	0.327
PF07120	+	Protein of unknown function (DUF1376)	0.323
PF04214	+	Protein of unknown function, DUF	0.322
PF11373	+	Protein of unknown function (DUF3175)	0.297
PF04011	+	LemA family	0.296
PF03797	+	Autotransporter beta-domain	0.288
PF13817	+	IS66 C-terminal element	0.281
PF05717	+	IS66 Orf2 like protein	0.281
PF13007	+	Transposase C of IS166 homeodomain	0.281
PF05707	+	Zonular occludens toxin (Zot)	0.255
PF04945	+	YHS domain	0.254
PF14595	+	Thioredoxin	0.233
PF05728	+	Uncharacterised protein family (UPF0227)	0.222
PF04972	+	BON domain	0.214
PF03918	+	Cytochrome C biogenesis protein	0.165

PF03379	+	CcmB protein	0.165
PF03100	+	CcmE	0.165
PF05930	+	Prophage CP4-57 regulatory protein (AlpA)	0.137
PF03681	+	Uncharacterised protein family (UPF0150)	0.108
PF02475	+	Met-10+ like-protein	0.081
PF02371	+	Transposase IS116/IS110/IS902 family	0.071
PF00145	+	C-5 cytosine-specific DNA methylase	0.043
PF12680	-	SnoaL-like domain	-0.078
PF12973	-	ChrR Cupin-like domain	-0.111
PF07228	-	Stage II sporulation protein E (SpoIIE)	-0.119
PF07103	-	Protein of unknown function (DUF1365)	-0.120
PF13813	-	Membrane bound O-acyl transferase family	-0.152
PF06293	-	Lipopolysaccharide kinase (Kdo/WaaP) family	-0.174
PF00195	-	Chalcone and stilbene synthases, N-terminal domain	-0.208
PF02275	-	Linear amide C-N hydrolases, choloylglycine hydrolase family	-0.222
PF04794	-	YdjC-like protein	-0.236
PF12792	-	CSS motif domain associated with EAL	-0.252
PF01306	-	LacY proton/sugar symporter	-0.260
PF01590	-	GAF domain	-0.263
PF05893	-	Acyl-CoA reductase (LuxC)	-0.263
PF02452	-	PemK-like protein	-0.269
PF04960	-	Glutaminase	-0.282
PF06414	-	Zeta toxin	-0.287
PF09330	-	D-lactate dehydrogenase, membrane binding	-0.288
PF00975	-	Thioesterase domain	-0.304
PF00654	-	Voltage gated chloride channel	-0.350
PF12833	-	Helix-turn-helix domain	-0.368
PF08544	-	GHMP kinases C terminal	-0.373
PF03848	-	Tellurite resistance protein TehB	-0.399
PF01047	-	MarR family	-0.405
PF04235	-	Protein of unknown function (DUF418)	-0.411
PF05116	-	Sucrose-6F-phosphate phosphohydrolase	-0.418
PF00393	-	6-phosphogluconate dehydrogenase, C-terminal domain	-0.426
PF10111	-	Glycosyltransferase like family 2	-0.434
PF00265	-	Thymidine kinase	-0.459
PF11799	-	impB/mucB/samB family C-terminal domain	-0.470
PF00480	-	ROK family	-0.487
PF01316	-	Arginine repressor, DNA binding domain	-0.502
PF00817	-	impB/mucB/samB family	-0.512

Phenotype: Nitrite to gas (Enzyme), Predictor: phypat+PGL

Pfam	class	Pfam_desc	cor
PF05573	+	NosL	0.469
PF04273	+	Putative phosphatase (DUF442)	0.403
PF02239	+	Cytochrome D1 heme domain	0.401
PF13429	+	Tetratricopeptide repeat	0.374
PF10294	+	Putative methyltransferase	0.361
PF02974	+	Protease inhibitor Inh	0.336

PF14451	+	Mut7-C ubiquitin	0.333
PF00274	+	Fructose-bisphosphate aldolase class-I	0.333
PF04392	+	ABC transporter substrate binding protein	0.327
PF07120	+	Protein of unknown function (DUF1376)	0.323
PF04214	+	Protein of unknown function, DUF	0.322
PF03797	+	Autotransporter beta-domain	0.288
PF12872	+	OST-HTH/LOTUS domain	0.282
PF10017	+	Histidine-specific methyltransferase, SAM-dependent	0.282
PF01960	+	ArgJ family	0.279
PF15569	+	Immunity protein 21	0.262
PF05707	+	Zonular occludens toxin (Zot)	0.255
PF04945	+	YHS domain	0.254
PF11860	+	Protein of unknown function (DUF3380)	0.239
PF14595	+	Thioredoxin	0.233
PF10589	+	NADH-ubiquinone oxidoreductase-F iron-sulfur binding region	0.230
PF07015	+	VirC1 protein	0.217
PF03413	+	Peptidase propeptide and YPEB domain	0.213
PF03713	+	Domain of unknown function (DUF305)	0.175
PF07536	+	HWE histidine kinase	0.169
PF03100	+	CcmE	0.165
PF03918	+	Cytochrome C biogenesis protein	0.165
PF11937	+	Protein of unknown function (DUF3455)	0.164
PF09834	+	Predicted membrane protein (DUF2061)	0.154
PF14362	+	Domain of unknown function (DUF4407)	0.143
PF07505	+	Phage protein Gp37/Gp68	0.141
PF05930	+	Prophage CP4-57 regulatory protein (AlpA)	0.137
PF05171	+	Haemin-degrading HemS.ChuX domain	0.110
PF00959	+	Phage lysozyme	0.094
PF00211	+	Adenylate and Guanylate cyclase catalytic domain	0.092
PF04717	+	Phage-related baseplate assembly protein	0.081
PF02475	+	Met-10+ like-protein	0.081
PF03534	+	Salmonella virulence plasmid 65kDa B protein	0.081
PF07486	+	Cell Wall Hydrolase	0.078
PF13528	+	Glycosyl transferase family 1	0.078
PF00982	+	Glycosyltransferase family 20	0.068
PF13360	+	PQQ-like domain	0.053
PF07695	-	7TM diverse intracellular signalling	0.030
PF14306	+	PUA-like domain	0.027
PF05896	+	Na(+)-translocating NADH-quinone reductase subunit A (NQRA)	0.018
PF11127	+	Protein of unknown function (DUF2892)	0.014
PF13707	+	RloB-like protein	0.008
PF05840	+	Bacteriophage replication gene A protein (GPA)	-0.019
PF12729	-	Four helix bundle sensory module for signal transduction	-0.029
PF07317	-	Flagellar regulator YcgR	-0.081
PF04014	-	Antidote-toxin recognition MazE	-0.085
PF05772	-	NinB protein	-0.092
PF12639	-	DNase/tRNase domain of colicin-like bacteriocin	-0.092
PF11086	-	Protein of unknown function (DUF2878)	-0.100

PF03070	-	TENA/THI-4/PQQC family	-0.104
PF02794	-	RTX toxin acyltransferase family	-0.110
PF08386	-	TAP-like protein	-0.111
PF04389	-	Peptidase family M28	-0.119
PF07103	-	Protein of unknown function (DUF1365)	-0.120
PF01219	-	Prokaryotic diacylglycerol kinase	-0.121
PF13606	-	Ankyrin repeat	-0.129
PF04860	-	Phage portal protein	-0.130
PF02589	-	Uncharacterised ACR, YkgG family COG1556	-0.132
PF06296	-	Protein of unknown function (DUF1044)	-0.138
PF05076	-	Suppressor of fused protein (SUFU)	-0.147
PF13857	-	Ankyrin repeats (many copies)	-0.150
PF01596	+	O-methyltransferase	-0.152
PF11870	-	Domain of unknown function (DUF3390)	-0.154
PF14883	-	Hypothetical glycosyl hydrolase family 13	-0.156
PF00082	-	Subtilase family	-0.171
PF14412	-	A nuclease family of the HNH/ENDO VII superfamily with conserved AHH	-0.192
PF00690	-	Cation transporter/ATPase, N-terminus	-0.194
PF00657	-	GDSL-like Lipase/Acylhydrolase	-0.201
PF03994	-	Domain of Unknown Function (DUF350)	-0.206
PF04076	-	Bacterial OB fold (BOF) protein	-0.213
PF01501	-	Glycosyl transferase family 8	-0.251
PF07859	-	alpha/beta hydrolase fold	-0.263
PF05893	-	Acyl-CoA reductase (LuxC)	-0.263
PF01268	-	Formate--tetrahydrofolate ligase	-0.288
PF06725	-	3D domain	-0.296
PF00975	-	Thioesterase domain	-0.304
PF01263	-	Aldose 1-epimerase	-0.304
PF14529	-	Endonuclease-reverse transcriptase	-0.308
PF06628	-	Catalase-related immune-responsive	-0.322
PF00703	-	Glycosyl hydrolases family 2	-0.327
PF02887	-	Pyruvate kinase, alpha/beta domain	-0.333
PF03880	-	DbpA RNA binding domain	-0.341
PF00654	-	Voltage gated chloride channel	-0.350
PF08279	-	HTH domain	-0.368
PF00903	-	Glyoxalase/Bleomycin resistance protein/Dioxygenase superfamily	-0.368
PF01325	-	Iron dependent repressor, N-terminal DNA binding domain	-0.397
PF00393	-	6-phosphogluconate dehydrogenase, C-terminal domain	-0.426
PF10111	-	Glycosyltransferase like family 2	-0.434
PF11799	-	impB/mucB/samB family C-terminal domain	-0.470
PF01316	-	Arginine repressor, DNA binding domain	-0.502
PF01048	-	Phosphorylase superfamily	-0.502
PF00817	-	impB/mucB/samB family	-0.512

Phenotype: Pyrrolidonyl-beta-naphthylamide (Enzyme), Predictor: phypat

Pfam	class	Pfam_desc	cor
PF01470	+	Pyroglutamyl peptidase	0.693
PF06149	+	Protein of unknown function (DUF969)	0.682

PF06166	+	Protein of unknown function (DUF979)	0.682
PF09851	+	Short C-terminal domain	0.460
PF13646	+	HEAT repeats	0.399
PF13370	+	4Fe-4S single cluster domain	0.387
PF04468	+	PSP1 C-terminal conserved region	0.383
PF13459	+	4Fe-4S single cluster domain	0.364
PF06605	+	Prophage endopeptidase tail	0.359
PF07849	+	Protein of unknown function (DUF1641)	0.343
PF05063	+	MT-A70	0.343
PF02694	+	Uncharacterised BCR, YnfA/UPF0060 family	0.321
PF08401	+	Domain of unknown function (DUF1738)	0.317
PF01175	+	Urocanase	0.309
PF13468	+	Glyoxalase-like domain	0.309
PF05532	+	CsbD-like	0.274
PF13555	+	P-loop containing region of AAA domain	0.268
PF06791	+	Prophage tail length tape measure protein	0.250
PF01008	+	Initiation factor 2 subunit family	0.242
PF14502	+	Helix-turn-helix domain	0.232
PF02502	+	Ribose/Galactose Isomerase	0.214
PF06754	+	Phosphonate metabolism protein PhnG	0.213
PF01642	+	Methylmalonyl-CoA mutase	0.207
PF02239	+	Cytochrome D1 heme domain	0.192
PF13768	+	von Willebrand factor type A domain	0.187
PF13408	+	Recombinase zinc beta ribbon domain	0.184
PF01729	+	Quinolinate phosphoribosyl transferase, C-terminal domain	0.184
PF02445	+	Quinolinate synthetase A protein	0.171
PF08212	+	Lipocalin-like domain	0.166
PF14863	+	Alkyl sulfatase dimerisation	0.140
PF03572	-	Peptidase family S41	0.007
PF13746	-	4Fe-4S dicluster domain	-0.029
PF04313	-	Type I restriction enzyme R protein N terminus (HSDR_N)	-0.039
PF08003	-	Protein of unknown function (DUF1698)	-0.045
PF14355	-	Abortive infection C-terminus	-0.046
PF00999	-	Sodium/hydrogen exchanger family	-0.062
PF01946	-	Thi4 family	-0.067
PF01906	-	Putative heavy-metal-binding	-0.083
PF02895	-	Signal transducing histidine kinase, homodimeric domain	-0.091
PF03553	-	Na+/H+ antiporter family	-0.096
PF00201	-	UDP-glucoronosyl and UDP-glucosyl transferase	-0.100
PF06769	-	Plasmid encoded toxin Txe	-0.104
PF13289	-	SIR2-like domain	-0.104
PF02624	-	YcaO-like family	-0.107
PF07732	-	Multicopper oxidase	-0.113
PF01627	-	Hpt domain	-0.115
PF09084	-	NMT1/THI5 like	-0.117
PF00391	-	PEP-utilising enzyme, mobile domain	-0.132
PF02806	-	Alpha amylase, C-terminal all-beta domain	-0.152
PF01435	-	Peptidase family M48	-0.166

PF13088	-	BNR repeat-like domain	-0.168
PF05157	-	Type II secretion system (T2SS), protein E, N-terminal domain	-0.181
PF13754	-	Bacterial Ig-like domain (group 3)	-0.184
PF01863	-	Protein of unknown function DUF45	-0.188
PF13491	-	Domain of unknown function (DUF4117)	-0.192
PF08544	-	GHMP kinases C terminal	-0.192
PF07655	-	Secretin N-terminal domain	-0.194
PF09848	-	Uncharacterized conserved protein (DUF2075)	-0.196
PF00343	-	Carbohydrate phosphorylase	-0.203
PF04011	-	LemA family	-0.209
PF00782	-	Dual specificity phosphatase, catalytic domain	-0.215
PF09392	-	Type III secretion needle MxiH like	-0.224
PF00107	-	Zinc-binding dehydrogenase	-0.233
PF07201	-	HrpJ-like domain	-0.239
PF01555	-	DNA methylase	-0.243
PF14667	-	Polysaccharide biosynthesis C-terminal domain	-0.251
PF01764	-	Lipase (class 3)	-0.254
PF14821	-	Threonine synthase N terminus	-0.268
PF07510	-	Protein of unknown function (DUF1524)	-0.272
PF13367	-	Protease prsW family	-0.282
PF01728	-	FtsJ-like methyltransferase	-0.342

Phenotype: Pyrrolidonyl-beta-naphthylamide (Enzyme), Predictor: phypat+PGL

Pfam	class	Pfam_desc	cor
PF01470	+	Pyroglutamyl peptidase	0.693
PF06149	+	Protein of unknown function (DUF969)	0.682
PF06166	+	Protein of unknown function (DUF979)	0.682
PF09851	+	Short C-terminal domain	0.460
PF13370	+	4Fe-4S single cluster domain	0.387
PF13459	+	4Fe-4S single cluster domain	0.364
PF13587	+	N-terminal domain of DJ-1_PfpI family	0.361
PF06605	+	Prophage endopeptidase tail	0.359
PF02694	+	Uncharacterised BCR, YnfA/UPF0060 family	0.321
PF13444	+	Acetyltransferase (GNAT) domain	0.321
PF01175	+	Urocanase	0.309
PF09524	+	Conserved phage C-terminus (Phg_2220_C)	0.296
PF13785	+	Domain of unknown function (DUF4178)	0.291
PF10990	+	Protein of unknown function (DUF2809)	0.291
PF05610	+	Protein of unknown function (DUF779)	0.290
PF07719	+	Tetratricopeptide repeat	0.263
PF01769	+	Divalent cation transporter	0.260
PF14042	+	Domain of unknown function (DUF4247)	0.251
PF01791	+	DeoC/LacD family aldolase	0.245
PF14226	+	non-haem dioxygenase in morphine synthesis N-terminal	0.242
PF01008	+	Initiation factor 2 subunit family	0.242
PF03649	+	Uncharacterised protein family (UPF0014)	0.231
PF03729	+	Short repeat of unknown function (DUF308)	0.227
PF13205	+	Bacterial Ig-like domain	0.225

PF06754	+	Phosphonate metabolism protein PhnG	0.213
PF01095	+	Pectinesterase	0.213
PF01642	+	Methylmalonyl-CoA mutase	0.207
PF10588	+	NADH-ubiquinone oxidoreductase-G iron-sulfur binding region	0.199
PF14337	+	Domain of unknown function (DUF4393)	0.192
PF02239	+	Cytochrome D1 heme domain	0.192
PF13768	+	von Willebrand factor type A domain	0.187
PF09565	+	NgoFVII restriction endonuclease	0.177
PF02445	+	Quinolinate synthetase A protein	0.171
PF08708	+	Primase C terminal 1 (PriCT-1)	0.144
PF13380	+	CoA binding domain	0.137
PF14606	+	GDSL-like Lipase/Acylhydrolase family	0.132
PF04235	+	Protein of unknown function (DUF418)	0.130
PF02604	+	Antitoxin Phd_YefM, type II toxin-antitoxin system	0.123
PF03308	+	ArgK protein	0.117
PF05076	+	Suppressor of fused protein (SUFU)	0.116
PF05145	+	Putative ammonia monooxygenase	0.102
PF02709	+	N-terminal domain of galactosyltransferase	0.091
PF05171	+	Haemin-degrading HemS.ChuX domain	0.090
PF00011	+	Hsp20/alpha crystallin family	0.083
PF00239	-	Resolvase, N terminal domain	0.082
PF09278	-	MerR, DNA binding	0.082
PF11734	-	TilS substrate C-terminal domain	0.069
PF02810	-	SEC-C motif	0.059
PF00909	-	Ammonium Transporter Family	0.053
PF01878	-	EVE domain	0.043
PF04466	+	Phage terminase large subunit	0.032
PF04606	-	Ogr/Delta-like zinc finger	0.019
PF14265	-	Domain of unknown function (DUF4355)	0.016
PF03572	-	Peptidase family S41	0.007
PF01326	-	Pyruvate phosphate dikinase, PEP/pyruvate binding domain	-0.001
PF02781	-	Glucose-6-phosphate dehydrogenase, C-terminal domain	-0.011
PF00479	-	Glucose-6-phosphate dehydrogenase, NAD binding domain	-0.011
PF04464	-	CDP-Glycerol:Poly(glycerophosphate) glycerophosphotransferase	-0.016
PF00848	-	Ring hydroxylating alpha subunit (catalytic domain)	-0.017
PF13359	-	DDE superfamily endonuclease	-0.046
PF13020	-	Domain of unknown function (DUF3883)	-0.059
PF08386	-	TAP-like protein	-0.064
PF06271	-	RDD family	-0.069
PF01906	-	Putative heavy-metal-binding	-0.083
PF13086	-	AAA domain	-0.091
PF13855	-	Leucine rich repeat	-0.097
PF07905	-	Purine catabolism regulatory protein-like family	-0.097
PF00043	-	Glutathione S-transferase, C-terminal domain	-0.108
PF13766	-	2-enoyl-CoA Hydratase C-terminal region	-0.116
PF09084	-	NMT1/THI5 like	-0.117
PF07015	-	VirC1 protein	-0.133
PF13435	-	Cytochrome c554 and c-prime	-0.142

PF03023	-	MviN-like protein	-0.154
PF00652	-	Ricin-type beta-trefoil lectin domain	-0.160
PF13088	-	BNR repeat-like domain	-0.168
PF07158	-	Dicarboxylate carrier protein MatC N-terminus	-0.177
PF13754	-	Bacterial Ig-like domain (group 3)	-0.184
PF07655	-	Secretin N-terminal domain	-0.194
PF04011	-	LemA family	-0.209
PF01764	-	Lipase (class 3)	-0.254
PF10076	-	Uncharacterized protein conserved in bacteria (DUF2313)	-0.258
PF07510	-	Protein of unknown function (DUF1524)	-0.272

Phenotype: Bile-susceptible (Growth), Predictor: phypat

Pfam	class	Pfam_desc	cor
PF13166	+	AAA domain	0.522
PF02355	+	Protein export membrane protein	0.520
PF04324	+	BFD-like [2Fe-2S] binding domain	0.496
PF00111	+	2Fe-2S iron-sulfur cluster binding domain	0.491
PF02545	+	Maf-like protein	0.480
PF03781	+	Sulfatase-modifying factor enzyme 1	0.467
PF00493	+	MCM2/3/5 family	0.465
PF01323	+	DSBA-like thioredoxin domain	0.463
PF02146	+	Sir2 family	0.434
PF02617	+	ATP-dependent Clp protease adaptor protein ClpS	0.422
PF13378	+	Enolase C-terminal domain-like	0.418
PF00733	+	Asparagine synthase	0.418
PF08298	+	PrkA AAA domain	0.411
PF01593	+	Flavin containing amine oxidoreductase	0.406
PF13592	+	Winged helix-turn helix	0.370
PF01471	+	Putative peptidoglycan binding domain	0.329
PF13462	+	Thioredoxin	0.306
PF13679	+	Methyltransferase domain	0.289
PF12796	+	Ankyrin repeats (3 copies)	0.283
PF07833	+	Copper amine oxidase N-terminal domain	0.278
PF03600	+	Citrate transporter	0.259
PF06144	+	DNA polymerase III, delta subunit	0.247
PF01680	+	SOR/SNZ family	0.236
PF03711	+	Orn/Lys/Arg decarboxylase, C-terminal domain	0.186
PF02274	+	Amidinotransferase	0.098
PF13413	+	Helix-turn-helix domain	0.084
PF03070	+	TENA/THI-4/PQQC family	0.031
PF01930	-	Domain of unknown function DUF83	-0.030
PF08448	-	PAS fold	-0.040
PF02812	-	Glu/Leu/Phe/Val dehydrogenase, dimerisation domain	-0.198
PF01638	-	HxlR-like helix-turn-helix	-0.207
PF00282	-	Pyridoxal-dependent decarboxylase conserved domain	-0.234
PF00135	-	Carboxylesterase family	-0.234
PF02646	-	RmuC family	-0.236
PF01906	-	Putative heavy-metal-binding	-0.259

PF08757	-	CotH protein	-0.275
PF03729	-	Short repeat of unknown function (DUF308)	-0.299
PF10544	-	T5orf172 domain	-0.299
PF04024	-	PspC domain	-0.306
PF09972	-	Predicted membrane protein (DUF2207)	-0.457
PF08244	-	Glycosyl hydrolases family 32 C terminal	-0.465
PF00251	-	Glycosyl hydrolases family 32 N-terminal domain	-0.473
PF00989	-	PAS fold	-0.491
PF06800	-	Sugar transport protein	-0.499
PF06908	-	Protein of unknown function (DUF1273)	-0.528
PF02525	-	Flavodoxin-like fold	-0.540
PF08455	-	Bacterial SNF2 helicase associated	-0.551
PF01219	-	Prokaryotic diacylglycerol kinase	-0.626

Phenotype: Bile-susceptible (Growth), Predictor: phypat+PGL

Pfam	class	Pfam_desc	cor
PF13166	+	AAA domain	0.522
PF02355	+	Protein export membrane protein	0.520
PF04324	+	BFD-like [2Fe-2S] binding domain	0.496
PF10150	+	Ribonuclease E/G family	0.491
PF08669	+	Glycine cleavage T-protein C-terminal barrel domain	0.491
PF03781	+	Sulfatase-modifying factor enzyme 1	0.467
PF01323	+	DSBA-like thioredoxin domain	0.463
PF02910	+	Fumarate reductase flavoprotein C-term	0.436
PF13378	+	Enolase C-terminal domain-like	0.418
PF00733	+	Asparagine synthase	0.418
PF02900	+	Catalytic LigB subunit of aromatic ring-opening dioxygenase	0.409
PF01593	+	Flavin containing amine oxidoreductase	0.406
PF01188	+	Mandelate racemase / muconate lactonizing enzyme, C-terminal domain	0.385
PF11185	+	Protein of unknown function (DUF2971)	0.344
PF10604	+	Polyketide cyclase / dehydrase and lipid transport	0.344
PF07638	+	ECF sigma factor	0.342
PF01471	+	Putative peptidoglycan binding domain	0.329
PF14489	+	QueF-like protein	0.325
PF13263	+	PHP-associated	0.299
PF03423	+	Carbohydrate binding domain (family 25)	0.299
PF13588	+	Type I restriction enzyme R protein N terminus (HSDR_N)	0.296
PF13428	+	Tetratricopeptide repeat	0.289
PF05973	+	Phage derived protein Gp49-like (DUF891)	0.283
PF07833	+	Copper amine oxidase N-terminal domain	0.278
PF14907	+	Uncharacterised nucleotidyltransferase	0.278
PF09563	+	LlaJI restriction endonuclease	0.274
PF09707	+	CRISPR-associated protein (Cas_Cas2CT1978)	0.274
PF13855	+	Leucine rich repeat	0.271
PF01758	+	Sodium Bile acid symporter family	0.264
PF08645	+	Polynucleotide kinase 3 phosphatase	0.262
PF03600	+	Citrate transporter	0.259
PF06144	+	DNA polymerase III, delta subunit	0.247

PF13714	+	Phosphoenolpyruvate phosphomutase	0.239
PF00501	+	AMP-binding enzyme	0.223
PF01032	+	FecCD transport family	0.223
PF07683	+	Cobalamin synthesis protein cobW C-terminal domain	0.223
PF03008	+	Archaea bacterial proteins of unknown function	0.203
PF12568	+	Acetyltransferase (GNAT) domain	0.203
PF02746	+	Mandelate racemase / muconate lactonizing enzyme, N-terminal domain	0.198
PF00468	+	Ribosomal protein L34	0.197
PF01614	+	Bacterial transcriptional regulator	0.186
PF13193	+	AMP-binding enzyme C-terminal domain	0.176
PF02870	+	6-O-methylguanine DNA methyltransferase, ribonuclease-like domain	0.130
PF09439	+	Signal recognition particle receptor beta subunit	0.099
PF02274	+	Amidinotransferase	0.098
PF02624	+	YcaO-like family	0.092
PF11335	+	Protein of unknown function (DUF3137)	0.081
PF09190	+	DALR domain	0.081
PF02604	+	Antitoxin Phd_YefM, type II toxin-antitoxin system	0.063
PF13787	+	Protein of unknown function with HXXEE motif	0.056
PF03812	+	2-keto-3-deoxygluconate permease	0.015
PF07090	+	Protein of unknown function (DUF1355)	0.011
PF01914	-	MarC family integral membrane protein	0.003
PF12895	-	Anaphase-promoting complex, cyclosome, subunit 3	0.003
PF01094	+	Receptor family ligand binding region	-0.013
PF13521	+	AAA domain	-0.018
PF09674	-	Protein of unknown function (DUF2400)	-0.024
PF01262	-	Alanine dehydrogenase/PNT, C-terminal domain	-0.045
PF05107	-	Family of unknown function (DUF694)	-0.046
PF13277	-	YmdB-like protein	-0.059
PF00108	+	Thiolase, N-terminal domain	-0.060
PF13591	-	MerR HTH family regulatory protein	-0.065
PF10551	-	MULE transposase domain	-0.065
PF03734	-	L,D-transpeptidase catalytic domain	-0.120
PF03235	-	Protein of unknown function DUF262	-0.129
PF03733	-	Domain of unknown function (DUF307)	-0.146
PF13612	-	Transposase DDE domain	-0.149
PF00724	-	NADH:flavin oxidoreductase / NADH oxidase family	-0.156
PF02230	-	Phospholipase/Carboxylesterase	-0.158
PF01496	-	V-type ATPase 116kDa subunit family	-0.197
PF03814	-	Potassium-transporting ATPase A subunit	-0.202
PF01638	-	HxlR-like helix-turn-helix	-0.207
PF13509	-	S1 domain	-0.208
PF00596	-	Class II Aldolase and Adducin N-terminal domain	-0.208
PF02225	+	PA domain	-0.209
PF00962	-	Adenosine/AMP deaminase	-0.234
PF02311	-	AraC-like ligand binding domain	-0.239
PF06293	-	Lipopolysaccharide kinase (Kdo/WaaP) family	-0.259
PF02275	-	Linear amide C-N hydrolases, choloylglycine hydrolase family	-0.259
PF01076	-	Plasmid recombination enzyme	-0.275

PF13587	-	N-terminal domain of DJ-1_PfpI family	-0.292
PF06199	-	Phage major tail protein 2	-0.292
PF00071	-	Ras family	-0.296
PF07022	-	Bacteriophage CI repressor helix-turn-helix domain	-0.298
PF03729	-	Short repeat of unknown function (DUF308)	-0.299
PF05065	-	Phage capsid family	-0.325
PF00459	-	Inositol monophosphatase family	-0.363
PF03590	-	Aspartate-ammonia ligase	-0.383
PF06971	-	Putative DNA-binding protein N-terminus	-0.473
PF01219	-	Prokaryotic diacylglycerol kinase	-0.626

Phenotype: Colistin-Polymyxin susceptible (Growth), Predictor: phypat

Pfam	class	Pfam_desc	cor
PF00263	+	Bacterial type II and III secretion system protein	0.578
PF00873	+	AcrB/AcrD/AcrF family	0.532
PF03279	+	Bacterial lipid A biosynthesis acyltransferase	0.513
PF03892	+	Nitrate reductase cytochrome c-type subunit (NapB)	0.506
PF03350	+	Uncharacterized protein family, UPF0114	0.503
PF06808	+	DctM-like transporters	0.497
PF03824	+	High-affinity nickel-transport protein	0.496
PF10707	+	PhoP regulatory network protein YrbL	0.490
PF04241	+	Protein of unknown function (DUF423)	0.486
PF00593	+	TonB dependent receptor	0.485
PF04076	+	Bacterial OB fold (BOF) protein	0.479
PF04461	+	Protein of unknown function (DUF520)	0.476
PF02682	+	Allophanate hydrolase subunit 1	0.458
PF06826	+	Predicted Permease Membrane Region	0.442
PF14301	+	Domain of unknown function (DUF4376)	0.441
PF08212	+	Lipocalin-like domain	0.436
PF00959	+	Phage lysozyme	0.432
PF03605	+	Anaerobic c4-dicarboxylate membrane transporter	0.414
PF00565	+	Staphylococcal nuclease homologue	0.397
PF04286	+	Protein of unknown function (DUF445)	0.387
PF02667	+	Short chain fatty acid transporter	0.385
PF02040	+	Arsenical pump membrane protein	0.384
PF04290	+	Tripartite ATP-independent periplasmic transporters, DctQ component	0.382
PF04945	+	YHS domain	0.368
PF03480	+	Bacterial extracellular solute-binding protein, family 7	0.363
PF04284	+	Protein of unknown function (DUF441)	0.360
PF01293	+	Phosphoenolpyruvate carboxykinase	0.359
PF03711	+	Orn/Lys/Arg decarboxylase, C-terminal domain	0.359
PF11575	+	FhuF 2Fe-2S C-terminal domain	0.349
PF08803	+	Putative mono-oxygenase ydhR	0.347
PF02146	+	Sir2 family	0.340
PF03069	+	Acetamidase/Formamidase family	0.333
PF01558	+	Pyruvate ferredoxin/flavodoxin oxidoreductase	0.309
PF10369	+	Small subunit of acetolactate synthase	0.307
PF00023	+	Ankyrin repeat	0.302

PF14502	+	Helix-turn-helix domain	0.290
PF13379	+	NMT1-like family	0.284
PF01638	+	HxlR-like helix-turn-helix	0.276
PF05971	+	Protein of unknown function (DUF890)	0.273
PF02694	+	Uncharacterised BCR, YnfA/UPF0060 family	0.262
PF13555	+	P-loop containing region of AAA domain	0.225
PF00135	+	Carboxylesterase family	0.202
PF04221	+	RelB antitoxin	0.141
PF02311	+	AraC-like ligand binding domain	0.130
PF11066	-	Protein of unknown function (DUF2867)	0.116
PF01610	+	Transposase	0.089
PF02475	+	Met-10+ like-protein	0.031
PF11870	-	Domain of unknown function (DUF3390)	-0.041
PF00668	-	Condensation domain	-0.059
PF00114	-	Pilin (bacterial filament)	-0.059
PF04909	-	Amidohydrolase	-0.079
PF04820	-	Tryptophan halogenase	-0.083
PF05014	-	Nucleoside 2-deoxyribosyltransferase	-0.102
PF13431	-	Tetratricopeptide repeat	-0.102
PF01235	-	Sodium:alanine symporter family	-0.103
PF02589	-	Uncharacterised ACR, YkgG family COG1556	-0.104
PF04962	-	KduI/IolB family	-0.104
PF04471	-	Restriction endonuclease	-0.112
PF08546	-	Ketopantoate reductase PanE/ApbA C terminal	-0.122
PF13518	-	Helix-turn-helix domain	-0.122
PF10412	-	Type IV secretion-system coupling protein DNA-binding domain	-0.125
PF01894	-	Uncharacterised protein family UPF0047	-0.146
PF02806	-	Alpha amylase, C-terminal all-beta domain	-0.148
PF06792	-	Uncharacterised protein family (UPF0261)	-0.167
PF08240	-	Alcohol dehydrogenase GroES-like domain	-0.233
PF00657	-	GDSL-like Lipase/Acylhydrolase	-0.257
PF00302	-	Chloramphenicol acetyltransferase	-0.258
PF02274	-	Amidinotransferase	-0.271
PF02498	-	BRO family, N-terminal domain	-0.271
PF04191	-	Phospholipid methyltransferase	-0.306
PF13275	-	S4 domain	-0.311
PF02838	-	Glycosyl hydrolase family 20, domain 2	-0.329
PF00176	-	SNF2 family N-terminal domain	-0.333
PF00690	-	Cation transporter/ATPase, N-terminus	-0.335
PF10544	-	T5orf172 domain	-0.340
PF13345	-	Domain of unknown function (DUF4098)	-0.356
PF13246	-	Putative hydrolase of sodium-potassium ATPase alpha subunit	-0.360
PF13455	-	Meiotically up-regulated gene 113	-0.360
PF02884	-	Polysaccharide lyase family 8, C-terminal beta-sandwich domain	-0.365
PF02734	-	DAK2 domain	-0.368
PF07475	-	HPr Serine kinase C-terminal domain	-0.382
PF02486	-	Replication initiation factor	-0.405
PF02278	-	Polysaccharide lyase family 8, super-sandwich domain	-0.407

PF07739	-	TipAS antibiotic-recognition domain	-0.413
PF02645	-	Uncharacterised protein, DegV family COG1307	-0.440
PF01076	-	Plasmid recombination enzyme	-0.449
PF01182	-	Glucosamine-6-phosphate isomerases/6-phosphogluconolactonase	-0.456
PF06081	-	Bacterial protein of unknown function (DUF939)	-0.483
PF02608	-	Basic membrane protein	-0.491

Phenotype: Colistin-Polymyxin susceptible (Growth), Predictor: phypat+PGL

Pfam	class	Pfam_desc	cor
PF00263	+	Bacterial type II and III secretion system protein	0.578
PF00873	+	AcrB/AcrD/AcrF family	0.532
PF03350	+	Uncharacterized protein family, UPF0114	0.503
PF06808	+	DctM-like transporters	0.497
PF03824	+	High-affinity nickel-transport protein	0.496
PF07549	+	SecD/SecF GG Motif	0.495
PF04241	+	Protein of unknown function (DUF423)	0.486
PF04748	+	Divergent polysaccharide deacetylase	0.457
PF02578	+	Multi-copper polyphenol oxidoreductase laccase	0.455
PF03737	+	Demethylmenaquinone methyltransferase	0.438
PF08212	+	Lipocalin-like domain	0.436
PF01810	+	LysE type translocator	0.433
PF04879	+	Molybdopterin oxidoreductase Fe4S4 domain	0.424
PF04247	+	Invasion gene expression up-regulator, SirB	0.417
PF05728	+	Uncharacterised protein family (UPF0227)	0.393
PF02541	+	Ppx/GppA phosphatase family	0.390
PF04286	+	Protein of unknown function (DUF445)	0.387
PF12167	+	Domain of unknown function (DUF3596)	0.384
PF04290	+	Tripartite ATP-independent periplasmic transporters, DctQ component	0.382
PF01019	+	Gamma-glutamyltranspeptidase	0.377
PF13974	+	YebO-like protein	0.373
PF10697	+	Protein of unknown function (DUF2502)	0.373
PF04945	+	YHS domain	0.368
PF03212	+	Pertactin	0.368
PF01293	+	Phosphoenolpyruvate carboxykinase	0.359
PF11612	+	Type II secretion system (T2SS), protein J	0.347
PF06415	+	BPG-independent PGAM N-terminus (iPGM_N)	0.333
PF07287	+	Protein of unknown function (DUF1446)	0.333
PF13644	+	DKNYY family	0.316
PF02230	+	Phospholipase/Carboxylesterase	0.313
PF04235	+	Protein of unknown function (DUF418)	0.310
PF01558	+	Pyruvate ferredoxin/flavodoxin oxidoreductase	0.309
PF10369	+	Small subunit of acetolactate synthase	0.307
PF05872	+	Bacterial protein of unknown function (DUF853)	0.298
PF13704	+	Glycosyl transferase family 2	0.288
PF08298	+	PrkA AAA domain	0.269
PF07286	+	Protein of unknown function (DUF1445)	0.267
PF05845	+	Bacterial phosphonate metabolism protein (PhnH)	0.262
PF06007	+	Phosphonate metabolism protein PhnJ	0.262

PF13244	+	Domain of unknown function (DUF4040)	0.257
PF06074	+	Protein of unknown function (DUF935)	0.237
PF01095	+	Pectinesterase	0.233
PF00782	-	Dual specificity phosphatase, catalytic domain	0.231
PF14281	+	PD-(D/E)XK nuclease superfamily	0.230
PF05069	+	Phage virion morphogenesis family	0.230
PF14226	+	non-haem dioxygenase in morphine synthesis N-terminal	0.213
PF07030	+	Protein of unknown function (DUF1320)	0.206
PF00135	+	Carboxylesterase family	0.202
PF13336	+	Acetyl-CoA hydrolase/transferase C-terminal domain	0.194
PF02617	-	ATP-dependent Clp protease adaptor protein ClpS	0.194
PF07070	+	SpoOM protein	0.190
PF13239	+	2TM domain	0.190
PF03829	+	PTS system glucitol/sorbitol-specific IIA component	0.183
PF09347	+	Domain of unknown function (DUF1989)	0.177
PF03808	+	Glycosyl transferase WecB/TagA/CpsF family	0.176
PF01068	+	ATP dependent DNA ligase domain	0.176
PF12392	+	Collagenase	0.175
PF04134	+	Protein of unknown function, DUF393	0.171
PF05400	-	Flagellar protein FliT	0.165
PF03588	-	Leucyl/phenylalanyl-tRNA protein transferase	0.162
PF07228	+	Stage II sporulation protein E (SpoIIE)	0.143
PF07352	+	Bacteriophage Mu Gam like protein	0.135
PF07804	-	HipA-like C-terminal domain	0.135
PF13801	-	Heavy-metal resistance	0.132
PF02311	+	AraC-like ligand binding domain	0.130
PF15604	+	Putative toxin 43	0.125
PF15538	+	Putative toxin 61	0.125
PF03929	+	PepSY-associated TM helix	0.125
PF05532	+	CsbD-like	0.122
PF04963	-	Sigma-54 factor, core binding domain	0.122
PF03435	+	Saccharopine dehydrogenase	0.121
PF06834	-	TraU protein	0.106
PF00325	-	Bacterial regulatory proteins, crp family	0.104
PF00255	+	Glutathione peroxidase	0.104
PF02929	+	Beta galactosidase small chain	0.102
PF05930	-	Prophage CP4-57 regulatory protein (AlpA)	0.096
PF01008	+	Initiation factor 2 subunit family	0.078
PF05076	+	Suppressor of fused protein (SUFU)	0.072
PF13657	-	HipA N-terminal domain	0.067
PF01161	-	Phosphatidylethanolamine-binding protein	0.066
PF14568	+	SMI1-KNR4 cell-wall	0.065
PF09838	-	Uncharacterized protein conserved in bacteria (DUF2065)	0.060
PF02839	-	Carbohydrate binding domain	0.060
PF03806	+	AbgT putative transporter family	0.047
PF09346	+	SMI1 / KNR4 family (SUKH-1)	0.043
PF02016	+	LD-carboxypeptidase	0.041
PF12476	+	Protein of unknown function (DUF3696)	0.039

PF02475	+	Met-10+ like-protein	0.031
PF05187	-	Electron transfer flavoprotein-ubiquinone oxidoreductase	0.022
PF14827	-	Sensory domain of two-component sensor kinase	-0.002
PF03649	-	Uncharacterised protein family (UPF0014)	-0.007
PF01447	-	Thermolysin metallopeptidase, catalytic domain	-0.031
PF13599	-	Pentapeptide repeats (9 copies)	-0.032
PF11870	-	Domain of unknown function (DUF3390)	-0.041
PF06564	-	YhjQ protein	-0.047
PF01915	-	Glycosyl hydrolase family 3 C-terminal domain	-0.050
PF14241	-	Domain of unknown function (DUF4341)	-0.051
PF02868	-	Thermolysin metallopeptidase, alpha-helical domain	-0.054
PF04131	+	Putative N-acetylmannosamine-6-phosphate epimerase	-0.063
PF03787	+	RAMP superfamily	-0.071
PF13480	-	Acetyltransferase (GNAT) domain	-0.077
PF04909	-	Amidohydrolase	-0.079
PF13007	-	Transposase C of IS166 homeodomain	-0.083
PF01880	+	Desulfoferrodoxin	-0.083
PF13551	-	Winged helix-turn helix	-0.095
PF00282	-	Pyridoxal-dependent decarboxylase conserved domain	-0.104
PF00891	-	O-methyltransferase	-0.110
PF04471	-	Restriction endonuclease	-0.112
PF05544	-	Proline racemase	-0.116
PF01680	+	SOR/SNZ family	-0.116
PF13518	-	Helix-turn-helix domain	-0.122
PF07943	-	Penicillin-binding protein 5, C-terminal domain	-0.128
PF00393	-	6-phosphogluconate dehydrogenase, C-terminal domain	-0.129
PF03382	+	Mycoplasma protein of unknown function, DUF285	-0.129
PF07603	-	Protein of unknown function (DUF1566)	-0.129
PF13683	-	Integrase core domain	-0.132
PF00545	-	ribonuclease	-0.135
PF02595	-	Glycerate kinase family	-0.143
PF01894	-	Uncharacterised protein family UPF0047	-0.146
PF02806	-	Alpha amylase, C-terminal all-beta domain	-0.148
PF13229	-	Right handed beta helix region	-0.164
PF13730	-	Helix-turn-helix domain	-0.167
PF07831	-	Pyrimidine nucleoside phosphorylase C-terminal domain	-0.176
PF13636	-	pre-rRNA processing and ribosome biogenesis	-0.177
PF00401	-	ATP synthase, Delta/Epsilon chain, long alpha-helix domain	-0.206
PF02543	-	Carbamoyltransferase	-0.209
PF13936	-	Helix-turn-helix domain	-0.210
PF00872	-	Transposase, Mutator family	-0.222
PF10551	-	MULE transposase domain	-0.225
PF13305	-	WHG domain	-0.241
PF03050	-	Transposase IS66 family	-0.256
PF11208	-	Protein of unknown function (DUF2992)	-0.263
PF00082	-	Subtilase family	-0.288
PF13472	-	GDSL-like Lipase/Acylhydrolase family	-0.290
PF06961	-	Protein of unknown function (DUF1294)	-0.315

PF02838	-	Glycosyl hydrolase family 20, domain 2	-0.329
PF13345	-	Domain of unknown function (DUF4098)	-0.356
PF13455	-	Meiotically up-regulated gene 113	-0.360
PF00069	-	Protein kinase domain	-0.365
PF02278	-	Polysaccharide lyase family 8, super-sandwich domain	-0.407
PF07739	-	TipAS antibiotic-recognition domain	-0.413
PF13578	-	Methyltransferase domain	-0.433
PF02608	-	Basic membrane protein	-0.491

Phenotype: DNase (Growth), Predictor: phypat

Pfam	class	Pfam_desc	cor
PF07968	+	Leukocidin/Hemolysin toxin family	0.596
PF00801	+	PKD domain	0.378
PF00850	+	Histone deacetylase domain	0.357
PF02588	+	Uncharacterized BCR, YitT family COG1284	0.347
PF03174	+	Chitobiase/beta-hexosaminidase C-terminal domain	0.346
PF04151	+	Bacterial pre-peptidase C-terminal domain	0.346
PF13424	+	Tetratricopeptide repeat	0.320
PF03067	+	Chitin binding domain	0.310
PF01126	+	Heme oxygenase	0.310
PF01633	+	Choline/ethanolamine kinase	0.305
PF06415	+	BPG-independent PGAM N-terminus (iPGM_N)	0.296
PF00728	+	Glycosyl hydrolase family 20, catalytic domain	0.293
PF00265	+	Thymidine kinase	0.281
PF03767	+	HAD superfamily, subfamily IIIB (Acid phosphatase)	0.261
PF12242	+	NAD(P)H binding domain of trans-2-enoyl-CoA reductase	0.246
PF03073	+	TspO/MBR family	0.234
PF13529	+	Peptidase_C39 like family	0.231
PF00082	+	Subtilase family	0.227
PF12821	+	Protein of unknown function (DUF3815)	0.198
PF08298	+	PrkA AAA domain	0.162
PF00487	+	Fatty acid desaturase	0.107
PF13482	+	RNase_H superfamily	0.102
PF04932	-	O-Antigen ligase	-0.151
PF04138	-	GtrA-like protein	-0.191
PF00923	-	Transaldolase	-0.191
PF02016	-	LD-carboxypeptidase	-0.197
PF01566	-	Natural resistance-associated macrophage protein	-0.198
PF08212	-	Lipocalin-like domain	-0.204
PF13580	-	SIS domain	-0.207
PF01935	-	Domain of unknown function DUF87	-0.208
PF01890	-	Cobalamin synthesis G C-terminus	-0.208
PF06912	-	Protein of unknown function (DUF1275)	-0.234
PF12682	-	Flavodoxin	-0.236
PF13103	-	TonB C terminal	-0.237
PF01555	-	DNA methylase	-0.242
PF00848	-	Ring hydroxylating alpha subunit (catalytic domain)	-0.254
PF13090	-	Polyphosphate kinase C-terminal domain	-0.257

PF01960	-	ArgJ family	-0.266
PF01661	-	Macro domain	-0.270
PF03432	-	Relaxase/Mobilisation nuclease domain	-0.309
PF04239	-	Protein of unknown function (DUF421)	-0.316
PF05992	-	SbmA/BacA-like family	-0.318
PF06472	-	ABC transporter transmembrane region 2	-0.318
PF01734	-	Patatin-like phospholipase	-0.319
PF04284	-	Protein of unknown function (DUF441)	-0.336
PF01094	-	Receptor family ligand binding region	-0.343
PF04221	-	RelB antitoxin	-0.347
PF02627	-	Carboxymuconolactone decarboxylase family	-0.354
PF00654	-	Voltage gated chloride channel	-0.378
PF00465	-	Iron-containing alcohol dehydrogenase	-0.380
PF13473	-	Cupredoxin-like domain	-0.417

Phenotype: DNase (Growth), Predictor: phypat+PGL

Pfam	class	Pfam_desc	cor
PF09818	+	Predicted ATPase of the ABC class	0.481
PF05547	+	Immune inhibitor A peptidase M6	0.476
PF07070	+	SpoOM protein	0.423
PF01731	+	Arylesterase	0.414
PF00801	+	PKD domain	0.378
PF00850	+	Histone deacetylase domain	0.357
PF04018	+	Domain of unknown function (DUF368)	0.346
PF03174	+	Chitobiase/beta-hexosaminidase C-terminal domain	0.346
PF10517	+	Electron transfer DM13	0.343
PF01769	+	Divalent cation transporter	0.337
PF08449	+	UAA transporter family	0.336
PF03929	+	PepSY-associated TM helix	0.336
PF12458	+	ATPase involved in DNA repair	0.336
PF11074	+	Domain of unknown function(DUF2779)	0.336
PF00949	+	Peptidase S7, Flavivirus NS3 serine protease	0.336
PF07198	+	Protein of unknown function (DUF1410)	0.336
PF00209	+	Sodium:neurotransmitter symporter family	0.335
PF01752	+	Collagenase	0.311
PF03496	+	ADP-ribosyltransferase exoenzyme	0.311
PF10552	+	ORF6C domain	0.311
PF05569	+	BlaR1 peptidase M56	0.295
PF13772	+	AIG2-like family	0.279
PF03767	+	HAD superfamily, subfamily IIIB (Acid phosphatase)	0.261
PF00932	+	Lamin Tail Domain	0.257
PF09903	+	Uncharacterized protein conserved in bacteria (DUF2130)	0.243
PF02958	+	Ecdysteroid kinase	0.243
PF04191	+	Phospholipid methyltransferase	0.243
PF02544	+	3-oxo-5-alpha-steroid 4-dehydrogenase	0.235
PF12811	+	Bax inhibitor 1 like	0.216
PF05219	+	DREV methyltransferase	0.212
PF02568	+	Thiamine biosynthesis protein (ThiI)	0.208

PF02030	+	Hypothetical lipoprotein (MG045 family)	0.204
PF14337	+	Domain of unknown function (DUF4393)	0.199
PF05076	+	Suppressor of fused protein (SUFU)	0.198
PF08495	+	FIST N domain	0.192
PF10442	+	FIST C domain	0.192
PF08325	+	WLM domain	0.192
PF05891	+	AdoMet dependent proline di-methyltransferase	0.189
PF12476	+	Protein of unknown function (DUF3696)	0.189
PF05971	+	Protein of unknown function (DUF890)	0.181
PF14903	+	WG containing repeat	0.169
PF08298	+	PrkA AAA domain	0.162
PF14539	+	Domain of unknown function (DUF4442)	0.151
PF01443	+	Viral (Superfamily 1) RNA helicase	0.151
PF05729	-	NACHT domain	0.148
PF03936	+	Terpene synthase family, metal binding domain	0.115
PF02450	+	Lecithin:cholesterol acyltransferase	0.115
PF13391	+	HNH endonuclease	0.114
PF13482	+	RNase_H superfamily	0.102
PF14595	+	Thioredoxin	0.096
PF06803	+	Protein of unknown function (DUF1232)	0.082
PF14281	+	PD-(D/E)XK nuclease superfamily	0.080
PF14390	+	Domain of unknown function (DUF4420)	0.080
PF03372	+	Endonuclease/Exonuclease/phosphatase family	0.071
PF04466	+	Phage terminase large subunit	0.061
PF04326	+	Divergent AAA domain	0.049
PF03096	+	Ndr family	0.004
PF00762	-	Ferrochelatase	-0.011
PF07670	-	Nucleoside recognition	-0.037
PF13248	+	zinc-ribbon domain	-0.039
PF12696	-	TraM recognition site of TraD and TraG	-0.052
PF07166	-	Protein of unknown function (DUF1398)	-0.057
PF10544	-	T5orf172 domain	-0.059
PF00872	+	Transposase, Mutator family	-0.092
PF05708	-	Orthopoxvirus protein of unknown function (DUF830)	-0.108
PF13936	-	Helix-turn-helix domain	-0.113
PF00302	-	Chloramphenicol acetyltransferase	-0.120
PF08007	-	Cupin superfamily protein	-0.143
PF07377	-	Protein of unknown function (DUF1493)	-0.151
PF00208	-	Glutamate/Leucine/Phenylalanine/Valine dehydrogenase	-0.157
PF01306	-	LacY proton/sugar symporter	-0.162
PF13378	-	Enolase C-terminal domain-like	-0.164
PF08401	-	Domain of unknown function (DUF1738)	-0.171
PF11974	-	Alpha-2-macroglobulin MG1 domain	-0.174
PF06754	-	Phosphonate metabolism protein PhnG	-0.180
PF05872	-	Bacterial protein of unknown function (DUF853)	-0.184
PF06564	-	YhjQ protein	-0.192
PF13338	-	Domain of unknown function (DUF4095)	-0.199
PF14310	-	Fibronectin type III-like domain	-0.204

PF00325	-	Bacterial regulatory proteins, crp family	-0.207
PF13580	-	SIS domain	-0.207
PF03894	-	D-xylulose 5-phosphate/D-fructose 6-phosphate phosphoketolase	-0.208
PF07411	-	Domain of unknown function (DUF1508)	-0.227
PF12682	-	Flavodoxin	-0.236
PF03170	-	Bacterial cellulose synthase subunit	-0.246
PF02550	-	Acetyl-CoA hydrolase/transferase N-terminal domain	-0.256
PF13596	-	PAS domain	-0.256
PF13089	-	Polyphosphate kinase N-terminal domain	-0.257
PF02503	-	Polyphosphate kinase middle domain	-0.257
PF13007	-	Transposase C of IS166 homeodomain	-0.286
PF02092	-	Glycyl-tRNA synthetase beta subunit	-0.292
PF10423	-	Bacterial AMP nucleoside phosphorylase N-terminus	-0.300
PF03432	-	Relaxase/Mobilisation nuclease domain	-0.309
PF03633	-	Glycosyl hydrolase family 65, C-terminal domain	-0.311
PF02705	-	K+ potassium transporter	-0.320
PF14821	-	Threonine synthase N terminus	-0.327
PF01094	-	Receptor family ligand binding region	-0.343
PF02627	-	Carboxymuconolactone decarboxylase family	-0.354
PF13473	-	Cupredoxin-like domain	-0.417

Phenotype: Growth at 42¬∞C (Growth), Predictor: phypat

Pfam	class	Pfam_desc	cor
PF13408	+	Recombinase zinc beta ribbon domain	0.468
PF01972	+	Serine dehydrogenase proteinase	0.455
PF04883	+	Bacteriophage HK97-gp10, putative tail-component	0.447
PF02568	+	Thiamine biosynthesis protein (ThiI)	0.445
PF05065	+	Phage capsid family	0.441
PF06580	+	Histidine kinase	0.433
PF13483	+	Beta-lactamase superfamily domain	0.418
PF04229	+	GrpB protein	0.410
PF04464	+	CDP-Glycerol:Poly(glycerophosphate) glycerophosphotransferase	0.407
PF06445	+	GyrI-like small molecule binding domain	0.371
PF12895	+	Anaphase-promoting complex, cyclosome, subunit 3	0.369
PF02274	+	Amidinotransferase	0.357
PF14635	+	Helix-hairpin-helix motif	0.352
PF03475	+	3-alpha domain	0.352
PF01814	+	Hemerythrin HHE cation binding domain	0.337
PF10662	+	Ethanolamine utilisation - propanediol utilisation	0.333
PF01865	+	Protein of unknown function DUF47	0.330
PF03845	+	Spore germination protein	0.315
PF12323	+	Helix-turn-helix domain	0.312
PF01740	+	STAS domain	0.293
PF13730	+	Helix-turn-helix domain	0.293
PF08463	+	EcoEI R protein C-terminal	0.292
PF01894	+	Uncharacterised protein family UPF0047	0.284
PF14691	+	Dihydroprymidine dehydrogenase domain II, 4Fe-4S cluster	0.258
PF04326	+	Divergent AAA domain	0.241

PF12481	+	Aluminium induced protein	0.205
PF05125	+	Phage major capsid protein, P2 family	0.205
PF05929	+	Phage capsid scaffolding protein (GPO) serine peptidase	0.205
PF05944	+	Phage small terminase subunit	0.205
PF03096	+	Ndr family	0.197
PF04143	+	Sulphur transport	0.169
PF06891	+	P2 phage tail completion protein R (GpR)	0.152
PF00394	+	Multicopper oxidase	0.117
PF13189	-	Cytidylate kinase-like family	-0.054
PF03073	-	TspO/MBR family	-0.084
PF09346	-	SMI1 / KNR4 family (SUKH-1)	-0.128
PF13536	-	Multidrug resistance efflux transporter	-0.129
PF14568	-	SMI1-KNR4 cell-wall	-0.150
PF13425	-	O-antigen ligase like membrane protein	-0.157
PF00614	-	Phospholipase D Active site motif	-0.157
PF01306	-	LacY proton/sugar symporter	-0.167
PF01051	-	Initiator Replication protein	-0.170
PF00697	-	N-(5'phosphoribosyl)anthranilate (PRA) isomerase	-0.179
PF04932	-	O-Antigen ligase	-0.190
PF01935	-	Domain of unknown function DUF87	-0.212
PF06210	-	Protein of unknown function (DUF1003)	-0.213
PF02016	-	LD-carboxypeptidase	-0.215
PF10543	-	ORF6N domain	-0.225
PF13086	-	AAA domain	-0.252
PF07005	-	Protein of unknown function, DUF1537	-0.264
PF14821	-	Threonine synthase N terminus	-0.266
PF07843	-	Protein of unknown function (DUF1634)	-0.269
PF13340	-	Putative transposase of IS4/5 family (DUF4096)	-0.285
PF07536	-	HWE histidine kinase	-0.301
PF07568	-	Histidine kinase	-0.301
PF13586	-	Transposase DDE domain	-0.308
PF03969	-	AFG1-like ATPase	-0.309
PF00146	-	NADH dehydrogenase	-0.310
PF10412	-	Type IV secretion-system coupling protein DNA-binding domain	-0.315
PF01161	-	Phosphatidylethanolamine-binding protein	-0.315
PF02534	-	Type IV secretory system Conjugative DNA transfer	-0.323
PF02503	-	Polyphosphate kinase middle domain	-0.327
PF13090	-	Polyphosphate kinase C-terminal domain	-0.327
PF03169	-	OPT oligopeptide transporter protein	-0.338
PF05015	-	Plasmid maintenance system killer protein	-0.340
PF13089	-	Polyphosphate kinase N-terminal domain	-0.347
PF12399	-	Branched-chain amino acid ATP-binding cassette transporter	-0.359
PF00043	-	Glutathione S-transferase, C-terminal domain	-0.371
PF03797	-	Autotransporter beta-domain	-0.389
PF08479	-	POTRA domain, ShlB-type	-0.408
PF03135	-	CagE, TrbE, VirB family, component of type IV transporter system	-0.474

Phenotype: Growth at 42¬∞C (Growth), Predictor: phypat+PGL

Pfam	class	Pfam_desc	cor
PF04883	+	Bacteriophage HK97-gp10, putative tail-component	0.447
PF05065	+	Phage capsid family	0.441
PF13483	+	Beta-lactamase superfamily domain	0.418
PF06445	+	GyrI-like small molecule binding domain	0.371
PF12895	+	Anaphase-promoting complex, cyclosome, subunit 3	0.369
PF13353	+	4Fe-4S single cluster domain	0.361
PF02274	+	Amidinotransferase	0.357
PF14635	+	Helix-hairpin-helix motif	0.352
PF03354	+	Phage Terminase	0.346
PF02475	+	Met-10+ like-protein	0.343
PF02737	+	3-hydroxyacyl-CoA dehydrogenase, NAD binding domain	0.340
PF00196	+	Bacterial regulatory proteins, luxR family	0.338
PF10662	+	Ethanolamine utilisation - propanediol utilisation	0.333
PF01865	+	Protein of unknown function DUF47	0.330
PF13597	+	Anaerobic ribonucleoside-triphosphate reductase	0.328
PF03845	+	Spore germination protein	0.315
PF01385	+	Probable transposase	0.312
PF01156	+	Inosine-uridine preferring nucleoside hydrolase	0.298
PF01844	+	HNH endonuclease	0.285
PF01894	+	Uncharacterised protein family UPF0047	0.284
PF14659	+	Phage integrase, N-terminal SAM-like domain	0.276
PF01420	+	Type I restriction modification DNA specificity domain	0.272
PF00480	+	ROK family	0.250
PF04326	+	Divergent AAA domain	0.241
PF13246	+	Putative hydrolase of sodium-potassium ATPase alpha subunit	0.239
PF13280	+	WYL domain	0.231
PF02583	+	Metal-sensitive transcriptional repressor	0.227
PF04072	+	Leucine carboxyl methyltransferase	0.220
PF01867	+	CRISPR associated protein Cas1	0.219
PF04342	+	Protein of unknown function, DUF486	0.217
PF12481	+	Aluminium induced protein	0.205
PF01235	+	Sodium:alanine symporter family	0.203
PF03096	+	Ndr family	0.197
PF02652	+	L-lactate permease	0.192
PF01676	+	Metalloenzyme superfamily	0.180
PF01910	+	Domain of unknown function DUF77	0.176
PF12728	+	Helix-turn-helix domain	0.176
PF00990	+	GGDEF domain	0.176
PF03453	+	MoeA N-terminal region (domain I and II)	0.176
PF00176	+	SNF2 family N-terminal domain	0.176
PF01174	-	SNO glutamine amidotransferase family	0.168
PF01883	+	Domain of unknown function DUF59	0.167
PF06808	+	DctM-like transporters	0.132
PF01936	+	NYN domain	0.130
PF09278	-	MerR, DNA binding	0.128
PF02388	-	FemAB family	0.128
PF02511	+	Thymidylate synthase complementing protein	0.127

PF08392	+	FAE1/Type III polyketide synthase-like protein	0.117
PF08239	+	Bacterial SH3 domain	0.108
PF01008	-	Initiation factor 2 subunit family	0.103
PF14451	+	Mut7-C ubiquitin	0.100
PF00145	+	C-5 cytosine-specific DNA methylase	0.098
PF03610	-	PTS system fructose IIA component	0.096
PF01470	-	Pyroglutamyl peptidase	0.093
PF08003	+	Protein of unknown function (DUF1698)	0.088
PF01921	+	tRNA synthetases class I (K)	0.085
PF04237	-	YjbR	0.085
PF04214	+	Protein of unknown function, DUF	0.082
PF05231	+	MASE1	0.077
PF03280	+	Proteobacterial lipase chaperone protein	0.077
PF09838	+	Uncharacterized protein conserved in bacteria (DUF2065)	0.077
PF01068	+	ATP dependent DNA ligase domain	0.071
PF05732	+	Firmicute plasmid replication protein (RepL)	0.068
PF02770	+	Acyl-CoA dehydrogenase, middle domain	0.062
PF07885	+	Ion channel	0.059
PF07876	+	Stress responsive A/B Barrel Domain	0.049
PF12680	+	SnoaL-like domain	0.048
PF06271	+	RDD family	0.035
PF07662	-	Na+ dependent nucleoside transporter C-terminus	0.029
PF00082	-	Subtilase family	-0.011
PF13166	-	AAA domain	-0.015
PF01557	-	Fumarylacetoacetate (FAA) hydrolase family	-0.016
PF11700	-	Vacuole effluxer Atg22 like	-0.020
PF12008	-	Type I restriction and modification enzyme - subunit R C terminal	-0.022
PF09924	-	Uncharacterized conserved protein (DUF2156)	-0.025
PF03575	+	Peptidase family S51	-0.033
PF02604	+	Antitoxin Phd_YefM, type II toxin-antitoxin system	-0.034
PF02682	-	Allophanate hydrolase subunit 1	-0.043
PF02626	-	Allophanate hydrolase subunit 2	-0.043
PF07905	-	Purine catabolism regulatory protein-like family	-0.046
PF10017	+	Histidine-specific methyltransferase, SAM-dependent	-0.046
PF01522	-	Polysaccharide deacetylase	-0.046
PF02230	-	Phospholipase/Carboxylesterase	-0.052
PF13189	-	Cytidylate kinase-like family	-0.054
PF12682	-	Flavodoxin	-0.058
PF05171	+	Haemin-degrading HemS.ChuX domain	-0.058
PF13768	-	von Willebrand factor type A domain	-0.071
PF00591	-	Glycosyl transferase family, a/b domain	-0.077
PF04221	-	RelB antitoxin	-0.083
PF05593	-	RHS Repeat	-0.084
PF03073	-	TspO/MBR family	-0.084
PF04261	-	Dyp-type peroxidase family	-0.085
PF03707	-	Bacterial signalling protein N terminal repeat	-0.087
PF01637	-	Archaeal ATPase	-0.096
PF07811	-	TadE-like protein	-0.096

PF04332	-	Protein of unknown function (DUF475)	-0.122
PF07732	-	Multicopper oxidase	-0.123
PF13593	-	SBF-like CPA transporter family (DUF4137)	-0.124
PF02237	-	Biotin protein ligase C terminal domain	-0.124
PF10001	+	Uncharacterized protein conserved in bacteria (DUF2242)	-0.127
PF04314	+	Protein of unknown function (DUF461)	-0.132
PF13404	-	AsnC-type helix-turn-helix domain	-0.146
PF13425	-	O-antigen ligase like membrane protein	-0.157
PF00614	-	Phospholipase D Active site motif	-0.157
PF01306	-	LacY proton/sugar symporter	-0.167
PF01051	-	Initiator Replication protein	-0.170
PF13567	-	Domain of unknown function (DUF4131)	-0.175
PF10994	-	Protein of unknown function (DUF2817)	-0.177
PF04480	-	Protein of unknown function (DUF559)	-0.180
PF00290	-	Tryptophan synthase alpha chain	-0.198
PF03681	-	Uncharacterised protein family (UPF0150)	-0.206
PF11941	-	Domain of unknown function (DUF3459)	-0.206
PF06210	-	Protein of unknown function (DUF1003)	-0.213
PF14588	-	YjgF/chorismate_mutase-like, putative endoribonuclease	-0.213
PF10099	-	Anti-sigma-K factor rskA	-0.222
PF03775	-	Septum formation inhibitor MinC, C-terminal domain	-0.264
PF12172	-	Rubredoxin-like zinc ribbon domain (DUF35_N)	-0.282
PF11920	-	Protein of unknown function (DUF3438)	-0.282
PF07568	-	Histidine kinase	-0.301
PF03969	-	AFG1-like ATPase	-0.309
PF01161	-	Phosphatidylethanolamine-binding protein	-0.315
PF12696	-	TraM recognition site of TraD and TraG	-0.323
PF02534	-	Type IV secretory system Conjugative DNA transfer	-0.323
PF02913	-	FAD linked oxidases, C-terminal domain	-0.325
PF03169	-	OPT oligopeptide transporter protein	-0.338
PF12399	-	Branched-chain amino acid ATP-binding cassette transporter	-0.359
PF03865	-	Haemolysin secretion/activation protein ShlB/FhaC/HecB	-0.378
PF03797	-	Autotransporter beta-domain	-0.389
PF08479	-	POTRA domain, ShlB-type	-0.408

Phenotype: Growth in 6.5% NaCl (Growth), Predictor: phypat

Pfam	class	Pfam_desc	cor
PF13456	+	Reverse transcriptase-like	0.721
PF13743	+	Thioredoxin	0.699
PF06778	+	Chlorite dismutase	0.633
PF04978	+	Protein of unknown function (DUF664)	0.559
PF03881	+	Fructosamine kinase	0.535
PF01904	+	Protein of unknown function DUF72	0.519
PF08338	+	Domain of unknown function (DUF1731)	0.504
PF02628	+	Cytochrome oxidase assembly protein	0.476
PF07831	+	Pyrimidine nucleoside phosphorylase C-terminal domain	0.476
PF13653	+	Glycerophosphoryl diester phosphodiesterase family	0.475
PF14827	+	Sensory domain of two-component sensor kinase	0.467

PF02436	+	Conserved carboxylase domain	0.464
PF04893	+	Yip1 domain	0.445
PF01566	+	Natural resistance-associated macrophage protein	0.440
PF06930	+	Protein of unknown function (DUF1282)	0.436
PF13806	+	Rieske-like [2Fe-2S] domain	0.428
PF03649	+	Uncharacterised protein family (UPF0014)	0.419
PF13305	+	WHG domain	0.414
PF03706	+	Uncharacterised protein family (UPF0104)	0.391
PF14657	+	AP2-like DNA-binding integrase domain	0.384
PF02909	+	Tetracyclin repressor, C-terminal all-alpha domain	0.356
PF09587	+	Bacterial capsule synthesis protein PGA_cap	0.281
PF13601	+	Winged helix DNA-binding domain	0.247
PF01923	+	Cobalamin adenosyltransferase	0.232
PF07275	+	Antirestriction protein (ArdA)	0.146
PF13360	+	PQQ-like domain	0.085
PF13263	-	PHP-associated	-0.034
PF14595	-	Thioredoxin	-0.082
PF01220	-	Dehydroquinase class II	-0.160
PF09359	-	VTC domain	-0.180
PF13292	-	1-deoxy-D-xylulose-5-phosphate synthase	-0.254
PF03459	-	TOBE domain	-0.291
PF00146	-	NADH dehydrogenase	-0.313
PF13386	-	Cytochrome C biogenesis protein transmembrane region	-0.322
PF05167	-	Uncharacterised ACR (DUF711)	-0.322
PF01914	-	MarC family integral membrane protein	-0.327
PF02705	-	K+ potassium transporter	-0.335
PF03577	-	Peptidase family C69	-0.337
PF03444	-	Winged helix-turn-helix transcription repressor, HrcA DNA-binding	-0.346
PF03472	-	Autoinducer binding domain	-0.348
PF07927	-	YcfA-like protein	-0.361
PF12724	-	Flavodoxin domain	-0.365
PF01555	-	DNA methylase	-0.367
PF13353	-	4Fe-4S single cluster domain	-0.392
PF12738	-	twin BRCT domain	-0.406
PF08238	-	Sel1 repeat	-0.423
PF07244	-	Surface antigen variable number repeat	-0.449
PF01103	-	Surface antigen	-0.449
PF00654	-	Voltage gated chloride channel	-0.479
PF04011	-	LemA family	-0.497

Phenotype: Growth in 6.5% NaCl (Growth), Predictor: phypat+PGL

Pfam	class	Pfam_desc	cor
PF13456	+	Reverse transcriptase-like	0.721
PF13743	+	Thioredoxin	0.699
PF04306	+	Protein of unknown function (DUF456)	0.620
PF02700	+	Phosphoribosylformylglycinamidine (FGAM) synthase	0.579
PF03881	+	Fructosamine kinase	0.535
PF11716	+	Mycothiol maleylpyruvate isomerase N-terminal domain	0.529

PF09685	+	Tic20-like protein	0.527
PF01910	+	Domain of unknown function DUF77	0.509
PF13398	+	Peptidase M50B-like	0.506
PF08338	+	Domain of unknown function (DUF1731)	0.504
PF03806	+	AbgT putative transporter family	0.488
PF07831	+	Pyrimidine nucleoside phosphorylase C-terminal domain	0.476
PF13653	+	Glycerophosphoryl diester phosphodiesterase family	0.475
PF14827	+	Sensory domain of two-component sensor kinase	0.467
PF02436	+	Conserved carboxylase domain	0.464
PF04893	+	Yip1 domain	0.445
PF14689	+	Sensor_kinase_SpoOB-type, alpha-helical domain	0.437
PF07907	+	YibE/F-like protein	0.436
PF06930	+	Protein of unknown function (DUF1282)	0.436
PF00282	+	Pyridoxal-dependent decarboxylase conserved domain	0.428
PF13806	+	Rieske-like [2Fe-2S] domain	0.428
PF09922	+	Cell wall-active antibiotics response protein (DUF2154)	0.428
PF13530	+	Sterol carrier protein domain	0.413
PF03102	+	NeuB family	0.404
PF04039	+	Domain related to MnhB subunit of Na+/H+ antiporter	0.397
PF13244	+	Domain of unknown function (DUF4040)	0.397
PF01899	+	Na+/H+ ion antiporter subunit	0.397
PF03334	+	Na+/H+ antiporter subunit	0.397
PF13468	+	Glyoxalase-like domain	0.391
PF03632	+	Glycosyl hydrolase family 65 central catalytic domain	0.381
PF03636	+	Glycosyl hydrolase family 65, N-terminal domain	0.381
PF12146	+	Putative lysophospholipase	0.380
PF08392	+	FAE1/Type III polyketide synthase-like protein	0.375
PF12464	+	Maltose acetyltransferase	0.365
PF06874	+	Firmicute fructose-1,6-bisphosphatase	0.356
PF01769	+	Divalent cation transporter	0.354
PF14542	+	GCN5-related N-acetyl-transferase	0.354
PF13434	+	L-lysine 6-monooxygenase (NADPH-requiring)	0.346
PF00209	+	Sodium:neurotransmitter symporter family	0.345
PF11314	+	Protein of unknown function (DUF3117)	0.339
PF01738	+	Dienelactone hydrolase family	0.335
PF08280	+	M protein trans-acting positive regulator (MGA) HTH domain	0.301
PF03606	+	C4-dicarboxylate anaerobic carrier	0.301
PF02656	+	Domain of unknown function (DUF202)	0.296
PF13726	+	Na+-H+ antiporter family	0.291
PF01928	+	CYTH domain	0.285
PF00135	-	Carboxylesterase family	0.260
PF11286	+	Protein of unknown function (DUF3087)	0.254
PF01443	+	Viral (Superfamily 1) RNA helicase	0.254
PF13601	+	Winged helix DNA-binding domain	0.247
PF01494	+	FAD binding domain	0.235
PF01923	+	Cobalamin adenosyltransferase	0.232
PF00211	+	Adenylate and Guanylate cyclase catalytic domain	0.181
PF02577	+	Bifunctional nuclease	0.178

PF10011	+	Predicted membrane protein (DUF2254)	0.165
PF08386	+	TAP-like protein	0.162
PF07853	-	Protein of unknown function (DUF1648)	0.160
PF14078	-	Domain of unknown function (DUF4259)	0.152
PF13936	+	Helix-turn-helix domain	0.137
PF00982	+	Glycosyltransferase family 20	0.111
PF13669	+	Glyoxalase/Bleomycin resistance protein/Dioxygenase superfamily	0.099
PF06165	+	Glycosyltransferase family 36	0.094
PF06204	+	Putative carbohydrate binding domain	0.094
PF02796	-	Helix-turn-helix domain of resolvase	0.088
PF13495	-	Phage integrase, N-terminal SAM-like domain	0.080
PF14568	-	SMI1-KNR4 cell-wall	0.073
PF01326	-	Pyruvate phosphate dikinase, PEP/pyruvate binding domain	0.066
PF04277	+	Oxaloacetate decarboxylase, gamma chain	0.048
PF11391	+	Protein of unknown function (DUF2798)	0.029
PF02630	-	SCO1/SenC	0.018
PF04239	-	Protein of unknown function (DUF421)	0.014
PF03575	+	Peptidase family S51	0.005
PF05544	-	Proline racemase	-0.019
PF01809	-	Haemolytic domain	-0.022
PF01643	-	Acyl-ACP thioesterase	-0.050
PF12161	-	HsdM N-terminal domain	-0.061
PF07885	-	Ion channel	-0.074
PF00596	-	Class II Aldolase and Adducin N-terminal domain	-0.093
PF02624	-	YcaO-like family	-0.094
PF08867	-	FRG domain	-0.111
PF05893	-	Acyl-CoA reductase (LuxC)	-0.136
PF02036	-	SCP-2 sterol transfer family	-0.155
PF13187	-	4Fe-4S dicluster domain	-0.193
PF01464	-	Transglycosylase SLT domain	-0.207
PF01512	-	Respiratory-chain NADH dehydrogenase 51 Kd subunit	-0.214
PF01420	-	Type I restriction modification DNA specificity domain	-0.215
PF02685	-	Glucokinase	-0.223
PF02086	-	D12 class N6 adenine-specific DNA methyltransferase	-0.281
PF01094	-	Receptor family ligand binding region	-0.287
PF00146	-	NADH dehydrogenase	-0.313
PF01223	-	DNA/RNA non-specific endonuclease	-0.321
PF13386	-	Cytochrome C biogenesis protein transmembrane region	-0.322
PF01914	-	MarC family integral membrane protein	-0.327
PF14697	-	4Fe-4S dicluster domain	-0.339
PF03472	-	Autoinducer binding domain	-0.348
PF12724	-	Flavodoxin domain	-0.365
PF01555	-	DNA methylase	-0.367
PF13353	-	4Fe-4S single cluster domain	-0.392

Phenotype: Growth in KCN (Growth), Predictor: phypat

Pfam	class	Pfam_desc	cor
PF03060	+	Nitronate monooxygenase	0.829

PF07739	+	TipAS antibiotic-recognition domain	0.683
PF04828	+	Glutathione-dependent formaldehyde-activating enzyme	0.676
PF06175	+	tRNA-(MS[2]IO[6]A)-hydroxylase (MiaE)	0.657
PF12852	+	Cupin	0.608
PF03350	+	Uncharacterized protein family, UPF0114	0.542
PF01883	+	Domain of unknown function DUF59	0.478
PF04883	+	Bacteriophage HK97-gp10, putative tail-component	0.478
PF11367	+	Protein of unknown function (DUF3168)	0.478
PF05063	+	MT-A70	0.387
PF11973	+	NQRA C-terminal domain	0.314
PF11659	-	Protein of unknown function (DUF3261)	-0.169
PF04290	-	Tripartite ATP-independent periplasmic transporters, DctQ component	-0.387
PF02283	-	Cobinamide kinase / cobinamide phosphate guanyltransferase	-0.486
PF02654	-	Cobalamin-5-phosphate synthase	-0.486
PF12910	-	Antitoxin of toxin-antitoxin stability system N-terminal	-0.486
PF13186	-	Iron-sulfur cluster-binding domain	-0.486
PF04995	-	Heme exporter protein D (CcmD)	-0.529
PF13269	-	Protein of unknown function (DUF4060)	-0.542
PF03432	-	Relaxase/Mobilisation nuclease domain	-0.543
PF14282	-	FlxA-like protein	-0.567
PF11119	-	Protein of unknown function (DUF2633)	-0.567
PF02585	-	GlcNAc-PI de-N-acetylase	-0.598
PF13006	-	Insertion element 4 transposase N-terminal	-0.608
PF08400	-	Prophage tail fibre N-terminal	-0.655
PF01609	-	Transposase DDE domain	-0.657
PF03892	-	Nitrate reductase cytochrome c-type subunit (NapB)	-0.657
PF03927	-	NapD protein	-0.657
PF05658	-	Head domain of trimeric autotransporter adhesin	-0.714
PF05662	-	Coiled stalk of trimeric autotransporter adhesin	-0.714
PF09829	-	Uncharacterized protein conserved in bacteria (DUF2057)	-0.759

Phenotype: Growth in KCN (Growth), Predictor: phypat+PGL

Pfam	class	Pfam_desc	cor
PF03060	+	Nitronate monooxygenase	0.829
PF04828	+	Glutathione-dependent formaldehyde-activating enzyme	0.676
PF06175	+	tRNA-(MS[2]IO[6]A)-hydroxylase (MiaE)	0.657
PF03350	+	Uncharacterized protein family, UPF0114	0.542
PF08521	+	Two-component sensor kinase N-terminal	0.478
PF06821	+	Serine hydrolase	0.478
PF05232	+	Bacterial Transmembrane Pair family	0.371
PF06226	+	Protein of unknown function (DUF1007)	0.371
PF05726	+	Pirin C-terminal cupin domain	0.371
PF01596	+	O-methyltransferase	0.306
PF01425	+	Amidase	0.200
PF04303	-	PrpF protein	-0.143
PF14031	-	Putative serine dehydratase domain	-0.170
PF01501	-	Glycosyl transferase family 8	-0.240
PF03480	-	Bacterial extracellular solute-binding protein, family 7	-0.387

PF03918	-	Cytochrome C biogenesis protein	-0.447
PF02371	-	Transposase IS116/IS110/IS902 family	-0.478
PF02283	-	Cobinamide kinase / cobinamide phosphate guanyltransferase	-0.486
PF02277	-	Phosphoribosyltransferase	-0.486
PF13333	-	Integrase core domain	-0.488
PF04995	-	Heme exporter protein D (CcmD)	-0.529
PF13269	-	Protein of unknown function (DUF4060)	-0.542
PF03264	-	NapC/NirT cytochrome c family, N-terminal region	-0.573
PF01609	-	Transposase DDE domain	-0.657
PF03892	-	Nitrate reductase cytochrome c-type subunit (NapB)	-0.657

Phenotype: Growth on MacConkey agar (Growth), Predictor: phypat

Pfam	class	Pfam_desc	cor
PF04379	+	Protein of unknown function (DUF525)	0.927
PF01218	+	Coproporphyrinogen III oxidase	0.919
PF06945	+	Protein of unknown function (DUF1289)	0.915
PF11808	+	Domain of unknown function (DUF3329)	0.914
PF00345	+	Pili and flagellar-assembly chaperone, PapD N-terminal domain	0.903
PF03922	+	OmpW family	0.892
PF07869	+	Protein of unknown function (DUF1656)	0.890
PF07152	+	YaeQ protein	0.889
PF13116	+	Protein of unknown function	0.855
PF02753	+	Pili assembly chaperone PapD, C-terminal domain	0.851
PF00419	+	Fimbrial protein	0.851
PF04403	+	Paraquat-inducible protein A	0.825
PF03502	+	Nucleoside-specific channel-forming protein, Tsx	0.804
PF13441	+	YMGG-like Gly-zipper	0.798
PF03588	+	Leucyl/phenylalanyl-tRNA protein transferase	0.789
PF04717	+	Phage-related baseplate assembly protein	0.781
PF09163	+	Formate dehydrogenase N, transmembrane	0.748
PF07063	+	Domain of unknown function (DUF1338)	0.748
PF06067	+	Domain of unknown function (DUF932)	0.738
PF03091	+	CutA1 divalent ion tolerance protein	0.733
PF10636	+	Hemin uptake protein hemP	0.723
PF12769	+	Domain of unknown function (DUF3814)	0.706
PF03594	+	Benzoate membrane transport protein	0.703
PF06798	+	PrkA serine protein kinase C-terminal domain	0.702
PF05962	+	HutD	0.675
PF08212	+	Lipocalin-like domain	0.671
PF00959	+	Phage lysozyme	0.669
PF04325	+	Protein of unknown function (DUF465)	0.608
PF00061	+	Lipocalin / cytosolic fatty-acid binding protein family	0.596
PF13332	+	Haemagluttinin repeat	0.559
PF13618	+	Gluconate 2-dehydrogenase subunit 3	0.537
PF00732	+	GMC oxidoreductase	0.530
PF06506	+	Propionate catabolism activator	0.479
PF06826	+	Predicted Permease Membrane Region	0.472
PF09413	+	Domain of unknown function (DUF2007)	0.463

PF04223	+	Citrate lyase, alpha subunit (CitF)	0.456
PF06035	+	Bacterial transglutaminase-like cysteine proteinase BTLCP	0.455
PF07366	+	SnoaL-like polyketide cyclase	0.425
PF08379	+	Bacterial transglutaminase-like N-terminal region	0.408
PF11391	+	Protein of unknown function (DUF2798)	0.332
PF02706	+	Chain length determinant protein	0.318
PF12846	-	AAA-like domain	0.145
PF02535	-	ZIP Zinc transporter	0.122
PF00391	-	PEP-utilising enzyme, mobile domain	0.065
PF01554	-	MatE	0.065
PF03483	-	B3/4 domain	0.056
PF00128	-	Alpha amylase, catalytic domain	0.052
PF01784	-	NIF3 (NGG1p interacting factor 3)	0.040
PF11987	-	Translation-initiation factor 2	-0.023
PF03448	-	MgtE intracellular N domain	-0.027
PF11738	-	Protein of unknown function (DUF3298)	-0.041
PF02544	-	3-oxo-5-alpha-steroid 4-dehydrogenase	-0.041
PF07726	-	ATPase family associated with various cellular activities (AAA)	-0.063
PF00692	-	dUTPase	-0.069
PF08544	-	GHMP kinases C terminal	-0.077
PF05198	-	Translation initiation factor IF-3, N-terminal domain	-0.078
PF03309	-	Type III pantothenate kinase	-0.084
PF02518	-	Histidine kinase-, DNA gyrase B-, and HSP90-like ATPase	-0.117
PF01926	-	50S ribosome-binding GTPase	-0.117
PF06421	-	GTP-binding protein LepA C-terminus	-0.117
PF00753	-	Metallo-beta-lactamase superfamily	-0.117
PF13184	-	NusA-like KH domain	-0.117
PF00009	-	Elongation factor Tu GTP binding domain	-0.117
PF00113	-	Enolase, C-terminal TIM barrel domain	-0.117
PF01176	-	Translation initiation factor 1A / IF-1	-0.117
PF01541	-	GIY-YIG catalytic domain	-0.117
PF13238	-	AAA domain	-0.123
PF14492	-	Elongation Factor G, domain II	-0.166
PF03952	-	Enolase, N-terminal domain	-0.166
PF04326	-	Divergent AAA domain	-0.188
PF13482	-	RNase_H superfamily	-0.194
PF13672	-	Protein phosphatase 2C	-0.286
PF09827	-	CRISPR associated protein Cas2	-0.367
PF02934	-	GatB/GatE catalytic domain	-0.420
PF13173	-	AAA domain	-0.446
PF01745	-	Isopentenyl transferase	-0.533
PF02457	-	DisA bacterial checkpoint controller nucleotide-binding	-0.593
PF14527	-	WhiA LAGLIDADG-like domain	-0.627
PF02650	-	WhiA C-terminal HTH domain	-0.627
PF03793	-	PASTA domain	-0.687
PF05746	-	DALR anticodon binding domain	

Phenotype: Growth on MacConkey agar (Growth), Predictor: phypat+PGL

Pfam	class	Pfam_desc	cor
PF06945	+	Protein of unknown function (DUF1289)	0.915
PF11808	+	Domain of unknown function (DUF3329)	0.914
PF03922	+	OmpW family	0.892
PF07869	+	Protein of unknown function (DUF1656)	0.890
PF05947	+	Bacterial protein of unknown function (DUF879)	0.864
PF13116	+	Protein of unknown function	0.855
PF05936	+	Bacterial protein of unknown function (DUF876)	0.852
PF08364	+	Bacterial translation initiation factor IF-2 associated region	0.836
PF03886	+	Protein of unknown function (DUF330)	0.827
PF04403	+	Paraquat-inducible protein A	0.825
PF02036	+	SCP-2 sterol transfer family	0.813
PF03502	+	Nucleoside-specific channel-forming protein, Tsx	0.804
PF13441	+	YMGG-like Gly-zipper	0.798
PF03942	+	DTW domain	0.788
PF09317	+	Domain of unknown function (DUF1974)	0.774
PF12857	+	TOBE-like domain	0.754
PF06067	+	Domain of unknown function (DUF932)	0.738
PF03091	+	CutA1 divalent ion tolerance protein	0.733
PF10636	+	Hemin uptake protein hemP	0.723
PF12769	+	Domain of unknown function (DUF3814)	0.706
PF03594	+	Benzoate membrane transport protein	0.703
PF02233	+	NAD(P) transhydrogenase beta subunit	0.671
PF08212	+	Lipocalin-like domain	0.671
PF00959	+	Phage lysozyme	0.669
PF08298	+	PrkA AAA domain	0.626
PF04325	+	Protein of unknown function (DUF465)	0.608
PF13332	+	Haemagluttinin repeat	0.559
PF12915	+	Protein of unknown function (DUF3833)	0.479
PF09413	+	Domain of unknown function (DUF2007)	0.463
PF06035	+	Bacterial transglutaminase-like cysteine proteinase BTLCP	0.455
PF04172	+	LrgB-like family	0.404
PF11391	+	Protein of unknown function (DUF2798)	0.332
PF13362	+	Toprim domain	0.331
PF04244	-	Deoxyribodipyrimidine photo-lyase-related protein	0.329
PF02706	+	Chain length determinant protein	0.318
PF09955	+	Predicted integral membrane protein (DUF2189)	0.314
PF00374	-	Nickel-dependent hydrogenase	0.305
PF01750	-	Hydrogenase maturation protease	0.305
PF11171	+	Protein of unknown function (DUF2958)	0.301
PF02230	-	Phospholipase/Carboxylesterase	0.299
PF01455	-	HupF/HypC family	0.296
PF12679	+	ABC-2 family transporter protein	0.267
PF14518	-	Iron-containing redox enzyme	0.249
PF04397	+	LytTr DNA-binding domain	0.227
PF14502	-	Helix-turn-helix domain	0.218
PF02583	+	Metal-sensitive transcriptional repressor	0.204
PF00685	-	Sulfotransferase domain	0.114

PF03681	-	Uncharacterised protein family (UPF0150)	0.106
PF09445	-	RNA cap guanine-N2 methyltransferase	0.081
PF13606	-	Ankyrin repeat	0.058
PF05378	-	Hydantoinase/oxoprolinase N-terminal region	0.039
PF00150	-	Cellulase (glycosyl hydrolase family 5)	0.033
PF02449	-	Beta-galactosidase	0.023
PF05014	+	Nucleoside 2-deoxyribosyltransferase	-0.012
PF02677	-	Uncharacterized BCR, COG1636	-0.037
PF13751	-	Transposase DDE domain	-0.052
PF01420	-	Type I restriction modification DNA specificity domain	-0.089
PF12705	-	PD-(D/E)XK nuclease superfamily	-0.108
PF02517	-	CAAX protease self-immunity	-0.148
PF04326	-	Divergent AAA domain	-0.188
PF02436	-	Conserved carboxylase domain	-0.205
PF00071	-	Ras family	-0.216
PF13395	-	HNH endonuclease	-0.228
PF02502	-	Ribose/Galactose Isomerase	-0.253
PF09587	-	Bacterial capsule synthesis protein PGA_cap	-0.259
PF13173	-	AAA domain	-0.446

Phenotype: Growth on ordinary blood agar (Growth), Predictor: phypat

Pfam	class	Pfam_desc	cor
PF01451	+	Low molecular weight phosphotyrosine protein phosphatase	0.480
PF00710	+	Asparaginase	0.435
PF01408	+	Oxidoreductase family, NAD-binding Rossmann fold	0.417
PF00781	+	Diacylglycerol kinase catalytic domain	0.405
PF04397	+	LytTr DNA-binding domain	0.396
PF05977	+	Transmembrane secretion effector	0.380
PF02706	+	Chain length determinant protein	0.372
PF04069	+	Substrate binding domain of ABC-type glycine betaine transport system	0.366
PF03601	+	Conserved hypothetical protein 698	0.366
PF06114	+	Domain of unknown function (DUF955)	0.332
PF07702	+	UTRA domain	0.326
PF01471	+	Putative peptidoglycan binding domain	0.323
PF06580	+	Histidine kinase	0.317
PF07085	+	DRTGG domain	0.279
PF05656	+	Protein of unknown function (DUF805)	0.266
PF02652	+	L-lactate permease	0.250
PF13247	+	4Fe-4S dicluster domain	0.245
PF04261	+	Dyp-type peroxidase family	0.243
PF07683	+	Cobalamin synthesis protein cobW C-terminal domain	0.241
PF03976	+	Polyphosphate kinase 2 (PPK2)	0.226
PF02589	+	Uncharacterised ACR, YkgG family COG1556	0.206
PF04186	+	FxsA cytoplasmic membrane protein	0.204
PF12840	-	Helix-turn-helix domain	0.197
PF01558	+	Pyruvate ferredoxin/flavodoxin oxidoreductase	0.191
PF05943	+	Protein of unknown function (DUF877)	0.158
PF09864	+	Membrane-bound lysozyme-inhibitor of c-type lysozyme	0.144

PF08019	+	Domain of unknown function (DUF1705)	0.110
PF00464	-	Serine hydroxymethyltransferase	0.061
PF13485	-	Peptidase MA superfamily	0.051
PF13492	-	GAF domain	0.035
PF01946	-	Thi4 family	0.016
PF02542	-	YgbB family	0.016
PF13392	-	HNH endonuclease	0.000
PF13371	-	Tetratricopeptide repeat	-0.019
PF13466	-	STAS domain	-0.021
PF00224	-	Pyruvate kinase, barrel domain	-0.025
PF07238	-	PilZ domain	-0.025
PF07927	-	YcfA-like protein	-0.032
PF00122	-	E1-E2 ATPase	-0.044
PF06831	-	Formamidopyrimidine-DNA glycosylase H2TH domain	-0.045
PF02224	-	Cytidylate kinase	-0.069
PF06723	-	MreB/Mbl protein	-0.069
PF05000	-	RNA polymerase Rpb1, domain 4	-0.075
PF00365	-	Phosphofructokinase	-0.076
PF04079	-	Putative transcriptional regulators (Ypuh-like)	-0.081
PF02616	-	ScpA/B protein	-0.086
PF02603	-	HPr Serine kinase N terminus	-0.089
PF05258	-	Protein of unknown function (DUF721)	-0.099
PF05191	-	Adenylate kinase, active site lid	-0.105
PF07916	-	TraG-like protein, N-terminal region	-0.106
PF06834	-	TraU protein	-0.111
PF03412	-	Peptidase C39 family	-0.112
PF13087	-	AAA domain	-0.116
PF08900	-	Domain of unknown function (DUF1845)	-0.118
PF00719	-	Inorganic pyrophosphatase	-0.138
PF13625	-	Helicase conserved C-terminal domain	-0.145
PF07602	-	Protein of unknown function (DUF1565)	-0.150
PF13482	-	RNase_H superfamily	-0.169
PF09836	-	Uncharacterized protein conserved in bacteria (DUF2063)	-0.169
PF00883	-	Cytosol aminopeptidase family, catalytic domain	-0.171
PF12762	-	ISXO2-like transposase domain	-0.195
PF11074	-	Domain of unknown function(DUF2779)	-0.541

Phenotype: Growth on ordinary blood agar (Growth), Predictor: phypat+PGL

Pfam	class	Pfam_desc	cor
PF01177	+	Asp/Glu/Hydantoin racemase	0.608
PF12704	+	MacB-like periplasmic core domain	0.573
PF03471	+	Transporter associated domain	0.541
PF01545	+	Cation efflux family	0.535
PF02620	+	Uncharacterized ACR, COG1399	0.496
PF00892	+	EamA-like transporter family	0.493
PF01451	+	Low molecular weight phosphotyrosine protein phosphatase	0.480
PF02787	+	Carbamoyl-phosphate synthetase large chain, oligomerisation domain	0.469
PF00924	+	Mechanosensitive ion channel	0.447

PF01113	+	Dihydrodipicolinate reductase, N-terminus	0.442
PF00710	+	Asparaginase	0.435
PF12698	+	ABC-2 family transporter protein	0.427
PF02576	+	Uncharacterised BCR, YhbC family COG0779	0.419
PF05173	+	Dihydrodipicolinate reductase, C-terminus	0.418
PF03668	+	P-loop ATPase protein family	0.414
PF01205	+	Uncharacterized protein family UPF0029	0.413
PF00781	+	Diacylglycerol kinase catalytic domain	0.405
PF13420	+	Acetyltransferase (GNAT) domain	0.382
PF05977	+	Transmembrane secretion effector	0.380
PF03601	+	Conserved hypothetical protein 698	0.366
PF06114	+	Domain of unknown function (DUF955)	0.332
PF04095	+	Nicotinate phosphoribosyltransferase (NAPRTase) family	0.324
PF01471	+	Putative peptidoglycan binding domain	0.323
PF13302	+	Acetyltransferase (GNAT) domain	0.302
PF02541	+	Ppx/GppA phosphatase family	0.301
PF12974	+	ABC transporter, phosphonate, periplasmic substrate-binding protein	0.293
PF00258	+	Flavodoxin	0.287
PF02806	+	Alpha amylase, C-terminal all-beta domain	0.266
PF13579	-	Glycosyl transferase 4-like domain	0.262
PF00984	+	UDP-glucose/GDP-mannose dehydrogenase family, central domain	0.257
PF01654	+	Bacterial Cytochrome Ubiquinol Oxidase	0.249
PF01136	+	Peptidase family U32	0.244
PF13275	+	S4 domain	0.242
PF01219	+	Prokaryotic diacylglycerol kinase	0.236
PF01507	+	Phosphoadenosine phosphosulfate reductase family	0.234
PF02634	+	FdhD/NarQ family	0.228
PF00708	+	Acylphosphatase	0.225
PF14698	-	Argininosuccinate lyase C-terminal	0.224
PF01583	+	Adenylylsulphate kinase	0.224
PF02602	+	Uroporphyrinogen-III synthase HemD	0.224
PF03780	+	Asp23 family	0.220
PF14490	+	Helix-hairpin-helix containing domain	0.216
PF02590	+	Predicted SPOUT methyltransferase	0.213
PF02589	+	Uncharacterised ACR, YkgG family COG1556	0.206
PF00475	-	Imidazoleglycerol-phosphate dehydratase	0.204
PF00815	-	Histidinol dehydrogenase	0.204
PF01634	-	ATP phosphoribosyltransferase	0.204
PF04186	+	FxsA cytoplasmic membrane protein	0.204
PF01022	-	Bacterial regulatory protein, arsR family	0.204
PF01745	+	Isopentenyl transferase	0.193
PF01558	+	Pyruvate ferredoxin/flavodoxin oxidoreductase	0.191
PF12730	+	ABC-2 family transporter protein	0.188
PF10369	-	Small subunit of acetolactate synthase	0.187
PF14534	+	Domain of unknown function (DUF4440)	0.186
PF00800	-	Prephenate dehydratase	0.186
PF03706	+	Uncharacterised protein family (UPF0104)	0.185
PF00195	+	Chalcone and stilbene synthases, N-terminal domain	0.185

PF01503	-	Phosphoribosyl-ATP pyrophosphohydrolase	0.169
PF08267	-	Cobalamin-independent synthase, N-terminal domain	0.164
PF02627	-	Carboxymuconolactone decarboxylase family	0.158
PF01794	+	Ferric reductase like transmembrane component	0.155
PF05954	+	Phage late control gene D protein (GPD)	0.144
PF09864	+	Membrane-bound lysozyme-inhibitor of c-type lysozyme	0.144
PF02678	+	Pirin	0.143
PF10410	-	DnaB-helicase binding domain of primase	0.140
PF02735	+	Ku70/Ku80 beta-barrel domain	0.136
PF04262	+	Glutamate-cysteine ligase	0.133
PF05853	+	Prokaryotic protein of unknown function (DUF849)	0.130
PF11734	+	TilS substrate C-terminal domain	0.129
PF13116	+	Protein of unknown function	0.128
PF02965	-	Vitamin B12 dependent methionine synthase, activation domain	0.126
PF03788	-	LrgA family	0.125
PF00657	-	GDSL-like Lipase/Acylhydrolase	0.125
PF05787	+	Bacterial protein of unknown function (DUF839)	0.122
PF11791	+	Aconitate B N-terminal domain	0.119
PF13593	-	SBF-like CPA transporter family (DUF4137)	0.116
PF08478	-	POTRA domain, FtsQ-type	0.113
PF04296	+	Protein of unknown function (DUF448)	0.112
PF01914	+	MarC family integral membrane protein	0.111
PF13519	+	von Willebrand factor type A domain	0.110
PF04325	+	Protein of unknown function (DUF465)	0.105
PF02436	-	Conserved carboxylase domain	0.104
PF06965	+	Na+/H+ antiporter 1	0.103
PF06742	+	Protein of unknown function (DUF1214)	0.101
PF02016	-	LD-carboxypeptidase	0.101
PF02371	-	Transposase IS116/IS110/IS902 family	0.101
PF08818	-	Domain of unknown function (DU1801)	0.098
PF04552	+	Sigma-54, DNA binding domain	0.095
PF01590	-	GAF domain	0.094
PF13714	-	Phosphoenolpyruvate phosphomutase	0.092
PF01841	-	Transglutaminase-like superfamily	0.091
PF12266	+	Protein of unknown function (DUF3613)	0.090
PF03358	-	NADPH-dependent FMN reductase	0.089
PF01980	-	Uncharacterised protein family UPF0066	0.088
PF00762	-	Ferrochelatase	0.085
PF09413	+	Domain of unknown function (DUF2007)	0.084
PF13578	-	Methyltransferase domain	0.084
PF01734	-	Patatin-like phospholipase	0.081
PF02782	-	FGGY family of carbohydrate kinases, C-terminal domain	0.079
PF00676	-	Dehydrogenase E1 component	0.079
PF06470	+	SMC proteins Flexible Hinge Domain	0.078
PF13623	+	SurA N-terminal domain	0.078
PF09019	+	EcoRII C terminal	0.077
PF05163	-	DinB family	0.076
PF01596	-	O-methyltransferase	0.076

PF10340	-	Protein of unknown function (DUF2424)	0.073
PF13360	-	PQQ-like domain	0.066
PF13704	-	Glycosyl transferase family 2	0.066
PF09848	-	Uncharacterized conserved protein (DUF2075)	0.066
PF03119	-	NAD-dependent DNA ligase C4 zinc finger domain	0.064
PF02645	+	Uncharacterised protein, DegV family COG1307	0.056
PF10097	+	Predicted membrane protein (DUF2335)	0.056
PF05049	+	Interferon-inducible GTPase (IIGP)	0.056
PF10544	+	T5orf172 domain	0.056
PF14384	+	Domain of unknown function (DUF4415)	0.053
PF01501	-	Glycosyl transferase family 8	0.050
PF13564	-	DoxX-like family	0.050
PF00116	-	Cytochrome C oxidase subunit II, periplasmic domain	0.048
PF13684	+	Dihydroxyacetone kinase family	0.041
PF05315	+	ICEA Protein	0.039
PF08402	-	TOBE domain	0.039
PF08386	-	TAP-like protein	0.037
PF13492	-	GAF domain	0.035
PF07714	-	Protein tyrosine kinase	0.029
PF06803	-	Protein of unknown function (DUF1232)	0.026
PF06827	-	Zinc finger found in FPG and IleRS	0.026
PF13248	-	zinc-ribbon domain	0.025
PF09445	-	RNA cap guanine-N2 methyltransferase	0.023
PF13550	-	Putative phage tail protein	0.022
PF10442	-	FIST C domain	0.017
PF01946	-	Thi4 family	0.016
PF14635	-	Helix-hairpin-helix motif	0.012
PF13174	-	Tetratricopeptide repeat	0.000
PF13392	-	HNH endonuclease	0.000
PF13490	-	Putative zinc-finger	-0.002
PF03681	-	Uncharacterised protein family (UPF0150)	-0.006
PF01555	-	DNA methylase	-0.007
PF13466	-	STAS domain	-0.021
PF02798	-	Glutathione S-transferase, N-terminal domain	-0.027
PF02592	-	Uncharacterized ACR, YhhQ family COG1738	-0.029
PF07927	-	YcfA-like protein	-0.032
PF10662	-	Ethanolamine utilisation - propanediol utilisation	-0.043
PF06831	-	Formamidopyrimidine-DNA glycosylase H2TH domain	-0.045
PF01523	-	Putative modulator of DNA gyrase	-0.061
PF02381	-	MraZ protein	-0.068
PF05258	-	Protein of unknown function (DUF721)	-0.099
PF09723	-	Zinc ribbon domain	-0.102
PF07916	-	TraG-like protein, N-terminal region	-0.106
PF13086	-	AAA domain	-0.112
PF03412	-	Peptidase C39 family	-0.112
PF11130	-	F pilus assembly Type-IV secretion system for plasmid transfer	-0.116
PF13087	-	AAA domain	-0.116
PF05050	-	Methyltransferase FkbM domain	-0.127

PF01633	-	Choline/ethanolamine kinase	-0.127
PF12762	-	ISXO2-like transposase domain	-0.195

Phenotype: Mucate utilization (Growth), Predictor: phypat

Pfam	class	Pfam_desc	cor
PF13557	+	Putative MetA-pathway of phenol degradation	0.795
PF00856	+	SET domain	0.736
PF01896	+	Eukaryotic and archaeal DNA primase small subunit	0.736
PF02586	+	Uncharacterised ACR, COG2135	0.692
PF07908	+	D-aminoacylase, C-terminal region	0.673
PF05138	+	Phenylacetic acid catabolic protein	0.673
PF14690	+	zinc-finger of transposase IS204/IS1001/IS1096/IS1165	0.654
PF03594	+	Benzoate membrane transport protein	0.652
PF04286	+	Protein of unknown function (DUF445)	0.620
PF06189	+	5'-nucleotidase	0.600
PF08386	+	TAP-like protein	0.597
PF05360	+	yiaA/B two helix domain	0.491
PF00221	+	Aromatic amino acid lyase	0.478
PF06416	+	Protein of unknown function (DUF1076)	0.450
PF13979	+	SopA-like catalytic domain	0.450
PF12834	+	Phage integrase, N-terminal	0.443
PF13750	+	Bacterial Ig-like domain (group 3)	0.423
PF06528	+	Phage P2 GpE	0.410
PF04536	-	TLP18.3, Psb32 and MOLO-1 founding proteins of phosphatase	-0.148
PF09669	-	Phage regulatory protein Rha (Phage_pRha)	-0.232
PF13704	-	Glycosyl transferase family 2	-0.243
PF02557	-	D-alanyl-D-alanine carboxypeptidase	-0.316
PF00195	-	Chalcone and stilbene synthases, N-terminal domain	-0.482
PF01226	-	Formate/nitrite transporter	-0.506
PF03205	-	Molybdopterin guanine dinucleotide synthesis protein B	-0.581
PF03222	-	Tryptophan/tyrosine permease family	-0.654
PF02086	-	D12 class N6 adenine-specific DNA methyltransferase	-0.669

Phenotype: Mucate utilization (Growth), Predictor: phypat+PGL

Pfam	class	Pfam_desc	cor
PF10017	+	Histidine-specific methyltransferase, SAM-dependent	0.736
PF02586	+	Uncharacterised ACR, COG2135	0.692
PF09290	+	Prokaryotic acetaldehyde dehydrogenase, dimerisation	0.654
PF14690	+	zinc-finger of transposase IS204/IS1001/IS1096/IS1165	0.654
PF06506	+	Propionate catabolism activator	0.652
PF01610	+	Transposase	0.652
PF05067	+	Manganese containing catalase	0.597
PF05717	+	IS66 Orf2 like protein	0.571
PF13007	+	Transposase C of IS166 homeodomain	0.571
PF01175	+	Urocanase	0.478
PF07804	+	HipA-like C-terminal domain	0.478
PF06353	+	Protein of unknown function (DUF1062)	0.450
PF10829	+	Protein of unknown function (DUF2554)	0.423

PF09857	+	Uncharacterized protein conserved in bacteria (DUF2084)	0.340
PF03437	+	BtpA family	0.333
PF10685	+	Stress-induced bacterial acidophilic repeat motif	0.298
PF03275	+	UDP-galactopyranose mutase	0.270
PF06902	+	Divergent 4Fe-4S mono-cluster	0.245
PF08085	-	Entericidin EcnA/B family	0.230
PF11682	+	Protein of unknown function (DUF3279)	0.175
PF10807	+	Protein of unknown function (DUF2541)	0.175
PF13610	-	DDE domain	0.116
PF03981	+	Ubiquinol-cytochrome C chaperone	0.114
PF02796	-	Helix-turn-helix domain of resolvase	0.068
PF01596	-	O-methyltransferase	0.068
PF03527	-	RHS protein	0.022
PF03235	-	Protein of unknown function DUF262	-0.022
PF01935	-	Domain of unknown function DUF87	-0.060
PF14864	-	Alkyl sulfatase C-terminal	-0.075
PF00150	-	Cellulase (glycosyl hydrolase family 5)	-0.116
PF03837	-	RecT family	-0.116
PF10617	-	Protein of unknown function (DUF2474)	-0.130
PF01156	-	Inosine-uridine preferring nucleoside hydrolase	-0.145
PF00092	-	von Willebrand factor type A domain	-0.147
PF07655	-	Secretin N-terminal domain	-0.195
PF09669	-	Phage regulatory protein Rha (Phage_pRha)	-0.232
PF02368	-	Bacterial Ig-like domain (group 2)	-0.248
PF03245	-	Bacteriophage Rz lysis protein	-0.285

Phenotype: Arginine dihydrolase (Growth: Amino acid), Predictor: phypat

Pfam	class	Pfam_desc	cor
PF02274	+	Amidinotransferase	0.492
PF03062	+	MBOAT, membrane-bound O-acyltransferase family	0.277
PF03577	+	Peptidase family C69	0.250
PF08928	+	Domain of unknown function (DUF1910)	0.246
PF04140	+	Isoprenylcysteine carboxyl methyltransferase (ICMT) family	0.226
PF10442	+	FIST C domain	0.218
PF01326	+	Pyruvate phosphate dikinase, PEP/pyruvate binding domain	0.215
PF07470	+	Glycosyl Hydrolase Family 88	0.207
PF05154	+	TM2 domain	0.205
PF10340	+	Protein of unknown function (DUF2424)	0.202
PF03618	+	Kinase/pyrophosphorylase	0.200
PF12392	+	Collagenase	0.194
PF13582	+	Metallo-peptidase family M12B Reprolysin-like	0.192
PF06568	+	Domain of unknown function (DUF1127)	0.164
PF02690	+	Na+/Pi-cotransporter	0.146
PF02810	+	SEC-C motif	0.136
PF03845	+	Spore germination protein	0.134
PF02834	+	LigT like Phosphoesterase	0.132
PF00565	+	Staphylococcal nuclease homologue	0.115
PF02368	+	Bacterial Ig-like domain (group 2)	0.104

PF03595	+	Voltage-dependent anion channel	0.102
PF01613	+	Flavin reductase like domain	0.081
PF05025	+	RbsD / FucU transport protein family	0.076
PF00662	+	NADH-Ubiquinone oxidoreductase (complex I), chain 5 N-terminus	0.072
PF00361	+	NADH-Ubiquinone/plastoquinone (complex I), various chains	0.060
PF04107	+	Glutamate-cysteine ligase family 2(GCS2)	0.040
PF00071	+	Ras family	0.031
PF00182	+	Chitinase class I	0.029
PF03275	-	UDP-galactopyranose mutase	-0.071
PF01633	-	Choline/ethanolamine kinase	-0.074
PF13362	-	Toprim domain	-0.135
PF01869	-	BadF/BadG/BcrA/BcrD ATPase family	-0.145
PF08378	-	Nuclease-related domain	-0.147
PF01555	-	DNA methylase	-0.154
PF05893	-	Acyl-CoA reductase (LuxC)	-0.169
PF03096	-	Ndr family	-0.177
PF04909	-	Amidohydrolase	-0.177
PF03417	-	Acyl-coenzyme A:6-aminopenicillanic acid acyl-transferase	-0.178
PF13723	-	Beta-ketoacyl synthase, N-terminal domain	-0.184
PF02737	-	3-hydroxyacyl-CoA dehydrogenase, NAD binding domain	-0.192
PF05191	-	Adenylate kinase, active site lid	-0.202
PF05148	-	Hypothetical methyltransferase	-0.204
PF05076	-	Suppressor of fused protein (SUFU)	-0.204
PF04014	-	Antidote-toxin recognition MazE	-0.204
PF02654	-	Cobalamin-5-phosphate synthase	-0.212
PF02550	-	Acetyl-CoA hydrolase/transferase N-terminal domain	-0.215
PF03711	-	Orn/Lys/Arg decarboxylase, C-terminal domain	-0.221
PF03616	-	Sodium/glutamate symporter	-0.223
PF04471	-	Restriction endonuclease	-0.230
PF03649	-	Uncharacterised protein family (UPF0014)	-0.230
PF12974	-	ABC transporter, phosphonate, periplasmic substrate-binding protein	-0.239
PF00022	-	Actin	-0.243
PF14552	-	Tautomerase enzyme	-0.248
PF04204	-	Homoserine O-succinyltransferase	-0.251
PF00023	-	Ankyrin repeat	-0.252
PF13857	-	Ankyrin repeats (many copies)	-0.252
PF02796	-	Helix-turn-helix domain of resolvase	-0.255
PF00267	-	Gram-negative porin	-0.258
PF02554	-	Carbon starvation protein CstA	-0.266
PF02371	-	Transposase IS116/IS110/IS902 family	-0.272
PF00591	-	Glycosyl transferase family, a/b domain	-0.277
PF00909	-	Ammonium Transporter Family	-0.287
PF01344	-	Kelch motif	-0.294
PF01208	-	Uroporphyrinogen decarboxylase (URO-D)	-0.300
PF07282	-	Putative transposase DNA-binding domain	-0.306
PF08267	-	Cobalamin-independent synthase, N-terminal domain	-0.312
PF07819	-	PGAP1-like protein	-0.327
PF14529	-	Endonuclease-reverse transcriptase	-0.328

PF04715	-	Anthranilate synthase component I, N terminal region	-0.404

Phenotype: Arginine dihydrolase (Growth: Amino acid), Predictor: phypat+PGL

Pfam	class	Pfam_desc	cor
PF02274	+	Amidinotransferase	0.492
PF03577	+	Peptidase family C69	0.250
PF13306	+	Leucine rich repeats (6 copies)	0.241
PF04140	+	Isoprenylcysteine carboxyl methyltransferase (ICMT) family	0.226
PF06715	+	Gp5 C-terminal repeat (3 copies)	0.216
PF01326	+	Pyruvate phosphate dikinase, PEP/pyruvate binding domain	0.215
PF01921	+	tRNA synthetases class I (K)	0.208
PF10340	+	Protein of unknown function (DUF2424)	0.202
PF13582	+	Metallo-peptidase family M12B Reprolysin-like	0.192
PF13574	+	Metallo-peptidase family M12B Reprolysin-like	0.192
PF12277	+	Protein of unknown function (DUF3618)	0.183
PF08020	+	Protein of unknown function (DUF1706)	0.154
PF04464	+	CDP-Glycerol:Poly(glycerophosphate) glycerophosphotransferase	0.154
PF10397	+	Adenylosuccinate lyase C-terminus	0.145
PF03845	+	Spore germination protein	0.134
PF13688	+	Metallo-peptidase family M12	0.130
PF00565	+	Staphylococcal nuclease homologue	0.115
PF14072	+	DNA-sulfur modification-associated	0.104
PF03606	+	C4-dicarboxylate anaerobic carrier	0.103
PF03595	+	Voltage-dependent anion channel	0.102
PF04131	+	Putative N-acetylmannosamine-6-phosphate epimerase	0.095
PF00176	+	SNF2 family N-terminal domain	0.087
PF00079	+	Serpin (serine protease inhibitor)	0.083
PF09360	+	Iron-binding zinc finger CDGSH type	0.082
PF01613	+	Flavin reductase like domain	0.081
PF13787	+	Protein of unknown function with HXXEE motif	0.077
PF05025	+	RbsD / FucU transport protein family	0.076
PF00150	+	Cellulase (glycosyl hydrolase family 5)	0.075
PF12229	+	Putative peptidoglycan binding domain	0.072
PF00662	+	NADH-Ubiquinone oxidoreductase (complex I), chain 5 N-terminus	0.072
PF00494	+	Squalene/phytoene synthase	0.071
PF03807	+	NADP oxidoreductase coenzyme F420-dependent	0.068
PF02733	+	Dak1 domain	0.068
PF05359	+	Domain of Unknown Function (DUF748)	0.067
PF04338	+	Protein of unknown function, DUF481	0.061
PF00361	+	NADH-Ubiquinone/plastoquinone (complex I), various chains	0.060
PF06888	+	Putative Phosphatase	0.057
PF00652	+	Ricin-type beta-trefoil lectin domain	0.048
PF10399	+	Ubiquitinol-cytochrome C reductase Fe-S subunit TAT signal	0.046
PF04107	+	Glutamate-cysteine ligase family 2(GCS2)	0.040
PF00011	+	Hsp20/alpha crystallin family	0.039
PF08218	-	Citrate lyase ligase C-terminal domain	0.033
PF07484	+	Phage Tail Collar Domain	0.031
PF08668	+	HDOD domain	0.023

PF13563	+	2'-5' RNA ligase superfamily	0.022
PF00801	+	PKD domain	0.022
PF02167	+	Cytochrome C1 family	0.017
PF00145	+	C-5 cytosine-specific DNA methylase	0.006
PF09411	+	Lipid A 3-O-deacylase (PagL)	0.006
PF00329	+	Respiratory-chain NADH dehydrogenase, 30 Kd subunit	0.001
PF02705	+	K+ potassium transporter	-0.008
PF05014	+	Nucleoside 2-deoxyribosyltransferase	-0.011
PF03412	-	Peptidase C39 family	-0.012
PF05962	+	HutD	-0.013
PF14305	+	TupA-like ATPgrasp	-0.025
PF07508	-	Recombinase	-0.027
PF01609	+	Transposase DDE domain	-0.033
PF13248	+	zinc-ribbon domain	-0.038
PF07523	+	Bacterial Ig-like domain (group 3)	-0.039
PF01863	+	Protein of unknown function DUF45	-0.048
PF00872	+	Transposase, Mutator family	-0.060
PF05724	+	Thiopurine S-methyltransferase (TPMT)	-0.061
PF00082	-	Subtilase family	-0.061
PF01306	+	LacY proton/sugar symporter	-0.070
PF10979	-	Protein of unknown function (DUF2786)	-0.087
PF13477	-	Glycosyl transferase 4-like	-0.087
PF13840	-	ACT domain	-0.098
PF00211	-	Adenylate and Guanylate cyclase catalytic domain	-0.100
PF04277	-	Oxaloacetate decarboxylase, gamma chain	-0.108
PF13483	-	Beta-lactamase superfamily domain	-0.112
PF13936	+	Helix-turn-helix domain	-0.114
PF01874	-	ATP:dephospho-CoA triphosphoribosyl transferase	-0.115
PF07005	+	Protein of unknown function, DUF1537	-0.121
PF13599	-	Pentapeptide repeats (9 copies)	-0.124
PF02275	-	Linear amide C-N hydrolases, choloylglycine hydrolase family	-0.127
PF02310	+	B12 binding domain	-0.138
PF01593	-	Flavin containing amine oxidoreductase	-0.139
PF13727	-	CoA-binding domain	-0.143
PF00903	-	Glyoxalase/Bleomycin resistance protein/Dioxygenase superfamily	-0.146
PF04493	-	Endonuclease V	-0.156
PF04230	-	Polysaccharide pyruvyl transferase	-0.156
PF02391	-	MoaE protein	-0.158
PF13278	-	Putative amidotransferase	-0.162
PF04266	-	ASCH domain	-0.167
PF03605	-	Anaerobic c4-dicarboxylate membrane transporter	-0.169
PF03096	-	Ndr family	-0.177
PF04909	-	Amidohydrolase	-0.177
PF06414	-	Zeta toxin	-0.181
PF01507	-	Phosphoadenosine phosphosulfate reductase family	-0.182
PF02230	-	Phospholipase/Carboxylesterase	-0.182
PF05704	-	Capsular polysaccharide synthesis protein	-0.182
PF06039	-	Malate:quinone oxidoreductase (Mqo)	-0.184

PF12821	-	Protein of unknown function (DUF3815)	-0.184
PF06738	-	Protein of unknown function (DUF1212)	-0.184
PF04608	-	Phosphatidylglycerophosphatase A	-0.189
PF02737	-	3-hydroxyacyl-CoA dehydrogenase, NAD binding domain	-0.192
PF01276	-	Orn/Lys/Arg decarboxylase, major domain	-0.201
PF04014	-	Antidote-toxin recognition MazE	-0.204
PF02550	-	Acetyl-CoA hydrolase/transferase N-terminal domain	-0.215
PF04471	-	Restriction endonuclease	-0.230
PF04655	-	Aminoglycoside/hydroxyurea antibiotic resistance kinase	-0.236
PF12974	-	ABC transporter, phosphonate, periplasmic substrate-binding protein	-0.239
PF13536	-	Multidrug resistance efflux transporter	-0.241
PF13580	-	SIS domain	-0.247
PF07511	-	Protein of unknown function (DUF1525)	-0.247
PF14552	-	Tautomerase enzyme	-0.248
PF04204	-	Homoserine O-succinyltransferase	-0.251
PF03775	-	Septum formation inhibitor MinC, C-terminal domain	-0.251
PF00591	-	Glycosyl transferase family, a/b domain	-0.277
PF01548	-	Transposase	-0.284
PF01208	-	Uroporphyrinogen decarboxylase (URO-D)	-0.300
PF07282	-	Putative transposase DNA-binding domain	-0.306
PF08267	-	Cobalamin-independent synthase, N-terminal domain	-0.312
PF14529	-	Endonuclease-reverse transcriptase	-0.328
PF04715	-	Anthranilate synthase component I, N terminal region	-0.404

Phenotype: Indole (Growth: Amino acid), Predictor: phypat

Pfam	class	Pfam_desc	cor
PF06519	+	TolA C-terminal	0.502
PF06368	+	Methylaspartate mutase E chain (MutE)	0.476
PF06291	+	Bor protein	0.476
PF03956	+	Membrane protein of unknown function (DUF340)	0.465
PF04338	+	Protein of unknown function, DUF481	0.454
PF14537	+	Cytochrome c3	0.431
PF11659	+	Protein of unknown function (DUF3261)	0.431
PF08029	+	HisG, C-terminal domain	0.428
PF02657	+	Fe-S metabolism associated domain	0.414
PF01184	+	GPR1/FUN34/yaaH family	0.398
PF08338	+	Domain of unknown function (DUF1731)	0.360
PF08433	+	Chromatin associated protein KTI12	0.360
PF07331	+	Tripartite tricarboxylate transporter TctB family	0.345
PF01970	+	Tripartite tricarboxylate transporter TctA family	0.337
PF02563	+	Polysaccharide biosynthesis/export protein	0.330
PF03724	+	META domain	0.327
PF13371	+	Tetratricopeptide repeat	0.315
PF01963	+	TraB family	0.307
PF03932	+	CutC family	0.304
PF11308	+	Glycosyl hydrolases related to GH101 family, GHL1-GHL3	0.302
PF03445	+	Putative nucleotidyltransferase DUF294	0.297
PF00491	+	Arginase family	0.289

PF02909	+	Tetracyclin repressor, C-terminal all-alpha domain	0.232
PF13936	+	Helix-turn-helix domain	0.225
PF07719	+	Tetratricopeptide repeat	0.225
PF02557	+	D-alanyl-D-alanine carboxypeptidase	0.225
PF05708	+	Orthopoxvirus protein of unknown function (DUF830)	0.203
PF04116	+	Fatty acid hydroxylase superfamily	0.196
PF07476	+	Methylaspartate ammonia-lyase C-terminus	0.194
PF02126	+	Phosphotriesterase family	0.192
PF01869	+	BadF/BadG/BcrA/BcrD ATPase family	0.179
PF05598	+	Transposase domain (DUF772)	0.165
PF13676	+	TIR domain	0.160
PF00939	-	Sodium:sulfate symporter transmembrane region	0.019
PF06559	-	2'-deoxycytidine 5'-triphosphate deaminase (DCD)	-0.012
PF13521	-	AAA domain	-0.016
PF01055	-	Glycosyl hydrolases family 31	-0.041
PF02677	-	Uncharacterized BCR, COG1636	-0.058
PF13377	-	Periplasmic binding protein-like domain	-0.059
PF06961	-	Protein of unknown function (DUF1294)	-0.070
PF01564	-	Spermine/spermidine synthase	-0.074
PF07927	-	YcfA-like protein	-0.079
PF05593	-	RHS Repeat	-0.083
PF02423	-	Ornithine cyclodeaminase/mu-crystallin family	-0.122
PF01289	-	Thiol-activated cytolysin	-0.126
PF05534	-	HicB family	-0.131
PF01850	-	PIN domain	-0.139
PF04239	-	Protein of unknown function (DUF421)	-0.146
PF03447	-	Homoserine dehydrogenase, NAD binding domain	-0.153
PF12738	-	twin BRCT domain	-0.160
PF03636	-	Glycosyl hydrolase family 65, N-terminal domain	-0.160
PF13470	-	PIN domain	-0.167
PF09851	-	Short C-terminal domain	-0.167
PF13229	-	Right handed beta helix region	-0.173
PF13473	-	Cupredoxin-like domain	-0.186
PF10503	-	Esterase PHB depolymerase	-0.186
PF05402	-	Coenzyme PQQ synthesis protein D (PqqD)	-0.186
PF01361	-	Tautomerase enzyme	-0.221
PF01244	-	Membrane dipeptidase (Peptidase family M19)	-0.227
PF05167	-	Uncharacterised ACR (DUF711)	-0.266
PF04079	-	Putative transcriptional regulators (Ypuh-like)	-0.361
PF02637	-	GatB domain	-0.448
PF02934	-	GatB/GatE catalytic domain	-0.448
PF02686	-	Glu-tRNAGln amidotransferase C subunit	-0.448

Phenotype: Indole (Growth: Amino acid), Predictor: phypat+PGL

Pfam	class	Pfam_desc	cor
PF06519	+	TolA C-terminal	0.502
PF06368	+	Methylaspartate mutase E chain (MutE)	0.476
PF04338	+	Protein of unknown function, DUF481	0.454

PF14537	+	Cytochrome c3	0.431
PF11659	+	Protein of unknown function (DUF3261)	0.431
PF08029	+	HisG, C-terminal domain	0.428
PF04187	+	Protein of unknown function, DUF399	0.426
PF01184	+	GPR1/FUN34/yaaH family	0.398
PF10554	+	Ash protein family	0.374
PF13808	+	DDE_Tnp_1-associated	0.373
PF04224	+	Protein of unknown function, DUF417	0.349
PF04170	+	NlpE N-terminal domain	0.345
PF02335	+	Cytochrome c552	0.343
PF08273	+	Zinc-binding domain of primase-helicase	0.331
PF00801	+	PKD domain	0.321
PF06992	+	Replication protein P	0.305
PF03932	+	CutC family	0.304
PF13583	+	Metallo-peptidase family M12B Reprolysin-like	0.302
PF11308	+	Glycosyl hydrolases related to GH101 family, GHL1-GHL3	0.302
PF00648	+	Calpain family cysteine protease	0.298
PF07675	+	Cleaved Adhesin Domain	0.298
PF03320	+	Bacterial fructose-1,6-bisphosphatase, glpX-encoded	0.298
PF13688	+	Metallo-peptidase family M12	0.287
PF02030	+	Hypothetical lipoprotein (MG045 family)	0.279
PF13574	+	Metallo-peptidase family M12B Reprolysin-like	0.273
PF00891	+	O-methyltransferase	0.262
PF04383	+	KilA-N domain	0.258
PF10509	+	Galactokinase galactose-binding signature	0.244
PF13148	+	Protein of unknown function (DUF3987)	0.237
PF03321	+	GH3 auxin-responsive promoter	0.227
PF02265	+	S1/P1 Nuclease	0.227
PF01983	+	Guanylyl transferase CofC like	0.225
PF13936	+	Helix-turn-helix domain	0.225
PF01316	+	Arginine repressor, DNA binding domain	0.219
PF06074	+	Protein of unknown function (DUF935)	0.215
PF14635	-	Helix-hairpin-helix motif	0.194
PF07476	+	Methylaspartate ammonia-lyase C-terminus	0.194
PF13728	+	F plasmid transfer operon protein	0.192
PF02744	+	Galactose-1-phosphate uridyl transferase, C-terminal domain	0.188
PF08463	+	EcoEI R protein C-terminal	0.187
PF05063	+	MT-A70	0.182
PF14305	+	TupA-like ATPgrasp	0.180
PF03186	+	CobD/Cbib protein	0.179
PF01869	+	BadF/BadG/BcrA/BcrD ATPase family	0.179
PF01609	+	Transposase DDE domain	0.166
PF02424	-	ApbE family	0.164
PF08020	+	Protein of unknown function (DUF1706)	0.163
PF14805	+	Tetrahydrodipicolinate N-succinyltransferase N-terminal	0.162
PF05090	+	Vitamin K-dependent gamma-carboxylase	0.151
PF02274	+	Amidinotransferase	0.146
PF03288	+	Poxvirus D5 protein-like	0.145

PF13310	+	Virulence protein RhuM family	0.143
PF14337	+	Domain of unknown function (DUF4393)	0.139
PF14351	+	Domain of unknown function (DUF4401)	0.137
PF14102	+	Capsule biosynthesis CapC	0.124
PF03616	-	Sodium/glutamate symporter	0.124
PF12680	+	SnoaL-like domain	0.123
PF13610	+	DDE domain	0.123
PF13569	+	Domain of unknown function (DUF4132)	0.118
PF14335	+	Domain of unknown function (DUF4391)	0.108
PF00331	+	Glycosyl hydrolase family 10	0.106
PF03352	+	Methyladenine glycosylase	0.102
PF03193	-	Protein of unknown function, DUF258	0.099
PF13247	-	4Fe-4S dicluster domain	0.092
PF14264	+	Glucosyl transferase GtrII	0.076
PF09439	+	Signal recognition particle receptor beta subunit	0.073
PF02469	+	Fasciclin domain	0.061
PF03486	-	HI0933-like protein	0.053
PF00022	-	Actin	0.049
PF05649	+	Peptidase family M13	0.048
PF01061	-	ABC-2 type transporter	0.046
PF00355	+	Rieske [2Fe-2S] domain	0.036
PF02659	-	Domain of unknown function DUF	0.033
PF01985	-	CRS1 / YhbY (CRM) domain	0.031
PF00939	-	Sodium:sulfate symporter transmembrane region	0.019
PF14821	-	Threonine synthase N terminus	0.007
PF10994	-	Protein of unknown function (DUF2817)	0.005
PF04657	-	Protein of unknown function, DUF606	-0.000
PF03006	-	Haemolysin-III related	-0.002
PF04093	-	rod shape-determining protein MreD	-0.005
PF02449	-	Beta-galactosidase	-0.012
PF13521	-	AAA domain	-0.016
PF08378	-	Nuclease-related domain	-0.024
PF08445	-	FR47-like protein	-0.029
PF13408	-	Recombinase zinc beta ribbon domain	-0.033
PF13563	-	2'-5' RNA ligase superfamily	-0.035
PF13385	-	Concanavalin A-like lectin/glucanases superfamily	-0.037
PF01035	-	6-O-methylguanine DNA methyltransferase, DNA binding domain	-0.043
PF01842	-	ACT domain	-0.061
PF06961	-	Protein of unknown function (DUF1294)	-0.070
PF05437	-	Branched-chain amino acid transport protein (AzlD)	-0.074
PF12766	+	Pyridoxamine 5'-phosphate oxidase	-0.074
PF04860	-	Phage portal protein	-0.077
PF13348	-	Tyrosine phosphatase family C-terminal region	-0.079
PF07927	-	YcfA-like protein	-0.079
PF05593	-	RHS Repeat	-0.083
PF10592	-	AIPR protein	-0.086
PF14698	-	Argininosuccinate lyase C-terminal	-0.086
PF05076	-	Suppressor of fused protein (SUFU)	-0.102

PF01958	-	Domain of unknown function DUF108	-0.110
PF00306	-	ATP synthase alpha/beta chain, C terminal domain	-0.129
PF01592	-	NifU-like N terminal domain	-0.147
PF03681	-	Uncharacterised protein family (UPF0150)	-0.157
PF03633	-	Glycosyl hydrolase family 65, C-terminal domain	-0.171
PF12395	-	Protein of unknown function	-0.175
PF13823	-	Alcohol dehydrogenase GroES-associated	-0.178
PF05816	-	Toxic anion resistance protein (TelA)	-0.200
PF02934	-	GatB/GatE catalytic domain	-0.448
PF02637	-	GatB domain	-0.448
PF07728	-	AAA domain (dynein-related subfamily)	

Phenotype: Lysine decarboxylase (Growth: Amino acid), Predictor: phypat

Pfam	class	Pfam_desc	cor
PF13265	+	Protein of unknown function (DUF4056)	0.514
PF09838	+	Uncharacterized protein conserved in bacteria (DUF2065)	0.453
PF06267	+	Family of unknown function (DUF1028)	0.450
PF03479	+	Domain of unknown function (DUF296)	0.404
PF05935	+	Arylsulfotransferase (ASST)	0.380
PF12792	+	CSS motif domain associated with EAL	0.377
PF13723	+	Beta-ketoacyl synthase, N-terminal domain	0.365
PF07449	+	Hydrogenase-1 expression protein HyaE	0.359
PF13402	+	Peptidase M60-like family	0.356
PF04655	+	Aminoglycoside/hydroxyurea antibiotic resistance kinase	0.344
PF06527	+	TniQ	0.331
PF01661	+	Macro domain	0.309
PF06674	+	Protein of unknown function (DUF1176)	0.294
PF00797	+	N-acetyltransferase	0.277
PF01385	+	Probable transposase	0.243
PF03239	-	Iron permease FTR1 family	-0.048
PF13473	-	Cupredoxin-like domain	-0.066
PF05861	-	Bacterial phosphonate metabolism protein (PhnI)	-0.106
PF06007	-	Phosphonate metabolism protein PhnJ	-0.106
PF05845	-	Bacterial phosphonate metabolism protein (PhnH)	-0.127
PF03374	-	Phage antirepressor protein KilAC domain	-0.148
PF01613	-	Flavin reductase like domain	-0.168
PF03681	-	Uncharacterised protein family (UPF0150)	-0.180
PF13646	-	HEAT repeats	-0.201
PF01804	-	Penicillin amidase	-0.218
PF04183	-	IucA / IucC family	-0.233
PF00707	-	Translation initiation factor IF-3, C-terminal domain	-0.236
PF01136	-	Peptidase family U32	-0.253
PF13555	-	P-loop containing region of AAA domain	-0.254
PF01174	-	SNO glutamine amidotransferase family	-0.272
PF02608	-	Basic membrane protein	-0.308
PF14595	-	Thioredoxin	-0.309
PF03062	-	MBOAT, membrane-bound O-acyltransferase family	-0.326
PF00565	-	Staphylococcal nuclease homologue	-0.453

Phenotype: Lysine decarboxylase (Growth: Amino acid), Predictor: phypat+PGL

Pfam	class	Pfam_desc	cor
PF13265	+	Protein of unknown function (DUF4056)	0.514
PF06006	+	Bacterial protein of unknown function (DUF905)	0.493
PF14557	+	Putative AphA-like transcriptional regulator	0.404
PF13737	+	Transposase DDE domain	0.404
PF07361	+	Cytochrome b562	0.393
PF05935	+	Arylsulfotransferase (ASST)	0.380
PF01894	+	Uncharacterised protein family UPF0047	0.377
PF06296	+	Protein of unknown function (DUF1044)	0.363
PF07449	+	Hydrogenase-1 expression protein HyaE	0.359
PF14367	+	Domain of unknown function (DUF4411)	0.359
PF13402	+	Peptidase M60-like family	0.356
PF06205	+	Glycosyltransferase 36 associated family	0.356
PF06165	+	Glycosyltransferase family 36	0.356
PF04655	+	Aminoglycoside/hydroxyurea antibiotic resistance kinase	0.344
PF10137	+	Predicted nucleotide-binding protein containing TIR-like domain	0.344
PF01483	+	Proprotein convertase P-domain	0.344
PF02001	+	Protein of unknown function DUF134	0.342
PF12158	+	Protein of unknown function (DUF3592)	0.331
PF00728	+	Glycosyl hydrolase family 20, catalytic domain	0.329
PF04383	+	KilA-N domain	0.314
PF11659	+	Protein of unknown function (DUF3261)	0.314
PF11459	+	Protein of unknwon function (DUF2893)	0.309
PF13711	+	Domain of unknown function (DUF4160)	0.309
PF09485	+	CRISPR-associated protein Cse2 (CRISPR_cse2)	0.279
PF08706	+	D5 N terminal like	0.276
PF09374	+	Predicted Peptidoglycan domain	0.276
PF13276	+	HTH-like domain	0.272
PF14883	+	Hypothetical glycosyl hydrolase family 13	0.259
PF06528	+	Phage P2 GpE	0.242
PF00782	+	Dual specificity phosphatase, catalytic domain	0.236
PF07603	+	Protein of unknown function (DUF1566)	0.236
PF11230	+	Protein of unknown function (DUF3029)	0.228
PF01169	+	Uncharacterized protein family UPF0016	0.227
PF07282	+	Putative transposase DNA-binding domain	0.227
PF13565	+	Homeodomain-like domain	0.222
PF09314	+	Domain of unknown function (DUF1972)	0.222
PF07394	+	Protein of unknown function (DUF1501)	0.206
PF05117	+	Family of unknown function (DUF695)	0.193
PF02450	+	Lecithin:cholesterol acyltransferase	0.168
PF01420	+	Type I restriction modification DNA specificity domain	0.159
PF13665	+	Domain of unknown function (DUF4150)	0.158
PF14531	+	Kinase-like	0.154
PF01865	+	Protein of unknown function DUF47	0.148
PF01970	+	Tripartite tricarboxylate transporter TctA family	0.148
PF02486	+	Replication initiation factor	0.146

PF00891	+	O-methyltransferase	0.130
PF05015	+	Plasmid maintenance system killer protein	0.130
PF09537	+	Domain of unknown function (DUF2383)	0.099
PF05893	+	Acyl-CoA reductase (LuxC)	0.066
PF06892	+	Phage regulatory protein CII (CP76)	0.057
PF02275	+	Linear amide C-N hydrolases, choloylglycine hydrolase family	0.048
PF02475	+	Met-10+ like-protein	0.046
PF12900	+	Pyridoxamine 5'-phosphate oxidase	0.043
PF05816	+	Toxic anion resistance protein (TelA)	0.029
PF03625	+	Domain of unknown function DUF302	0.022
PF06042	-	Bacterial protein of unknown function (DUF925)	-0.034
PF01850	-	PIN domain	-0.038
PF11870	-	Domain of unknown function (DUF3390)	-0.047
PF06530	-	Phage antitermination protein Q	-0.060
PF11008	-	Protein of unknown function (DUF2846)	-0.068
PF13241	-	Putative NAD(P)-binding	-0.102
PF14301	-	Domain of unknown function (DUF4376)	-0.105
PF13264	-	Domain of unknown function (DUF4055)	-0.105
PF10099	-	Anti-sigma-K factor rskA	-0.116
PF02589	-	Uncharacterised ACR, YkgG family COG1556	-0.137
PF03497	-	Anthrax toxin LF subunit	-0.175
PF03681	-	Uncharacterised protein family (UPF0150)	-0.180
PF07849	-	Protein of unknown function (DUF1641)	-0.211
PF11391	-	Protein of unknown function (DUF2798)	-0.228
PF02386	-	Cation transport protein	-0.228
PF00707	-	Translation initiation factor IF-3, C-terminal domain	-0.236
PF08486	-	Stage II sporulation protein	-0.246
PF01174	-	SNO glutamine amidotransferase family	-0.272
PF02655	-	ATP-grasp domain	-0.280
PF00565	-	Staphylococcal nuclease homologue	-0.453

Phenotype: Ornithine decarboxylase (Growth: Amino acid), Predictor: phypat

Pfam	class	Pfam_desc	cor
PF03701	+	Uncharacterised protein family (UPF0181)	0.605
PF10625	+	Universal stress protein B (UspB)	0.600
PF13718	+	GNAT acetyltransferase 2	0.597
PF06496	+	Protein of unknown function (DUF1097)	0.560
PF12431	+	Transcriptional regulator	0.496
PF02901	+	Pyruvate formate lyase	0.492
PF04492	+	Bacteriophage replication protein O	0.490
PF02030	+	Hypothetical lipoprotein (MG045 family)	0.490
PF04993	+	TfoX N-terminal domain	0.482
PF00061	+	Lipocalin / cytosolic fatty-acid binding protein family	0.461
PF03709	+	Orn/Lys/Arg decarboxylase, N-terminal domain	0.447
PF04391	+	Protein of unknown function (DUF533)	0.416
PF09831	+	Uncharacterized protein conserved in bacteria (DUF2058)	0.368
PF12320	+	Type 5 capsule protein repressor C-terminal domain	0.334
PF09374	+	Predicted Peptidoglycan domain	0.325

PF06130	+	Propanediol utilisation protein PduL	0.307
PF03475	+	3-alpha domain	0.268
PF02502	+	Ribose/Galactose Isomerase	0.268
PF03773	+	Predicted permease	0.244
PF02567	+	Phenazine biosynthesis-like protein	0.190
PF00797	+	N-acetyltransferase	0.160
PF00023	+	Ankyrin repeat	0.089
PF13289	+	SIR2-like domain	0.008
PF03288	-	Poxvirus D5 protein-like	-0.003
PF01094	-	Receptor family ligand binding region	-0.057
PF07732	-	Multicopper oxidase	-0.057
PF00690	-	Cation transporter/ATPase, N-terminus	-0.084
PF02445	-	Quinolinate synthetase A protein	-0.120
PF01613	-	Flavin reductase like domain	-0.152
PF13551	-	Winged helix-turn helix	-0.160
PF00848	-	Ring hydroxylating alpha subunit (catalytic domain)	-0.163
PF00092	-	von Willebrand factor type A domain	-0.229
PF13086	-	AAA domain	-0.232
PF04011	-	LemA family	-0.232
PF01609	-	Transposase DDE domain	-0.243
PF04326	-	Divergent AAA domain	-0.272
PF13340	-	Putative transposase of IS4/5 family (DUF4096)	-0.293
PF01321	-	Creatinase/Prolidase N-terminal domain	-0.294
PF04115	-	Ureidoglycolate hydrolase	-0.300
PF00908	-	dTDP-4-dehydrorhamnose 3,5-epimerase	-0.323
PF05221	-	S-adenosyl-L-homocysteine hydrolase	-0.355
PF14552	-	Tautomerase enzyme	-0.410
PF12867	-	DinB superfamily	-0.435
PF01960	-	ArgJ family	-0.492
PF00142	-	4Fe-4S iron sulfur cluster binding proteins, NifH/frxC family	-0.520
PF00670	-	S-adenosyl-L-homocysteine hydrolase, NAD binding domain	-0.520

Phenotype: Ornithine decarboxylase (Growth: Amino acid), Predictor: phypat+PGL

Pfam	class	Pfam_desc	cor
PF10022	+	Uncharacterized protein conserved in bacteria (DUF2264)	0.507
PF04131	+	Putative N-acetylmannosamine-6-phosphate epimerase	0.505
PF04492	+	Bacteriophage replication protein O	0.490
PF02030	+	Hypothetical lipoprotein (MG045 family)	0.490
PF07450	+	Formate hydrogenlyase maturation protein HycH	0.468
PF13729	+	F plasmid transfer operon, TraF, protein	0.455
PF03709	+	Orn/Lys/Arg decarboxylase, N-terminal domain	0.447
PF02288	+	Dehydratase medium subunit	0.384
PF02287	+	Dehydratase small subunit	0.384
PF02286	+	Dehydratase large subunit	0.384
PF12568	-	Acetyltransferase (GNAT) domain	0.369
PF09831	+	Uncharacterized protein conserved in bacteria (DUF2058)	0.368
PF03612	+	Sorbitol phosphotransferase enzyme II N-terminus	0.336
PF09374	+	Predicted Peptidoglycan domain	0.325

PF03829	+	PTS system glucitol/sorbitol-specific IIA component	0.315
PF02110	+	Hydroxyethylthiazole kinase family	0.306
PF05063	+	MT-A70	0.305
PF05971	-	Protein of unknown function (DUF890)	0.291
PF14331	+	ImcF-related N-terminal domain	0.282
PF01614	+	Bacterial transcriptional regulator	0.263
PF10423	-	Bacterial AMP nucleoside phosphorylase N-terminus	0.257
PF09313	+	Domain of unknown function (DUF1971)	0.255
PF11319	+	Protein of unknown function (DUF3121)	0.229
PF12224	+	Putative amidoligase enzyme	0.218
PF13461	+	Cell-wall surface anchor repeat	0.218
PF06141	+	Phage minor tail protein U	0.209
PF01483	+	Proprotein convertase P-domain	0.209
PF07278	+	Protein of unknown function (DUF1441)	0.205
PF13591	+	MerR HTH family regulatory protein	0.191
PF11862	-	Domain of unknown function (DUF3382)	0.188
PF05195	-	Aminopeptidase P, N-terminal domain	0.179
PF13186	+	Iron-sulfur cluster-binding domain	0.175
PF01337	-	Barstar (barnase inhibitor)	0.172
PF08361	-	MAATS-type transcriptional repressor, C-terminal region	0.172
PF10994	+	Protein of unknown function (DUF2817)	0.172
PF08878	+	Domain of unknown function (DUF1837)	0.170
PF06029	-	AlkA N-terminal domain	0.163
PF06719	-	AraC-type transcriptional regulator N-terminus	0.160
PF05345	+	Putative Ig domain	0.156
PF07308	-	Protein of unknown function (DUF1456)	0.154
PF14310	-	Fibronectin type III-like domain	0.150
PF06224	-	Winged helix DNA-binding domain	0.141
PF10124	+	Mu-like prophage major head subunit gpT	0.139
PF09392	-	Type III secretion needle MxiH like	0.136
PF00781	-	Diacylglycerol kinase catalytic domain	0.128
PF07769	-	psiF repeat	0.119
PF00182	-	Chitinase class I	0.119
PF13977	-	Bacterial transcriptional repressor	0.114
PF13088	+	BNR repeat-like domain	0.111
PF01915	-	Glycosyl hydrolase family 3 C-terminal domain	0.101
PF06945	-	Protein of unknown function (DUF1289)	0.101
PF13440	+	Polysaccharide biosynthesis protein	0.093
PF13806	-	Rieske-like [2Fe-2S] domain	0.093
PF12949	+	HeH/LEM domain	0.090
PF00023	+	Ankyrin repeat	0.089
PF01019	-	Gamma-glutamyltranspeptidase	0.086
PF03831	-	PhnA protein	0.080
PF02028	-	BCCT family transporter	0.073
PF05962	-	HutD	0.066
PF13577	+	SnoaL-like domain	0.061
PF05593	+	RHS Repeat	0.054
PF06754	-	Phosphonate metabolism protein PhnG	0.051

PF07511	+	Protein of unknown function (DUF1525)	0.048
PF00484	-	Carbonic anhydrase	0.045
PF06007	-	Phosphonate metabolism protein PhnJ	0.037
PF05861	-	Bacterial phosphonate metabolism protein (PhnI)	0.037
PF00576	-	HIUase/Transthyretin family	0.031
PF03575	-	Peptidase family S51	0.030
PF03092	+	BT1 family	0.027
PF00782	-	Dual specificity phosphatase, catalytic domain	0.020
PF01633	-	Choline/ethanolamine kinase	0.018
PF02366	-	Dolichyl-phosphate-mannose-protein mannosyltransferase	0.018
PF00302	-	Chloramphenicol acetyltransferase	0.011
PF05658	-	Head domain of trimeric autotransporter adhesin	0.009
PF00037	-	4Fe-4S binding domain	0.001
PF01058	-	NADH ubiquinone oxidoreductase, 20 Kd subunit	-0.023
PF05232	-	Bacterial Transmembrane Pair family	-0.029
PF00941	-	FAD binding domain in molybdopterin dehydrogenase	-0.029
PF01799	-	[2Fe-2S] binding domain	-0.029
PF05857	-	TraX protein	-0.030
PF03308	-	ArgK protein	-0.032
PF14488	-	Domain of unknown function (DUF4434)	-0.036
PF11041	-	Protein of unknown function (DUF2612)	-0.036
PF00723	+	Glycosyl hydrolases family 15	-0.060
PF13429	+	Tetratricopeptide repeat	-0.066
PF00689	-	Cation transporting ATPase, C-terminus	-0.070
PF13396	+	Phospholipase_D-nuclease N-terminal	-0.077
PF04773	+	FecR protein	-0.078
PF00545	-	ribonuclease	-0.081
PF00690	-	Cation transporter/ATPase, N-terminus	-0.084
PF13087	-	AAA domain	-0.114
PF02445	-	Quinolinate synthetase A protein	-0.120
PF06042	-	Bacterial protein of unknown function (DUF925)	-0.122
PF04471	-	Restriction endonuclease	-0.124
PF05899	-	Protein of unknown function (DUF861)	-0.137
PF05985	-	Ethanolamine ammonia-lyase light chain (EutC)	-0.140
PF13358	-	DDE superfamily endonuclease	-0.142
PF00296	-	Luciferase-like monooxygenase	-0.153
PF13551	-	Winged helix-turn helix	-0.160
PF05426	-	Alginate lyase	-0.165
PF03412	-	Peptidase C39 family	-0.172
PF03171	-	2OG-Fe(II) oxygenase superfamily	-0.172
PF06751	-	Ethanolamine ammonia lyase large subunit (EutB)	-0.179
PF09290	+	Prokaryotic acetaldehyde dehydrogenase, dimerisation	-0.180
PF07719	+	Tetratricopeptide repeat	-0.185
PF11744	-	Aluminium activated malate transporter	-0.190
PF07685	+	CobB/CobQ-like glutamine amidotransferase domain	-0.206
PF03703	+	Bacterial PH domain	-0.212
PF00035	-	Double-stranded RNA binding motif	-0.218
PF09364	+	XFP N-terminal domain	-0.225

PF13539	-	D-alanyl-D-alanine carboxypeptidase	-0.252
PF14595	+	Thioredoxin	-0.291
PF01321	-	Creatinase/Prolidase N-terminal domain	-0.294
PF06821	-	Serine hydrolase	-0.314
PF01425	-	Amidase	-0.315
PF12867	-	DinB superfamily	-0.435

Phenotype: Acetate utilization (Growth: Carboxylic acid), Predictor: phypat

Pfam	class	Pfam_desc	cor
PF04444	+	Catechol dioxygenase N terminus	0.704
PF13503	+	Domain of unknown function (DUF4123)	0.647
PF02694	+	Uncharacterised BCR, YnfA/UPF0060 family	0.638
PF00190	+	Cupin	0.613
PF06779	+	Protein of unknown function (DUF1228)	0.597
PF02274	+	Amidinotransferase	0.574
PF04828	+	Glutathione-dependent formaldehyde-activating enzyme	0.552
PF07739	+	TipAS antibiotic-recognition domain	0.551
PF00221	+	Aromatic amino acid lyase	0.547
PF03413	+	Peptidase propeptide and YPEB domain	0.486
PF13563	+	2'-5' RNA ligase superfamily	0.461
PF08818	+	Domain of unknown function (DU1801)	0.459
PF04229	+	GrpB protein	0.428
PF08378	+	Nuclease-related domain	0.427
PF13362	+	Toprim domain	0.405
PF06029	+	AlkA N-terminal domain	0.405
PF07331	+	Tripartite tricarboxylate transporter TctB family	0.361
PF03472	+	Autoinducer binding domain	0.358
PF09346	+	SMI1 / KNR4 family (SUKH-1)	0.335
PF11340	+	Protein of unknown function (DUF3142)	0.330
PF00350	+	Dynamin family	0.244
PF11153	+	Protein of unknown function (DUF2931)	0.228
PF00545	-	ribonuclease	-0.066
PF07411	-	Domain of unknown function (DUF1508)	-0.142
PF13676	-	TIR domain	-0.170
PF04371	-	Porphyromonas-type peptidyl-arginine deiminase	-0.193
PF01548	-	Transposase	-0.242
PF13591	-	MerR HTH family regulatory protein	-0.244
PF02361	-	Cobalt transport protein	-0.258
PF01769	-	Divalent cation transporter	-0.273
PF13310	-	Virulence protein RhuM family	-0.307
PF03448	-	MgtE intracellular N domain	-0.345
PF01112	-	Asparaginase	-0.346
PF06965	-	Na+/H+ antiporter 1	-0.395
PF02677	-	Uncharacterized BCR, COG1636	-0.407
PF02253	-	Phospholipase A1	-0.469
PF01633	-	Choline/ethanolamine kinase	-0.500
PF01443	-	Viral (Superfamily 1) RNA helicase	-0.501
PF13698	-	Domain of unknown function (DUF4156)	-0.503

PF03738	-	Glutathionylspermidine synthase preATP-grasp	-0.538
PF11119	-	Protein of unknown function (DUF2633)	-0.565
PF02952	-	L-fucose isomerase, C-terminal domain	-0.588

Phenotype: Acetate utilization (Growth: Carboxylic acid), Predictor: phypat+PGL

Pfam	class	Pfam_desc	cor
PF13503	+	Domain of unknown function (DUF4123)	0.647
PF02694	+	Uncharacterised BCR, YnfA/UPF0060 family	0.638
PF00190	+	Cupin	0.613
PF06779	+	Protein of unknown function (DUF1228)	0.597
PF02274	+	Amidinotransferase	0.574
PF13577	+	SnoaL-like domain	0.561
PF00221	+	Aromatic amino acid lyase	0.547
PF14535	+	AMP-binding enzyme C-terminal domain	0.524
PF01175	+	Urocanase	0.500
PF04140	+	Isoprenylcysteine carboxyl methyltransferase (ICMT) family	0.492
PF07719	+	Tetratricopeptide repeat	0.486
PF03060	+	Nitronate monooxygenase	0.461
PF13563	+	2'-5' RNA ligase superfamily	0.461
PF02133	+	Permease for cytosine/purines, uracil, thiamine, allantoin	0.454
PF13362	+	Toprim domain	0.405
PF01970	+	Tripartite tricarboxylate transporter TctA family	0.397
PF05544	+	Proline racemase	0.368
PF07331	+	Tripartite tricarboxylate transporter TctB family	0.361
PF03472	+	Autoinducer binding domain	0.358
PF04492	+	Bacteriophage replication protein O	0.325
PF00848	+	Ring hydroxylating alpha subunit (catalytic domain)	0.323
PF03350	+	Uncharacterized protein family, UPF0114	0.309
PF02517	+	CAAX protease self-immunity	0.309
PF00932	+	Lamin Tail Domain	0.302
PF00350	+	Dynamin family	0.244
PF07582	+	AP endonuclease family 2 C terminus	0.232
PF01011	+	PQQ enzyme repeat	0.222
PF08722	-	TnsA endonuclease N terminal	0.207
PF05199	-	GMC oxidoreductase	0.205
PF02796	+	Helix-turn-helix domain of resolvase	0.193
PF05426	-	Alginate lyase	0.185
PF10029	-	Predicted periplasmic protein (DUF2271)	0.160
PF03235	+	Protein of unknown function DUF262	0.148
PF07055	-	Enoyl reductase FAD binding domain	0.148
PF00908	-	dTDP-4-dehydrorhamnose 3,5-epimerase	0.129
PF11086	-	Protein of unknown function (DUF2878)	0.056
PF07508	-	Recombinase	0.044
PF10670	-	Domain of unknown function (DUF4198)	0.036
PF03524	-	Conjugal transfer protein	-0.008
PF04313	-	Type I restriction enzyme R protein N terminus (HSDR_N)	-0.018
PF07484	-	Phage Tail Collar Domain	-0.036
PF13499	-	EF-hand domain pair	-0.068

PF00480	-	ROK family	-0.075
PF08719	-	Domain of unknown function (DUF1768)	-0.076
PF13087	-	AAA domain	-0.076
PF06293	-	Lipopolysaccharide kinase (Kdo/WaaP) family	-0.093
PF00468	-	Ribosomal protein L34	-0.102
PF08544	-	GHMP kinases C terminal	-0.110
PF08843	-	Nucleotidyl transferase of unknown function (DUF1814)	-0.129
PF13676	-	TIR domain	-0.170
PF00041	-	Fibronectin type III domain	-0.170
PF03547	-	Membrane transport protein	-0.194
PF01548	-	Transposase	-0.242
PF00245	-	Alkaline phosphatase	-0.257
PF02361	-	Cobalt transport protein	-0.258
PF03681	-	Uncharacterised protein family (UPF0150)	-0.272
PF01769	-	Divalent cation transporter	-0.273
PF03806	-	AbgT putative transporter family	-0.306
PF03448	-	MgtE intracellular N domain	-0.345
PF01112	-	Asparaginase	-0.346
PF14537	-	Cytochrome c3	-0.358
PF02502	-	Ribose/Galactose Isomerase	-0.361
PF01633	-	Choline/ethanolamine kinase	-0.500
PF13698	-	Domain of unknown function (DUF4156)	-0.503
PF03738	-	Glutathionylspermidine synthase preATP-grasp	-0.538

Phenotype: Citrate (Growth: Carboxylic acid), Predictor: phypat

Pfam	class	Pfam_desc	cor
PF02449	+	Beta-galactosidase	0.606
PF08532	+	Beta-galactosidase trimerisation domain	0.527
PF04616	+	Glycosyl hydrolases family 43	0.517
PF02903	+	Alpha amylase, N-terminal ig-like domain	0.493
PF12558	+	ATP-binding cassette cobalt transporter	0.471
PF07470	+	Glycosyl Hydrolase Family 88	0.470
PF00135	+	Carboxylesterase family	0.458
PF03390	+	2-hydroxycarboxylate transporter family	0.457
PF01915	+	Glycosyl hydrolase family 3 C-terminal domain	0.439
PF06134	+	L-rhamnose isomerase (RhaA)	0.438
PF14310	+	Fibronectin type III-like domain	0.414
PF03829	+	PTS system glucitol/sorbitol-specific IIA component	0.409
PF02230	+	Phospholipase/Carboxylesterase	0.403
PF07748	+	Glycosyl hydrolases family 38 C-terminal domain	0.375
PF02016	+	LD-carboxypeptidase	0.364
PF04295	+	D-galactarate dehydratase / Altronate hydrolase, C terminus	0.351
PF02588	+	Uncharacterized BCR, YitT family COG1284	0.339
PF00722	+	Glycosyl hydrolases family 16	0.306
PF05035	+	2-keto-3-deoxy-galactonokinase	0.289
PF12708	+	Pectate lyase superfamily protein	0.288
PF06204	+	Putative carbohydrate binding domain	0.267
PF04984	+	Phage tail sheath protein	0.240

PF08212	+	Lipocalin-like domain	0.212
PF01869	+	BadF/BadG/BcrA/BcrD ATPase family	0.210
PF10397	+	Adenylosuccinate lyase C-terminus	0.204
PF14031	+	Putative serine dehydratase domain	0.204
PF05014	-	Nucleoside 2-deoxyribosyltransferase	0.027
PF00282	-	Pyridoxal-dependent decarboxylase conserved domain	-0.056
PF13495	-	Phage integrase, N-terminal SAM-like domain	-0.064
PF10417	-	C-terminal domain of 1-Cys peroxiredoxin	-0.073
PF01633	-	Choline/ethanolamine kinase	-0.079
PF00872	-	Transposase, Mutator family	-0.088
PF02498	-	BRO family, N-terminal domain	-0.127
PF09924	-	Uncharacterized conserved protein (DUF2156)	-0.163
PF03824	-	High-affinity nickel-transport protein	-0.171
PF13086	-	AAA domain	-0.182
PF02388	-	FemAB family	-0.184
PF10415	-	Fumarase C C-terminus	-0.194
PF03308	-	ArgK protein	-0.206
PF01637	-	Archaeal ATPase	-0.209
PF02541	-	Ppx/GppA phosphatase family	-0.217
PF00745	-	Glutamyl-tRNAGlu reductase, dimerisation domain	-0.235
PF02597	-	ThiS family	-0.271
PF00883	-	Cytosol aminopeptidase family, catalytic domain	-0.288
PF12838	-	4Fe-4S dicluster domain	-0.306
PF00209	-	Sodium:neurotransmitter symporter family	-0.339

Phenotype: Citrate (Growth: Carboxylic acid), Predictor: phypat+PGL

Pfam	class	Pfam_desc	cor
PF02449	+	Beta-galactosidase	0.606
PF04616	+	Glycosyl hydrolases family 43	0.517
PF05870	+	Phenolic acid decarboxylase (PAD)	0.500
PF02903	+	Alpha amylase, N-terminal ig-like domain	0.493
PF12558	+	ATP-binding cassette cobalt transporter	0.471
PF07470	+	Glycosyl Hydrolase Family 88	0.470
PF00135	+	Carboxylesterase family	0.458
PF03390	+	2-hydroxycarboxylate transporter family	0.457
PF06161	+	Protein of unknown function (DUF975)	0.452
PF06134	+	L-rhamnose isomerase (RhaA)	0.438
PF11975	+	Family 4 glycosyl hydrolase C-terminal domain	0.427
PF00232	+	Glycosyl hydrolase family 1	0.420
PF12464	+	Maltose acetyltransferase	0.410
PF02230	+	Phospholipase/Carboxylesterase	0.403
PF00150	+	Cellulase (glycosyl hydrolase family 5)	0.402
PF00251	+	Glycosyl hydrolases family 32 N-terminal domain	0.380
PF06833	+	Malonate decarboxylase gamma subunit (MdcE)	0.369
PF06964	+	Alpha-L-arabinofuranosidase C-terminus	0.369
PF02016	+	LD-carboxypeptidase	0.364
PF03812	+	2-keto-3-deoxygluconate permease	0.360
PF04295	+	D-galactarate dehydratase / Altronate hydrolase, C terminus	0.351

PF12535	+	Hydrolase of X-linked nucleoside diphosphate N terminal	0.341
PF00781	+	Diacylglycerol kinase catalytic domain	0.336
PF03935	+	Beta-glucan synthesis-associated protein (SKN1)	0.323
PF03786	+	D-mannonate dehydratase (UxuA)	0.312
PF00722	+	Glycosyl hydrolases family 16	0.306
PF05229	+	Spore Coat Protein U domain	0.302
PF02829	+	3H domain	0.297
PF12708	+	Pectate lyase superfamily protein	0.288
PF00520	+	Ion transport protein	0.288
PF01548	+	Transposase	0.276
PF02746	+	Mandelate racemase / muconate lactonizing enzyme, N-terminal domain	0.276
PF06204	+	Putative carbohydrate binding domain	0.267
PF06165	+	Glycosyltransferase family 36	0.267
PF03632	+	Glycosyl hydrolase family 65 central catalytic domain	0.263
PF01204	+	Trehalase	0.249
PF07221	+	N-acylglucosamine 2-epimerase (GlcNAc 2-epimerase)	0.244
PF04984	+	Phage tail sheath protein	0.240
PF06149	+	Protein of unknown function (DUF969)	0.240
PF06568	+	Domain of unknown function (DUF1127)	0.237
PF02156	+	Glycosyl hydrolase family 26	0.236
PF03328	+	HpcH/HpaI aldolase/citrate lyase family	0.232
PF08323	+	Starch synthase catalytic domain	0.231
PF01094	+	Receptor family ligand binding region	0.222
PF05913	+	Bacterial protein of unknown function (DUF871)	0.221
PF08376	+	Nitrate and nitrite sensing	0.217
PF08212	+	Lipocalin-like domain	0.212
PF01869	+	BadF/BadG/BcrA/BcrD ATPase family	0.210
PF10397	+	Adenylosuccinate lyase C-terminus	0.204
PF03425	+	Carbohydrate binding domain (family 11)	0.201
PF07714	+	Protein tyrosine kinase	0.200
PF06082	+	Bacterial putative lipoprotein (DUF940)	0.197
PF13434	+	L-lysine 6-monooxygenase (NADPH-requiring)	0.193
PF00359	+	Phosphoenolpyruvate-dependent sugar phosphotransferase system, EIIA 2	0.185
PF03306	-	Alpha-acetolactate decarboxylase	0.181
PF11208	-	Protein of unknown function (DUF2992)	0.161
PF03733	-	Domain of unknown function (DUF307)	0.158
PF00962	+	Adenosine/AMP deaminase	0.154
PF02225	-	PA domain	0.147
PF09346	+	SMI1 / KNR4 family (SUKH-1)	0.147
PF05954	+	Phage late control gene D protein (GPD)	0.130
PF07949	-	YbbR-like protein	0.130
PF13395	-	HNH endonuclease	0.129
PF00381	-	PTS HPr component phosphorylation site	0.126
PF08495	+	FIST N domain	0.124
PF01345	-	Domain of unknown function DUF11	0.118
PF13240	-	zinc-ribbon domain	0.116
PF00710	+	Asparaginase	0.113
PF00302	-	Chloramphenicol acetyltransferase	0.112

PF09851	+	Short C-terminal domain	0.112
PF08843	-	Nucleotidyl transferase of unknown function (DUF1814)	0.100
PF04203	-	Sortase family	0.099
PF13768	+	von Willebrand factor type A domain	0.099
PF01223	-	DNA/RNA non-specific endonuclease	0.098
PF13542	-	Helix-turn-helix domain of transposase family ISL3	0.097
PF13378	+	Enolase C-terminal domain-like	0.083
PF08378	-	Nuclease-related domain	0.082
PF00198	+	2-oxoacid dehydrogenases acyltransferase (catalytic domain)	0.070
PF02452	-	PemK-like protein	0.068
PF09709	+	CRISPR-associated protein (Cas_Csd1)	0.064
PF05119	-	Phage terminase, small subunit	0.059
PF07685	-	CobB/CobQ-like glutamine amidotransferase domain	0.058
PF00665	-	Integrase core domain	0.058
PF07282	-	Putative transposase DNA-binding domain	0.057
PF01188	+	Mandelate racemase / muconate lactonizing enzyme, C-terminal domain	0.054
PF01523	+	Putative modulator of DNA gyrase	0.049
PF05544	-	Proline racemase	0.048
PF09250	-	Bifunctional DNA primase/polymerase, N-terminal	0.048
PF13988	-	Protein of unknown function (DUF4225)	0.045
PF07831	-	Pyrimidine nucleoside phosphorylase C-terminal domain	0.045
PF13243	+	Prenyltransferase-like	0.044
PF10145	-	Phage-related minor tail protein	0.041
PF12821	-	Protein of unknown function (DUF3815)	0.038
PF07853	-	Protein of unknown function (DUF1648)	0.038
PF13474	-	SnoaL-like domain	0.038
PF13426	-	PAS domain	0.035
PF12051	-	Protein of unknown function (DUF3533)	0.031
PF00805	-	Pentapeptide repeats (8 copies)	0.027
PF07664	-	Ferrous iron transport protein B C terminus	0.026
PF06983	+	3-demethylubiquinone-9 3-methyltransferase	0.018
PF09134	+	Invasin, domain 3	0.016
PF14690	-	zinc-finger of transposase IS204/IS1001/IS1096/IS1165	0.007
PF06605	-	Prophage endopeptidase tail	0.007
PF04332	-	Protein of unknown function (DUF475)	0.003
PF03174	-	Chitobiase/beta-hexosaminidase C-terminal domain	-0.002
PF01867	-	CRISPR associated protein Cas1	-0.008
PF13372	+	Alginate export	-0.009
PF06808	-	DctM-like transporters	-0.009
PF11794	-	4-hydroxyphenylacetate 3-hydroxylase N terminal	-0.016
PF13415	+	Galactose oxidase, central domain	-0.020
PF04011	-	LemA family	-0.028
PF02535	-	ZIP Zinc transporter	-0.034
PF13456	-	Reverse transcriptase-like	-0.038
PF07501	-	G5 domain	-0.040
PF07729	-	FCD domain	-0.041
PF02557	-	D-alanyl-D-alanine carboxypeptidase	-0.041
PF01773	-	Na+ dependent nucleoside transporter N-terminus	-0.045

PF07662	-	Na+ dependent nucleoside transporter C-terminus	-0.045
PF13624	+	SurA N-terminal domain	-0.049
PF00931	-	NB-ARC domain	-0.050
PF00282	-	Pyridoxal-dependent decarboxylase conserved domain	-0.056
PF07274	-	Protein of unknown function (DUF1440)	-0.057
PF01855	-	Pyruvate flavodoxin/ferredoxin oxidoreductase, thiamine diP-bdg	-0.058
PF13338	-	Domain of unknown function (DUF4095)	-0.070
PF08000	-	Bacterial PH domain	-0.071
PF02436	+	Conserved carboxylase domain	-0.073
PF01633	-	Choline/ethanolamine kinase	-0.079
PF03741	-	Integral membrane protein TerC family	-0.082
PF06889	+	Protein of unknown function (DUF1266)	-0.088
PF01740	-	STAS domain	-0.100
PF03575	-	Peptidase family S51	-0.105
PF00221	-	Aromatic amino acid lyase	-0.117
PF03806	-	AbgT putative transporter family	-0.127
PF01520	-	N-acetylmuramoyl-L-alanine amidase	-0.130
PF13241	-	Putative NAD(P)-binding	-0.130
PF07536	-	HWE histidine kinase	-0.131
PF04134	-	Protein of unknown function, DUF393	-0.138
PF03445	-	Putative nucleotidyltransferase DUF294	-0.139
PF13398	-	Peptidase M50B-like	-0.141
PF00850	-	Histone deacetylase domain	-0.146
PF01551	-	Peptidase family M23	-0.147
PF00762	-	Ferrochelatase	-0.168
PF01663	-	Type I phosphodiesterase / nucleotide pyrophosphatase	-0.171
PF01208	-	Uroporphyrinogen decarboxylase (URO-D)	-0.181
PF02754	-	Cysteine-rich domain	-0.182
PF00565	-	Staphylococcal nuclease homologue	-0.183
PF06411	+	HdeA/HdeB family	-0.197
PF01637	-	Archaeal ATPase	-0.209
PF01755	-	Glycosyltransferase family 25 (LPS biosynthesis protein)	-0.233
PF02597	-	ThiS family	-0.271

Phenotype: Malonate (Growth: Carboxylic acid), Predictor: phypat

Pfam	class	Pfam_desc	cor
PF06833	+	Malonate decarboxylase gamma subunit (MdcE)	0.749
PF10620	+	Phosphoribosyl-dephospho-CoA transferase MdcG	0.696
PF05402	+	Coenzyme PQQ synthesis protein D (PqqD)	0.550
PF11453	+	Protein of unknown function (DUF2950)	0.550
PF06906	+	Protein of unknown function (DUF1272)	0.525
PF13723	+	Beta-ketoacyl synthase, N-terminal domain	0.423
PF04284	+	Protein of unknown function (DUF441)	0.416
PF04233	+	Phage Mu protein F like protein	0.392
PF07358	+	Protein of unknown function (DUF1482)	0.370
PF10397	+	Adenylosuccinate lyase C-terminus	0.362
PF02588	+	Uncharacterized BCR, YitT family COG1284	0.311
PF03306	+	Alpha-acetolactate decarboxylase	0.290

PF12343	+	Cold shock protein DEAD box A	0.263
PF05866	+	Endodeoxyribonuclease RusA	0.231
PF02361	+	Cobalt transport protein	0.208
PF13596	+	PAS domain	0.135
PF04586	-	Caudovirus prohead protease	-0.100
PF00282	-	Pyridoxal-dependent decarboxylase conserved domain	-0.217
PF06048	-	Domain of unknown function (DUF927)	-0.274
PF03350	-	Uncharacterized protein family, UPF0114	-0.290
PF08238	-	Sel1 repeat	-0.330
PF00355	-	Rieske [2Fe-2S] domain	-0.339
PF00350	-	Dynamin family	-0.343
PF01227	-	GTP cyclohydrolase I	-0.355
PF09107	-	Elongation factor SelB, winged helix	-0.373
PF00092	-	von Willebrand factor type A domain	-0.411
PF13356	-	Domain of unknown function (DUF4102)	-0.444
PF01311	-	Bacterial export proteins, family 1	-0.614
PF01312	-	FlhB HrpN YscU SpaS Family	-0.614
PF01313	-	Bacterial export proteins, family 3	-0.614
PF00771	-	FHIPEP family	-0.614
PF00669	-	Bacterial flagellin N-terminal helical region	-0.614
PF03963	-	Flagellar hook capping protein - N-terminal region	-0.614
PF02107	-	Flagellar L-ring protein	-0.614
PF02119	-	Flagellar P-ring protein	-0.614
PF06429	-	Flagellar basal body rod FlgEFG protein C-terminal	-0.614
PF00460	-	Flagella basal body rod protein	-0.614
PF00813	-	FliP family	-0.614
PF01052	-	Surface presentation of antigens (SPOA)	-0.614
PF01514	-	Secretory protein of YscJ/FliF family	-0.614

Phenotype: Malonate (Growth: Carboxylic acid), Predictor: phypat+PGL

Pfam	class	Pfam_desc	cor
PF06833	+	Malonate decarboxylase gamma subunit (MdcE)	0.749
PF09995	+	Uncharacterized protein conserved in bacteria (DUF2236)	0.607
PF06906	+	Protein of unknown function (DUF1272)	0.525
PF14493	+	Helix-turn-helix domain	0.462
PF07582	+	AP endonuclease family 2 C terminus	0.420
PF04284	+	Protein of unknown function (DUF441)	0.416
PF06993	+	Protein of unknown function (DUF1304)	0.373
PF06772	+	Bacterial low temperature requirement A protein (LtrA)	0.368
PF10397	+	Adenylosuccinate lyase C-terminus	0.362
PF02588	+	Uncharacterized BCR, YitT family COG1284	0.311
PF04404	+	ERF superfamily	0.275
PF12343	+	Cold shock protein DEAD box A	0.263
PF09364	+	XFP N-terminal domain	0.250
PF09907	+	Uncharacterized protein conserved in bacteria (DUF2136)	0.248
PF05866	+	Endodeoxyribonuclease RusA	0.231
PF00932	+	Lamin Tail Domain	0.158
PF02028	+	BCCT family transporter	0.102

PF13350	+	Tyrosine phosphatase family	0.086
PF03918	+	Cytochrome C biogenesis protein	0.037
PF13637	-	Ankyrin repeats (many copies)	-0.006
PF03435	-	Saccharopine dehydrogenase	-0.101
PF04606	-	Ogr/Delta-like zinc finger	-0.118
PF03230	-	Antirestriction protein	-0.160
PF02413	-	Caudovirales tail fibre assembly protein	-0.199
PF05932	-	Tir chaperone protein (CesT) family	-0.226
PF05016	-	Plasmid stabilisation system protein	-0.252
PF06048	-	Domain of unknown function (DUF927)	-0.274
PF01522	-	Polysaccharide deacetylase	-0.275
PF13231	-	Dolichyl-phosphate-mannose-protein mannosyltransferase	-0.392
PF13356	-	Domain of unknown function (DUF4102)	-0.444

Phenotype: Tartrate utilization (Growth: Carboxylic acid), Predictor: phypat

Pfam	class	Pfam_desc	cor
n/a	n/a	n/a	n/a

Phenotype: Tartrate utilization (Growth: Carboxylic acid), Predictor: phypat+PGL

Pfam	class	Pfam_desc	cor
PF07237	+	Protein of unknown function (DUF1428)	0.626
PF08908	+	Domain of unknown function (DUF1852)	0.602
PF02253	+	Phospholipase A1	0.600
PF01055	+	Glycosyl hydrolases family 31	0.596
PF11659	+	Protein of unknown function (DUF3261)	0.559
PF03649	+	Uncharacterised protein family (UPF0014)	0.544
PF13286	+	Phosphohydrolase-associated domain	0.544
PF10994	+	Protein of unknown function (DUF2817)	0.533
PF03230	+	Antirestriction protein	0.519
PF04227	+	Indigoidine synthase A like protein	0.519
PF12910	+	Antitoxin of toxin-antitoxin stability system N-terminal	0.519
PF00295	+	Glycosyl hydrolases family 28	0.465
PF06966	+	Protein of unknown function (DUF1295)	0.465
PF01112	+	Asparaginase	0.447
PF13454	+	FAD-NAD(P)-binding	0.447
PF01144	+	Coenzyme A transferase	0.447
PF01223	+	DNA/RNA non-specific endonuclease	0.409
PF13591	+	MerR HTH family regulatory protein	0.378
PF03994	+	Domain of Unknown Function (DUF350)	0.316
PF04014	+	Antidote-toxin recognition MazE	0.316
PF00023	+	Ankyrin repeat	0.294
PF01968	+	Hydantoinase/oxoprolinase	0.224
PF02894	-	Oxidoreductase family, C-terminal alpha/beta domain	-0.042
PF10340	-	Protein of unknown function (DUF2424)	-0.053
PF03783	-	Curli production assembly/transport component CsgG	-0.053
PF03824	-	High-affinity nickel-transport protein	-0.067
PF13517	+	Repeat domain in Vibrio, Colwellia, Bradyrhizobium and Shewanella	-0.086
PF00239	-	Resolvase, N terminal domain	-0.086

PF03681	-	Uncharacterised protein family (UPF0150)	-0.089
PF05069	-	Phage virion morphogenesis family	-0.142
PF13753	-	Putative flagellar system-associated repeat	-0.194
PF03079	-	ARD/ARD' family	-0.194
PF07520	-	Virulence factor SrfB	-0.208
PF00195	-	Chalcone and stilbene synthases, N-terminal domain	-0.222
PF13578	-	Methyltransferase domain	-0.222
PF13687	-	Domain of unknown function (DUF4153)	-0.244
PF13823	-	Alcohol dehydrogenase GroES-associated	-0.246
PF13246	-	Putative hydrolase of sodium-potassium ATPase alpha subunit	-0.258
PF13086	-	AAA domain	-0.298
PF05643	-	Putative bacterial lipoprotein (DUF799)	-0.306
PF04860	-	Phage portal protein	-0.378
PF07885	-	Ion channel	-0.431

Phenotype: Gas from glucose (Growth: Glucose), Predictor: phypat

Pfam	class	Pfam_desc	cor
PF04616	+	Glycosyl hydrolases family 43	0.650
PF07450	+	Formate hydrogenlyase maturation protein HycH	0.645
PF11659	+	Protein of unknown function (DUF3261)	0.579
PF12917	+	HD containing hydrolase-like enzyme	0.564
PF00445	+	Ribonuclease T2 family	0.559
PF01948	+	Aspartate carbamoyltransferase regulatory chain, allosteric domain	0.544
PF01915	+	Glycosyl hydrolase family 3 C-terminal domain	0.532
PF13723	+	Beta-ketoacyl synthase, N-terminal domain	0.525
PF13753	+	Putative flagellar system-associated repeat	0.509
PF05128	+	Domain of unknown function (DUF697)	0.499
PF11941	+	Domain of unknown function (DUF3459)	0.498
PF02508	+	Rnf-Nqr subunit, membrane protein	0.479
PF11756	+	Nitrous oxide-stimulated promoter	0.442
PF13556	+	PucR C-terminal helix-turn-helix domain	0.421
PF03830	+	PTS system sorbose subfamily IIB component	0.395
PF12392	+	Collagenase	0.394
PF04230	+	Polysaccharide pyruvyl transferase	0.387
PF13391	+	HNH endonuclease	0.365
PF00367	+	phosphotransferase system, EIIB	0.365
PF02133	+	Permease for cytosine/purines, uracil, thiamine, allantoin	0.349
PF07331	+	Tripartite tricarboxylate transporter TctB family	0.336
PF07833	+	Copper amine oxidase N-terminal domain	0.292
PF13567	+	Domain of unknown function (DUF4131)	0.250
PF08415	+	Nonribosomal peptide synthase	0.246
PF02746	-	Mandelate racemase / muconate lactonizing enzyme, N-terminal domain	0.179
PF06838	+	Methionine gamma-lyase	0.139
PF08643	+	Fungal family of unknown function (DUF1776)	0.139
PF07555	+	beta-N-acetylglucosaminidase	0.138
PF04474	-	Protein of unknown function (DUF554)	0.131
PF04138	+	GtrA-like protein	0.131
PF13379	-	NMT1-like family	0.123

PF05016	-	Plasmid stabilisation system protein	0.117
PF13378	-	Enolase C-terminal domain-like	0.103
PF04198	-	Putative sugar-binding domain	0.097
PF04439	+	Streptomycin adenylyltransferase	0.067
PF02350	-	UDP-N-acetylglucosamine 2-epimerase	0.061
PF02810	-	SEC-C motif	0.041
PF13483	-	Beta-lactamase superfamily domain	0.040
PF03458	-	UPF0126 domain	0.032
PF03119	-	NAD-dependent DNA ligase C4 zinc finger domain	0.004
PF00288	-	GHMP kinases N terminal domain	0.003
PF02785	-	Biotin carboxylase C-terminal domain	-0.008
PF13103	-	TonB C terminal	-0.018
PF13854	-	Kelch motif	-0.032
PF00209	+	Sodium:neurotransmitter symporter family	-0.040
PF13392	-	HNH endonuclease	-0.068
PF06769	-	Plasmid encoded toxin Txe	-0.072
PF03308	-	ArgK protein	-0.076
PF00355	-	Rieske [2Fe-2S] domain	-0.084
PF04452	-	RNA methyltransferase	-0.095
PF12822	-	Protein of unknown function (DUF3816)	-0.096
PF12895	-	Anaphase-promoting complex, cyclosome, subunit 3	-0.098
PF01610	-	Transposase	-0.132
PF13519	-	von Willebrand factor type A domain	-0.134
PF02498	-	BRO family, N-terminal domain	-0.149
PF00188	-	Cysteine-rich secretory protein family	-0.167
PF13672	-	Protein phosphatase 2C	-0.177
PF13359	-	DDE superfamily endonuclease	-0.178
PF00754	-	F5/8 type C domain	-0.197
PF01878	-	EVE domain	-0.213
PF00025	-	ADP-ribosylation factor family	-0.269
PF00071	-	Ras family	-0.270
PF01790	-	Prolipoprotein diacylglyceryl transferase	-0.277
PF01252	-	Signal peptidase (SPase) II	-0.277
PF01921	-	tRNA synthetases class I (K)	-0.279
PF01809	-	Haemolytic domain	-0.292
PF01745	-	Isopentenyl transferase	-0.304
PF14693	-	Ribosomal protein TL5, C-terminal domain	-0.352
PF02632	-	BioY family	-0.361
PF07521	-	RNA-metabolising metallo-beta-lactamase	-0.440
PF02637	-	GatB domain	-0.486
PF02934	-	GatB/GatE catalytic domain	-0.486

Phenotype: Gas from glucose (Growth: Glucose), Predictor: phypat+PGL

Pfam	class	Pfam_desc	cor
PF04616	+	Glycosyl hydrolases family 43	0.650
PF08525	+	Opacity-associated protein A N-terminal motif	0.594
PF09994	+	Uncharacterized alpha/beta hydrolase domain (DUF2235)	0.570
PF10678	+	Protein of unknown function (DUF2492)	0.567

PF12917	+	HD containing hydrolase-like enzyme	0.564
PF07273	+	Protein of unknown function (DUF1439)	0.560
PF00445	+	Ribonuclease T2 family	0.559
PF06992	+	Replication protein P	0.504
PF13503	+	Domain of unknown function (DUF4123)	0.478
PF05651	+	Putative sugar diacid recognition	0.474
PF00959	+	Phage lysozyme	0.442
PF11756	+	Nitrous oxide-stimulated promoter	0.442
PF02369	+	Bacterial Ig-like domain (group 1)	0.437
PF10022	+	Uncharacterized protein conserved in bacteria (DUF2264)	0.437
PF12408	+	Ribose-5-phosphate isomerase	0.434
PF03802	+	Apo-citrate lyase phosphoribosyl-dephospho-CoA transferase	0.429
PF04492	+	Bacteriophage replication protein O	0.422
PF03450	+	CO dehydrogenase flavoprotein C-terminal domain	0.418
PF10065	+	Uncharacterized conserved protein (DUF2303)	0.408
PF03830	+	PTS system sorbose subfamily IIB component	0.395
PF03609	+	PTS system sorbose-specific iic component	0.395
PF10437	+	Bacterial lipoate protein ligase C-terminus	0.368
PF06296	+	Protein of unknown function (DUF1044)	0.349
PF09938	+	Uncharacterized protein conserved in bacteria (DUF2170)	0.340
PF03795	+	YCII-related domain	0.331
PF14378	+	PAP2 superfamily	0.322
PF03390	+	2-hydroxycarboxylate transporter family	0.314
PF08681	+	Protein of unknown function (DUF1778)	0.301
PF07833	+	Copper amine oxidase N-terminal domain	0.292
PF04463	+	Protein of unknown function (DUF523)	0.282
PF02955	-	Prokaryotic glutathione synthetase, ATP-grasp domain	0.270
PF01914	-	MarC family integral membrane protein	0.263
PF03853	+	YjeF-related protein N-terminus	0.257
PF13567	+	Domain of unknown function (DUF4131)	0.250
PF08415	+	Nonribosomal peptide synthase	0.246
PF04131	+	Putative N-acetylmannosamine-6-phosphate epimerase	0.236
PF14393	+	Domain of unknown function (DUF4422)	0.233
PF14497	-	Glutathione S-transferase, C-terminal domain	0.211
PF03972	+	MmgE/PrpD family	0.211
PF00463	-	Isocitrate lyase family	0.209
PF00102	+	Protein-tyrosine phosphatase	0.209
PF05263	+	Protein of unknown function (DUF722)	0.203
PF09375	-	Imelysin	0.202
PF06821	+	Serine hydrolase	0.188
PF09223	-	YodA lipocalin-like domain	0.184
PF08020	+	Protein of unknown function (DUF1706)	0.177
PF10101	+	Predicted membrane protein (DUF2339)	0.160
PF02646	-	RmuC family	0.159
PF06430	+	Lactococcus lactis RepB C-terminus	0.156
PF12801	+	4Fe-4S binding domain	0.148
PF08643	+	Fungal family of unknown function (DUF1776)	0.139
PF07555	+	beta-N-acetylglucosaminidase	0.138

PF08274	-	PhnA Zinc-Ribbon	0.123
PF00544	+	Pectate lyase	0.122
PF14238	+	Domain of unknown function (DUF4340)	0.122
PF11794	-	4-hydroxyphenylacetate 3-hydroxylase N terminal	0.122
PF09508	+	Lacto-N-biose phosphorylase	0.118
PF12708	-	Pectate lyase superfamily protein	0.110
PF13443	-	Cro/C1-type HTH DNA-binding domain	0.105
PF08751	-	TrwC relaxase	0.101
PF04198	-	Putative sugar-binding domain	0.097
PF13384	-	Homeodomain-like domain	0.094
PF13380	-	CoA binding domain	0.091
PF03096	+	Ndr family	0.090
PF09681	+	N-terminal phage replisome organiser (Phage_rep_org_N)	0.078
PF03577	+	Peptidase family C69	0.072
PF14535	+	AMP-binding enzyme C-terminal domain	0.056
PF01116	-	Fructose-bisphosphate aldolase class-II	0.037
PF01163	-	RIO1 family	0.024
PF07216	+	LcrG protein	0.015
PF09025	+	YopR Core	0.015
PF03400	-	IS1 transposase	0.012
PF00908	-	dTDP-4-dehydrorhamnose 3,5-epimerase	0.005
PF04221	-	RelB antitoxin	0.004
PF13103	-	TonB C terminal	-0.018
PF00670	+	S-adenosyl-L-homocysteine hydrolase, NAD binding domain	-0.026
PF05690	-	Thiazole biosynthesis protein ThiG	-0.032
PF13749	-	ATP-dependent DNA helicase recG C-terminal	-0.055
PF12010	+	Domain of unknown function (DUF3502)	-0.056
PF06769	-	Plasmid encoded toxin Txe	-0.072
PF07508	-	Recombinase	-0.075
PF00515	-	Tetratricopeptide repeat	-0.075
PF08708	-	Primase C terminal 1 (PriCT-1)	-0.079
PF00355	-	Rieske [2Fe-2S] domain	-0.084
PF12822	-	Protein of unknown function (DUF3816)	-0.096
PF05219	-	DREV methyltransferase	-0.098
PF09848	-	Uncharacterized conserved protein (DUF2075)	-0.115
PF05154	-	TM2 domain	-0.119
PF13011	-	leucine-zipper of insertion element IS481	-0.125
PF03435	-	Saccharopine dehydrogenase	-0.129
PF01610	-	Transposase	-0.132
PF13519	-	von Willebrand factor type A domain	-0.134
PF10592	-	AIPR protein	-0.139
PF14096	-	Domain of unknown function (DUF4274)	-0.140
PF14716	-	Helix-hairpin-helix domain	-0.147
PF02498	-	BRO family, N-terminal domain	-0.149
PF00368	-	Hydroxymethylglutaryl-coenzyme A reductase	-0.160
PF03444	+	Winged helix-turn-helix transcription repressor, HrcA DNA-binding	-0.161
PF13857	-	Ankyrin repeats (many copies)	-0.173
PF13672	-	Protein phosphatase 2C	-0.177

PF04011	-	LemA family	-0.185
PF05258	-	Protein of unknown function (DUF721)	-0.200
PF01878	-	EVE domain	-0.213
PF00071	-	Ras family	-0.270

Phenotype: Glucose fermenter (Growth: Glucose), Predictor: phypat

Pfam	class	Pfam_desc	cor
PF00874	+	PRD domain	0.699
PF01238	+	Phosphomannose isomerase type I	0.697
PF05116	+	Sucrose-6F-phosphate phosphohydrolase	0.657
PF00265	+	Thymidine kinase	0.636
PF10509	+	Galactokinase galactose-binding signature	0.631
PF00358	+	phosphoenolpyruvate-dependent sugar phosphotransferase system, EIIA 1	0.630
PF03611	+	PTS system sugar-specific permease component	0.626
PF03830	+	PTS system sorbose subfamily IIB component	0.605
PF00232	+	Glycosyl hydrolase family 1	0.603
PF03123	+	CAT RNA binding domain	0.600
PF02744	+	Galactose-1-phosphate uridyl transferase, C-terminal domain	0.597
PF12464	+	Maltose acetyltransferase	0.593
PF00251	+	Glycosyl hydrolases family 32 N-terminal domain	0.588
PF03306	+	Alpha-acetolactate decarboxylase	0.545
PF02929	+	Beta galactosidase small chain	0.524
PF00128	+	Alpha amylase, catalytic domain	0.524
PF01263	+	Aldose 1-epimerase	0.460
PF03881	+	Fructosamine kinase	0.430
PF00455	+	DeoR C terminal sensor domain	0.425
PF00614	+	Phospholipase D Active site motif	0.393
PF07470	+	Glycosyl Hydrolase Family 88	0.393
PF02926	+	THUMP domain	0.372
PF00854	+	POT family	0.349
PF00303	+	Thymidylate synthase	0.348
PF03733	+	Domain of unknown function (DUF307)	0.333
PF01643	+	Acyl-ACP thioesterase	0.330
PF02952	+	L-fucose isomerase, C-terminal domain	0.316
PF03632	+	Glycosyl hydrolase family 65 central catalytic domain	0.308
PF02486	+	Replication initiation factor	0.288
PF05524	+	PEP-utilising enzyme, N-terminal	0.250
PF00756	+	Putative esterase	0.232
PF02110	-	Hydroxyethylthiazole kinase family	0.226
PF09382	+	RQC domain	0.223
PF03956	+	Membrane protein of unknown function (DUF340)	0.215
PF00359	+	Phosphoenolpyruvate-dependent sugar phosphotransferase system, EIIA 2	0.206
PF01980	+	Uncharacterised protein family UPF0066	0.184
PF08240	+	Alcohol dehydrogenase GroES-like domain	0.156
PF02498	+	BRO family, N-terminal domain	0.118
PF11139	+	Protein of unknown function (DUF2910)	0.117
PF12161	+	HsdM N-terminal domain	0.099
PF06293	+	Lipopolysaccharide kinase (Kdo/WaaP) family	0.097

PF09297	+	NADH pyrophosphatase zinc ribbon domain	0.075
PF10544	+	T5orf172 domain	0.065
PF01844	-	HNH endonuclease	0.041
PF13276	-	HTH-like domain	0.034
PF07508	-	Recombinase	0.007
PF03486	-	HI0933-like protein	-0.013
PF13530	-	Sterol carrier protein domain	-0.031
PF12831	-	FAD dependent oxidoreductase	-0.034
PF03845	-	Spore germination protein	-0.049
PF01210	-	NAD-dependent glycerol-3-phosphate dehydrogenase N-terminus	-0.056
PF13594	-	Amidohydrolase	-0.063
PF02894	-	Oxidoreductase family, C-terminal alpha/beta domain	-0.081
PF03681	-	Uncharacterised protein family (UPF0150)	-0.099
PF04452	-	RNA methyltransferase	-0.117
PF00467	-	KOW motif	-0.117
PF02322	-	Cytochrome oxidase subunit II	-0.119
PF00916	-	Sulfate transporter family	-0.130
PF04011	-	LemA family	-0.131
PF12974	-	ABC transporter, phosphonate, periplasmic substrate-binding protein	-0.149
PF01728	-	FtsJ-like methyltransferase	-0.192
PF01558	-	Pyruvate ferredoxin/flavodoxin oxidoreductase	-0.197
PF13563	-	2'-5' RNA ligase superfamily	-0.204
PF01326	-	Pyruvate phosphate dikinase, PEP/pyruvate binding domain	-0.241
PF05962	-	HutD	-0.241
PF00015	-	Methyl-accepting chemotaxis protein (MCP) signalling domain	-0.245
PF03458	-	UPF0126 domain	-0.247
PF13533	-	Biotin-lipoyl like	-0.250
PF07568	-	Histidine kinase	-0.250
PF12797	-	4Fe-4S binding domain	-0.256
PF01882	-	Protein of unknown function DUF58	-0.259
PF02233	-	NAD(P) transhydrogenase beta subunit	-0.264
PF04473	-	Transglutaminase-like domain	-0.269
PF04536	-	TLP18.3, Psb32 and MOLO-1 founding proteins of phosphatase	-0.276
PF04879	-	Molybdopterin oxidoreductase Fe4S4 domain	-0.284
PF02511	-	Thymidylate synthase complementing protein	-0.291
PF04324	-	BFD-like [2Fe-2S] binding domain	-0.291
PF02503	-	Polyphosphate kinase middle domain	-0.310
PF00563	-	EAL domain	-0.310
PF02934	-	GatB/GatE catalytic domain	-0.330
PF02637	-	GatB domain	-0.330
PF03976	-	Polyphosphate kinase 2 (PPK2)	-0.340
PF02515	-	CoA-transferase family III	-0.361
PF01774	-	UreD urease accessory protein	-0.366
PF04402	-	Protein of unknown function (DUF541)	-0.370
PF13478	-	XdhC Rossmann domain	-0.377
PF04299	-	Putative FMN-binding domain	-0.378
PF00037	-	4Fe-4S binding domain	-0.386
PF00883	-	Cytosol aminopeptidase family, catalytic domain	-0.386

PF13746	-	4Fe-4S dicluster domain	-0.410
PF10588	-	NADH-ubiquinone oxidoreductase-G iron-sulfur binding region	-0.424
PF03594	-	Benzoate membrane transport protein	-0.437
PF03968	-	OstA-like protein	-0.448
PF04185	-	Phosphoesterase family	-0.452
PF14693	-	Ribosomal protein TL5, C-terminal domain	-0.459
PF00743	-	Flavin-binding monooxygenase-like	-0.460
PF05726	-	Pirin C-terminal cupin domain	-0.463
PF00146	-	NADH dehydrogenase	-0.471
PF01329	-	Pterin 4 alpha carbinolamine dehydratase	-0.506
PF05221	-	S-adenosyl-L-homocysteine hydrolase	-0.565

Phenotype: Glucose fermenter (Growth: Glucose), Predictor: phypat+PGL

Pfam	class	Pfam_desc	cor
PF01238	+	Phosphomannose isomerase type I	0.697
PF05116	+	Sucrose-6F-phosphate phosphohydrolase	0.657
PF02664	+	S-Ribosylhomocysteinase (LuxS)	0.632
PF10509	+	Galactokinase galactose-binding signature	0.631
PF00358	+	phosphoenolpyruvate-dependent sugar phosphotransferase system, EIIA 1	0.630
PF03611	+	PTS system sugar-specific permease component	0.626
PF00367	+	phosphotransferase system, EIIB	0.605
PF00232	+	Glycosyl hydrolase family 1	0.603
PF03123	+	CAT RNA binding domain	0.600
PF02744	+	Galactose-1-phosphate uridyl transferase, C-terminal domain	0.597
PF00251	+	Glycosyl hydrolases family 32 N-terminal domain	0.588
PF02863	+	Arginine repressor, C-terminal domain	0.574
PF01316	+	Arginine repressor, DNA binding domain	0.563
PF10437	+	Bacterial lipoate protein ligase C-terminus	0.551
PF02929	+	Beta galactosidase small chain	0.524
PF00128	+	Alpha amylase, catalytic domain	0.524
PF03932	+	CutC family	0.437
PF03881	+	Fructosamine kinase	0.430
PF00455	+	DeoR C terminal sensor domain	0.425
PF13349	+	Domain of unknown function (DUF4097)	0.421
PF02610	+	L-arabinose isomerase	0.398
PF02457	+	DisA bacterial checkpoint controller nucleotide-binding	0.377
PF02926	+	THUMP domain	0.372
PF01418	+	Helix-turn-helix domain, rpiR family	0.370
PF13684	+	Dihydroxyacetone kinase family	0.361
PF00854	+	POT family	0.349
PF00186	+	Dihydrofolate reductase	0.348
PF01487	+	Type I 3-dehydroquinase	0.338
PF01643	+	Acyl-ACP thioesterase	0.330
PF00871	+	Acetokinase family	0.322
PF13930	+	DNA/RNA non-specific endonuclease	0.317
PF02386	+	Cation transport protein	0.316
PF01371	+	Trp repressor protein	0.310
PF03632	+	Glycosyl hydrolase family 65 central catalytic domain	0.308

PF02486	+	Replication initiation factor	0.288
PF01458	-	Uncharacterized protein family (UPF0051)	0.285
PF05738	-	Cna protein B-type domain	0.283
PF09313	+	Domain of unknown function (DUF1971)	0.280
PF05133	+	Phage portal protein, SPP1 Gp6-like	0.279
PF11611	+	Domain of unknown function (DUF4352)	0.277
PF00689	+	Cation transporting ATPase, C-terminus	0.261
PF04399	+	Glutaredoxin 2, C terminal domain	0.254
PF00056	+	lactate/malate dehydrogenase, NAD binding domain	0.251
PF02866	+	lactate/malate dehydrogenase, alpha/beta C-terminal domain	0.251
PF13246	+	Putative hydrolase of sodium-potassium ATPase alpha subunit	0.228
PF04298	+	Putative neutral zinc metallopeptidase	0.224
PF13173	+	AAA domain	0.219
PF05592	+	Bacterial alpha-L-rhamnosidase	0.216
PF03956	+	Membrane protein of unknown function (DUF340)	0.215
PF08531	+	Alpha-L-rhamnosidase N-terminal domain	0.215
PF01680	+	SOR/SNZ family	0.212
PF02424	-	ApbE family	0.199
PF01232	-	Mannitol dehydrogenase Rossmann domain	0.197
PF02446	+	4-alpha-glucanotransferase	0.197
PF07751	+	Abi-like protein	0.193
PF01694	-	Rhomboid family	0.189
PF01943	-	Polysaccharide biosynthesis protein	0.184
PF12911	+	N-terminal TM domain of oligopeptide transport permease C	0.175
PF02080	+	TrkA-C domain	0.173
PF13408	+	Recombinase zinc beta ribbon domain	0.169
PF04883	-	Bacteriophage HK97-gp10, putative tail-component	0.169
PF06245	+	Protein of unknown function (DUF1015)	0.158
PF14827	-	Sensory domain of two-component sensor kinase	0.148
PF03808	-	Glycosyl transferase WecB/TagA/CpsF family	0.141
PF09509	+	Protein of unknown function (Hypoth_ymh)	0.138
PF08817	+	WXG100 protein secretion system (Wss), protein YukD	0.132
PF11842	+	Domain of unknown function (DUF3362)	0.131
PF10004	+	Uncharacterized protein conserved in bacteria (DUF2247)	0.128
PF11139	+	Protein of unknown function (DUF2910)	0.117
PF07739	-	TipAS antibiotic-recognition domain	0.112
PF14436	+	Bacterial EndoU nuclease	0.111
PF02653	+	Branched-chain amino acid transport system / permease component	0.109
PF01081	+	KDPG and KHG aldolase	0.108
PF02796	-	Helix-turn-helix domain of resolvase	0.099
PF12161	+	HsdM N-terminal domain	0.099
PF05065	-	Phage capsid family	0.086
PF00722	+	Glycosyl hydrolases family 16	0.081
PF06575	+	Protein of unknown function (DUF1132)	0.076
PF00591	+	Glycosyl transferase family, a/b domain	0.074
PF13669	-	Glyoxalase/Bleomycin resistance protein/Dioxygenase superfamily	0.068
PF10544	+	T5orf172 domain	0.065
PF00665	-	Integrase core domain	0.058

PF09375	+	Imelysin	0.058
PF13287	+	Fn3 associated	0.052
PF00078	-	Reverse transcriptase (RNA-dependent DNA polymerase)	0.052
PF07606	+	Protein of unknown function (DUF1569)	0.051
PF01885	-	RNA 2'-phosphotransferase, Tpt1 / KptA family	0.049
PF03575	-	Peptidase family S51	0.047
PF02225	-	PA domain	0.043
PF06044	+	Dam-replacing family	0.034
PF13276	-	HTH-like domain	0.034
PF09364	-	XFP N-terminal domain	0.032
PF13353	-	4Fe-4S single cluster domain	0.031
PF01726	-	LexA DNA binding domain	0.027
PF00596	-	Class II Aldolase and Adducin N-terminal domain	0.019
PF03029	+	Conserved hypothetical ATP binding protein	0.014
PF13485	-	Peptidase MA superfamily	0.014
PF08392	+	FAE1/Type III polyketide synthase-like protein	0.008
PF07508	-	Recombinase	0.007
PF12437	+	Glutamine synthetase type III N terminal	0.002
PF00239	-	Resolvase, N terminal domain	0.002
PF13551	+	Winged helix-turn helix	-0.000
PF14078	+	Domain of unknown function (DUF4259)	-0.001
PF03432	-	Relaxase/Mobilisation nuclease domain	-0.005
PF00989	+	PAS fold	-0.006
PF01188	-	Mandelate racemase / muconate lactonizing enzyme, C-terminal domain	-0.010
PF02868	-	Thermolysin metallopeptidase, alpha-helical domain	-0.014
PF06210	-	Protein of unknown function (DUF1003)	-0.014
PF11941	-	Domain of unknown function (DUF3459)	-0.016
PF12627	-	Probable RNA and SrmB- binding site of polymerase A	-0.023
PF00724	-	NADH:flavin oxidoreductase / NADH oxidase family	-0.028
PF13530	-	Sterol carrier protein domain	-0.031
PF08443	+	RimK-like ATP-grasp domain	-0.036
PF04343	+	Protein of unknown function, DUF488	-0.041
PF00933	-	Glycosyl hydrolase family 3 N terminal domain	-0.042
PF13088	-	BNR repeat-like domain	-0.044
PF13434	-	L-lysine 6-monooxygenase (NADPH-requiring)	-0.045
PF00805	-	Pentapeptide repeats (8 copies)	-0.045
PF12846	+	AAA-like domain	-0.053
PF12696	-	TraM recognition site of TraD and TraG	-0.058
PF13392	-	HNH endonuclease	-0.063
PF04134	-	Protein of unknown function, DUF393	-0.068
PF13855	-	Leucine rich repeat	-0.068
PF13378	-	Enolase C-terminal domain-like	-0.070
PF04389	-	Peptidase family M28	-0.070
PF01402	+	Ribbon-helix-helix protein, copG family	-0.075
PF10397	+	Adenylosuccinate lyase C-terminus	-0.076
PF01946	-	Thi4 family	-0.077
PF14552	-	Tautomerase enzyme	-0.078
PF02894	-	Oxidoreductase family, C-terminal alpha/beta domain	-0.081

PF01510	-	N-acetylmuramoyl-L-alanine amidase	-0.086
PF00042	-	Globin	-0.089
PF04371	-	Porphyromonas-type peptidyl-arginine deiminase	-0.093
PF08843	-	Nucleotidyl transferase of unknown function (DUF1814)	-0.097
PF03681	-	Uncharacterised protein family (UPF0150)	-0.099
PF13827	+	Domain of unknown function (DUF4189)	-0.107
PF02646	+	RmuC family	-0.110
PF13586	+	Transposase DDE domain	-0.110
PF01478	-	Type IV leader peptidase family	-0.121
PF13454	-	FAD-NAD(P)-binding	-0.125
PF01663	-	Type I phosphodiesterase / nucleotide pyrophosphatase	-0.128
PF04011	-	LemA family	-0.131
PF05621	+	Bacterial TniB protein	-0.135
PF07804	+	HipA-like C-terminal domain	-0.136
PF08645	-	Polynucleotide kinase 3 phosphatase	-0.137
PF00877	-	NlpC/P60 family	-0.138
PF01678	+	Diaminopimelate epimerase	-0.147
PF01891	-	Cobalt uptake substrate-specific transmembrane region	-0.148
PF00493	-	MCM2/3/5 family	-0.155
PF07683	+	Cobalamin synthesis protein cobW C-terminal domain	-0.155
PF08436	-	1-deoxy-D-xylulose 5-phosphate reductoisomerase C-terminal	-0.162
PF03023	-	MviN-like protein	-0.171
PF11791	+	Aconitate B N-terminal domain	-0.187
PF06564	-	YhjQ protein	-0.188
PF01728	-	FtsJ-like methyltransferase	-0.192
PF06751	-	Ethanolamine ammonia lyase large subunit (EutB)	-0.193
PF08007	+	Cupin superfamily protein	-0.195
PF13745	-	HxxPF-repeated domain	-0.197
PF01551	-	Peptidase family M23	-0.198
PF03737	-	Demethylmenaquinone methyltransferase	-0.199
PF01914	-	MarC family integral membrane protein	-0.199
PF13591	-	MerR HTH family regulatory protein	-0.199
PF01124	+	MAPEG family	-0.201
PF08484	+	C-methyltransferase C-terminal domain	-0.205
PF10503	+	Esterase PHB depolymerase	-0.207
PF02543	-	Carbamoyltransferase	-0.207
PF02469	-	Fasciclin domain	-0.207
PF13468	-	Glyoxalase-like domain	-0.223
PF00565	-	Staphylococcal nuclease homologue	-0.231
PF08643	-	Fungal family of unknown function (DUF1776)	-0.240
PF05893	-	Acyl-CoA reductase (LuxC)	-0.245
PF04199	-	Putative cyclase	-0.249
PF13533	-	Biotin-lipoyl like	-0.250
PF03572	-	Peptidase family S41	-0.250
PF08223	-	PaaX-like protein C-terminal domain	-0.277
PF00174	-	Oxidoreductase molybdopterin binding domain	-0.285
PF12680	-	SnoaL-like domain	-0.288
PF04324	-	BFD-like [2Fe-2S] binding domain	-0.291

PF01896	-	Eukaryotic and archaeal DNA primase small subunit	-0.295
PF13470	+	PIN domain	-0.305
PF07366	-	SnoaL-like polyketide cyclase	-0.315
PF13400	-	Putative Flp pilus-assembly TadE/G-like	-0.316
PF01258	-	Prokaryotic dksA/traR C4-type zinc finger	-0.318
PF01618	-	MotA/TolQ/ExbB proton channel family	-0.330
PF03976	-	Polyphosphate kinase 2 (PPK2)	-0.340
PF14031	-	Putative serine dehydratase domain	-0.343
PF04610	-	TrbL/VirB6 plasmid conjugal transfer protein	-0.348
PF13577	-	SnoaL-like domain	-0.366
PF00355	-	Rieske [2Fe-2S] domain	-0.375
PF10996	-	Beta-Casp domain	-0.379
PF14759	-	Reductase C-terminal	-0.404

Phenotype: Glucose oxidizer (Growth: Glucose), Predictor: phypat

Pfam	class	Pfam_desc	cor
PF10118	+	Predicted metal-dependent hydrolase	0.814
PF12391	+	Protocatechuate 3,4-dioxygenase beta subunit N terminal	0.769
PF12806	+	Acetyl-CoA dehydrogenase C-terminal like	0.750
PF12418	+	Acyl-CoA dehydrogenase N terminal	0.750
PF13298	+	DNA polymerase Ligase (LigD)	0.737
PF00775	+	Dioxygenase	0.712
PF13628	+	Domain of unknown function (DUF4142)	0.705
PF09349	+	OHCU decarboxylase	0.700
PF04115	+	Ureidoglycolate hydrolase	0.659
PF08494	+	DEAD/H associated	0.588
PF03576	+	Peptidase family S58	0.543
PF08450	+	SMP-30/Gluconolaconase/LRE-like region	0.526
PF01804	+	Penicillin amidase	0.520
PF01774	+	UreD urease accessory protein	0.513
PF09423	+	PhoD-like phosphatase	0.506
PF13835	+	Domain of unknown function (DUF4194)	0.392
PF06912	+	Protein of unknown function (DUF1275)	0.328
PF13414	-	TPR repeat	0.020
PF01938	-	TRAM domain	-0.001
PF02080	-	TrkA-C domain	-0.051
PF00689	-	Cation transporting ATPase, C-terminus	-0.052
PF00871	-	Acetokinase family	-0.097
PF05198	-	Translation initiation factor IF-3, N-terminal domain	-0.118
PF03222	-	Tryptophan/tyrosine permease family	-0.123
PF08211	-	Cytidine and deoxycytidylate deaminase zinc-binding region	-0.219
PF05193	-	Peptidase M16 inactive domain	-0.259
PF10509	-	Galactokinase galactose-binding signature	-0.304
PF07155	-	ECF-type riboflavin transporter, S component	-0.351
PF00265	-	Thymidine kinase	-0.358
PF00365	-	Phosphofructokinase	-0.376
PF01228	-	Glycine radical	-0.429
PF02664	-	S-Ribosylhomocysteinase (LuxS)	-0.461

Phenotype: Glucose oxidizer (Growth: Glucose), Predictor: phypat+PGL

Pfam	class	Pfam_desc	cor
PF10118	+	Predicted metal-dependent hydrolase	0.814
PF12391	+	Protocatechuate 3,4-dioxygenase beta subunit N terminal	0.769
PF13298	+	DNA polymerase Ligase (LigD)	0.737
PF13628	+	Domain of unknown function (DUF4142)	0.705
PF03168	+	Late embryogenesis abundant protein	0.691
PF06155	+	Protein of unknown function (DUF971)	0.675
PF11583	+	P-aminobenzoate N-oxygenase AurF	0.657
PF01028	+	Eukaryotic DNA topoisomerase I, catalytic core	0.630
PF04185	+	Phosphoesterase family	0.628
PF09490	+	Probable cobalt transporter subunit (CbtA)	0.606
PF08494	+	DEAD/H associated	0.588
PF07077	+	Protein of unknown function (DUF1345)	0.581
PF10129	+	OpgC protein	0.562
PF08450	+	SMP-30/Gluconolaconase/LRE-like region	0.526
PF03621	+	MbtH-like protein	0.496
PF02814	+	UreE urease accessory protein, N-terminal domain	0.464
PF04675	+	DNA ligase N terminus	0.453
PF05194	+	UreE urease accessory protein, C-terminal domain	0.445
PF10503	+	Esterase PHB depolymerase	0.445
PF06833	+	Malonate decarboxylase gamma subunit (MdcE)	0.429
PF12766	+	Pyridoxamine 5'-phosphate oxidase	0.429
PF09981	+	Uncharacterized protein conserved in bacteria (DUF2218)	0.409
PF04655	+	Aminoglycoside/hydroxyurea antibiotic resistance kinase	0.395
PF13835	+	Domain of unknown function (DUF4194)	0.392
PF12769	+	Domain of unknown function (DUF3814)	0.374
PF13449	+	Esterase-like activity of phytase	0.367
PF13340	+	Putative transposase of IS4/5 family (DUF4096)	0.361
PF00211	+	Adenylate and Guanylate cyclase catalytic domain	0.343
PF02469	+	Fasciclin domain	0.339
PF07286	+	Protein of unknown function (DUF1445)	0.324
PF13700	+	Domain of unknown function (DUF4158)	0.318
PF06966	+	Protein of unknown function (DUF1295)	0.298
PF10517	+	Electron transfer DM13	0.261
PF12796	-	Ankyrin repeats (3 copies)	0.255
PF02915	+	Rubrerythrin	0.247
PF10397	+	Adenylosuccinate lyase C-terminus	0.236
PF02720	+	Domain of unknown function (DUF222)	0.236
PF02954	-	Bacterial regulatory protein, Fis family	0.204
PF04473	+	Transglutaminase-like domain	0.185
PF01447	-	Thermolysin metallopeptidase, catalytic domain	0.181
PF13156	+	Restriction endonuclease	0.181
PF02624	+	YcaO-like family	0.178
PF02868	-	Thermolysin metallopeptidase, alpha-helical domain	0.176
PF02554	-	Carbon starvation protein CstA	0.170
PF13722	-	C-terminal domain on CstA (DUF4161)	0.170

PF01894	-	Uncharacterised protein family UPF0047	0.149
PF04794	-	YdjC-like protein	0.140
PF13020	+	Domain of unknown function (DUF3883)	0.139
PF07584	+	Aerotolerance regulator N-terminal	0.097
PF13087	-	AAA domain	0.092
PF04172	-	LrgB-like family	0.086
PF00482	-	Type II secretion system (T2SS), protein F	0.070
PF04213	+	Htaa	0.061
PF01408	-	Oxidoreductase family, NAD-binding Rossmann fold	0.052
PF00704	-	Glycosyl hydrolases family 18	0.037
PF00755	+	Choline/Carnitine o-acyltransferase	0.036
PF04183	-	IucA / IucC family	0.035
PF02896	-	PEP-utilising enzyme, TIM barrel domain	0.033
PF03209	+	PUCC protein	0.014
PF02592	-	Uncharacterized ACR, YhhQ family COG1738	0.000
PF10145	-	Phage-related minor tail protein	-0.015
PF03681	-	Uncharacterised protein family (UPF0150)	-0.016
PF13730	-	Helix-turn-helix domain	-0.023
PF02086	-	D12 class N6 adenine-specific DNA methyltransferase	-0.025
PF14133	+	Domain of unknown function (DUF4300)	-0.052
PF00195	-	Chalcone and stilbene synthases, N-terminal domain	-0.052
PF07553	+	Host cell surface-exposed lipoprotein	-0.072
PF02535	-	ZIP Zinc transporter	-0.079
PF14897	-	EpsG family	-0.087
PF05198	-	Translation initiation factor IF-3, N-terminal domain	-0.118
PF06415	-	BPG-independent PGAM N-terminus (iPGM_N)	-0.123
PF02659	-	Domain of unknown function DUF	-0.149
PF02074	-	Carboxypeptidase Taq (M32) metallopeptidase	-0.182
PF12464	-	Maltose acetyltransferase	-0.200
PF08211	-	Cytidine and deoxycytidylate deaminase zinc-binding region	-0.219
PF09445	-	RNA cap guanine-N2 methyltransferase	-0.240
PF00265	-	Thymidine kinase	-0.358
PF01228	-	Glycine radical	-0.429
PF02664	-	S-Ribosylhomocysteinase (LuxS)	-0.461

Phenotype: Methyl red (Growth: Glucose), Predictor: phypat

Pfam	class	Pfam_desc	cor
PF12793	+	Sugar transport-related sRNA regulator N-term	0.857
PF07694	+	5TMR of 5TMR-LYT	0.825
PF06146	+	Phosphate-starvation-inducible E	0.825
PF13375	+	RnfC Barrel sandwich hybrid domain	0.825
PF11398	+	Protein of unknown function (DUF2813)	0.763
PF05728	+	Uncharacterised protein family (UPF0227)	0.747
PF11922	+	Domain of unknown function (DUF3440)	0.700
PF07383	+	Protein of unknown function (DUF1496)	0.691
PF09829	+	Uncharacterized protein conserved in bacteria (DUF2057)	0.691
PF04227	+	Indigoidine synthase A like protein	0.665
PF04888	+	Secretion system effector C (SseC) like family	0.648

PF07201	+	HrpJ-like domain	0.648
PF07559	+	Flagellar basal body protein FlaE	0.648
PF02126	+	Phosphotriesterase family	0.617
PF05106	+	Phage holin family (Lysis protein S)	0.615
PF02839	+	Carbohydrate binding domain	0.611
PF11319	+	Protein of unknown function (DUF3121)	0.582
PF05171	+	Haemin-degrading HemS.ChuX domain	0.581
PF05985	+	Ethanolamine ammonia-lyase light chain (EutC)	0.581
PF07369	+	Protein of unknown function (DUF1488)	0.581
PF04264	+	YceI-like domain	0.567
PF07105	+	Protein of unknown function (DUF1367)	0.523
PF10145	+	Phage-related minor tail protein	0.503
PF03067	+	Chitin binding domain	0.469
PF02308	+	MgtC family	0.465
PF13087	+	AAA domain	0.331
PF03729	+	Short repeat of unknown function (DUF308)	0.247
PF02677	-	Uncharacterized BCR, COG1636	-0.113
PF05870	-	Phenolic acid decarboxylase (PAD)	-0.140
PF01235	-	Sodium:alanine symporter family	-0.158
PF12740	-	Chlorophyllase enzyme	-0.165
PF01935	-	Domain of unknown function DUF87	-0.166
PF02397	-	Bacterial sugar transferase	-0.171
PF05402	-	Coenzyme PQQ synthesis protein D (PqqD)	-0.200
PF02588	-	Uncharacterized BCR, YitT family COG1284	-0.220
PF08450	-	SMP-30/Gluconolaconase/LRE-like region	-0.290
PF13442	-	Cytochrome C oxidase, cbb3-type, subunit III	-0.306
PF05893	-	Acyl-CoA reductase (LuxC)	-0.311
PF02541	-	Ppx/GppA phosphatase family	-0.324
PF13587	-	N-terminal domain of DJ-1_PfpI family	-0.366
PF07685	-	CobB/CobQ-like glutamine amidotransferase domain	-0.370
PF00481	-	Protein phosphatase 2C	-0.371
PF01637	-	Archaeal ATPase	-0.380
PF03070	-	TENA/THI-4/PQQC family	-0.403
PF03060	-	Nitronate monooxygenase	-0.420
PF03976	-	Polyphosphate kinase 2 (PPK2)	-0.469
PF00071	-	Ras family	-0.503
PF04296	-	Protein of unknown function (DUF448)	-0.514
PF07521	-	RNA-metabolising metallo-beta-lactamase	-0.538
PF02934	-	GatB/GatE catalytic domain	-0.547
PF02637	-	GatB domain	-0.547
PF02686	-	Glu-tRNAGln amidotransferase C subunit	-0.547

Phenotype: Methyl red (Growth: Glucose), Predictor: phypat+PGL

Pfam	class	Pfam_desc	cor
PF12793	+	Sugar transport-related sRNA regulator N-term	0.857
PF11922	+	Domain of unknown function (DUF3440)	0.700
PF07383	+	Protein of unknown function (DUF1496)	0.691
PF09829	+	Uncharacterized protein conserved in bacteria (DUF2057)	0.691

PF04227	+	Indigoidine synthase A like protein	0.665
PF10678	+	Protein of unknown function (DUF2492)	0.665
PF11756	+	Nitrous oxide-stimulated promoter	0.654
PF07559	+	Flagellar basal body protein FlaE	0.648
PF02126	+	Phosphotriesterase family	0.617
PF03170	+	Bacterial cellulose synthase subunit	0.616
PF08351	+	Domain of unknown function (DUF1726)	0.615
PF01931	+	Protein of unknown function DUF84	0.609
PF07369	+	Protein of unknown function (DUF1488)	0.581
PF05134	+	Type II secretion system (T2SS), protein L	0.559
PF05987	+	Bacterial protein of unknown function (DUF898)	0.544
PF02316	+	Mu DNA-binding domain	0.523
PF06998	+	Protein of unknown function (DUF1307)	0.523
PF06923	+	Glucitol operon activator protein (GutM)	0.491
PF03067	+	Chitin binding domain	0.469
PF11903	+	Protein of unknown function (DUF3423)	0.411
PF05145	+	Putative ammonia monooxygenase	0.410
PF07916	+	TraG-like protein, N-terminal region	0.392
PF13107	+	Protein of unknown function (DUF3964)	0.324
PF08191	+	LRR adjacent	0.324
PF12354	+	Bacterial adhesion/invasion protein N terminal	0.324
PF12181	+	DNA binding domain of the motility gene repressor (MogR)	0.324
PF11313	+	Protein of unknown function (DUF3116)	0.324
PF07252	+	Protein of unknown function (DUF1433)	0.324
PF11328	+	Protein of unknown function (DUF3130	0.324
PF04480	+	Protein of unknown function (DUF559)	0.309
PF00145	-	C-5 cytosine-specific DNA methylase	0.260
PF03806	+	AbgT putative transporter family	0.245
PF14305	+	TupA-like ATPgrasp	0.235
PF02544	+	3-oxo-5-alpha-steroid 4-dehydrogenase	0.216
PF05973	-	Phage derived protein Gp49-like (DUF891)	0.205
PF01321	+	Creatinase/Prolidase N-terminal domain	0.177
PF00576	-	HIUase/Transthyretin family	0.171
PF10571	+	Uncharacterised protein family UPF0547	0.157
PF02814	+	UreE urease accessory protein, N-terminal domain	0.112
PF00413	+	Matrixin	0.112
PF12969	+	Domain of Unknown Function with PDB structure (DUF3857)	0.104
PF03237	-	Terminase-like family	0.090
PF13610	-	DDE domain	0.032
PF10127	-	Predicted nucleotidyltransferase	0.013
PF00135	-	Carboxylesterase family	0.004
PF05015	-	Plasmid maintenance system killer protein	-0.067
PF04313	-	Type I restriction enzyme R protein N terminus (HSDR_N)	-0.085
PF09296	-	NADH pyrophosphatase-like rudimentary NUDIX domain	-0.090
PF02677	-	Uncharacterized BCR, COG1636	-0.113
PF03448	+	MgtE intracellular N domain	-0.135
PF05870	-	Phenolic acid decarboxylase (PAD)	-0.140
PF03070	-	TENA/THI-4/PQQC family	-0.403

Phenotype: Voges Proskauer (Growth: Glucose), Predictor: phypat

Pfam	class	Pfam_desc	cor
PF03306	+	Alpha-acetolactate decarboxylase	0.746
PF00135	+	Carboxylesterase family	0.399
PF03390	+	2-hydroxycarboxylate transporter family	0.318
PF13576	+	Pentapeptide repeats (9 copies)	0.304
PF13558	+	Putative exonuclease SbcCD, C subunit	0.301
PF04616	+	Glycosyl hydrolases family 43	0.279
PF13601	+	Winged helix DNA-binding domain	0.270
PF03706	+	Uncharacterised protein family (UPF0104)	0.251
PF09314	+	Domain of unknown function (DUF1972)	0.235
PF02350	+	UDP-N-acetylglucosamine 2-epimerase	0.223
PF13528	+	Glycosyl transferase family 1	0.216
PF12799	+	Leucine Rich repeats (2 copies)	0.215
PF03837	+	RecT family	0.163
PF13454	+	FAD-NAD(P)-binding	0.155
PF13730	+	Helix-turn-helix domain	0.146
PF04471	+	Restriction endonuclease	0.054
PF01637	-	Archaeal ATPase	0.021
PF00082	-	Subtilase family	-0.035
PF04956	+	TrbC/VIRB2 family	-0.037
PF09378	-	HAS barrel domain	-0.066
PF02797	-	Chalcone and stilbene synthases, C-terminal domain	-0.073
PF08903	-	Domain of unknown function (DUF1846)	-0.089
PF02870	+	6-O-methylguanine DNA methyltransferase, ribonuclease-like domain	-0.091
PF13521	-	AAA domain	-0.100
PF00754	-	F5/8 type C domain	-0.159
PF00702	-	haloacid dehalogenase-like hydrolase	-0.160
PF04011	-	LemA family	-0.169
PF02537	-	CrcB-like protein	-0.179
PF02667	-	Short chain fatty acid transporter	-0.186
PF03616	-	Sodium/glutamate symporter	-0.191
PF03047	-	COMC family	-0.193
PF01923	-	Cobalamin adenosyltransferase	-0.196
PF03186	-	CobD/Cbib protein	-0.200
PF04973	-	Nicotinamide mononucleotide transporter	-0.201
PF08843	-	Nucleotidyl transferase of unknown function (DUF1814)	-0.224
PF01555	-	DNA methylase	-0.275
PF13482	-	RNase_H superfamily	-0.284
PF03883	-	Protein of unknown function (DUF328)	-0.292
PF03308	-	ArgK protein	-0.318
PF01794	-	Ferric reductase like transmembrane component	-0.353
PF03264	-	NapC/NirT cytochrome c family, N-terminal region	-0.427
PF01472	-	PUA domain	-0.452
PF00766	-	Electron transfer flavoprotein FAD-binding domain	-0.491
PF01012	-	Electron transfer flavoprotein domain	-0.491
PF12801	-	4Fe-4S binding domain	-0.496

PF02683	-	Cytochrome C biogenesis protein transmembrane region	-0.602

Phenotype: Voges Proskauer (Growth: Glucose), Predictor: phypat+PGL

Pfam	class	Pfam_desc	cor
PF03306	+	Alpha-acetolactate decarboxylase	0.746
PF07099	+	Protein of unknown function (DUF1361)	0.603
PF05709	+	Phage tail protein	0.487
PF13303	+	Phosphotransferase system, EIIC	0.486
PF06993	+	Protein of unknown function (DUF1304)	0.486
PF02742	+	Iron dependent repressor, metal binding and dimerisation domain	0.473
PF12464	+	Maltose acetyltransferase	0.440
PF08660	+	Oligosaccharide biosynthesis protein Alg14 like	0.417
PF09966	+	Uncharacterized protein conserved in bacteria (DUF2200)	0.416
PF02664	+	S-Ribosylhomocysteinase (LuxS)	0.383
PF13578	+	Methyltransferase domain	0.379
PF08481	+	GBS Bsp-like repeat	0.349
PF13791	+	Sigma factor regulator C-terminal	0.282
PF00854	+	POT family	0.276
PF07081	+	Protein of unknown function (DUF1349)	0.265
PF03706	+	Uncharacterised protein family (UPF0104)	0.251
PF08546	+	Ketopantoate reductase PanE/ApbA C terminal	0.236
PF02350	+	UDP-N-acetylglucosamine 2-epimerase	0.223
PF08533	+	Beta-galactosidase C-terminal domain	0.221
PF12799	+	Leucine Rich repeats (2 copies)	0.215
PF03632	+	Glycosyl hydrolase family 65 central catalytic domain	0.215
PF03636	+	Glycosyl hydrolase family 65, N-terminal domain	0.215
PF13367	-	Protease prsW family	0.159
PF10020	+	Uncharacterized protein conserved in bacteria (DUF2262)	0.148
PF10371	-	Domain of unknown function	0.096
PF14567	+	SMI1-KNR4 cell-wall	0.096
PF13380	+	CoA binding domain	0.082
PF07358	+	Protein of unknown function (DUF1482)	0.081
PF10551	+	MULE transposase domain	0.077
PF07669	-	Eco57I restriction-modification methylase	0.052
PF00703	-	Glycosyl hydrolases family 2	0.032
PF11958	+	Domain of unknown function (DUF3472)	0.024
PF01637	-	Archaeal ATPase	0.021
PF05145	-	Putative ammonia monooxygenase	0.018
PF01641	-	SelR domain	0.015
PF13754	-	Bacterial Ig-like domain (group 3)	-0.007
PF03767	-	HAD superfamily, subfamily IIIB (Acid phosphatase)	-0.010
PF01478	-	Type IV leader peptidase family	-0.027
PF08535	-	KorB domain	-0.031
PF00082	-	Subtilase family	-0.035
PF09995	+	Uncharacterized protein conserved in bacteria (DUF2236)	-0.053
PF00210	-	Ferritin-like domain	-0.055
PF00850	+	Histone deacetylase domain	-0.065
PF05139	-	Erythromycin esterase	-0.087

PF08323	-	Starch synthase catalytic domain	-0.108
PF14133	+	Domain of unknown function (DUF4300)	-0.111
PF02133	-	Permease for cytosine/purines, uracil, thiamine, allantoin	-0.111
PF07971	-	Glycosyl hydrolase family 92	-0.120
PF03309	-	Type III pantothenate kinase	-0.136
PF02613	+	Nitrate reductase delta subunit	-0.141
PF02127	-	Aminopeptidase I zinc metalloprotease (M18)	-0.150
PF09313	-	Domain of unknown function (DUF1971)	-0.159
PF01896	-	Eukaryotic and archaeal DNA primase small subunit	-0.173
PF04221	-	RelB antitoxin	-0.185
PF00891	-	O-methyltransferase	-0.186
PF05768	-	Glutaredoxin-like domain (DUF836)	-0.189
PF02316	-	Mu DNA-binding domain	-0.200
PF08843	-	Nucleotidyl transferase of unknown function (DUF1814)	-0.224
PF13007	-	Transposase C of IS166 homeodomain	-0.284
PF13857	-	Ankyrin repeats (many copies)	-0.297
PF05717	-	IS66 Orf2 like protein	-0.306
PF13409	-	Glutathione S-transferase, N-terminal domain	-0.312
PF00877	-	NlpC/P60 family	-0.319
PF02550	-	Acetyl-CoA hydrolase/transferase N-terminal domain	-0.331
PF00529	-	HlyD family secretion protein	-0.338
PF03458	+	UPF0126 domain	-0.376
PF03480	-	Bacterial extracellular solute-binding protein, family 7	-0.381
PF03748	-	Flagellar basal body-associated protein FliL	-0.386
PF13386	-	Cytochrome C biogenesis protein transmembrane region	-0.419
PF03264	-	NapC/NirT cytochrome c family, N-terminal region	-0.427

Phenotype: Cellobiose (Growth: Sugar), Predictor: phypat

Pfam	class	Pfam_desc	cor
PF11162	+	Protein of unknown function (DUF2946)	0.790
PF03594	+	Benzoate membrane transport protein	0.696
PF08521	+	Two-component sensor kinase N-terminal	0.694
PF14525	+	AraC-binding-like domain	0.682
PF13444	+	Acetyltransferase (GNAT) domain	0.651
PF10617	+	Protein of unknown function (DUF2474)	0.631
PF13577	+	SnoaL-like domain	0.631
PF03746	+	LamB/YcsF family	0.622
PF05995	+	Cysteine dioxygenase type I	0.620
PF14696	+	Hydroxyphenylpyruvate dioxygenase, HPPD, N-terminal	0.620
PF05138	+	Phenylacetic acid catabolic protein	0.620
PF06243	+	Phenylacetic acid degradation B	0.620
PF12852	+	Cupin	0.617
PF07366	+	SnoaL-like polyketide cyclase	0.605
PF01011	+	PQQ enzyme repeat	0.598
PF14518	+	Iron-containing redox enzyme	0.595
PF05787	+	Bacterial protein of unknown function (DUF839)	0.584
PF07331	+	Tripartite tricarboxylate transporter TctB family	0.570
PF06476	+	Protein of unknown function (DUF1090)	0.565

PF13172	+	PepSY-associated TM helix	0.564
PF08908	+	Domain of unknown function (DUF1852)	0.559
PF05235	+	CHAD domain	0.553
PF03401	+	Tripartite tricarboxylate transporter family receptor	0.553
PF13679	+	Methyltransferase domain	0.546
PF08450	+	SMP-30/Gluconolaconase/LRE-like region	0.545
PF03552	+	Cellulose synthase	0.527
PF03972	+	MmgE/PrpD family	0.527
PF06175	+	tRNA-(MS[2]IO[6]A)-hydroxylase (MiaE)	0.521
PF13703	+	PepSY-associated TM helix	0.504
PF04328	+	Protein of unknown function (DUF466)	0.491
PF03441	+	FAD binding domain of DNA photolyase	0.490
PF01928	+	CYTH domain	0.476
PF12729	+	Four helix bundle sensory module for signal transduction	0.473
PF07077	+	Protein of unknown function (DUF1345)	0.467
PF08808	+	RES domain	0.419
PF02447	+	GntP family permease	0.412
PF05345	+	Putative Ig domain	0.399
PF12568	+	Acetyltransferase (GNAT) domain	0.391
PF13305	+	WHG domain	0.308
PF04230	+	Polysaccharide pyruvyl transferase	0.306
PF01527	+	Transposase	0.252
PF03595	+	Voltage-dependent anion channel	0.246
PF01386	-	Ribosomal L25p family	0.235
PF07732	-	Multicopper oxidase	0.224
PF02277	-	Phosphoribosyltransferase	0.211
PF05656	+	Protein of unknown function (DUF805)	0.211
PF04239	+	Protein of unknown function (DUF421)	0.211
PF02909	+	Tetracyclin repressor, C-terminal all-alpha domain	0.181
PF12294	+	Protein of unknown function (DUF3626)	0.179
PF02322	-	Cytochrome oxidase subunit II	0.175
PF06969	-	HemN C-terminal domain	0.155
PF01890	-	Cobalamin synthesis G C-terminus	0.093
PF13744	-	Helix-turn-helix domain	0.092
PF13286	-	Phosphohydrolase-associated domain	0.034
PF13482	-	RNase_H superfamily	0.027
PF02424	-	ApbE family	-0.018
PF06723	-	MreB/Mbl protein	-0.028
PF03681	-	Uncharacterised protein family (UPF0150)	-0.047
PF04888	-	Secretion system effector C (SseC) like family	-0.048
PF01170	-	Putative RNA methylase family UPF0020	-0.050
PF13643	-	Domain of unknown function (DUF4145)	-0.065
PF11185	-	Protein of unknown function (DUF2971)	-0.067
PF05662	-	Coiled stalk of trimeric autotransporter adhesin	-0.078
PF04326	-	Divergent AAA domain	-0.109
PF09397	-	Ftsk gamma domain	-0.122
PF05658	-	Head domain of trimeric autotransporter adhesin	-0.145
PF06559	-	2'-deoxycytidine 5'-triphosphate deaminase (DCD)	-0.152

PF04011	-	LemA family	-0.160
PF00830	-	Ribosomal L28 family	-0.178
PF00692	-	dUTPase	-0.178
PF00829	-	Ribosomal prokaryotic L21 protein	-0.178
PF02709	-	N-terminal domain of galactosyltransferase	-0.191
PF01809	-	Haemolytic domain	-0.191
PF14078	-	Domain of unknown function (DUF4259)	-0.192
PF14301	-	Domain of unknown function (DUF4376)	-0.222
PF02502	-	Ribose/Galactose Isomerase	-0.238
PF02475	-	Met-10+ like-protein	-0.250
PF06738	-	Protein of unknown function (DUF1212)	-0.318
PF13345	-	Domain of unknown function (DUF4098)	-0.320
PF10662	-	Ethanolamine utilisation - propanediol utilisation	-0.334
PF01154	-	Hydroxymethylglutaryl-coenzyme A synthase N terminal	-0.359
PF08540	-	Hydroxymethylglutaryl-coenzyme A synthase C terminal	-0.379
PF01458	-	Uncharacterized protein family (UPF0051)	-0.381
PF01183	-	Glycosyl hydrolases family 25	-0.417
PF01268	-	Formate--tetrahydrofolate ligase	-0.523

Phenotype: Cellobiose (Growth: Sugar), Predictor: phypat+PGL

Pfam	class	Pfam_desc	cor
PF11162	+	Protein of unknown function (DUF2946)	0.790
PF08521	+	Two-component sensor kinase N-terminal	0.694
PF14535	+	AMP-binding enzyme C-terminal domain	0.648
PF04828	+	Glutathione-dependent formaldehyde-activating enzyme	0.637
PF10617	+	Protein of unknown function (DUF2474)	0.631
PF13577	+	SnoaL-like domain	0.631
PF05232	+	Bacterial Transmembrane Pair family	0.626
PF03746	+	LamB/YcsF family	0.622
PF12852	+	Cupin	0.617
PF04116	+	Fatty acid hydroxylase superfamily	0.600
PF07080	+	Protein of unknown function (DUF1348)	0.597
PF09994	+	Uncharacterized alpha/beta hydrolase domain (DUF2235)	0.597
PF14518	+	Iron-containing redox enzyme	0.595
PF05787	+	Bacterial protein of unknown function (DUF839)	0.584
PF07311	+	Dodecin	0.576
PF05726	+	Pirin C-terminal cupin domain	0.563
PF03450	+	CO dehydrogenase flavoprotein C-terminal domain	0.553
PF03401	+	Tripartite tricarboxylate transporter family receptor	0.553
PF07287	+	Protein of unknown function (DUF1446)	0.544
PF03972	+	MmgE/PrpD family	0.527
PF06175	+	tRNA-(MS[2]IO[6]A)-hydroxylase (MiaE)	0.521
PF00576	+	HIUase/Transthyretin family	0.511
PF02805	+	Metal binding domain of Ada	0.506
PF13703	+	PepSY-associated TM helix	0.504
PF04328	+	Protein of unknown function (DUF466)	0.491
PF13622	+	Thioesterase-like superfamily	0.476
PF01928	+	CYTH domain	0.476

PF12729	+	Four helix bundle sensory module for signal transduction	0.473
PF07077	+	Protein of unknown function (DUF1345)	0.467
PF05899	+	Protein of unknown function (DUF861)	0.419
PF08808	+	RES domain	0.419
PF11160	+	Protein of unknown function (DUF2945)	0.417
PF09859	+	Oxygenase, catalysing oxidative methylation of damaged DNA	0.411
PF06808	+	DctM-like transporters	0.406
PF12568	+	Acetyltransferase (GNAT) domain	0.391
PF02667	+	Short chain fatty acid transporter	0.372
PF08386	+	TAP-like protein	0.370
PF05048	-	Periplasmic copper-binding protein (NosD)	0.364
PF02040	+	Arsenical pump membrane protein	0.362
PF01804	+	Penicillin amidase	0.349
PF04267	+	Sarcosine oxidase, delta subunit family	0.324
PF06983	-	3-demethylubiquinone-9 3-methyltransferase	0.318
PF00394	-	Multicopper oxidase	0.316
PF00821	+	Phosphoenolpyruvate carboxykinase	0.308
PF13305	+	WHG domain	0.308
PF00449	-	Urease alpha-subunit, N-terminal domain	0.306
PF00547	-	Urease, gamma subunit	0.306
PF00699	-	Urease beta subunit	0.306
PF04250	+	Protein of unknown function (DUF429)	0.303
PF11528	+	Protein of unknown function (DUF3224)	0.303
PF14864	-	Alkyl sulfatase C-terminal	0.299
PF03788	-	LrgA family	0.289
PF03412	-	Peptidase C39 family	0.287
PF02133	-	Permease for cytosine/purines, uracil, thiamine, allantoin	0.285
PF00891	-	O-methyltransferase	0.284
PF12242	-	NAD(P)H binding domain of trans-2-enoyl-CoA reductase	0.250
PF15604	+	Putative toxin 43	0.248
PF05973	-	Phage derived protein Gp49-like (DUF891)	0.244
PF11563	-	Protoglobin	0.233
PF07732	-	Multicopper oxidase	0.224
PF08786	-	Domain of unknown function (DUF1795)	0.223
PF04239	+	Protein of unknown function (DUF421)	0.211
PF08274	-	PhnA Zinc-Ribbon	0.211
PF07929	+	Plasmid pRiA4b ORF-3-like protein	0.206
PF11625	+	Protein of unknown function (DUF3253)	0.202
PF02654	-	Cobalamin-5-phosphate synthase	0.196
PF00710	+	Asparaginase	0.192
PF13205	-	Bacterial Ig-like domain	0.191
PF00150	+	Cellulase (glycosyl hydrolase family 5)	0.186
PF02604	-	Antitoxin Phd_YefM, type II toxin-antitoxin system	0.167
PF06969	-	HemN C-terminal domain	0.155
PF05065	+	Phage capsid family	0.154
PF01923	-	Cobalamin adenosyltransferase	0.153
PF02613	-	Nitrate reductase delta subunit	0.152
PF03601	-	Conserved hypothetical protein 698	0.149

PF09314	+	Domain of unknown function (DUF1972)	0.143
PF07199	+	Protein of unknown function (DUF1411)	0.142
PF12495	+	Vegetative insecticide protein 3A N terminal	0.142
PF09131	+	Bacillus thuringiensis delta-Endotoxin, middle domain	0.142
PF08003	-	Protein of unknown function (DUF1698)	0.137
PF07885	-	Ion channel	0.134
PF00933	-	Glycosyl hydrolase family 3 N terminal domain	0.130
PF07693	-	KAP family P-loop domain	0.127
PF00872	+	Transposase, Mutator family	0.113
PF13565	-	Homeodomain-like domain	0.103
PF03831	-	PhnA protein	0.093
PF13540	+	Regulator of chromosome condensation (RCC1) repeat	0.068
PF06966	+	Protein of unknown function (DUF1295)	0.066
PF03895	-	YadA-like C-terminal region	0.060
PF07029	+	CryBP1 protein	0.048
PF03616	-	Sodium/glutamate symporter	0.040
PF00908	-	dTDP-4-dehydrorhamnose 3,5-epimerase	0.030
PF01885	+	RNA 2'-phosphotransferase, Tpt1 / KptA family	0.012
PF14378	-	PAP2 superfamily	0.000
PF09669	-	Phage regulatory protein Rha (Phage_pRha)	-0.000
PF13936	+	Helix-turn-helix domain	-0.008
PF07022	-	Bacteriophage CI repressor helix-turn-helix domain	-0.029
PF01642	-	Methylmalonyl-CoA mutase	-0.062
PF13749	-	ATP-dependent DNA helicase recG C-terminal	-0.063
PF09445	-	RNA cap guanine-N2 methyltransferase	-0.103
PF03308	-	ArgK protein	-0.111
PF03432	-	Relaxase/Mobilisation nuclease domain	-0.123
PF14635	-	Helix-hairpin-helix motif	-0.127
PF01420	-	Type I restriction modification DNA specificity domain	-0.140
PF12895	-	Anaphase-promoting complex, cyclosome, subunit 3	-0.154
PF07669	+	Eco57I restriction-modification methylase	-0.168
PF07155	-	ECF-type riboflavin transporter, S component	-0.182
PF01809	-	Haemolytic domain	-0.191
PF13854	+	Kelch motif	-0.203
PF08706	-	D5 N terminal like	-0.204
PF13495	-	Phage integrase, N-terminal SAM-like domain	-0.210
PF02335	-	Cytochrome c552	-0.249
PF02475	-	Met-10+ like-protein	-0.250
PF10662	-	Ethanolamine utilisation - propanediol utilisation	-0.334

Phenotype: D-Mannitol (Growth: Sugar), Predictor: phypat

Pfam	class	Pfam_desc	cor
PF01232	+	Mannitol dehydrogenase Rossmann domain	0.711
PF00136	+	DNA polymerase family B	0.592
PF13464	+	Domain of unknown function (DUF4115)	0.568
PF00667	+	FAD binding domain	0.552
PF01894	+	Uncharacterised protein family UPF0047	0.537
PF14689	+	Sensor_kinase_SpoOB-type, alpha-helical domain	0.536

PF07663	+	Sorbitol phosphotransferase enzyme II C-terminus	0.529
PF03612	+	Sorbitol phosphotransferase enzyme II N-terminus	0.529
PF01904	+	Protein of unknown function DUF72	0.527
PF09619	+	Type III secretion system lipoprotein chaperone (YscW)	0.498
PF02694	+	Uncharacterised BCR, YnfA/UPF0060 family	0.489
PF00367	+	phosphotransferase system, EIIB	0.486
PF11941	+	Domain of unknown function (DUF3459)	0.485
PF07005	+	Protein of unknown function, DUF1537	0.444
PF02733	+	Dak1 domain	0.439
PF03649	+	Uncharacterised protein family (UPF0014)	0.437
PF06779	+	Protein of unknown function (DUF1228)	0.433
PF02870	+	6-O-methylguanine DNA methyltransferase, ribonuclease-like domain	0.420
PF02706	+	Chain length determinant protein	0.416
PF01909	+	Nucleotidyltransferase domain	0.407
PF08532	+	Beta-galactosidase trimerisation domain	0.401
PF07745	+	Glycosyl hydrolase family 53	0.394
PF08533	+	Beta-galactosidase C-terminal domain	0.381
PF02929	+	Beta galactosidase small chain	0.378
PF14502	+	Helix-turn-helix domain	0.370
PF13425	+	O-antigen ligase like membrane protein	0.367
PF06414	+	Zeta toxin	0.356
PF13727	+	CoA-binding domain	0.355
PF07655	+	Secretin N-terminal domain	0.354
PF02894	+	Oxidoreductase family, C-terminal alpha/beta domain	0.330
PF14742	+	N-terminal domain of (some) glycogen debranching enzymes	0.323
PF13337	+	Putative ATP-dependent Lon protease	0.317
PF08849	+	Putative inner membrane protein (DUF1819)	0.314
PF02604	+	Antitoxin Phd_YefM, type II toxin-antitoxin system	0.307
PF00251	+	Glycosyl hydrolases family 32 N-terminal domain	0.296
PF08747	+	Domain of unknown function (DUF1788)	0.291
PF07905	+	Purine catabolism regulatory protein-like family	0.288
PF08818	+	Domain of unknown function (DU1801)	0.274
PF13565	+	Homeodomain-like domain	0.266
PF10442	+	FIST C domain	0.238
PF08495	+	FIST N domain	0.238
PF00847	+	AP2 domain	0.238
PF07969	-	Amidohydrolase family	0.235
PF09423	+	PhoD-like phosphatase	0.227
PF02368	+	Bacterial Ig-like domain (group 2)	0.226
PF14267	+	Domain of unknown function (DUF4357)	0.219
PF06445	-	GyrI-like small molecule binding domain	0.194
PF01934	+	Protein of unknown function DUF86	0.145
PF01814	-	Hemerythrin HHE cation binding domain	0.134
PF12682	+	Flavodoxin	0.126
PF13536	-	Multidrug resistance efflux transporter	0.099
PF07885	-	Ion channel	0.091
PF01476	-	LysM domain	0.091
PF00268	-	Ribonucleotide reductase, small chain	0.081

PF00885	-	6,7-dimethyl-8-ribityllumazine synthase	0.077
PF03352	-	Methyladenine glycosylase	0.066
PF01432	-	Peptidase family M3	0.054
PF08242	-	Methyltransferase domain	0.051
PF13586	-	Transposase DDE domain	0.051
PF00186	-	Dihydrofolate reductase	0.050
PF00149	-	Calcineurin-like phosphoesterase	0.034
PF13489	-	Methyltransferase domain	0.034
PF06144	-	DNA polymerase III, delta subunit	0.020
PF09439	-	Signal recognition particle receptor beta subunit	0.017
PF01244	-	Membrane dipeptidase (Peptidase family M19)	0.002
PF07853	+	Protein of unknown function (DUF1648)	-0.005
PF13088	-	BNR repeat-like domain	-0.006
PF03576	-	Peptidase family S58	-0.021
PF13385	-	Concanavalin A-like lectin/glucanases superfamily	-0.025
PF06961	-	Protein of unknown function (DUF1294)	-0.026
PF03308	-	ArgK protein	-0.033
PF00578	-	AhpC/TSA family	-0.040
PF00253	-	Ribosomal protein S14p/S29e	-0.040
PF00908	-	dTDP-4-dehydrorhamnose 3,5-epimerase	-0.045
PF08207	-	Elongation factor P (EF-P) KOW-like domain	-0.048
PF00684	-	DnaJ central domain	-0.048
PF01809	-	Haemolytic domain	-0.070
PF07523	-	Bacterial Ig-like domain (group 3)	-0.082
PF11738	-	Protein of unknown function (DUF3298)	-0.099
PF02608	-	Basic membrane protein	-0.105
PF05031	-	Iron Transport-associated domain	-0.149
PF00830	-	Ribosomal L28 family	-0.157
PF09359	-	VTC domain	-0.166
PF00209	-	Sodium:neurotransmitter symporter family	-0.171
PF01183	-	Glycosyl hydrolases family 25	-0.186
PF14253	-	Bacteriophage abortive infection AbiH	-0.195
PF07751	-	Abi-like protein	-0.207
PF02361	-	Cobalt transport protein	-0.218
PF09848	-	Uncharacterized conserved protein (DUF2075)	-0.222
PF03062	-	MBOAT, membrane-bound O-acyltransferase family	-0.223
PF04011	-	LemA family	-0.226
PF01289	-	Thiol-activated cytolysin	-0.239
PF02934	-	GatB/GatE catalytic domain	-0.254
PF02637	-	GatB domain	-0.254
PF01745	-	Isopentenyl transferase	-0.266
PF01988	-	VIT family	-0.279
PF01637	-	Archaeal ATPase	-0.318

Phenotype: D-Mannitol (Growth: Sugar), Predictor: phypat+PGL

Pfam	class	Pfam_desc	cor
PF01232	+	Mannitol dehydrogenase Rossmann domain	0.711
PF13464	+	Domain of unknown function (DUF4115)	0.568

PF04958	+	Arginine N-succinyltransferase beta subunit	0.563
PF04996	+	Succinylarginine dihydrolase	0.530
PF03612	+	Sorbitol phosphotransferase enzyme II N-terminus	0.529
PF06923	+	Glucitol operon activator protein (GutM)	0.529
PF01904	+	Protein of unknown function DUF72	0.527
PF02610	+	L-arabinose isomerase	0.525
PF03170	+	Bacterial cellulose synthase subunit	0.501
PF02056	+	Family 4 glycosyl hydrolase	0.493
PF11975	+	Family 4 glycosyl hydrolase C-terminal domain	0.493
PF02694	+	Uncharacterised BCR, YnfA/UPF0060 family	0.489
PF00367	+	phosphotransferase system, EIIB	0.486
PF06134	+	L-rhamnose isomerase (RhaA)	0.481
PF04347	+	Flagellar biosynthesis protein, FliO	0.473
PF13309	+	HTH domain	0.457
PF03711	+	Orn/Lys/Arg decarboxylase, C-terminal domain	0.446
PF07005	+	Protein of unknown function, DUF1537	0.444
PF02733	+	Dak1 domain	0.439
PF02675	+	S-adenosylmethionine decarboxylase	0.423
PF02839	+	Carbohydrate binding domain	0.418
PF13807	+	G-rich domain on putative tyrosine kinase	0.412
PF07995	-	Glucose / Sorbosone dehydrogenase	0.403
PF08532	+	Beta-galactosidase trimerisation domain	0.401
PF02613	+	Nitrate reductase delta subunit	0.388
PF11533	+	Protein of unknown function (DUF3225)	0.385
PF09317	+	Domain of unknown function (DUF1974)	0.384
PF05656	-	Protein of unknown function (DUF805)	0.381
PF03702	+	Uncharacterised protein family (UPF0075)	0.370
PF00011	+	Hsp20/alpha crystallin family	0.368
PF13425	+	O-antigen ligase like membrane protein	0.367
PF07655	+	Secretin N-terminal domain	0.354
PF02962	+	5-carboxymethyl-2-hydroxymuconate isomerase	0.349
PF03401	+	Tripartite tricarboxylate transporter family receptor	0.348
PF02311	-	AraC-like ligand binding domain	0.346
PF11876	+	Protein of unknown function (DUF3396)	0.338
PF02922	+	Carbohydrate-binding module 48 (Isoamylase N-terminal domain)	0.337
PF04166	+	Pyridoxal phosphate biosynthetic protein PdxA	0.331
PF03441	-	FAD binding domain of DNA photolyase	0.330
PF02734	+	DAK2 domain	0.330
PF02894	+	Oxidoreductase family, C-terminal alpha/beta domain	0.330
PF01293	+	Phosphoenolpyruvate carboxykinase	0.314
PF00724	-	NADH:flavin oxidoreductase / NADH oxidase family	0.313
PF00245	+	Alkaline phosphatase	0.312
PF05401	-	Nodulation protein S (NodS)	0.311
PF02604	+	Antitoxin Phd_YefM, type II toxin-antitoxin system	0.307
PF07166	+	Protein of unknown function (DUF1398)	0.300
PF10052	+	Protein of unknown function (DUF2288)	0.300
PF00251	+	Glycosyl hydrolases family 32 N-terminal domain	0.296
PF01527	+	Transposase	0.293

PF07905	+	Purine catabolism regulatory protein-like family	0.288
PF04963	+	Sigma-54 factor, core binding domain	0.284
PF12639	+	DNase/tRNase domain of colicin-like bacteriocin	0.281
PF07201	-	HrpJ-like domain	0.277
PF01112	-	Asparaginase	0.276
PF13276	+	HTH-like domain	0.275
PF14697	-	4Fe-4S dicluster domain	0.274
PF08818	+	Domain of unknown function (DU1801)	0.274
PF03230	-	Antirestriction protein	0.273
PF13636	+	pre-rRNA processing and ribosome biogenesis	0.269
PF05137	+	Fimbrial assembly protein (PilN)	0.268
PF11638	+	DnaA N-terminal domain	0.266
PF13565	+	Homeodomain-like domain	0.266
PF02627	+	Carboxymuconolactone decarboxylase family	0.264
PF00343	+	Carbohydrate phosphorylase	0.252
PF13551	+	Winged helix-turn helix	0.252
PF03561	+	Allantoicase repeat	0.247
PF01326	-	Pyruvate phosphate dikinase, PEP/pyruvate binding domain	0.247
PF13409	-	Glutathione S-transferase, N-terminal domain	0.244
PF04235	-	Protein of unknown function (DUF418)	0.244
PF01729	-	Quinolinate phosphoribosyl transferase, C-terminal domain	0.240
PF13426	-	PAS domain	0.240
PF10442	+	FIST C domain	0.238
PF08495	+	FIST N domain	0.238
PF03845	+	Spore germination protein	0.235
PF07969	-	Amidohydrolase family	0.235
PF01361	+	Tautomerase enzyme	0.230
PF13358	+	DDE superfamily endonuclease	0.228
PF03572	+	Peptidase family S41	0.228
PF13391	+	HNH endonuclease	0.222
PF03972	-	MmgE/PrpD family	0.219
PF10145	-	Phage-related minor tail protein	0.214
PF02698	-	DUF218 domain	0.210
PF01427	+	D-ala-D-ala dipeptidase	0.200
PF10979	+	Protein of unknown function (DUF2786)	0.200
PF07286	+	Protein of unknown function (DUF1445)	0.200
PF03253	+	Urea transporter	0.200
PF05257	+	CHAP domain	0.200
PF06769	+	Plasmid encoded toxin Txe	0.194
PF00657	-	GDSL-like Lipase/Acylhydrolase	0.192
PF13769	+	Virulence factor	0.192
PF01676	-	Metalloenzyme superfamily	0.183
PF13632	-	Glycosyl transferase family group 2	0.183
PF13744	-	Helix-turn-helix domain	0.182
PF01051	-	Initiator Replication protein	0.173
PF13246	-	Putative hydrolase of sodium-potassium ATPase alpha subunit	0.168
PF07768	+	PVL ORF-50-like family	0.164
PF10656	+	Hypothetical protein of unknown function (DUF2483)	0.164

PF13186	-	Iron-sulfur cluster-binding domain	0.154
PF03599	+	CO dehydrogenase/acetyl-CoA synthase delta subunit	0.151
PF03070	-	TENA/THI-4/PQQC family	0.149
PF13576	-	Pentapeptide repeats (9 copies)	0.146
PF05163	-	DinB family	0.146
PF01934	+	Protein of unknown function DUF86	0.145
PF02515	-	CoA-transferase family III	0.143
PF07511	+	Protein of unknown function (DUF1525)	0.142
PF06439	+	Domain of Unknown Function (DUF1080)	0.141
PF00394	-	Multicopper oxidase	0.138
PF09860	+	Uncharacterized protein conserved in bacteria (DUF2087)	0.122
PF12840	-	Helix-turn-helix domain	0.117
PF03747	-	ADP-ribosylglycohydrolase	0.116
PF11072	+	Protein of unknown function (DUF2859)	0.115
PF03442	+	Carbohydrate binding domain X2	0.114
PF12307	+	Protein of unknown function (DUF3631)	0.107
PF08401	-	Domain of unknown function (DUF1738)	0.105
PF05015	-	Plasmid maintenance system killer protein	0.097
PF07885	-	Ion channel	0.091
PF04432	+	Coenzyme F420 hydrogenase/dehydrogenase, beta subunit C terminus	0.090
PF13114	+	RecO N terminal	0.088
PF07739	+	TipAS antibiotic-recognition domain	0.085
PF06605	+	Prophage endopeptidase tail	0.085
PF12647	+	RNHCP domain	0.084
PF06941	+	5' nucleotidase, deoxy (Pyrimidine), cytosolic type C protein (NT5C)	0.083
PF00037	-	4Fe-4S binding domain	0.079
PF04221	+	RelB antitoxin	0.077
PF00925	-	GTP cyclohydrolase II	0.077
PF03714	+	Bacterial pullanase-associated domain	0.073
PF06030	+	Bacterial protein of unknown function (DUF916)	0.071
PF11797	+	Protein of unknown function C-terminal (DUF3324)	0.071
PF10096	+	Uncharacterized protein conserved in bacteria (DUF2334)	0.071
PF03352	-	Methyladenine glycosylase	0.066
PF05709	+	Phage tail protein	0.065
PF13345	-	Domain of unknown function (DUF4098)	0.060
PF04531	+	Bacteriophage holin	0.058
PF00899	-	ThiF family	0.056
PF01432	-	Peptidase family M3	0.054
PF04140	+	Isoprenylcysteine carboxyl methyltransferase (ICMT) family	0.052
PF06874	+	Firmicute fructose-1,6-bisphosphatase	0.049
PF09369	+	Domain of unknown function (DUF1998)	0.042
PF05048	-	Periplasmic copper-binding protein (NosD)	0.040
PF13424	+	Tetratricopeptide repeat	0.034
PF00565	-	Staphylococcal nuclease homologue	0.022
PF12822	-	Protein of unknown function (DUF3816)	0.014
PF04260	+	Protein of unknown function (DUF436)	0.008
PF03729	-	Short repeat of unknown function (DUF308)	0.006
PF12687	+	Protein of unknown function (DUF3801)	0.006

PF01244	-	Membrane dipeptidase (Peptidase family M19)	0.002
PF13088	-	BNR repeat-like domain	-0.006
PF09992	-	Predicted periplasmic protein (DUF2233)	-0.009
PF02667	-	Short chain fatty acid transporter	-0.009
PF13574	-	Metallo-peptidase family M12B Reprolysin-like	-0.011
PF11804	+	Protein of unknown function (DUF3325)	-0.020
PF05198	-	Translation initiation factor IF-3, N-terminal domain	-0.024
PF09142	+	tRNA Pseudouridine synthase II, C terminal	-0.044
PF12229	+	Putative peptidoglycan binding domain	-0.049
PF03703	-	Bacterial PH domain	-0.063
PF08439	-	Oligopeptidase F	-0.076
PF04914	-	DltD C-terminal region	-0.087
PF13588	-	Type I restriction enzyme R protein N terminus (HSDR_N)	-0.089
PF01174	+	SNO glutamine amidotransferase family	-0.098
PF01710	-	Transposase	-0.115
PF08903	-	Domain of unknown function (DUF1846)	-0.182
PF14253	-	Bacteriophage abortive infection AbiH	-0.195
PF01921	-	tRNA synthetases class I (K)	-0.201
PF07751	-	Abi-like protein	-0.207
PF00071	+	Ras family	-0.219
PF13173	-	AAA domain	-0.251

Phenotype: D-Mannose (Growth: Sugar), Predictor: phypat

Pfam	class	Pfam_desc	cor
PF00251	+	Glycosyl hydrolases family 32 N-terminal domain	0.598
PF08244	+	Glycosyl hydrolases family 32 C terminal	0.564
PF00367	+	phosphotransferase system, EIIB	0.554
PF03830	+	PTS system sorbose subfamily IIB component	0.525
PF03611	+	PTS system sugar-specific permease component	0.515
PF01928	+	CYTH domain	0.510
PF11798	+	IMS family HHH motif	0.497
PF01238	+	Phosphomannose isomerase type I	0.496
PF01232	+	Mannitol dehydrogenase Rossmann domain	0.481
PF02449	+	Beta-galactosidase	0.463
PF01915	+	Glycosyl hydrolase family 3 C-terminal domain	0.459
PF00343	+	Carbohydrate phosphorylase	0.431
PF04337	+	Protein of unknown function, DUF480	0.428
PF06719	+	AraC-type transcriptional regulator N-terminus	0.425
PF02806	+	Alpha amylase, C-terminal all-beta domain	0.424
PF13521	+	AAA domain	0.409
PF06202	+	Amylo-alpha-1,6-glucosidase	0.399
PF11941	+	Domain of unknown function (DUF3459)	0.395
PF13570	+	PQQ-like domain	0.382
PF02901	+	Pyruvate formate lyase	0.381
PF04338	+	Protein of unknown function, DUF481	0.380
PF00728	+	Glycosyl hydrolase family 20, catalytic domain	0.372
PF01909	+	Nucleotidyltransferase domain	0.365
PF02230	+	Phospholipase/Carboxylesterase	0.342

PF06293	+	Lipopolysaccharide kinase (Kdo/WaaP) family	0.321
PF00782	+	Dual specificity phosphatase, catalytic domain	0.321
PF13629	+	Pilus formation protein N terminal region	0.320
PF00135	+	Carboxylesterase family	0.318
PF13488	+	Glycine zipper	0.290
PF05272	+	Virulence-associated protein E	0.289
PF13189	+	Cytidylate kinase-like family	0.279
PF13007	+	Transposase C of IS166 homeodomain	0.245
PF01661	+	Macro domain	0.241
PF12729	+	Four helix bundle sensory module for signal transduction	0.220
PF02557	-	D-alanyl-D-alanine carboxypeptidase	0.204
PF13565	+	Homeodomain-like domain	0.185
PF13657	+	HipA N-terminal domain	0.174
PF10117	+	McrBC 5-methylcytosine restriction system component	0.153
PF01391	+	Collagen triple helix repeat (20 copies)	0.126
PF01050	+	Mannose-6-phosphate isomerase	0.120
PF13428	+	Tetratricopeptide repeat	0.094
PF01784	-	NIF3 (NGG1p interacting factor 3)	0.074
PF10604	-	Polyketide cyclase / dehydrase and lipid transport	0.062
PF13175	-	AAA ATPase domain	0.061
PF13348	-	Tyrosine phosphatase family C-terminal region	0.055
PF01510	-	N-acetylmuramoyl-L-alanine amidase	0.050
PF01633	-	Choline/ethanolamine kinase	0.037
PF02110	-	Hydroxyethylthiazole kinase family	0.036
PF02275	-	Linear amide C-N hydrolases, choloylglycine hydrolase family	0.027
PF08448	-	PAS fold	0.024
PF03119	-	NAD-dependent DNA ligase C4 zinc finger domain	0.010
PF00467	-	KOW motif	0.007
PF00793	-	DAHP synthetase I family	-0.006
PF03577	-	Peptidase family C69	-0.037
PF03681	-	Uncharacterised protein family (UPF0150)	-0.051
PF04023	-	FeoA domain	-0.055
PF01551	-	Peptidase family M23	-0.089
PF02535	-	ZIP Zinc transporter	-0.090
PF13412	-	Winged helix-turn-helix DNA-binding	-0.100
PF01728	-	FtsJ-like methyltransferase	-0.104
PF04011	-	LemA family	-0.114
PF02541	-	Ppx/GppA phosphatase family	-0.124
PF14102	-	Capsule biosynthesis CapC	-0.128
PF12229	-	Putative peptidoglycan binding domain	-0.154
PF00795	-	Carbon-nitrogen hydrolase	-0.162
PF13785	-	Domain of unknown function (DUF4178)	-0.177
PF13187	-	4Fe-4S dicluster domain	-0.187
PF03596	-	Cadmium resistance transporter	-0.191
PF12900	-	Pyridoxamine 5'-phosphate oxidase	-0.299
PF02686	-	Glu-tRNAGln amidotransferase C subunit	-0.392
PF02934	-	GatB/GatE catalytic domain	-0.402
PF02637	-	GatB domain	-0.402

Phenotype: D-Mannose (Growth: Sugar), Predictor: phypat+PGL

Pfam	class	Pfam_desc	cor
PF00251	+	Glycosyl hydrolases family 32 N-terminal domain	0.598
PF08244	+	Glycosyl hydrolases family 32 C terminal	0.564
PF00367	+	phosphotransferase system, EIIB	0.554
PF03611	+	PTS system sugar-specific permease component	0.515
PF01928	+	CYTH domain	0.510
PF11798	+	IMS family HHH motif	0.497
PF01238	+	Phosphomannose isomerase type I	0.496
PF00232	+	Glycosyl hydrolase family 1	0.487
PF01232	+	Mannitol dehydrogenase Rossmann domain	0.481
PF02449	+	Beta-galactosidase	0.463
PF01915	+	Glycosyl hydrolase family 3 C-terminal domain	0.459
PF02378	+	Phosphotransferase system, EIIC	0.453
PF00614	+	Phospholipase D Active site motif	0.450
PF01055	+	Glycosyl hydrolases family 31	0.447
PF12464	+	Maltose acetyltransferase	0.442
PF14310	+	Fibronectin type III-like domain	0.439
PF00343	+	Carbohydrate phosphorylase	0.431
PF00265	+	Thymidine kinase	0.429
PF08323	+	Starch synthase catalytic domain	0.419
PF13521	+	AAA domain	0.409
PF06202	+	Amylo-alpha-1,6-glucosidase	0.399
PF11941	+	Domain of unknown function (DUF3459)	0.395
PF10263	+	SprT-like family	0.392
PF04893	+	Yip1 domain	0.386
PF03009	+	Glycerophosphoryl diester phosphodiesterase family	0.379
PF00393	+	6-phosphogluconate dehydrogenase, C-terminal domain	0.374
PF01909	+	Nucleotidyltransferase domain	0.365
PF01699	+	Sodium/calcium exchanger protein	0.363
PF13597	+	Anaerobic ribonucleoside-triphosphate reductase	0.343
PF06580	+	Histidine kinase	0.329
PF00479	+	Glucose-6-phosphate dehydrogenase, NAD binding domain	0.327
PF04012	+	PspA/IM30 family	0.319
PF00135	+	Carboxylesterase family	0.318
PF04266	-	ASCH domain	0.313
PF13807	+	G-rich domain on putative tyrosine kinase	0.313
PF09863	+	Uncharacterized protein conserved in bacteria (DUF2090)	0.305
PF07221	+	N-acylglucosamine 2-epimerase (GlcNAc 2-epimerase)	0.299
PF06414	+	Zeta toxin	0.284
PF02805	+	Metal binding domain of Ada	0.282
PF07730	+	Histidine kinase	0.281
PF12728	+	Helix-turn-helix domain	0.279
PF06196	-	Protein of unknown function (DUF997)	0.278
PF02624	+	YcaO-like family	0.275
PF00150	+	Cellulase (glycosyl hydrolase family 5)	0.275
PF04198	+	Putative sugar-binding domain	0.272

PF06276	+	Ferric iron reductase FhuF-like transporter	0.271
PF03374	+	Phage antirepressor protein KilAC domain	0.264
PF01116	+	Fructose-bisphosphate aldolase class-II	0.256
PF06115	+	Domain of unknown function (DUF956)	0.255
PF00722	+	Glycosyl hydrolases family 16	0.250
PF01161	+	Phosphatidylethanolamine-binding protein	0.249
PF07005	+	Protein of unknown function, DUF1537	0.248
PF13483	+	Beta-lactamase superfamily domain	0.245
PF13007	+	Transposase C of IS166 homeodomain	0.245
PF01661	+	Macro domain	0.241
PF04343	+	Protein of unknown function, DUF488	0.241
PF00596	+	Class II Aldolase and Adducin N-terminal domain	0.237
PF07022	-	Bacteriophage CI repressor helix-turn-helix domain	0.234
PF02698	+	DUF218 domain	0.228
PF04865	+	Baseplate J-like protein	0.219
PF01432	+	Peptidase family M3	0.213
PF03141	-	Putative S-adenosyl-L-methionine-dependent methyltransferase	0.201
PF06147	-	Protein of unknown function (DUF968)	0.198
PF03724	+	META domain	0.194
PF03306	-	Alpha-acetolactate decarboxylase	0.193
PF06964	+	Alpha-L-arabinofuranosidase C-terminus	0.189
PF09223	-	YodA lipocalin-like domain	0.189
PF05840	-	Bacteriophage replication gene A protein (GPA)	0.186
PF13440	+	Polysaccharide biosynthesis protein	0.185
PF08503	+	Tetrahydrodipicolinate succinyltransferase N-terminal	0.179
PF02653	+	Branched-chain amino acid transport system / permease component	0.177
PF03050	+	Transposase IS66 family	0.172
PF13551	+	Winged helix-turn helix	0.160
PF13727	+	CoA-binding domain	0.156
PF05402	+	Coenzyme PQQ synthesis protein D (PqqD)	0.155
PF03063	-	Prismane/CO dehydrogenase family	0.154
PF04960	+	Glutaminase	0.151
PF13354	-	Beta-lactamase enzyme family	0.151
PF14542	+	GCN5-related N-acetyl-transferase	0.151
PF04268	+	Sarcosine oxidase, gamma subunit family	0.148
PF12679	+	ABC-2 family transporter protein	0.140
PF01609	+	Transposase DDE domain	0.127
PF01391	+	Collagen triple helix repeat (20 copies)	0.126
PF02274	+	Amidinotransferase	0.123
PF02764	+	Diphtheria toxin, T domain	0.122
PF01324	+	Diphtheria toxin, R domain	0.122
PF02763	+	Diphtheria toxin, C domain	0.122
PF03783	-	Curli production assembly/transport component CsgG	0.114
PF12846	+	AAA-like domain	0.112
PF01507	+	Phosphoadenosine phosphosulfate reductase family	0.110
PF03616	-	Sodium/glutamate symporter	0.109
PF07504	-	Fungalysin/Thermolysin Propeptide Motif	0.107
PF13426	-	PAS domain	0.106

PF00201	-	UDP-glucoronosyl and UDP-glucosyl transferase	0.105
PF03744	+	6-carboxyhexanoate--CoA ligase	0.100
PF12945	-	Flagellar protein YcgR	0.100
PF02423	+	Ornithine cyclodeaminase/mu-crystallin family	0.098
PF00909	-	Ammonium Transporter Family	0.098
PF13428	+	Tetratricopeptide repeat	0.094
PF10417	-	C-terminal domain of 1-Cys peroxiredoxin	0.094
PF02661	-	Fic/DOC family	0.089
PF01470	-	Pyroglutamyl peptidase	0.085
PF11367	-	Protein of unknown function (DUF3168)	0.083
PF13470	+	PIN domain	0.078
PF14265	+	Domain of unknown function (DUF4355)	0.077
PF06772	+	Bacterial low temperature requirement A protein (LtrA)	0.077
PF14525	+	AraC-binding-like domain	0.076
PF12895	-	Anaphase-promoting complex, cyclosome, subunit 3	0.068
PF13086	+	AAA domain	0.065
PF03354	-	Phage Terminase	0.062
PF10604	-	Polyketide cyclase / dehydrase and lipid transport	0.062
PF01384	-	Phosphate transporter family	0.059
PF13686	+	DsrE/DsrF/DrsH-like family	0.058
PF14529	-	Endonuclease-reverse transcriptase	0.057
PF08392	+	FAE1/Type III polyketide synthase-like protein	0.055
PF13348	-	Tyrosine phosphatase family C-terminal region	0.055
PF08666	+	SAF domain	0.053
PF00496	-	Bacterial extracellular solute-binding proteins, family 5 Middle	0.052
PF01510	-	N-acetylmuramoyl-L-alanine amidase	0.050
PF05889	-	Soluble liver antigen/liver pancreas antigen (SLA/LP autoantigen)	0.045
PF02245	+	Methylpurine-DNA glycosylase (MPG)	0.043
PF10979	-	Protein of unknown function (DUF2786)	0.043
PF01633	-	Choline/ethanolamine kinase	0.037
PF12437	+	Glutamine synthetase type III N terminal	0.036
PF02514	+	CobN/Magnesium Chelatase	0.035
PF08439	+	Oligopeptidase F	0.027
PF02275	-	Linear amide C-N hydrolases, choloylglycine hydrolase family	0.027
PF01740	-	STAS domain	0.024
PF08448	-	PAS fold	0.024
PF13542	-	Helix-turn-helix domain of transposase family ISL3	0.023
PF13424	+	Tetratricopeptide repeat	0.022
PF14659	-	Phage integrase, N-terminal SAM-like domain	0.020
PF05057	-	Putative serine esterase (DUF676)	0.020
PF06114	-	Domain of unknown function (DUF955)	0.019
PF00903	-	Glyoxalase/Bleomycin resistance protein/Dioxygenase superfamily	0.017
PF13683	-	Integrase core domain	0.017
PF09515	-	Thiamine transporter protein (Thia_YuaJ)	0.013
PF07664	+	Ferrous iron transport protein B C terminus	0.012
PF10066	+	Uncharacterized conserved protein (DUF2304)	0.010
PF06486	-	Protein of unknown function (DUF1093)	0.010
PF08453	-	Peptidase family M9 N-terminal	0.008

PF00390	-	Malic enzyme, N-terminal domain	0.003
PF03949	-	Malic enzyme, NAD binding domain	0.003
PF01613	-	Flavin reductase like domain	-0.001
PF05076	-	Suppressor of fused protein (SUFU)	-0.003
PF04313	+	Type I restriction enzyme R protein N terminus (HSDR_N)	-0.003
PF03446	-	NAD binding domain of 6-phosphogluconate dehydrogenase	-0.005
PF13408	-	Recombinase zinc beta ribbon domain	-0.006
PF03741	-	Integral membrane protein TerC family	-0.016
PF13772	-	AIG2-like family	-0.016
PF10662	-	Ethanolamine utilisation - propanediol utilisation	-0.024
PF00239	-	Resolvase, N terminal domain	-0.029
PF01326	-	Pyruvate phosphate dikinase, PEP/pyruvate binding domain	-0.030
PF10727	+	Rossmann-like domain	-0.031
PF00762	-	Ferrochelatase	-0.036
PF00301	-	Rubredoxin	-0.043
PF07476	-	Methylaspartate ammonia-lyase C-terminus	-0.045
PF06821	-	Serine hydrolase	-0.046
PF03083	+	Sugar efflux transporter for intercellular exchange	-0.047
PF13536	-	Multidrug resistance efflux transporter	-0.049
PF03681	-	Uncharacterised protein family (UPF0150)	-0.051
PF04023	-	FeoA domain	-0.055
PF01850	-	PIN domain	-0.056
PF07695	-	7TM diverse intracellular signalling	-0.057
PF13566	+	Domain of unknown function (DUF4130	-0.057
PF01512	-	Respiratory-chain NADH dehydrogenase 51 Kd subunit	-0.057
PF08877	-	MepB protein	-0.057
PF13370	-	4Fe-4S single cluster domain	-0.064
PF02082	-	Transcriptional regulator	-0.064
PF13813	-	Membrane bound O-acyl transferase family	-0.082
PF00350	-	Dynamin family	-0.090
PF02535	-	ZIP Zinc transporter	-0.090
PF13412	-	Winged helix-turn-helix DNA-binding	-0.100
PF01728	-	FtsJ-like methyltransferase	-0.104
PF09827	+	CRISPR associated protein Cas2	-0.106
PF12705	-	PD-(D/E)XK nuclease superfamily	-0.118
PF13173	-	AAA domain	-0.123
PF01745	-	Isopentenyl transferase	-0.123
PF02541	-	Ppx/GppA phosphatase family	-0.124
PF03990	-	Domain of unknown function (DUF348)	-0.133
PF12229	-	Putative peptidoglycan binding domain	-0.154
PF13452	-	N-terminal half of MaoC dehydratase	-0.166
PF06271	+	RDD family	-0.177
PF10947	-	Protein of unknown function (DUF2628)	-0.179
PF01425	-	Amidase	-0.222
PF13083	-	KH domain	-0.229
PF03309	-	Type III pantothenate kinase	-0.289

Phenotype: D-Sorbitol (Growth: Sugar), Predictor: phypat

Pfam	class	Pfam_desc	cor
PF06923	+	Glucitol operon activator protein (GutM)	0.715
PF07663	+	Sorbitol phosphotransferase enzyme II C-terminus	0.695
PF03612	+	Sorbitol phosphotransferase enzyme II N-terminus	0.695
PF03170	+	Bacterial cellulose synthase subunit	0.558
PF03812	+	2-keto-3-deoxygluconate permease	0.524
PF10685	+	Stress-induced bacterial acidophilic repeat motif	0.517
PF01320	+	Colicin immunity protein / pyocin immunity protein	0.494
PF01337	+	Barstar (barnase inhibitor)	0.489
PF02694	+	Uncharacterised BCR, YnfA/UPF0060 family	0.467
PF03621	+	MbtH-like protein	0.466
PF01232	+	Mannitol dehydrogenase Rossmann domain	0.455
PF05171	+	Haemin-degrading HemS.ChuX domain	0.451
PF03594	+	Benzoate membrane transport protein	0.439
PF04951	+	D-aminopeptidase	0.418
PF09347	+	Domain of unknown function (DUF1989)	0.383
PF13577	+	SnoaL-like domain	0.365
PF01564	+	Spermine/spermidine synthase	0.359
PF14031	+	Putative serine dehydratase domain	0.357
PF03649	+	Uncharacterised protein family (UPF0014)	0.352
PF12708	+	Pectate lyase superfamily protein	0.344
PF00847	+	AP2 domain	0.335
PF01526	+	Tn3 transposase DDE domain	0.303
PF14502	+	Helix-turn-helix domain	0.261
PF01175	+	Urocanase	0.248
PF06750	+	Bacterial Peptidase A24 N-terminal domain	0.233
PF11755	+	Protein of unknown function (DUF3311)	0.228
PF01391	+	Collagen triple helix repeat (20 copies)	0.195
PF04266	-	ASCH domain	0.133
PF09851	+	Short C-terminal domain	0.095
PF13023	-	HD domain	0.087
PF02690	-	Na+/Pi-cotransporter	0.087
PF13432	-	Tetratricopeptide repeat	0.079
PF14559	-	Tetratricopeptide repeat	0.067
PF08003	-	Protein of unknown function (DUF1698)	0.060
PF10415	-	Fumarase C C-terminus	0.055
PF06969	-	HemN C-terminal domain	0.031
PF01809	-	Haemolytic domain	-0.057
PF13394	-	4Fe-4S single cluster domain	-0.063
PF01609	-	Transposase DDE domain	-0.066
PF01807	-	CHC2 zinc finger	-0.092
PF00625	-	Guanylate kinase	-0.092
PF13519	-	von Willebrand factor type A domain	-0.099
PF07726	-	ATPase family associated with various cellular activities (AAA)	-0.119
PF02677	-	Uncharacterized BCR, COG1636	-0.119
PF09397	-	Ftsk gamma domain	-0.132
PF13672	-	Protein phosphatase 2C	-0.141
PF01867	-	CRISPR associated protein Cas1	-0.160

PF00025	-	ADP-ribosylation factor family	-0.162
PF00830	-	Ribosomal L28 family	-0.163
PF02384	-	N-6 DNA Methylase	-0.175
PF00829	-	Ribosomal prokaryotic L21 protein	-0.192
PF04011	-	LemA family	-0.205
PF01148	-	Cytidylyltransferase family	-0.221
PF01580	-	FtsK/SpoIIIE family	-0.239
PF00071	-	Ras family	-0.244
PF01268	-	Formate--tetrahydrofolate ligase	-0.269
PF00209	-	Sodium:neurotransmitter symporter family	-0.275
PF01637	-	Archaeal ATPase	-0.321

Phenotype: D-Sorbitol (Growth: Sugar), Predictor: phypat+PGL

Pfam	class	Pfam_desc	cor
PF06923	+	Glucitol operon activator protein (GutM)	0.715
PF03612	+	Sorbitol phosphotransferase enzyme II N-terminus	0.695
PF03812	+	2-keto-3-deoxygluconate permease	0.524
PF02694	+	Uncharacterised BCR, YnfA/UPF0060 family	0.467
PF05171	+	Haemin-degrading HemS.ChuX domain	0.451
PF04951	+	D-aminopeptidase	0.418
PF07007	+	Protein of unknown function (DUF1311)	0.416
PF05336	+	Domain of unknown function (DUF718)	0.405
PF02056	+	Family 4 glycosyl hydrolase	0.388
PF05063	+	MT-A70	0.366
PF13577	+	SnoaL-like domain	0.365
PF04794	+	YdjC-like protein	0.351
PF02955	+	Prokaryotic glutathione synthetase, ATP-grasp domain	0.350
PF06067	+	Domain of unknown function (DUF932)	0.350
PF12708	+	Pectate lyase superfamily protein	0.344
PF00847	+	AP2 domain	0.335
PF03972	+	MmgE/PrpD family	0.316
PF09224	+	Domain of unknown function (DUF1961)	0.314
PF01526	+	Tn3 transposase DDE domain	0.303
PF00295	+	Glycosyl hydrolases family 28	0.302
PF01894	+	Uncharacterised protein family UPF0047	0.294
PF14549	+	DNA-binding transcriptional regulator Cro	0.266
PF07732	-	Multicopper oxidase	0.264
PF01161	+	Phosphatidylethanolamine-binding protein	0.261
PF13728	+	F plasmid transfer operon protein	0.253
PF03606	+	C4-dicarboxylate anaerobic carrier	0.251
PF01175	+	Urocanase	0.248
PF05534	+	HicB family	0.240
PF06750	+	Bacterial Peptidase A24 N-terminal domain	0.233
PF03412	+	Peptidase C39 family	0.233
PF00875	+	DNA photolyase	0.229
PF00245	+	Alkaline phosphatase	0.229
PF00441	+	Acyl-CoA dehydrogenase, C-terminal domain	0.229
PF13814	+	Replication-relaxation	0.228

PF00190	+	Cupin	0.226
PF00221	+	Aromatic amino acid lyase	0.224
PF08433	+	Chromatin associated protein KTI12	0.216
PF06042	+	Bacterial protein of unknown function (DUF925)	0.212
PF13521	+	AAA domain	0.208
PF11459	+	Protein of unknwon function (DUF2893)	0.208
PF04616	+	Glycosyl hydrolases family 43	0.206
PF05935	+	Arylsulfotransferase (ASST)	0.202
PF05015	+	Plasmid maintenance system killer protein	0.171
PF07411	+	Domain of unknown function (DUF1508)	0.169
PF12169	-	DNA polymerase III subunits gamma and tau domain III	0.168
PF00931	+	NB-ARC domain	0.163
PF08922	+	Domain of unknown function (DUF1905)	0.163
PF09314	+	Domain of unknown function (DUF1972)	0.151
PF02368	-	Bacterial Ig-like domain (group 2)	0.149
PF13379	-	NMT1-like family	0.146
PF14145	+	YrhK-like protein	0.145
PF04143	-	Sulphur transport	0.143
PF01891	+	Cobalt uptake substrate-specific transmembrane region	0.135
PF02534	-	Type IV secretory system Conjugative DNA transfer	0.133
PF09423	+	PhoD-like phosphatase	0.132
PF09588	+	YqaJ-like viral recombinase domain	0.125
PF05065	-	Phage capsid family	0.081
PF08415	-	Nonribosomal peptide synthase	0.074
PF06508	-	Queuosine biosynthesis protein QueC	0.069
PF10415	-	Fumarase C C-terminus	0.055
PF01018	-	GTP1/OBG	0.045
PF14338	+	Mrr N-terminal domain	0.031
PF02502	-	Ribose/Galactose Isomerase	0.023
PF04685	+	Protein of unknown function, DUF608	-0.007
PF08719	-	Domain of unknown function (DUF1768)	-0.015
PF00468	-	Ribosomal protein L34	-0.019
PF09903	+	Uncharacterized protein conserved in bacteria (DUF2130)	-0.019
PF00041	-	Fibronectin type III domain	-0.034
PF09997	-	Predicted membrane protein (DUF2238)	-0.043
PF01809	-	Haemolytic domain	-0.057
PF11907	+	Domain of unknown function (DUF3427)	-0.073
PF09704	-	CRISPR-associated protein (Cas_Cas5)	-0.076
PF00092	-	von Willebrand factor type A domain	-0.087
PF00829	-	Ribosomal prokaryotic L21 protein	-0.192
PF04011	-	LemA family	-0.205

Phenotype: D-Xylose (Growth: Sugar), Predictor: phypat

Pfam	class	Pfam_desc	cor
PF04295	+	D-galactarate dehydratase / Altronate hydrolase, C terminus	0.613
PF14310	+	Fibronectin type III-like domain	0.566
PF01011	+	PQQ enzyme repeat	0.556
PF09163	+	Formate dehydrogenase N, transmembrane	0.546

PF06995	+	Phage P2 GpU	0.531
PF05489	+	Phage Tail Protein X	0.514
PF03825	+	Nucleoside H+ symporter	0.504
PF02702	+	Osmosensitive K+ channel His kinase sensor domain	0.504
PF02610	+	L-arabinose isomerase	0.499
PF11762	+	L-arabinose isomerase C-terminal domain	0.499
PF04984	+	Phage tail sheath protein	0.496
PF01232	+	Mannitol dehydrogenase Rossmann domain	0.493
PF07944	+	Putative glycosyl hydrolase of unknown function (DUF1680)	0.487
PF06224	+	Winged helix DNA-binding domain	0.485
PF13229	+	Right handed beta helix region	0.485
PF11575	+	FhuF 2Fe-2S C-terminal domain	0.479
PF13493	+	Domain of unknown function (DUF4118)	0.476
PF13802	+	Galactose mutarotase-like	0.470
PF01564	+	Spermine/spermidine synthase	0.454
PF03707	+	Bacterial signalling protein N terminal repeat	0.451
PF06833	+	Malonate decarboxylase gamma subunit (MdcE)	0.449
PF06964	+	Alpha-L-arabinofuranosidase C-terminus	0.449
PF13391	+	HNH endonuclease	0.448
PF03786	+	D-mannonate dehydratase (UxuA)	0.446
PF08450	+	SMP-30/Gluconolaconase/LRE-like region	0.446
PF00596	+	Class II Aldolase and Adducin N-terminal domain	0.440
PF04616	+	Glycosyl hydrolases family 43	0.430
PF05402	+	Coenzyme PQQ synthesis protein D (PqqD)	0.428
PF09296	+	NADH pyrophosphatase-like rudimentary NUDIX domain	0.421
PF00543	+	Nitrogen regulatory protein P-II	0.421
PF07582	+	AP endonuclease family 2 C terminus	0.418
PF13556	+	PucR C-terminal helix-turn-helix domain	0.414
PF11922	+	Domain of unknown function (DUF3440)	0.409
PF11700	+	Vacuole effluxer Atg22 like	0.396
PF08401	+	Domain of unknown function (DUF1738)	0.388
PF04234	+	CopC domain	0.383
PF06056	+	Putative ATPase subunit of terminase (gpP-like)	0.366
PF13599	+	Pentapeptide repeats (9 copies)	0.361
PF01638	+	HxlR-like helix-turn-helix	0.360
PF02369	+	Bacterial Ig-like domain (group 1)	0.355
PF00145	+	C-5 cytosine-specific DNA methylase	0.344
PF02447	+	GntP family permease	0.316
PF12682	+	Flavodoxin	0.315
PF01548	+	Transposase	0.300
PF14436	+	Bacterial EndoU nuclease	0.285
PF12889	+	Protein of unknown function (DUF3829)	0.284
PF04951	+	D-aminopeptidase	0.277
PF04014	+	Antidote-toxin recognition MazE	0.275
PF00146	-	NADH dehydrogenase	0.263
PF12106	+	Colicin C terminal ribonuclease domain	0.261
PF05935	+	Arylsulfotransferase (ASST)	0.253
PF10547	+	P22_AR N-terminal domain	0.251

PF14269	+	Arylsulfotransferase (ASST)	0.226
PF10503	+	Esterase PHB depolymerase	0.220
PF07286	+	Protein of unknown function (DUF1445)	0.211
PF13310	+	Virulence protein RhuM family	0.208
PF01957	-	NfeD-like C-terminal, partner-binding	0.198
PF02594	-	Uncharacterised ACR, YggU family COG1872	0.186
PF02690	-	Na+/Pi-cotransporter	0.170
PF08020	+	Protein of unknown function (DUF1706)	0.165
PF14659	+	Phage integrase, N-terminal SAM-like domain	0.154
PF02770	-	Acyl-CoA dehydrogenase, middle domain	0.152
PF07702	-	UTRA domain	0.152
PF00441	-	Acyl-CoA dehydrogenase, C-terminal domain	0.138
PF02416	-	mttA/Hcf106 family	0.130
PF01906	-	Putative heavy-metal-binding	0.121
PF02491	-	SHS2 domain inserted in FTSA	0.100
PF12631	-	Catalytic cysteine-containing C-terminus of GTPase, MnmE	0.087
PF13857	-	Ankyrin repeats (many copies)	0.074
PF13191	-	AAA ATPase domain	0.073
PF13610	-	DDE domain	0.061
PF12961	-	Domain of Unknown Function with PDB structure (DUF3850)	0.056
PF00112	+	Papain family cysteine protease	0.054
PF07994	+	Myo-inositol-1-phosphate synthase	0.042
PF03641	-	Possible lysine decarboxylase	0.041
PF00355	-	Rieske [2Fe-2S] domain	0.038
PF02504	-	Fatty acid synthesis protein	0.028
PF00561	-	alpha/beta hydrolase fold	0.027
PF13855	-	Leucine rich repeat	0.022
PF01641	-	SelR domain	0.018
PF01368	-	DHH family	0.016
PF00210	-	Ferritin-like domain	0.014
PF01609	-	Transposase DDE domain	0.008
PF01728	-	FtsJ-like methyltransferase	0.007
PF00857	-	Isochorismatase family	-0.001
PF00444	-	Ribosomal protein L36	-0.012
PF06723	-	MreB/Mbl protein	-0.016
PF02867	-	Ribonucleotide reductase, barrel domain	-0.024
PF04480	-	Protein of unknown function (DUF559)	-0.024
PF00578	-	AhpC/TSA family	-0.027
PF03595	-	Voltage-dependent anion channel	-0.037
PF00550	-	Phosphopantetheine attachment site	-0.053
PF00675	-	Insulinase (Peptidase family M16)	-0.066
PF14253	-	Bacteriophage abortive infection AbiH	-0.073
PF01783	-	Ribosomal L32p protein family	-0.074
PF03102	-	NeuB family	-0.081
PF00149	-	Calcineurin-like phosphoesterase	-0.082
PF05525	-	Branched-chain amino acid transport protein	-0.082
PF02384	-	N-6 DNA Methylase	-0.088
PF13395	-	HNH endonuclease	-0.102

PF02223	-	Thymidylate kinase	-0.109
PF08207	-	Elongation factor P (EF-P) KOW-like domain	-0.122
PF05014	-	Nucleoside 2-deoxyribosyltransferase	-0.124
PF01171	-	PP-loop family	-0.129
PF04011	-	LemA family	-0.135
PF08000	-	Bacterial PH domain	-0.177
PF13495	-	Phage integrase, N-terminal SAM-like domain	-0.184
PF04079	-	Putative transcriptional regulators (Ypuh-like)	-0.195
PF02388	-	FemAB family	-0.226
PF01268	-	Formate--tetrahydrofolate ligase	-0.321
PF02361	-	Cobalt transport protein	-0.334
PF04296	-	Protein of unknown function (DUF448)	-0.356

Phenotype: D-Xylose (Growth: Sugar), Predictor: phypat+PGL

Pfam	class	Pfam_desc	cor
PF04295	+	D-galactarate dehydratase / Altronate hydrolase, C terminus	0.613
PF14310	+	Fibronectin type III-like domain	0.566
PF06995	+	Phage P2 GpU	0.531
PF05229	+	Spore Coat Protein U domain	0.528
PF06719	+	AraC-type transcriptional regulator N-terminus	0.519
PF04985	+	Phage tail tube protein FII	0.514
PF05489	+	Phage Tail Protein X	0.514
PF03825	+	Nucleoside H+ symporter	0.504
PF02610	+	L-arabinose isomerase	0.499
PF11762	+	L-arabinose isomerase C-terminal domain	0.499
PF04984	+	Phage tail sheath protein	0.496
PF00295	+	Glycosyl hydrolases family 28	0.496
PF01232	+	Mannitol dehydrogenase Rossmann domain	0.493
PF07944	+	Putative glycosyl hydrolase of unknown function (DUF1680)	0.487
PF13229	+	Right handed beta helix region	0.485
PF08125	+	Mannitol dehydrogenase C-terminal domain	0.484
PF13802	+	Galactose mutarotase-like	0.470
PF06964	+	Alpha-L-arabinofuranosidase C-terminus	0.449
PF13391	+	HNH endonuclease	0.448
PF08450	+	SMP-30/Gluconolaconase/LRE-like region	0.446
PF02694	+	Uncharacterised BCR, YnfA/UPF0060 family	0.438
PF04616	+	Glycosyl hydrolases family 43	0.430
PF07582	+	AP endonuclease family 2 C terminus	0.418
PF08379	+	Bacterial transglutaminase-like N-terminal region	0.418
PF05195	+	Aminopeptidase P, N-terminal domain	0.415
PF02614	+	Glucuronate isomerase	0.407
PF02659	+	Domain of unknown function DUF	0.403
PF00182	+	Chitinase class I	0.403
PF04168	+	A predicted alpha-helical domain with a conserved ER motif.	0.403
PF11198	+	Protein of unknown function (DUF2857)	0.402
PF01081	+	KDPG and KHG aldolase	0.400
PF02746	+	Mandelate racemase / muconate lactonizing enzyme, N-terminal domain	0.399
PF04230	+	Polysaccharide pyruvyl transferase	0.394

PF08401	+	Domain of unknown function (DUF1738)	0.388
PF14535	+	AMP-binding enzyme C-terminal domain	0.388
PF07196	+	Flagellin hook IN motif	0.387
PF04492	+	Bacteriophage replication protein O	0.387
PF00959	+	Phage lysozyme	0.376
PF01008	+	Initiation factor 2 subunit family	0.367
PF12708	+	Pectate lyase superfamily protein	0.362
PF02446	+	4-alpha-glucanotransferase	0.358
PF04657	+	Protein of unknown function, DUF606	0.347
PF00145	+	C-5 cytosine-specific DNA methylase	0.344
PF02839	+	Carbohydrate binding domain	0.335
PF11863	+	Protein of unknown function (DUF3383)	0.334
PF04257	+	Exodeoxyribonuclease V, gamma subunit	0.333
PF11175	+	Protein of unknown function (DUF2961)	0.321
PF08402	+	TOBE domain	0.321
PF00190	+	Cupin	0.320
PF02447	+	GntP family permease	0.316
PF02554	+	Carbon starvation protein CstA	0.316
PF04463	+	Protein of unknown function (DUF523)	0.310
PF13740	+	ACT domain	0.309
PF14549	+	DNA-binding transcriptional regulator Cro	0.306
PF01139	+	tRNA-splicing ligase RtcB	0.303
PF01548	+	Transposase	0.300
PF13539	+	D-alanyl-D-alanine carboxypeptidase	0.296
PF00135	+	Carboxylesterase family	0.294
PF12889	+	Protein of unknown function (DUF3829)	0.284
PF04551	+	GcpE protein	0.279
PF12696	+	TraM recognition site of TraD and TraG	0.278
PF04951	+	D-aminopeptidase	0.277
PF07876	+	Stress responsive A/B Barrel Domain	0.265
PF00657	+	GDSL-like Lipase/Acylhydrolase	0.260
PF13262	+	Protein of unknown function (DUF4054)	0.247
PF01730	+	UreF	0.243
PF12846	+	AAA-like domain	0.241
PF00848	-	Ring hydroxylating alpha subunit (catalytic domain)	0.241
PF02814	+	UreE urease accessory protein, N-terminal domain	0.241
PF12727	+	PBP superfamily domain	0.240
PF00699	+	Urease beta subunit	0.234
PF00547	+	Urease, gamma subunit	0.234
PF00449	+	Urease alpha-subunit, N-terminal domain	0.234
PF02534	+	Type IV secretory system Conjugative DNA transfer	0.225
PF01590	-	GAF domain	0.224
PF03969	-	AFG1-like ATPase	0.222
PF13088	+	BNR repeat-like domain	0.221
PF05194	+	UreE urease accessory protein, C-terminal domain	0.221
PF10503	+	Esterase PHB depolymerase	0.220
PF11367	-	Protein of unknown function (DUF3168)	0.220
PF00781	+	Diacylglycerol kinase catalytic domain	0.217

PF02152	-	Dihydroneopterin aldolase	0.213
PF11066	-	Protein of unknown function (DUF2867)	0.211
PF13189	+	Cytidylate kinase-like family	0.210
PF13484	-	4Fe-4S double cluster binding domain	0.210
PF13310	+	Virulence protein RhuM family	0.208
PF10417	-	C-terminal domain of 1-Cys peroxiredoxin	0.205
PF13620	+	Carboxypeptidase regulatory-like domain	0.204
PF05532	+	CsbD-like	0.202
PF13683	-	Integrase core domain	0.191
PF03781	-	Sulfatase-modifying factor enzyme 1	0.189
PF13348	-	Tyrosine phosphatase family C-terminal region	0.186
PF00665	-	Integrase core domain	0.182
PF00255	-	Glutathione peroxidase	0.181
PF10994	-	Protein of unknown function (DUF2817)	0.178
PF01427	-	D-ala-D-ala dipeptidase	0.174
PF02915	+	Rubrerythrin	0.172
PF03096	-	Ndr family	0.172
PF12500	-	TRSP domain C terminus to PRTase_2	0.170
PF01654	-	Bacterial Cytochrome Ubiquinol Oxidase	0.169
PF01520	-	N-acetylmuramoyl-L-alanine amidase	0.169
PF13192	+	Thioredoxin domain	0.168
PF13006	-	Insertion element 4 transposase N-terminal	0.166
PF08378	-	Nuclease-related domain	0.165
PF04854	+	Protein of unknown function, DUF624	0.163
PF00925	-	GTP cyclohydrolase II	0.156
PF02322	-	Cytochrome oxidase subunit II	0.154
PF14335	+	Domain of unknown function (DUF4391)	0.153
PF15617	-	C-C_Bond_Lyase of the TIM-Barrel fold	0.153
PF00420	-	NADH-ubiquinone/plastoquinone oxidoreductase chain 4L	0.152
PF09997	-	Predicted membrane protein (DUF2238)	0.149
PF08843	+	Nucleotidyl transferase of unknown function (DUF1814)	0.145
PF00201	-	UDP-glucoronosyl and UDP-glucosyl transferase	0.143
PF03417	+	Acyl-coenzyme A:6-aminopenicillanic acid acyl-transferase	0.139
PF11870	-	Domain of unknown function (DUF3390)	0.139
PF03050	-	Transposase IS66 family	0.136
PF05154	+	TM2 domain	0.131
PF02416	-	mttA/Hcf106 family	0.130
PF08643	-	Fungal family of unknown function (DUF1776)	0.123
PF13455	-	Meiotically up-regulated gene 113	0.115
PF08751	-	TrwC relaxase	0.109
PF07719	-	Tetratricopeptide repeat	0.109
PF14056	+	Domain of unknown function (DUF4250)	0.108
PF00908	-	dTDP-4-dehydrorhamnose 3,5-epimerase	0.108
PF00545	-	ribonuclease	0.101
PF00296	-	Luciferase-like monooxygenase	0.096
PF00350	-	Dynamin family	0.094
PF13166	+	AAA domain	0.091
PF10418	+	Iron-sulfur cluster binding domain of dihydroorotate dehydrogenase B	0.087

PF05729	-	NACHT domain	0.085
PF10881	-	Protein of unknown function (DUF2726)	0.084
PF01593	-	Flavin containing amine oxidoreductase	0.080
PF02129	-	X-Pro dipeptidyl-peptidase (S15 family)	0.079
PF02275	+	Linear amide C-N hydrolases, choloylglycine hydrolase family	0.078
PF13424	-	Tetratricopeptide repeat	0.076
PF00092	-	von Willebrand factor type A domain	0.074
PF01773	-	Na+ dependent nucleoside transporter N-terminus	0.071
PF07662	-	Na+ dependent nucleoside transporter C-terminus	0.071
PF13467	-	Ribbon-helix-helix domain	0.066
PF08388	-	Group II intron, maturase-specific domain	0.064
PF13610	-	DDE domain	0.061
PF04454	+	Encapsulating protein for peroxidase	0.059
PF00994	-	Probable molybdopterin binding domain	0.059
PF01925	-	Sulfite exporter TauE/SafE	0.059
PF03205	-	Molybdopterin guanine dinucleotide synthesis protein B	0.053
PF03611	-	PTS system sugar-specific permease component	0.048
PF13408	-	Recombinase zinc beta ribbon domain	0.039
PF00801	-	PKD domain	0.035
PF05708	-	Orthopoxvirus protein of unknown function (DUF830)	0.033
PF08840	-	BAAT / Acyl-CoA thioester hydrolase C terminal	0.022
PF03444	+	Winged helix-turn-helix transcription repressor, HrcA DNA-binding	0.020
PF03432	-	Relaxase/Mobilisation nuclease domain	0.020
PF01641	-	SelR domain	0.018
PF00210	-	Ferritin-like domain	0.014
PF01555	-	DNA methylase	0.012
PF01609	-	Transposase DDE domain	0.008
PF13635	+	Domain of unknown function (DUF4143)	0.007
PF14256	+	YwiC-like protein	0.005
PF03916	-	Polysulphide reductase, NrfD	0.004
PF07726	-	ATPase family associated with various cellular activities (AAA)	-0.002
PF13791	+	Sigma factor regulator C-terminal	-0.004
PF03610	-	PTS system fructose IIA component	-0.005
PF12206	+	Domain of unknown function (DUF3599)	-0.008
PF00444	-	Ribosomal protein L36	-0.012
PF00565	-	Staphylococcal nuclease homologue	-0.014
PF11997	-	Domain of unknown function (DUF3492)	-0.018
PF14588	-	YjgF/chorismate_mutase-like, putative endoribonuclease	-0.023
PF02867	-	Ribonucleotide reductase, barrel domain	-0.024
PF12895	-	Anaphase-promoting complex, cyclosome, subunit 3	-0.024
PF04480	-	Protein of unknown function (DUF559)	-0.024
PF13672	-	Protein phosphatase 2C	-0.031
PF13766	-	2-enoyl-CoA Hydratase C-terminal region	-0.033
PF01633	-	Choline/ethanolamine kinase	-0.036
PF03595	-	Voltage-dependent anion channel	-0.037
PF07523	-	Bacterial Ig-like domain (group 3)	-0.040
PF04466	-	Phage terminase large subunit	-0.041
PF03965	+	Penicillinase repressor	-0.043

PF06769	-	Plasmid encoded toxin Txe	-0.043
PF03971	-	Monomeric isocitrate dehydrogenase	-0.043
PF04023	-	FeoA domain	-0.051
PF01867	-	CRISPR associated protein Cas1	-0.053
PF08706	-	D5 N terminal like	-0.059
PF01470	-	Pyroglutamyl peptidase	-0.064
PF00675	-	Insulinase (Peptidase family M16)	-0.066
PF02502	-	Ribose/Galactose Isomerase	-0.068
PF01783	-	Ribosomal L32p protein family	-0.074
PF13552	+	Protein of unknown function (DUF4127)	-0.075
PF05525	-	Branched-chain amino acid transport protein	-0.082
PF14657	+	AP2-like DNA-binding integrase domain	-0.084
PF04392	-	ABC transporter substrate binding protein	-0.098
PF13128	+	Protein of unknown function (DUF3954)	-0.109
PF05014	-	Nucleoside 2-deoxyribosyltransferase	-0.124
PF06152	-	Phage minor capsid protein 2	-0.165
PF08000	-	Bacterial PH domain	-0.177
PF13495	-	Phage integrase, N-terminal SAM-like domain	-0.184
PF05066	-	HB1, ASXL, restriction endonuclease HTH domain	-0.231
PF02361	-	Cobalt transport protein	-0.334

Phenotype: Esculin hydrolysis (Growth: Sugar), Predictor: phypat

Pfam	class	Pfam_desc	cor
PF13345	+	Domain of unknown function (DUF4098)	0.554
PF02449	+	Beta-galactosidase	0.510
PF06993	+	Protein of unknown function (DUF1304)	0.507
PF02065	+	Melibiase	0.490
PF04616	+	Glycosyl hydrolases family 43	0.487
PF08533	+	Beta-galactosidase C-terminal domain	0.485
PF07745	+	Glycosyl hydrolase family 53	0.485
PF00232	+	Glycosyl hydrolase family 1	0.472
PF12464	+	Maltose acetyltransferase	0.445
PF03306	+	Alpha-acetolactate decarboxylase	0.439
PF01055	+	Glycosyl hydrolases family 31	0.435
PF01915	+	Glycosyl hydrolase family 3 C-terminal domain	0.429
PF05870	+	Phenolic acid decarboxylase (PAD)	0.428
PF05592	+	Bacterial alpha-L-rhamnosidase	0.425
PF00150	+	Cellulase (glycosyl hydrolase family 5)	0.417
PF11975	+	Family 4 glycosyl hydrolase C-terminal domain	0.417
PF02056	+	Family 4 glycosyl hydrolase	0.404
PF02074	+	Carboxypeptidase Taq (M32) metallopeptidase	0.401
PF02588	+	Uncharacterized BCR, YitT family COG1284	0.400
PF12459	+	D-Ala-teichoic acid biosynthesis protein	0.399
PF03932	+	CutC family	0.387
PF14310	+	Fibronectin type III-like domain	0.383
PF04024	+	PspC domain	0.365
PF11762	+	L-arabinose isomerase C-terminal domain	0.365
PF03390	+	2-hydroxycarboxylate transporter family	0.318

PF00704	+	Glycosyl hydrolases family 18	0.317
PF00135	+	Carboxylesterase family	0.317
PF07081	+	Protein of unknown function (DUF1349)	0.309
PF08840	+	BAAT / Acyl-CoA thioester hydrolase C terminal	0.306
PF14526	+	Integron-associated effector binding protein	0.306
PF10662	+	Ethanolamine utilisation - propanediol utilisation	0.298
PF00251	+	Glycosyl hydrolases family 32 N-terminal domain	0.289
PF06347	+	Bacterial SH3 domain	0.270
PF05336	+	Domain of unknown function (DUF718)	0.269
PF09314	+	Domain of unknown function (DUF1972)	0.266
PF00201	+	UDP-glucoronosyl and UDP-glucosyl transferase	0.241
PF04239	+	Protein of unknown function (DUF421)	0.241
PF01008	+	Initiation factor 2 subunit family	0.237
PF13828	+	Domain of unknown function (DUF4190)	0.234
PF07885	+	Ion channel	0.219
PF10101	+	Predicted membrane protein (DUF2339)	0.186
PF10091	+	Putative glucoamylase	0.178
PF04371	+	Porphyromonas-type peptidyl-arginine deiminase	0.157
PF00990	+	GGDEF domain	0.141
PF04235	+	Protein of unknown function (DUF418)	0.120
PF05973	+	Phage derived protein Gp49-like (DUF891)	0.101
PF13278	-	Putative amidotransferase	0.099
PF01661	-	Macro domain	0.089
PF00282	+	Pyridoxal-dependent decarboxylase conserved domain	0.086
PF01909	+	Nucleotidyltransferase domain	0.083
PF03435	+	Saccharopine dehydrogenase	0.057
PF01769	+	Divalent cation transporter	0.024
PF02580	-	D-Tyr-tRNA(Tyr) deacylase	0.007
PF10031	-	Small integral membrane protein (DUF2273)	-0.004
PF00639	-	PPIC-type PPIASE domain	-0.006
PF13413	-	Helix-turn-helix domain	-0.009
PF00239	-	Resolvase, N terminal domain	-0.012
PF08708	-	Primase C terminal 1 (PriCT-1)	-0.022
PF01451	-	Low molecular weight phosphotyrosine protein phosphatase	-0.024
PF02110	-	Hydroxyethylthiazole kinase family	-0.026
PF14691	-	Dihydroprymidine dehydrogenase domain II, 4Fe-4S cluster	-0.061
PF13518	-	Helix-turn-helix domain	-0.063
PF08774	-	VRR-NUC domain	-0.076
PF13542	-	Helix-turn-helix domain of transposase family ISL3	-0.086
PF13088	-	BNR repeat-like domain	-0.097
PF13412	-	Winged helix-turn-helix DNA-binding	-0.100
PF00665	-	Integrase core domain	-0.101
PF01205	-	Uncharacterized protein family UPF0029	-0.106
PF05437	-	Branched-chain amino acid transport protein (AzlD)	-0.109
PF10340	-	Protein of unknown function (DUF2424)	-0.120
PF03432	-	Relaxase/Mobilisation nuclease domain	-0.121
PF00903	-	Glyoxalase/Bleomycin resistance protein/Dioxygenase superfamily	-0.124
PF03180	-	NLPA lipoprotein	-0.124

PF00334	-	Nucleoside diphosphate kinase	-0.131
PF13274	-	Protein of unknown function (DUF4065)	-0.132
PF13186	-	Iron-sulfur cluster-binding domain	-0.132
PF01610	-	Transposase	-0.132
PF14711	-	Respiratory nitrate reductase beta C-terminal	-0.149
PF01764	-	Lipase (class 3)	-0.162
PF11066	-	Protein of unknown function (DUF2867)	-0.162
PF07670	-	Nucleoside recognition	-0.165
PF14690	-	zinc-finger of transposase IS204/IS1001/IS1096/IS1165	-0.166
PF02498	-	BRO family, N-terminal domain	-0.170
PF04140	-	Isoprenylcysteine carboxyl methyltransferase (ICMT) family	-0.180
PF00764	-	Arginosuccinate synthase	-0.196
PF00725	-	3-hydroxyacyl-CoA dehydrogenase, C-terminal domain	-0.211
PF03720	-	UDP-glucose/GDP-mannose dehydrogenase family, UDP binding domain	-0.214
PF10417	-	C-terminal domain of 1-Cys peroxiredoxin	-0.222
PF14281	-	PD-(D/E)XK nuclease superfamily	-0.228
PF02152	-	Dihydroneopterin aldolase	-0.230
PF02812	-	Glu/Leu/Phe/Val dehydrogenase, dimerisation domain	-0.230
PF02626	-	Allophanate hydrolase subunit 2	-0.233
PF02730	-	Aldehyde ferredoxin oxidoreductase, N-terminal domain	-0.235
PF01314	-	Aldehyde ferredoxin oxidoreductase, domains 2 & 3	-0.235
PF02682	-	Allophanate hydrolase subunit 1	-0.243
PF14698	-	Argininosuccinate lyase C-terminal	-0.246
PF02652	-	L-lactate permease	-0.255
PF01975	-	Survival protein SurE	-0.281
PF00766	-	Electron transfer flavoprotein FAD-binding domain	-0.284
PF02511	-	Thymidylate synthase complementing protein	-0.285
PF05145	-	Putative ammonia monooxygenase	-0.293
PF01012	-	Electron transfer flavoprotein domain	-0.295
PF02550	-	Acetyl-CoA hydrolase/transferase N-terminal domain	-0.298
PF00745	-	Glutamyl-tRNAGlu reductase, dimerisation domain	-0.298
PF02515	-	CoA-transferase family III	-0.307
PF01235	-	Sodium:alanine symporter family	-0.311
PF13247	-	4Fe-4S dicluster domain	-0.311
PF14697	-	4Fe-4S dicluster domain	-0.320
PF03900	-	Porphobilinogen deaminase, C-terminal domain	-0.320
PF00490	-	Delta-aminolevulinic acid dehydratase	-0.320
PF02683	-	Cytochrome C biogenesis protein transmembrane region	-0.321
PF12801	-	4Fe-4S binding domain	-0.327
PF00899	-	ThiF family	-0.329
PF01379	-	Porphobilinogen deaminase, dipyromethane cofactor binding domain	-0.333
PF00809	-	Pterin binding enzyme	-0.333
PF04143	-	Sulphur transport	-0.337
PF06968	-	Biotin and Thiamin Synthesis associated domain	-0.351
PF03971	-	Monomeric isocitrate dehydrogenase	-0.355
PF01288	-	7,8-dihydro-6-hydroxymethylpterin-pyrophosphokinase (HPPK)	-0.373
PF02541	-	Ppx/GppA phosphatase family	-0.452

Phenotype: Esculin hydrolysis (Growth: Sugar), Predictor: phypat+PGL

Pfam	class	Pfam_desc	cor
PF13345	+	Domain of unknown function (DUF4098)	0.554
PF02449	+	Beta-galactosidase	0.510
PF06993	+	Protein of unknown function (DUF1304)	0.507
PF02065	+	Melibiase	0.490
PF04616	+	Glycosyl hydrolases family 43	0.487
PF00232	+	Glycosyl hydrolase family 1	0.472
PF08503	+	Tetrahydrodipicolinate succinyltransferase N-terminal	0.453
PF13802	+	Galactose mutarotase-like	0.453
PF02903	+	Alpha amylase, N-terminal ig-like domain	0.439
PF03306	+	Alpha-acetolactate decarboxylase	0.439
PF12558	+	ATP-binding cassette cobalt transporter	0.428
PF08713	+	DNA alkylation repair enzyme	0.426
PF00150	+	Cellulase (glycosyl hydrolase family 5)	0.417
PF07697	+	7TM-HD extracellular	0.399
PF03932	+	CutC family	0.387
PF14310	+	Fibronectin type III-like domain	0.383
PF09966	+	Uncharacterized protein conserved in bacteria (DUF2200)	0.379
PF14501	+	GHKL domain	0.372
PF04024	+	PspC domain	0.365
PF04041	+	Domain of unknown function (DUF377)	0.340
PF08840	+	BAAT / Acyl-CoA thioester hydrolase C terminal	0.306
PF14526	+	Integron-associated effector binding protein	0.306
PF10662	+	Ethanolamine utilisation - propanediol utilisation	0.298
PF14568	+	SMI1-KNR4 cell-wall	0.286
PF04456	+	Protein of unknown function (DUF503)	0.285
PF00722	+	Glycosyl hydrolases family 16	0.285
PF13344	+	Haloacid dehalogenase-like hydrolase	0.280
PF01361	+	Tautomerase enzyme	0.274
PF06347	+	Bacterial SH3 domain	0.270
PF14689	+	Sensor_kinase_SpoOB-type, alpha-helical domain	0.270
PF05336	+	Domain of unknown function (DUF718)	0.269
PF09314	+	Domain of unknown function (DUF1972)	0.266
PF01944	+	Integral membrane protein DUF95	0.247
PF12641	+	Flavodoxin domain	0.246
PF13559	+	Domain of unknown function (DUF4129)	0.243
PF04239	+	Protein of unknown function (DUF421)	0.241
PF13828	+	Domain of unknown function (DUF4190)	0.234
PF02916	+	DNA polymerase processivity factor	0.234
PF00614	+	Phospholipase D Active site motif	0.222
PF13602	+	Zinc-binding dehydrogenase	0.220
PF07885	+	Ion channel	0.219
PF03663	+	Glycosyl hydrolase family 76	0.205
PF03729	+	Short repeat of unknown function (DUF308)	0.201
PF05116	-	Sucrose-6F-phosphate phosphohydrolase	0.200
PF03951	+	Glutamine synthetase, beta-Grasp domain	0.194
PF13239	+	2TM domain	0.193

PF01987	+	Mitochondrial biogenesis AIM24	0.192
PF13578	+	Methyltransferase domain	0.190
PF10091	+	Putative glucoamylase	0.178
PF09346	+	SMI1 / KNR4 family (SUKH-1)	0.177
PF02784	+	Pyridoxal-dependent decarboxylase, pyridoxal binding domain	0.160
PF08592	+	Domain of unknown function (DUF1772)	0.158
PF04371	+	Porphyromonas-type peptidyl-arginine deiminase	0.157
PF09851	+	Short C-terminal domain	0.151
PF00255	+	Glutathione peroxidase	0.150
PF12895	-	Anaphase-promoting complex, cyclosome, subunit 3	0.149
PF04326	+	Divergent AAA domain	0.149
PF00278	+	Pyridoxal-dependent decarboxylase, C-terminal sheet domain	0.147
PF13704	+	Glycosyl transferase family 2	0.147
PF00563	+	EAL domain	0.145
PF06961	-	Protein of unknown function (DUF1294)	0.142
PF00990	+	GGDEF domain	0.141
PF01113	+	Dihydrodipicolinate reductase, N-terminus	0.134
PF06180	-	Cobalt chelatase (CbiK)	0.129
PF06224	+	Winged helix DNA-binding domain	0.125
PF04235	+	Protein of unknown function (DUF418)	0.120
PF05045	+	Rhamnan synthesis protein F	0.118
PF02030	-	Hypothetical lipoprotein (MG045 family)	0.116
PF03190	+	Protein of unknown function, DUF255	0.115
PF02534	+	Type IV secretory system Conjugative DNA transfer	0.115
PF12320	-	Type 5 capsule protein repressor C-terminal domain	0.115
PF02230	+	Phospholipase/Carboxylesterase	0.112
PF09347	+	Domain of unknown function (DUF1989)	0.111
PF01447	-	Thermolysin metallopeptidase, catalytic domain	0.108
PF01678	+	Diaminopimelate epimerase	0.108
PF00805	-	Pentapeptide repeats (8 copies)	0.105
PF13278	-	Putative amidotransferase	0.099
PF01244	+	Membrane dipeptidase (Peptidase family M19)	0.098
PF13597	-	Anaerobic ribonucleoside-triphosphate reductase	0.097
PF08327	+	Activator of Hsp90 ATPase homolog 1-like protein	0.097
PF03733	+	Domain of unknown function (DUF307)	0.097
PF01464	+	Transglycosylase SLT domain	0.086
PF06165	+	Glycosyltransferase family 36	0.085
PF01909	+	Nucleotidyltransferase domain	0.083
PF03845	-	Spore germination protein	0.081
PF13342	-	C-terminal repeat of topoisomerase	0.080
PF02502	-	Ribose/Galactose Isomerase	0.076
PF13280	-	WYL domain	0.075
PF04332	+	Protein of unknown function (DUF475)	0.071
PF05228	+	CHASE4 domain	0.067
PF05119	-	Phage terminase, small subunit	0.066
PF02146	-	Sir2 family	0.062
PF14226	+	non-haem dioxygenase in morphine synthesis N-terminal	0.060
PF01391	-	Collagen triple helix repeat (20 copies)	0.058

PF00561	+	alpha/beta hydrolase fold	0.050
PF00082	-	Subtilase family	0.030
PF13408	-	Recombinase zinc beta ribbon domain	0.030
PF06832	+	Penicillin-Binding Protein C-terminus Family	0.024
PF04860	-	Phage portal protein	0.013
PF13669	-	Glyoxalase/Bleomycin resistance protein/Dioxygenase superfamily	0.010
PF01564	+	Spermine/spermidine synthase	0.006
PF03050	-	Transposase IS66 family	0.002
PF07508	-	Recombinase	0.001
PF07021	+	Methionine biosynthesis protein MetW	-0.000
PF02583	+	Metal-sensitive transcriptional repressor	-0.002
PF13420	-	Acetyltransferase (GNAT) domain	-0.008
PF08706	-	D5 N terminal like	-0.009
PF07703	+	Alpha-2-macroglobulin family N-terminal region	-0.011
PF00239	-	Resolvase, N terminal domain	-0.012
PF03096	-	Ndr family	-0.013
PF01493	-	GXGXG motif	-0.014
PF00670	+	S-adenosyl-L-homocysteine hydrolase, NAD binding domain	-0.015
PF13424	-	Tetratricopeptide repeat	-0.021
PF00465	-	Iron-containing alcohol dehydrogenase	-0.023
PF01451	-	Low molecular weight phosphotyrosine protein phosphatase	-0.024
PF02133	-	Permease for cytosine/purines, uracil, thiamine, allantoin	-0.026
PF13378	-	Enolase C-terminal domain-like	-0.030
PF00311	-	Phosphoenolpyruvate carboxylase	-0.034
PF13936	-	Helix-turn-helix domain	-0.038
PF03444	-	Winged helix-turn-helix transcription repressor, HrcA DNA-binding	-0.039
PF01261	-	Xylose isomerase-like TIM barrel	-0.039
PF00274	+	Fructose-bisphosphate aldolase class-I	-0.049
PF03649	-	Uncharacterised protein family (UPF0014)	-0.049
PF13438	-	Domain of unknown function (DUF4113)	-0.051
PF13683	-	Integrase core domain	-0.053
PF01226	-	Formate/nitrite transporter	-0.057
PF06742	+	Protein of unknown function (DUF1214)	-0.062
PF13518	-	Helix-turn-helix domain	-0.063
PF01978	-	Sugar-specific transcriptional regulator TrmB	-0.065
PF05899	+	Protein of unknown function (DUF861)	-0.066
PF04909	-	Amidohydrolase	-0.073
PF03320	+	Bacterial fructose-1,6-bisphosphatase, glpX-encoded	-0.077
PF00325	-	Bacterial regulatory proteins, crp family	-0.078
PF00350	-	Dynamin family	-0.079
PF01256	-	Carbohydrate kinase	-0.080
PF01977	-	3-octaprenyl-4-hydroxybenzoate carboxy-lyase	-0.081
PF00585	-	C-terminal regulatory domain of Threonine dehydratase	-0.091
PF07963	-	Prokaryotic N-terminal methylation motif	-0.091
PF03591	-	AzlC protein	-0.101
PF00665	-	Integrase core domain	-0.101
PF05437	-	Branched-chain amino acid transport protein (AzlD)	-0.109
PF13977	-	Bacterial transcriptional repressor	-0.109

PF10340	-	Protein of unknown function (DUF2424)	-0.120
PF06628	-	Catalase-related immune-responsive	-0.120
PF13309	-	HTH domain	-0.131
PF08348	-	YheO-like PAS domain	-0.131
PF13186	-	Iron-sulfur cluster-binding domain	-0.132
PF01610	-	Transposase	-0.132
PF13682	-	Chemoreceptor zinc-binding domain	-0.132
PF02446	-	4-alpha-glucanotransferase	-0.157
PF01764	-	Lipase (class 3)	-0.162
PF11066	-	Protein of unknown function (DUF2867)	-0.162
PF07670	-	Nucleoside recognition	-0.165
PF14690	-	zinc-finger of transposase IS204/IS1001/IS1096/IS1165	-0.166
PF03693	-	Uncharacterised protein family (UPF0156)	-0.172
PF05114	-	Protein of unknown function (DUF692)	-0.178
PF06821	+	Serine hydrolase	-0.180
PF13606	-	Ankyrin repeat	-0.184
PF11964	-	SpoIIAA-like	-0.184
PF07286	-	Protein of unknown function (DUF1445)	-0.189
PF06508	-	Queuosine biosynthesis protein QueC	-0.197
PF07729	-	FCD domain	-0.203
PF02615	-	Malate/L-lactate dehydrogenase	-0.217
PF00762	-	Ferrochelatase	-0.220
PF00301	-	Rubredoxin	-0.220
PF00984	-	UDP-glucose/GDP-mannose dehydrogenase family, central domain	-0.227
PF04290	-	Tripartite ATP-independent periplasmic transporters, DctQ component	-0.229
PF02152	-	Dihydroneopterin aldolase	-0.230
PF02277	-	Phosphoribosyltransferase	-0.230
PF14698	-	Argininosuccinate lyase C-terminal	-0.246
PF02649	-	Type I GTP cyclohydrolase folE2	-0.247
PF02771	-	Acyl-CoA dehydrogenase, N-terminal domain	-0.250
PF02652	-	L-lactate permease	-0.255
PF01957	-	NfeD-like C-terminal, partner-binding	-0.257
PF13899	-	Thioredoxin-like	-0.263
PF02511	-	Thymidylate synthase complementing protein	-0.285
PF01494	-	FAD binding domain	-0.294
PF02550	-	Acetyl-CoA hydrolase/transferase N-terminal domain	-0.298
PF00745	-	Glutamyl-tRNAGlu reductase, dimerisation domain	-0.298
PF13247	-	4Fe-4S dicluster domain	-0.311
PF02789	+	Cytosol aminopeptidase family, N-terminal domain	-0.328
PF03916	-	Polysulphide reductase, NrfD	-0.330
PF00809	-	Pterin binding enzyme	-0.333
PF04143	-	Sulphur transport	-0.337
PF03971	-	Monomeric isocitrate dehydrogenase	-0.355
PF01288	-	7,8-dihydro-6-hydroxymethylpterin-pyrophosphokinase (HPPK)	-0.373

Phenotype: Glycerol (Growth: Sugar), Predictor: phypat

Pfam	class	Pfam_desc	cor
PF02694	+	Uncharacterised BCR, YnfA/UPF0060 family	0.703

PF00667	+	FAD binding domain	0.703
PF01904	+	Protein of unknown function DUF72	0.632
PF08327	+	Activator of Hsp90 ATPase homolog 1-like protein	0.615
PF02649	+	Type I GTP cyclohydrolase folE2	0.594
PF13745	+	HxxPF-repeated domain	0.583
PF12708	+	Pectate lyase superfamily protein	0.574
PF04309	+	Glycerol-3-phosphate responsive antiterminator	0.561
PF07683	+	Cobalamin synthesis protein cobW C-terminal domain	0.556
PF09423	+	PhoD-like phosphatase	0.516
PF13188	+	PAS domain	0.483
PF04879	+	Molybdopterin oxidoreductase Fe4S4 domain	0.480
PF00670	+	S-adenosyl-L-homocysteine hydrolase, NAD binding domain	0.470
PF11604	+	Copper binding periplasmic protein CusF	0.445
PF09588	+	YqaJ-like viral recombinase domain	0.444
PF05724	+	Thiopurine S-methyltransferase (TPMT)	0.436
PF01526	+	Tn3 transposase DDE domain	0.431
PF00990	+	GGDEF domain	0.420
PF00420	+	NADH-ubiquinone/plastoquinone oxidoreductase chain 4L	0.416
PF13700	+	Domain of unknown function (DUF4158)	0.406
PF02909	+	Tetracyclin repressor, C-terminal all-alpha domain	0.399
PF13493	+	Domain of unknown function (DUF4118)	0.395
PF04233	+	Phage Mu protein F like protein	0.382
PF08877	+	MepB protein	0.367
PF05985	+	Ethanolamine ammonia-lyase light chain (EutC)	0.361
PF11168	+	Protein of unknown function (DUF2955)	0.361
PF12996	+	DUF based on E. rectale Gene description (DUF3880)	0.361
PF00232	+	Glycosyl hydrolase family 1	0.350
PF01418	+	Helix-turn-helix domain, rpiR family	0.346
PF01558	+	Pyruvate ferredoxin/flavodoxin oxidoreductase	0.341
PF15617	+	C-C_Bond_Lyase of the TIM-Barrel fold	0.338
PF02237	+	Biotin protein ligase C terminal domain	0.337
PF06568	+	Domain of unknown function (DUF1127)	0.309
PF14337	+	Domain of unknown function (DUF4393)	0.292
PF13172	+	PepSY-associated TM helix	0.276
PF06414	+	Zeta toxin	0.252
PF08495	+	FIST N domain	0.216
PF10442	+	FIST C domain	0.216
PF10551	+	MULE transposase domain	0.206
PF00520	+	Ion transport protein	0.171
PF04984	-	Phage tail sheath protein	0.150
PF01243	-	Pyridoxamine 5'-phosphate oxidase	0.129
PF04371	+	Porphyromonas-type peptidyl-arginine deiminase	0.101
PF00950	-	ABC 3 transport family	0.063
PF02627	-	Carboxymuconolactone decarboxylase family	0.058
PF13386	-	Cytochrome C biogenesis protein transmembrane region	0.044
PF13333	-	Integrase core domain	0.030
PF01070	-	FMN-dependent dehydrogenase	0.030
PF12796	-	Ankyrin repeats (3 copies)	0.023

PF13707	+	RloB-like protein	0.018
PF14622	-	Ribonuclease-III-like	0.010
PF13530	-	Sterol carrier protein domain	0.006
PF13186	-	Iron-sulfur cluster-binding domain	0.004
PF00636	-	Ribonuclease III domain	-0.015
PF00035	-	Double-stranded RNA binding motif	-0.015
PF02550	-	Acetyl-CoA hydrolase/transferase N-terminal domain	-0.023
PF05157	-	Type II secretion system (T2SS), protein E, N-terminal domain	-0.024
PF03633	-	Glycosyl hydrolase family 65, C-terminal domain	-0.035
PF13485	-	Peptidase MA superfamily	-0.038
PF04239	-	Protein of unknown function (DUF421)	-0.046
PF03328	-	HpcH/HpaI aldolase/citrate lyase family	-0.063
PF03458	-	UPF0126 domain	-0.063
PF01906	-	Putative heavy-metal-binding	-0.081
PF01210	-	NAD-dependent glycerol-3-phosphate dehydrogenase N-terminus	-0.093
PF04474	-	Protein of unknown function (DUF554)	-0.096
PF13482	-	RNase_H superfamily	-0.119
PF01612	-	3'-5' exonuclease	-0.122
PF05362	-	Lon protease (S16) C-terminal proteolytic domain	-0.133
PF02384	-	N-6 DNA Methylase	-0.148
PF00692	-	dUTPase	-0.164
PF13292	-	1-deoxy-D-xylulose-5-phosphate synthase	-0.166
PF02511	-	Thymidylate synthase complementing protein	-0.172
PF12706	-	Beta-lactamase superfamily domain	-0.181
PF00071	-	Ras family	-0.187
PF04892	-	VanZ like family	-0.195
PF00025	-	ADP-ribosylation factor family	-0.210
PF03883	-	Protein of unknown function (DUF328)	-0.218
PF05704	-	Capsular polysaccharide synthesis protein	-0.279
PF05649	-	Peptidase family M13	-0.280
PF03051	-	Peptidase C1-like family	-0.370
PF04011	-	LemA family	-0.409

Phenotype: Glycerol (Growth: Sugar), Predictor: phypat+PGL

Pfam	class	Pfam_desc	cor
PF02694	+	Uncharacterised BCR, YnfA/UPF0060 family	0.703
PF01904	+	Protein of unknown function DUF72	0.632
PF08327	+	Activator of Hsp90 ATPase homolog 1-like protein	0.615
PF12708	+	Pectate lyase superfamily protein	0.574
PF04309	+	Glycerol-3-phosphate responsive antiterminator	0.561
PF07683	+	Cobalamin synthesis protein cobW C-terminal domain	0.556
PF01019	+	Gamma-glutamyltranspeptidase	0.533
PF10588	+	NADH-ubiquinone oxidoreductase-G iron-sulfur binding region	0.450
PF09588	+	YqaJ-like viral recombinase domain	0.444
PF05724	+	Thiopurine S-methyltransferase (TPMT)	0.436
PF06779	+	Protein of unknown function (DUF1228)	0.433
PF01526	+	Tn3 transposase DDE domain	0.431
PF00990	+	GGDEF domain	0.420

PF14464	+	Prokaryotic homologs of the JAB domain	0.420
PF02203	+	Tar ligand binding domain homologue	0.415
PF00245	+	Alkaline phosphatase	0.412
PF13700	+	Domain of unknown function (DUF4158)	0.406
PF13493	+	Domain of unknown function (DUF4118)	0.395
PF14411	+	A nuclease of the HNH/ENDO VII superfamily with conserved LHH	0.368
PF06751	+	Ethanolamine ammonia lyase large subunit (EutB)	0.361
PF12996	+	DUF based on E. rectale Gene description (DUF3880)	0.361
PF11611	+	Domain of unknown function (DUF4352)	0.354
PF00232	+	Glycosyl hydrolase family 1	0.350
PF01418	+	Helix-turn-helix domain, rpiR family	0.346
PF06081	+	Bacterial protein of unknown function (DUF939)	0.345
PF01008	+	Initiation factor 2 subunit family	0.341
PF01558	+	Pyruvate ferredoxin/flavodoxin oxidoreductase	0.341
PF13620	+	Carboxypeptidase regulatory-like domain	0.340
PF02733	+	Dak1 domain	0.322
PF05154	+	TM2 domain	0.321
PF12957	+	Domain of unknown function (DUF3846)	0.317
PF11528	+	Protein of unknown function (DUF3224)	0.317
PF02274	+	Amidinotransferase	0.303
PF04439	+	Streptomycin adenylyltransferase	0.302
PF01361	+	Tautomerase enzyme	0.300
PF03104	+	DNA polymerase family B, exonuclease domain	0.283
PF05899	+	Protein of unknown function (DUF861)	0.276
PF06888	+	Putative Phosphatase	0.261
PF06414	+	Zeta toxin	0.252
PF01425	+	Amidase	0.249
PF01238	+	Phosphomannose isomerase type I	0.245
PF01844	-	HNH endonuclease	0.243
PF04962	+	KduI/IolB family	0.241
PF04740	+	LXG domain of WXG superfamily	0.239
PF14815	+	NUDIX domain	0.228
PF10442	+	FIST C domain	0.216
PF04964	+	Flp/Fap pilin component	0.216
PF08495	+	FIST N domain	0.216
PF13231	+	Dolichyl-phosphate-mannose-protein mannosyltransferase	0.211
PF04138	+	GtrA-like protein	0.208
PF10400	+	Virulence activator alpha C-term	0.183
PF00520	+	Ion transport protein	0.171
PF04332	+	Protein of unknown function (DUF475)	0.166
PF02744	+	Galactose-1-phosphate uridyl transferase, C-terminal domain	0.163
PF12840	-	Helix-turn-helix domain	0.156
PF13539	+	D-alanyl-D-alanine carboxypeptidase	0.140
PF00302	-	Chloramphenicol acetyltransferase	0.121
PF10145	-	Phage-related minor tail protein	0.112
PF13653	-	Glycerophosphoryl diester phosphodiesterase family	0.107
PF00150	+	Cellulase (glycosyl hydrolase family 5)	0.101
PF06993	-	Protein of unknown function (DUF1304)	0.098

PF13280	-	WYL domain	0.097
PF03553	-	Na+/H+ antiporter family	0.096
PF05673	+	Protein of unknown function (DUF815)	0.094
PF13384	-	Homeodomain-like domain	0.087
PF01885	-	RNA 2'-phosphotransferase, Tpt1 / KptA family	0.083
PF06961	-	Protein of unknown function (DUF1294)	0.083
PF13936	+	Helix-turn-helix domain	0.083
PF01258	-	Prokaryotic dksA/traR C4-type zinc finger	0.078
PF00962	-	Adenosine/AMP deaminase	0.078
PF11066	-	Protein of unknown function (DUF2867)	0.063
PF13744	-	Helix-turn-helix domain	0.059
PF02368	-	Bacterial Ig-like domain (group 2)	0.054
PF03576	-	Peptidase family S58	0.053
PF13899	-	Thioredoxin-like	0.050
PF00657	-	GDSL-like Lipase/Acylhydrolase	0.046
PF02397	-	Bacterial sugar transferase	0.044
PF07963	-	Prokaryotic N-terminal methylation motif	0.038
PF13544	-	Type IV pilin N-term methylation site GFxxxE	0.028
PF10410	-	DnaB-helicase binding domain of primase	0.023
PF13707	+	RloB-like protein	0.018
PF13186	-	Iron-sulfur cluster-binding domain	0.004
PF06250	+	Protein of unknown function (DUF1016)	-0.013
PF04973	-	Nicotinamide mononucleotide transporter	-0.031
PF05935	-	Arylsulfotransferase (ASST)	-0.043
PF04239	-	Protein of unknown function (DUF421)	-0.046
PF00877	-	NlpC/P60 family	-0.050
PF02677	+	Uncharacterized BCR, COG1636	-0.056
PF03328	-	HpcH/HpaI aldolase/citrate lyase family	-0.063
PF01906	-	Putative heavy-metal-binding	-0.081
PF01055	-	Glycosyl hydrolases family 31	-0.089
PF05195	-	Aminopeptidase P, N-terminal domain	-0.106
PF13482	-	RNase_H superfamily	-0.119
PF01867	-	CRISPR associated protein Cas1	-0.137
PF03993	-	Domain of Unknown Function (DUF349)	-0.138
PF14529	-	Endonuclease-reverse transcriptase	-0.147
PF02384	-	N-6 DNA Methylase	-0.148
PF12900	-	Pyridoxamine 5'-phosphate oxidase	-0.154
PF00071	-	Ras family	-0.187
PF06824	-	Protein of unknown function (DUF1237)	-0.192
PF02618	-	YceG-like family	-0.195
PF14010	-	Phosphoenolpyruvate carboxylase	-0.258
PF00654	-	Voltage gated chloride channel	-0.283

Phenotype: Lactose (Growth: Sugar), Predictor: phypat

Pfam	class	Pfam_desc	cor
PF00703	+	Glycosyl hydrolases family 2	0.573
PF02836	+	Glycosyl hydrolases family 2, TIM barrel domain	0.563
PF06054	+	Competence protein CoiA-like family	0.532

PF08244	+	Glycosyl hydrolases family 32 C terminal	0.517
PF13906	+	C-terminus of AA_permease	0.488
PF04616	+	Glycosyl hydrolases family 43	0.477
PF12464	+	Maltose acetyltransferase	0.455
PF02929	+	Beta galactosidase small chain	0.454
PF06279	+	Protein of unknown function (DUF1033)	0.438
PF02903	+	Alpha amylase, N-terminal ig-like domain	0.422
PF03306	+	Alpha-acetolactate decarboxylase	0.411
PF08532	+	Beta-galactosidase trimerisation domain	0.392
PF00401	+	ATP synthase, Delta/Epsilon chain, long alpha-helix domain	0.387
PF02901	+	Pyruvate formate lyase	0.362
PF03773	+	Predicted permease	0.359
PF06202	+	Amylo-alpha-1,6-glucosidase	0.355
PF06018	+	CodY GAF-like domain	0.355
PF05870	+	Phenolic acid decarboxylase (PAD)	0.343
PF04464	+	CDP-Glycerol:Poly(glycerophosphate) glycerophosphotransferase	0.331
PF07929	+	Plasmid pRiA4b ORF-3-like protein	0.327
PF06993	+	Protein of unknown function (DUF1304)	0.323
PF13248	+	zinc-ribbon domain	0.313
PF04205	+	FMN-binding domain	0.310
PF02475	+	Met-10+ like-protein	0.283
PF13088	+	BNR repeat-like domain	0.272
PF03050	+	Transposase IS66 family	0.267
PF01928	+	CYTH domain	0.266
PF12098	+	Protein of unknown function (DUF3574)	0.258
PF02922	+	Carbohydrate-binding module 48 (Isoamylase N-terminal domain)	0.248
PF13240	+	zinc-ribbon domain	0.248
PF07693	+	KAP family P-loop domain	0.242
PF12969	+	Domain of Unknown Function with PDB structure (DUF3857)	0.242
PF07751	+	Abi-like protein	0.242
PF12708	+	Pectate lyase superfamily protein	0.240
PF06902	+	Divergent 4Fe-4S mono-cluster	0.238
PF07221	+	N-acylglucosamine 2-epimerase (GlcNAc 2-epimerase)	0.234
PF00908	+	dTDP-4-dehydrorhamnose 3,5-epimerase	0.231
PF04892	+	VanZ like family	0.227
PF01883	+	Domain of unknown function DUF59	0.222
PF14253	+	Bacteriophage abortive infection AbiH	0.216
PF12895	+	Anaphase-promoting complex, cyclosome, subunit 3	0.209
PF01661	+	Macro domain	0.203
PF02661	+	Fic/DOC family	0.188
PF00854	+	POT family	0.188
PF07799	+	Protein of unknown function (DUF1643)	0.174
PF13227	+	Protein of unknown function (DUF4035)	0.172
PF11745	+	Protein of unknown function (DUF3304)	0.172
PF09863	+	Uncharacterized protein conserved in bacteria (DUF2090)	0.163
PF06833	+	Malonate decarboxylase gamma subunit (MdcE)	0.155
PF09903	-	Uncharacterized protein conserved in bacteria (DUF2130)	0.136
PF06445	+	GyrI-like small molecule binding domain	0.126

PF05035	+	2-keto-3-deoxy-galactonokinase	0.122
PF13506	+	Glycosyl transferase family 21	0.115
PF01814	+	Hemerythrin HHE cation binding domain	0.111
PF08388	-	Group II intron, maturase-specific domain	0.106
PF13744	+	Helix-turn-helix domain	0.103
PF10106	+	Uncharacterized protein conserved in bacteria (DUF2345)	0.084
PF13740	-	ACT domain	0.080
PF05119	-	Phage terminase, small subunit	0.078
PF03432	-	Relaxase/Mobilisation nuclease domain	0.073
PF02583	+	Metal-sensitive transcriptional repressor	0.055
PF00239	-	Resolvase, N terminal domain	0.052
PF05708	-	Orthopoxvirus protein of unknown function (DUF830)	0.049
PF13503	+	Domain of unknown function (DUF4123)	0.047
PF08386	+	TAP-like protein	0.042
PF13599	+	Pentapeptide repeats (9 copies)	0.031
PF02649	+	Type I GTP cyclohydrolase folE2	0.023
PF04313	-	Type I restriction enzyme R protein N terminus (HSDR_N)	0.019
PF11974	+	Alpha-2-macroglobulin MG1 domain	0.010
PF05488	+	PAAR motif	0.010
PF13629	+	Pilus formation protein N terminal region	0.005
PF06114	-	Domain of unknown function (DUF955)	-0.000
PF01163	-	RIO1 family	-0.012
PF07703	+	Alpha-2-macroglobulin family N-terminal region	-0.013
PF12852	+	Cupin	-0.034
PF03239	+	Iron permease FTR1 family	-0.036
PF01844	-	HNH endonuclease	-0.041
PF13305	-	WHG domain	-0.052
PF04286	+	Protein of unknown function (DUF445)	-0.055
PF14525	+	AraC-binding-like domain	-0.058
PF01396	-	Topoisomerase DNA binding C4 zinc finger	-0.058
PF13539	+	D-alanyl-D-alanine carboxypeptidase	-0.061
PF03865	+	Haemolysin secretion/activation protein ShlB/FhaC/HecB	-0.071
PF12832	+	MFS_1 like family	-0.074
PF07885	-	Ion channel	-0.083
PF13289	-	SIR2-like domain	-0.087
PF03102	-	NeuB family	-0.089
PF02126	-	Phosphotriesterase family	-0.092
PF13555	-	P-loop containing region of AAA domain	-0.094
PF05977	-	Transmembrane secretion effector	-0.101
PF04715	+	Anthranilate synthase component I, N terminal region	-0.102
PF13186	-	Iron-sulfur cluster-binding domain	-0.106
PF01507	+	Phosphoadenosine phosphosulfate reductase family	-0.117
PF02447	-	GntP family permease	-0.130
PF14503	-	YhfZ C-terminal domain	-0.131
PF02464	-	Competence-damaged protein	-0.139
PF13378	-	Enolase C-terminal domain-like	-0.140
PF12759	-	InsA C-terminal domain	-0.143
PF13089	-	Polyphosphate kinase N-terminal domain	-0.156

PF07683	+	Cobalamin synthesis protein cobW C-terminal domain	-0.159
PF04471	-	Restriction endonuclease	-0.165
PF13519	-	von Willebrand factor type A domain	-0.167
PF12900	-	Pyridoxamine 5'-phosphate oxidase	-0.170
PF00724	-	NADH:flavin oxidoreductase / NADH oxidase family	-0.172
PF08003	-	Protein of unknown function (DUF1698)	-0.174
PF13090	-	Polyphosphate kinase C-terminal domain	-0.178
PF10294	-	Putative methyltransferase	-0.192
PF02803	-	Thiolase, C-terminal domain	-0.194
PF12399	-	Branched-chain amino acid ATP-binding cassette transporter	-0.201
PF03308	-	ArgK protein	-0.205
PF01243	-	Pyridoxamine 5'-phosphate oxidase	-0.213
PF03994	-	Domain of Unknown Function (DUF350)	-0.222
PF00984	-	UDP-glucose/GDP-mannose dehydrogenase family, central domain	-0.233
PF13362	-	Toprim domain	-0.241
PF01769	-	Divalent cation transporter	-0.243
PF00939	-	Sodium:sulfate symporter transmembrane region	-0.257
PF01728	-	FtsJ-like methyltransferase	-0.258
PF08298	-	PrkA AAA domain	-0.259
PF01614	-	Bacterial transcriptional regulator	-0.262
PF13490	-	Putative zinc-finger	-0.266
PF03572	-	Peptidase family S41	-0.280
PF05076	-	Suppressor of fused protein (SUFU)	-0.291
PF00545	-	ribonuclease	-0.303
PF03892	-	Nitrate reductase cytochrome c-type subunit (NapB)	-0.321
PF03927	-	NapD protein	-0.329
PF01478	-	Type IV leader peptidase family	-0.361
PF02515	-	CoA-transferase family III	-0.378
PF00463	-	Isocitrate lyase family	-0.378
PF03320	-	Bacterial fructose-1,6-bisphosphatase, glpX-encoded	-0.386
PF02190	-	ATP-dependent protease La (LON) domain	-0.391
PF01863	-	Protein of unknown function DUF45	-0.398
PF01957	-	NfeD-like C-terminal, partner-binding	-0.414
PF13766	-	2-enoyl-CoA Hydratase C-terminal region	-0.423
PF01012	-	Electron transfer flavoprotein domain	-0.430
PF00355	-	Rieske [2Fe-2S] domain	-0.476
PF02913	-	FAD linked oxidases, C-terminal domain	-0.476
PF00883	-	Cytosol aminopeptidase family, catalytic domain	-0.558

Phenotype: Lactose (Growth: Sugar), Predictor: phypat+PGL

Pfam	class	Pfam_desc	cor
PF00703	+	Glycosyl hydrolases family 2	0.573
PF03051	+	Peptidase C1-like family	0.549
PF08244	+	Glycosyl hydrolases family 32 C terminal	0.517
PF13906	+	C-terminus of AA_permease	0.488
PF06824	+	Protein of unknown function (DUF1237)	0.485
PF04616	+	Glycosyl hydrolases family 43	0.477
PF02929	+	Beta galactosidase small chain	0.454

PF01154	+	Hydroxymethylglutaryl-coenzyme A synthase N terminal	0.435
PF13636	-	pre-rRNA processing and ribosome biogenesis	0.432
PF04854	+	Protein of unknown function, DUF624	0.427
PF02903	+	Alpha amylase, N-terminal ig-like domain	0.422
PF08532	+	Beta-galactosidase trimerisation domain	0.392
PF00401	+	ATP synthase, Delta/Epsilon chain, long alpha-helix domain	0.387
PF14498	+	Glycosyl hydrolase family 65, N-terminal domain	0.386
PF02901	+	Pyruvate formate lyase	0.362
PF11694	+	Protein of unknown function (DUF3290)	0.358
PF08455	+	Bacterial SNF2 helicase associated	0.344
PF01263	+	Aldose 1-epimerase	0.337
PF07929	+	Plasmid pRiA4b ORF-3-like protein	0.327
PF07944	+	Putative glycosyl hydrolase of unknown function (DUF1680)	0.326
PF02486	+	Replication initiation factor	0.323
PF07532	+	Bacterial Ig-like domain (group 4)	0.319
PF13248	+	zinc-ribbon domain	0.313
PF01487	+	Type I 3-dehydroquinase	0.311
PF04205	+	FMN-binding domain	0.310
PF00413	+	Matrixin	0.306
PF03590	+	Aspartate-ammonia ligase	0.298
PF13173	+	AAA domain	0.296
PF06962	+	Putative rRNA methylase	0.286
PF02475	+	Met-10+ like-protein	0.283
PF00722	+	Glycosyl hydrolases family 16	0.273
PF00532	+	Periplasmic binding proteins and sugar binding domain of LacI family	0.272
PF14393	+	Domain of unknown function (DUF4422)	0.272
PF13154	+	Protein of unknown function (DUF3991)	0.272
PF13088	+	BNR repeat-like domain	0.272
PF03050	+	Transposase IS66 family	0.267
PF01928	+	CYTH domain	0.266
PF12733	+	Cadherin-like beta sandwich domain	0.262
PF01915	+	Glycosyl hydrolase family 3 C-terminal domain	0.257
PF13377	+	Periplasmic binding protein-like domain	0.257
PF02595	+	Glycerate kinase family	0.257
PF01289	+	Thiol-activated cytolysin	0.254
PF06874	+	Firmicute fructose-1,6-bisphosphatase	0.250
PF13240	+	zinc-ribbon domain	0.248
PF07751	+	Abi-like protein	0.242
PF04883	+	Bacteriophage HK97-gp10, putative tail-component	0.240
PF03083	+	Sugar efflux transporter for intercellular exchange	0.238
PF06902	+	Divergent 4Fe-4S mono-cluster	0.238
PF04991	+	LicD family	0.237
PF14659	+	Phage integrase, N-terminal SAM-like domain	0.237
PF03629	+	Domain of unknown function (DUF303)	0.235
PF07221	+	N-acylglucosamine 2-epimerase (GlcNAc 2-epimerase)	0.234
PF00069	+	Protein kinase domain	0.233
PF00908	+	dTDP-4-dehydrorhamnose 3,5-epimerase	0.231
PF01476	+	LysM domain	0.228

PF04892	+	VanZ like family	0.227
PF11611	+	Domain of unknown function (DUF4352)	0.222
PF13635	+	Domain of unknown function (DUF4143)	0.220
PF02742	+	Iron dependent repressor, metal binding and dimerisation domain	0.218
PF03390	+	2-hydroxycarboxylate transporter family	0.217
PF14253	+	Bacteriophage abortive infection AbiH	0.216
PF09355	+	Phage protein Gp19/Gp15/Gp42	0.213
PF03275	+	UDP-galactopyranose mutase	0.210
PF12895	+	Anaphase-promoting complex, cyclosome, subunit 3	0.209
PF00186	+	Dihydrofolate reductase	0.209
PF01661	+	Macro domain	0.203
PF12773	+	Double zinc ribbon	0.199
PF13567	+	Domain of unknown function (DUF4131)	0.191
PF02661	+	Fic/DOC family	0.188
PF12669	+	Virus attachment protein p12 family	0.180
PF00135	+	Carboxylesterase family	0.179
PF12966	+	N-ATPase, AtpR subunit	0.178
PF01116	+	Fructose-bisphosphate aldolase class-II	0.177
PF07799	+	Protein of unknown function (DUF1643)	0.174
PF08240	+	Alcohol dehydrogenase GroES-like domain	0.174
PF00231	+	ATP synthase	0.173
PF00119	+	ATP synthase A chain	0.173
PF11745	+	Protein of unknown function (DUF3304)	0.172
PF02614	+	Glucuronate isomerase	0.168
PF00145	+	C-5 cytosine-specific DNA methylase	0.166
PF10933	+	Protein of unknown function (DUF2827)	0.158
PF07350	+	Protein of unknown function (DUF1479)	0.156
PF02884	-	Polysaccharide lyase family 8, C-terminal beta-sandwich domain	0.153
PF02397	-	Bacterial sugar transferase	0.150
PF07730	+	Histidine kinase	0.150
PF07358	+	Protein of unknown function (DUF1482)	0.149
PF08707	+	Primase C terminal 2 (PriCT-2)	0.148
PF11876	+	Protein of unknown function (DUF3396)	0.148
PF06245	+	Protein of unknown function (DUF1015)	0.147
PF15534	+	Putative toxin 56	0.145
PF07377	+	Protein of unknown function (DUF1493)	0.131
PF07859	+	alpha/beta hydrolase fold	0.131
PF06445	+	GyrI-like small molecule binding domain	0.126
PF08323	-	Starch synthase catalytic domain	0.124
PF02278	-	Polysaccharide lyase family 8, super-sandwich domain	0.122
PF02677	+	Uncharacterized BCR, COG1636	0.120
PF13506	+	Glycosyl transferase family 21	0.115
PF10145	-	Phage-related minor tail protein	0.114
PF11700	+	Vacuole effluxer Atg22 like	0.114
PF13350	+	Tyrosine phosphatase family	0.113
PF00194	-	Eukaryotic-type carbonic anhydrase	0.111
PF13408	-	Recombinase zinc beta ribbon domain	0.108
PF09524	+	Conserved phage C-terminus (Phg_2220_C)	0.107

PF00872	+	Transposase, Mutator family	0.106
PF00205	+	Thiamine pyrophosphate enzyme, central domain	0.102
PF13310	+	Virulence protein RhuM family	0.100
PF05971	-	Protein of unknown function (DUF890)	0.098
PF04172	+	LrgB-like family	0.094
PF11213	+	Protein of unknown function (DUF3006)	0.092
PF01638	-	HxlR-like helix-turn-helix	0.091
PF04940	+	Sensors of blue-light using FAD	0.088
PF11258	+	Protein of unknown function (DUF3048)	0.084
PF02894	-	Oxidoreductase family, C-terminal alpha/beta domain	0.074
PF05857	-	TraX protein	0.065
PF03812	+	2-keto-3-deoxygluconate permease	0.062
PF14635	+	Helix-hairpin-helix motif	0.050
PF06296	+	Protein of unknown function (DUF1044)	0.047
PF13480	-	Acetyltransferase (GNAT) domain	0.046
PF02146	-	Sir2 family	0.046
PF01027	+	Inhibitor of apoptosis-promoting Bax1	0.045
PF00201	+	UDP-glucoronosyl and UDP-glucosyl transferase	0.042
PF09365	+	Conserved hypothetical protein (DUF2461)	0.040
PF08681	-	Protein of unknown function (DUF1778)	0.040
PF01156	-	Inosine-uridine preferring nucleoside hydrolase	0.040
PF04657	-	Protein of unknown function, DUF606	0.038
PF06342	+	Alpha/beta hydrolase of unknown function (DUF1057)	0.037
PF01894	+	Uncharacterised protein family UPF0047	0.032
PF14526	-	Integron-associated effector binding protein	0.028
PF01841	-	Transglutaminase-like superfamily	0.007
PF10711	+	Hypothetical protein (DUF2513)	0.004
PF14785	-	Maltose transport system permease protein MalF P2 domain	-0.005
PF13531	-	Bacterial extracellular solute-binding protein	-0.009
PF05016	-	Plasmid stabilisation system protein	-0.033
PF01555	-	DNA methylase	-0.048
PF13653	-	Glycerophosphoryl diester phosphodiesterase family	-0.049
PF04304	+	Protein of unknown function (DUF454)	-0.049
PF13305	-	WHG domain	-0.052
PF05729	-	NACHT domain	-0.054
PF11985	+	Protein of unknown function (DUF3486)	-0.054
PF01645	-	Conserved region in glutamate synthase	-0.054
PF03703	-	Bacterial PH domain	-0.054
PF04286	+	Protein of unknown function (DUF445)	-0.055
PF01161	-	Phosphatidylethanolamine-binding protein	-0.055
PF14525	+	AraC-binding-like domain	-0.058
PF08239	-	Bacterial SH3 domain	-0.064
PF07819	-	PGAP1-like protein	-0.064
PF07383	-	Protein of unknown function (DUF1496)	-0.067
PF06966	-	Protein of unknown function (DUF1295)	-0.067
PF03865	+	Haemolysin secretion/activation protein ShlB/FhaC/HecB	-0.071
PF13586	-	Transposase DDE domain	-0.082
PF07885	-	Ion channel	-0.083

PF06564	-	YhjQ protein	-0.088
PF08392	-	FAE1/Type III polyketide synthase-like protein	-0.090
PF13458	-	Periplasmic binding protein	-0.092
PF04170	-	NlpE N-terminal domain	-0.092
PF01891	+	Cobalt uptake substrate-specific transmembrane region	-0.095
PF05901	-	Excalibur calcium-binding domain	-0.100
PF09600	-	Cyd operon protein YbgE (Cyd_oper_YbgE)	-0.101
PF00325	-	Bacterial regulatory proteins, crp family	-0.101
PF01977	+	3-octaprenyl-4-hydroxybenzoate carboxy-lyase	-0.102
PF03479	-	Domain of unknown function (DUF296)	-0.104
PF12738	-	twin BRCT domain	-0.106
PF02915	-	Rubrerythrin	-0.115
PF09297	-	NADH pyrophosphatase zinc ribbon domain	-0.118
PF01155	-	Hydrogenase expression/synthesis hypA family	-0.121
PF06196	-	Protein of unknown function (DUF997)	-0.121
PF02447	-	GntP family permease	-0.130
PF01750	-	Hydrogenase maturation protease	-0.131
PF00092	-	von Willebrand factor type A domain	-0.138
PF13378	-	Enolase C-terminal domain-like	-0.140
PF05724	+	Thiopurine S-methyltransferase (TPMT)	-0.141
PF11066	-	Protein of unknown function (DUF2867)	-0.146
PF07157	-	DNA circularisation protein N-terminus	-0.155
PF00491	+	Arginase family	-0.155
PF13667	-	ThiC-associated domain	-0.156
PF09424	-	Yqey-like protein	-0.164
PF04471	-	Restriction endonuclease	-0.165
PF13519	-	von Willebrand factor type A domain	-0.167
PF04655	-	Aminoglycoside/hydroxyurea antibiotic resistance kinase	-0.174
PF13090	-	Polyphosphate kinase C-terminal domain	-0.178
PF09829	-	Uncharacterized protein conserved in bacteria (DUF2057)	-0.184
PF01734	-	Patatin-like phospholipase	-0.187
PF03738	-	Glutathionylspermidine synthase preATP-grasp	-0.187
PF10294	-	Putative methyltransferase	-0.192
PF01243	-	Pyridoxamine 5'-phosphate oxidase	-0.213
PF03853	-	YjeF-related protein N-terminus	-0.215
PF02607	+	B12 binding domain	-0.218
PF07731	-	Multicopper oxidase	-0.220
PF03720	-	UDP-glucose/GDP-mannose dehydrogenase family, UDP binding domain	-0.221
PF03994	-	Domain of Unknown Function (DUF350)	-0.222
PF10615	-	Protein of unknown function (DUF2470)	-0.224
PF00984	-	UDP-glucose/GDP-mannose dehydrogenase family, central domain	-0.233
PF13231	-	Dolichyl-phosphate-mannose-protein mannosyltransferase	-0.234
PF01769	-	Divalent cation transporter	-0.243
PF13606	-	Ankyrin repeat	-0.244
PF00023	-	Ankyrin repeat	-0.250
PF04107	-	Glutamate-cysteine ligase family 2(GCS2)	-0.257
PF01614	-	Bacterial transcriptional regulator	-0.262
PF03916	-	Polysulphide reductase, NrfD	-0.275

PF02667	-	Short chain fatty acid transporter	-0.291
PF02550	-	Acetyl-CoA hydrolase/transferase N-terminal domain	-0.293
PF13241	-	Putative NAD(P)-binding	-0.294
PF07411	-	Domain of unknown function (DUF1508)	-0.300
PF13857	-	Ankyrin repeats (many copies)	-0.301
PF13452	-	N-terminal half of MaoC dehydratase	-0.302
PF00925	-	GTP cyclohydrolase II	-0.323
PF01258	-	Prokaryotic dksA/traR C4-type zinc finger	-0.337
PF02545	-	Maf-like protein	-0.355
PF01478	-	Type IV leader peptidase family	-0.361
PF02541	-	Ppx/GppA phosphatase family	-0.370
PF01957	-	NfeD-like C-terminal, partner-binding	-0.414
PF02913	-	FAD linked oxidases, C-terminal domain	-0.476

Phenotype: L-Arabinose (Growth: Sugar), Predictor: phypat

Pfam	class	Pfam_desc	cor
PF11762	+	L-arabinose isomerase C-terminal domain	0.841
PF02610	+	L-arabinose isomerase	0.841
PF04295	+	D-galactarate dehydratase / Altronate hydrolase, C terminus	0.585
PF01011	+	PQQ enzyme repeat	0.582
PF11575	+	FhuF 2Fe-2S C-terminal domain	0.562
PF11941	+	Domain of unknown function (DUF3459)	0.534
PF07745	+	Glycosyl hydrolase family 53	0.504
PF06964	+	Alpha-L-arabinofuranosidase C-terminus	0.465
PF01614	+	Bacterial transcriptional regulator	0.416
PF04328	+	Protein of unknown function (DUF466)	0.390
PF06276	+	Ferric iron reductase FhuF-like transporter	0.379
PF01161	+	Phosphatidylethanolamine-binding protein	0.375
PF12835	+	Integrase	0.340
PF01638	+	HxlR-like helix-turn-helix	0.325
PF07005	+	Protein of unknown function, DUF1537	0.323
PF04024	+	PspC domain	0.319
PF03551	+	Transcriptional regulator PadR-like family	0.310
PF07015	+	VirC1 protein	0.303
PF00135	+	Carboxylesterase family	0.297
PF13229	+	Right handed beta helix region	0.296
PF03812	-	2-keto-3-deoxygluconate permease	0.281
PF04266	+	ASCH domain	0.270
PF13565	+	Homeodomain-like domain	0.268
PF13005	+	zinc-finger binding domain of transposase IS66	0.266
PF07075	+	Protein of unknown function (DUF1343)	0.248
PF14354	+	Restriction alleviation protein Lar	0.218
PF13599	+	Pentapeptide repeats (9 copies)	0.198
PF14529	-	Endonuclease-reverse transcriptase	0.188
PF13620	+	Carboxypeptidase regulatory-like domain	0.184
PF03632	-	Glycosyl hydrolase family 65 central catalytic domain	0.183
PF06965	-	Na+/H+ antiporter 1	0.173
PF01989	+	Protein of unknown function DUF126	0.172

PF03636	-	Glycosyl hydrolase family 65, N-terminal domain	0.161
PF06953	+	Arsenical resistance operon trans-acting repressor ArsD	0.155
PF03306	+	Alpha-acetolactate decarboxylase	0.145
PF01612	-	3'-5' exonuclease	0.145
PF10099	+	Anti-sigma-K factor rskA	0.136
PF13470	+	PIN domain	0.131
PF03186	+	CobD/Cbib protein	0.130
PF08818	+	Domain of unknown function (DU1801)	0.125
PF03354	+	Phage Terminase	0.124
PF01435	-	Peptidase family M48	0.112
PF01476	-	LysM domain	0.109
PF09424	+	Yqey-like protein	0.105
PF14256	+	YwiC-like protein	0.103
PF02823	-	ATP synthase, Delta/Epsilon chain, beta-sandwich domain	0.092
PF05050	+	Methyltransferase FkbM domain	0.091
PF02604	-	Antitoxin Phd_YefM, type II toxin-antitoxin system	0.077
PF13514	+	AAA domain	0.072
PF01243	-	Pyridoxamine 5'-phosphate oxidase	0.066
PF01676	-	Metalloenzyme superfamily	0.065
PF01844	-	HNH endonuclease	0.061
PF02424	-	ApbE family	0.056
PF10544	+	T5orf172 domain	0.043
PF06250	-	Protein of unknown function (DUF1016)	0.038
PF00195	-	Chalcone and stilbene synthases, N-terminal domain	0.022
PF00268	-	Ribonucleotide reductase, small chain	0.016
PF04471	-	Restriction endonuclease	0.011
PF03193	-	Protein of unknown function, DUF258	0.004
PF00485	-	Phosphoribulokinase / Uridine kinase family	0.003
PF04548	-	AIG1 family	-0.003
PF03575	-	Peptidase family S51	-0.009
PF13175	-	AAA ATPase domain	-0.018
PF00578	-	AhpC/TSA family	-0.024
PF03065	-	Glycosyl hydrolase family 57	-0.057
PF14082	-	Domain of unknown function (DUF4263)	-0.072
PF13087	-	AAA domain	-0.090
PF01555	-	DNA methylase	-0.097
PF13564	-	DoxX-like family	-0.102
PF05193	-	Peptidase M16 inactive domain	-0.104
PF00830	-	Ribosomal L28 family	-0.114
PF01633	-	Choline/ethanolamine kinase	-0.121
PF04326	-	Divergent AAA domain	-0.125
PF02272	-	DHHA1 domain	-0.128
PF02677	-	Uncharacterized BCR, COG1636	-0.141
PF10067	-	Predicted membrane protein (DUF2306)	-0.144
PF00365	-	Phosphofructokinase	-0.145
PF12893	-	Putative lumazine-binding	-0.153
PF13289	-	SIR2-like domain	-0.157
PF13495	-	Phage integrase, N-terminal SAM-like domain	-0.183

PF07510	-	Protein of unknown function (DUF1524)	-0.198
PF07536	-	HWE histidine kinase	-0.210
PF02502	-	Ribose/Galactose Isomerase	-0.213
PF06280	-	Fn3-like domain (DUF1034)	-0.258
PF02361	-	Cobalt transport protein	-0.327

Phenotype: L-Arabinose (Growth: Sugar), Predictor: phypat+PGL

Pfam	class	Pfam_desc	cor
PF11762	+	L-arabinose isomerase C-terminal domain	0.841
PF04295	+	D-galactarate dehydratase / Altronate hydrolase, C terminus	0.585
PF01011	+	PQQ enzyme repeat	0.582
PF13802	+	Galactose mutarotase-like	0.546
PF11941	+	Domain of unknown function (DUF3459)	0.534
PF14310	+	Fibronectin type III-like domain	0.514
PF08362	+	YcdC-like protein, C-terminal region	0.506
PF00930	+	Dipeptidyl peptidase IV (DPP IV) N-terminal region	0.470
PF06964	+	Alpha-L-arabinofuranosidase C-terminus	0.465
PF01963	+	TraB family	0.433
PF02929	-	Beta galactosidase small chain	0.417
PF01614	+	Bacterial transcriptional regulator	0.416
PF00982	+	Glycosyltransferase family 20	0.398
PF06276	+	Ferric iron reductase FhuF-like transporter	0.379
PF10694	-	Protein of unknown function (DUF2500)	0.372
PF01931	-	Protein of unknown function DUF84	0.356
PF04230	+	Polysaccharide pyruvyl transferase	0.350
PF02381	+	MraZ protein	0.342
PF12835	+	Integrase	0.340
PF01566	+	Natural resistance-associated macrophage protein	0.325
PF07005	+	Protein of unknown function, DUF1537	0.323
PF02586	+	Uncharacterised ACR, COG2135	0.318
PF03551	+	Transcriptional regulator PadR-like family	0.310
PF06808	+	DctM-like transporters	0.306
PF07015	+	VirC1 protein	0.303
PF00135	+	Carboxylesterase family	0.297
PF02595	+	Glycerate kinase family	0.281
PF13565	+	Homeodomain-like domain	0.268
PF13005	+	zinc-finger binding domain of transposase IS66	0.266
PF00848	+	Ring hydroxylating alpha subunit (catalytic domain)	0.266
PF10145	+	Phage-related minor tail protein	0.259
PF01627	-	Hpt domain	0.258
PF05534	+	HicB family	0.249
PF01472	-	PUA domain	0.247
PF05985	+	Ethanolamine ammonia-lyase light chain (EutC)	0.247
PF14497	-	Glutathione S-transferase, C-terminal domain	0.244
PF00703	-	Glycosyl hydrolases family 2	0.244
PF01081	-	KDPG and KHG aldolase	0.241
PF13391	+	HNH endonuclease	0.237
PF13744	-	Helix-turn-helix domain	0.236

PF13434	+	L-lysine 6-monooxygenase (NADPH-requiring)	0.232
PF00296	+	Luciferase-like monooxygenase	0.219
PF11373	+	Protein of unknown function (DUF3175)	0.213
PF08323	+	Starch synthase catalytic domain	0.211
PF11902	+	Protein of unknown function (DUF3422)	0.210
PF10263	+	SprT-like family	0.195
PF03320	-	Bacterial fructose-1,6-bisphosphatase, glpX-encoded	0.195
PF00394	-	Multicopper oxidase	0.192
PF05145	+	Putative ammonia monooxygenase	0.190
PF02219	-	Methylenetetrahydrofolate reductase	0.190
PF01324	+	Diphtheria toxin, R domain	0.182
PF02763	+	Diphtheria toxin, C domain	0.182
PF04402	-	Protein of unknown function (DUF541)	0.181
PF12696	+	TraM recognition site of TraD and TraG	0.179
PF13628	+	Domain of unknown function (DUF4142)	0.177
PF12281	+	Protein of unknown function (DUF3620)	0.170
PF03773	+	Predicted permease	0.169
PF02733	+	Dak1 domain	0.165
PF10387	+	Protein of unknown function (DUF2442)	0.158
PF09413	+	Domain of unknown function (DUF2007)	0.158
PF05988	+	Bacterial protein of unknown function (DUF899)	0.154
PF03613	-	PTS system mannose/fructose/sorbose family IID component	0.153
PF06628	-	Catalase-related immune-responsive	0.149
PF03830	-	PTS system sorbose subfamily IIB component	0.148
PF07748	-	Glycosyl hydrolases family 38 C-terminal domain	0.146
PF00732	-	GMC oxidoreductase	0.139
PF00190	+	Cupin	0.131
PF13470	+	PIN domain	0.131
PF08818	+	Domain of unknown function (DU1801)	0.125
PF03354	+	Phage Terminase	0.124
PF03070	+	TENA/THI-4/PQQC family	0.124
PF01447	-	Thermolysin metallopeptidase, catalytic domain	0.120
PF13676	+	TIR domain	0.115
PF05119	+	Phage terminase, small subunit	0.115
PF00221	-	Aromatic amino acid lyase	0.112
PF04020	+	Membrane protein of unknown function	0.112
PF00484	-	Carbonic anhydrase	0.111
PF13533	-	Biotin-lipoyl like	0.111
PF01476	-	LysM domain	0.109
PF02868	-	Thermolysin metallopeptidase, alpha-helical domain	0.109
PF00670	-	S-adenosyl-L-homocysteine hydrolase, NAD binding domain	0.104
PF06277	-	Ethanolamine utilisation protein EutA	0.102
PF13768	+	von Willebrand factor type A domain	0.098
PF04909	-	Amidohydrolase	0.097
PF13597	+	Anaerobic ribonucleoside-triphosphate reductase	0.093
PF00728	-	Glycosyl hydrolase family 20, catalytic domain	0.093
PF04586	+	Caudovirus prohead protease	0.093
PF11896	+	Domain of unknown function (DUF3416)	0.091

PF05050	+	Methyltransferase FkbM domain	0.091
PF02040	-	Arsenical pump membrane protein	0.076
PF01613	-	Flavin reductase like domain	0.075
PF10672	-	S-adenosylmethionine-dependent methyltransferase	0.073
PF13514	+	AAA domain	0.072
PF01814	-	Hemerythrin HHE cation binding domain	0.071
PF13621	-	Cupin-like domain	0.070
PF03744	+	6-carboxyhexanoate--CoA ligase	0.070
PF01243	-	Pyridoxamine 5'-phosphate oxidase	0.066
PF08392	+	FAE1/Type III polyketide synthase-like protein	0.057
PF02424	-	ApbE family	0.056
PF01074	-	Glycosyl hydrolases family 38 N-terminal domain	0.055
PF13519	-	von Willebrand factor type A domain	0.047
PF01425	+	Amidase	0.029
PF01609	-	Transposase DDE domain	0.019
PF03781	-	Sulfatase-modifying factor enzyme 1	0.015
PF13788	+	Domain of unknown function (DUF4180)	0.012
PF03653	-	Uncharacterised protein family (UPF0093)	0.012
PF00932	+	Lamin Tail Domain	0.012
PF04471	-	Restriction endonuclease	0.011
PF03481	+	Putative GTP-binding controlling metal-binding	0.005
PF13424	-	Tetratricopeptide repeat	-0.009
PF00940	+	DNA-dependent RNA polymerase	-0.010
PF05045	+	Rhamnan synthesis protein F	-0.013
PF00081	-	Iron/manganese superoxide dismutases, alpha-hairpin domain	-0.014
PF02777	-	Iron/manganese superoxide dismutases, C-terminal domain	-0.014
PF02790	-	Cytochrome C oxidase subunit II, transmembrane domain	-0.016
PF13175	-	AAA ATPase domain	-0.018
PF01470	-	Pyroglutamyl peptidase	-0.021
PF07691	-	PA14 domain	-0.021
PF09369	+	Domain of unknown function (DUF1998)	-0.029
PF01882	-	Protein of unknown function DUF58	-0.058
PF07786	-	Protein of unknown function (DUF1624)	-0.058
PF00565	-	Staphylococcal nuclease homologue	-0.081
PF14659	-	Phage integrase, N-terminal SAM-like domain	-0.084
PF01878	-	EVE domain	-0.085
PF01555	-	DNA methylase	-0.097
PF09605	+	Hypothetical bacterial integral membrane protein (Trep_Strep)	-0.102
PF05193	-	Peptidase M16 inactive domain	-0.104
PF02498	+	BRO family, N-terminal domain	-0.123
PF11716	-	Mycothiol maleylpyruvate isomerase N-terminal domain	-0.125
PF04326	-	Divergent AAA domain	-0.125
PF13735	+	tRNA nucleotidyltransferase domain 2 putative	-0.153
PF13289	-	SIR2-like domain	-0.157
PF00274	+	Fructose-bisphosphate aldolase class-I	-0.168

Phenotype: L-Rhamnose (Growth: Sugar), Predictor: phypat

Pfam	class	Pfam_desc	cor

PF06134	+	L-rhamnose isomerase (RhaA)	0.681
PF05336	+	Domain of unknown function (DUF718)	0.647
PF09857	+	Uncharacterized protein conserved in bacteria (DUF2084)	0.511
PF11575	+	FhuF 2Fe-2S C-terminal domain	0.440
PF00061	+	Lipocalin / cytosolic fatty-acid binding protein family	0.440
PF00982	+	Glycosyltransferase family 20	0.439
PF02358	+	Trehalose-phosphatase	0.418
PF07071	+	Protein of unknown function (DUF1341)	0.416
PF04261	+	Dyp-type peroxidase family	0.381
PF08212	+	Lipocalin-like domain	0.376
PF11941	+	Domain of unknown function (DUF3459)	0.371
PF05834	+	Lycopene cyclase protein	0.338
PF08021	+	Siderophore-interacting FAD-binding domain	0.334
PF01904	+	Protein of unknown function DUF72	0.299
PF06249	+	Ethanolamine utilisation protein EutQ	0.290
PF02567	+	Phenazine biosynthesis-like protein	0.281
PF01906	+	Putative heavy-metal-binding	0.247
PF03611	+	PTS system sugar-specific permease component	0.247
PF06152	+	Phage minor capsid protein 2	0.158
PF12008	+	Type I restriction and modification enzyme - subunit R C terminal	0.124
PF03374	+	Phage antirepressor protein KilAC domain	0.114
PF13166	+	AAA domain	0.112
PF05401	-	Nodulation protein S (NodS)	0.046
PF13581	-	Histidine kinase-like ATPase domain	0.046
PF02664	-	S-Ribosylhomocysteinase (LuxS)	-0.001
PF01814	-	Hemerythrin HHE cation binding domain	-0.001
PF13192	-	Thioredoxin domain	-0.007
PF00725	-	3-hydroxyacyl-CoA dehydrogenase, C-terminal domain	-0.011
PF07963	-	Prokaryotic N-terminal methylation motif	-0.013
PF14501	-	GHKL domain	-0.026
PF12738	-	twin BRCT domain	-0.043
PF00529	-	HlyD family secretion protein	-0.044
PF02515	-	CoA-transferase family III	-0.066
PF04548	-	AIG1 family	-0.067
PF02660	-	Glycerol-3-phosphate acyltransferase	-0.068
PF01734	-	Patatin-like phospholipase	-0.070
PF00872	-	Transposase, Mutator family	-0.070
PF06480	-	FtsH Extracellular	-0.077
PF02082	-	Transcriptional regulator	-0.077
PF13519	-	von Willebrand factor type A domain	-0.083
PF00355	-	Rieske [2Fe-2S] domain	-0.089
PF14529	-	Endonuclease-reverse transcriptase	-0.101
PF03547	-	Membrane transport protein	-0.107
PF06081	-	Bacterial protein of unknown function (DUF939)	-0.115
PF14539	-	Domain of unknown function (DUF4442)	-0.115
PF05000	-	RNA polymerase Rpb1, domain 4	-0.116
PF08423	-	Rad51	-0.116
PF00565	-	Staphylococcal nuclease homologue	-0.121

PF12822	-	Protein of unknown function (DUF3816)	-0.122
PF02557	-	D-alanyl-D-alanine carboxypeptidase	-0.123
PF01663	-	Type I phosphodiesterase / nucleotide pyrophosphatase	-0.124
PF03432	-	Relaxase/Mobilisation nuclease domain	-0.140
PF05658	-	Head domain of trimeric autotransporter adhesin	-0.144
PF05662	-	Coiled stalk of trimeric autotransporter adhesin	-0.150
PF04221	-	RelB antitoxin	-0.165
PF01809	-	Haemolytic domain	-0.172
PF01978	-	Sugar-specific transcriptional regulator TrmB	-0.184
PF10881	-	Protein of unknown function (DUF2726)	-0.185
PF04191	-	Phospholipid methyltransferase	-0.221
PF04892	-	VanZ like family	-0.224
PF13495	-	Phage integrase, N-terminal SAM-like domain	-0.231
PF04392	-	ABC transporter substrate binding protein	-0.259
PF07521	-	RNA-metabolising metallo-beta-lactamase	-0.288
PF02637	-	GatB domain	-0.288
PF02934	-	GatB/GatE catalytic domain	-0.288

Phenotype: L-Rhamnose (Growth: Sugar), Predictor: phypat+PGL

Pfam	class	Pfam_desc	cor
PF06134	+	L-rhamnose isomerase (RhaA)	0.681
PF05336	+	Domain of unknown function (DUF718)	0.647
PF09857	+	Uncharacterized protein conserved in bacteria (DUF2084)	0.511
PF06224	+	Winged helix DNA-binding domain	0.464
PF00982	+	Glycosyltransferase family 20	0.439
PF02358	+	Trehalose-phosphatase	0.418
PF07071	+	Protein of unknown function (DUF1341)	0.416
PF05035	+	2-keto-3-deoxy-galactonokinase	0.391
PF04261	+	Dyp-type peroxidase family	0.381
PF06568	+	Domain of unknown function (DUF1127)	0.366
PF09485	+	CRISPR-associated protein Cse2 (CRISPR_cse2)	0.366
PF13496	+	Domain of unknown function (DUF4120)	0.360
PF12408	+	Ribose-5-phosphate isomerase	0.359
PF08021	+	Siderophore-interacting FAD-binding domain	0.334
PF06353	+	Protein of unknown function (DUF1062)	0.334
PF00797	+	N-acetyltransferase	0.322
PF05145	+	Putative ammonia monooxygenase	0.317
PF01904	+	Protein of unknown function DUF72	0.299
PF09617	+	CRISPR-associated protein GSU0053 (Cas_GSU0053)	0.293
PF00161	+	Ribosome inactivating protein	0.293
PF03752	+	Short repeats of unknown function	0.293
PF00135	+	Carboxylesterase family	0.289
PF09907	+	Uncharacterized protein conserved in bacteria (DUF2136)	0.283
PF02567	+	Phenazine biosynthesis-like protein	0.281
PF13375	+	RnfC Barrel sandwich hybrid domain	0.273
PF12852	+	Cupin	0.250
PF11651	+	P22 coat protein - gene protein 5	0.231
PF02485	+	Core-2/I-Branching enzyme	0.219

PF12866	+	Protein of unknown function (DUF3823)	0.218
PF15599	+	Immunity protein 38	0.211
PF05015	+	Plasmid maintenance system killer protein	0.200
PF02055	+	O-Glycosyl hydrolase family 30	0.196
PF02469	+	Fasciclin domain	0.196
PF09314	+	Domain of unknown function (DUF1972)	0.195
PF14568	+	SMI1-KNR4 cell-wall	0.192
PF14031	+	Putative serine dehydratase domain	0.180
PF13701	+	Transposase DDE domain group 1	0.170
PF08803	-	Putative mono-oxygenase ydhR	0.164
PF06152	+	Phage minor capsid protein 2	0.158
PF04466	+	Phage terminase large subunit	0.151
PF07849	+	Protein of unknown function (DUF1641)	0.141
PF10145	+	Phage-related minor tail protein	0.139
PF00891	+	O-methyltransferase	0.138
PF04606	-	Ogr/Delta-like zinc finger	0.136
PF01658	+	Myo-inositol-1-phosphate synthase	0.124
PF08774	+	VRR-NUC domain	0.124
PF06634	+	Protein of unknown function (DUF1156)	0.120
PF03354	+	Phage Terminase	0.113
PF13166	+	AAA domain	0.112
PF13156	+	Restriction endonuclease	0.099
PF13309	-	HTH domain	0.098
PF08348	-	YheO-like PAS domain	0.098
PF01471	-	Putative peptidoglycan binding domain	0.095
PF03241	-	4-hydroxyphenylacetate 3-hydroxylase C terminal	0.090
PF13432	-	Tetratricopeptide repeat	0.089
PF02927	+	N-terminal ig-like domain of cellulase	0.086
PF13007	-	Transposase C of IS166 homeodomain	0.086
PF13333	+	Integrase core domain	0.076
PF01051	-	Initiator Replication protein	0.068
PF11015	+	Protein of unknown function (DUF2853)	0.067
PF08327	+	Activator of Hsp90 ATPase homolog 1-like protein	0.060
PF13470	+	PIN domain	0.053
PF08000	+	Bacterial PH domain	0.053
PF09369	+	Domain of unknown function (DUF1998)	0.052
PF14390	+	Domain of unknown function (DUF4420)	0.051
PF03994	-	Domain of Unknown Function (DUF350)	0.043
PF05133	+	Phage portal protein, SPP1 Gp6-like	0.035
PF01740	-	STAS domain	0.035
PF08818	+	Domain of unknown function (DU1801)	0.034
PF10076	-	Uncharacterized protein conserved in bacteria (DUF2313)	0.034
PF15644	+	Papain fold toxin 1	0.025
PF06736	+	Protein of unknown function (DUF1211)	0.023
PF03572	-	Peptidase family S41	0.018
PF01418	-	Helix-turn-helix domain, rpiR family	0.013
PF08483	+	IstB-like ATP binding N-terminal	0.007
PF00908	-	dTDP-4-dehydrorhamnose 3,5-epimerase	-0.001

PF09932	-	Uncharacterized conserved protein (DUF2164)	-0.006
PF01555	-	DNA methylase	-0.008
PF00735	+	Septin	-0.008
PF08544	-	GHMP kinases C terminal	-0.023
PF05154	-	TM2 domain	-0.023
PF01165	-	Ribosomal protein S21	-0.026
PF05857	-	TraX protein	-0.033
PF00037	-	4Fe-4S binding domain	-0.034
PF12696	+	TraM recognition site of TraD and TraG	-0.043
PF01078	-	Magnesium chelatase, subunit ChlI	-0.052
PF02534	+	Type IV secretory system Conjugative DNA transfer	-0.053
PF13274	+	Protein of unknown function (DUF4065)	-0.055
PF10979	-	Protein of unknown function (DUF2786)	-0.057
PF01734	-	Patatin-like phospholipase	-0.070
PF02082	-	Transcriptional regulator	-0.077
PF02504	-	Fatty acid synthesis protein	-0.084
PF13412	-	Winged helix-turn-helix DNA-binding	-0.086
PF10551	-	MULE transposase domain	-0.108
PF14622	-	Ribonuclease-III-like	-0.109
PF05000	-	RNA polymerase Rpb1, domain 4	-0.116
PF06821	-	Serine hydrolase	-0.117
PF01663	-	Type I phosphodiesterase / nucleotide pyrophosphatase	-0.124
PF02384	-	N-6 DNA Methylase	-0.150
PF01632	-	Ribosomal protein L35	-0.156
PF13289	-	SIR2-like domain	-0.173
PF08874	-	Domain of unknown function (DUF1835)	-0.178
PF10881	-	Protein of unknown function (DUF2726)	-0.185
PF13421	-	SPFH domain-Band 7 family	-0.194
PF13452	-	N-terminal half of MaoC dehydratase	-0.195
PF13495	-	Phage integrase, N-terminal SAM-like domain	-0.231

Phenotype: Maltose (Growth: Sugar), Predictor: phypat

Pfam	class	Pfam_desc	cor
PF00251	+	Glycosyl hydrolases family 32 N-terminal domain	0.567
PF01228	+	Glycine radical	0.554
PF01238	+	Phosphomannose isomerase type I	0.531
PF02744	+	Galactose-1-phosphate uridyl transferase, C-terminal domain	0.509
PF02837	+	Glycosyl hydrolases family 2, sugar binding domain	0.489
PF03613	+	PTS system mannose/fructose/sorbose family IID component	0.489
PF08244	+	Glycosyl hydrolases family 32 C terminal	0.489
PF00356	+	Bacterial regulatory proteins, lacI family	0.478
PF13377	+	Periplasmic binding protein-like domain	0.448
PF00343	+	Carbohydrate phosphorylase	0.443
PF00401	+	ATP synthase, Delta/Epsilon chain, long alpha-helix domain	0.437
PF06054	+	Competence protein CoiA-like family	0.434
PF02903	+	Alpha amylase, N-terminal ig-like domain	0.404
PF00230	+	Major intrinsic protein	0.399
PF01418	+	Helix-turn-helix domain, rpiR family	0.385

PF01773	+	Na+ dependent nucleoside transporter N-terminus	0.383
PF07662	+	Na+ dependent nucleoside transporter C-terminus	0.383
PF01055	+	Glycosyl hydrolases family 31	0.383
PF07702	+	UTRA domain	0.374
PF04973	+	Nicotinamide mononucleotide transporter	0.369
PF02446	+	4-alpha-glucanotransferase	0.359
PF11762	+	L-arabinose isomerase C-terminal domain	0.358
PF02610	+	L-arabinose isomerase	0.358
PF13802	+	Galactose mutarotase-like	0.349
PF03610	+	PTS system fructose IIA component	0.344
PF09922	+	Cell wall-active antibiotics response protein (DUF2154)	0.341
PF13735	+	tRNA nucleotidyltransferase domain 2 putative	0.340
PF00854	+	POT family	0.336
PF03714	+	Bacterial pullanase-associated domain	0.334
PF05651	+	Putative sugar diacid recognition	0.329
PF02475	+	Met-10+ like-protein	0.327
PF01915	+	Glycosyl hydrolase family 3 C-terminal domain	0.323
PF01643	+	Acyl-ACP thioesterase	0.322
PF03633	+	Glycosyl hydrolase family 65, C-terminal domain	0.321
PF01943	+	Polysaccharide biosynthesis protein	0.316
PF11734	+	TilS substrate C-terminal domain	0.312
PF03596	+	Cadmium resistance transporter	0.305
PF12481	+	Aluminium induced protein	0.300
PF13439	+	Glycosyltransferase Family 4	0.298
PF04993	+	TfoX N-terminal domain	0.298
PF11188	+	Protein of unknown function (DUF2975)	0.293
PF02424	+	ApbE family	0.277
PF02074	+	Carboxypeptidase Taq (M32) metallopeptidase	0.271
PF07022	+	Bacteriophage CI repressor helix-turn-helix domain	0.268
PF05135	+	Phage gp6-like head-tail connector protein	0.255
PF02796	+	Helix-turn-helix domain of resolvase	0.254
PF03799	+	Cell division protein FtsQ	0.248
PF07853	+	Protein of unknown function (DUF1648)	0.239
PF04230	+	Polysaccharide pyruvyl transferase	0.238
PF10711	+	Hypothetical protein (DUF2513)	0.233
PF07275	+	Antirestriction protein (ArdA)	0.231
PF08274	+	PhnA Zinc-Ribbon	0.229
PF03174	+	Chitobiase/beta-hexosaminidase C-terminal domain	0.224
PF09019	+	EcoRII C terminal	0.218
PF03406	+	Phage tail fibre repeat	0.213
PF13483	+	Beta-lactamase superfamily domain	0.208
PF13333	+	Integrase core domain	0.206
PF00328	+	Histidine phosphatase superfamily (branch 2)	0.205
PF05857	+	TraX protein	0.192
PF04860	+	Phage portal protein	0.190
PF06953	+	Arsenical resistance operon trans-acting repressor ArsD	0.189
PF02225	+	PA domain	0.186
PF11575	+	FhuF 2Fe-2S C-terminal domain	0.166

PF09299	+	Mu transposase, C-terminal	0.158
PF13620	+	Carboxypeptidase regulatory-like domain	0.157
PF07784	+	Protein of unknown function (DUF1622)	0.149
PF00697	+	N-(5'phosphoribosyl)anthranilate (PRA) isomerase	0.148
PF03612	-	Sorbitol phosphotransferase enzyme II N-terminus	0.147
PF07221	+	N-acylglucosamine 2-epimerase (GlcNAc 2-epimerase)	0.146
PF08463	+	EcoEI R protein C-terminal	0.144
PF00908	+	dTDP-4-dehydrorhamnose 3,5-epimerase	0.127
PF02870	+	6-O-methylguanine DNA methyltransferase, ribonuclease-like domain	0.117
PF05713	-	Bacterial mobilisation protein (MobC)	0.115
PF02652	+	L-lactate permease	0.099
PF01758	+	Sodium Bile acid symporter family	0.097
PF05954	+	Phage late control gene D protein (GPD)	0.095
PF00246	+	Zinc carboxypeptidase	0.090
PF04286	+	Protein of unknown function (DUF445)	0.089
PF02535	+	ZIP Zinc transporter	0.080
PF00782	+	Dual specificity phosphatase, catalytic domain	0.072
PF01165	-	Ribosomal protein S21	0.062
PF13346	-	ABC-2 family transporter protein	0.048
PF03374	-	Phage antirepressor protein KilAC domain	0.035
PF13435	+	Cytochrome c554 and c-prime	0.034
PF07683	+	Cobalamin synthesis protein cobW C-terminal domain	-0.001
PF13278	-	Putative amidotransferase	-0.004
PF07927	-	YcfA-like protein	-0.008
PF02608	-	Basic membrane protein	-0.017
PF02502	-	Ribose/Galactose Isomerase	-0.020
PF02894	-	Oxidoreductase family, C-terminal alpha/beta domain	-0.031
PF02133	-	Permease for cytosine/purines, uracil, thiamine, allantoin	-0.048
PF07804	-	HipA-like C-terminal domain	-0.052
PF03432	-	Relaxase/Mobilisation nuclease domain	-0.053
PF05015	-	Plasmid maintenance system killer protein	-0.055
PF00627	-	UBA/TS-N domain	-0.061
PF03729	-	Short repeat of unknown function (DUF308)	-0.071
PF00467	-	KOW motif	-0.077
PF11974	-	Alpha-2-macroglobulin MG1 domain	-0.082
PF14305	-	TupA-like ATPgrasp	-0.090
PF10417	-	C-terminal domain of 1-Cys peroxiredoxin	-0.101
PF07331	-	Tripartite tricarboxylate transporter TctB family	-0.109
PF02575	-	YbaB/EbfC DNA-binding family	-0.113
PF02423	-	Ornithine cyclodeaminase/mu-crystallin family	-0.117
PF01729	-	Quinolinate phosphoribosyl transferase, C-terminal domain	-0.121
PF02615	-	Malate/L-lactate dehydrogenase	-0.129
PF07969	-	Amidohydrolase family	-0.131
PF02040	-	Arsenical pump membrane protein	-0.133
PF02436	-	Conserved carboxylase domain	-0.134
PF01558	-	Pyruvate ferredoxin/flavodoxin oxidoreductase	-0.135
PF02749	-	Quinolinate phosphoribosyl transferase, N-terminal domain	-0.142
PF04670	-	Gtr1/RagA G protein conserved region	-0.158

PF01206	-	Sulfurtransferase TusA	-0.160
PF01175	-	Urocanase	-0.171
PF04228	-	Putative neutral zinc metallopeptidase	-0.181
PF07726	-	ATPase family associated with various cellular activities (AAA)	-0.196
PF02464	-	Competence-damaged protein	-0.198
PF00725	-	3-hydroxyacyl-CoA dehydrogenase, C-terminal domain	-0.214
PF01243	-	Pyridoxamine 5'-phosphate oxidase	-0.214
PF13591	-	MerR HTH family regulatory protein	-0.215
PF06965	-	Na+/H+ antiporter 1	-0.215
PF13482	-	RNase_H superfamily	-0.224
PF02503	-	Polyphosphate kinase middle domain	-0.229
PF01769	-	Divalent cation transporter	-0.229
PF01613	-	Flavin reductase like domain	-0.229
PF01957	-	NfeD-like C-terminal, partner-binding	-0.239
PF03308	-	ArgK protein	-0.241
PF02277	-	Phosphoribosyltransferase	-0.244
PF05853	-	Prokaryotic protein of unknown function (DUF849)	-0.250
PF07866	-	Protein of unknown function (DUF1653)	-0.251
PF02934	-	GatB/GatE catalytic domain	-0.255
PF02637	-	GatB domain	-0.255
PF04536	-	TLP18.3, Psb32 and MOLO-1 founding proteins of phosphatase	-0.268
PF01258	-	Prokaryotic dksA/traR C4-type zinc finger	-0.278
PF07568	-	Histidine kinase	-0.285
PF10588	-	NADH-ubiquinone oxidoreductase-G iron-sulfur binding region	-0.338
PF02515	-	CoA-transferase family III	-0.341
PF10589	-	NADH-ubiquinone oxidoreductase-F iron-sulfur binding region	-0.355
PF13335	-	Magnesium chelatase, subunit ChlI	-0.373

Phenotype: Maltose (Growth: Sugar), Predictor: phypat+PGL

Pfam	class	Pfam_desc	cor
PF00251	+	Glycosyl hydrolases family 32 N-terminal domain	0.567
PF01228	+	Glycine radical	0.554
PF01238	+	Phosphomannose isomerase type I	0.531
PF05116	+	Sucrose-6F-phosphate phosphohydrolase	0.527
PF01263	+	Aldose 1-epimerase	0.508
PF02836	+	Glycosyl hydrolases family 2, TIM barrel domain	0.500
PF02837	+	Glycosyl hydrolases family 2, sugar binding domain	0.489
PF03932	+	CutC family	0.461
PF00532	+	Periplasmic binding proteins and sugar binding domain of LacI family	0.451
PF13377	+	Periplasmic binding protein-like domain	0.448
PF00230	+	Major intrinsic protein	0.399
PF02449	+	Beta-galactosidase	0.390
PF01418	+	Helix-turn-helix domain, rpiR family	0.385
PF00393	+	6-phosphogluconate dehydrogenase, C-terminal domain	0.383
PF07702	+	UTRA domain	0.374
PF08211	+	Cytidine and deoxycytidylate deaminase zinc-binding region	0.369
PF00186	+	Dihydrofolate reductase	0.364
PF02446	+	4-alpha-glucanotransferase	0.359

PF03610	+	PTS system fructose IIA component	0.344
PF13735	+	tRNA nucleotidyltransferase domain 2 putative	0.340
PF00854	+	POT family	0.336
PF13189	+	Cytidylate kinase-like family	0.330
PF01915	+	Glycosyl hydrolase family 3 C-terminal domain	0.323
PF05656	+	Protein of unknown function (DUF805)	0.322
PF03633	+	Glycosyl hydrolase family 65, C-terminal domain	0.321
PF01943	+	Polysaccharide biosynthesis protein	0.316
PF11734	+	TilS substrate C-terminal domain	0.312
PF03596	+	Cadmium resistance transporter	0.305
PF03965	+	Penicillinase repressor	0.299
PF13439	+	Glycosyltransferase Family 4	0.298
PF06993	+	Protein of unknown function (DUF1304)	0.289
PF07831	+	Pyrimidine nucleoside phosphorylase C-terminal domain	0.284
PF02457	+	DisA bacterial checkpoint controller nucleotide-binding	0.277
PF01183	+	Glycosyl hydrolases family 25	0.274
PF10111	+	Glycosyltransferase like family 2	0.252
PF03799	+	Cell division protein FtsQ	0.248
PF00194	+	Eukaryotic-type carbonic anhydrase	0.239
PF04230	+	Polysaccharide pyruvyl transferase	0.238
PF00258	+	Flavodoxin	0.236
PF13185	+	GAF domain	0.233
PF07275	+	Antirestriction protein (ArdA)	0.231
PF13477	+	Glycosyl transferase 4-like	0.223
PF13413	+	Helix-turn-helix domain	0.218
PF03406	+	Phage tail fibre repeat	0.213
PF13333	+	Integrase core domain	0.206
PF07799	+	Protein of unknown function (DUF1643)	0.203
PF02899	+	Phage integrase, N-terminal SAM-like domain	0.199
PF01676	+	Metalloenzyme superfamily	0.197
PF03141	+	Putative S-adenosyl-L-methionine-dependent methyltransferase	0.196
PF12724	+	Flavodoxin domain	0.195
PF06414	+	Zeta toxin	0.193
PF05198	+	Translation initiation factor IF-3, N-terminal domain	0.190
PF04860	+	Phage portal protein	0.190
PF13617	+	YnbE-like lipoprotein	0.187
PF04245	-	37-kD nucleoid-associated bacterial protein	0.184
PF03632	+	Glycosyl hydrolase family 65 central catalytic domain	0.183
PF14840	+	Processivity clamp loader gamma complex DNA pol III C-term	0.173
PF11575	+	FhuF 2Fe-2S C-terminal domain	0.166
PF00481	-	Protein phosphatase 2C	0.161
PF04892	+	VanZ like family	0.158
PF09299	+	Mu transposase, C-terminal	0.158
PF04326	+	Divergent AAA domain	0.156
PF09983	+	Uncharacterized protein conserved in bacteria C-term(DUF2220)	0.155
PF01276	-	Orn/Lys/Arg decarboxylase, major domain	0.154
PF05065	+	Phage capsid family	0.153
PF02368	-	Bacterial Ig-like domain (group 2)	0.149

PF04023	+	FeoA domain	0.148
PF07739	+	TipAS antibiotic-recognition domain	0.147
PF00676	+	Dehydrogenase E1 component	0.145
PF09838	+	Uncharacterized protein conserved in bacteria (DUF2065)	0.145
PF08924	+	Domain of unknown function (DUF1906)	0.137
PF07369	-	Protein of unknown function (DUF1488)	0.137
PF06081	-	Bacterial protein of unknown function (DUF939)	0.135
PF03845	+	Spore germination protein	0.129
PF13484	+	4Fe-4S double cluster binding domain	0.128
PF00245	+	Alkaline phosphatase	0.127
PF13671	+	AAA domain	0.126
PF05896	+	Na(+)-translocating NADH-quinone reductase subunit A (NQRA)	0.121
PF02870	+	6-O-methylguanine DNA methyltransferase, ribonuclease-like domain	0.117
PF03070	+	TENA/THI-4/PQQC family	0.117
PF06166	+	Protein of unknown function (DUF979)	0.116
PF06149	+	Protein of unknown function (DUF969)	0.116
PF13240	-	zinc-ribbon domain	0.114
PF01978	+	Sugar-specific transcriptional regulator TrmB	0.112
PF05521	+	Phage head-tail joining protein	0.111
PF02146	-	Sir2 family	0.110
PF14690	+	zinc-finger of transposase IS204/IS1001/IS1096/IS1165	0.109
PF03747	+	ADP-ribosylglycohydrolase	0.106
PF07885	+	Ion channel	0.102
PF08713	+	DNA alkylation repair enzyme	0.101
PF05569	+	BlaR1 peptidase M56	0.100
PF02652	+	L-lactate permease	0.099
PF14595	+	Thioredoxin	0.098
PF01758	+	Sodium Bile acid symporter family	0.097
PF05954	+	Phage late control gene D protein (GPD)	0.095
PF13310	+	Virulence protein RhuM family	0.090
PF13551	+	Winged helix-turn helix	0.089
PF04286	+	Protein of unknown function (DUF445)	0.089
PF00710	+	Asparaginase	0.085
PF02535	+	ZIP Zinc transporter	0.080
PF13565	+	Homeodomain-like domain	0.079
PF06144	+	DNA polymerase III, delta subunit	0.079
PF13727	+	CoA-binding domain	0.077
PF01704	-	UTP--glucose-1-phosphate uridylyltransferase	0.076
PF04392	+	ABC transporter substrate binding protein	0.071
PF13350	-	Tyrosine phosphatase family	0.068
PF09704	+	CRISPR-associated protein (Cas_Cas5)	0.065
PF09587	+	Bacterial capsule synthesis protein PGA_cap	0.062
PF05437	-	Branched-chain amino acid transport protein (AzlD)	0.061
PF02219	-	Methylenetetrahydrofolate reductase	0.061
PF00891	-	O-methyltransferase	0.056
PF14907	+	Uncharacterised nucleotidyltransferase	0.051
PF13466	-	STAS domain	0.049
PF02958	-	Ecdysteroid kinase	0.047

PF13418	-	Galactose oxidase, central domain	0.046
PF05621	+	Bacterial TniB protein	0.046
PF13380	-	CoA binding domain	0.044
PF09519	+	HindVP restriction endonuclease	0.043
PF06865	+	Protein of unknown function (DUF1255)	0.037
PF03374	-	Phage antirepressor protein KilAC domain	0.035
PF03703	-	Bacterial PH domain	0.034
PF07503	+	HypF finger	0.034
PF05717	-	IS66 Orf2 like protein	0.030
PF13480	-	Acetyltransferase (GNAT) domain	0.029
PF01869	-	BadF/BadG/BcrA/BcrD ATPase family	0.023
PF05145	-	Putative ammonia monooxygenase	0.019
PF13438	-	Domain of unknown function (DUF4113)	0.018
PF07291	+	Methylamine utilisation protein MauE	0.018
PF09718	+	Lambda phage tail tape-measure protein (Tape_meas_lam_C)	0.018
PF07110	+	EthD domain	0.012
PF00218	-	Indole-3-glycerol phosphate synthase	0.011
PF00939	+	Sodium:sulfate symporter transmembrane region	0.010
PF10397	-	Adenylosuccinate lyase C-terminus	0.006
PF04715	-	Anthranilate synthase component I, N terminal region	0.002
PF00933	-	Glycosyl hydrolase family 3 N terminal domain	0.000
PF02498	-	BRO family, N-terminal domain	-0.003
PF07729	-	FCD domain	-0.006
PF02579	+	Dinitrogenase iron-molybdenum cofactor	-0.013
PF13924	+	Lipocalin-like domain	-0.018
PF12843	-	Protein of unknown function (DUF3820)	-0.021
PF04343	-	Protein of unknown function, DUF488	-0.023
PF12867	-	DinB superfamily	-0.027
PF13305	-	WHG domain	-0.033
PF07719	-	Tetratricopeptide repeat	-0.034
PF14090	+	Helix-turn-helix domain	-0.034
PF05016	-	Plasmid stabilisation system protein	-0.034
PF04480	+	Protein of unknown function (DUF559)	-0.038
PF14691	-	Dihydroprymidine dehydrogenase domain II, 4Fe-4S cluster	-0.042
PF00682	-	HMGL-like	-0.044
PF06032	+	Protein of unknown function (DUF917)	-0.045
PF02133	-	Permease for cytosine/purines, uracil, thiamine, allantoin	-0.048
PF12852	-	Cupin	-0.050
PF05015	-	Plasmid maintenance system killer protein	-0.055
PF10011	+	Predicted membrane protein (DUF2254)	-0.058
PF14489	-	QueF-like protein	-0.059
PF01555	-	DNA methylase	-0.061
PF02909	-	Tetracyclin repressor, C-terminal all-alpha domain	-0.063
PF14765	-	Polyketide synthase dehydratase	-0.064
PF13714	+	Phosphoenolpyruvate phosphomutase	-0.077
PF01619	-	Proline dehydrogenase	-0.077
PF10388	+	EAL-domain associated signalling protein domain	-0.079
PF01597	-	Glycine cleavage H-protein	-0.080

PF06267	-	Family of unknown function (DUF1028)	-0.080
PF13581	-	Histidine kinase-like ATPase domain	-0.090
PF00733	+	Asparagine synthase	-0.091
PF08643	-	Fungal family of unknown function (DUF1776)	-0.091
PF03994	-	Domain of Unknown Function (DUF350)	-0.104
PF01888	+	CbiD	-0.113
PF03707	-	Bacterial signalling protein N terminal repeat	-0.114
PF02423	-	Ornithine cyclodeaminase/mu-crystallin family	-0.117
PF01740	-	STAS domain	-0.117
PF01292	+	Prokaryotic cytochrome b561	-0.118
PF01841	-	Transglutaminase-like superfamily	-0.118
PF03350	-	Uncharacterized protein family, UPF0114	-0.120
PF12625	-	Arabinose-binding domain of AraC transcription regulator, N-term	-0.121
PF02814	-	UreE urease accessory protein, N-terminal domain	-0.126
PF01208	-	Uroporphyrinogen decarboxylase (URO-D)	-0.127
PF07969	-	Amidohydrolase family	-0.131
PF02040	-	Arsenical pump membrane protein	-0.133
PF14532	+	Sigma-54 interaction domain	-0.137
PF02586	-	Uncharacterised ACR, COG2135	-0.137
PF00732	-	GMC oxidoreductase	-0.142
PF13406	-	Transglycosylase SLT domain	-0.152
PF00071	-	Ras family	-0.159
PF07411	-	Domain of unknown function (DUF1508)	-0.162
PF04342	+	Protein of unknown function, DUF486	-0.164
PF01175	-	Urocanase	-0.171
PF01810	-	LysE type translocator	-0.171
PF13768	-	von Willebrand factor type A domain	-0.180
PF04228	-	Putative neutral zinc metallopeptidase	-0.181
PF02310	-	B12 binding domain	-0.186
PF02667	-	Short chain fatty acid transporter	-0.189
PF03865	-	Haemolysin secretion/activation protein ShlB/FhaC/HecB	-0.193
PF13356	-	Domain of unknown function (DUF4102)	-0.193
PF12900	-	Pyridoxamine 5'-phosphate oxidase	-0.197
PF07287	-	Protein of unknown function (DUF1446)	-0.198
PF03435	-	Saccharopine dehydrogenase	-0.199
PF00725	-	3-hydroxyacyl-CoA dehydrogenase, C-terminal domain	-0.214
PF01243	-	Pyridoxamine 5'-phosphate oxidase	-0.214
PF06271	-	RDD family	-0.223
PF01769	-	Divalent cation transporter	-0.229
PF01613	-	Flavin reductase like domain	-0.229
PF03190	-	Protein of unknown function, DUF255	-0.236
PF14241	-	Domain of unknown function (DUF4341)	-0.237
PF03572	-	Peptidase family S41	-0.237
PF01957	-	NfeD-like C-terminal, partner-binding	-0.239
PF14031	-	Putative serine dehydratase domain	-0.241
PF05853	-	Prokaryotic protein of unknown function (DUF849)	-0.250
PF13362	-	Toprim domain	-0.254
PF02913	-	FAD linked oxidases, C-terminal domain	-0.258

PF01258	-	Prokaryotic dksA/traR C4-type zinc finger	-0.278
PF02515	-	CoA-transferase family III	-0.341
PF10589	-	NADH-ubiquinone oxidoreductase-F iron-sulfur binding region	-0.355
PF13706	-	PepSY-associated TM helix	-0.363

Phenotype: Melibiose (Growth: Sugar), Predictor: phypat

Pfam	class	Pfam_desc	cor
PF07745	+	Glycosyl hydrolase family 53	0.640
PF02610	+	L-arabinose isomerase	0.580
PF11762	+	L-arabinose isomerase C-terminal domain	0.580
PF02065	+	Melibiase	0.542
PF02449	+	Beta-galactosidase	0.537
PF01055	+	Glycosyl hydrolases family 31	0.523
PF05592	+	Bacterial alpha-L-rhamnosidase	0.511
PF14310	+	Fibronectin type III-like domain	0.465
PF06134	+	L-rhamnose isomerase (RhaA)	0.444
PF07929	+	Plasmid pRiA4b ORF-3-like protein	0.439
PF11533	+	Protein of unknown function (DUF3225)	0.394
PF00909	+	Ammonium Transporter Family	0.374
PF00135	+	Carboxylesterase family	0.373
PF02614	+	Glucuronate isomerase	0.348
PF05336	+	Domain of unknown function (DUF718)	0.336
PF08379	+	Bacterial transglutaminase-like N-terminal region	0.317
PF11700	+	Vacuole effluxer Atg22 like	0.315
PF03649	+	Uncharacterised protein family (UPF0014)	0.307
PF02586	+	Uncharacterised ACR, COG2135	0.298
PF07662	+	Na+ dependent nucleoside transporter C-terminus	0.291
PF01773	+	Na+ dependent nucleoside transporter N-terminus	0.291
PF09848	+	Uncharacterized conserved protein (DUF2075)	0.286
PF06175	+	tRNA-(MS[2]IO[6]A)-hydroxylase (MiaE)	0.276
PF02659	+	Domain of unknown function DUF	0.274
PF01223	+	DNA/RNA non-specific endonuclease	0.273
PF03869	+	Arc-like DNA binding domain	0.254
PF01988	+	VIT family	0.252
PF13587	+	N-terminal domain of DJ-1_PfpI family	0.242
PF14534	+	Domain of unknown function (DUF4440)	0.241
PF03109	+	ABC1 family	0.239
PF08223	+	PaaX-like protein C-terminal domain	0.237
PF01427	+	D-ala-D-ala dipeptidase	0.201
PF07853	+	Protein of unknown function (DUF1648)	0.162
PF02274	-	Amidinotransferase	-0.026
PF02350	-	UDP-N-acetylglucosamine 2-epimerase	-0.027
PF00455	-	DeoR C terminal sensor domain	-0.040
PF05866	-	Endodeoxyribonuclease RusA	-0.054
PF04326	-	Divergent AAA domain	-0.066
PF01609	-	Transposase DDE domain	-0.074
PF13521	-	AAA domain	-0.116
PF08843	-	Nucleotidyl transferase of unknown function (DUF1814)	-0.117

PF00891	-	O-methyltransferase	-0.130
PF09084	-	NMT1/THI5 like	-0.139
PF00939	-	Sodium:sulfate symporter transmembrane region	-0.162
PF13424	-	Tetratricopeptide repeat	-0.168
PF01637	-	Archaeal ATPase	-0.181
PF04313	-	Type I restriction enzyme R protein N terminus (HSDR_N)	-0.185
PF02608	-	Basic membrane protein	-0.198
PF00071	-	Ras family	-0.212
PF03916	-	Polysulphide reductase, NrfD	-0.216
PF01555	-	DNA methylase	-0.220
PF12161	-	HsdM N-terminal domain	-0.230
PF01891	-	Cobalt uptake substrate-specific transmembrane region	-0.236
PF00188	-	Cysteine-rich secretory protein family	-0.251
PF00420	-	NADH-ubiquinone/plastoquinone oxidoreductase chain 4L	-0.255
PF07021	-	Methionine biosynthesis protein MetW	-0.257
PF13857	-	Ankyrin repeats (many copies)	-0.257
PF01235	-	Sodium:alanine symporter family	-0.264
PF04325	-	Protein of unknown function (DUF465)	-0.264
PF03572	-	Peptidase family S41	-0.307
PF02502	-	Ribose/Galactose Isomerase	-0.322
PF04191	-	Phospholipid methyltransferase	-0.337
PF00209	-	Sodium:neurotransmitter symporter family	-0.339

Phenotype: Melibiose (Growth: Sugar), Predictor: phypat+PGL

Pfam	class	Pfam_desc	cor
PF08532	+	Beta-galactosidase trimerisation domain	0.599
PF02065	+	Melibiase	0.542
PF02449	+	Beta-galactosidase	0.537
PF05592	+	Bacterial alpha-L-rhamnosidase	0.511
PF04616	+	Glycosyl hydrolases family 43	0.487
PF02929	+	Beta galactosidase small chain	0.470
PF05870	+	Phenolic acid decarboxylase (PAD)	0.456
PF06134	+	L-rhamnose isomerase (RhaA)	0.444
PF07929	+	Plasmid pRiA4b ORF-3-like protein	0.439
PF11175	+	Protein of unknown function (DUF2961)	0.404
PF13636	+	pre-rRNA processing and ribosome biogenesis	0.394
PF01306	+	LacY proton/sugar symporter	0.384
PF07748	+	Glycosyl hydrolases family 38 C-terminal domain	0.384
PF13811	+	Domain of unknown function (DUF4186)	0.382
PF00909	+	Ammonium Transporter Family	0.374
PF09363	+	XFP C-terminal domain	0.355
PF03894	+	D-xylulose 5-phosphate/D-fructose 6-phosphate phosphoketolase	0.355
PF02614	+	Glucuronate isomerase	0.348
PF03051	+	Peptidase C1-like family	0.343
PF14897	+	EpsG family	0.341
PF05336	+	Domain of unknown function (DUF718)	0.336
PF03881	+	Fructosamine kinase	0.331
PF11622	+	Protein of unknown function (DUF3251)	0.328

PF13473	+	Cupredoxin-like domain	0.318
PF11700	+	Vacuole effluxer Atg22 like	0.315
PF03649	+	Uncharacterised protein family (UPF0014)	0.307
PF02586	+	Uncharacterised ACR, COG2135	0.298
PF09250	+	Bifunctional DNA primase/polymerase, N-terminal	0.292
PF07662	+	Na+ dependent nucleoside transporter C-terminus	0.291
PF09364	+	XFP N-terminal domain	0.288
PF08244	+	Glycosyl hydrolases family 32 C terminal	0.286
PF01301	+	Glycosyl hydrolases family 35	0.273
PF01223	+	DNA/RNA non-specific endonuclease	0.273
PF13565	+	Homeodomain-like domain	0.260
PF13461	+	Cell-wall surface anchor repeat	0.253
PF01988	+	VIT family	0.252
PF13587	+	N-terminal domain of DJ-1_PfpI family	0.242
PF06210	+	Protein of unknown function (DUF1003)	0.234
PF10022	-	Uncharacterized protein conserved in bacteria (DUF2264)	0.231
PF06800	+	Sugar transport protein	0.220
PF03390	+	2-hydroxycarboxylate transporter family	0.215
PF01427	+	D-ala-D-ala dipeptidase	0.201
PF02275	+	Linear amide C-N hydrolases, choloylglycine hydrolase family	0.189
PF12320	+	Type 5 capsule protein repressor C-terminal domain	0.189
PF01634	+	ATP phosphoribosyltransferase	0.173
PF00815	+	Histidinol dehydrogenase	0.173
PF00475	+	Imidazoleglycerol-phosphate dehydratase	0.173
PF07731	-	Multicopper oxidase	0.169
PF12674	+	Putative zinc ribbon domain	0.162
PF04029	+	2-phosphosulpholactate phosphatase	0.158
PF07099	+	Protein of unknown function (DUF1361)	0.154
PF01610	+	Transposase	0.153
PF09382	-	RQC domain	0.145
PF08887	+	GAD-like domain	0.129
PF13468	+	Glyoxalase-like domain	0.128
PF04138	-	GtrA-like protein	0.127
PF02371	-	Transposase IS116/IS110/IS902 family	0.123
PF01921	+	tRNA synthetases class I (K)	0.114
PF00543	+	Nitrogen regulatory protein P-II	0.088
PF13480	-	Acetyltransferase (GNAT) domain	0.079
PF13306	+	Leucine rich repeats (6 copies)	0.074
PF04464	-	CDP-Glycerol:Poly(glycerophosphate) glycerophosphotransferase	0.072
PF02423	+	Ornithine cyclodeaminase/mu-crystallin family	0.069
PF00591	+	Glycosyl transferase family, a/b domain	0.069
PF13378	-	Enolase C-terminal domain-like	0.060
PF00697	+	N-(5'phosphoribosyl)anthranilate (PRA) isomerase	0.057
PF01257	+	Thioredoxin-like [2Fe-2S] ferredoxin	0.045
PF01174	+	SNO glutamine amidotransferase family	0.045
PF13345	-	Domain of unknown function (DUF4098)	0.031
PF07476	-	Methylaspartate ammonia-lyase C-terminus	0.022
PF13800	+	Sigma factor regulator N-terminal	0.019

PF10110	-	Membrane domain of glycerophosphoryl diester phosphodiesterase	0.018
PF05015	-	Plasmid maintenance system killer protein	0.013
PF11611	+	Domain of unknown function (DUF4352)	0.007
PF01844	-	HNH endonuclease	0.003
PF02302	-	PTS system, Lactose/Cellobiose specific IIB subunit	-0.002
PF03083	-	Sugar efflux transporter for intercellular exchange	-0.005
PF03610	-	PTS system fructose IIA component	-0.017
PF13509	-	S1 domain	-0.022
PF13643	-	Domain of unknown function (DUF4145)	-0.024
PF01192	-	RNA polymerase Rpb6	-0.051
PF05866	-	Endodeoxyribonuclease RusA	-0.054
PF04198	-	Putative sugar-binding domain	-0.054
PF02686	-	Glu-tRNAGln amidotransferase C subunit	-0.058
PF10397	-	Adenylosuccinate lyase C-terminus	-0.062
PF03432	-	Relaxase/Mobilisation nuclease domain	-0.066
PF03848	-	Tellurite resistance protein TehB	-0.075
PF08351	-	Domain of unknown function (DUF1726)	-0.091
PF06081	-	Bacterial protein of unknown function (DUF939)	-0.094
PF03743	-	Bacterial conjugation TrbI-like protein	-0.099
PF03934	-	Type II secretion system (T2SS), protein K	-0.115
PF04014	-	Antidote-toxin recognition MazE	-0.118
PF01471	-	Putative peptidoglycan binding domain	-0.119
PF05194	-	UreE urease accessory protein, C-terminal domain	-0.120
PF00033	+	Cytochrome b(N-terminal)/b6/petB	-0.126
PF00359	-	Phosphoenolpyruvate-dependent sugar phosphotransferase system, EIIA 2	-0.126
PF03781	-	Sulfatase-modifying factor enzyme 1	-0.137
PF09084	-	NMT1/THI5 like	-0.139
PF02814	-	UreE urease accessory protein, N-terminal domain	-0.145
PF13424	-	Tetratricopeptide repeat	-0.168
PF07521	-	RNA-metabolising metallo-beta-lactamase	-0.196
PF03916	-	Polysulphide reductase, NrfD	-0.216
PF01555	-	DNA methylase	-0.220
PF00565	-	Staphylococcal nuclease homologue	-0.236
PF03572	-	Peptidase family S41	-0.307

Phenotype: myo-Inositol (Growth: Sugar), Predictor: phypat

Pfam	class	Pfam_desc	cor
PF09347	+	Domain of unknown function (DUF1989)	0.568
PF09349	+	OHCU decarboxylase	0.526
PF01958	+	Domain of unknown function DUF108	0.523
PF05995	+	Cysteine dioxygenase type I	0.511
PF08521	+	Two-component sensor kinase N-terminal	0.501
PF06267	+	Family of unknown function (DUF1028)	0.501
PF04982	+	HPP family	0.494
PF02909	+	Tetracyclin repressor, C-terminal all-alpha domain	0.462
PF14031	+	Putative serine dehydratase domain	0.451
PF05063	+	MT-A70	0.438
PF02694	+	Uncharacterised BCR, YnfA/UPF0060 family	0.425

PF12708	+	Pectate lyase superfamily protein	0.401
PF04962	+	KduI/IolB family	0.387
PF05893	+	Acyl-CoA reductase (LuxC)	0.384
PF05145	+	Putative ammonia monooxygenase	0.371
PF11794	+	4-hydroxyphenylacetate 3-hydroxylase N terminal	0.299
PF03241	+	4-hydroxyphenylacetate 3-hydroxylase C terminal	0.299
PF06912	+	Protein of unknown function (DUF1275)	0.292
PF07786	-	Protein of unknown function (DUF1624)	-0.050
PF01814	-	Hemerythrin HHE cation binding domain	-0.055
PF08238	-	Sel1 repeat	-0.059
PF01906	-	Putative heavy-metal-binding	-0.064
PF08003	-	Protein of unknown function (DUF1698)	-0.096
PF05157	-	Type II secretion system (T2SS), protein E, N-terminal domain	-0.096
PF01235	-	Sodium:alanine symporter family	-0.127
PF00142	-	4Fe-4S iron sulfur cluster binding proteins, NifH/frxC family	-0.155
PF09829	-	Uncharacterized protein conserved in bacteria (DUF2057)	-0.159
PF12323	-	Helix-turn-helix domain	-0.180
PF07751	-	Abi-like protein	-0.183
PF14492	-	Elongation Factor G, domain II	-0.196
PF07228	-	Stage II sporulation protein E (SpoIIE)	-0.213
PF01205	-	Uncharacterized protein family UPF0029	-0.216
PF03553	-	Na+/H+ antiporter family	-0.223
PF04313	-	Type I restriction enzyme R protein N terminus (HSDR_N)	-0.224
PF12161	-	HsdM N-terminal domain	-0.238
PF03432	-	Relaxase/Mobilisation nuclease domain	-0.269
PF04221	-	RelB antitoxin	-0.269
PF05193	-	Peptidase M16 inactive domain	-0.281
PF13495	-	Phage integrase, N-terminal SAM-like domain	-0.318
PF01268	-	Formate--tetrahydrofolate ligase	-0.348

Phenotype: myo-Inositol (Growth: Sugar), Predictor: phypat+PGL

Pfam	class	Pfam_desc	cor
PF09347	+	Domain of unknown function (DUF1989)	0.568
PF10005	+	Uncharacterized protein conserved in bacteria (DUF2248)	0.540
PF12098	+	Protein of unknown function (DUF3574)	0.540
PF06475	+	Putative glycolipid-binding	0.530
PF09349	+	OHCU decarboxylase	0.526
PF01958	+	Domain of unknown function DUF108	0.523
PF06267	+	Family of unknown function (DUF1028)	0.501
PF04982	+	HPP family	0.494
PF10081	+	Alpha/beta-hydrolase family	0.483
PF02909	+	Tetracyclin repressor, C-terminal all-alpha domain	0.462
PF04250	+	Protein of unknown function (DUF429)	0.438
PF05063	+	MT-A70	0.438
PF01011	+	PQQ enzyme repeat	0.420
PF11373	+	Protein of unknown function (DUF3175)	0.389
PF13444	+	Acetyltransferase (GNAT) domain	0.354
PF11755	+	Protein of unknown function (DUF3311)	0.344

PF07366	+	SnoaL-like polyketide cyclase	0.343
PF09821	+	C-terminal AAA-associated domain	0.340
PF05090	+	Vitamin K-dependent gamma-carboxylase	0.337
PF14226	+	non-haem dioxygenase in morphine synthesis N-terminal	0.335
PF08643	+	Fungal family of unknown function (DUF1776)	0.330
PF08818	+	Domain of unknown function (DU1801)	0.322
PF00295	+	Glycosyl hydrolases family 28	0.321
PF04268	+	Sarcosine oxidase, gamma subunit family	0.293
PF01341	+	Glycosyl hydrolases family 6	0.279
PF12680	-	SnoaL-like domain	0.269
PF01361	+	Tautomerase enzyme	0.236
PF04473	+	Transglutaminase-like domain	0.184
PF07978	-	NIPSNAP	0.124
PF01643	+	Acyl-ACP thioesterase	0.120
PF05598	+	Transposase domain (DUF772)	0.114
PF06559	+	2'-deoxycytidine 5'-triphosphate deaminase (DCD)	0.090
PF12019	-	Type II transport protein GspH	0.088
PF03799	-	Cell division protein FtsQ	0.076
PF08437	+	Glycosyl transferase family 8 C-terminal	0.016
PF01972	-	Serine dehydrogenase proteinase	0.001
PF13424	-	Tetratricopeptide repeat	-0.034
PF13413	-	Helix-turn-helix domain	-0.043
PF01814	-	Hemerythrin HHE cation binding domain	-0.055
PF04138	-	GtrA-like protein	-0.055
PF14559	-	Tetratricopeptide repeat	-0.057
PF02655	-	ATP-grasp domain	-0.068
PF08485	-	Polysaccharide biosynthesis protein C-terminal	-0.130
PF05219	-	DREV methyltransferase	-0.155
PF04011	-	LemA family	-0.180
PF07751	-	Abi-like protein	-0.183
PF02677	-	Uncharacterized BCR, COG1636	-0.193
PF00675	-	Insulinase (Peptidase family M16)	-0.197
PF05958	-	tRNA (Uracil-5-)-methyltransferase	-0.279
PF05193	-	Peptidase M16 inactive domain	-0.281

Phenotype: ONPG (beta galactosidase) (Growth: Sugar), Predictor: phypat

Pfam	class	Pfam_desc	cor
PF02929	+	Beta galactosidase small chain	0.781
PF00703	+	Glycosyl hydrolases family 2	0.704
PF02610	+	L-arabinose isomerase	0.666
PF11762	+	L-arabinose isomerase C-terminal domain	0.666
PF04616	+	Glycosyl hydrolases family 43	0.652
PF08532	+	Beta-galactosidase trimerisation domain	0.607
PF01055	+	Glycosyl hydrolases family 31	0.582
PF02449	+	Beta-galactosidase	0.573
PF07745	+	Glycosyl hydrolase family 53	0.555
PF00728	+	Glycosyl hydrolase family 20, catalytic domain	0.496
PF00150	+	Cellulase (glycosyl hydrolase family 5)	0.346

PF00754	+	F5/8 type C domain	0.313
PF05651	+	Putative sugar diacid recognition	0.308
PF07804	+	HipA-like C-terminal domain	0.297
PF02537	+	CrcB-like protein	0.287
PF10418	+	Iron-sulfur cluster binding domain of dihydroorotate dehydrogenase B	0.282
PF13248	+	zinc-ribbon domain	0.280
PF14526	+	Integron-associated effector binding protein	0.274
PF00667	+	FAD binding domain	0.259
PF09313	+	Domain of unknown function (DUF1971)	0.244
PF04991	+	LicD family	0.223
PF07660	+	Secretin and TonB N terminus short domain	0.172
PF04205	-	FMN-binding domain	0.164
PF03848	+	Tellurite resistance protein TehB	0.159
PF03649	-	Uncharacterised protein family (UPF0014)	0.145
PF04612	+	Type II secretion system (T2SS), protein M	0.139
PF08386	+	TAP-like protein	0.139
PF11398	-	Protein of unknown function (DUF2813)	0.119
PF11042	+	Protein of unknown function (DUF2750)	0.090
PF05598	+	Transposase domain (DUF772)	0.087
PF00350	-	Dynamin family	0.081
PF02580	-	D-Tyr-tRNA(Tyr) deacylase	0.061
PF03601	-	Conserved hypothetical protein 698	0.061
PF14552	+	Tautomerase enzyme	0.012
PF13091	-	PLD-like domain	-0.004
PF01243	-	Pyridoxamine 5'-phosphate oxidase	-0.007
PF03432	-	Relaxase/Mobilisation nuclease domain	-0.011
PF13491	-	Domain of unknown function (DUF4117)	-0.028
PF07996	-	Type IV secretion system proteins	-0.033
PF13594	-	Amidohydrolase	-0.042
PF01039	-	Carboxyl transferase domain	-0.042
PF02541	-	Ppx/GppA phosphatase family	-0.055
PF01068	+	ATP dependent DNA ligase domain	-0.061
PF12974	-	ABC transporter, phosphonate, periplasmic substrate-binding protein	-0.065
PF00111	-	2Fe-2S iron-sulfur cluster binding domain	-0.066
PF01226	-	Formate/nitrite transporter	-0.073
PF08028	-	Acyl-CoA dehydrogenase, C-terminal domain	-0.081
PF02589	-	Uncharacterised ACR, YkgG family COG1556	-0.082
PF04471	-	Restriction endonuclease	-0.084
PF09669	-	Phage regulatory protein Rha (Phage_pRha)	-0.086
PF01613	-	Flavin reductase like domain	-0.089
PF12706	-	Beta-lactamase superfamily domain	-0.092
PF13692	-	Glycosyl transferases group 1	-0.092
PF13359	-	DDE superfamily endonuclease	-0.100
PF06969	-	HemN C-terminal domain	-0.108
PF00595	-	PDZ domain (Also known as DHR or GLGF)	-0.131
PF00692	-	dUTPase	-0.132
PF14657	-	AP2-like DNA-binding integrase domain	-0.135
PF03352	-	Methyladenine glycosylase	-0.139

PF04226	-	Transglycosylase associated protein	-0.144
PF02277	-	Phosphoribosyltransferase	-0.147
PF01555	-	DNA methylase	-0.154
PF07669	-	Eco57I restriction-modification methylase	-0.160
PF01420	-	Type I restriction modification DNA specificity domain	-0.163
PF04233	-	Phage Mu protein F like protein	-0.173
PF07508	-	Recombinase	-0.191
PF04466	-	Phage terminase large subunit	-0.195
PF00732	-	GMC oxidoreductase	-0.200
PF13274	-	Protein of unknown function (DUF4065)	-0.206
PF03061	-	Thioesterase superfamily	-0.231
PF02515	-	CoA-transferase family III	-0.241
PF01135	-	Protein-L-isoaspartate(D-aspartate) O-methyltransferase (PCMT)	-0.243
PF02632	-	BioY family	-0.245
PF08460	-	Bacterial SH3 domain	-0.261
PF12799	-	Leucine Rich repeats (2 copies)	-0.267
PF04140	-	Isoprenylcysteine carboxyl methyltransferase (ICMT) family	-0.273
PF13336	-	Acetyl-CoA hydrolase/transferase C-terminal domain	-0.277
PF02550	-	Acetyl-CoA hydrolase/transferase N-terminal domain	-0.317
PF02637	-	GatB domain	-0.422
PF02934	-	GatB/GatE catalytic domain	-0.422

Phenotype: ONPG (beta galactosidase) (Growth: Sugar), Predictor: phypat+PGL

Pfam	class	Pfam_desc	cor
PF02929	+	Beta galactosidase small chain	0.781
PF00703	+	Glycosyl hydrolases family 2	0.704
PF02836	+	Glycosyl hydrolases family 2, TIM barrel domain	0.674
PF02610	+	L-arabinose isomerase	0.666
PF02837	+	Glycosyl hydrolases family 2, sugar binding domain	0.659
PF04616	+	Glycosyl hydrolases family 43	0.652
PF08532	+	Beta-galactosidase trimerisation domain	0.607
PF10509	+	Galactokinase galactose-binding signature	0.594
PF01055	+	Glycosyl hydrolases family 31	0.582
PF02449	+	Beta-galactosidase	0.573
PF08533	+	Beta-galactosidase C-terminal domain	0.543
PF01915	+	Glycosyl hydrolase family 3 C-terminal domain	0.529
PF04204	+	Homoserine O-succinyltransferase	0.510
PF00728	+	Glycosyl hydrolase family 20, catalytic domain	0.496
PF00011	+	Hsp20/alpha crystallin family	0.403
PF00596	+	Class II Aldolase and Adducin N-terminal domain	0.349
PF12821	-	Protein of unknown function (DUF3815)	0.311
PF01583	+	Adenylylsulphate kinase	0.306
PF10418	+	Iron-sulfur cluster binding domain of dihydroorotate dehydrogenase B	0.282
PF11941	+	Domain of unknown function (DUF3459)	0.280
PF14526	+	Integron-associated effector binding protein	0.274
PF06476	+	Protein of unknown function (DUF1090)	0.244
PF10688	-	Bacterial inner membrane protein	0.244
PF13192	+	Thioredoxin domain	0.241

PF01914	-	MarC family integral membrane protein	0.241
PF08240	+	Alcohol dehydrogenase GroES-like domain	0.230
PF03881	-	Fructosamine kinase	0.227
PF06296	+	Protein of unknown function (DUF1044)	0.218
PF09364	+	XFP N-terminal domain	0.213
PF02153	+	Prephenate dehydrogenase	0.213
PF03825	-	Nucleoside H+ symporter	0.206
PF13807	-	G-rich domain on putative tyrosine kinase	0.203
PF03190	+	Protein of unknown function, DUF255	0.199
PF05168	+	HEPN domain	0.195
PF11694	+	Protein of unknown function (DUF3290)	0.193
PF13727	+	CoA-binding domain	0.188
PF12790	+	Type VI secretion lipoprotein	0.186
PF02638	-	Glycosyl hydrolase like GH101	0.178
PF13391	+	HNH endonuclease	0.173
PF02694	+	Uncharacterised BCR, YnfA/UPF0060 family	0.172
PF09445	-	RNA cap guanine-N2 methyltransferase	0.164
PF13635	+	Domain of unknown function (DUF4143)	0.163
PF06414	+	Zeta toxin	0.160
PF03848	+	Tellurite resistance protein TehB	0.159
PF04239	+	Protein of unknown function (DUF421)	0.152
PF03649	-	Uncharacterised protein family (UPF0014)	0.145
PF04612	+	Type II secretion system (T2SS), protein M	0.139
PF02424	-	ApbE family	0.137
PF13280	+	WYL domain	0.137
PF06528	+	Phage P2 GpE	0.134
PF13493	+	Domain of unknown function (DUF4118)	0.123
PF00025	+	ADP-ribosylation factor family	0.120
PF00069	+	Protein kinase domain	0.108
PF07963	+	Prokaryotic N-terminal methylation motif	0.096
PF10400	+	Virulence activator alpha C-term	0.096
PF01188	-	Mandelate racemase / muconate lactonizing enzyme, C-terminal domain	0.095
PF13714	+	Phosphoenolpyruvate phosphomutase	0.092
PF08239	-	Bacterial SH3 domain	0.092
PF12797	-	4Fe-4S binding domain	0.090
PF11042	+	Protein of unknown function (DUF2750)	0.090
PF03320	+	Bacterial fructose-1,6-bisphosphatase, glpX-encoded	0.088
PF01590	+	GAF domain	0.085
PF13350	+	Tyrosine phosphatase family	0.082
PF00022	-	Actin	0.082
PF03824	-	High-affinity nickel-transport protein	0.080
PF14253	+	Bacteriophage abortive infection AbiH	0.076
PF14531	+	Kinase-like	0.069
PF02580	-	D-Tyr-tRNA(Tyr) deacylase	0.061
PF02698	+	DUF218 domain	0.061
PF03601	-	Conserved hypothetical protein 698	0.061
PF01453	+	D-mannose binding lectin	0.052
PF05272	+	Virulence-associated protein E	0.043

PF06800	-	Sugar transport protein	0.043
PF03767	-	HAD superfamily, subfamily IIIB (Acid phosphatase)	0.043
PF05015	+	Plasmid maintenance system killer protein	0.043
PF12147	+	Putative methyltransferase	0.042
PF08218	-	Citrate lyase ligase C-terminal domain	0.039
PF01019	+	Gamma-glutamyltranspeptidase	0.037
PF12724	-	Flavodoxin domain	0.036
PF05708	-	Orthopoxvirus protein of unknown function (DUF830)	0.035
PF09924	-	Uncharacterized conserved protein (DUF2156)	0.033
PF08332	+	Calcium/calmodulin dependent protein kinase II Association	0.032
PF02350	-	UDP-N-acetylglucosamine 2-epimerase	0.030
PF13792	-	Sulfate transporter N-terminal domain with GLY motif	0.024
PF00916	-	Sulfate transporter family	0.024
PF02278	-	Polysaccharide lyase family 8, super-sandwich domain	0.007
PF13176	-	Tetratricopeptide repeat	0.005
PF00239	-	Resolvase, N terminal domain	0.005
PF12146	-	Putative lysophospholipase	0.005
PF09997	-	Predicted membrane protein (DUF2238)	-0.000
PF03432	-	Relaxase/Mobilisation nuclease domain	-0.011
PF13936	-	Helix-turn-helix domain	-0.012
PF01642	-	Methylmalonyl-CoA mutase	-0.014
PF13181	-	Tetratricopeptide repeat	-0.014
PF11985	+	Protein of unknown function (DUF3486)	-0.018
PF02796	-	Helix-turn-helix domain of resolvase	-0.019
PF13395	-	HNH endonuclease	-0.022
PF13586	-	Transposase DDE domain	-0.024
PF12008	+	Type I restriction and modification enzyme - subunit R C terminal	-0.024
PF07201	-	HrpJ-like domain	-0.026
PF04888	-	Secretion system effector C (SseC) like family	-0.026
PF00080	-	Copper/zinc superoxide dismutase (SODC)	-0.028
PF02797	+	Chalcone and stilbene synthases, C-terminal domain	-0.033
PF00872	-	Transposase, Mutator family	-0.034
PF00975	-	Thioesterase domain	-0.036
PF14497	-	Glutathione S-transferase, C-terminal domain	-0.039
PF13474	+	SnoaL-like domain	-0.042
PF05191	-	Adenylate kinase, active site lid	-0.044
PF11760	-	Cobalamin synthesis G N-terminal	-0.045
PF02737	-	3-hydroxyacyl-CoA dehydrogenase, NAD binding domain	-0.047
PF06961	-	Protein of unknown function (DUF1294)	-0.050
PF01208	-	Uroporphyrinogen decarboxylase (URO-D)	-0.051
PF11870	-	Domain of unknown function (DUF3390)	-0.055
PF01226	-	Formate/nitrite transporter	-0.073
PF01890	-	Cobalamin synthesis G C-terminus	-0.075
PF13518	-	Helix-turn-helix domain	-0.082
PF04471	-	Restriction endonuclease	-0.084
PF01244	-	Membrane dipeptidase (Peptidase family M19)	-0.085
PF02677	-	Uncharacterized BCR, COG1636	-0.087
PF13601	-	Winged helix DNA-binding domain	-0.088

PF03450	-	CO dehydrogenase flavoprotein C-terminal domain	-0.096
PF04183	-	IucA / IucC family	-0.100
PF12363	-	Phage protein	-0.101
PF02608	-	Basic membrane protein	-0.104
PF06969	-	HemN C-terminal domain	-0.108
PF02535	-	ZIP Zinc transporter	-0.112
PF02654	-	Cobalamin-5-phosphate synthase	-0.120
PF01610	-	Transposase	-0.120
PF01809	-	Haemolytic domain	-0.123
PF00850	+	Histone deacetylase domain	-0.127
PF00595	-	PDZ domain (Also known as DHR or GLGF)	-0.131
PF14657	-	AP2-like DNA-binding integrase domain	-0.135
PF01555	-	DNA methylase	-0.154
PF06114	-	Domain of unknown function (DUF955)	-0.160
PF01878	-	EVE domain	-0.163
PF00355	+	Rieske [2Fe-2S] domain	-0.167
PF06983	-	3-demethylubiquinone-9 3-methyltransferase	-0.178
PF10294	-	Putative methyltransferase	-0.180
PF07508	-	Recombinase	-0.191
PF00732	-	GMC oxidoreductase	-0.200
PF13274	-	Protein of unknown function (DUF4065)	-0.206
PF03444	-	Winged helix-turn-helix transcription repressor, HrcA DNA-binding	-0.209
PF00023	-	Ankyrin repeat	-0.218
PF06068	+	TIP49 C-terminus	-0.223
PF10503	-	Esterase PHB depolymerase	-0.234
PF10397	-	Adenylosuccinate lyase C-terminus	-0.240
PF03060	-	Nitronate monooxygenase	-0.243
PF01135	-	Protein-L-isoaspartate(D-aspartate) O-methyltransferase (PCMT)	-0.243
PF01478	-	Type IV leader peptidase family	-0.248
PF08460	-	Bacterial SH3 domain	-0.261
PF02550	-	Acetyl-CoA hydrolase/transferase N-terminal domain	-0.317
PF07521	-	RNA-metabolising metallo-beta-lactamase	-0.327

Phenotype: Raffinose (Growth: Sugar), Predictor: phypat

Pfam	class	Pfam_desc	cor
PF07745	+	Glycosyl hydrolase family 53	0.554
PF02065	+	Melibiase	0.547
PF01055	+	Glycosyl hydrolases family 31	0.539
PF08532	+	Beta-galactosidase trimerisation domain	0.536
PF02449	+	Beta-galactosidase	0.521
PF08244	+	Glycosyl hydrolases family 32 C terminal	0.520
PF07929	+	Plasmid pRiA4b ORF-3-like protein	0.473
PF00251	+	Glycosyl hydrolases family 32 N-terminal domain	0.453
PF03051	+	Peptidase C1-like family	0.435
PF01223	+	DNA/RNA non-specific endonuclease	0.393
PF06202	+	Amylo-alpha-1,6-glucosidase	0.388
PF14310	+	Fibronectin type III-like domain	0.388
PF04204	+	Homoserine O-succinyltransferase	0.375

PF05870	+	Phenolic acid decarboxylase (PAD)	0.323
PF05402	+	Coenzyme PQQ synthesis protein D (PqqD)	0.287
PF09849	+	Uncharacterized protein conserved in bacteria (DUF2076)	0.287
PF13248	+	zinc-ribbon domain	0.286
PF03306	+	Alpha-acetolactate decarboxylase	0.277
PF04230	+	Polysaccharide pyruvyl transferase	0.259
PF06993	+	Protein of unknown function (DUF1304)	0.242
PF13723	+	Beta-ketoacyl synthase, N-terminal domain	0.234
PF01174	+	SNO glutamine amidotransferase family	0.223
PF05971	+	Protein of unknown function (DUF890)	0.212
PF01661	+	Macro domain	0.196
PF03070	+	TENA/THI-4/PQQC family	0.195
PF03837	+	RecT family	0.184
PF00754	-	F5/8 type C domain	0.184
PF13565	+	Homeodomain-like domain	0.181
PF07582	+	AP endonuclease family 2 C terminus	0.175
PF11700	+	Vacuole effluxer Atg22 like	0.153
PF14534	+	Domain of unknown function (DUF4440)	0.135
PF04261	+	Dyp-type peroxidase family	0.133
PF00576	+	HIUase/Transthyretin family	0.115
PF13599	+	Pentapeptide repeats (9 copies)	0.112
PF11367	+	Protein of unknown function (DUF3168)	0.093
PF02574	+	Homocysteine S-methyltransferase	0.087
PF00805	+	Pentapeptide repeats (8 copies)	0.085
PF10417	-	C-terminal domain of 1-Cys peroxiredoxin	0.068
PF03235	-	Protein of unknown function DUF262	0.046
PF07969	-	Amidohydrolase family	0.038
PF07510	-	Protein of unknown function (DUF1524)	0.011
PF03432	-	Relaxase/Mobilisation nuclease domain	0.003
PF04221	-	RelB antitoxin	-0.002
PF07751	-	Abi-like protein	-0.005
PF13545	-	Crp-like helix-turn-helix domain	-0.027
PF13114	-	RecO N terminal	-0.035
PF01663	-	Type I phosphodiesterase / nucleotide pyrophosphatase	-0.060
PF07685	-	CobB/CobQ-like glutamine amidotransferase domain	-0.069
PF13305	-	WHG domain	-0.075
PF00570	-	HRDC domain	-0.093
PF13379	-	NMT1-like family	-0.094
PF08937	-	MTH538 TIR-like domain (DUF1863)	-0.101
PF12161	-	HsdM N-terminal domain	-0.112
PF13191	-	AAA ATPase domain	-0.113
PF09397	-	Ftsk gamma domain	-0.118
PF09084	-	NMT1/THI5 like	-0.125
PF02698	-	DUF218 domain	-0.135
PF03400	-	IS1 transposase	-0.146
PF08352	-	Oligopeptide/dipeptide transporter, C-terminal region	-0.149
PF08003	-	Protein of unknown function (DUF1698)	-0.150
PF05913	-	Bacterial protein of unknown function (DUF871)	-0.152

PF00462	-	Glutaredoxin	-0.153
PF01555	-	DNA methylase	-0.156
PF13370	-	4Fe-4S single cluster domain	-0.161
PF02686	-	Glu-tRNAGln amidotransferase C subunit	-0.174
PF03102	-	NeuB family	-0.175
PF02934	-	GatB/GatE catalytic domain	-0.184
PF04079	-	Putative transcriptional regulators (Ypuh-like)	-0.185
PF03413	-	Peptidase propeptide and YPEB domain	-0.190
PF13231	-	Dolichyl-phosphate-mannose-protein mannosyltransferase	-0.192
PF02502	-	Ribose/Galactose Isomerase	-0.203
PF11230	-	Protein of unknown function (DUF3029)	-0.206
PF13857	-	Ankyrin repeats (many copies)	-0.213
PF03572	-	Peptidase family S41	-0.224
PF04264	-	YceI-like domain	-0.232
PF00188	-	Cysteine-rich secretory protein family	-0.234
PF00108	-	Thiolase, N-terminal domain	-0.234
PF01012	-	Electron transfer flavoprotein domain	-0.243
PF03618	-	Kinase/pyrophosphorylase	-0.244
PF07521	-	RNA-metabolising metallo-beta-lactamase	-0.259
PF07729	-	FCD domain	-0.260
PF02803	-	Thiolase, C-terminal domain	-0.268
PF00496	-	Bacterial extracellular solute-binding proteins, family 5 Middle	-0.283
PF00355	-	Rieske [2Fe-2S] domain	-0.354
PF00883	-	Cytosol aminopeptidase family, catalytic domain	-0.413

Phenotype: Raffinose (Growth: Sugar), Predictor: phypat+PGL

Pfam	class	Pfam_desc	cor
PF07745	+	Glycosyl hydrolase family 53	0.554
PF02065	+	Melibiase	0.547
PF01055	+	Glycosyl hydrolases family 31	0.539
PF08532	+	Beta-galactosidase trimerisation domain	0.536
PF08244	+	Glycosyl hydrolases family 32 C terminal	0.520
PF03051	+	Peptidase C1-like family	0.435
PF14897	+	EpsG family	0.418
PF01223	+	DNA/RNA non-specific endonuclease	0.393
PF04204	+	Homoserine O-succinyltransferase	0.375
PF11175	+	Protein of unknown function (DUF2961)	0.358
PF03629	+	Domain of unknown function (DUF303)	0.331
PF05870	+	Phenolic acid decarboxylase (PAD)	0.323
PF05402	+	Coenzyme PQQ synthesis protein D (PqqD)	0.287
PF03825	+	Nucleoside H+ symporter	0.285
PF06458	+	MucBP domain	0.273
PF11240	+	Protein of unknown function (DUF3042)	0.265
PF00520	+	Ion transport protein	0.260
PF06993	+	Protein of unknown function (DUF1304)	0.242
PF00781	-	Diacylglycerol kinase catalytic domain	0.236
PF04991	+	LicD family	0.234
PF13320	+	Domain of unknown function (DUF4091)	0.233

PF03162	+	Tyrosine phosphatase family	0.233
PF13811	+	Domain of unknown function (DUF4186)	0.229
PF01174	+	SNO glutamine amidotransferase family	0.223
PF04630	+	Phage major tail protein	0.212
PF07358	+	Protein of unknown function (DUF1482)	0.211
PF11694	+	Protein of unknown function (DUF3290)	0.201
PF14253	+	Bacteriophage abortive infection AbiH	0.196
PF03070	+	TENA/THI-4/PQQC family	0.195
PF11622	+	Protein of unknown function (DUF3251)	0.190
PF04170	+	NlpE N-terminal domain	0.178
PF02899	+	Phage integrase, N-terminal SAM-like domain	0.171
PF07302	+	AroM protein	0.171
PF13556	-	PucR C-terminal helix-turn-helix domain	0.168
PF10091	+	Putative glucoamylase	0.167
PF07555	+	beta-N-acetylglucosaminidase	0.160
PF09681	+	N-terminal phage replisome organiser (Phage_rep_org_N)	0.160
PF14501	-	GHKL domain	0.159
PF04262	+	Glutamate-cysteine ligase	0.157
PF03288	+	Poxvirus D5 protein-like	0.156
PF12459	+	D-Ala-teichoic acid biosynthesis protein	0.149
PF14845	+	beta-acetyl hexosaminidase like	0.145
PF01814	+	Hemerythrin HHE cation binding domain	0.138
PF13727	+	CoA-binding domain	0.138
PF03721	+	UDP-glucose/GDP-mannose dehydrogenase family, NAD binding domain	0.137
PF03649	-	Uncharacterised protein family (UPF0014)	0.134
PF06580	-	Histidine kinase	0.132
PF03613	-	PTS system mannose/fructose/sorbose family IID component	0.129
PF00078	-	Reverse transcriptase (RNA-dependent DNA polymerase)	0.125
PF13181	+	Tetratricopeptide repeat	0.125
PF00756	-	Putative esterase	0.121
PF07885	+	Ion channel	0.119
PF00195	+	Chalcone and stilbene synthases, N-terminal domain	0.118
PF09643	+	YopX protein	0.115
PF04464	-	CDP-Glycerol:Poly(glycerophosphate) glycerophosphotransferase	0.115
PF13480	-	Acetyltransferase (GNAT) domain	0.112
PF00988	-	Carbamoyl-phosphate synthase small chain, CPSase domain	0.102
PF00232	-	Glycosyl hydrolase family 1	0.096
PF13434	+	L-lysine 6-monooxygenase (NADPH-requiring)	0.091
PF10127	-	Predicted nucleotidyltransferase	0.081
PF00092	-	von Willebrand factor type A domain	0.074
PF08984	+	Domain of unknown function (DUF1858)	0.070
PF10417	-	C-terminal domain of 1-Cys peroxiredoxin	0.068
PF13380	-	CoA binding domain	0.066
PF13899	+	Thioredoxin-like	0.058
PF09669	-	Phage regulatory protein Rha (Phage_pRha)	0.057
PF08212	-	Lipocalin-like domain	0.051
PF06769	+	Plasmid encoded toxin Txe	0.045
PF13280	-	WYL domain	0.044

PF01593	+	Flavin containing amine oxidoreductase	0.041
PF12727	-	PBP superfamily domain	0.040
PF03382	+	Mycoplasma protein of unknown function, DUF285	0.040
PF02255	-	PTS system, Lactose/Cellobiose specific IIA subunit	0.039
PF04820	+	Tryptophan halogenase	0.038
PF02016	-	LD-carboxypeptidase	0.035
PF08448	+	PAS fold	0.033
PF03013	+	Pyrimidine dimer DNA glycosylase	0.031
PF14399	+	NlpC/p60-like transpeptidase	0.019
PF11638	+	DnaA N-terminal domain	0.014
PF10933	+	Protein of unknown function (DUF2827)	0.013
PF06912	-	Protein of unknown function (DUF1275)	0.011
PF13345	-	Domain of unknown function (DUF4098)	0.010
PF03237	-	Terminase-like family	0.008
PF13193	+	AMP-binding enzyme C-terminal domain	0.008
PF03829	-	PTS system glucitol/sorbitol-specific IIA component	0.004
PF14864	-	Alkyl sulfatase C-terminal	0.003
PF02056	-	Family 4 glycosyl hydrolase	0.001
PF11975	-	Family 4 glycosyl hydrolase C-terminal domain	0.001
PF04131	-	Putative N-acetylmannosamine-6-phosphate epimerase	0.001
PF04932	-	O-Antigen ligase	-0.002
PF11734	+	TilS substrate C-terminal domain	-0.004
PF13424	-	Tetratricopeptide repeat	-0.008
PF00239	-	Resolvase, N terminal domain	-0.014
PF02965	+	Vitamin B12 dependent methionine synthase, activation domain	-0.016
PF07943	+	Penicillin-binding protein 5, C-terminal domain	-0.019
PF13768	-	von Willebrand factor type A domain	-0.024
PF11319	+	Protein of unknown function (DUF3121)	-0.027
PF04199	+	Putative cyclase	-0.027
PF01470	-	Pyroglutamyl peptidase	-0.030
PF08535	-	KorB domain	-0.031
PF06953	-	Arsenical resistance operon trans-acting repressor ArsD	-0.031
PF01139	-	tRNA-splicing ligase RtcB	-0.033
PF02388	-	FemAB family	-0.038
PF11066	-	Protein of unknown function (DUF2867)	-0.039
PF01420	-	Type I restriction modification DNA specificity domain	-0.043
PF07228	-	Stage II sporulation protein E (SpoIIE)	-0.044
PF03330	+	Rare lipoprotein A (RlpA)-like double-psi beta-barrel	-0.048
PF07007	-	Protein of unknown function (DUF1311)	-0.050
PF12147	+	Putative methyltransferase	-0.051
PF02696	-	Uncharacterized ACR, YdiU/UPF0061 family	-0.052
PF09684	-	Phage tail protein (Tail_P2_I)	-0.058
PF03994	-	Domain of Unknown Function (DUF350)	-0.058
PF07484	-	Phage Tail Collar Domain	-0.059
PF13303	+	Phosphotransferase system, EIIC	-0.068
PF00923	-	Transaldolase	-0.069
PF02302	-	PTS system, Lactose/Cellobiose specific IIB subunit	-0.072
PF03831	-	PhnA protein	-0.078

PF13840	+	ACT domain	-0.081
PF00782	+	Dual specificity phosphatase, catalytic domain	-0.085
PF14526	-	Integron-associated effector binding protein	-0.087
PF11356	-	Type IV pilus biogenesis	-0.092
PF00570	-	HRDC domain	-0.093
PF12911	-	N-terminal TM domain of oligopeptide transport permease C	-0.093
PF06048	-	Domain of unknown function (DUF927)	-0.096
PF07731	-	Multicopper oxidase	-0.104
PF01946	+	Thi4 family	-0.104
PF12729	-	Four helix bundle sensory module for signal transduction	-0.108
PF13408	-	Recombinase zinc beta ribbon domain	-0.108
PF12161	-	HsdM N-terminal domain	-0.112
PF13191	-	AAA ATPase domain	-0.113
PF04134	-	Protein of unknown function, DUF393	-0.113
PF09084	-	NMT1/THI5 like	-0.125
PF00313	-	'Cold-shock' DNA-binding domain	-0.127
PF01740	-	STAS domain	-0.129
PF07508	-	Recombinase	-0.132
PF09299	-	Mu transposase, C-terminal	-0.146
PF13813	-	Membrane bound O-acyl transferase family	-0.146
PF05913	-	Bacterial protein of unknown function (DUF871)	-0.152
PF01555	-	DNA methylase	-0.156
PF07559	-	Flagellar basal body protein FlaE	-0.175
PF02814	-	UreE urease accessory protein, N-terminal domain	-0.181
PF00023	-	Ankyrin repeat	-0.190
PF04264	-	YceI-like domain	-0.232
PF00188	-	Cysteine-rich secretory protein family	-0.234
PF03618	-	Kinase/pyrophosphorylase	-0.244
PF13362	-	Toprim domain	-0.248
PF00355	-	Rieske [2Fe-2S] domain	-0.354

Phenotype: Salicin (Growth: Sugar), Predictor: phypat

Pfam	class	Pfam_desc	cor
PF03123	+	CAT RNA binding domain	0.585
PF07997	+	Protein of unknown function (DUF1694)	0.537
PF00232	+	Glycosyl hydrolase family 1	0.509
PF00251	+	Glycosyl hydrolases family 32 N-terminal domain	0.506
PF05043	+	Mga helix-turn-helix domain	0.505
PF02449	+	Beta-galactosidase	0.471
PF03830	+	PTS system sorbose subfamily IIB component	0.466
PF02903	+	Alpha amylase, N-terminal ig-like domain	0.466
PF03390	+	2-hydroxycarboxylate transporter family	0.442
PF00367	+	phosphotransferase system, EIIB	0.412
PF07745	+	Glycosyl hydrolase family 53	0.403
PF04616	+	Glycosyl hydrolases family 43	0.403
PF13930	+	DNA/RNA non-specific endonuclease	0.387
PF05870	+	Phenolic acid decarboxylase (PAD)	0.380
PF05592	+	Bacterial alpha-L-rhamnosidase	0.379

PF01087	+	Galactose-1-phosphate uridyl transferase, N-terminal domain	0.371
PF04239	+	Protein of unknown function (DUF421)	0.346
PF01238	+	Phosphomannose isomerase type I	0.344
PF03714	+	Bacterial pullanase-associated domain	0.329
PF01425	+	Amidase	0.318
PF07714	+	Protein tyrosine kinase	0.318
PF13345	+	Domain of unknown function (DUF4098)	0.315
PF13239	+	2TM domain	0.311
PF07470	+	Glycosyl Hydrolase Family 88	0.297
PF13347	+	MFS/sugar transport protein	0.278
PF02522	+	Aminoglycoside 3-N-acetyltransferase	0.278
PF01883	+	Domain of unknown function DUF59	0.275
PF13395	+	HNH endonuclease	0.269
PF02074	+	Carboxypeptidase Taq (M32) metallopeptidase	0.265
PF00756	+	Putative esterase	0.262
PF12996	+	DUF based on E. rectale Gene description (DUF3880)	0.248
PF03412	+	Peptidase C39 family	0.248
PF00295	+	Glycosyl hydrolases family 28	0.245
PF12486	+	ImpA domain protein	0.243
PF00311	+	Phosphoenolpyruvate carboxylase	0.233
PF02230	+	Phospholipase/Carboxylesterase	0.228
PF09084	+	NMT1/THI5 like	0.220
PF08774	+	VRR-NUC domain	0.219
PF01679	+	Proteolipid membrane potential modulator	0.205
PF01638	+	HxlR-like helix-turn-helix	0.204
PF08000	+	Bacterial PH domain	0.176
PF12822	-	Protein of unknown function (DUF3816)	0.171
PF01306	+	LacY proton/sugar symporter	0.168
PF13343	+	Bacterial extracellular solute-binding protein	0.163
PF06166	-	Protein of unknown function (DUF979)	0.147
PF09339	+	IclR helix-turn-helix domain	0.122
PF00977	+	Histidine biosynthesis protein	0.121
PF01522	+	Polysaccharide deacetylase	0.113
PF04235	+	Protein of unknown function (DUF418)	0.109
PF12867	+	DinB superfamily	0.100
PF00182	+	Chitinase class I	0.099
PF09827	+	CRISPR associated protein Cas2	0.094
PF13704	+	Glycosyl transferase family 2	0.091
PF04466	+	Phage terminase large subunit	0.090
PF01596	+	O-methyltransferase	0.079
PF00487	+	Fatty acid desaturase	0.072
PF13751	-	Transposase DDE domain	0.062
PF02371	-	Transposase IS116/IS110/IS902 family	0.061
PF13542	-	Helix-turn-helix domain of transposase family ISL3	0.059
PF07554	-	Uncharacterised Sugar-binding Domain	0.055
PF13384	-	Homeodomain-like domain	0.052
PF13599	+	Pentapeptide repeats (9 copies)	0.047
PF05866	-	Endodeoxyribonuclease RusA	0.042

PF06609	+	Fungal trichothecene efflux pump (TRI12)	0.041
PF10503	+	Esterase PHB depolymerase	0.028
PF04198	-	Putative sugar-binding domain	0.024
PF01050	+	Mannose-6-phosphate isomerase	0.011
PF03881	-	Fructosamine kinase	0.006
PF05191	-	Adenylate kinase, active site lid	-0.018
PF02384	-	N-6 DNA Methylase	-0.026
PF03681	-	Uncharacterised protein family (UPF0150)	-0.026
PF01633	-	Choline/ethanolamine kinase	-0.029
PF14493	-	Helix-turn-helix domain	-0.046
PF03435	+	Saccharopine dehydrogenase	-0.056
PF01192	-	RNA polymerase Rpb6	-0.058
PF09512	-	Thiamine-precursor transporter protein (ThiW)	-0.062
PF03848	-	Tellurite resistance protein TehB	-0.071
PF06983	-	3-demethylubiquinone-9 3-methyltransferase	-0.074
PF13342	-	C-terminal repeat of topoisomerase	-0.080
PF07669	-	Eco57I restriction-modification methylase	-0.087
PF00665	-	Integrase core domain	-0.109
PF02308	-	MgtC family	-0.133
PF01555	-	DNA methylase	-0.134
PF00561	-	alpha/beta hydrolase fold	-0.155
PF06172	-	Cupin superfamily (DUF985)	-0.163
PF00239	-	Resolvase, N terminal domain	-0.171
PF13426	-	PAS domain	-0.179
PF03746	-	LamB/YcsF family	-0.182
PF10604	-	Polyketide cyclase / dehydrase and lipid transport	-0.188
PF02682	-	Allophanate hydrolase subunit 1	-0.192
PF13482	-	RNase_H superfamily	-0.192
PF13186	-	Iron-sulfur cluster-binding domain	-0.192
PF06889	-	Protein of unknown function (DUF1266)	-0.204
PF01865	-	Protein of unknown function DUF47	-0.212
PF05237	-	MoeZ/MoeB domain	-0.247
PF02515	-	CoA-transferase family III	-0.250
PF00725	-	3-hydroxyacyl-CoA dehydrogenase, C-terminal domain	-0.271
PF14693	-	Ribosomal protein TL5, C-terminal domain	-0.275
PF04879	-	Molybdopterin oxidoreductase Fe4S4 domain	-0.285
PF02492	-	CobW/HypB/UreG, nucleotide-binding domain	-0.289
PF03916	-	Polysulphide reductase, NrfD	-0.296
PF02541	-	Ppx/GppA phosphatase family	-0.304
PF00885	-	6,7-dimethyl-8-ribityllumazine synthase	-0.312
PF00209	-	Sodium:neurotransmitter symporter family	-0.314
PF00677	-	Lumazine binding domain	-0.336
PF07549	-	SecD/SecF GG Motif	-0.353
PF00529	-	HlyD family secretion protein	-0.376
PF00795	-	Carbon-nitrogen hydrolase	-0.385
PF12801	-	4Fe-4S binding domain	-0.403
PF08028	-	Acyl-CoA dehydrogenase, C-terminal domain	-0.412
PF01012	-	Electron transfer flavoprotein domain	-0.447

Phenotype: Salicin (Growth: Sugar), Predictor: phypat+PGL

Pfam	class	Pfam_desc	cor
PF03123	+	CAT RNA binding domain	0.585
PF00232	+	Glycosyl hydrolase family 1	0.509
PF05043	+	Mga helix-turn-helix domain	0.505
PF08951	+	Enterocin A Immunity	0.494
PF00874	+	PRD domain	0.477
PF02449	+	Beta-galactosidase	0.471
PF06161	+	Protein of unknown function (DUF975)	0.469
PF02903	+	Alpha amylase, N-terminal ig-like domain	0.466
PF03306	+	Alpha-acetolactate decarboxylase	0.447
PF03390	+	2-hydroxycarboxylate transporter family	0.442
PF07745	+	Glycosyl hydrolase family 53	0.403
PF04616	+	Glycosyl hydrolases family 43	0.403
PF12535	+	Hydrolase of X-linked nucleoside diphosphate N terminal	0.370
PF02486	+	Replication initiation factor	0.347
PF01238	+	Phosphomannose isomerase type I	0.344
PF10140	+	WXG100 protein secretion system (Wss), protein YukC	0.339
PF09913	+	Predicted membrane protein (DUF2142)	0.332
PF03714	+	Bacterial pullanase-associated domain	0.329
PF06800	+	Sugar transport protein	0.328
PF13345	+	Domain of unknown function (DUF4098)	0.315
PF13239	+	2TM domain	0.311
PF07748	+	Glycosyl hydrolases family 38 C-terminal domain	0.297
PF02065	+	Melibiase	0.291
PF00413	-	Matrixin	0.281
PF08530	-	X-Pro dipeptidyl-peptidase C-terminal non-catalytic domain	0.278
PF01883	+	Domain of unknown function DUF59	0.275
PF13395	+	HNH endonuclease	0.269
PF02608	+	Basic membrane protein	0.258
PF00295	+	Glycosyl hydrolases family 28	0.245
PF09849	+	Uncharacterized protein conserved in bacteria (DUF2076)	0.243
PF13672	-	Protein phosphatase 2C	0.241
PF00359	+	Phosphoenolpyruvate-dependent sugar phosphotransferase system, EIIA 2	0.220
PF08774	+	VRR-NUC domain	0.219
PF12538	+	DNA transporter	0.211
PF05426	+	Alginate lyase	0.210
PF08439	+	Oligopeptidase F	0.206
PF07885	+	Ion channel	0.201
PF02733	+	Dak1 domain	0.198
PF09643	+	YopX protein	0.197
PF02674	+	Colicin V production protein	0.193
PF05133	+	Phage portal protein, SPP1 Gp6-like	0.192
PF14470	+	Bacterial PH domain	0.191
PF05270	+	Alpha-L-arabinofuranosidase B (ABFB)	0.185
PF08000	+	Bacterial PH domain	0.176
PF04282	-	Family of unknown function (DUF438)	0.170

PF01306	+	LacY proton/sugar symporter	0.168
PF12368	-	Protein of unknown function (DUF3650)	0.159
PF02129	-	X-Pro dipeptidyl-peptidase (S15 family)	0.158
PF05257	-	CHAP domain	0.157
PF11611	+	Domain of unknown function (DUF4352)	0.154
PF06114	-	Domain of unknown function (DUF955)	0.149
PF12708	+	Pectate lyase superfamily protein	0.148
PF12666	-	PrgI family protein	0.148
PF06149	-	Protein of unknown function (DUF969)	0.147
PF06166	-	Protein of unknown function (DUF979)	0.147
PF13156	+	Restriction endonuclease	0.145
PF01487	-	Type I 3-dehydroquinase	0.145
PF02872	+	5'-nucleotidase, C-terminal domain	0.137
PF14845	+	beta-acetyl hexosaminidase like	0.135
PF02817	+	e3 binding domain	0.130
PF00161	+	Ribosome inactivating protein	0.118
PF01522	+	Polysaccharide deacetylase	0.113
PF07905	+	Purine catabolism regulatory protein-like family	0.112
PF00296	-	Luciferase-like monooxygenase	0.109
PF02388	-	FemAB family	0.109
PF09709	+	CRISPR-associated protein (Cas_Csd1)	0.105
PF01094	+	Receptor family ligand binding region	0.103
PF05656	+	Protein of unknown function (DUF805)	0.101
PF05402	+	Coenzyme PQQ synthesis protein D (PqqD)	0.097
PF10397	-	Adenylosuccinate lyase C-terminus	0.095
PF09827	+	CRISPR associated protein Cas2	0.094
PF00195	+	Chalcone and stilbene synthases, N-terminal domain	0.094
PF13704	+	Glycosyl transferase family 2	0.091
PF13936	-	Helix-turn-helix domain	0.086
PF13587	+	N-terminal domain of DJ-1_PfpI family	0.085
PF05065	-	Phage capsid family	0.076
PF13749	+	ATP-dependent DNA helicase recG C-terminal	0.075
PF04794	+	YdjC-like protein	0.074
PF01527	-	Transposase	0.073
PF01614	+	Bacterial transcriptional regulator	0.071
PF08386	+	TAP-like protein	0.071
PF02371	-	Transposase IS116/IS110/IS902 family	0.061
PF12229	-	Putative peptidoglycan binding domain	0.061
PF12897	+	Alanine-glyoxylate amino-transferase	0.056
PF06204	+	Putative carbohydrate binding domain	0.055
PF07554	-	Uncharacterised Sugar-binding Domain	0.055
PF13370	-	4Fe-4S single cluster domain	0.049
PF00268	-	Ribonucleotide reductase, small chain	0.038
PF13567	-	Domain of unknown function (DUF4131)	0.029
PF13392	-	HNH endonuclease	0.028
PF02447	+	GntP family permease	0.027
PF13088	-	BNR repeat-like domain	0.025
PF09234	+	Domain of unknown function (DUF1963)	0.023

PF13514	-	AAA domain	0.016
PF02110	-	Hydroxyethylthiazole kinase family	0.014
PF13612	-	Transposase DDE domain	0.013
PF13539	+	D-alanyl-D-alanine carboxypeptidase	0.012
PF08450	+	SMP-30/Gluconolaconase/LRE-like region	0.008
PF01593	+	Flavin containing amine oxidoreductase	0.002
PF05713	-	Bacterial mobilisation protein (MobC)	-0.004
PF12910	-	Antitoxin of toxin-antitoxin stability system N-terminal	-0.005
PF13240	-	zinc-ribbon domain	-0.005
PF07501	-	G5 domain	-0.008
PF08863	-	YolD-like protein	-0.010
PF04970	+	Lecithin retinol acyltransferase	-0.010
PF13229	+	Right handed beta helix region	-0.012
PF00317	-	Ribonucleotide reductase, all-alpha domain	-0.012
PF00872	-	Transposase, Mutator family	-0.013
PF00585	-	C-terminal regulatory domain of Threonine dehydratase	-0.019
PF12905	-	Endo-alpha-N-acetylgalactosaminidase	-0.022
PF03681	-	Uncharacterised protein family (UPF0150)	-0.026
PF01402	+	Ribbon-helix-helix protein, copG family	-0.026
PF01633	-	Choline/ethanolamine kinase	-0.029
PF06769	-	Plasmid encoded toxin Txe	-0.030
PF14344	-	Domain of unknown function (DUF4397)	-0.031
PF13518	-	Helix-turn-helix domain	-0.032
PF03994	-	Domain of Unknown Function (DUF350)	-0.032
PF13480	-	Acetyltransferase (GNAT) domain	-0.033
PF11148	-	Protein of unknown function (DUF2922)	-0.034
PF13385	-	Concanavalin A-like lectin/glucanases superfamily	-0.034
PF00975	-	Thioesterase domain	-0.039
PF13289	-	SIR2-like domain	-0.040
PF04257	-	Exodeoxyribonuclease V, gamma subunit	-0.040
PF13333	-	Integrase core domain	-0.044
PF05360	-	yiaA/B two helix domain	-0.045
PF13391	-	HNH endonuclease	-0.051
PF01764	-	Lipase (class 3)	-0.057
PF01192	-	RNA polymerase Rpb6	-0.058
PF12437	+	Glutamine synthetase type III N terminal	-0.058
PF13425	+	O-antigen ligase like membrane protein	-0.059
PF07729	-	FCD domain	-0.062
PF09512	-	Thiamine-precursor transporter protein (ThiW)	-0.062
PF05901	-	Excalibur calcium-binding domain	-0.062
PF03445	-	Putative nucleotidyltransferase DUF294	-0.063
PF03432	-	Relaxase/Mobilisation nuclease domain	-0.070
PF00903	-	Glyoxalase/Bleomycin resistance protein/Dioxygenase superfamily	-0.071
PF08378	-	Nuclease-related domain	-0.074
PF04993	-	TfoX N-terminal domain	-0.075
PF13342	-	C-terminal repeat of topoisomerase	-0.080
PF13173	-	AAA domain	-0.084
PF07411	-	Domain of unknown function (DUF1508)	-0.085

PF13166	+	AAA domain	-0.096
PF03788	-	LrgA family	-0.101
PF01292	+	Prokaryotic cytochrome b561	-0.103
PF00665	-	Integrase core domain	-0.109
PF01925	-	Sulfite exporter TauE/SafE	-0.110
PF04199	-	Putative cyclase	-0.110
PF05145	-	Putative ammonia monooxygenase	-0.110
PF00515	+	Tetratricopeptide repeat	-0.126
PF13084	+	Domain of unknown function (DUF3943)	-0.127
PF08761	-	dUTPase	-0.133
PF01555	-	DNA methylase	-0.134
PF02028	-	BCCT family transporter	-0.139
PF00033	+	Cytochrome b(N-terminal)/b6/petB	-0.142
PF01262	-	Alanine dehydrogenase/PNT, C-terminal domain	-0.157
PF00239	-	Resolvase, N terminal domain	-0.171
PF03100	-	CcmE	-0.176
PF13426	-	PAS domain	-0.179
PF06821	+	Serine hydrolase	-0.183
PF03379	-	CcmB protein	-0.185
PF05222	-	Alanine dehydrogenase/PNT, N-terminal domain	-0.191
PF13186	-	Iron-sulfur cluster-binding domain	-0.192
PF01494	-	FAD binding domain	-0.193
PF13813	-	Membrane bound O-acyl transferase family	-0.195
PF01175	-	Urocanase	-0.196
PF02628	-	Cytochrome oxidase assembly protein	-0.212
PF01865	-	Protein of unknown function DUF47	-0.212
PF13336	-	Acetyl-CoA hydrolase/transferase C-terminal domain	-0.230
PF03205	-	Molybdopterin guanine dinucleotide synthesis protein B	-0.236
PF13247	-	4Fe-4S dicluster domain	-0.237
PF01288	-	7,8-dihydro-6-hydroxymethylpterin-pyrophosphokinase (HPPK)	-0.241
PF13536	-	Multidrug resistance efflux transporter	-0.248
PF06463	-	Molybdenum Cofactor Synthesis C	-0.258
PF01967	-	MoaC family	-0.258
PF00725	+	3-hydroxyacyl-CoA dehydrogenase, C-terminal domain	-0.271
PF13624	+	SurA N-terminal domain	-0.275
PF02550	-	Acetyl-CoA hydrolase/transferase N-terminal domain	-0.301
PF00209	-	Sodium:neurotransmitter symporter family	-0.314
PF00795	-	Carbon-nitrogen hydrolase	-0.385

Phenotype: Starch hydrolysis (Growth: Sugar), Predictor: phypat

Pfam	class	Pfam_desc	cor
PF03714	+	Bacterial pullanase-associated domain	0.586
PF10997	+	Protein of unknown function (DUF2837)	0.511
PF09323	+	Domain of unknown function (DUF1980)	0.493
PF00041	+	Fibronectin type III domain	0.465
PF02903	+	Alpha amylase, N-terminal ig-like domain	0.400
PF14689	+	Sensor_kinase_SpoOB-type, alpha-helical domain	0.399
PF00686	+	Starch binding domain	0.394

PF12464	+	Maltose acetyltransferase	0.383
PF08244	+	Glycosyl hydrolases family 32 C terminal	0.380
PF00201	+	UDP-glucoronosyl and UDP-glucosyl transferase	0.379
PF02868	+	Thermolysin metallopeptidase, alpha-helical domain	0.379
PF01447	+	Thermolysin metallopeptidase, catalytic domain	0.379
PF07694	+	5TMR of 5TMR-LYT	0.366
PF06054	+	Competence protein CoiA-like family	0.352
PF04397	+	LytTr DNA-binding domain	0.351
PF06580	+	Histidine kinase	0.338
PF02838	+	Glycosyl hydrolase family 20, domain 2	0.332
PF06013	+	Proteins of 100 residues with WXG	0.324
PF14267	+	Domain of unknown function (DUF4357)	0.317
PF03496	+	ADP-ribosyltransferase exoenzyme	0.317
PF01869	+	BadF/BadG/BcrA/BcrD ATPase family	0.310
PF07784	+	Protein of unknown function (DUF1622)	0.309
PF08378	+	Nuclease-related domain	0.298
PF11127	+	Protein of unknown function (DUF2892)	0.285
PF07221	+	N-acylglucosamine 2-epimerase (GlcNAc 2-epimerase)	0.269
PF13408	+	Recombinase zinc beta ribbon domain	0.266
PF08327	+	Activator of Hsp90 ATPase homolog 1-like protein	0.232
PF07739	+	TipAS antibiotic-recognition domain	0.224
PF02870	+	6-O-methylguanine DNA methyltransferase, ribonuclease-like domain	0.210
PF03553	+	Na+/H+ antiporter family	0.167
PF00962	+	Adenosine/AMP deaminase	0.159
PF13346	-	ABC-2 family transporter protein	0.010
PF11734	-	TilS substrate C-terminal domain	-0.046
PF02110	-	Hydroxyethylthiazole kinase family	-0.075
PF05148	-	Hypothetical methyltransferase	-0.077
PF02350	-	UDP-N-acetylglucosamine 2-epimerase	-0.081
PF03389	-	MobA/MobL family	-0.093
PF13007	-	Transposase C of IS166 homeodomain	-0.096
PF09966	-	Uncharacterized protein conserved in bacteria (DUF2200)	-0.114
PF05402	-	Coenzyme PQQ synthesis protein D (PqqD)	-0.120
PF02659	-	Domain of unknown function DUF	-0.125
PF02580	-	D-Tyr-tRNA(Tyr) deacylase	-0.130
PF13348	-	Tyrosine phosphatase family C-terminal region	-0.139
PF02667	-	Short chain fatty acid transporter	-0.153
PF02498	-	BRO family, N-terminal domain	-0.161
PF01613	-	Flavin reductase like domain	-0.163
PF08903	-	Domain of unknown function (DUF1846)	-0.166
PF08867	-	FRG domain	-0.170
PF05866	-	Endodeoxyribonuclease RusA	-0.170
PF02920	-	DNA binding domain of tn916 integrase	-0.185
PF01938	-	TRAM domain	-0.190
PF01510	-	N-acetylmuramoyl-L-alanine amidase	-0.198
PF00925	-	GTP cyclohydrolase II	-0.202
PF00926	-	3,4-dihydroxy-2-butanone 4-phosphate synthase	-0.202
PF03253	-	Urea transporter	-0.220

PF03374	-	Phage antirepressor protein KilAC domain	-0.220
PF01402	-	Ribbon-helix-helix protein, copG family	-0.245
PF02348	-	Cytidylyltransferase	-0.282
PF02541	-	Ppx/GppA phosphatase family	-0.431

Phenotype: Starch hydrolysis (Growth: Sugar), Predictor: phypat+PGL

Pfam	class	Pfam_desc	cor
PF03714	+	Bacterial pullanase-associated domain	0.586
PF10997	+	Protein of unknown function (DUF2837)	0.511
PF09323	+	Domain of unknown function (DUF1980)	0.493
PF00041	+	Fibronectin type III domain	0.465
PF14689	+	Sensor_kinase_SpoOB-type, alpha-helical domain	0.399
PF02225	+	PA domain	0.385
PF12464	+	Maltose acetyltransferase	0.383
PF07694	+	5TMR of 5TMR-LYT	0.366
PF01712	+	Deoxynucleoside kinase	0.349
PF06580	+	Histidine kinase	0.338
PF04854	+	Protein of unknown function, DUF624	0.334
PF02838	+	Glycosyl hydrolase family 20, domain 2	0.332
PF02449	+	Beta-galactosidase	0.331
PF08532	+	Beta-galactosidase trimerisation domain	0.320
PF05651	+	Putative sugar diacid recognition	0.319
PF14267	+	Domain of unknown function (DUF4357)	0.317
PF03496	+	ADP-ribosyltransferase exoenzyme	0.317
PF02065	+	Melibiase	0.314
PF02156	+	Glycosyl hydrolase family 26	0.313
PF01869	+	BadF/BadG/BcrA/BcrD ATPase family	0.310
PF07784	+	Protein of unknown function (DUF1622)	0.309
PF13906	+	C-terminus of AA_permease	0.301
PF08378	+	Nuclease-related domain	0.298
PF06182	+	ABC-2 family transporter protein	0.284
PF02744	+	Galactose-1-phosphate uridyl transferase, C-terminal domain	0.283
PF07555	+	beta-N-acetylglucosaminidase	0.277
PF13592	+	Winged helix-turn helix	0.270
PF13156	+	Restriction endonuclease	0.270
PF08020	+	Protein of unknown function (DUF1706)	0.234
PF14355	+	Abortive infection C-terminus	0.232
PF01865	+	Protein of unknown function DUF47	0.232
PF00909	+	Ammonium Transporter Family	0.220
PF04266	+	ASCH domain	0.220
PF06202	+	Amylo-alpha-1,6-glucosidase	0.218
PF07905	+	Purine catabolism regulatory protein-like family	0.215
PF02229	+	Transcriptional Coactivator p15 (PC4)	0.208
PF07971	+	Glycosyl hydrolase family 92	0.208
PF01590	+	GAF domain	0.207
PF05598	+	Transposase domain (DUF772)	0.205
PF05709	+	Phage tail protein	0.202
PF14200	+	Ricin-type beta-trefoil lectin domain-like	0.190

PF06439	+	Domain of Unknown Function (DUF1080)	0.175
PF00962	+	Adenosine/AMP deaminase	0.159
PF07994	+	Myo-inositol-1-phosphate synthase	0.158
PF01658	+	Myo-inositol-1-phosphate synthase	0.158
PF04465	+	Protein of unknown function (DUF499)	0.150
PF11716	-	Mycothiol maleylpyruvate isomerase N-terminal domain	0.150
PF04993	+	TfoX N-terminal domain	0.124
PF01169	+	Uncharacterized protein family UPF0016	0.123
PF02674	+	Colicin V production protein	0.122
PF08308	+	PEGA domain	0.120
PF09848	+	Uncharacterized conserved protein (DUF2075)	0.112
PF10029	+	Predicted periplasmic protein (DUF2271)	0.105
PF09563	+	LlaJI restriction endonuclease	0.097
PF03704	+	Bacterial transcriptional activator domain	0.081
PF02666	+	Phosphatidylserine decarboxylase	0.077
PF07751	+	Abi-like protein	0.061
PF12687	+	Protein of unknown function (DUF3801)	0.059
PF02036	+	SCP-2 sterol transfer family	0.031
PF02391	-	MoaE protein	0.030
PF00403	+	Heavy-metal-associated domain	0.029
PF05954	+	Phage late control gene D protein (GPD)	0.010
PF13414	+	TPR repeat	0.009
PF01729	-	Quinolinate phosphoribosyl transferase, C-terminal domain	-0.004
PF00302	-	Chloramphenicol acetyltransferase	-0.007
PF00872	-	Transposase, Mutator family	-0.016
PF01243	-	Pyridoxamine 5'-phosphate oxidase	-0.019
PF02583	-	Metal-sensitive transcriptional repressor	-0.020
PF12708	-	Pectate lyase superfamily protein	-0.033
PF08774	+	VRR-NUC domain	-0.034
PF13672	-	Protein phosphatase 2C	-0.036
PF08343	-	Ribonucleotide reductase N-terminal	-0.036
PF03881	-	Fructosamine kinase	-0.041
PF03734	-	L,D-transpeptidase catalytic domain	-0.048
PF04221	-	RelB antitoxin	-0.050
PF01471	-	Putative peptidoglycan binding domain	-0.056
PF01262	-	Alanine dehydrogenase/PNT, C-terminal domain	-0.081
PF01040	-	UbiA prenyltransferase family	-0.082
PF12897	-	Alanine-glyoxylate amino-transferase	-0.085
PF05861	-	Bacterial phosphonate metabolism protein (PhnI)	-0.090
PF10551	-	MULE transposase domain	-0.090
PF03389	-	MobA/MobL family	-0.093
PF01614	-	Bacterial transcriptional regulator	-0.100
PF13524	-	Glycosyl transferases group 1	-0.103
PF05076	-	Suppressor of fused protein (SUFU)	-0.107
PF06559	-	2'-deoxycytidine 5'-triphosphate deaminase (DCD)	-0.111
PF06039	-	Malate:quinone oxidoreductase (Mqo)	-0.120
PF02659	-	Domain of unknown function DUF	-0.125
PF09339	-	IclR helix-turn-helix domain	-0.128

PF01774	-	UreD urease accessory protein	-0.129
PF13340	-	Putative transposase of IS4/5 family (DUF4096)	-0.139
PF13454	-	FAD-NAD(P)-binding	-0.153
PF10417	-	C-terminal domain of 1-Cys peroxiredoxin	-0.153
PF13362	-	Toprim domain	-0.153
PF13586	-	Transposase DDE domain	-0.153
PF09924	-	Uncharacterized conserved protein (DUF2156)	-0.157
PF01613	-	Flavin reductase like domain	-0.163
PF13591	-	MerR HTH family regulatory protein	-0.192
PF00202	-	Aminotransferase class-III	-0.224
PF05437	-	Branched-chain amino acid transport protein (AzlD)	-0.247
PF01988	-	VIT family	-0.270
PF12738	-	twin BRCT domain	-0.276
PF02541	-	Ppx/GppA phosphatase family	-0.431

Phenotype: Sucrose (Growth: Sugar), Predictor: phypat

Pfam	class	Pfam_desc	cor
PF00251	+	Glycosyl hydrolases family 32 N-terminal domain	0.731
PF08244	+	Glycosyl hydrolases family 32 C terminal	0.664
PF06993	+	Protein of unknown function (DUF1304)	0.400
PF08532	+	Beta-galactosidase trimerisation domain	0.379
PF14010	+	Phosphoenolpyruvate carboxylase	0.332
PF00195	+	Chalcone and stilbene synthases, N-terminal domain	0.318
PF03773	+	Predicted permease	0.296
PF00135	+	Carboxylesterase family	0.286
PF03390	+	2-hydroxycarboxylate transporter family	0.267
PF01928	+	CYTH domain	0.258
PF10127	+	Predicted nucleotidyltransferase	0.226
PF13558	+	Putative exonuclease SbcCD, C subunit	0.176
PF02237	+	Biotin protein ligase C terminal domain	0.157
PF13473	+	Cupredoxin-like domain	0.134
PF04343	+	Protein of unknown function, DUF488	0.112
PF10340	+	Protein of unknown function (DUF2424)	0.096
PF01909	+	Nucleotidyltransferase domain	0.074
PF12727	-	PBP superfamily domain	0.063
PF07885	-	Ion channel	0.005
PF01575	+	MaoC like domain	-0.014
PF13444	+	Acetyltransferase (GNAT) domain	-0.019
PF03444	-	Winged helix-turn-helix transcription repressor, HrcA DNA-binding	-0.056
PF02934	-	GatB/GatE catalytic domain	-0.095
PF02637	-	GatB domain	-0.095
PF01564	-	Spermine/spermidine synthase	-0.116
PF03432	-	Relaxase/Mobilisation nuclease domain	-0.148
PF01592	-	NifU-like N terminal domain	-0.161
PF02498	-	BRO family, N-terminal domain	-0.163
PF03618	-	Kinase/pyrophosphorylase	-0.180
PF13359	-	DDE superfamily endonuclease	-0.194
PF06969	-	HemN C-terminal domain	-0.213

PF02511	-	Thymidylate synthase complementing protein	-0.223
PF13905	-	Thioredoxin-like	-0.228
PF14497	-	Glutathione S-transferase, C-terminal domain	-0.233
PF02667	-	Short chain fatty acid transporter	-0.291
PF12738	-	twin BRCT domain	-0.291
PF04303	-	PrpF protein	-0.301
PF00848	-	Ring hydroxylating alpha subunit (catalytic domain)	-0.334
PF14532	-	Sigma-54 interaction domain	-0.358
PF02550	-	Acetyl-CoA hydrolase/transferase N-terminal domain	-0.365
PF12801	-	4Fe-4S binding domain	-0.367
PF03572	-	Peptidase family S41	-0.381
PF00883	-	Cytosol aminopeptidase family, catalytic domain	-0.384
PF01863	-	Protein of unknown function DUF45	-0.386
PF02075	-	Crossover junction endodeoxyribonuclease RuvC	-0.409
PF01012	-	Electron transfer flavoprotein domain	-0.410
PF03968	-	OstA-like protein	-0.454

Phenotype: Sucrose (Growth: Sugar), Predictor: phypat+PGL

Pfam	class	Pfam_desc	cor
PF00251	+	Glycosyl hydrolases family 32 N-terminal domain	0.731
PF08244	+	Glycosyl hydrolases family 32 C terminal	0.664
PF03306	+	Alpha-acetolactate decarboxylase	0.550
PF06054	+	Competence protein CoiA-like family	0.474
PF02449	+	Beta-galactosidase	0.454
PF09922	+	Cell wall-active antibiotics response protein (DUF2154)	0.427
PF01316	+	Arginine repressor, DNA binding domain	0.422
PF03780	+	Asp23 family	0.387
PF08532	+	Beta-galactosidase trimerisation domain	0.379
PF00230	+	Major intrinsic protein	0.376
PF05116	-	Sucrose-6F-phosphate phosphohydrolase	0.373
PF03611	+	PTS system sugar-specific permease component	0.353
PF13396	+	Phospholipase_D-nuclease N-terminal	0.335
PF00195	+	Chalcone and stilbene synthases, N-terminal domain	0.318
PF13579	+	Glycosyl transferase 4-like domain	0.314
PF07745	+	Glycosyl hydrolase family 53	0.309
PF03773	+	Predicted permease	0.296
PF05592	+	Bacterial alpha-L-rhamnosidase	0.292
PF00708	+	Acylphosphatase	0.288
PF00135	+	Carboxylesterase family	0.286
PF02706	+	Chain length determinant protein	0.275
PF04204	+	Homoserine O-succinyltransferase	0.275
PF08713	+	DNA alkylation repair enzyme	0.273
PF01928	+	CYTH domain	0.258
PF02146	+	Sir2 family	0.254
PF13672	-	Protein phosphatase 2C	0.234
PF13345	-	Domain of unknown function (DUF4098)	0.228
PF03881	+	Fructosamine kinase	0.228
PF13596	+	PAS domain	0.227

PF01408	+	Oxidoreductase family, NAD-binding Rossmann fold	0.223
PF04239	+	Protein of unknown function (DUF421)	0.218
PF03632	-	Glycosyl hydrolase family 65 central catalytic domain	0.209
PF00722	+	Glycosyl hydrolases family 16	0.208
PF13483	+	Beta-lactamase superfamily domain	0.200
PF14501	-	GHKL domain	0.195
PF00082	+	Subtilase family	0.195
PF14595	+	Thioredoxin	0.193
PF03633	-	Glycosyl hydrolase family 65, C-terminal domain	0.192
PF13189	-	Cytidylate kinase-like family	0.191
PF13597	-	Anaerobic ribonucleoside-triphosphate reductase	0.184
PF01636	+	Phosphotransferase enzyme family	0.183
PF07859	+	alpha/beta hydrolase fold	0.176
PF13558	+	Putative exonuclease SbcCD, C subunit	0.176
PF13185	+	GAF domain	0.174
PF13303	+	Phosphotransferase system, EIIC	0.173
PF14526	+	Integron-associated effector binding protein	0.171
PF01903	+	CbiX	0.171
PF07501	-	G5 domain	0.170
PF04326	+	Divergent AAA domain	0.168
PF14690	+	zinc-finger of transposase IS204/IS1001/IS1096/IS1165	0.163
PF02237	+	Biotin protein ligase C terminal domain	0.157
PF12867	+	DinB superfamily	0.157
PF02245	+	Methylpurine-DNA glycosylase (MPG)	0.156
PF09860	+	Uncharacterized protein conserved in bacteria (DUF2087)	0.151
PF12098	+	Protein of unknown function (DUF3574)	0.149
PF10111	-	Glycosyltransferase like family 2	0.145
PF03756	+	A-factor biosynthesis hotdog domain	0.141
PF13473	+	Cupredoxin-like domain	0.134
PF06114	-	Domain of unknown function (DUF955)	0.133
PF14041	+	LppP/LprE lipoprotein	0.130
PF00381	+	PTS HPr component phosphorylation site	0.127
PF03808	+	Glycosyl transferase WecB/TagA/CpsF family	0.125
PF00657	+	GDSL-like Lipase/Acylhydrolase	0.123
PF14337	+	Domain of unknown function (DUF4393)	0.123
PF03659	+	Glycosyl hydrolase family 71	0.112
PF04343	+	Protein of unknown function, DUF488	0.112
PF02424	-	ApbE family	0.111
PF11314	+	Protein of unknown function (DUF3117)	0.104
PF04860	+	Phage portal protein	0.103
PF09348	+	Domain of unknown function (DUF1990)	0.097
PF10340	+	Protein of unknown function (DUF2424)	0.096
PF01443	-	Viral (Superfamily 1) RNA helicase	0.095
PF04411	+	Protein of unknown function (DUF524)	0.083
PF04404	-	ERF superfamily	0.083
PF01385	+	Probable transposase	0.082
PF12846	+	AAA-like domain	0.081
PF03575	-	Peptidase family S51	0.072

PF12532	+	Protein of unknown function (DUF3732)	0.072
PF02368	-	Bacterial Ig-like domain (group 2)	0.072
PF03845	-	Spore germination protein	0.069
PF06414	+	Zeta toxin	0.068
PF13280	-	WYL domain	0.067
PF01895	-	PhoU domain	0.059
PF01593	+	Flavin containing amine oxidoreductase	0.053
PF04892	-	VanZ like family	0.051
PF04586	+	Caudovirus prohead protease	0.044
PF03601	-	Conserved hypothetical protein 698	0.043
PF08870	+	Domain of unknown function (DUF1832)	0.043
PF10727	+	Rossmann-like domain	0.041
PF03606	-	C4-dicarboxylate anaerobic carrier	0.040
PF08906	+	Domain of unknown function (DUF1851)	0.039
PF13250	+	Domain of unknown function (DUF4041)	0.036
PF07313	+	Protein of unknown function (DUF1460)	0.036
PF07228	-	Stage II sporulation protein E (SpoIIE)	0.029
PF02436	+	Conserved carboxylase domain	0.029
PF07719	-	Tetratricopeptide repeat	0.029
PF03237	+	Terminase-like family	0.028
PF04332	+	Protein of unknown function (DUF475)	0.025
PF01814	+	Hemerythrin HHE cation binding domain	0.021
PF01548	-	Transposase	0.020
PF05896	+	Na(+)-translocating NADH-quinone reductase subunit A (NQRA)	0.019
PF07969	+	Amidohydrolase family	0.018
PF12679	-	ABC-2 family transporter protein	0.014
PF03412	-	Peptidase C39 family	0.013
PF05163	+	DinB family	0.011
PF01867	+	CRISPR associated protein Cas1	0.009
PF07885	-	Ion channel	0.005
PF00145	+	C-5 cytosine-specific DNA methylase	0.002
PF05684	+	Protein of unknown function (DUF819)	-0.003
PF02652	+	L-lactate permease	-0.003
PF00582	-	Universal stress protein family	-0.004
PF13412	-	Winged helix-turn-helix DNA-binding	-0.014
PF01575	+	MaoC like domain	-0.014
PF11867	+	Domain of unknown function (DUF3387)	-0.019
PF06204	+	Putative carbohydrate binding domain	-0.019
PF06165	+	Glycosyltransferase family 36	-0.019
PF06339	+	Ectoine synthase	-0.020
PF02040	+	Arsenical pump membrane protein	-0.023
PF13310	+	Virulence protein RhuM family	-0.023
PF01663	-	Type I phosphodiesterase / nucleotide pyrophosphatase	-0.024
PF13580	-	SIS domain	-0.025
PF11682	-	Protein of unknown function (DUF3279)	-0.025
PF00182	+	Chitinase class I	-0.026
PF01427	-	D-ala-D-ala dipeptidase	-0.027
PF06480	-	FtsH Extracellular	-0.027

PF13704	+	Glycosyl transferase family 2	-0.027
PF03806	-	AbgT putative transporter family	-0.028
PF10410	+	DnaB-helicase binding domain of primase	-0.033
PF01420	+	Type I restriction modification DNA specificity domain	-0.034
PF06205	+	Glycosyltransferase 36 associated family	-0.035
PF09704	-	CRISPR-associated protein (Cas_Cas5)	-0.037
PF00161	+	Ribosome inactivating protein	-0.037
PF10091	+	Putative glucoamylase	-0.040
PF13593	+	SBF-like CPA transporter family (DUF4137)	-0.043
PF05155	+	Phage X family	-0.043
PF13454	-	FAD-NAD(P)-binding	-0.045
PF01637	-	Archaeal ATPase	-0.047
PF05973	-	Phage derived protein Gp49-like (DUF891)	-0.050
PF04183	-	IucA / IucC family	-0.051
PF08908	-	Domain of unknown function (DUF1852)	-0.053
PF09346	-	SMI1 / KNR4 family (SUKH-1)	-0.054
PF05069	+	Phage virion morphogenesis family	-0.054
PF11066	-	Protein of unknown function (DUF2867)	-0.057
PF12974	-	ABC transporter, phosphonate, periplasmic substrate-binding protein	-0.062
PF06559	-	2'-deoxycytidine 5'-triphosphate deaminase (DCD)	-0.063
PF12832	-	MFS_1 like family	-0.067
PF02082	-	Transcriptional regulator	-0.068
PF02417	+	Chromate transporter	-0.068
PF13728	+	F plasmid transfer operon protein	-0.069
PF00491	+	Arginase family	-0.069
PF01614	-	Bacterial transcriptional regulator	-0.071
PF13401	-	AAA domain	-0.076
PF11896	-	Domain of unknown function (DUF3416)	-0.077
PF04066	+	Multiple resistance and pH regulation protein F (MrpF / PhaF)	-0.078
PF06966	+	Protein of unknown function (DUF1295)	-0.079
PF04221	-	RelB antitoxin	-0.079
PF06719	-	AraC-type transcriptional regulator N-terminus	-0.081
PF02698	-	DUF218 domain	-0.082
PF13744	-	Helix-turn-helix domain	-0.083
PF13714	+	Phosphoenolpyruvate phosphomutase	-0.083
PF06325	-	Ribosomal protein L11 methyltransferase (PrmA)	-0.083
PF01220	+	Dehydroquinase class II	-0.085
PF01809	-	Haemolytic domain	-0.085
PF07681	-	DoxX	-0.086
PF11563	-	Protoglobin	-0.087
PF06833	-	Malonate decarboxylase gamma subunit (MdcE)	-0.094
PF10011	+	Predicted membrane protein (DUF2254)	-0.094
PF04299	-	Putative FMN-binding domain	-0.103
PF03974	-	Ecotin	-0.107
PF07811	-	TadE-like protein	-0.108
PF02133	-	Permease for cytosine/purines, uracil, thiamine, allantoin	-0.108
PF10947	-	Protein of unknown function (DUF2628)	-0.110
PF08239	-	Bacterial SH3 domain	-0.115

PF07963	-	Prokaryotic N-terminal methylation motif	-0.120
PF03824	-	High-affinity nickel-transport protein	-0.127
PF04794	-	YdjC-like protein	-0.127
PF03720	-	UDP-glucose/GDP-mannose dehydrogenase family, UDP binding domain	-0.132
PF06406	-	StbA protein	-0.133
PF08786	-	Domain of unknown function (DUF1795)	-0.133
PF11171	-	Protein of unknown function (DUF2958)	-0.135
PF13581	-	Histidine kinase-like ATPase domain	-0.135
PF01513	-	ATP-NAD kinase	-0.138
PF05199	-	GMC oxidoreductase	-0.139
PF00984	-	UDP-glucose/GDP-mannose dehydrogenase family, central domain	-0.144
PF14759	-	Reductase C-terminal	-0.146
PF00227	-	Proteasome subunit	-0.146
PF00732	-	GMC oxidoreductase	-0.150
PF08816	-	Inhibitor of vertebrate lysozyme (Ivy)	-0.156
PF00933	-	Glycosyl hydrolase family 3 N terminal domain	-0.159
PF13618	-	Gluconate 2-dehydrogenase subunit 3	-0.161
PF02498	-	BRO family, N-terminal domain	-0.163
PF11391	-	Protein of unknown function (DUF2798)	-0.167
PF02900	+	Catalytic LigB subunit of aromatic ring-opening dioxygenase	-0.169
PF14720	+	NiFe/NiFeSe hydrogenase small subunit C-terminal	-0.170
PF04014	-	Antidote-toxin recognition MazE	-0.174
PF00023	-	Ankyrin repeat	-0.180
PF11638	+	DnaA N-terminal domain	-0.180
PF00543	-	Nitrogen regulatory protein P-II	-0.181
PF12837	-	4Fe-4S binding domain	-0.182
PF03527	-	RHS protein	-0.191
PF05284	-	Protein of unknown function (DUF736)	-0.193
PF13089	-	Polyphosphate kinase N-terminal domain	-0.206
PF01925	-	Sulfite exporter TauE/SafE	-0.210
PF06969	-	HemN C-terminal domain	-0.213
PF05726	+	Pirin C-terminal cupin domain	-0.216
PF00565	-	Staphylococcal nuclease homologue	-0.218
PF02503	-	Polyphosphate kinase middle domain	-0.219
PF05573	-	NosL	-0.221
PF05962	-	HutD	-0.223
PF13905	-	Thioredoxin-like	-0.228
PF04264	-	YceI-like domain	-0.228
PF02416	-	mttA/Hcf106 family	-0.229
PF03625	-	Domain of unknown function DUF302	-0.230
PF01326	-	Pyruvate phosphate dikinase, PEP/pyruvate binding domain	-0.230
PF14497	-	Glutathione S-transferase, C-terminal domain	-0.233
PF13857	-	Ankyrin repeats (many copies)	-0.234
PF07498	-	Rho termination factor, N-terminal domain	-0.238
PF01551	-	Peptidase family M23	-0.239
PF06277	-	Ethanolamine utilisation protein EutA	-0.257
PF04963	-	Sigma-54 factor, core binding domain	-0.263
PF00309	-	Sigma-54 factor, Activator interacting domain (AID)	-0.264

PF03971	-	Monomeric isocitrate dehydrogenase	-0.264
PF01804	-	Penicillin amidase	-0.267
PF01124	+	MAPEG family	-0.270
PF13766	-	2-enoyl-CoA Hydratase C-terminal region	-0.275
PF07497	-	Rho termination factor, RNA-binding domain	-0.282
PF01627	-	Hpt domain	-0.284
PF04115	-	Ureidoglycolate hydrolase	-0.288
PF13362	-	Toprim domain	-0.293
PF06968	-	Biotin and Thiamin Synthesis associated domain	-0.297
PF10135	-	Rod binding protein	-0.300
PF00529	-	HlyD family secretion protein	-0.306
PF03176	-	MMPL family	-0.325
PF05658	-	Head domain of trimeric autotransporter adhesin	-0.340
PF14532	-	Sigma-54 interaction domain	-0.358
PF10588	-	NADH-ubiquinone oxidoreductase-G iron-sulfur binding region	-0.364
PF02550	-	Acetyl-CoA hydrolase/transferase N-terminal domain	-0.365
PF13510	-	2Fe-2S iron-sulfur cluster binding domain	-0.366
PF01258	-	Prokaryotic dksA/traR C4-type zinc finger	-0.377
PF03572	-	Peptidase family S41	-0.381

Phenotype: Trehalose (Growth: Sugar), Predictor: phypat

Pfam	class	Pfam_desc	cor
PF07702	+	UTRA domain	0.564
PF00232	+	Glycosyl hydrolase family 1	0.561
PF02255	+	PTS system, Lactose/Cellobiose specific IIA subunit	0.561
PF03932	+	CutC family	0.548
PF00367	+	phosphotransferase system, EIIB	0.542
PF03830	+	PTS system sorbose subfamily IIB component	0.535
PF04069	+	Substrate binding domain of ABC-type glycine betaine transport system	0.523
PF01238	+	Phosphomannose isomerase type I	0.516
PF04237	+	YjbR	0.502
PF00667	+	FAD binding domain	0.500
PF07085	+	DRTGG domain	0.487
PF00251	+	Glycosyl hydrolases family 32 N-terminal domain	0.474
PF00854	+	POT family	0.472
PF03551	+	Transcriptional regulator PadR-like family	0.469
PF06445	+	GyrI-like small molecule binding domain	0.458
PF04235	+	Protein of unknown function (DUF418)	0.456
PF01583	+	Adenylylsulphate kinase	0.444
PF08244	+	Glycosyl hydrolases family 32 C terminal	0.437
PF12464	+	Maltose acetyltransferase	0.433
PF13556	+	PucR C-terminal helix-turn-helix domain	0.428
PF02733	+	Dak1 domain	0.412
PF02901	+	Pyruvate formate lyase	0.399
PF07470	+	Glycosyl Hydrolase Family 88	0.393
PF14526	+	Integron-associated effector binding protein	0.393
PF06224	+	Winged helix DNA-binding domain	0.389
PF04898	+	Glutamate synthase central domain	0.388

PF02706	+	Chain length determinant protein	0.387
PF02016	+	LD-carboxypeptidase	0.382
PF12648	+	TcpE family	0.377
PF04261	+	Dyp-type peroxidase family	0.369
PF01909	+	Nucleotidyltransferase domain	0.359
PF12708	+	Pectate lyase superfamily protein	0.355
PF06993	+	Protein of unknown function (DUF1304)	0.353
PF01638	+	HxlR-like helix-turn-helix	0.342
PF03773	+	Predicted permease	0.331
PF04860	+	Phage portal protein	0.327
PF04616	+	Glycosyl hydrolases family 43	0.322
PF04284	+	Protein of unknown function (DUF441)	0.322
PF14502	+	Helix-turn-helix domain	0.318
PF13906	+	C-terminus of AA_permease	0.310
PF02036	+	SCP-2 sterol transfer family	0.303
PF05368	+	NmrA-like family	0.302
PF13576	+	Pentapeptide repeats (9 copies)	0.293
PF12242	+	NAD(P)H binding domain of trans-2-enoyl-CoA reductase	0.280
PF12558	+	ATP-binding cassette cobalt transporter	0.280
PF13714	+	Phosphoenolpyruvate phosphomutase	0.272
PF00290	+	Tryptophan synthase alpha chain	0.268
PF12910	+	Antitoxin of toxin-antitoxin stability system N-terminal	0.265
PF13710	+	ACT domain	0.264
PF03595	+	Voltage-dependent anion channel	0.254
PF10979	+	Protein of unknown function (DUF2786)	0.253
PF04230	+	Polysaccharide pyruvyl transferase	0.250
PF00302	+	Chloramphenicol acetyltransferase	0.245
PF13358	+	DDE superfamily endonuclease	0.241
PF13382	+	Adenine deaminase C-terminal domain	0.233
PF08808	+	RES domain	0.214
PF04586	+	Caudovirus prohead protease	0.190
PF14542	+	GCN5-related N-acetyl-transferase	0.171
PF07853	+	Protein of unknown function (DUF1648)	0.166
PF07075	+	Protein of unknown function (DUF1343)	0.150
PF02224	-	Cytidylate kinase	0.146
PF02803	-	Thiolase, C-terminal domain	0.142
PF07811	+	TadE-like protein	0.129
PF03606	-	C4-dicarboxylate anaerobic carrier	0.123
PF05525	-	Branched-chain amino acid transport protein	0.122
PF14010	+	Phosphoenolpyruvate carboxylase	0.114
PF04072	+	Leucine carboxyl methyltransferase	0.109
PF06961	-	Protein of unknown function (DUF1294)	0.106
PF13428	+	Tetratricopeptide repeat	0.095
PF12682	+	Flavodoxin	0.091
PF03601	-	Conserved hypothetical protein 698	0.089
PF03703	+	Bacterial PH domain	0.086
PF01192	-	RNA polymerase Rpb6	0.080
PF00488	-	MutS domain V	0.059

PF05402	-	Coenzyme PQQ synthesis protein D (PqqD)	0.055
PF10551	-	MULE transposase domain	0.040
PF12146	-	Putative lysophospholipase	0.036
PF13396	-	Phospholipase_D-nuclease N-terminal	0.036
PF04205	-	FMN-binding domain	0.034
PF01315	-	Aldehyde oxidase and xanthine dehydrogenase, a/b hammerhead domain	0.029
PF13545	-	Crp-like helix-turn-helix domain	0.027
PF13589	-	Histidine kinase-, DNA gyrase B-, and HSP90-like ATPase	0.019
PF03616	-	Sodium/glutamate symporter	0.017
PF00306	-	ATP synthase alpha/beta chain, C terminal domain	0.011
PF00939	-	Sodium:sulfate symporter transmembrane region	0.006
PF03142	-	Chitin synthase	0.005
PF06912	-	Protein of unknown function (DUF1275)	-0.002
PF01609	-	Transposase DDE domain	-0.011
PF01070	-	FMN-dependent dehydrogenase	-0.022
PF02086	-	D12 class N6 adenine-specific DNA methyltransferase	-0.022
PF06808	-	DctM-like transporters	-0.036
PF13088	-	BNR repeat-like domain	-0.052
PF04326	-	Divergent AAA domain	-0.056
PF01850	-	PIN domain	-0.058
PF03444	-	Winged helix-turn-helix transcription repressor, HrcA DNA-binding	-0.060
PF00239	-	Resolvase, N terminal domain	-0.066
PF02683	-	Cytochrome C biogenesis protein transmembrane region	-0.081
PF04610	-	TrbL/VirB6 plasmid conjugal transfer protein	-0.086
PF00188	-	Cysteine-rich secretory protein family	-0.094
PF08392	-	FAE1/Type III polyketide synthase-like protein	-0.099
PF05014	-	Nucleoside 2-deoxyribosyltransferase	-0.101
PF07927	-	YcfA-like protein	-0.106
PF01551	-	Peptidase family M23	-0.113
PF03572	-	Peptidase family S41	-0.114
PF01628	-	HrcA protein C terminal domain	-0.122
PF04296	-	Protein of unknown function (DUF448)	-0.126
PF08207	-	Elongation factor P (EF-P) KOW-like domain	-0.137
PF03681	-	Uncharacterised protein family (UPF0150)	-0.139
PF00924	-	Mechanosensitive ion channel	-0.158
PF02384	-	N-6 DNA Methylase	-0.161
PF03432	-	Relaxase/Mobilisation nuclease domain	-0.161
PF01012	-	Electron transfer flavoprotein domain	-0.165
PF01555	-	DNA methylase	-0.168
PF02515	-	CoA-transferase family III	-0.170
PF11867	-	Domain of unknown function (DUF3387)	-0.171
PF13011	-	leucine-zipper of insertion element IS481	-0.176
PF01888	-	CbiD	-0.185
PF13491	-	Domain of unknown function (DUF4117)	-0.201
PF03576	-	Peptidase family S58	-0.232
PF13173	-	AAA domain	-0.269
PF03030	-	Inorganic H+ pyrophosphatase	-0.270
PF01637	-	Archaeal ATPase	-0.293

PF00142	-	4Fe-4S iron sulfur cluster binding proteins, NifH/frxC family	-0.328

Phenotype: Trehalose (Growth: Sugar), Predictor: phypat+PGL

Pfam	class	Pfam_desc	cor
PF07702	+	UTRA domain	0.564
PF00232	+	Glycosyl hydrolase family 1	0.561
PF03932	+	CutC family	0.548
PF04069	+	Substrate binding domain of ABC-type glycine betaine transport system	0.523
PF04237	+	YjbR	0.502
PF00667	+	FAD binding domain	0.500
PF00265	+	Thymidine kinase	0.491
PF00251	+	Glycosyl hydrolases family 32 N-terminal domain	0.474
PF00854	+	POT family	0.472
PF03551	+	Transcriptional regulator PadR-like family	0.469
PF11975	+	Family 4 glycosyl hydrolase C-terminal domain	0.463
PF02056	+	Family 4 glycosyl hydrolase	0.463
PF06445	+	GyrI-like small molecule binding domain	0.458
PF04235	+	Protein of unknown function (DUF418)	0.456
PF08402	+	TOBE domain	0.448
PF01583	+	Adenylylsulphate kinase	0.444
PF12464	+	Maltose acetyltransferase	0.433
PF05343	+	M42 glutamyl aminopeptidase	0.430
PF03611	+	PTS system sugar-specific permease component	0.418
PF02733	+	Dak1 domain	0.412
PF00781	+	Diacylglycerol kinase catalytic domain	0.402
PF00393	+	6-phosphogluconate dehydrogenase, C-terminal domain	0.401
PF02901	+	Pyruvate formate lyase	0.399
PF14526	+	Integron-associated effector binding protein	0.393
PF00365	+	Phosphofructokinase	0.391
PF06224	+	Winged helix DNA-binding domain	0.389
PF04657	+	Protein of unknown function, DUF606	0.388
PF02016	+	LD-carboxypeptidase	0.382
PF01943	+	Polysaccharide biosynthesis protein	0.379
PF02030	+	Hypothetical lipoprotein (MG045 family)	0.373
PF03390	+	2-hydroxycarboxylate transporter family	0.370
PF13360	+	PQQ-like domain	0.358
PF05336	+	Domain of unknown function (DUF718)	0.358
PF06993	+	Protein of unknown function (DUF1304)	0.353
PF00334	+	Nucleoside diphosphate kinase	0.349
PF01503	+	Phosphoribosyl-ATP pyrophosphohydrolase	0.347
PF03773	+	Predicted permease	0.331
PF04860	+	Phage portal protein	0.327
PF04993	+	TfoX N-terminal domain	0.322
PF13521	+	AAA domain	0.311
PF13906	+	C-terminus of AA_permease	0.310
PF02894	+	Oxidoreductase family, C-terminal alpha/beta domain	0.308
PF00343	+	Carbohydrate phosphorylase	0.293
PF03354	+	Phage Terminase	0.292

PF05135	+	Phage gp6-like head-tail connector protein	0.290
PF01699	+	Sodium/calcium exchanger protein	0.288
PF02806	+	Alpha amylase, C-terminal all-beta domain	0.278
PF04794	+	YdjC-like protein	0.278
PF00195	+	Chalcone and stilbene synthases, N-terminal domain	0.272
PF13646	+	HEAT repeats	0.271
PF00682	+	HMGL-like	0.270
PF12910	+	Antitoxin of toxin-antitoxin stability system N-terminal	0.265
PF13710	+	ACT domain	0.264
PF08029	+	HisG, C-terminal domain	0.262
PF06983	+	3-demethylubiquinone-9 3-methyltransferase	0.259
PF03595	+	Voltage-dependent anion channel	0.254
PF04389	+	Peptidase family M28	0.250
PF04230	+	Polysaccharide pyruvyl transferase	0.250
PF03949	+	Malic enzyme, NAD binding domain	0.249
PF00390	+	Malic enzyme, N-terminal domain	0.249
PF01643	+	Acyl-ACP thioesterase	0.247
PF05866	+	Endodeoxyribonuclease RusA	0.247
PF00977	+	Histidine biosynthesis protein	0.245
PF04023	+	FeoA domain	0.235
PF02583	+	Metal-sensitive transcriptional repressor	0.234
PF13376	+	Bacteriocin-protection, YdeI or OmpD-Associated	0.232
PF13347	-	MFS/sugar transport protein	0.219
PF05724	+	Thiopurine S-methyltransferase (TPMT)	0.218
PF07103	+	Protein of unknown function (DUF1365)	0.211
PF05495	+	CHY zinc finger	0.211
PF01094	+	Receptor family ligand binding region	0.210
PF11974	+	Alpha-2-macroglobulin MG1 domain	0.207
PF04608	+	Phosphatidylglycerophosphatase A	0.205
PF03073	+	TspO/MBR family	0.195
PF08218	-	Citrate lyase ligase C-terminal domain	0.192
PF13154	+	Protein of unknown function (DUF3991)	0.181
PF05272	+	Virulence-associated protein E	0.180
PF01738	-	Dienelactone hydrolase family	0.174
PF07523	-	Bacterial Ig-like domain (group 3)	0.171
PF01739	-	CheR methyltransferase, SAM binding domain	0.168
PF07853	+	Protein of unknown function (DUF1648)	0.166
PF01175	+	Urocanase	0.158
PF13683	-	Integrase core domain	0.151
PF01661	+	Macro domain	0.149
PF13936	-	Helix-turn-helix domain	0.145
PF05145	-	Putative ammonia monooxygenase	0.142
PF00268	-	Ribonucleotide reductase, small chain	0.140
PF01208	-	Uroporphyrinogen decarboxylase (URO-D)	0.139
PF09617	+	CRISPR-associated protein GSU0053 (Cas_GSU0053)	0.137
PF01174	+	SNO glutamine amidotransferase family	0.136
PF03205	-	Molybdopterin guanine dinucleotide synthesis protein B	0.134
PF13378	-	Enolase C-terminal domain-like	0.131

PF00722	+	Glycosyl hydrolases family 16	0.129
PF01641	-	SelR domain	0.120
PF07729	-	FCD domain	0.119
PF11188	-	Protein of unknown function (DUF2975)	0.119
PF13510	+	2Fe-2S iron-sulfur cluster binding domain	0.118
PF07498	+	Rho termination factor, N-terminal domain	0.116
PF03707	-	Bacterial signalling protein N terminal repeat	0.115
PF14010	+	Phosphoenolpyruvate carboxylase	0.114
PF03806	+	AbgT putative transporter family	0.110
PF04072	+	Leucine carboxyl methyltransferase	0.109
PF06826	+	Predicted Permease Membrane Region	0.109
PF01256	-	Carbohydrate kinase	0.106
PF06769	+	Plasmid encoded toxin Txe	0.105
PF09355	+	Phage protein Gp19/Gp15/Gp42	0.103
PF05163	-	DinB family	0.101
PF02796	-	Helix-turn-helix domain of resolvase	0.100
PF00375	-	Sodium:dicarboxylate symporter family	0.098
PF06207	-	Protein of unknown function (DUF1002)	0.095
PF13428	+	Tetratricopeptide repeat	0.095
PF13551	+	Winged helix-turn helix	0.093
PF12682	+	Flavodoxin	0.091
PF09818	+	Predicted ATPase of the ABC class	0.086
PF01797	-	Transposase IS200 like	0.086
PF02424	-	ApbE family	0.076
PF06965	+	Na+/H+ antiporter 1	0.073
PF02148	+	Zn-finger in ubiquitin-hydrolases and other protein	0.072
PF05133	-	Phage portal protein, SPP1 Gp6-like	0.065
PF13342	-	C-terminal repeat of topoisomerase	0.060
PF02599	-	Global regulator protein family	0.055
PF08666	-	SAF domain	0.045
PF12146	-	Putative lysophospholipase	0.036
PF04199	-	Putative cyclase	0.036
PF05099	-	Tellurite resistance protein TerB	0.036
PF00176	-	SNF2 family N-terminal domain	0.034
PF04205	-	FMN-binding domain	0.034
PF00041	-	Fibronectin type III domain	0.033
PF13408	-	Recombinase zinc beta ribbon domain	0.028
PF03741	-	Integral membrane protein TerC family	0.026
PF11208	-	Protein of unknown function (DUF2992)	0.020
PF01730	-	UreF	0.014
PF05194	-	UreE urease accessory protein, C-terminal domain	-0.001
PF13247	-	4Fe-4S dicluster domain	-0.003
PF10926	-	Protein of unknown function (DUF2800)	-0.005
PF01609	-	Transposase DDE domain	-0.011
PF01869	+	BadF/BadG/BcrA/BcrD ATPase family	-0.012
PF02086	-	D12 class N6 adenine-specific DNA methyltransferase	-0.022
PF00493	-	MCM2/3/5 family	-0.027
PF06808	-	DctM-like transporters	-0.036

PF06564	-	YhjQ protein	-0.049
PF03480	-	Bacterial extracellular solute-binding protein, family 7	-0.052
PF13088	-	BNR repeat-like domain	-0.052
PF00899	-	ThiF family	-0.053
PF01850	-	PIN domain	-0.058
PF00529	-	HlyD family secretion protein	-0.058
PF01728	-	FtsJ-like methyltransferase	-0.059
PF11148	-	Protein of unknown function (DUF2922)	-0.061
PF12229	-	Putative peptidoglycan binding domain	-0.086
PF13229	-	Right handed beta helix region	-0.088
PF06271	-	RDD family	-0.089
PF04313	-	Type I restriction enzyme R protein N terminus (HSDR_N)	-0.089
PF02335	-	Cytochrome c552	-0.100
PF07501	-	G5 domain	-0.103
PF02277	-	Phosphoribosyltransferase	-0.116
PF01867	-	CRISPR associated protein Cas1	-0.116
PF05949	-	Bacterial protein of unknown function (DUF881)	-0.122
PF06947	-	Protein of unknown function (DUF1290)	-0.122
PF01930	+	Domain of unknown function DUF83	-0.134
PF03681	-	Uncharacterised protein family (UPF0150)	-0.139
PF02384	-	N-6 DNA Methylase	-0.161
PF03432	-	Relaxase/Mobilisation nuclease domain	-0.161
PF01555	-	DNA methylase	-0.168
PF04011	-	LemA family	-0.172
PF03976	-	Polyphosphate kinase 2 (PPK2)	-0.187
PF07695	-	7TM diverse intracellular signalling	-0.194
PF13491	-	Domain of unknown function (DUF4117)	-0.201
PF13173	-	AAA domain	-0.269
PF05970	-	PIF1-like helicase	-0.343

Phenotype: Urea hydrolysis (Growth: Sugar), Predictor: phypat

Pfam	class	Pfam_desc	cor
PF01774	+	UreD urease accessory protein	0.789
PF02814	+	UreE urease accessory protein, N-terminal domain	0.771
PF05199	+	GMC oxidoreductase	0.528
PF06172	+	Cupin superfamily (DUF985)	0.353
PF06628	+	Catalase-related immune-responsive	0.303
PF13622	+	Thioesterase-like superfamily	0.295
PF05232	+	Bacterial Transmembrane Pair family	0.271
PF03186	+	CobD/Cbib protein	0.270
PF04199	+	Putative cyclase	0.269
PF02733	+	Dak1 domain	0.233
PF00668	+	Condensation domain	0.164
PF08843	+	Nucleotidyl transferase of unknown function (DUF1814)	0.121
PF02667	-	Short chain fatty acid transporter	-0.039
PF01734	-	Patatin-like phospholipase	-0.062
PF04998	-	RNA polymerase Rpb1, domain 5	-0.104
PF04997	-	RNA polymerase Rpb1, domain 1	-0.104

PF00623	-	RNA polymerase Rpb1, domain 2	-0.104
PF04011	-	LemA family	-0.130
PF13440	-	Polysaccharide biosynthesis protein	-0.142
PF01464	-	Transglycosylase SLT domain	-0.145
PF05857	-	TraX protein	-0.151
PF06480	-	FtsH Extracellular	-0.153
PF00056	-	lactate/malate dehydrogenase, NAD binding domain	-0.157
PF05000	-	RNA polymerase Rpb1, domain 4	-0.160
PF01661	-	Macro domain	-0.167
PF05598	-	Transposase domain (DUF772)	-0.169
PF01227	-	GTP cyclohydrolase I	-0.179
PF13704	-	Glycosyl transferase family 2	-0.189
PF00142	-	4Fe-4S iron sulfur cluster binding proteins, NifH/frxC family	-0.192
PF00654	-	Voltage gated chloride channel	-0.201
PF01207	-	Dihydrouridine synthase (Dus)	-0.221
PF13588	-	Type I restriction enzyme R protein N terminus (HSDR_N)	-0.263
PF02086	-	D12 class N6 adenine-specific DNA methyltransferase	-0.275
PF01906	-	Putative heavy-metal-binding	-0.286
PF05116	-	Sucrose-6F-phosphate phosphohydrolase	-0.317
PF02664	-	S-Ribosylhomocysteinase (LuxS)	-0.357
PF13495	-	Phage integrase, N-terminal SAM-like domain	-0.361

Phenotype: Urea hydrolysis (Growth: Sugar), Predictor: phypat+PGL

Pfam	class	Pfam_desc	cor
PF01774	+	UreD urease accessory protein	0.789
PF00699	+	Urease beta subunit	0.774
PF00449	+	Urease alpha-subunit, N-terminal domain	0.774
PF02814	+	UreE urease accessory protein, N-terminal domain	0.771
PF01730	+	UreF	0.749
PF09490	+	Probable cobalt transporter subunit (CbtA)	0.429
PF03253	+	Urea transporter	0.420
PF10861	+	Protein of Unknown function (DUF2784)	0.382
PF04066	+	Multiple resistance and pH regulation protein F (MrpF / PhaF)	0.366
PF06172	+	Cupin superfamily (DUF985)	0.353
PF03243	+	Alkylmercury lyase	0.327
PF11860	+	Protein of unknown function (DUF3380)	0.314
PF08421	+	Putative zinc binding domain	0.303
PF07080	+	Protein of unknown function (DUF1348)	0.303
PF04199	+	Putative cyclase	0.269
PF03625	+	Domain of unknown function DUF302	0.259
PF02633	+	Creatinine amidohydrolase	0.258
PF02570	+	Precorrin-8X methylmutase	0.254
PF09275	+	Pertussis toxin S4 subunit	0.243
PF02571	+	Precorrin-6x reductase CbiJ/CobK	0.233
PF02668	+	Taurine catabolism dioxygenase TauD, TfdA family	0.230
PF00394	+	Multicopper oxidase	0.209
PF12900	+	Pyridoxamine 5'-phosphate oxidase	0.184
PF07103	+	Protein of unknown function (DUF1365)	0.182

PF05544	+	Proline racemase	0.181
PF02805	+	Metal binding domain of Ada	0.178
PF01208	-	Uroporphyrinogen decarboxylase (URO-D)	0.175
PF03869	+	Arc-like DNA binding domain	0.171
PF11459	+	Protein of unknwon function (DUF2893)	0.170
PF00668	+	Condensation domain	0.164
PF05048	+	Periplasmic copper-binding protein (NosD)	0.157
PF01921	+	tRNA synthetases class I (K)	0.145
PF09707	+	CRISPR-associated protein (Cas_Cas2CT1978)	0.134
PF13391	+	HNH endonuclease	0.133
PF00902	-	Sec-independent protein translocase protein (TatC)	0.133
PF12684	+	PDDEXK-like domain of unknown function (DUF3799)	0.130
PF14269	+	Arylsulfotransferase (ASST)	0.130
PF12221	-	Bacterial membrane protein N terminal	0.122
PF01890	+	Cobalamin synthesis G C-terminus	0.122
PF03616	+	Sodium/glutamate symporter	0.122
PF14253	+	Bacteriophage abortive infection AbiH	0.115
PF05114	+	Protein of unknown function (DUF692)	0.097
PF02913	-	FAD linked oxidases, C-terminal domain	0.097
PF13166	+	AAA domain	0.094
PF05930	-	Prophage CP4-57 regulatory protein (AlpA)	0.092
PF13156	+	Restriction endonuclease	0.086
PF01981	+	Peptidyl-tRNA hydrolase PTH2	0.081
PF11795	+	Uncharacterized protein conserved in bacteria N-term (DUF3322)	0.080
PF06545	+	Protein of unknown function (DUF1116)	0.073
PF03767	+	HAD superfamily, subfamily IIIB (Acid phosphatase)	0.069
PF06048	+	Domain of unknown function (DUF927)	0.059
PF04261	-	Dyp-type peroxidase family	0.055
PF13338	+	Domain of unknown function (DUF4095)	0.053
PF06892	+	Phage regulatory protein CII (CP76)	0.052
PF02040	+	Arsenical pump membrane protein	0.050
PF14436	+	Bacterial EndoU nuclease	0.049
PF00145	+	C-5 cytosine-specific DNA methylase	0.043
PF13559	+	Domain of unknown function (DUF4129)	0.039
PF13435	+	Cytochrome c554 and c-prime	0.016
PF11870	-	Domain of unknown function (DUF3390)	0.012
PF09932	-	Uncharacterized conserved protein (DUF2164)	-0.004
PF03837	+	RecT family	-0.005
PF01503	+	Phosphoribosyl-ATP pyrophosphohydrolase	-0.012
PF03606	+	C4-dicarboxylate anaerobic carrier	-0.015
PF01139	+	tRNA-splicing ligase RtcB	-0.020
PF11127	+	Protein of unknown function (DUF2892)	-0.022
PF13517	-	Repeat domain in Vibrio, Colwellia, Bradyrhizobium and Shewanella	-0.031
PF07282	+	Putative transposase DNA-binding domain	-0.032
PF02545	-	Maf-like protein	-0.035
PF13289	+	SIR2-like domain	-0.047
PF04999	-	Cell division protein FtsL	-0.049
PF13742	-	OB-fold nucleic acid binding domain	-0.052

PF06769	+	Plasmid encoded toxin Txe	-0.062
PF13175	+	AAA ATPase domain	-0.067
PF03275	-	UDP-galactopyranose mutase	-0.072
PF02719	-	Polysaccharide biosynthesis protein	-0.099
PF09140	-	ATPase MipZ	-0.099
PF03572	-	Peptidase family S41	-0.099
PF05973	-	Phage derived protein Gp49-like (DUF891)	-0.104
PF01420	+	Type I restriction modification DNA specificity domain	-0.106
PF00692	-	dUTPase	-0.116
PF01637	+	Archaeal ATPase	-0.121
PF12102	-	Domain of unknown function (DUF3578)	-0.124
PF13567	-	Domain of unknown function (DUF4131)	-0.126
PF07663	-	Sorbitol phosphotransferase enzyme II C-terminus	-0.127
PF04011	-	LemA family	-0.130
PF01745	+	Isopentenyl transferase	-0.132
PF12728	-	Helix-turn-helix domain	-0.137
PF10396	-	GTP-binding protein TrmE N-terminus	-0.138
PF01715	-	IPP transferase	-0.140
PF06356	-	Protein of unknown function (DUF1064)	-0.142
PF12889	-	Protein of unknown function (DUF3829)	-0.142
PF13402	-	Peptidase M60-like family	-0.143
PF05192	-	MutS domain III	-0.145
PF01624	-	MutS domain I	-0.145
PF01464	-	Transglycosylase SLT domain	-0.145
PF02590	-	Predicted SPOUT methyltransferase	-0.148
PF01391	-	Collagen triple helix repeat (20 copies)	-0.151
PF04608	-	Phosphatidylglycerophosphatase A	-0.159
PF14393	-	Domain of unknown function (DUF4422)	-0.165
PF08543	-	Phosphomethylpyrimidine kinase	-0.167
PF01661	-	Macro domain	-0.167
PF01136	-	Peptidase family U32	-0.180
PF07977	-	FabA-like domain	-0.194
PF12850	-	Calcineurin-like phosphoesterase superfamily domain	-0.199
PF04221	-	RelB antitoxin	-0.206
PF01938	-	TRAM domain	-0.216
PF01207	-	Dihydrouridine synthase (Dus)	-0.221
PF13306	-	Leucine rich repeats (6 copies)	-0.230
PF10418	-	Iron-sulfur cluster binding domain of dihydroorotate dehydrogenase B	-0.263
PF13588	-	Type I restriction enzyme R protein N terminus (HSDR_N)	-0.263
PF13636	-	pre-rRNA processing and ribosome biogenesis	-0.273
PF02086	-	D12 class N6 adenine-specific DNA methyltransferase	-0.275
PF00044	-	Glyceraldehyde 3-phosphate dehydrogenase, NAD binding domain	-0.299
PF13495	-	Phage integrase, N-terminal SAM-like domain	-0.361

Phenotype: Bacillus or coccobacillus (Morphology), Predictor: phypat

Pfam	class	Pfam_desc	cor
PF01220	+	Dehydroquinase class II	0.582
PF03880	+	DbpA RNA binding domain	0.522

PF01188	+	Mandelate racemase / muconate lactonizing enzyme, C-terminal domain	0.499
PF07638	+	ECF sigma factor	0.494
PF00733	+	Asparagine synthase	0.486
PF08447	+	PAS fold	0.469
PF07676	+	WD40-like Beta Propeller Repeat	0.451
PF10410	+	DnaB-helicase binding domain of primase	0.430
PF03775	+	Septum formation inhibitor MinC, C-terminal domain	0.421
PF06609	+	Fungal trichothecene efflux pump (TRI12)	0.403
PF00563	+	EAL domain	0.403
PF13524	+	Glycosyl transferases group 1	0.399
PF13231	+	Dolichyl-phosphate-mannose-protein mannosyltransferase	0.397
PF13144	+	SAF-like	0.396
PF02277	+	Phosphoribosyltransferase	0.391
PF00022	+	Actin	0.386
PF04316	+	Anti-sigma-28 factor, FlgM	0.382
PF13464	+	Domain of unknown function (DUF4115)	0.376
PF01923	+	Cobalamin adenosyltransferase	0.370
PF07885	+	Ion channel	0.346
PF04962	+	KduI/IolB family	0.336
PF12897	+	Alanine-glyoxylate amino-transferase	0.329
PF00227	+	Proteasome subunit	0.317
PF01915	+	Glycosyl hydrolase family 3 C-terminal domain	0.316
PF12146	+	Putative lysophospholipase	0.300
PF00596	+	Class II Aldolase and Adducin N-terminal domain	0.295
PF02574	+	Homocysteine S-methyltransferase	0.278
PF13458	+	Periplasmic binding protein	0.228
PF03576	+	Peptidase family S58	0.219
PF03711	+	Orn/Lys/Arg decarboxylase, C-terminal domain	0.211
PF10543	+	ORF6N domain	0.207
PF02311	+	AraC-like ligand binding domain	0.205
PF03990	+	Domain of unknown function (DUF348)	0.205
PF13350	+	Tyrosine phosphatase family	0.202
PF13593	-	SBF-like CPA transporter family (DUF4137)	0.195
PF01276	+	Orn/Lys/Arg decarboxylase, major domain	0.192
PF04461	-	Protein of unknown function (DUF520)	0.181
PF02929	+	Beta galactosidase small chain	0.165
PF04389	-	Peptidase family M28	0.153
PF09704	+	CRISPR-associated protein (Cas_Cas5)	0.152
PF00082	+	Subtilase family	0.112
PF01206	-	Sulfurtransferase TusA	0.106
PF01693	+	Caulimovirus viroplasmin	0.100
PF03237	+	Terminase-like family	0.081
PF13114	+	RecO N terminal	0.064
PF13409	-	Glutathione S-transferase, N-terminal domain	0.063
PF02028	-	BCCT family transporter	0.060
PF02903	+	Alpha amylase, N-terminal ig-like domain	0.041
PF00850	-	Histone deacetylase domain	0.028
PF03969	-	AFG1-like ATPase	0.021

PF04266	+	ASCH domain	0.016
PF03747	-	ADP-ribosylglycohydrolase	0.015
PF03448	-	MgtE intracellular N domain	-0.022
PF04892	+	VanZ like family	-0.026
PF13454	-	FAD-NAD(P)-binding	-0.036
PF03060	+	Nitronate monooxygenase	-0.057
PF01769	-	Divalent cation transporter	-0.058
PF04131	+	Putative N-acetylmannosamine-6-phosphate epimerase	-0.072
PF03328	-	HpcH/HpaI aldolase/citrate lyase family	-0.097
PF13530	-	Sterol carrier protein domain	-0.101
PF13434	-	L-lysine 6-monooxygenase (NADPH-requiring)	-0.128
PF13669	-	Glyoxalase/Bleomycin resistance protein/Dioxygenase superfamily	-0.128
PF12681	-	Glyoxalase-like domain	-0.162
PF04093	-	rod shape-determining protein MreD	-0.164
PF02557	-	D-alanyl-D-alanine carboxypeptidase	-0.192
PF03352	-	Methyladenine glycosylase	-0.197
PF00359	-	Phosphoenolpyruvate-dependent sugar phosphotransferase system, EIIA 2	-0.200
PF09587	-	Bacterial capsule synthesis protein PGA_cap	-0.305
PF02616	-	ScpA/B protein	-0.333
PF08535	-	KorB domain	-0.341
PF04079	-	Putative transcriptional regulators (Ypuh-like)	-0.349
PF04018	-	Domain of unknown function (DUF368)	-0.367
PF05913	-	Bacterial protein of unknown function (DUF871)	-0.368
PF06737	-	Transglycosylase-like domain	-0.432
PF07949	-	YbbR-like protein	-0.462
PF02229	-	Transcriptional Coactivator p15 (PC4)	-0.468
PF10031	-	Small integral membrane protein (DUF2273)	-0.532
PF01487	-	Type I 3-dehydroquinase	-0.561
PF02388	-	FemAB family	-0.570

Phenotype: Bacillus or coccobacillus (Morphology), Predictor: phypat+PGL

Pfam	class	Pfam_desc	cor
PF01220	+	Dehydroquinase class II	0.582
PF03880	+	DbpA RNA binding domain	0.522
PF01188	+	Mandelate racemase / muconate lactonizing enzyme, C-terminal domain	0.499
PF07638	+	ECF sigma factor	0.494
PF00733	+	Asparagine synthase	0.486
PF13292	+	1-deoxy-D-xylulose-5-phosphate synthase	0.464
PF13624	+	SurA N-terminal domain	0.437
PF10410	+	DnaB-helicase binding domain of primase	0.430
PF03775	+	Septum formation inhibitor MinC, C-terminal domain	0.421
PF13401	+	AAA domain	0.420
PF02602	+	Uroporphyrinogen-III synthase HemD	0.415
PF02355	+	Protein export membrane protein	0.413
PF06609	+	Fungal trichothecene efflux pump (TRI12)	0.403
PF13286	+	Phosphohydrolase-associated domain	0.403
PF00563	+	EAL domain	0.403
PF09339	+	IclR helix-turn-helix domain	0.399

PF13231	+	Dolichyl-phosphate-mannose-protein mannosyltransferase	0.397
PF03176	+	MMPL family	0.394
PF02277	+	Phosphoribosyltransferase	0.391
PF00022	+	Actin	0.386
PF06965	+	Na+/H+ antiporter 1	0.378
PF13464	+	Domain of unknown function (DUF4115)	0.376
PF01923	+	Cobalamin adenosyltransferase	0.370
PF13432	+	Tetratricopeptide repeat	0.362
PF07885	+	Ion channel	0.346
PF01882	+	Protein of unknown function DUF58	0.330
PF06305	+	Protein of unknown function (DUF1049)	0.328
PF02678	-	Pirin	0.314
PF08666	-	SAF domain	0.311
PF00668	+	Condensation domain	0.307
PF14310	+	Fibronectin type III-like domain	0.302
PF12146	+	Putative lysophospholipase	0.300
PF00596	+	Class II Aldolase and Adducin N-terminal domain	0.295
PF01583	+	Adenylylsulphate kinase	0.292
PF02634	+	FdhD/NarQ family	0.291
PF02574	+	Homocysteine S-methyltransferase	0.278
PF05899	+	Protein of unknown function (DUF861)	0.262
PF00041	+	Fibronectin type III domain	0.257
PF06347	+	Bacterial SH3 domain	0.256
PF01914	-	MarC family integral membrane protein	0.255
PF01841	+	Transglutaminase-like superfamily	0.251
PF00325	+	Bacterial regulatory proteins, crp family	0.236
PF13458	+	Periplasmic binding protein	0.228
PF13473	+	Cupredoxin-like domain	0.227
PF02806	+	Alpha amylase, C-terminal all-beta domain	0.224
PF13559	+	Domain of unknown function (DUF4129)	0.220
PF10087	+	Uncharacterized protein conserved in bacteria (DUF2325)	0.220
PF10009	+	Uncharacterized protein conserved in bacteria (DUF2252)	0.216
PF13278	+	Putative amidotransferase	0.214
PF01450	+	Acetohydroxy acid isomeroreductase, catalytic domain	0.211
PF13089	+	Polyphosphate kinase N-terminal domain	0.207
PF02311	+	AraC-like ligand binding domain	0.205
PF14552	+	Tautomerase enzyme	0.202
PF02592	+	Uncharacterized ACR, YhhQ family COG1738	0.198
PF00920	+	Dehydratase family	0.196
PF13593	-	SBF-like CPA transporter family (DUF4137)	0.195
PF01758	-	Sodium Bile acid symporter family	0.195
PF03706	+	Uncharacterised protein family (UPF0104)	0.189
PF14534	-	Domain of unknown function (DUF4440)	0.188
PF13449	-	Esterase-like activity of phytase	0.185
PF06245	+	Protein of unknown function (DUF1015)	0.180
PF02559	+	CarD-like/TRCF domain	0.180
PF13379	-	NMT1-like family	0.178
PF13620	+	Carboxypeptidase regulatory-like domain	0.177

PF00984	-	UDP-glucose/GDP-mannose dehydrogenase family, central domain	0.172
PF02230	+	Phospholipase/Carboxylesterase	0.172
PF07331	-	Tripartite tricarboxylate transporter TctB family	0.172
PF04715	-	Anthranilate synthase component I, N terminal region	0.167
PF13581	-	Histidine kinase-like ATPase domain	0.165
PF00370	+	FGGY family of carbohydrate kinases, N-terminal domain	0.163
PF12680	-	SnoaL-like domain	0.162
PF03575	+	Peptidase family S51	0.159
PF13086	-	AAA domain	0.151
PF01293	+	Phosphoenolpyruvate carboxykinase	0.147
PF00079	+	Serpin (serine protease inhibitor)	0.147
PF04228	-	Putative neutral zinc metallopeptidase	0.146
PF02620	+	Uncharacterized ACR, COG1399	0.141
PF04264	-	YceI-like domain	0.131
PF01408	+	Oxidoreductase family, NAD-binding Rossmann fold	0.128
PF01094	+	Receptor family ligand binding region	0.127
PF02643	+	Uncharacterized ACR, COG1430	0.124
PF01208	-	Uroporphyrinogen decarboxylase (URO-D)	0.123
PF03976	-	Polyphosphate kinase 2 (PPK2)	0.121
PF05662	+	Coiled stalk of trimeric autotransporter adhesin	0.116
PF04257	-	Exodeoxyribonuclease V, gamma subunit	0.115
PF13558	-	Putative exonuclease SbcCD, C subunit	0.114
PF13704	-	Glycosyl transferase family 2	0.109
PF08450	-	SMP-30/Gluconolaconase/LRE-like region	0.106
PF00585	-	C-terminal regulatory domain of Threonine dehydratase	0.100
PF00081	+	Iron/manganese superoxide dismutases, alpha-hairpin domain	0.100
PF02777	+	Iron/manganese superoxide dismutases, C-terminal domain	0.100
PF01693	+	Caulimovirus viroplasmin	0.100
PF03819	-	MazG nucleotide pyrophosphohydrolase domain	0.099
PF02325	+	YGGT family	0.095
PF07745	-	Glycosyl hydrolase family 53	0.095
PF09348	+	Domain of unknown function (DUF1990)	0.094
PF07077	-	Protein of unknown function (DUF1345)	0.089
PF04536	-	TLP18.3, Psb32 and MOLO-1 founding proteins of phosphatase	0.083
PF05673	-	Protein of unknown function (DUF815)	0.077
PF03812	-	2-keto-3-deoxygluconate permease	0.070
PF13410	-	Glutathione S-transferase, C-terminal domain	0.069
PF07179	-	SseB protein N-terminal domain	0.068
PF07470	-	Glycosyl Hydrolase Family 88	0.066
PF14489	-	QueF-like protein	0.064
PF13409	-	Glutathione S-transferase, N-terminal domain	0.063
PF00501	+	AMP-binding enzyme	0.063
PF02447	-	GntP family permease	0.062
PF13599	-	Pentapeptide repeats (9 copies)	0.059
PF10551	-	MULE transposase domain	0.055
PF01741	+	Large-conductance mechanosensitive channel, MscL	0.052
PF01371	+	Trp repressor protein	0.050
PF07694	+	5TMR of 5TMR-LYT	0.047

PF01595	+	Domain of unknown function DUF21	0.047
PF03713	-	Domain of unknown function (DUF305)	0.046
PF12229	+	Putative peptidoglycan binding domain	0.044
PF01443	-	Viral (Superfamily 1) RNA helicase	0.035
PF01883	+	Domain of unknown function DUF59	0.030
PF00850	-	Histone deacetylase domain	0.028
PF13407	+	Periplasmic binding protein domain	0.027
PF09669	-	Phage regulatory protein Rha (Phage_pRha)	0.027
PF00970	-	Oxidoreductase FAD-binding domain	0.024
PF00194	-	Eukaryotic-type carbonic anhydrase	0.023
PF02958	-	Ecdysteroid kinase	0.023
PF03969	-	AFG1-like ATPase	0.021
PF13396	+	Phospholipase_D-nuclease N-terminal	0.019
PF03747	-	ADP-ribosylglycohydrolase	0.015
PF13587	-	N-terminal domain of DJ-1_PfpI family	0.013
PF02885	-	Glycosyl transferase family, helical bundle domain	0.007
PF09375	-	Imelysin	0.002
PF13241	-	Putative NAD(P)-binding	0.002
PF01844	-	HNH endonuclease	-0.013
PF04085	+	rod shape-determining protein MreC	-0.017
PF14716	+	Helix-hairpin-helix domain	-0.019
PF00657	-	GDSL-like Lipase/Acylhydrolase	-0.020
PF02583	-	Metal-sensitive transcriptional repressor	-0.029
PF01503	-	Phosphoribosyl-ATP pyrophosphohydrolase	-0.030
PF13454	-	FAD-NAD(P)-binding	-0.036
PF06166	-	Protein of unknown function (DUF979)	-0.046
PF01680	+	SOR/SNZ family	-0.048
PF01717	-	Cobalamin-independent synthase, Catalytic domain	-0.049
PF04978	-	Protein of unknown function (DUF664)	-0.052
PF01946	-	Thi4 family	-0.053
PF07831	-	Pyrimidine nucleoside phosphorylase C-terminal domain	-0.055
PF03668	+	P-loop ATPase protein family	-0.057
PF03060	+	Nitronate monooxygenase	-0.057
PF03788	-	LrgA family	-0.060
PF13346	+	ABC-2 family transporter protein	-0.060
PF13521	-	AAA domain	-0.062
PF09335	-	SNARE associated Golgi protein	-0.070
PF04131	+	Putative N-acetylmannosamine-6-phosphate epimerase	-0.072
PF00467	-	KOW motif	-0.072
PF07022	-	Bacteriophage CI repressor helix-turn-helix domain	-0.073
PF08211	-	Cytidine and deoxycytidylate deaminase zinc-binding region	-0.081
PF07508	-	Recombinase	-0.091
PF05708	-	Orthopoxvirus protein of unknown function (DUF830)	-0.092
PF03328	-	HpcH/HpaI aldolase/citrate lyase family	-0.097
PF01625	-	Peptide methionine sulfoxide reductase	-0.101
PF13530	-	Sterol carrier protein domain	-0.101
PF09991	-	Predicted membrane protein (DUF2232)	-0.103
PF05866	-	Endodeoxyribonuclease RusA	-0.105

PF00994	-	Probable molybdopterin binding domain	-0.111
PF03979	-	Sigma-70 factor, region 1.1	-0.114
PF10672	-	S-adenosylmethionine-dependent methyltransferase	-0.118
PF03390	-	2-hydroxycarboxylate transporter family	-0.119
PF13434	-	L-lysine 6-monooxygenase (NADPH-requiring)	-0.128
PF13669	-	Glyoxalase/Bleomycin resistance protein/Dioxygenase superfamily	-0.128
PF08020	-	Protein of unknown function (DUF1706)	-0.132
PF04883	-	Bacteriophage HK97-gp10, putative tail-component	-0.136
PF01235	-	Sodium:alanine symporter family	-0.144
PF01470	-	Pyroglutamyl peptidase	-0.147
PF11798	-	IMS family HHH motif	-0.147
PF13791	-	Sigma factor regulator C-terminal	-0.159
PF01076	-	Plasmid recombination enzyme	-0.190
PF02557	-	D-alanyl-D-alanine carboxypeptidase	-0.192
PF03352	-	Methyladenine glycosylase	-0.197
PF07085	-	DRTGG domain	-0.199
PF01745	+	Isopentenyl transferase	-0.225
PF02255	-	PTS system, Lactose/Cellobiose specific IIA subunit	-0.247
PF04468	-	PSP1 C-terminal conserved region	-0.265
PF09314	-	Domain of unknown function (DUF1972)	-0.272
PF05991	-	YacP-like NYN domain	-0.292
PF04296	+	Protein of unknown function (DUF448)	-0.294
PF13370	-	4Fe-4S single cluster domain	-0.295
PF09587	-	Bacterial capsule synthesis protein PGA_cap	-0.305
PF12892	-	T surface-antigen of pili	-0.318
PF02073	-	Thermophilic metalloprotease (M29)	-0.319
PF07155	-	ECF-type riboflavin transporter, S component	-0.320
PF02616	-	ScpA/B protein	-0.333
PF08503	+	Tetrahydrodipicolinate succinyltransferase N-terminal	-0.339
PF12642	-	Conjugative transposon protein TcpC	-0.340
PF08535	-	KorB domain	-0.341
PF07476	-	Methylaspartate ammonia-lyase C-terminus	-0.346
PF04079	-	Putative transcriptional regulators (Ypuh-like)	-0.349
PF04018	-	Domain of unknown function (DUF368)	-0.367
PF06737	-	Transglycosylase-like domain	-0.432
PF15432	+	Accessory Sec secretory system ASP3	-0.461
PF07949	-	YbbR-like protein	-0.462
PF02229	-	Transcriptional Coactivator p15 (PC4)	-0.468
PF10031	-	Small integral membrane protein (DUF2273)	-0.532
PF01487	-	Type I 3-dehydroquinase	-0.561
PF02388	-	FemAB family	-0.570
PF06279	-	Protein of unknown function (DUF1033)	-0.589

Phenotype: Coccus (Morphology), Predictor: phypat

Pfam	class	Pfam_desc	cor
PF11858	+	Domain of unknown function (DUF3378)	0.564
PF10031	+	Small integral membrane protein (DUF2273)	0.564
PF06962	+	Putative rRNA methylase	0.558

PF01487	+	Type I 3-dehydroquinase	0.543
PF02388	+	FemAB family	0.538
PF06949	+	Protein of unknown function (DUF1292)	0.499
PF02229	+	Transcriptional Coactivator p15 (PC4)	0.419
PF08535	+	KorB domain	0.356
PF10552	+	ORF6C domain	0.332
PF09587	+	Bacterial capsule synthesis protein PGA_cap	0.290
PF07085	+	DRTGG domain	0.263
PF03352	+	Methyladenine glycosylase	0.242
PF02302	+	PTS system, Lactose/Cellobiose specific IIB subunit	0.237
PF01226	+	Formate/nitrite transporter	0.208
PF02557	+	D-alanyl-D-alanine carboxypeptidase	0.194
PF05525	-	Branched-chain amino acid transport protein	0.187
PF08000	+	Bacterial PH domain	0.165
PF13434	+	L-lysine 6-monooxygenase (NADPH-requiring)	0.157
PF13669	+	Glyoxalase/Bleomycin resistance protein/Dioxygenase superfamily	0.154
PF08367	+	Peptidase M16C associated	0.149
PF05065	+	Phage capsid family	0.113
PF04131	-	Putative N-acetylmannosamine-6-phosphate epimerase	0.108
PF13530	+	Sterol carrier protein domain	0.098
PF13468	+	Glyoxalase-like domain	0.080
PF03848	+	Tellurite resistance protein TehB	0.077
PF03577	-	Peptidase family C69	0.077
PF13454	+	FAD-NAD(P)-binding	0.061
PF00657	+	GDSL-like Lipase/Acylhydrolase	0.046
PF04266	-	ASCH domain	0.039
PF01814	+	Hemerythrin HHE cation binding domain	0.035
PF01946	+	Thi4 family	0.035
PF00970	+	Oxidoreductase FAD-binding domain	0.030
PF01769	+	Divalent cation transporter	0.028
PF03969	+	AFG1-like ATPase	0.018
PF08713	-	DNA alkylation repair enzyme	0.003
PF01867	-	CRISPR associated protein Cas1	-0.005
PF10117	+	McrBC 5-methylcytosine restriction system component	-0.007
PF00850	+	Histone deacetylase domain	-0.010
PF13409	+	Glutathione S-transferase, N-terminal domain	-0.027
PF00082	-	Subtilase family	-0.037
PF02028	+	BCCT family transporter	-0.047
PF04239	-	Protein of unknown function (DUF421)	-0.054
PF13455	-	Meiotically up-regulated gene 113	-0.059
PF04326	-	Divergent AAA domain	-0.063
PF01206	+	Sulfurtransferase TusA	-0.085
PF13704	+	Glycosyl transferase family 2	-0.088
PF01590	-	GAF domain	-0.097
PF01208	+	Uroporphyrinogen decarboxylase (URO-D)	-0.114
PF01175	+	Urocanase	-0.115
PF01661	-	Macro domain	-0.134
PF08323	-	Starch synthase catalytic domain	-0.142

PF09704	-	CRISPR-associated protein (Cas_Cas5)	-0.146
PF10704	-	Protein of unknown function (DUF2508)	-0.148
PF02782	-	FGGY family of carbohydrate kinases, C-terminal domain	-0.149
PF03706	-	Uncharacterised protein family (UPF0104)	-0.159
PF04389	+	Peptidase family M28	-0.162
PF03473	+	MOSC domain	-0.163
PF14552	-	Tautomerase enzyme	-0.169
PF04461	+	Protein of unknown function (DUF520)	-0.180
PF03959	-	Serine hydrolase (FSH1)	-0.185
PF13473	-	Cupredoxin-like domain	-0.187
PF13348	-	Tyrosine phosphatase family C-terminal region	-0.189
PF10543	-	ORF6N domain	-0.189
PF03576	-	Peptidase family S58	-0.218
PF13278	-	Putative amidotransferase	-0.222
PF13746	+	4Fe-4S dicluster domain	-0.231
PF02870	-	6-O-methylguanine DNA methyltransferase, ribonuclease-like domain	-0.248
PF02574	-	Homocysteine S-methyltransferase	-0.258
PF12481	-	Aluminium induced protein	-0.277
PF12146	-	Putative lysophospholipase	-0.295
PF00041	-	Fibronectin type III domain	-0.307
PF00596	-	Class II Aldolase and Adducin N-terminal domain	-0.311
PF13464	-	Domain of unknown function (DUF4115)	-0.338
PF01923	-	Cobalamin adenosyltransferase	-0.347
PF00227	-	Proteasome subunit	-0.349
PF01882	-	Protein of unknown function DUF58	-0.359
PF01520	-	N-acetylmuramoyl-L-alanine amidase	-0.369
PF13524	-	Glycosyl transferases group 1	-0.369
PF00022	-	Actin	-0.378
PF02277	-	Phosphoribosyltransferase	-0.395
PF03775	-	Septum formation inhibitor MinC, C-terminal domain	-0.398
PF13231	-	Dolichyl-phosphate-mannose-protein mannosyltransferase	-0.399
PF13286	-	Phosphohydrolase-associated domain	-0.399
PF13144	-	SAF-like	-0.405
PF13432	-	Tetratricopeptide repeat	-0.408
PF01188	-	Mandelate racemase / muconate lactonizing enzyme, C-terminal domain	-0.419
PF13899	-	Thioredoxin-like	-0.431
PF00015	-	Methyl-accepting chemotaxis protein (MCP) signalling domain	-0.438
PF08345	-	Flagellar M-ring protein C-terminal	-0.442
PF00733	-	Asparagine synthase	-0.451
PF03963	-	Flagellar hook capping protein - N-terminal region	-0.459
PF01627	-	Hpt domain	-0.462
PF00183	-	Hsp90 protein	-0.479
PF07638	-	ECF sigma factor	-0.487
PF03880	-	DbpA RNA binding domain	-0.497
PF13292	-	1-deoxy-D-xylulose-5-phosphate synthase	-0.499
PF14532	-	Sigma-54 interaction domain	-0.515
PF01220	-	Dehydroquinase class II	-0.545

Phenotype: Coccus (Morphology), Predictor: phypat+PGL

Pfam	class	Pfam_desc	cor
PF06279	+	Protein of unknown function (DUF1033)	0.630
PF08820	+	Domain of unknown function (DUF1803)	0.614
PF10031	+	Small integral membrane protein (DUF2273)	0.564
PF04260	+	Protein of unknown function (DUF436)	0.558
PF06962	+	Putative rRNA methylase	0.558
PF11217	+	Protein of unknown function (DUF3013)	0.551
PF01487	+	Type I 3-dehydroquinase	0.543
PF02388	+	FemAB family	0.538
PF06949	+	Protein of unknown function (DUF1292)	0.499
PF08866	+	Putative amino acid metabolism	0.494
PF15432	+	Accessory Sec secretory system ASP3	0.492
PF06737	+	Transglycosylase-like domain	0.471
PF09419	+	Mitochondrial PGP phosphatase	0.457
PF03596	+	Cadmium resistance transporter	0.456
PF06177	+	QueT transporter	0.442
PF08353	+	Domain of unknown function (DUF1727)	0.438
PF06115	+	Domain of unknown function (DUF956)	0.428
PF02229	+	Transcriptional Coactivator p15 (PC4)	0.419
PF02486	+	Replication initiation factor	0.409
PF08363	+	Glucan-binding protein C	0.403
PF09223	+	YodA lipocalin-like domain	0.400
PF05257	+	CHAP domain	0.398
PF05816	+	Toxic anion resistance protein (TelA)	0.366
PF08503	+	Tetrahydrodipicolinate succinyltransferase N-terminal	0.359
PF08535	+	KorB domain	0.356
PF10552	+	ORF6C domain	0.332
PF08708	+	Primase C terminal 1 (PriCT-1)	0.309
PF14131	+	Domain of unknown function (DUF4298)	0.288
PF02677	+	Uncharacterized BCR, COG1636	0.280
PF02686	+	Glu-tRNAGln amidotransferase C subunit	0.277
PF06961	+	Protein of unknown function (DUF1294)	0.258
PF03352	+	Methyladenine glycosylase	0.242
PF00781	+	Diacylglycerol kinase catalytic domain	0.240
PF10576	+	Iron-sulfur binding domain of endonuclease III	0.239
PF06044	+	Dam-replacing family	0.231
PF01910	+	Domain of unknown function DUF77	0.229
PF06100	+	Streptococcal 67 kDa myosin-cross-reactive antigen like family	0.217
PF04392	+	ABC transporter substrate binding protein	0.215
PF15569	+	Immunity protein 21	0.194
PF12358	+	Protein of unknown function (DUF3644)	0.186
PF04306	+	Protein of unknown function (DUF456)	0.179
PF03610	-	PTS system fructose IIA component	0.160
PF04226	+	Transglycosylase associated protein	0.158
PF07532	+	Bacterial Ig-like domain (group 4)	0.154
PF08367	+	Peptidase M16C associated	0.149
PF07523	+	Bacterial Ig-like domain (group 3)	0.143

PF00413	-	Matrixin	0.142
PF01515	+	Phosphate acetyl/butaryl transferase	0.140
PF10989	+	Protein of unknown function (DUF2808)	0.135
PF09557	+	Domain of unknown function (DUF2382)	0.126
PF01633	-	Choline/ethanolamine kinase	0.122
PF08797	+	HIRAN domain	0.122
PF13556	+	PucR C-terminal helix-turn-helix domain	0.115
PF06810	-	Phage minor structural protein GP20	0.115
PF02498	+	BRO family, N-terminal domain	0.104
PF02092	+	Glycyl-tRNA synthetase beta subunit	0.099
PF14133	-	Domain of unknown function (DUF4300)	0.085
PF11840	+	Protein of unknown function (DUF3360)	0.083
PF13540	+	Regulator of chromosome condensation (RCC1) repeat	0.064
PF13630	-	SdpI/YhfL protein family	0.043
PF13610	-	DDE domain	0.033
PF01769	+	Divalent cation transporter	0.028
PF02744	-	Galactose-1-phosphate uridyl transferase, C-terminal domain	0.018
PF03592	-	Terminase small subunit	0.017
PF13338	+	Domain of unknown function (DUF4095)	0.017
PF10923	+	P-loop Domain of unknown function (DUF2791)	0.006
PF06838	-	Methionine gamma-lyase	0.005
PF08713	-	DNA alkylation repair enzyme	0.003
PF07694	+	5TMR of 5TMR-LYT	-0.001
PF00201	-	UDP-glucoronosyl and UDP-glucosyl transferase	-0.002
PF00850	+	Histone deacetylase domain	-0.010
PF13707	+	RloB-like protein	-0.012
PF13240	-	zinc-ribbon domain	-0.014
PF08818	-	Domain of unknown function (DU1801)	-0.019
PF03382	+	Mycoplasma protein of unknown function, DUF285	-0.019
PF10026	-	Predicted Zn-dependent protease (DUF2268)	-0.020
PF02646	+	RmuC family	-0.021
PF02958	-	Ecdysteroid kinase	-0.025
PF13409	+	Glutathione S-transferase, N-terminal domain	-0.027
PF08843	+	Nucleotidyl transferase of unknown function (DUF1814)	-0.039
PF12895	-	Anaphase-promoting complex, cyclosome, subunit 3	-0.049
PF00078	-	Reverse transcriptase (RNA-dependent DNA polymerase)	-0.049
PF10509	-	Galactokinase galactose-binding signature	-0.050
PF13114	-	RecO N terminal	-0.050
PF00872	-	Transposase, Mutator family	-0.054
PF13455	-	Meiotically up-regulated gene 113	-0.059
PF05223	-	NTF2-like N-terminal transpeptidase domain	-0.060
PF04326	-	Divergent AAA domain	-0.063
PF12102	-	Domain of unknown function (DUF3578)	-0.065
PF05658	+	Head domain of trimeric autotransporter adhesin	-0.071
PF03237	-	Terminase-like family	-0.081
PF01968	+	Hydantoinase/oxoprolinase	-0.082
PF05662	+	Coiled stalk of trimeric autotransporter adhesin	-0.087
PF11213	+	Protein of unknown function (DUF3006)	-0.088

PF13551	+	Winged helix-turn helix	-0.092
PF13018	+	Extended Signal Peptide of Type V secretion system	-0.097
PF03895	+	YadA-like C-terminal region	-0.100
PF07866	+	Protein of unknown function (DUF1653)	-0.100
PF03746	+	LamB/YcsF family	-0.105
PF05128	+	Domain of unknown function (DUF697)	-0.109
PF03808	-	Glycosyl transferase WecB/TagA/CpsF family	-0.110
PF13588	+	Type I restriction enzyme R protein N terminus (HSDR_N)	-0.112
PF01293	+	Phosphoenolpyruvate carboxykinase	-0.116
PF13490	+	Putative zinc-finger	-0.119
PF09723	+	Zinc ribbon domain	-0.121
PF03629	-	Domain of unknown function (DUF303)	-0.134
PF03956	+	Membrane protein of unknown function (DUF340)	-0.136
PF01960	-	ArgJ family	-0.139
PF00931	-	NB-ARC domain	-0.140
PF06414	-	Zeta toxin	-0.143
PF13088	-	BNR repeat-like domain	-0.144
PF00652	-	Ricin-type beta-trefoil lectin domain	-0.144
PF11734	-	TilS substrate C-terminal domain	-0.144
PF04143	+	Sulphur transport	-0.147
PF10728	+	Domain of unknown function (DUF2520)	-0.149
PF08463	-	EcoEI R protein C-terminal	-0.149
PF13470	+	PIN domain	-0.150
PF08924	-	Domain of unknown function (DUF1906)	-0.154
PF00697	-	N-(5'phosphoribosyl)anthranilate (PRA) isomerase	-0.163
PF03733	-	Domain of unknown function (DUF307)	-0.164
PF13379	-	NMT1-like family	-0.165
PF13342	-	C-terminal repeat of topoisomerase	-0.167
PF06940	-	Domain of unknown function (DUF1287)	-0.172
PF01758	-	Sodium Bile acid symporter family	-0.173
PF00325	-	Bacterial regulatory proteins, crp family	-0.177
PF04461	+	Protein of unknown function (DUF520)	-0.180
PF01315	+	Aldehyde oxidase and xanthine dehydrogenase, a/b hammerhead domain	-0.181
PF11975	-	Family 4 glycosyl hydrolase C-terminal domain	-0.181
PF13089	+	Polyphosphate kinase N-terminal domain	-0.184
PF13348	-	Tyrosine phosphatase family C-terminal region	-0.189
PF12974	-	ABC transporter, phosphonate, periplasmic substrate-binding protein	-0.191
PF08031	-	Berberine and berberine like	-0.201
PF12796	+	Ankyrin repeats (3 copies)	-0.203
PF08532	-	Beta-galactosidase trimerisation domain	-0.206
PF04235	-	Protein of unknown function (DUF418)	-0.206
PF08719	-	Domain of unknown function (DUF1768)	-0.209
PF13807	-	G-rich domain on putative tyrosine kinase	-0.218
PF02086	-	D12 class N6 adenine-specific DNA methyltransferase	-0.219
PF08443	-	RimK-like ATP-grasp domain	-0.223
PF07411	-	Domain of unknown function (DUF1508)	-0.226
PF01270	-	Glycosyl hydrolases family 8	-0.226
PF06226	-	Protein of unknown function (DUF1007)	-0.229

PF01914	-	MarC family integral membrane protein	-0.236
PF11127	+	Protein of unknown function (DUF2892)	-0.236
PF12571	-	Phage tail-collar fibre protein	-0.236
PF02348	+	Cytidylyltransferase	-0.237
PF02574	-	Homocysteine S-methyltransferase	-0.258
PF10101	-	Predicted membrane protein (DUF2339)	-0.259
PF01490	-	Transmembrane amino acid transporter protein	-0.261
PF00782	-	Dual specificity phosphatase, catalytic domain	-0.266
PF11638	-	DnaA N-terminal domain	-0.268
PF00933	-	Glycosyl hydrolase family 3 N terminal domain	-0.269
PF00092	-	von Willebrand factor type A domain	-0.289
PF05954	-	Phage late control gene D protein (GPD)	-0.301
PF00596	-	Class II Aldolase and Adducin N-terminal domain	-0.311
PF13360	+	PQQ-like domain	-0.313
PF01923	-	Cobalamin adenosyltransferase	-0.347
PF00202	-	Aminotransferase class-III	-0.348
PF00227	-	Proteasome subunit	-0.349
PF02599	-	Global regulator protein family	-0.349
PF09312	-	SurA N-terminal domain	-0.357
PF13533	-	Biotin-lipoyl like	-0.359
PF13524	-	Glycosyl transferases group 1	-0.369
PF05130	-	FlgN protein	-0.374
PF01339	-	CheB methylesterase	-0.385
PF00111	+	2Fe-2S iron-sulfur cluster binding domain	-0.390
PF00563	-	EAL domain	-0.396
PF03775	+	Septum formation inhibitor MinC, C-terminal domain	-0.398
PF13432	+	Tetratricopeptide repeat	-0.408
PF02120	-	Flagellar hook-length control protein FliK	-0.446
PF00733	-	Asparagine synthase	-0.451

Phenotype: Coccus - clusters or groups predominate (Morphology), Predictor: phypat

Pfam	class	Pfam_desc	cor
PF13038	+	Domain of unknown function (DUF3899)	0.493
PF06737	+	Transglycosylase-like domain	0.472
PF04306	+	Protein of unknown function (DUF456)	0.457
PF15432	+	Accessory Sec secretory system ASP3	0.455
PF01910	+	Domain of unknown function DUF77	0.359
PF06874	+	Firmicute fructose-1,6-bisphosphatase	0.303
PF02423	+	Ornithine cyclodeaminase/mu-crystallin family	0.249
PF14542	+	GCN5-related N-acetyl-transferase	0.247
PF14690	+	zinc-finger of transposase IS204/IS1001/IS1096/IS1165	0.225
PF04389	+	Peptidase family M28	0.200
PF08392	+	FAE1/Type III polyketide synthase-like protein	0.056
PF01867	-	CRISPR associated protein Cas1	-0.092
PF01590	-	GAF domain	-0.097
PF13533	-	Biotin-lipoyl like	-0.108
PF09704	-	CRISPR-associated protein (Cas_Cas5)	-0.152
PF13437	-	HlyD family secretion protein	-0.173

PF00793	-	DAHP synthetase I family	-0.178
PF08713	-	DNA alkylation repair enzyme	-0.188
PF13192	-	Thioredoxin domain	-0.190
PF12738	-	twin BRCT domain	-0.195
PF12169	-	DNA polymerase III subunits gamma and tau domain III	-0.207
PF12897	-	Alanine-glyoxylate amino-transferase	-0.218
PF13740	-	ACT domain	-0.233
PF04608	-	Phosphatidylglycerophosphatase A	-0.245
PF00908	-	dTDP-4-dehydrorhamnose 3,5-epimerase	-0.257
PF07638	-	ECF sigma factor	-0.264
PF00022	-	Actin	-0.271
PF00933	-	Glycosyl hydrolase family 3 N terminal domain	-0.276
PF10417	-	C-terminal domain of 1-Cys peroxiredoxin	-0.276
PF07943	-	Penicillin-binding protein 5, C-terminal domain	-0.290
PF13231	-	Dolichyl-phosphate-mannose-protein mannosyltransferase	-0.293
PF13386	-	Cytochrome C biogenesis protein transmembrane region	-0.295
PF03279	-	Bacterial lipid A biosynthesis acyltransferase	-0.298
PF02666	-	Phosphatidylserine decarboxylase	-0.310
PF13519	-	von Willebrand factor type A domain	-0.318
PF01227	-	GTP cyclohydrolase I	-0.323
PF02190	-	ATP-dependent protease La (LON) domain	-0.339
PF00654	-	Voltage gated chloride channel	-0.364
PF13292	-	1-deoxy-D-xylulose-5-phosphate synthase	-0.440

Phenotype: Coccus - clusters or groups predominate (Morphology), Predictor: phypat+PGL

Pfam	class	Pfam_desc	cor
PF10425	+	C-terminus of bacterial fibrinogen-binding adhesin	0.559
PF13038	+	Domain of unknown function (DUF3899)	0.493
PF06737	+	Transglycosylase-like domain	0.472
PF04306	+	Protein of unknown function (DUF456)	0.457
PF15432	+	Accessory Sec secretory system ASP3	0.455
PF05495	+	CHY zinc finger	0.429
PF01910	+	Domain of unknown function DUF77	0.359
PF09685	+	Tic20-like protein	0.346
PF06335	+	Protein of unknown function (DUF1054)	0.312
PF14145	+	YrhK-like protein	0.279
PF11192	+	Protein of unknown function (DUF2977)	0.267
PF09524	+	Conserved phage C-terminus (Phg_2220_C)	0.236
PF09557	+	Domain of unknown function (DUF2382)	0.210
PF07274	+	Protein of unknown function (DUF1440)	0.206
PF06769	+	Plasmid encoded toxin Txe	0.202
PF04389	+	Peptidase family M28	0.200
PF13370	-	4Fe-4S single cluster domain	0.156
PF06486	+	Protein of unknown function (DUF1093)	0.147
PF02770	+	Acyl-CoA dehydrogenase, middle domain	0.144
PF01721	+	Class II bacteriocin	0.144
PF02578	+	Multi-copper polyphenol oxidoreductase laccase	0.093
PF01663	+	Type I phosphodiesterase / nucleotide pyrophosphatase	0.079

PF05569	-	BlaR1 peptidase M56	0.075
PF13087	+	AAA domain	0.058
PF02604	+	Antitoxin Phd_YefM, type II toxin-antitoxin system	0.054
PF07670	+	Nucleoside recognition	0.049
PF05223	-	NTF2-like N-terminal transpeptidase domain	0.040
PF10397	-	Adenylosuccinate lyase C-terminus	0.034
PF01864	+	Putative integral membrane protein DUF46	0.029
PF00585	-	C-terminal regulatory domain of Threonine dehydratase	0.023
PF10096	+	Uncharacterized protein conserved in bacteria (DUF2334)	0.019
PF10926	-	Protein of unknown function (DUF2800)	-0.009
PF04191	-	Phospholipid methyltransferase	-0.009
PF02553	+	Cobalt transport protein component CbiN	-0.015
PF09371	-	Tex-like protein N-terminal domain	-0.022
PF00081	-	Iron/manganese superoxide dismutases, alpha-hairpin domain	-0.034
PF02777	-	Iron/manganese superoxide dismutases, C-terminal domain	-0.034
PF03887	+	YfbU domain	-0.054
PF01590	-	GAF domain	-0.097
PF10418	-	Iron-sulfur cluster binding domain of dihydroorotate dehydrogenase B	-0.113
PF13088	-	BNR repeat-like domain	-0.119
PF05448	-	Acetyl xylan esterase (AXE1)	-0.119
PF02749	+	Quinolinate phosphoribosyl transferase, N-terminal domain	-0.129
PF07366	-	SnoaL-like polyketide cyclase	-0.143
PF14378	-	PAP2 superfamily	-0.143
PF03459	-	TOBE domain	-0.147
PF09704	-	CRISPR-associated protein (Cas_Cas5)	-0.152
PF03733	-	Domain of unknown function (DUF307)	-0.163
PF05973	-	Phage derived protein Gp49-like (DUF891)	-0.168
PF02446	-	4-alpha-glucanotransferase	-0.178
PF02277	-	Phosphoribosyltransferase	-0.209
PF13386	-	Cytochrome C biogenesis protein transmembrane region	-0.295

Phenotype: Coccus - pairs or chains predominate (Morphology), Predictor: phypat

Pfam	class	Pfam_desc	cor
PF08820	+	Domain of unknown function (DUF1803)	0.804
PF11983	+	Domain of unknown function (DUF3484)	0.735
PF02229	+	Transcriptional Coactivator p15 (PC4)	0.555
PF06177	+	QueT transporter	0.547
PF11858	+	Domain of unknown function (DUF3378)	0.541
PF01487	+	Type I 3-dehydroquinase	0.521
PF02486	+	Replication initiation factor	0.497
PF09314	+	Domain of unknown function (DUF1972)	0.459
PF10031	+	Small integral membrane protein (DUF2273)	0.427
PF06961	+	Protein of unknown function (DUF1294)	0.406
PF08535	+	KorB domain	0.349
PF04392	+	ABC transporter substrate binding protein	0.322
PF06044	+	Dam-replacing family	0.318
PF08274	+	PhnA Zinc-Ribbon	0.291
PF02616	+	ScpA/B protein	0.281

PF04991	+	LicD family	0.276
PF02091	+	Glycyl-tRNA synthetase alpha subunit	0.275
PF02092	+	Glycyl-tRNA synthetase beta subunit	0.275
PF14815	+	NUDIX domain	0.249
PF03352	+	Methyladenine glycosylase	0.221
PF03979	+	Sigma-70 factor, region 1.1	0.219
PF06827	+	Zinc finger found in FPG and IleRS	0.215
PF13669	+	Glyoxalase/Bleomycin resistance protein/Dioxygenase superfamily	0.210
PF02547	+	Queuosine biosynthesis protein	0.201
PF13343	+	Bacterial extracellular solute-binding protein	0.184
PF03328	+	HpcH/HpaI aldolase/citrate lyase family	0.184
PF01503	+	Phosphoribosyl-ATP pyrophosphohydrolase	0.179
PF00657	+	GDSL-like Lipase/Acylhydrolase	0.137
PF03590	-	Aspartate-ammonia ligase	0.104
PF01814	+	Hemerythrin HHE cation binding domain	0.041
PF05658	+	Head domain of trimeric autotransporter adhesin	0.022
PF08479	+	POTRA domain, ShlB-type	0.010
PF04608	+	Phosphatidylglycerophosphatase A	0.009
PF03895	+	YadA-like C-terminal region	0.005
PF05673	+	Protein of unknown function (DUF815)	0.003
PF01791	-	DeoC/LacD family aldolase	-0.029
PF13173	-	AAA domain	-0.030
PF01680	-	SOR/SNZ family	-0.111
PF02325	-	YGGT family	-0.152
PF03009	-	Glycerophosphoryl diester phosphodiesterase family	-0.158
PF00596	-	Class II Aldolase and Adducin N-terminal domain	-0.165
PF01661	-	Macro domain	-0.165
PF13350	-	Tyrosine phosphatase family	-0.194
PF02782	-	FGGY family of carbohydrate kinases, C-terminal domain	-0.225
PF00350	-	Dynamin family	-0.235
PF06559	-	2'-deoxycytidine 5'-triphosphate deaminase (DCD)	-0.261
PF13278	-	Putative amidotransferase	-0.275
PF05222	-	Alanine dehydrogenase/PNT, N-terminal domain	-0.280
PF01262	-	Alanine dehydrogenase/PNT, C-terminal domain	-0.285
PF10410	-	DnaB-helicase binding domain of primase	-0.287
PF05195	-	Aminopeptidase P, N-terminal domain	-0.336
PF07726	-	ATPase family associated with various cellular activities (AAA)	-0.345
PF01313	-	Bacterial export proteins, family 3	-0.346
PF06429	-	Flagellar basal body rod FlgEFG protein C-terminal	-0.349
PF00771	-	FHIPEP family	-0.349
PF01726	-	LexA DNA binding domain	-0.351
PF03963	-	Flagellar hook capping protein - N-terminal region	-0.352
PF08338	-	Domain of unknown function (DUF1731)	-0.366
PF00227	-	Proteasome subunit	-0.378
PF02634	-	FdhD/NarQ family	-0.380
PF13286	-	Phosphohydrolase-associated domain	-0.386
PF00510	-	Cytochrome c oxidase subunit III	-0.391
PF08028	-	Acyl-CoA dehydrogenase, C-terminal domain	-0.392

PF12146	-	Putative lysophospholipase	-0.393
PF06609	-	Fungal trichothecene efflux pump (TRI12)	-0.414
PF13607	-	Succinyl-CoA ligase like flavodoxin domain	-0.416
PF03880	-	DbpA RNA binding domain	-0.425
PF00883	-	Cytosol aminopeptidase family, catalytic domain	-0.427
PF13524	-	Glycosyl transferases group 1	-0.436
PF13432	-	Tetratricopeptide repeat	-0.446
PF03741	-	Integral membrane protein TerC family	-0.462
PF01220	-	Dehydroquinase class II	-0.488
PF07497	-	Rho termination factor, RNA-binding domain	-0.615

Phenotype: Coccus - pairs or chains predominate (Morphology), Predictor: phypat+PGL

Pfam	class	Pfam_desc	cor
PF08820	+	Domain of unknown function (DUF1803)	0.804
PF11217	+	Protein of unknown function (DUF3013)	0.735
PF11240	+	Protein of unknown function (DUF3042)	0.627
PF11184	+	Protein of unknown function (DUF2969)	0.601
PF02229	+	Transcriptional Coactivator p15 (PC4)	0.555
PF06177	+	QueT transporter	0.547
PF11858	+	Domain of unknown function (DUF3378)	0.541
PF04260	+	Protein of unknown function (DUF436)	0.522
PF01487	+	Type I 3-dehydroquinase	0.521
PF04525	+	Tubby C 2	0.520
PF02486	+	Replication initiation factor	0.497
PF06962	+	Putative rRNA methylase	0.480
PF03444	+	Winged helix-turn-helix transcription repressor, HrcA DNA-binding	0.463
PF09314	+	Domain of unknown function (DUF1972)	0.459
PF02829	+	3H domain	0.433
PF06961	+	Protein of unknown function (DUF1294)	0.406
PF02388	+	FemAB family	0.393
PF11694	+	Protein of unknown function (DUF3290)	0.362
PF14131	+	Domain of unknown function (DUF4298)	0.331
PF06044	+	Dam-replacing family	0.318
PF03062	+	MBOAT, membrane-bound O-acyltransferase family	0.300
PF01076	+	Plasmid recombination enzyme	0.286
PF02608	+	Basic membrane protein	0.270
PF15569	+	Immunity protein 21	0.270
PF10552	+	ORF6C domain	0.257
PF11283	+	Protein of unknown function (DUF3084)	0.253
PF03390	+	2-hydroxycarboxylate transporter family	0.250
PF02324	+	Glycosyl hydrolase family 70	0.219
PF04087	+	Domain of unknown function (DUF389)	0.218
PF04245	+	37-kD nucleoid-associated bacterial protein	0.190
PF03610	-	PTS system fructose IIA component	0.170
PF00367	-	phosphotransferase system, EIIB	0.162
PF11840	+	Protein of unknown function (DUF3360)	0.142
PF04830	+	Possible hemagglutinin (DUF637)	0.111
PF13515	+	Fusaric acid resistance protein-like	0.093

PF01238	-	Phosphomannose isomerase type I	0.090
PF11208	-	Protein of unknown function (DUF2992)	0.090
PF14393	+	Domain of unknown function (DUF4422)	0.089
PF02734	-	DAK2 domain	0.083
PF07669	-	Eco57I restriction-modification methylase	0.075
PF01633	-	Choline/ethanolamine kinase	0.074
PF08367	+	Peptidase M16C associated	0.065
PF06857	+	Malonate decarboxylase delta subunit (MdcD)	0.057
PF00485	-	Phosphoribulokinase / Uridine kinase family	0.055
PF03432	-	Relaxase/Mobilisation nuclease domain	0.031
PF03848	-	Tellurite resistance protein TehB	0.023
PF00078	-	Reverse transcriptase (RNA-dependent DNA polymerase)	0.022
PF05658	+	Head domain of trimeric autotransporter adhesin	0.022
PF00708	-	Acylphosphatase	0.013
PF03895	+	YadA-like C-terminal region	0.005
PF00188	+	Cysteine-rich secretory protein family	0.004
PF10412	-	Type IV secretion-system coupling protein DNA-binding domain	0.004
PF13018	+	Extended Signal Peptide of Type V secretion system	-0.002
PF12724	-	Flavodoxin domain	-0.008
PF07991	+	Acetohydroxy acid isomeroreductase, catalytic domain	-0.010
PF12696	-	TraM recognition site of TraD and TraG	-0.016
PF05690	+	Thiazole biosynthesis protein ThiG	-0.020
PF01427	+	D-ala-D-ala dipeptidase	-0.023
PF02629	-	CoA binding domain	-0.025
PF07179	-	SseB protein N-terminal domain	-0.027
PF01791	-	DeoC/LacD family aldolase	-0.029
PF13173	-	AAA domain	-0.030
PF03237	-	Terminase-like family	-0.042
PF01527	-	Transposase	-0.043
PF02872	-	5'-nucleotidase, C-terminal domain	-0.052
PF04326	-	Divergent AAA domain	-0.054
PF07866	+	Protein of unknown function (DUF1653)	-0.056
PF00239	-	Resolvase, N terminal domain	-0.056
PF14502	-	Helix-turn-helix domain	-0.060
PF05154	-	TM2 domain	-0.065
PF05437	-	Branched-chain amino acid transport protein (AzlD)	-0.065
PF00201	-	UDP-glucoronosyl and UDP-glucosyl transferase	-0.081
PF03649	-	Uncharacterised protein family (UPF0014)	-0.083
PF05128	+	Domain of unknown function (DUF697)	-0.092
PF02219	-	Methylenetetrahydrofolate reductase	-0.101
PF06414	-	Zeta toxin	-0.116
PF08392	-	FAE1/Type III polyketide synthase-like protein	-0.121
PF02447	-	GntP family permease	-0.138
PF02624	-	YcaO-like family	-0.141
PF00296	-	Luciferase-like monooxygenase	-0.141
PF10127	-	Predicted nucleotidyltransferase	-0.142
PF01270	-	Glycosyl hydrolases family 8	-0.150
PF07411	-	Domain of unknown function (DUF1508)	-0.150

PF02325	-	YGGT family	-0.152
PF05872	-	Bacterial protein of unknown function (DUF853)	-0.154
PF11398	-	Protein of unknown function (DUF2813)	-0.161
PF01661	-	Macro domain	-0.165
PF05164	-	Cell division protein ZapA	-0.182
PF13350	-	Tyrosine phosphatase family	-0.194
PF01243	-	Pyridoxamine 5'-phosphate oxidase	-0.211
PF00350	-	Dynamin family	-0.235
PF12974	-	ABC transporter, phosphonate, periplasmic substrate-binding protein	-0.257
PF08443	-	RimK-like ATP-grasp domain	-0.273
PF00719	-	Inorganic pyrophosphatase	-0.299
PF11638	-	DnaA N-terminal domain	-0.300
PF03626	-	Prokaryotic Cytochrome C oxidase subunit IV	-0.311
PF03572	-	Peptidase family S41	-0.342
PF00011	-	Hsp20/alpha crystallin family	-0.354
PF01738	-	Dienelactone hydrolase family	-0.358
PF00227	-	Proteasome subunit	-0.378
PF01037	-	AsnC family	-0.387
PF12146	-	Putative lysophospholipase	-0.393
PF02597	-	ThiS family	-0.411
PF03454	-	MoeA C-terminal region (domain IV)	-0.418
PF13432	-	Tetratricopeptide repeat	-0.446
PF00075	-	RNase H	-0.603

Phenotype: Gram negative (Morphology), Predictor: phypat

Pfam	class	Pfam_desc	cor
PF01103	+	Surface antigen	0.983
PF13720	+	Udp N-acetylglucosamine O-acyltransferase; Domain 2	0.974
PF02472	+	Biopolymer transport protein ExbD/TolR	0.974
PF03331	+	UDP-3-O-acyl N-acetylglycosamine deacetylase	0.974
PF02321	+	Outer membrane efflux protein	0.948
PF13505	+	Outer membrane protein beta-barrel domain	0.939
PF04357	+	Family of unknown function (DUF490)	0.866
PF08645	+	Polynucleotide kinase 3 phosphatase	0.688
PF05683	+	Fumarase C-terminus	0.578
PF05860	+	haemagglutination activity domain	0.548
PF02091	+	Glycyl-tRNA synthetase alpha subunit	0.425
PF02417	+	Chromate transporter	0.336
PF12392	+	Collagenase	0.257
PF07498	-	Rho termination factor, N-terminal domain	0.241
PF02677	+	Uncharacterized BCR, COG1636	0.200
PF01925	+	Sulfite exporter TauE/SafE	0.174
PF09383	+	NIL domain	0.016
PF05437	+	Branched-chain amino acid transport protein (AzlD)	-0.013
PF00444	-	Ribosomal protein L36	-0.127
PF03449	-	Transcription elongation factor, N-terminal	-0.146
PF00211	-	Adenylate and Guanylate cyclase catalytic domain	-0.179
PF03009	-	Glycerophosphoryl diester phosphodiesterase family	-0.208

PF01182	-	Glucosamine-6-phosphate isomerases/6-phosphogluconolactonase	-0.228
PF01633	-	Choline/ethanolamine kinase	-0.320
PF07475	-	HPr Serine kinase C-terminal domain	-0.357
PF02603	-	HPr Serine kinase N terminus	-0.435
PF02388	-	FemAB family	-0.486
PF11838	-	ERAP1-like C-terminal domain	-0.607
PF13735	-	tRNA nucleotidyltransferase domain 2 putative	-0.674
PF00481	-	Protein phosphatase 2C	-0.761
PF04203	-	Sortase family	-0.857
PF03816	-	Cell envelope-related transcriptional attenuator domain	-0.868
PF05103	-	DivIVA protein	-0.878
PF14527	-	WhiA LAGLIDADG-like domain	-0.913

Phenotype: Gram negative (Morphology), Predictor: phypat+PGL

Pfam	class	Pfam_desc	cor
PF01103	+	Surface antigen	0.983
PF03331	+	UDP-3-O-acyl N-acetylglycosamine deacetylase	0.974
PF02472	+	Biopolymer transport protein ExbD/TolR	0.974
PF13720	+	Udp N-acetylglucosamine O-acyltransferase; Domain 2	0.974
PF04413	+	3-Deoxy-D-manno-octulosonic-acid transferase (kdotransferase)	0.965
PF02321	+	Outer membrane efflux protein	0.948
PF13505	+	Outer membrane protein beta-barrel domain	0.939

Phenotype: Gram positive (Morphology), Predictor: phypat

Pfam	class	Pfam_desc	cor
PF04203	+	Sortase family	0.938
PF10298	+	WhiA N-terminal LAGLIDADG-like domain	0.921
PF03816	+	Cell envelope-related transcriptional attenuator domain	0.907
PF04472	+	Protein of unknown function (DUF552)	0.904
PF05103	+	DivIVA protein	0.884
PF08353	+	Domain of unknown function (DUF1727)	0.815
PF03793	+	PASTA domain	0.777
PF12890	+	Dihydro-orotase-like	0.768
PF06257	+	Protein of unknown function (DUF1021)	0.729
PF12051	+	Protein of unknown function (DUF3533)	0.691
PF06971	+	Putative DNA-binding protein N-terminus	0.625
PF01183	+	Glycosyl hydrolases family 25	0.594
PF01680	+	SOR/SNZ family	0.579
PF01910	+	Domain of unknown function DUF77	0.571
PF02452	+	PemK-like protein	0.523
PF01174	+	SNO glutamine amidotransferase family	0.489
PF04138	+	GtrA-like protein	0.479
PF13783	+	Domain of unknown function (DUF4177)	0.398
PF13307	+	Helicase C-terminal domain	0.371
PF13556	+	PucR C-terminal helix-turn-helix domain	0.371
PF06738	+	Protein of unknown function (DUF1212)	0.356
PF12821	+	Protein of unknown function (DUF3815)	0.356
PF03951	+	Glutamine synthetase, beta-Grasp domain	0.250

PF03729	+	Short repeat of unknown function (DUF308)	0.205
PF02557	+	D-alanyl-D-alanine carboxypeptidase	0.195
PF13277	-	YmdB-like protein	0.070
PF09954	-	Uncharacterized protein conserved in bacteria (DUF2188)	0.068
PF01637	-	Archaeal ATPase	-0.042
PF01396	-	Topoisomerase DNA binding C4 zinc finger	-0.113
PF11074	-	Domain of unknown function(DUF2779)	-0.210
PF13195	-	Protein of unknown function (DUF4011)	-0.210
PF06415	-	BPG-independent PGAM N-terminus (iPGM_N)	-0.287
PF10396	-	GTP-binding protein TrmE N-terminus	-0.475
PF08298	-	PrkA AAA domain	-0.508
PF02190	-	ATP-dependent protease La (LON) domain	-0.754
PF04357	-	Family of unknown function (DUF490)	-0.816
PF07244	-	Surface antigen variable number repeat	-0.923
PF01103	-	Surface antigen	-0.940

Phenotype: Gram positive (Morphology), Predictor: phypat+PGL

Pfam	class	Pfam_desc	cor
PF04203	+	Sortase family	0.938
PF03816	+	Cell envelope-related transcriptional attenuator domain	0.907
PF04472	+	Protein of unknown function (DUF552)	0.904
PF05103	+	DivIVA protein	0.884
PF14527	+	WhiA LAGLIDADG-like domain	0.876
PF02650	+	WhiA C-terminal HTH domain	0.876
PF08353	+	Domain of unknown function (DUF1727)	0.815
PF02645	+	Uncharacterised protein, DegV family COG1307	0.772
PF06257	+	Protein of unknown function (DUF1021)	0.729
PF12051	+	Protein of unknown function (DUF3533)	0.691
PF14480	+	DNA polymerase III polC-type N-terminus I	0.683
PF11490	+	DNA polymerase III polC-type N-terminus II	0.675
PF11838	+	ERAP1-like C-terminal domain	0.639
PF01183	+	Glycosyl hydrolases family 25	0.594
PF07739	+	TipAS antibiotic-recognition domain	0.542
PF06737	+	Transglycosylase-like domain	0.529
PF06153	+	Protein of unknown function (DUF970)	0.506
PF03703	+	Bacterial PH domain	0.492
PF12401	+	Protein of unknown function (DUF2662)	0.465
PF10704	+	Protein of unknown function (DUF2508)	0.448
PF04417	+	Protein of unknown function (DUF501)	0.440
PF09754	+	PAC2 family	0.440
PF12029	+	Domain of unknown function (DUF3516)	0.440
PF13307	+	Helicase C-terminal domain	0.371
PF06738	+	Protein of unknown function (DUF1212)	0.356
PF07561	+	Domain of Unknown Function (DUF1540)	0.341
PF12685	+	SpoIIIAH-like protein	0.321
PF08769	+	Sporulation initiation factor Spo0A C terminal	0.310
PF09551	+	Stage II sporulation protein R (spore_II_R)	0.310
PF09581	+	Stage III sporulation protein AF (Spore_III_AF)	0.300

PF12652	+	CotJB protein	0.289
PF09548	+	Stage III sporulation protein AB (spore_III_AB)	0.289
PF03951	+	Glutamine synthetase, beta-Grasp domain	0.250
PF01544	+	CorA-like Mg2+ transporter protein	0.215
PF13636	+	pre-rRNA processing and ribosome biogenesis	0.127
PF03837	-	RecT family	0.075
PF09954	-	Uncharacterized protein conserved in bacteria (DUF2188)	0.068
PF02872	-	5'-nucleotidase, C-terminal domain	0.060
PF03932	-	CutC family	0.042
PF13274	-	Protein of unknown function (DUF4065)	-0.003
PF04471	-	Restriction endonuclease	-0.059
PF10117	-	McrBC 5-methylcytosine restriction system component	-0.081
PF09992	-	Predicted periplasmic protein (DUF2233)	-0.104
PF13424	-	Tetratricopeptide repeat	-0.127
PF01051	-	Initiator Replication protein	-0.134
PF11738	-	Protein of unknown function (DUF3298)	-0.185
PF01293	-	Phosphoenolpyruvate carboxykinase	-0.218
PF06414	-	Zeta toxin	-0.220
PF13336	-	Acetyl-CoA hydrolase/transferase C-terminal domain	-0.234
PF00023	-	Ankyrin repeat	-0.278
PF02091	-	Glycyl-tRNA synthetase alpha subunit	-0.301
PF03616	-	Sodium/glutamate symporter	-0.305
PF12399	-	Branched-chain amino acid ATP-binding cassette transporter	-0.307
PF05973	-	Phage derived protein Gp49-like (DUF891)	-0.336
PF14532	-	Sigma-54 interaction domain	-0.525
PF00873	-	AcrB/AcrD/AcrF family	-0.683
PF01618	-	MotA/TolQ/ExbB proton channel family	-0.750
PF03544	+	Gram-negative bacterial TonB protein C-terminal	-0.831

Phenotype: Motile (Morphology), Predictor: phypat

Pfam	class	Pfam_desc	cor
PF02154	+	Flagellar motor switch protein FliM	0.832
PF14842	+	FliG N-terminal domain	0.823
PF13677	+	Membrane MotB of proton-channel complex MotA/MotB	0.803
PF08345	+	Flagellar M-ring protein C-terminal	0.803
PF02561	+	Flagellar protein FliS	0.796
PF01739	+	CheR methyltransferase, SAM binding domain	0.734
PF08668	+	HDOD domain	0.569
PF02743	+	Cache domain	0.561
PF13429	+	Tetratricopeptide repeat	0.338
PF01116	-	Fructose-bisphosphate aldolase class-II	-0.047
PF02424	-	ApbE family	-0.094
PF00288	-	GHMP kinases N terminal domain	-0.096
PF00210	-	Ferritin-like domain	-0.098
PF02673	-	Bacitracin resistance protein BacA	-0.103
PF03606	-	C4-dicarboxylate anaerobic carrier	-0.112
PF03883	-	Protein of unknown function (DUF328)	-0.160
PF03668	-	P-loop ATPase protein family	-0.167

PF00871	-	Acetokinase family	-0.183
PF00392	-	Bacterial regulatory proteins, gntR family	-0.187
PF12836	-	Helix-hairpin-helix motif	-0.238
PF05116	-	Sucrose-6F-phosphate phosphohydrolase	-0.269
PF03119	-	NAD-dependent DNA ligase C4 zinc finger domain	-0.272
PF01183	-	Glycosyl hydrolases family 25	-0.459
PF08353	-	Domain of unknown function (DUF1727)	-0.498

Phenotype: Motile (Morphology), Predictor: phypat+PGL

Pfam	class	Pfam_desc	cor
PF13677	+	Membrane MotB of proton-channel complex MotA/MotB	0.803
PF03963	+	Flagellar hook capping protein - N-terminal region	0.802
PF02561	+	Flagellar protein FliS	0.796
PF02050	+	Flagellar FliJ protein	0.724
PF07559	+	Flagellar basal body protein FlaE	0.681
PF07238	+	PilZ domain	0.590
PF02743	+	Cache domain	0.561
PF08269	+	Cache domain	0.557
PF13682	+	Chemoreceptor zinc-binding domain	0.493
PF03350	+	Uncharacterized protein family, UPF0114	0.464
PF05226	+	CHASE2 domain	0.455
PF04116	+	Fatty acid hydroxylase superfamily	0.450
PF08666	+	SAF domain	0.449
PF07194	+	P2 response regulator binding domain	0.448
PF04982	+	HPP family	0.445
PF13103	+	TonB C terminal	0.440
PF03972	+	MmgE/PrpD family	0.417
PF08521	+	Two-component sensor kinase N-terminal	0.407
PF03401	+	Tripartite tricarboxylate transporter family receptor	0.405
PF09994	+	Uncharacterized alpha/beta hydrolase domain (DUF2235)	0.401
PF09976	+	Tetratricopeptide repeat	0.398
PF04187	+	Protein of unknown function, DUF399	0.379
PF01062	+	Bestrophin, RFP-TM, chloride channel	0.371
PF12860	+	PAS fold	0.365
PF05171	+	Haemin-degrading HemS.ChuX domain	0.356
PF06228	+	Haem utilisation ChuX/HutX	0.346
PF13429	+	Tetratricopeptide repeat	0.338
PF13637	+	Ankyrin repeats (many copies)	0.337
PF00141	-	Peroxidase	0.318
PF07642	+	Outer membrane protein family (DUF1597)	0.318
PF07719	+	Tetratricopeptide repeat	0.311
PF07120	+	Protein of unknown function (DUF1376)	0.311
PF03922	-	OmpW family	0.310
PF01878	+	EVE domain	0.308
PF09588	+	YqaJ-like viral recombinase domain	0.308
PF01904	+	Protein of unknown function DUF72	0.305
PF07804	-	HipA-like C-terminal domain	0.305
PF00174	-	Oxidoreductase molybdopterin binding domain	0.302

PF09378	+	HAS barrel domain	0.301
PF01068	+	ATP dependent DNA ligase domain	0.292
PF04389	+	Peptidase family M28	0.292
PF02789	+	Cytosol aminopeptidase family, N-terminal domain	0.287
PF03892	-	Nitrate reductase cytochrome c-type subunit (NapB)	0.286
PF09997	-	Predicted membrane protein (DUF2238)	0.284
PF03927	-	NapD protein	0.276
PF02086	+	D12 class N6 adenine-specific DNA methyltransferase	0.266
PF13474	-	SnoaL-like domain	0.265
PF03527	+	RHS protein	0.264
PF03783	-	Curli production assembly/transport component CsgG	0.261
PF14072	+	DNA-sulfur modification-associated	0.256
PF13488	+	Glycine zipper	0.253
PF13567	+	Domain of unknown function (DUF4131)	0.250
PF03481	+	Putative GTP-binding controlling metal-binding	0.249
PF00016	+	Ribulose bisphosphate carboxylase large chain, catalytic domain	0.240
PF03880	+	DbpA RNA binding domain	0.237
PF02574	-	Homocysteine S-methyltransferase	0.234
PF04679	+	ATP dependent DNA ligase C terminal region	0.232
PF14501	+	GHKL domain	0.226
PF08007	-	Cupin superfamily protein	0.217
PF10417	-	C-terminal domain of 1-Cys peroxiredoxin	0.215
PF10604	-	Polyketide cyclase / dehydrase and lipid transport	0.212
PF01081	-	KDPG and KHG aldolase	0.208
PF07102	+	Protein of unknown function (DUF1364)	0.208
PF04932	-	O-Antigen ligase	0.207
PF01909	-	Nucleotidyltransferase domain	0.206
PF01914	-	MarC family integral membrane protein	0.206
PF00043	+	Glutathione S-transferase, C-terminal domain	0.199
PF08173	-	Membrane bound YbgT-like protein	0.198
PF13298	+	DNA polymerase Ligase (LigD)	0.198
PF09956	+	Uncharacterized conserved protein (DUF2190)	0.198
PF12837	-	4Fe-4S binding domain	0.193
PF02915	-	Rubrerythrin	0.192
PF03869	+	Arc-like DNA binding domain	0.190
PF13527	+	Acetyltransferase (GNAT) domain	0.189
PF02624	-	YcaO-like family	0.187
PF14552	-	Tautomerase enzyme	0.187
PF06092	+	Enterobacterial putative membrane protein (DUF943)	0.178
PF09298	-	Fumarylacetoacetase N-terminal	0.177
PF01227	+	GTP cyclohydrolase I	0.177
PF14096	+	Domain of unknown function (DUF4274)	0.176
PF07732	-	Multicopper oxidase	0.166
PF01661	+	Macro domain	0.165
PF11041	-	Protein of unknown function (DUF2612)	0.164
PF01112	-	Asparaginase	0.162
PF13278	-	Putative amidotransferase	0.162
PF00282	+	Pyridoxal-dependent decarboxylase conserved domain	0.159

PF13425	-	O-antigen ligase like membrane protein	0.157
PF05360	-	yiaA/B two helix domain	0.155
PF10117	+	McrBC 5-methylcytosine restriction system component	0.154
PF00585	-	C-terminal regulatory domain of Threonine dehydratase	0.153
PF07283	+	Conjugal transfer protein TrbH	0.152
PF12951	-	Autotransporter-associated beta strand repeat	0.150
PF02615	-	Malate/L-lactate dehydrogenase	0.145
PF13391	-	HNH endonuclease	0.142
PF08774	+	VRR-NUC domain	0.141
PF13452	+	N-terminal half of MaoC dehydratase	0.141
PF12573	-	2-oxoisovalerate dehydrogenase E1 alpha subunit N terminal	0.138
PF04492	+	Bacteriophage replication protein O	0.138
PF04267	+	Sarcosine oxidase, delta subunit family	0.133
PF01923	-	Cobalamin adenosyltransferase	0.132
PF13576	-	Pentapeptide repeats (9 copies)	0.132
PF13807	+	G-rich domain on putative tyrosine kinase	0.127
PF01262	-	Alanine dehydrogenase/PNT, C-terminal domain	0.124
PF13593	-	SBF-like CPA transporter family (DUF4137)	0.123
PF08125	-	Mannitol dehydrogenase C-terminal domain	0.119
PF09296	+	NADH pyrophosphatase-like rudimentary NUDIX domain	0.119
PF06050	+	2-hydroxyglutaryl-CoA dehydratase, D-component	0.118
PF03681	-	Uncharacterised protein family (UPF0150)	0.116
PF01425	+	Amidase	0.112
PF08379	-	Bacterial transglutaminase-like N-terminal region	0.109
PF03060	+	Nitronate monooxygenase	0.108
PF07521	+	RNA-metabolising metallo-beta-lactamase	0.107
PF05521	+	Phage head-tail joining protein	0.105
PF10397	+	Adenylosuccinate lyase C-terminus	0.097
PF00258	-	Flavodoxin	0.086
PF11700	-	Vacuole effluxer Atg22 like	0.086
PF14302	-	Domain of unknown function (DUF4377)	0.084
PF02583	+	Metal-sensitive transcriptional repressor	0.084
PF01391	-	Collagen triple helix repeat (20 copies)	0.079
PF03547	-	Membrane transport protein	0.076
PF04223	-	Citrate lyase, alpha subunit (CitF)	0.075
PF07332	-	Protein of unknown function (DUF1469)	0.072
PF03354	+	Phage Terminase	0.072
PF00108	-	Thiolase, N-terminal domain	0.070
PF09669	-	Phage regulatory protein Rha (Phage_pRha)	0.068
PF00702	+	haloacid dehalogenase-like hydrolase	0.068
PF02237	-	Biotin protein ligase C terminal domain	0.068
PF01418	-	Helix-turn-helix domain, rpiR family	0.066
PF02274	+	Amidinotransferase	0.066
PF01059	+	NADH-ubiquinone oxidoreductase chain 4, amino terminus	0.063
PF01758	-	Sodium Bile acid symporter family	0.060
PF07411	-	Domain of unknown function (DUF1508)	0.054
PF01051	-	Initiator Replication protein	0.053
PF06252	+	Protein of unknown function (DUF1018)	0.053

PF05198	+	Translation initiation factor IF-3, N-terminal domain	0.052
PF02146	-	Sir2 family	0.045
PF13473	-	Cupredoxin-like domain	0.045
PF02113	-	D-Ala-D-Ala carboxypeptidase 3 (S13) family	0.040
PF05336	-	Domain of unknown function (DUF718)	0.039
PF01344	-	Kelch motif	0.038
PF03601	-	Conserved hypothetical protein 698	0.035
PF06293	-	Lipopolysaccharide kinase (Kdo/WaaP) family	0.031
PF03486	-	HI0933-like protein	0.031
PF13408	+	Recombinase zinc beta ribbon domain	0.023
PF11139	+	Protein of unknown function (DUF2910)	0.016
PF00665	-	Integrase core domain	0.015
PF02926	-	THUMP domain	0.013
PF00255	-	Glutathione peroxidase	0.003
PF05872	-	Bacterial protein of unknown function (DUF853)	0.002
PF10340	-	Protein of unknown function (DUF2424)	-0.005
PF02585	-	GlcNAc-PI de-N-acetylase	-0.009
PF07663	-	Sorbitol phosphotransferase enzyme II C-terminus	-0.013
PF02812	-	Glu/Leu/Phe/Val dehydrogenase, dimerisation domain	-0.015
PF11798	-	IMS family HHH motif	-0.023
PF13683	-	Integrase core domain	-0.023
PF03772	+	Competence protein	-0.024
PF01609	-	Transposase DDE domain	-0.025
PF13601	-	Winged helix DNA-binding domain	-0.026
PF03437	+	BtpA family	-0.027
PF12730	-	ABC-2 family transporter protein	-0.034
PF13333	-	Integrase core domain	-0.038
PF02317	-	NAD/NADP octopine/nopaline dehydrogenase, alpha-helical domain	-0.039
PF01642	-	Methylmalonyl-CoA mutase	-0.041
PF02706	-	Chain length determinant protein	-0.052
PF03406	-	Phage tail fibre repeat	-0.053
PF11611	+	Domain of unknown function (DUF4352)	-0.053
PF13523	-	Acetyltransferase (GNAT) domain	-0.059
PF03006	-	Haemolysin-III related	-0.060
PF03444	-	Winged helix-turn-helix transcription repressor, HrcA DNA-binding	-0.061
PF03308	-	ArgK protein	-0.063
PF06769	-	Plasmid encoded toxin Txe	-0.064
PF07669	-	Eco57I restriction-modification methylase	-0.065
PF08220	-	DeoR-like helix-turn-helix domain	-0.072
PF02806	-	Alpha amylase, C-terminal all-beta domain	-0.072
PF02922	-	Carbohydrate-binding module 48 (Isoamylase N-terminal domain)	-0.079
PF00908	-	dTDP-4-dehydrorhamnose 3,5-epimerase	-0.087
PF02673	-	Bacitracin resistance protein BacA	-0.103
PF00532	-	Periplasmic binding proteins and sugar binding domain of LacI family	-0.109
PF03606	-	C4-dicarboxylate anaerobic carrier	-0.112
PF00654	-	Voltage gated chloride channel	-0.121
PF04973	-	Nicotinamide mononucleotide transporter	-0.126
PF13377	-	Periplasmic binding protein-like domain	-0.132

PF01867	-	CRISPR associated protein Cas1	-0.132
PF04480	-	Protein of unknown function (DUF559)	-0.133
PF09827	-	CRISPR associated protein Cas2	-0.140
PF13936	+	Helix-turn-helix domain	-0.142
PF04221	-	RelB antitoxin	-0.143
PF00071	-	Ras family	-0.152
PF01443	-	Viral (Superfamily 1) RNA helicase	-0.179
PF13338	-	Domain of unknown function (DUF4095)	-0.185
PF13189	-	Cytidylate kinase-like family	-0.194
PF01637	-	Archaeal ATPase	-0.216
PF08244	-	Glycosyl hydrolases family 32 C terminal	-0.232

Phenotype: Spore formation (Morphology), Predictor: phypat

Pfam	class	Pfam_desc	cor
PF07875	+	Coat F domain	0.938
PF09560	+	Sporulation protein YunB (Spo_YunB)	0.938
PF13803	+	Domain of unknown function (DUF4184)	0.630
PF04456	+	Protein of unknown function (DUF503)	0.585
PF07736	+	Chorismate mutase type I	0.560
PF01944	+	Integral membrane protein DUF95	0.559
PF14431	+	YwqJ-like deaminase	0.515
PF01987	+	Mitochondrial biogenesis AIM24	0.489
PF00882	+	Zinc dependent phospholipase C	0.487
PF05991	+	YacP-like NYN domain	0.476
PF14689	+	Sensor_kinase_SpoOB-type, alpha-helical domain	0.470
PF04029	+	2-phosphosulpholactate phosphatase	0.458
PF13382	+	Adenine deaminase C-terminal domain	0.457
PF06803	+	Protein of unknown function (DUF1232)	0.455
PF01345	+	Domain of unknown function DUF11	0.443
PF09860	+	Uncharacterized protein conserved in bacteria (DUF2087)	0.437
PF12323	+	Helix-turn-helix domain	0.429
PF08031	+	Berberine and berberine like	0.427
PF03845	+	Spore germination protein	0.425
PF02522	+	Aminoglycoside 3-N-acetyltransferase	0.408
PF00704	+	Glycosyl hydrolases family 18	0.394
PF01865	+	Protein of unknown function DUF47	0.348
PF06675	+	Protein of unknown function (DUF1177)	0.335
PF10400	+	Virulence activator alpha C-term	0.325
PF02638	+	Glycosyl hydrolase like GH101	0.322
PF08378	+	Nuclease-related domain	0.313
PF10105	+	Uncharacterized protein conserved in bacteria (DUF2344)	0.282
PF00712	-	DNA polymerase III beta subunit, N-terminal domain	0.019
PF01171	-	PP-loop family	0.019
PF00308	-	Bacterial dnaA protein	0.019
PF00773	-	RNB domain	-0.008
PF01368	-	DHH family	-0.017
PF01935	-	Domain of unknown function DUF87	-0.025
PF01702	-	Queuine tRNA-ribosyltransferase	-0.037

PF00186	-	Dihydrofolate reductase	-0.046
PF04983	-	RNA polymerase Rpb1, domain 3	-0.055
PF04998	-	RNA polymerase Rpb1, domain 5	-0.055
PF04997	-	RNA polymerase Rpb1, domain 1	-0.055
PF00623	-	RNA polymerase Rpb1, domain 2	-0.055
PF02381	-	MraZ protein	-0.065
PF02661	-	Fic/DOC family	-0.092
PF01555	-	DNA methylase	-0.099
PF02424	-	ApbE family	-0.136
PF01431	-	Peptidase family M13	-0.138
PF03275	-	UDP-galactopyranose mutase	-0.162
PF01515	-	Phosphate acetyl/butaryl transferase	-0.176
PF13338	-	Domain of unknown function (DUF4095)	-0.189
PF02092	-	Glycyl-tRNA synthetase beta subunit	-0.194
PF04011	-	LemA family	-0.219
PF10417	-	C-terminal domain of 1-Cys peroxiredoxin	-0.221
PF02233	-	NAD(P) transhydrogenase beta subunit	-0.239
PF05016	-	Plasmid stabilisation system protein	-0.240
PF01027	-	Inhibitor of apoptosis-promoting Bax1	-0.252
PF13491	-	Domain of unknown function (DUF4117)	-0.267
PF03352	-	Methyladenine glycosylase	-0.276
PF01103	-	Surface antigen	-0.296
PF02646	-	RmuC family	-0.308
PF13245	-	Part of AAA domain	
PF02874	-	ATP synthase alpha/beta family, beta-barrel domain	
PF03120	-	NAD-dependent DNA ligase OB-fold domain	
PF13177	-	DNA polymerase III, delta subunit	

Phenotype: Spore formation (Morphology), Predictor: phypat+PGL

Pfam	class	Pfam_desc	cor
PF09560	+	Sporulation protein YunB (Spo_YunB)	0.938
PF07875	+	Coat F domain	0.938
PF07561	+	Domain of Unknown Function (DUF1540)	0.731
PF13803	+	Domain of unknown function (DUF4184)	0.630
PF02983	+	Alpha-lytic protease prodomain	0.322
PF00773	-	RNB domain	-0.008

Phenotype: Yellow pigment (Morphology), Predictor: phypat

Pfam	class	Pfam_desc	cor
PF12098	+	Protein of unknown function (DUF3574)	0.458
PF03988	+	Repeat of Unknown Function (DUF347)	0.406
PF13844	+	Glycosyl transferase family 41	0.398
PF13823	+	Alcohol dehydrogenase GroES-associated	0.388
PF00723	+	Glycosyl hydrolases family 15	0.364
PF08450	+	SMP-30/Gluconolaconase/LRE-like region	0.363
PF00797	+	N-acetyltransferase	0.351
PF13453	+	Transcription factor zinc-finger	0.335
PF05449	+	Protein of unknown function (DUF754)	0.330

PF12867	+	DinB superfamily	0.323
PF05227	+	CHASE3 domain	0.310
PF09811	+	Essential protein Yae1, N terminal	0.302
PF12872	+	OST-HTH/LOTUS domain	0.301
PF07024	+	ImpE protein	0.297
PF00494	+	Squalene/phytoene synthase	0.292
PF01152	+	Bacterial-like globin	0.287
PF11700	+	Vacuole effluxer Atg22 like	0.284
PF00982	+	Glycosyltransferase family 20	0.276
PF09863	+	Uncharacterized protein conserved in bacteria (DUF2090)	0.275
PF11744	+	Aluminium activated malate transporter	0.246
PF04606	+	Ogr/Delta-like zinc finger	0.239
PF04307	+	Predicted membrane-bound metal-dependent hydrolase (DUF457)	0.227
PF13176	+	Tetratricopeptide repeat	0.224
PF01661	+	Macro domain	0.210
PF04464	+	CDP-Glycerol:Poly(glycerophosphate) glycerophosphotransferase	0.201
PF09250	+	Bifunctional DNA primase/polymerase, N-terminal	0.187
PF01527	+	Transposase	0.177
PF07662	+	Na+ dependent nucleoside transporter C-terminus	0.157
PF01773	+	Na+ dependent nucleoside transporter N-terminus	0.157
PF14528	+	LAGLIDADG-like domain	0.157
PF05521	+	Phage head-tail joining protein	0.132
PF08211	+	Cytidine and deoxycytidylate deaminase zinc-binding region	0.129
PF03354	+	Phage Terminase	0.109
PF09587	+	Bacterial capsule synthesis protein PGA_cap	0.079
PF03831	-	PhnA protein	-0.055
PF13248	-	zinc-ribbon domain	-0.090
PF04205	-	FMN-binding domain	-0.102
PF04233	-	Phage Mu protein F like protein	-0.102
PF00232	-	Glycosyl hydrolase family 1	-0.119
PF07411	-	Domain of unknown function (DUF1508)	-0.121
PF00733	-	Asparagine synthase	-0.126
PF06042	-	Bacterial protein of unknown function (DUF925)	-0.132
PF04332	-	Protein of unknown function (DUF475)	-0.135
PF01116	-	Fructose-bisphosphate aldolase class-II	-0.137
PF08401	-	Domain of unknown function (DUF1738)	-0.137
PF00654	-	Voltage gated chloride channel	-0.146
PF13292	-	1-deoxy-D-xylulose-5-phosphate synthase	-0.146
PF03169	-	OPT oligopeptide transporter protein	-0.149
PF03734	-	L,D-transpeptidase catalytic domain	-0.172
PF13231	-	Dolichyl-phosphate-mannose-protein mannosyltransferase	-0.172
PF01637	-	Archaeal ATPase	-0.180
PF03432	-	Relaxase/Mobilisation nuclease domain	-0.194
PF03616	-	Sodium/glutamate symporter	-0.194
PF12631	-	Catalytic cysteine-containing C-terminus of GTPase, MnmE	-0.195
PF13932	-	GidA associated domain 3	-0.195
PF02386	-	Cation transport protein	-0.197
PF01554	-	MatE	-0.212

PF02575	-	YbaB/EbfC DNA-binding family	-0.212
PF10418	-	Iron-sulfur cluster binding domain of dihydroorotate dehydrogenase B	-0.228
PF13173	-	AAA domain	-0.237
PF13589	-	Histidine kinase-, DNA gyrase B-, and HSP90-like ATPase	-0.252
PF01790	-	Prolipoprotein diacylglyceryl transferase	-0.379

Phenotype: Yellow pigment (Morphology), Predictor: phypat+PGL

Pfam	class	Pfam_desc	cor
PF11397	+	Glycosyltransferase (GlcNAc)	0.399
PF03283	+	Pectinacetylesterase	0.394
PF13823	+	Alcohol dehydrogenase GroES-associated	0.388
PF08450	+	SMP-30/Gluconolaconase/LRE-like region	0.363
PF13453	+	Transcription factor zinc-finger	0.335
PF05449	+	Protein of unknown function (DUF754)	0.330
PF09995	+	Uncharacterized protein conserved in bacteria (DUF2236)	0.320
PF09811	+	Essential protein Yae1, N terminal	0.302
PF00494	+	Squalene/phytoene synthase	0.292
PF03583	+	Secretory lipase	0.276
PF06243	+	Phenylacetic acid degradation B	0.275
PF07652	+	Flavivirus DEAD domain	0.268
PF13428	+	Tetratricopeptide repeat	0.254
PF04264	+	YceI-like domain	0.247
PF02656	+	Domain of unknown function (DUF202)	0.232
PF07077	+	Protein of unknown function (DUF1345)	0.232
PF00875	+	DNA photolyase	0.231
PF06150	+	ChaB	0.230
PF04328	+	Protein of unknown function (DUF466)	0.225
PF13176	+	Tetratricopeptide repeat	0.224
PF01323	+	DSBA-like thioredoxin domain	0.219
PF14022	+	Protein of unknown function (DUF4238)	0.212
PF01719	+	Plasmid replication protein	0.212
PF05067	+	Manganese containing catalase	0.204
PF11867	+	Domain of unknown function (DUF3387)	0.204
PF04464	+	CDP-Glycerol:Poly(glycerophosphate) glycerophosphotransferase	0.201
PF09250	+	Bifunctional DNA primase/polymerase, N-terminal	0.187
PF13587	+	N-terminal domain of DJ-1_PfpI family	0.187
PF06356	+	Protein of unknown function (DUF1064)	0.181
PF13011	+	leucine-zipper of insertion element IS481	0.180
PF09152	+	Domain of unknown function (DUF1937)	0.174
PF07582	+	AP endonuclease family 2 C terminus	0.173
PF07081	+	Protein of unknown function (DUF1349)	0.172
PF10026	+	Predicted Zn-dependent protease (DUF2268)	0.169
PF11533	+	Protein of unknown function (DUF3225)	0.169
PF13079	+	Protein of unknown function (DUF3916)	0.164
PF02567	+	Phenazine biosynthesis-like protein	0.160
PF10459	+	Peptidase S46	0.157
PF13565	+	Homeodomain-like domain	0.153
PF09330	+	D-lactate dehydrogenase, membrane binding	0.151

PF06993	+	Protein of unknown function (DUF1304)	0.144
PF13020	+	Domain of unknown function (DUF3883)	0.127
PF06983	-	3-demethylubiquinone-9 3-methyltransferase	0.124
PF13551	+	Winged helix-turn helix	0.123
PF13391	+	HNH endonuclease	0.114
PF01051	-	Initiator Replication protein	0.101
PF04199	+	Putative cyclase	0.093
PF07015	-	VirC1 protein	0.091
PF07559	-	Flagellar basal body protein FlaE	0.086
PF09587	+	Bacterial capsule synthesis protein PGA_cap	0.079
PF07729	-	FCD domain	0.078
PF07308	+	Protein of unknown function (DUF1456)	0.071
PF06078	-	Bacterial protein of unknown function (DUF937)	0.071
PF13250	+	Domain of unknown function (DUF4041)	0.068
PF02136	+	Nuclear transport factor 2 (NTF2) domain	0.066
PF04191	-	Phospholipid methyltransferase	0.065
PF03235	+	Protein of unknown function DUF262	0.058
PF01739	-	CheR methyltransferase, SAM binding domain	0.058
PF05130	-	FlgN protein	0.048
PF05816	+	Toxic anion resistance protein (TelA)	0.044
PF11742	+	Protein of unknown function (DUF3302)	0.041
PF13539	-	D-alanyl-D-alanine carboxypeptidase	0.040
PF11188	+	Protein of unknown function (DUF2975)	0.036
PF13166	+	AAA domain	0.030
PF00766	-	Electron transfer flavoprotein FAD-binding domain	0.017
PF13567	-	Domain of unknown function (DUF4131)	0.017
PF03775	-	Septum formation inhibitor MinC, C-terminal domain	0.009
PF10110	+	Membrane domain of glycerophosphoryl diester phosphodiesterase	0.009
PF04087	+	Domain of unknown function (DUF389)	0.008
PF01914	-	MarC family integral membrane protein	-0.009
PF13576	-	Pentapeptide repeats (9 copies)	-0.030
PF03960	-	ArsC family	-0.030
PF04608	-	Phosphatidylglycerophosphatase A	-0.035
PF02954	-	Bacterial regulatory protein, Fis family	-0.038
PF10009	-	Uncharacterized protein conserved in bacteria (DUF2252)	-0.046
PF00931	-	NB-ARC domain	-0.081
PF07110	-	EthD domain	-0.087
PF05076	-	Suppressor of fused protein (SUFU)	-0.091
PF12799	-	Leucine Rich repeats (2 copies)	-0.094
PF07751	-	Abi-like protein	-0.096
PF04011	-	LemA family	-0.104
PF12008	-	Type I restriction and modification enzyme - subunit R C terminal	-0.108
PF10979	-	Protein of unknown function (DUF2786)	-0.116
PF06838	-	Methionine gamma-lyase	-0.117
PF03205	-	Molybdopterin guanine dinucleotide synthesis protein B	-0.118
PF07411	-	Domain of unknown function (DUF1508)	-0.121
PF14595	-	Thioredoxin	-0.129
PF02275	-	Linear amide C-N hydrolases, choloylglycine hydrolase family	-0.135

PF14720	-	NiFe/NiFeSe hydrogenase small subunit C-terminal	-0.137
PF07669	-	Eco57I restriction-modification methylase	-0.138
PF10662	+	Ethanolamine utilisation - propanediol utilisation	-0.138
PF07282	-	Putative transposase DNA-binding domain	-0.150
PF04221	-	RelB antitoxin	-0.169
PF01368	-	DHH family	-0.169
PF10418	-	Iron-sulfur cluster binding domain of dihydroorotate dehydrogenase B	-0.228
PF13173	-	AAA domain	-0.237
PF10458	-	Valyl tRNA synthetase tRNA binding arm	-0.252

Phenotype: Aerobe (Oxygen), Predictor: phypat

Pfam	class	Pfam_desc	cor
PF12766	+	Pyridoxamine 5'-phosphate oxidase	0.712
PF00743	+	Flavin-binding monooxygenase-like	0.700
PF04314	+	Protein of unknown function (DUF461)	0.619
PF00850	+	Histone deacetylase domain	0.614
PF00494	+	Squalene/phytoene synthase	0.596
PF00487	+	Fatty acid desaturase	0.586
PF03707	+	Bacterial signalling protein N terminal repeat	0.573
PF05227	+	CHASE3 domain	0.561
PF14539	+	Domain of unknown function (DUF4442)	0.558
PF01257	+	Thioredoxin-like [2Fe-2S] ferredoxin	0.536
PF13244	+	Domain of unknown function (DUF4040)	0.525
PF04066	+	Multiple resistance and pH regulation protein F (MrpF / PhaF)	0.521
PF08450	+	SMP-30/Gluconolaconase/LRE-like region	0.503
PF00662	+	NADH-Ubiquinone oxidoreductase (complex I), chain 5 N-terminus	0.502
PF11706	+	CGNR zinc finger	0.480
PF00080	+	Copper/zinc superoxide dismutase (SODC)	0.446
PF13667	+	ThiC-associated domain	0.406
PF13823	+	Alcohol dehydrogenase GroES-associated	0.403
PF04951	+	D-aminopeptidase	0.398
PF07286	+	Protein of unknown function (DUF1445)	0.378
PF01423	+	LSM domain	0.359
PF04140	+	Isoprenylcysteine carboxyl methyltransferase (ICMT) family	0.356
PF09179	+	TilS substrate binding domain	0.356
PF02954	+	Bacterial regulatory protein, Fis family	0.350
PF01510	+	N-acetylmuramoyl-L-alanine amidase	0.331
PF13565	+	Homeodomain-like domain	0.287
PF00175	+	Oxidoreductase NAD-binding domain	0.279
PF13646	+	HEAT repeats	0.251
PF07486	+	Cell Wall Hydrolase	0.245
PF04261	+	Dyp-type peroxidase family	0.234
PF06912	+	Protein of unknown function (DUF1275)	0.231
PF03413	+	Peptidase propeptide and YPEB domain	0.222
PF04186	+	FxsA cytoplasmic membrane protein	0.216
PF00939	+	Sodium:sulfate symporter transmembrane region	0.151
PF03853	-	YjeF-related protein N-terminus	0.142
PF02016	+	LD-carboxypeptidase	0.113

PF14789	-	Tetrahydrodipicolinate N-succinyltransferase middle	0.086
PF14436	+	Bacterial EndoU nuclease	0.076
PF08274	+	PhnA Zinc-Ribbon	0.071
PF04548	-	AIG1 family	0.058
PF02452	+	PemK-like protein	0.039
PF03729	-	Short repeat of unknown function (DUF308)	0.005
PF08352	-	Oligopeptide/dipeptide transporter, C-terminal region	-0.020
PF09924	-	Uncharacterized conserved protein (DUF2156)	-0.033
PF10417	-	C-terminal domain of 1-Cys peroxiredoxin	-0.043
PF01261	-	Xylose isomerase-like TIM barrel	-0.052
PF01443	+	Viral (Superfamily 1) RNA helicase	-0.053
PF03502	-	Nucleoside-specific channel-forming protein, Tsx	-0.060
PF02591	-	Putative zinc ribbon domain	-0.068
PF01906	-	Putative heavy-metal-binding	-0.085
PF01544	-	CorA-like Mg2+ transporter protein	-0.089
PF03610	-	PTS system fructose IIA component	-0.129
PF13813	-	Membrane bound O-acyl transferase family	-0.147
PF05135	-	Phage gp6-like head-tail connector protein	-0.166
PF13189	-	Cytidylate kinase-like family	-0.200
PF01855	-	Pyruvate flavodoxin/ferredoxin oxidoreductase, thiamine diP-bdg	-0.245
PF01633	-	Choline/ethanolamine kinase	-0.283
PF04023	-	FeoA domain	-0.287
PF02302	-	PTS system, Lactose/Cellobiose specific IIB subunit	-0.296
PF02588	-	Uncharacterized BCR, YitT family COG1284	-0.326
PF00232	-	Glycosyl hydrolase family 1	-0.328
PF12724	-	Flavodoxin domain	-0.346
PF01676	-	Metalloenzyme superfamily	-0.376
PF03605	-	Anaerobic c4-dicarboxylate membrane transporter	-0.379
PF07664	-	Ferrous iron transport protein B C terminus	-0.383
PF13173	-	AAA domain	-0.423
PF05116	-	Sucrose-6F-phosphate phosphohydrolase	-0.426
PF02568	-	Thiamine biosynthesis protein (ThiI)	-0.458
PF03590	-	Aspartate-ammonia ligase	-0.461
PF00365	-	Phosphofructokinase	-0.470
PF01268	-	Formate--tetrahydrofolate ligase	-0.512
PF02664	-	S-Ribosylhomocysteinase (LuxS)	-0.623
PF13597	-	Anaerobic ribonucleoside-triphosphate reductase	-0.653
PF01228	-	Glycine radical	-0.721

Phenotype: Aerobe (Oxygen), Predictor: phypat+PGL

Pfam	class	Pfam_desc	cor
PF12766	+	Pyridoxamine 5'-phosphate oxidase	0.712
PF00743	+	Flavin-binding monooxygenase-like	0.700
PF11953	+	Domain of unknown function (DUF3470)	0.684
PF00494	+	Squalene/phytoene synthase	0.596
PF00487	+	Fatty acid desaturase	0.586
PF05227	+	CHASE3 domain	0.561
PF14539	+	Domain of unknown function (DUF4442)	0.558

PF09832	+	Uncharacterized protein conserved in bacteria (DUF2059)	0.547
PF02636	+	Putative S-adenosyl-L-methionine-dependent methyltransferase	0.546
PF01257	+	Thioredoxin-like [2Fe-2S] ferredoxin	0.536
PF13670	+	Peptidase propeptide and YPEB domain	0.534
PF04066	+	Multiple resistance and pH regulation protein F (MrpF / PhaF)	0.521
PF13621	+	Cupin-like domain	0.514
PF03171	+	2OG-Fe(II) oxygenase superfamily	0.510
PF00662	+	NADH-Ubiquinone oxidoreductase (complex I), chain 5 N-terminus	0.502
PF05610	+	Protein of unknown function (DUF779)	0.487
PF05048	+	Periplasmic copper-binding protein (NosD)	0.484
PF03969	+	AFG1-like ATPase	0.479
PF14693	+	Ribosomal protein TL5, C-terminal domain	0.463
PF10604	+	Polyketide cyclase / dehydrase and lipid transport	0.459
PF10136	+	Site-specific recombinase	0.423
PF13823	+	Alcohol dehydrogenase GroES-associated	0.403
PF05573	+	NosL	0.381
PF10099	+	Anti-sigma-K factor rskA	0.379
PF07286	+	Protein of unknown function (DUF1445)	0.378
PF01126	+	Heme oxygenase	0.374
PF01019	+	Gamma-glutamyltranspeptidase	0.348
PF04536	+	TLP18.3, Psb32 and MOLO-1 founding proteins of phosphatase	0.339
PF00141	+	Peroxidase	0.327
PF04332	+	Protein of unknown function (DUF475)	0.306
PF08238	+	Sel1 repeat	0.302
PF11716	+	Mycothiol maleylpyruvate isomerase N-terminal domain	0.300
PF01135	+	Protein-L-isoaspartate(D-aspartate) O-methyltransferase (PCMT)	0.293
PF13011	+	leucine-zipper of insertion element IS481	0.289
PF13565	+	Homeodomain-like domain	0.287
PF00175	+	Oxidoreductase NAD-binding domain	0.279
PF03239	+	Iron permease FTR1 family	0.277
PF01936	-	NYN domain	0.275
PF07947	+	YhhN-like protein	0.272
PF05962	-	HutD	0.251
PF09844	+	Uncharacterized conserved protein (COG2071)	0.249
PF03454	-	MoeA C-terminal region (domain IV)	0.243
PF03352	+	Methyladenine glycosylase	0.242
PF02685	+	Glucokinase	0.239
PF01493	-	GXGXG motif	0.235
PF06912	+	Protein of unknown function (DUF1275)	0.231
PF01946	+	Thi4 family	0.229
PF14552	-	Tautomerase enzyme	0.211
PF02348	+	Cytidylyltransferase	0.207
PF01734	-	Patatin-like phospholipase	0.200
PF01139	+	tRNA-splicing ligase RtcB	0.192
PF02709	+	N-terminal domain of galactosyltransferase	0.185
PF07729	-	FCD domain	0.182
PF13185	-	GAF domain	0.176
PF09375	+	Imelysin	0.175

PF10842	+	Protein of unknown function (DUF2642)	0.173
PF00939	+	Sodium:sulfate symporter transmembrane region	0.151
PF13744	+	Helix-turn-helix domain	0.150
PF13086	+	AAA domain	0.146
PF04343	+	Protein of unknown function, DUF488	0.145
PF12729	-	Four helix bundle sensory module for signal transduction	0.126
PF13544	+	Type IV pilin N-term methylation site GFxxxE	0.118
PF13676	-	TIR domain	0.115
PF02040	-	Arsenical pump membrane protein	0.107
PF06414	+	Zeta toxin	0.091
PF03591	-	AzlC protein	0.091
PF14789	-	Tetrahydrodipicolinate N-succinyltransferase middle	0.086
PF05437	-	Branched-chain amino acid transport protein (AzlD)	0.074
PF00782	-	Dual specificity phosphatase, catalytic domain	0.074
PF04029	+	2-phosphosulpholactate phosphatase	0.073
PF13440	-	Polysaccharide biosynthesis protein	0.071
PF08274	+	PhnA Zinc-Ribbon	0.071
PF00722	+	Glycosyl hydrolases family 16	0.062
PF12974	-	ABC transporter, phosphonate, periplasmic substrate-binding protein	0.060
PF00825	+	Ribonuclease P	0.058
PF01555	-	DNA methylase	0.053
PF04069	-	Substrate binding domain of ABC-type glycine betaine transport system	0.052
PF08000	+	Bacterial PH domain	0.046
PF00350	-	Dynamin family	0.043
PF13443	+	Cro/C1-type HTH DNA-binding domain	0.039
PF02397	-	Bacterial sugar transferase	0.020
PF08003	-	Protein of unknown function (DUF1698)	0.019
PF03961	+	Protein of unknown function (DUF342)	0.016
PF01566	-	Natural resistance-associated macrophage protein	0.003
PF02311	-	AraC-like ligand binding domain	-0.007
PF01522	-	Polysaccharide deacetylase	-0.007
PF01558	-	Pyruvate ferredoxin/flavodoxin oxidoreductase	-0.013
PF07927	-	YcfA-like protein	-0.028
PF04519	-	Polymer-forming cytoskeletal	-0.028
PF10881	-	Protein of unknown function (DUF2726)	-0.029
PF09924	-	Uncharacterized conserved protein (DUF2156)	-0.033
PF07510	-	Protein of unknown function (DUF1524)	-0.081
PF03551	-	Transcriptional regulator PadR-like family	-0.082
PF07714	-	Protein tyrosine kinase	-0.097
PF04657	-	Protein of unknown function, DUF606	-0.106
PF02086	-	D12 class N6 adenine-specific DNA methyltransferase	-0.126
PF03610	-	PTS system fructose IIA component	-0.129
PF11907	+	Domain of unknown function (DUF3427)	-0.130
PF10145	-	Phage-related minor tail protein	-0.134
PF10387	-	Protein of unknown function (DUF2442)	-0.135
PF02733	-	Dak1 domain	-0.141
PF00481	-	Protein phosphatase 2C	-0.157
PF13407	-	Periplasmic binding protein domain	-0.160

PF14720	-	NiFe/NiFeSe hydrogenase small subunit C-terminal	-0.172
PF07831	-	Pyrimidine nucleoside phosphorylase C-terminal domain	-0.190
PF08497	-	Radical SAM N-terminal	-0.190
PF02872	-	5'-nucleotidase, C-terminal domain	-0.190
PF03773	-	Predicted permease	-0.200
PF04085	-	rod shape-determining protein MreC	-0.229
PF02378	-	Phosphotransferase system, EIIC	-0.307
PF12724	-	Flavodoxin domain	-0.346
PF01226	-	Formate/nitrite transporter	-0.356
PF07664	-	Ferrous iron transport protein B C terminus	-0.383
PF12464	-	Maltose acetyltransferase	-0.405
PF00365	-	Phosphofructokinase	-0.470
PF13597	-	Anaerobic ribonucleoside-triphosphate reductase	-0.653

Phenotype: Anaerobe (Oxygen), Predictor: phypat

Pfam	class	Pfam_desc	cor
PF02906	+	Iron only hydrogenase large subunit, C-terminal domain	0.738
PF09989	+	CoA enzyme activase uncharacterised domain (DUF2229)	0.732
PF01880	+	Desulfoferrodoxin	0.663
PF06050	+	2-hydroxyglutaryl-CoA dehydratase, D-component	0.659
PF10418	+	Iron-sulfur cluster binding domain of dihydroorotate dehydrogenase B	0.575
PF12392	+	Collagenase	0.560
PF03063	+	Prismane/CO dehydrogenase family	0.502
PF08984	+	Domain of unknown function (DUF1858)	0.502
PF02915	+	Rubrerythrin	0.484
PF02579	+	Dinitrogenase iron-molybdenum cofactor	0.483
PF13173	+	AAA domain	0.451
PF12900	+	Pyridoxamine 5'-phosphate oxidase	0.426
PF13248	+	zinc-ribbon domain	0.356
PF04205	+	FMN-binding domain	0.355
PF14393	+	Domain of unknown function (DUF4422)	0.348
PF03116	+	NQR2, RnfD, RnfE family	0.332
PF02508	+	Rnf-Nqr subunit, membrane protein	0.326
PF05896	+	Na(+)-translocating NADH-quinone reductase subunit A (NQRA)	0.307
PF14691	+	Dihydroprymidine dehydrogenase domain II, 4Fe-4S cluster	0.294
PF02659	+	Domain of unknown function DUF	0.284
PF01890	+	Cobalamin synthesis G C-terminus	0.262
PF13686	+	DsrE/DsrF/DrsH-like family	0.260
PF07691	+	PA14 domain	0.249
PF05272	+	Virulence-associated protein E	0.217
PF01661	+	Macro domain	0.201
PF01734	+	Patatin-like phospholipase	0.185
PF13463	+	Winged helix DNA-binding domain	0.168
PF04074	+	Domain of unknown function (DUF386)	0.165
PF01863	+	Protein of unknown function DUF45	0.149
PF01326	+	Pyruvate phosphate dikinase, PEP/pyruvate binding domain	0.148
PF02580	+	D-Tyr-tRNA(Tyr) deacylase	0.143
PF13192	+	Thioredoxin domain	0.135

PF04060	+	Putative Fe-S cluster	0.130
PF03950	+	tRNA synthetases class I (E and Q), anti-codon binding domain	0.082
PF03773	+	Predicted permease	0.082
PF13429	+	Tetratricopeptide repeat	0.064
PF01850	+	PIN domain	0.044
PF13506	+	Glycosyl transferase family 21	-0.044
PF07521	-	RNA-metabolising metallo-beta-lactamase	-0.046
PF01633	-	Choline/ethanolamine kinase	-0.086
PF01935	-	Domain of unknown function DUF87	-0.092
PF02568	-	Thiamine biosynthesis protein (ThiI)	-0.099
PF01396	-	Topoisomerase DNA binding C4 zinc finger	-0.113
PF00485	-	Phosphoribulokinase / Uridine kinase family	-0.149
PF02646	-	RmuC family	-0.163
PF01712	-	Deoxynucleoside kinase	-0.217
PF05191	-	Adenylate kinase, active site lid	-0.235
PF01161	-	Phosphatidylethanolamine-binding protein	-0.237
PF01219	-	Prokaryotic diacylglycerol kinase	-0.261
PF03672	-	Uncharacterised protein family (UPF0154)	-0.291
PF04461	-	Protein of unknown function (DUF520)	-0.292
PF03692	-	Putative zinc- or iron-chelating domain	-0.295
PF04167	-	Protein of unknown function (DUF402)	-0.315
PF02028	-	BCCT family transporter	-0.325
PF02237	-	Biotin protein ligase C terminal domain	-0.346
PF04314	-	Protein of unknown function (DUF461)	-0.355
PF13116	-	Protein of unknown function	-0.355
PF01432	-	Peptidase family M3	-0.355
PF03352	-	Methyladenine glycosylase	-0.378
PF02803	-	Thiolase, C-terminal domain	-0.381
PF00311	-	Phosphoenolpyruvate carboxylase	-0.383
PF03960	-	ArsC family	-0.384
PF00108	-	Thiolase, N-terminal domain	-0.384
PF00893	-	Small Multidrug Resistance protein	-0.390
PF07972	-	NrdI Flavodoxin like	-0.396
PF13442	-	Cytochrome C oxidase, cbb3-type, subunit III	-0.423
PF00034	-	Cytochrome c	-0.439
PF00317	-	Ribonucleotide reductase, all-alpha domain	-0.453
PF13410	-	Glutathione S-transferase, C-terminal domain	-0.468
PF03969	-	AFG1-like ATPase	-0.469
PF03831	-	PhnA protein	-0.490
PF13434	-	L-lysine 6-monooxygenase (NADPH-requiring)	-0.500
PF00334	-	Nucleoside diphosphate kinase	-0.544
PF00585	-	C-terminal regulatory domain of Threonine dehydratase	-0.546
PF00296	-	Luciferase-like monooxygenase	-0.552
PF01127	-	Succinate dehydrogenase/Fumarate reductase transmembrane subunit	-0.558
PF00115	-	Cytochrome C and Quinol oxidase polypeptide I	-0.565
PF02817	-	e3 binding domain	-0.696
PF00198	-	2-oxoacid dehydrogenases acyltransferase (catalytic domain)	-0.696
PF01149	-	Formamidopyrimidine-DNA glycosylase N-terminal domain	-0.712

Phenotype: Anaerobe (Oxygen), Predictor: phypat+PGL

Pfam	class	Pfam_desc	cor
PF02906	+	Iron only hydrogenase large subunit, C-terminal domain	0.738
PF09989	+	CoA enzyme activase uncharacterised domain (DUF2229)	0.732
PF06050	+	2-hydroxyglutaryl-CoA dehydratase, D-component	0.659
PF04961	+	Formiminotransferase-cyclodeaminase	0.654
PF10105	+	Uncharacterized protein conserved in bacteria (DUF2344)	0.590
PF12437	+	Glutamine synthetase type III N terminal	0.578
PF10418	+	Iron-sulfur cluster binding domain of dihydroorotate dehydrogenase B	0.575
PF04015	+	Domain of unknown function (DUF362)	0.562
PF13635	+	Domain of unknown function (DUF4143)	0.484
PF02579	+	Dinitrogenase iron-molybdenum cofactor	0.483
PF03577	+	Peptidase family C69	0.429
PF13240	+	zinc-ribbon domain	0.429
PF12900	+	Pyridoxamine 5'-phosphate oxidase	0.426
PF03030	+	Inorganic H+ pyrophosphatase	0.419
PF01268	+	Formate--tetrahydrofolate ligase	0.368
PF08486	+	Stage II sporulation protein	0.367
PF02457	+	DisA bacterial checkpoint controller nucleotide-binding	0.345
PF13399	+	LytR cell envelope-related transcriptional attenuator	0.338
PF07664	+	Ferrous iron transport protein B C terminus	0.332
PF03116	+	NQR2, RnfD, RnfE family	0.332
PF01228	+	Glycine radical	0.318
PF05896	+	Na(+)-translocating NADH-quinone reductase subunit A (NQRA)	0.307
PF03853	+	YjeF-related protein N-terminus	0.304
PF14691	+	Dihydroprymidine dehydrogenase domain II, 4Fe-4S cluster	0.294
PF02901	+	Pyruvate formate lyase	0.291
PF13686	+	DsrE/DsrF/DrsH-like family	0.260
PF04551	+	GcpE protein	0.252
PF06250	+	Protein of unknown function (DUF1016)	0.233
PF00754	-	F5/8 type C domain	0.233
PF13196	+	Protein of unknown function (DUF4012)	0.208
PF07670	+	Nucleoside recognition	0.206
PF03275	+	UDP-galactopyranose mutase	0.206
PF07510	-	Protein of unknown function (DUF1524)	0.201
PF04246	+	Positive regulator of sigma(E), RseC/MucC	0.201
PF11387	+	Protein of unknown function (DUF2795)	0.186
PF01734	+	Patatin-like phospholipase	0.185
PF00389	+	D-isomer specific 2-hydroxyacid dehydrogenase, catalytic domain	0.181
PF00722	-	Glycosyl hydrolases family 16	0.178
PF13690	+	Chemotaxis phosphatase CheX	0.173
PF02589	-	Uncharacterised ACR, YkgG family COG1556	0.155
PF01863	+	Protein of unknown function DUF45	0.149
PF01643	-	Acyl-ACP thioesterase	0.142
PF04511	+	Der1-like family	0.131
PF02632	+	BioY family	0.127
PF01008	-	Initiation factor 2 subunit family	0.122

PF13600	+	N-terminal domain of unknown function (DUF4140)	0.120
PF13657	+	HipA N-terminal domain	0.103
PF09913	+	Predicted membrane protein (DUF2142)	0.088
PF02922	-	Carbohydrate-binding module 48 (Isoamylase N-terminal domain)	0.087
PF13458	+	Periplasmic binding protein	0.072
PF14524	+	Wzt C-terminal domain	0.049
PF04313	-	Type I restriction enzyme R protein N terminus (HSDR_N)	0.030
PF03646	-	FlaG protein	0.029
PF14237	+	Domain of unknown function (DUF4339)	0.028
PF00682	+	HMGL-like	0.027
PF01618	+	MotA/TolQ/ExbB proton channel family	0.026
PF10589	+	NADH-ubiquinone oxidoreductase-F iron-sulfur binding region	0.025
PF12890	-	Dihydro-orotase-like	0.017
PF00903	+	Glyoxalase/Bleomycin resistance protein/Dioxygenase superfamily	0.014
PF01420	-	Type I restriction modification DNA specificity domain	0.013
PF12681	+	Glyoxalase-like domain	-0.000
PF03932	-	CutC family	-0.002
PF13087	-	AAA domain	-0.016
PF07476	-	Methylaspartate ammonia-lyase C-terminus	-0.031
PF02965	-	Vitamin B12 dependent methionine synthase, activation domain	-0.040
PF05656	+	Protein of unknown function (DUF805)	-0.053
PF05065	-	Phage capsid family	-0.063
PF03837	-	RecT family	-0.067
PF03259	+	Roadblock/LC7 domain	-0.070
PF13556	-	PucR C-terminal helix-turn-helix domain	-0.071
PF08269	-	Cache domain	-0.072
PF03592	-	Terminase small subunit	-0.077
PF12708	-	Pectate lyase superfamily protein	-0.078
PF13744	-	Helix-turn-helix domain	-0.080
PF09346	-	SMI1 / KNR4 family (SUKH-1)	-0.082
PF05971	-	Protein of unknown function (DUF890)	-0.096
PF01564	-	Spermine/spermidine synthase	-0.099
PF13354	+	Beta-lactamase enzyme family	-0.100
PF08546	-	Ketopantoate reductase PanE/ApbA C terminal	-0.104
PF04402	-	Protein of unknown function (DUF541)	-0.108
PF14789	+	Tetrahydrodipicolinate N-succinyltransferase middle	-0.114
PF03405	+	Fatty acid desaturase	-0.115
PF04860	-	Phage portal protein	-0.118
PF01844	-	HNH endonuclease	-0.120
PF04962	-	KduI/IolB family	-0.120
PF03547	-	Membrane transport protein	-0.122
PF00255	-	Glutathione peroxidase	-0.136
PF01928	-	CYTH domain	-0.140
PF03205	-	Molybdopterin guanine dinucleotide synthesis protein B	-0.144
PF00990	-	GGDEF domain	-0.151
PF13797	+	Post-transcriptional regulator	-0.153
PF00916	-	Sulfate transporter family	-0.156
PF13792	-	Sulfate transporter N-terminal domain with GLY motif	-0.156

PF08002	-	Protein of unknown function (DUF1697)	-0.162
PF02900	-	Catalytic LigB subunit of aromatic ring-opening dioxygenase	-0.162
PF00246	-	Zinc carboxypeptidase	-0.172
PF02604	-	Antitoxin Phd_YefM, type II toxin-antitoxin system	-0.173
PF01232	-	Mannitol dehydrogenase Rossmann domain	-0.176
PF08864	+	UPF0302 domain	-0.181
PF13483	-	Beta-lactamase superfamily domain	-0.188
PF08125	-	Mannitol dehydrogenase C-terminal domain	-0.190
PF13023	-	HD domain	-0.205
PF00854	-	POT family	-0.208
PF02569	-	Pantoate-beta-alanine ligase	-0.213
PF00144	-	Beta-lactamase	-0.220
PF02548	-	Ketopantoate hydroxymethyltransferase	-0.243
PF03707	+	Bacterial signalling protein N terminal repeat	-0.246
PF03971	-	Monomeric isocitrate dehydrogenase	-0.246
PF01507	-	Phosphoadenosine phosphosulfate reductase family	-0.247
PF03595	-	Voltage-dependent anion channel	-0.261
PF01219	-	Prokaryotic diacylglycerol kinase	-0.261
PF01154	+	Hydroxymethylglutaryl-coenzyme A synthase N terminal	-0.276
PF01192	-	RNA polymerase Rpb6	-0.292
PF01590	-	GAF domain	-0.303
PF13185	-	GAF domain	-0.314
PF13426	-	PAS domain	-0.314
PF05977	-	Transmembrane secretion effector	-0.315
PF00902	-	Sec-independent protein translocase protein (TatC)	-0.323
PF01503	-	Phosphoribosyl-ATP pyrophosphohydrolase	-0.337
PF02237	-	Biotin protein ligase C terminal domain	-0.346
PF03992	-	Antibiotic biosynthesis monooxygenase	-0.351
PF00989	-	PAS fold	-0.364
PF05768	-	Glutaredoxin-like domain (DUF836)	-0.380
PF00893	-	Small Multidrug Resistance protein	-0.390
PF05199	-	GMC oxidoreductase	-0.403
PF01904	-	Protein of unknown function DUF72	-0.406
PF01361	-	Tautomerase enzyme	-0.406
PF08274	-	PhnA Zinc-Ribbon	-0.419
PF00227	-	Proteasome subunit	-0.448
PF00762	-	Ferrochelatase	-0.546
PF00296	-	Luciferase-like monooxygenase	-0.552
PF02817	-	e3 binding domain	-0.696
PF00198	-	2-oxoacid dehydrogenases acyltransferase (catalytic domain)	-0.696
PF04673	+	Polyketide synthesis cyclase	

Phenotype: Capnophilic (Oxygen), Predictor: phypat

Pfam	class	Pfam_desc	cor
PF01637	+	Archaeal ATPase	0.224
PF07669	+	Eco57I restriction-modification methylase	0.217
PF02190	+	ATP-dependent protease La (LON) domain	0.161
PF00719	+	Inorganic pyrophosphatase	0.132

PF05860	+	haemagglutination activity domain	0.107
PF01420	+	Type I restriction modification DNA specificity domain	0.105
PF10592	+	AIPR protein	0.100
PF02660	+	Glycerol-3-phosphate acyltransferase	0.099
PF13505	+	Outer membrane protein beta-barrel domain	0.098
PF00216	+	Bacterial DNA-binding protein	0.098
PF00561	+	alpha/beta hydrolase fold	0.098
PF01396	+	Topoisomerase DNA binding C4 zinc finger	0.095
PF05198	+	Translation initiation factor IF-3, N-terminal domain	0.090
PF01555	+	DNA methylase	0.088
PF09848	+	Uncharacterized conserved protein (DUF2075)	0.085
PF00213	+	ATP synthase delta (OSCP) subunit	0.078
PF00707	+	Translation initiation factor IF-3, C-terminal domain	0.063
PF13726	+	Na+-H+ antiporter family	0.060
PF02504	+	Fatty acid synthesis protein	0.055
PF12826	+	Helix-hairpin-helix motif	0.051
PF03309	+	Type III pantothenate kinase	0.045
PF03636	+	Glycosyl hydrolase family 65, N-terminal domain	0.039
PF01633	+	Choline/ethanolamine kinase	0.031
PF00145	+	C-5 cytosine-specific DNA methylase	0.020
PF03616	+	Sodium/glutamate symporter	0.013
PF01935	+	Domain of unknown function DUF87	0.005
PF06325	+	Ribosomal protein L11 methyltransferase (PrmA)	-0.009
PF01027	+	Inhibitor of apoptosis-promoting Bax1	-0.019
PF03831	+	PhnA protein	-0.020
PF02541	+	Ppx/GppA phosphatase family	-0.042
PF13671	+	AAA domain	-0.048
PF03611	+	PTS system sugar-specific permease component	-0.052
PF00268	+	Ribonucleotide reductase, small chain	-0.058
PF02903	+	Alpha amylase, N-terminal ig-like domain	-0.059
PF00115	+	Cytochrome C and Quinol oxidase polypeptide I	-0.062
PF01554	+	MatE	-0.077
PF13089	+	Polyphosphate kinase N-terminal domain	-0.082
PF03060	+	Nitronate monooxygenase	-0.088
PF13090	+	Polyphosphate kinase C-terminal domain	-0.090
PF03706	+	Uncharacterised protein family (UPF0104)	-0.145
PF13426	+	PAS domain	-0.149
PF00800	+	Prephenate dehydratase	-0.173
PF05708	-	Orthopoxvirus protein of unknown function (DUF830)	-0.194
PF13181	-	Tetratricopeptide repeat	-0.201
PF12797	-	4Fe-4S binding domain	-0.246
PF01841	-	Transglutaminase-like superfamily	-0.271
PF13371	-	Tetratricopeptide repeat	-0.281
PF13610	-	DDE domain	-0.289
PF01610	-	Transposase	-0.306
PF10672	-	S-adenosylmethionine-dependent methyltransferase	-0.310
PF02557	-	D-alanyl-D-alanine carboxypeptidase	-0.322
PF02698	-	DUF218 domain	-0.324

PF04023	-	FeoA domain	-0.329
PF01758	-	Sodium Bile acid symporter family	-0.334
PF01729	-	Quinolinate phosphoribosyl transferase, C-terminal domain	-0.348
PF13492	-	GAF domain	-0.354
PF12974	-	ABC transporter, phosphonate, periplasmic substrate-binding protein	-0.372
PF09084	-	NMT1/THI5 like	-0.376
PF01527	-	Transposase	-0.381
PF01738	-	Dienelactone hydrolase family	-0.395
PF13555	-	P-loop containing region of AAA domain	-0.406
PF04138	-	GtrA-like protein	-0.408
PF13278	-	Putative amidotransferase	-0.412
PF02595	-	Glycerate kinase family	-0.419
PF09339	-	IclR helix-turn-helix domain	-0.429
PF01740	-	STAS domain	-0.431
PF07969	-	Amidohydrolase family	-0.446
PF01566	-	Natural resistance-associated macrophage protein	-0.448
PF00725	-	3-hydroxyacyl-CoA dehydrogenase, C-terminal domain	-0.452
PF02580	-	D-Tyr-tRNA(Tyr) deacylase	-0.456
PF02311	-	AraC-like ligand binding domain	-0.472
PF13602	-	Zinc-binding dehydrogenase	-0.475
PF14691	-	Dihydroprymidine dehydrogenase domain II, 4Fe-4S cluster	-0.483
PF00144	-	Beta-lactamase	-0.512
PF09186	-	Domain of unknown function (DUF1949)	-0.526
PF02782	-	FGGY family of carbohydrate kinases, C-terminal domain	-0.552
PF00370	-	FGGY family of carbohydrate kinases, N-terminal domain	-0.563
PF12802	-	MarR family	-0.628
PF12727	-	PBP superfamily domain	-0.633
PF13463	-	Winged helix DNA-binding domain	-0.642

Phenotype: Capnophilic (Oxygen), Predictor: phypat+PGL

Pfam	class	Pfam_desc	cor
PF11074	+	Domain of unknown function(DUF2779)	0.475
PF07672	+	Mycoplasma MFS transporter	0.406
PF13118	+	Protein of unknown function (DUF3972)	0.351
PF08966	+	Domain of unknown function (DUF1882)	0.351
PF10788	+	Protein of unknown function (DUF2603)	0.351
PF03498	+	Cytolethal distending toxin A/C family	0.250
PF14131	+	Domain of unknown function (DUF4298)	0.249
PF13252	+	Protein of unknown function (DUF4043)	0.231
PF07669	+	Eco57I restriction-modification methylase	0.217
PF09936	+	SAM-dependent RNA methyltransferase	0.193
PF13277	+	YmdB-like protein	0.185
PF13086	+	AAA domain	0.184
PF10119	+	Predicted methyltransferase regulatory domain	0.181
PF13173	+	AAA domain	0.160
PF09527	+	Putative F0F1-ATPase subunit (ATPase_gene1)	0.160
PF01921	+	tRNA synthetases class I (K)	0.142
PF00719	+	Inorganic pyrophosphatase	0.132

PF05990	+	Alpha/beta hydrolase of unknown function (DUF900)	0.124
PF05860	+	haemagglutination activity domain	0.107
PF12762	+	ISXO2-like transposase domain	0.107
PF10592	+	AIPR protein	0.100
PF13505	+	Outer membrane protein beta-barrel domain	0.098
PF08439	+	Oligopeptidase F	0.091
PF09848	+	Uncharacterized conserved protein (DUF2075)	0.085
PF13346	+	ABC-2 family transporter protein	0.085
PF05658	+	Head domain of trimeric autotransporter adhesin	0.083
PF06230	+	Protein of unknown function (DUF1009)	0.081
PF02677	+	Uncharacterized BCR, COG1636	0.072
PF02794	+	RTX toxin acyltransferase family	0.072
PF13310	+	Virulence protein RhuM family	0.071
PF04830	+	Possible hemagglutinin (DUF637)	0.065
PF08346	+	AntA/AntB antirepressor	0.062
PF09439	+	Signal recognition particle receptor beta subunit	0.062
PF13726	+	Na+-H+ antiporter family	0.060
PF03672	+	Uncharacterised protein family (UPF0154)	0.052
PF03968	+	OstA-like protein	0.042
PF10662	+	Ethanolamine utilisation - propanediol utilisation	0.034
PF09588	+	YqaJ-like viral recombinase domain	0.031
PF01633	+	Choline/ethanolamine kinase	0.031
PF03412	+	Peptidase C39 family	0.030
PF13787	+	Protein of unknown function with HXXEE motif	0.022
PF03605	+	Anaerobic c4-dicarboxylate membrane transporter	0.021
PF01268	+	Formate--tetrahydrofolate ligase	0.016
PF02030	+	Hypothetical lipoprotein (MG045 family)	0.010
PF05534	+	HicB family	0.009
PF06426	+	Serine acetyltransferase, N-terminal	-0.001
PF10711	+	Hypothetical protein (DUF2513)	-0.007
PF05154	+	TM2 domain	-0.012
PF11694	+	Protein of unknown function (DUF3290)	-0.012
PF13635	+	Domain of unknown function (DUF4143)	-0.014
PF10137	+	Predicted nucleotide-binding protein containing TIR-like domain	-0.023
PF04345	+	Chorismate lyase	-0.036
PF06965	+	Na+/H+ antiporter 1	-0.041
PF02685	+	Glucokinase	-0.058
PF02903	+	Alpha amylase, N-terminal ig-like domain	-0.059
PF09364	+	XFP N-terminal domain	-0.063
PF13166	-	AAA domain	-0.092
PF08447	+	PAS fold	-0.095
PF08274	+	PhnA Zinc-Ribbon	-0.098
PF01791	+	DeoC/LacD family aldolase	-0.099
PF06250	+	Protein of unknown function (DUF1016)	-0.106
PF12869	-	tRNA_anti-like	-0.112
PF11041	-	Protein of unknown function (DUF2612)	-0.119
PF01149	+	Formamidopyrimidine-DNA glycosylase N-terminal domain	-0.119
PF07549	-	SecD/SecF GG Motif	-0.122

PF03595	-	Voltage-dependent anion channel	-0.122
PF04170	-	NlpE N-terminal domain	-0.123
PF02475	+	Met-10+ like-protein	-0.124
PF01161	-	Phosphatidylethanolamine-binding protein	-0.127
PF10076	-	Uncharacterized protein conserved in bacteria (DUF2313)	-0.132
PF07021	-	Methionine biosynthesis protein MetW	-0.133
PF05272	-	Virulence-associated protein E	-0.136
PF00211	-	Adenylate and Guanylate cyclase catalytic domain	-0.142
PF06056	-	Putative ATPase subunit of terminase (gpP-like)	-0.145
PF07805	-	HipA-like N-terminal domain	-0.149
PF07179	-	SseB protein N-terminal domain	-0.155
PF06293	-	Lipopolysaccharide kinase (Kdo/WaaP) family	-0.155
PF08903	-	Domain of unknown function (DUF1846)	-0.156
PF06941	-	5' nucleotidase, deoxy (Pyrimidine), cytosolic type C protein (NT5C)	-0.156
PF13521	+	AAA domain	-0.158
PF00891	-	O-methyltransferase	-0.161
PF08415	-	Nonribosomal peptide synthase	-0.162
PF11794	-	4-hydroxyphenylacetate 3-hydroxylase N terminal	-0.165
PF12535	-	Hydrolase of X-linked nucleoside diphosphate N terminal	-0.165
PF01337	-	Barstar (barnase inhibitor)	-0.165
PF05857	-	TraX protein	-0.167
PF14502	-	Helix-turn-helix domain	-0.168
PF01443	-	Viral (Superfamily 1) RNA helicase	-0.168
PF14864	-	Alkyl sulfatase C-terminal	-0.171
PF03806	-	AbgT putative transporter family	-0.171
PF07811	+	TadE-like protein	-0.179
PF13653	-	Glycerophosphoryl diester phosphodiesterase family	-0.185
PF04606	-	Ogr/Delta-like zinc finger	-0.185
PF01223	-	DNA/RNA non-specific endonuclease	-0.185
PF00301	-	Rubredoxin	-0.192
PF01447	-	Thermolysin metallopeptidase, catalytic domain	-0.193
PF03825	+	Nucleoside H+ symporter	-0.194
PF13348	-	Tyrosine phosphatase family C-terminal region	-0.196
PF02868	-	Thermolysin metallopeptidase, alpha-helical domain	-0.196
PF03390	-	2-hydroxycarboxylate transporter family	-0.196
PF00201	-	UDP-glucoronosyl and UDP-glucosyl transferase	-0.198
PF02839	-	Carbohydrate binding domain	-0.198
PF03109	+	ABC1 family	-0.204
PF01226	-	Formate/nitrite transporter	-0.204
PF14690	-	zinc-finger of transposase IS204/IS1001/IS1096/IS1165	-0.204
PF03170	-	Bacterial cellulose synthase subunit	-0.207
PF13342	-	C-terminal repeat of topoisomerase	-0.207
PF06865	-	Protein of unknown function (DUF1255)	-0.207
PF13474	+	SnoaL-like domain	-0.209
PF02667	-	Short chain fatty acid transporter	-0.210
PF04183	-	IucA / IucC family	-0.212
PF03773	-	Predicted permease	-0.213
PF14526	-	Integron-associated effector binding protein	-0.217

PF06094	-	AIG2-like family	-0.218
PF03845	-	Spore germination protein	-0.221
PF13766	-	2-enoyl-CoA Hydratase C-terminal region	-0.227
PF00585	-	C-terminal regulatory domain of Threonine dehydratase	-0.228
PF03969	-	AFG1-like ATPase	-0.229
PF01855	-	Pyruvate flavodoxin/ferredoxin oxidoreductase, thiamine diP-bdg	-0.240
PF00116	-	Cytochrome C oxidase subunit II, periplasmic domain	-0.242
PF01957	-	NfeD-like C-terminal, partner-binding	-0.246
PF07683	-	Cobalamin synthesis protein cobW C-terminal domain	-0.248
PF03050	-	Transposase IS66 family	-0.249
PF02277	-	Phosphoribosyltransferase	-0.254
PF04262	-	Glutamate-cysteine ligase	-0.260
PF06081	-	Bacterial protein of unknown function (DUF939)	-0.260
PF08338	-	Domain of unknown function (DUF1731)	-0.263
PF14497	-	Glutathione S-transferase, C-terminal domain	-0.266
PF00092	-	von Willebrand factor type A domain	-0.266
PF04203	-	Sortase family	-0.267
PF02690	-	Na+/Pi-cotransporter	-0.271
PF00296	-	Luciferase-like monooxygenase	-0.277
PF04234	-	CopC domain	-0.278
PF07739	-	TipAS antibiotic-recognition domain	-0.286
PF12728	-	Helix-turn-helix domain	-0.295
PF00665	-	Integrase core domain	-0.302
PF10672	-	S-adenosylmethionine-dependent methyltransferase	-0.310
PF01590	-	GAF domain	-0.324
PF04960	-	Glutaminase	-0.324
PF02322	-	Cytochrome oxidase subunit II	-0.331
PF08448	+	PAS fold	-0.335
PF08240	+	Alcohol dehydrogenase GroES-like domain	-0.373
PF02803	+	Thiolase, C-terminal domain	-0.373
PF13185	-	GAF domain	-0.373
PF13480	-	Acetyltransferase (GNAT) domain	-0.374
PF02517	-	CAAX protease self-immunity	-0.383
PF01316	-	Arginine repressor, DNA binding domain	-0.384
PF01694	-	Rhomboid family	-0.407
PF09186	-	Domain of unknown function (DUF1949)	-0.526

Phenotype: Facultative (Oxygen), Predictor: phypat

Pfam	class	Pfam_desc	cor
PF02030	+	Hypothetical lipoprotein (MG045 family)	0.587
PF00874	+	PRD domain	0.556
PF00265	+	Thymidine kinase	0.527
PF02976	+	DNA mismatch repair enzyme MutH	0.499
PF01226	+	Formate/nitrite transporter	0.496
PF01238	+	Phosphomannose isomerase type I	0.493
PF12793	+	Sugar transport-related sRNA regulator N-term	0.489
PF00367	+	phosphotransferase system, EIIB	0.482
PF02901	+	Pyruvate formate lyase	0.478

PF01228	+	Glycine radical	0.476
PF03306	+	Alpha-acetolactate decarboxylase	0.473
PF00365	+	Phosphofructokinase	0.455
PF07694	+	5TMR of 5TMR-LYT	0.453
PF08343	+	Ribonucleotide reductase N-terminal	0.435
PF04245	+	37-kD nucleoid-associated bacterial protein	0.426
PF08211	+	Cytidine and deoxycytidylate deaminase zinc-binding region	0.425
PF03610	+	PTS system fructose IIA component	0.422
PF05257	+	CHAP domain	0.417
PF00485	+	Phosphoribulokinase / Uridine kinase family	0.417
PF05116	+	Sucrose-6F-phosphate phosphohydrolase	0.414
PF01633	+	Choline/ethanolamine kinase	0.409
PF04167	+	Protein of unknown function (DUF402)	0.407
PF14635	+	Helix-hairpin-helix motif	0.397
PF03605	+	Anaerobic c4-dicarboxylate membrane transporter	0.397
PF13275	+	S4 domain	0.393
PF03831	+	PhnA protein	0.388
PF00455	+	DeoR C terminal sensor domain	0.363
PF00198	+	2-oxoacid dehydrogenases acyltransferase (catalytic domain)	0.357
PF06426	+	Serine acetyltransferase, N-terminal	0.350
PF02733	+	Dak1 domain	0.346
PF13597	+	Anaerobic ribonucleoside-triphosphate reductase	0.342
PF01928	+	CYTH domain	0.339
PF04241	+	Protein of unknown function (DUF423)	0.333
PF04393	+	Protein of unknown function (DUF535)	0.332
PF02817	+	e3 binding domain	0.329
PF12911	+	N-terminal TM domain of oligopeptide transport permease C	0.318
PF04237	+	YjbR	0.308
PF04405	+	Domain of Unknown function (DUF542)	0.304
PF02624	+	YcaO-like family	0.302
PF08352	+	Oligopeptide/dipeptide transporter, C-terminal region	0.302
PF01219	+	Prokaryotic diacylglycerol kinase	0.275
PF08267	+	Cobalamin-independent synthase, N-terminal domain	0.274
PF02839	+	Carbohydrate binding domain	0.260
PF02324	+	Glycosyl hydrolase family 70	0.253
PF01712	+	Deoxynucleoside kinase	0.239
PF05135	+	Phage gp6-like head-tail connector protein	0.231
PF04606	+	Ogr/Delta-like zinc finger	0.231
PF12730	+	ABC-2 family transporter protein	0.224
PF14256	+	YwiC-like protein	0.216
PF03899	+	ATP synthase I chain	0.216
PF14526	+	Integron-associated effector binding protein	0.212
PF03848	+	Tellurite resistance protein TehB	0.210
PF07739	+	TipAS antibiotic-recognition domain	0.206
PF00311	+	Phosphoenolpyruvate carboxylase	0.202
PF01268	+	Formate--tetrahydrofolate ligase	0.191
PF13380	+	CoA binding domain	0.176
PF03441	+	FAD binding domain of DNA photolyase	0.162

PF02613	+	Nitrate reductase delta subunit	0.162
PF03264	+	NapC/NirT cytochrome c family, N-terminal region	0.150
PF02595	-	Glycerate kinase family	0.145
PF13410	+	Glutathione S-transferase, C-terminal domain	0.141
PF00145	+	C-5 cytosine-specific DNA methylase	0.140
PF03379	+	CcmB protein	0.124
PF11563	+	Protoglobin	0.122
PF05239	-	PRC-barrel domain	0.120
PF05728	+	Uncharacterised protein family (UPF0227)	0.106
PF04261	-	Dyp-type peroxidase family	0.084
PF04794	+	YdjC-like protein	0.081
PF13354	-	Beta-lactamase enzyme family	0.058
PF03649	-	Uncharacterised protein family (UPF0014)	0.057
PF01643	-	Acyl-ACP thioesterase	0.051
PF06207	-	Protein of unknown function (DUF1002)	0.047
PF04548	+	AIG1 family	0.045
PF04461	+	Protein of unknown function (DUF520)	0.041
PF10592	+	AIPR protein	0.040
PF01507	+	Phosphoadenosine phosphosulfate reductase family	0.039
PF00520	+	Ion transport protein	0.038
PF00903	-	Glyoxalase/Bleomycin resistance protein/Dioxygenase superfamily	0.034
PF13544	-	Type IV pilin N-term methylation site GFxxxE	0.030
PF11208	-	Protein of unknown function (DUF2992)	0.023
PF00258	-	Flavodoxin	0.018
PF02452	-	PemK-like protein	0.000
PF10593	+	Z1 domain	-0.012
PF06414	-	Zeta toxin	-0.012
PF06100	-	Streptococcal 67 kDa myosin-cross-reactive antigen like family	-0.026
PF01638	-	HxlR-like helix-turn-helix	-0.031
PF12802	-	MarR family	-0.040
PF01510	-	N-acetylmuramoyl-L-alanine amidase	-0.048
PF14690	-	zinc-finger of transposase IS204/IS1001/IS1096/IS1165	-0.048
PF00491	+	Arginase family	-0.053
PF13632	-	Glycosyl transferase family group 2	-0.059
PF04488	-	Glycosyltransferase sugar-binding region containing DXD motif	-0.063
PF13643	-	Domain of unknown function (DUF4145)	-0.063
PF03062	-	MBOAT, membrane-bound O-acyltransferase family	-0.064
PF01610	-	Transposase	-0.075
PF00022	-	Actin	-0.076
PF04263	-	Thiamin pyrophosphokinase, catalytic domain	-0.076
PF14897	-	EpsG family	-0.081
PF03577	-	Peptidase family C69	-0.082
PF02146	-	Sir2 family	-0.084
PF09250	-	Bifunctional DNA primase/polymerase, N-terminal	-0.094
PF13348	-	Tyrosine phosphatase family C-terminal region	-0.102
PF10604	-	Polyketide cyclase / dehydrase and lipid transport	-0.105
PF00939	-	Sodium:sulfate symporter transmembrane region	-0.117
PF01965	-	DJ-1/PfpI family	-0.124

PF06912	-	Protein of unknown function (DUF1275)	-0.136
PF13565	-	Homeodomain-like domain	-0.146
PF01427	-	D-ala-D-ala dipeptidase	-0.148
PF07883	-	Cupin domain	-0.150
PF13240	-	zinc-ribbon domain	-0.154
PF13083	-	KH domain	-0.156
PF13528	-	Glycosyl transferase family 1	-0.185
PF05893	-	Acyl-CoA reductase (LuxC)	-0.195
PF01243	-	Pyridoxamine 5'-phosphate oxidase	-0.196
PF05036	-	Sporulation related domain	-0.201
PF01869	-	BadF/BadG/BcrA/BcrD ATPase family	-0.202
PF01734	-	Patatin-like phospholipase	-0.210
PF00515	-	Tetratricopeptide repeat	-0.215
PF01850	-	PIN domain	-0.216
PF04343	-	Protein of unknown function, DUF488	-0.221
PF01326	-	Pyruvate phosphate dikinase, PEP/pyruvate binding domain	-0.232
PF04332	-	Protein of unknown function (DUF475)	-0.236
PF01809	-	Haemolytic domain	-0.236
PF07286	-	Protein of unknown function (DUF1445)	-0.237
PF00011	-	Hsp20/alpha crystallin family	-0.237
PF06947	-	Protein of unknown function (DUF1290)	-0.262
PF05949	-	Bacterial protein of unknown function (DUF881)	-0.262
PF09967	-	VWA-like domain (DUF2201)	-0.266
PF01315	-	Aldehyde oxidase and xanthine dehydrogenase, a/b hammerhead domain	-0.278
PF05683	-	Fumarase C-terminus	-0.280
PF05681	-	Fumarate hydratase (Fumerase)	-0.280
PF02666	-	Phosphatidylserine decarboxylase	-0.283
PF13286	-	Phosphohydrolase-associated domain	-0.290
PF06314	-	Acetoacetate decarboxylase (ADC)	-0.292
PF04909	-	Amidohydrolase	-0.298
PF01890	-	Cobalamin synthesis G C-terminus	-0.304
PF00743	-	Flavin-binding monooxygenase-like	-0.315
PF00075	-	RNase H	-0.315
PF00301	-	Rubredoxin	-0.350
PF13229	-	Right handed beta helix region	-0.359
PF12900	-	Pyridoxamine 5'-phosphate oxidase	-0.378
PF12766	-	Pyridoxamine 5'-phosphate oxidase	-0.391
PF03576	-	Peptidase family S58	-0.391
PF03853	-	YjeF-related protein N-terminus	-0.399
PF02401	-	LytB protein	-0.402
PF03309	-	Type III pantothenate kinase	-0.555
PF00142	-	4Fe-4S iron sulfur cluster binding proteins, NifH/frxC family	-0.604

Phenotype: Facultative (Oxygen), Predictor: phypat+PGL

Pfam	class	Pfam_desc	cor
PF02030	+	Hypothetical lipoprotein (MG045 family)	0.587
PF00874	+	PRD domain	0.556
PF02976	+	DNA mismatch repair enzyme MutH	0.499

PF10263	+	SprT-like family	0.495
PF01238	+	Phosphomannose isomerase type I	0.493
PF00367	+	phosphotransferase system, EIIB	0.482
PF03123	+	CAT RNA binding domain	0.482
PF02901	+	Pyruvate formate lyase	0.478
PF00358	+	phosphoenolpyruvate-dependent sugar phosphotransferase system, EIIA 1	0.478
PF01228	+	Glycine radical	0.476
PF04131	+	Putative N-acetylmannosamine-6-phosphate epimerase	0.463
PF00365	+	Phosphofructokinase	0.455
PF07694	+	5TMR of 5TMR-LYT	0.453
PF07972	+	NrdI Flavodoxin like	0.443
PF04245	+	37-kD nucleoid-associated bacterial protein	0.426
PF01791	+	DeoC/LacD family aldolase	0.424
PF03610	+	PTS system fructose IIA component	0.422
PF02664	+	S-Ribosylhomocysteinase (LuxS)	0.416
PF07085	+	DRTGG domain	0.416
PF03672	+	Uncharacterised protein family (UPF0154)	0.411
PF01633	+	Choline/ethanolamine kinase	0.409
PF04167	+	Protein of unknown function (DUF402)	0.407
PF14635	+	Helix-hairpin-helix motif	0.397
PF03605	+	Anaerobic c4-dicarboxylate membrane transporter	0.397
PF03831	+	PhnA protein	0.388
PF02903	+	Alpha amylase, N-terminal ig-like domain	0.372
PF01154	+	Hydroxymethylglutaryl-coenzyme A synthase N terminal	0.369
PF01184	+	GPR1/FUN34/yaaH family	0.365
PF06426	+	Serine acetyltransferase, N-terminal	0.350
PF07437	+	YfaZ precursor	0.340
PF04393	+	Protein of unknown function (DUF535)	0.332
PF03502	+	Nucleoside-specific channel-forming protein, Tsx	0.325
PF12911	+	N-terminal TM domain of oligopeptide transport permease C	0.318
PF04320	+	Protein with unknown function (DUF469)	0.316
PF04237	+	YjbR	0.308
PF04405	+	Domain of Unknown function (DUF542)	0.304
PF03799	+	Cell division protein FtsQ	0.304
PF02624	+	YcaO-like family	0.302
PF08352	+	Oligopeptide/dipeptide transporter, C-terminal region	0.302
PF13038	+	Domain of unknown function (DUF3899)	0.293
PF12481	+	Aluminium induced protein	0.284
PF13344	-	Haloacid dehalogenase-like hydrolase	0.263
PF15432	+	Accessory Sec secretory system ASP3	0.262
PF01095	+	Pectinesterase	0.253
PF13273	+	Protein of unknown function (DUF4064)	0.247
PF07759	+	Protein of unknown function (DUF1615)	0.245
PF01712	+	Deoxynucleoside kinase	0.239
PF01676	+	Metalloenzyme superfamily	0.231
PF14256	+	YwiC-like protein	0.216
PF04958	+	Arginine N-succinyltransferase beta subunit	0.207
PF07252	+	Protein of unknown function (DUF1433)	0.205

PF07274	-	Protein of unknown function (DUF1440)	0.197
PF01268	+	Formate--tetrahydrofolate ligase	0.191
PF11070	+	Protein of unknown function (DUF2871)	0.185
PF03486	+	HI0933-like protein	0.180
PF14319	+	Transposase zinc-binding domain	0.180
PF04986	+	Putative transposase	0.180
PF13726	+	Na+-H+ antiporter family	0.169
PF11042	+	Protein of unknown function (DUF2750)	0.165
PF02613	+	Nitrate reductase delta subunit	0.162
PF12571	+	Phage tail-collar fibre protein	0.161
PF00781	+	Diacylglycerol kinase catalytic domain	0.160
PF02922	+	Carbohydrate-binding module 48 (Isoamylase N-terminal domain)	0.157
PF13185	-	GAF domain	0.156
PF14490	-	Helix-hairpin-helix containing domain	0.154
PF13349	-	Domain of unknown function (DUF4097)	0.153
PF03264	+	NapC/NirT cytochrome c family, N-terminal region	0.150
PF13558	-	Putative exonuclease SbcCD, C subunit	0.149
PF12008	+	Type I restriction and modification enzyme - subunit R C terminal	0.147
PF02595	-	Glycerate kinase family	0.145
PF04962	+	KduI/IolB family	0.142
PF04186	-	FxsA cytoplasmic membrane protein	0.142
PF08003	-	Protein of unknown function (DUF1698)	0.140
PF02335	+	Cytochrome c552	0.139
PF13434	-	L-lysine 6-monooxygenase (NADPH-requiring)	0.137
PF09318	-	Domain of unknown function (DUF1975)	0.136
PF13520	+	Amino acid permease	0.132
PF03379	+	CcmB protein	0.124
PF14542	+	GCN5-related N-acetyl-transferase	0.114
PF07476	-	Methylaspartate ammonia-lyase C-terminus	0.110
PF13367	-	Protease prsW family	0.110
PF01289	+	Thiol-activated cytolysin	0.108
PF05728	+	Uncharacterised protein family (UPF0227)	0.106
PF02806	+	Alpha amylase, C-terminal all-beta domain	0.102
PF04204	-	Homoserine O-succinyltransferase	0.102
PF01443	-	Viral (Superfamily 1) RNA helicase	0.101
PF13276	-	HTH-like domain	0.099
PF02748	+	Aspartate carbamoyltransferase regulatory chain, metal binding domain	0.090
PF01270	-	Glycosyl hydrolases family 8	0.090
PF06149	-	Protein of unknown function (DUF969)	0.090
PF09179	+	TilS substrate binding domain	0.090
PF01011	-	PQQ enzyme repeat	0.088
PF12890	-	Dihydro-orotase-like	0.087
PF02690	-	Na+/Pi-cotransporter	0.086
PF00248	-	Aldo/keto reductase family	0.082
PF05991	-	YacP-like NYN domain	0.079
PF05065	-	Phage capsid family	0.079
PF05534	+	HicB family	0.079
PF14133	+	Domain of unknown function (DUF4300)	0.076

PF13596	-	PAS domain	0.075
PF04213	+	Htaa	0.075
PF10662	+	Ethanolamine utilisation - propanediol utilisation	0.063
PF01420	+	Type I restriction modification DNA specificity domain	0.061
PF04404	-	ERF superfamily	0.057
PF02086	-	D12 class N6 adenine-specific DNA methyltransferase	0.056
PF12197	+	Bacillus cereus group antimicrobial protein	0.056
PF13730	-	Helix-turn-helix domain	0.052
PF14306	-	PUA-like domain	0.048
PF06207	-	Protein of unknown function (DUF1002)	0.047
PF06810	-	Phage minor structural protein GP20	0.046
PF04548	+	AIG1 family	0.045
PF10592	+	AIPR protein	0.040
PF14489	-	QueF-like protein	0.039
PF02457	+	DisA bacterial checkpoint controller nucleotide-binding	0.038
PF01076	-	Plasmid recombination enzyme	0.037
PF08328	+	Adenylosuccinate lyase C-terminal	0.033
PF03618	-	Kinase/pyrophosphorylase	0.032
PF03977	-	Na+-transporting oxaloacetate decarboxylase beta subunit	0.029
PF01946	-	Thi4 family	0.028
PF03547	-	Membrane transport protein	0.026
PF06999	+	Sucrase/ferredoxin-like	0.025
PF12733	+	Cadherin-like beta sandwich domain	0.025
PF00216	+	Bacterial DNA-binding protein	0.023
PF04886	+	PT repeat	0.020
PF11794	-	4-hydroxyphenylacetate 3-hydroxylase N terminal	0.018
PF02574	+	Homocysteine S-methyltransferase	0.016
PF13376	+	Bacteriocin-protection, YdeI or OmpD-Associated	0.014
PF13723	-	Beta-ketoacyl synthase, N-terminal domain	0.014
PF01391	-	Collagen triple helix repeat (20 copies)	0.010
PF00318	+	Ribosomal protein S2	0.008
PF11870	-	Domain of unknown function (DUF3390)	0.007
PF03413	-	Peptidase propeptide and YPEB domain	0.007
PF03243	-	Alkylmercury lyase	0.006
PF14552	-	Tautomerase enzyme	0.004
PF14804	-	Jag N-terminus	0.004
PF14464	-	Prokaryotic homologs of the JAB domain	0.001
PF01797	-	Transposase IS200 like	-0.000
PF12242	+	NAD(P)H binding domain of trans-2-enoyl-CoA reductase	-0.002
PF06508	-	Queuosine biosynthesis protein QueC	-0.002
PF02609	-	Exonuclease VII small subunit	-0.005
PF05729	-	NACHT domain	-0.006
PF03746	-	LamB/YcsF family	-0.008
PF06414	-	Zeta toxin	-0.012
PF02682	-	Allophanate hydrolase subunit 1	-0.013
PF13682	-	Chemoreceptor zinc-binding domain	-0.013
PF13529	-	Peptidase_C39 like family	-0.014
PF13394	-	4Fe-4S single cluster domain	-0.014

PF03050	-	Transposase IS66 family	-0.015
PF00282	+	Pyridoxal-dependent decarboxylase conserved domain	-0.016
PF13360	+	PQQ-like domain	-0.019
PF00331	+	Glycosyl hydrolase family 10	-0.020
PF01385	-	Probable transposase	-0.020
PF00719	+	Inorganic pyrophosphatase	-0.026
PF06100	-	Streptococcal 67 kDa myosin-cross-reactive antigen like family	-0.026
PF12072	-	Domain of unknown function (DUF3552)	-0.031
PF12740	-	Chlorophyllase enzyme	-0.031
PF01638	-	HxlR-like helix-turn-helix	-0.031
PF01699	-	Sodium/calcium exchanger protein	-0.032
PF07282	-	Putative transposase DNA-binding domain	-0.033
PF13247	+	4Fe-4S dicluster domain	-0.036
PF07683	-	Cobalamin synthesis protein cobW C-terminal domain	-0.038
PF12127	-	SigmaW regulon antibacterial	-0.038
PF03845	-	Spore germination protein	-0.038
PF08757	-	CotH protein	-0.043
PF10704	-	Protein of unknown function (DUF2508)	-0.048
PF01547	-	Bacterial extracellular solute-binding protein	-0.048
PF01008	-	Initiation factor 2 subunit family	-0.050
PF14378	-	PAP2 superfamily	-0.050
PF04014	-	Antidote-toxin recognition MazE	-0.053
PF00491	+	Arginase family	-0.053
PF04392	-	ABC transporter substrate binding protein	-0.054
PF14531	+	Kinase-like	-0.056
PF00080	-	Copper/zinc superoxide dismutase (SODC)	-0.057
PF13463	-	Winged helix DNA-binding domain	-0.057
PF01745	-	Isopentenyl transferase	-0.059
PF10531	-	SLBB domain	-0.060
PF13542	-	Helix-turn-helix domain of transposase family ISL3	-0.061
PF01051	-	Initiator Replication protein	-0.062
PF04488	-	Glycosyltransferase sugar-binding region containing DXD motif	-0.063
PF13643	-	Domain of unknown function (DUF4145)	-0.063
PF04519	+	Polymer-forming cytoskeletal	-0.064
PF09084	-	NMT1/THI5 like	-0.068
PF09965	+	Uncharacterized protein conserved in bacteria (DUF2199)	-0.071
PF02965	+	Vitamin B12 dependent methionine synthase, activation domain	-0.071
PF03703	-	Bacterial PH domain	-0.072
PF00082	-	Subtilase family	-0.073
PF04263	-	Thiamin pyrophosphokinase, catalytic domain	-0.076
PF14897	-	EpsG family	-0.081
PF10340	-	Protein of unknown function (DUF2424)	-0.084
PF13500	+	AAA domain	-0.085
PF01408	-	Oxidoreductase family, NAD-binding Rossmann fold	-0.087
PF05272	-	Virulence-associated protein E	-0.091
PF14697	-	4Fe-4S dicluster domain	-0.092
PF06833	-	Malonate decarboxylase gamma subunit (MdcE)	-0.094
PF14358	-	Domain of unknown function (DUF4405)	-0.100

PF01978	-	Sugar-specific transcriptional regulator TrmB	-0.102
PF13348	-	Tyrosine phosphatase family C-terminal region	-0.102
PF01891	-	Cobalt uptake substrate-specific transmembrane region	-0.103
PF09991	-	Predicted membrane protein (DUF2232)	-0.105
PF07905	-	Purine catabolism regulatory protein-like family	-0.108
PF07083	-	Protein of unknown function (DUF1351)	-0.110
PF03741	-	Integral membrane protein TerC family	-0.112
PF02275	-	Linear amide C-N hydrolases, choloylglycine hydrolase family	-0.112
PF02436	-	Conserved carboxylase domain	-0.115
PF01050	+	Mannose-6-phosphate isomerase	-0.116
PF09369	-	Domain of unknown function (DUF1998)	-0.117
PF07510	-	Protein of unknown function (DUF1524)	-0.119
PF10081	-	Alpha/beta-hydrolase family	-0.124
PF15420	-	Alpha/beta-hydrolase family N-terminus	-0.124
PF02585	-	GlcNAc-PI de-N-acetylase	-0.127
PF14393	-	Domain of unknown function (DUF4422)	-0.127
PF05544	-	Proline racemase	-0.128
PF08392	-	FAE1/Type III polyketide synthase-like protein	-0.140
PF13379	-	NMT1-like family	-0.144
PF12784	-	PD-(D/E)XK nuclease family transposase	-0.145
PF13565	-	Homeodomain-like domain	-0.146
PF02310	+	B12 binding domain	-0.151
PF13240	-	zinc-ribbon domain	-0.154
PF07804	-	HipA-like C-terminal domain	-0.156
PF14532	-	Sigma-54 interaction domain	-0.161
PF13768	-	von Willebrand factor type A domain	-0.161
PF05402	-	Coenzyme PQQ synthesis protein D (PqqD)	-0.164
PF01144	-	Coenzyme A transferase	-0.164
PF00725	-	3-hydroxyacyl-CoA dehydrogenase, C-terminal domain	-0.166
PF04011	-	LemA family	-0.168
PF01969	-	Protein of unknown function DUF111	-0.171
PF07228	-	Stage II sporulation protein E (SpoIIE)	-0.176
PF01613	-	Flavin reductase like domain	-0.178
PF01957	-	NfeD-like C-terminal, partner-binding	-0.185
PF08666	-	SAF domain	-0.188
PF04951	-	D-aminopeptidase	-0.188
PF13243	-	Prenyltransferase-like	-0.189
PF00931	-	NB-ARC domain	-0.197
PF13432	+	Tetratricopeptide repeat	-0.205
PF00670	+	S-adenosyl-L-homocysteine hydrolase, NAD binding domain	-0.206
PF01734	-	Patatin-like phospholipase	-0.210
PF03481	-	Putative GTP-binding controlling metal-binding	-0.212
PF00515	-	Tetratricopeptide repeat	-0.215
PF01850	-	PIN domain	-0.216
PF08486	-	Stage II sporulation protein	-0.217
PF10294	-	Putative methyltransferase	-0.217
PF12682	-	Flavodoxin	-0.218
PF00111	+	2Fe-2S iron-sulfur cluster binding domain	-0.219

PF03186	-	CobD/Cbib protein	-0.219
PF01809	-	Haemolytic domain	-0.236
PF03008	-	Archaea bacterial proteins of unknown function	-0.239
PF07021	-	Methionine biosynthesis protein MetW	-0.245
PF13524	-	Glycosyl transferases group 1	-0.251
PF13621	+	Cupin-like domain	-0.255
PF01964	-	ThiC family	-0.255
PF06947	-	Protein of unknown function (DUF1290)	-0.262
PF05949	-	Bacterial protein of unknown function (DUF881)	-0.262
PF01968	+	Hydantoinase/oxoprolinase	-0.266
PF09967	-	VWA-like domain (DUF2201)	-0.266
PF11760	-	Cobalamin synthesis G N-terminal	-0.268
PF00733	-	Asparagine synthase	-0.278
PF07685	-	CobB/CobQ-like glutamine amidotransferase domain	-0.289
PF04909	-	Amidohydrolase	-0.298
PF00075	-	RNase H	-0.315
PF00301	-	Rubredoxin	-0.350
PF13229	-	Right handed beta helix region	-0.359
PF12900	-	Pyridoxamine 5'-phosphate oxidase	-0.378
PF01012	-	Electron transfer flavoprotein domain	-0.386
PF03576	-	Peptidase family S58	-0.391
PF02915	+	Rubrerythrin	-0.483
PF03309	-	Type III pantothenate kinase	-0.555
PF00142	-	4Fe-4S iron sulfur cluster binding proteins, NifH/frxC family	-0.604

Phenotype: Catalase (Oxygen:Enzyme), Predictor: phypat

Pfam	class	Pfam_desc	cor
PF00199	+	Catalase	0.885
PF06628	+	Catalase-related immune-responsive	0.867
PF01619	+	Proline dehydrogenase	0.834
PF04264	+	YceI-like domain	0.769
PF01208	+	Uroporphyrinogen decarboxylase (URO-D)	0.759
PF00463	+	Isocitrate lyase family	0.735
PF00355	+	Rieske [2Fe-2S] domain	0.719
PF00585	+	C-terminal regulatory domain of Threonine dehydratase	0.677
PF04715	+	Anthranilate synthase component I, N terminal region	0.671
PF04241	+	Protein of unknown function (DUF423)	0.652
PF02622	+	Uncharacterized ACR, COG1678	0.643
PF04234	+	CopC domain	0.632
PF00487	+	Fatty acid desaturase	0.632
PF06609	+	Fungal trichothecene efflux pump (TRI12)	0.624
PF02628	+	Cytochrome oxidase assembly protein	0.609
PF01152	+	Bacterial-like globin	0.600
PF02668	+	Taurine catabolism dioxygenase TauD, TfdA family	0.593
PF04898	+	Glutamate synthase central domain	0.575
PF02803	+	Thiolase, C-terminal domain	0.550
PF09339	+	IclR helix-turn-helix domain	0.498
PF13857	+	Ankyrin repeats (many copies)	0.478

PF03350	+	Uncharacterized protein family, UPF0114	0.454
PF02746	+	Mandelate racemase / muconate lactonizing enzyme, N-terminal domain	0.411
PF10503	+	Esterase PHB depolymerase	0.406
PF00561	-	alpha/beta hydrolase fold	0.259
PF00493	-	MCM2/3/5 family	0.246
PF01636	-	Phosphotransferase enzyme family	0.211
PF13086	-	AAA domain	0.135
PF01554	-	MatE	0.119
PF13167	-	GTP-binding GTPase N-terminal	0.114
PF02151	-	UvrB/uvrC motif	0.083
PF08765	-	Mor transcription activator family	0.074
PF01145	-	SPFH domain / Band 7 family	0.051
PF14821	+	Threonine synthase N terminus	0.039
PF03706	-	Uncharacterised protein family (UPF0104)	0.030
PF05521	-	Phage head-tail joining protein	0.004
PF01895	-	PhoU domain	-0.008
PF00216	-	Bacterial DNA-binding protein	-0.014
PF12631	-	Catalytic cysteine-containing C-terminus of GTPase, MnmE	-0.033
PF13932	-	GidA associated domain 3	-0.033
PF03193	-	Protein of unknown function, DUF258	-0.056
PF08423	-	Rad51	-0.100
PF12706	-	Beta-lactamase superfamily domain	-0.119
PF02677	-	Uncharacterized BCR, COG1636	-0.132
PF04205	-	FMN-binding domain	-0.135
PF03065	-	Glycosyl hydrolase family 57	-0.143
PF10080	-	Predicted membrane protein (DUF2318)	-0.187
PF10662	-	Ethanolamine utilisation - propanediol utilisation	-0.204
PF09848	-	Uncharacterized conserved protein (DUF2075)	-0.237
PF02664	-	S-Ribosylhomocysteinase (LuxS)	-0.250
PF14595	-	Thioredoxin	-0.251
PF09861	-	Domain of unknown function (DUF2088)	-0.256
PF04392	-	ABC transporter substrate binding protein	-0.261
PF01921	-	tRNA synthetases class I (K)	-0.273
PF01643	-	Acyl-ACP thioesterase	-0.274
PF03275	-	UDP-galactopyranose mutase	-0.275
PF01969	-	Protein of unknown function DUF111	-0.281
PF13597	-	Anaerobic ribonucleoside-triphosphate reductase	-0.310
PF01228	-	Glycine radical	-0.331
PF06050	-	2-hydroxyglutaryl-CoA dehydratase, D-component	-0.332
PF04011	-	LemA family	-0.364
PF02361	-	Cobalt transport protein	-0.377
PF13083	-	KH domain	-0.424
PF13173	-	AAA domain	-0.458
PF12072	-	Domain of unknown function (DUF3552)	-0.499
PF08984	-	Domain of unknown function (DUF1858)	-0.500
PF10035	-	Uncharacterized protein conserved in bacteria (DUF2179)	-0.512
PF04263	-	Thiamin pyrophosphokinase, catalytic domain	-0.528
PF03577	-	Peptidase family C69	-0.539

Phenotype: Catalase (Oxygen:Enzyme), Predictor: phypat+PGL

Pfam	class	Pfam_desc	cor
PF00199	+	Catalase	0.885
PF06628	+	Catalase-related immune-responsive	0.867
PF01619	+	Proline dehydrogenase	0.834
PF04264	+	YceI-like domain	0.769
PF00762	+	Ferrochelatase	0.735
PF00034	+	Cytochrome c	0.691
PF04715	+	Anthranilate synthase component I, N terminal region	0.671
PF00285	+	Citrate synthase	0.668
PF13806	+	Rieske-like [2Fe-2S] domain	0.666
PF04241	+	Protein of unknown function (DUF423)	0.652
PF08338	+	Domain of unknown function (DUF1731)	0.646
PF02622	+	Uncharacterized ACR, COG1678	0.643
PF00487	+	Fatty acid desaturase	0.632
PF02628	+	Cytochrome oxidase assembly protein	0.609
PF01152	+	Bacterial-like globin	0.600
PF04898	+	Glutamate synthase central domain	0.575
PF02803	+	Thiolase, C-terminal domain	0.550
PF02834	-	LigT like Phosphoesterase	0.517
PF00198	+	2-oxoacid dehydrogenases acyltransferase (catalytic domain)	0.516
PF00719	+	Inorganic pyrophosphatase	0.488
PF13857	+	Ankyrin repeats (many copies)	0.478
PF00108	+	Thiolase, N-terminal domain	0.467
PF08447	+	PAS fold	0.460
PF01645	+	Conserved region in glutamate synthase	0.458
PF02230	+	Phospholipase/Carboxylesterase	0.447
PF01706	-	FliG C-terminal domain	0.441
PF14841	-	FliG middle domain	0.441
PF14842	-	FliG N-terminal domain	0.434
PF14805	+	Tetrahydrodipicolinate N-succinyltransferase N-terminal	0.432
PF14849	+	YidC periplasmic domain	0.419
PF14748	+	Pyrroline-5-carboxylate reductase dimerisation	0.381
PF05728	-	Uncharacterised protein family (UPF0227)	0.369
PF08240	+	Alcohol dehydrogenase GroES-like domain	0.353
PF01315	-	Aldehyde oxidase and xanthine dehydrogenase, a/b hammerhead domain	0.351
PF12832	-	MFS_1 like family	0.348
PF07660	-	Secretin and TonB N terminus short domain	0.340
PF13036	+	Protein of unknown function (DUF3897)	0.333
PF13428	-	Tetratricopeptide repeat	0.326
PF13531	+	Bacterial extracellular solute-binding protein	0.311
PF03693	-	Uncharacterised protein family (UPF0156)	0.298
PF13347	+	MFS/sugar transport protein	0.295
PF06569	+	Protein of unknown function (DUF1128)	0.291
PF06751	-	Ethanolamine ammonia lyase large subunit (EutB)	0.289
PF07695	-	7TM diverse intracellular signalling	0.286
PF13727	+	CoA-binding domain	0.278

PF04226	-	Transglycosylase associated protein	0.275
PF00563	-	EAL domain	0.273
PF04865	-	Baseplate J-like protein	0.272
PF13480	-	Acetyltransferase (GNAT) domain	0.272
PF13426	-	PAS domain	0.268
PF13343	+	Bacterial extracellular solute-binding protein	0.265
PF03960	+	ArsC family	0.252
PF06283	+	Trehalose utilisation	0.245
PF07943	+	Penicillin-binding protein 5, C-terminal domain	0.242
PF00685	-	Sulfotransferase domain	0.207
PF13427	-	Domain of unknown function (DUF4111)	0.202
PF13418	+	Galactose oxidase, central domain	0.202
PF03448	-	MgtE intracellular N domain	0.197
PF07411	-	Domain of unknown function (DUF1508)	0.195
PF09186	+	Domain of unknown function (DUF1949)	0.186
PF10340	-	Protein of unknown function (DUF2424)	0.180
PF12911	-	N-terminal TM domain of oligopeptide transport permease C	0.169
PF00350	-	Dynamin family	0.169
PF04280	-	Tim44-like domain	0.168
PF08349	-	Protein of unknown function (DUF1722)	0.166
PF01270	-	Glycosyl hydrolases family 8	0.148
PF13087	-	AAA domain	0.138
PF00011	-	Hsp20/alpha crystallin family	0.130
PF13594	+	Amidohydrolase	0.123
PF13807	-	G-rich domain on putative tyrosine kinase	0.119
PF01769	-	Divalent cation transporter	0.107
PF06452	+	Domain of unknown function (DUF1083)	0.103
PF08668	-	HDOD domain	0.091
PF13499	-	EF-hand domain pair	0.088
PF03610	-	PTS system fructose IIA component	0.088
PF13433	-	Periplasmic binding protein domain	0.074
PF08765	-	Mor transcription activator family	0.074
PF03747	-	ADP-ribosylglycohydrolase	0.072
PF07386	-	Protein of unknown function (DUF1499)	0.069
PF07508	-	Recombinase	0.069
PF13514	-	AAA domain	0.054
PF09084	-	NMT1/THI5 like	0.054
PF02110	-	Hydroxyethylthiazole kinase family	0.047
PF12895	-	Anaphase-promoting complex, cyclosome, subunit 3	0.032
PF00520	-	Ion transport protein	0.016
PF13588	-	Type I restriction enzyme R protein N terminus (HSDR_N)	-0.004
PF01895	-	PhoU domain	-0.008
PF00071	-	Ras family	-0.013
PF03102	-	NeuB family	-0.022
PF05147	-	Lanthionine synthetase C-like protein	-0.027
PF13643	-	Domain of unknown function (DUF4145)	-0.032
PF04024	-	PspC domain	-0.032
PF07022	-	Bacteriophage CI repressor helix-turn-helix domain	-0.039

PF13751	-	Transposase DDE domain	-0.046
PF04464	-	CDP-Glycerol:Poly(glycerophosphate) glycerophosphotransferase	-0.104
PF08780	+	Nucleotidyltransferase substrate binding protein like	-0.116
PF13395	-	HNH endonuclease	-0.123
PF01867	-	CRISPR associated protein Cas1	-0.142
PF01930	-	Domain of unknown function DUF83	-0.148
PF04018	-	Domain of unknown function (DUF368)	-0.166
PF10662	-	Ethanolamine utilisation - propanediol utilisation	-0.204
PF13181	-	Tetratricopeptide repeat	-0.206
PF07521	-	RNA-metabolising metallo-beta-lactamase	-0.216
PF04392	-	ABC transporter substrate binding protein	-0.261
PF01871	-	AMMECR1	-0.269
PF01643	-	Acyl-ACP thioesterase	-0.274
PF02065	-	Melibiase	-0.276
PF09827	-	CRISPR associated protein Cas2	-0.310
PF04011	-	LemA family	-0.364
PF02361	-	Cobalt transport protein	-0.377
PF13173	-	AAA domain	-0.458

Phenotype: Oxidase (Oxygen:Enzyme), Predictor: phypat

Pfam	class	Pfam_desc	cor
PF00032	+	Cytochrome b(C-terminal)/b6/petD	0.805
PF11614	+	IG-like fold at C-terminal of FixG, putative oxidoreductase	0.798
PF13631	+	Cytochrome b(N-terminal)/b6/petB	0.699
PF13442	+	Cytochrome C oxidase, cbb3-type, subunit III	0.661
PF01329	+	Pterin 4 alpha carbinolamine dehydratase	0.655
PF01068	+	ATP dependent DNA ligase domain	0.612
PF01292	+	Prokaryotic cytochrome b561	0.575
PF05573	+	NosL	0.566
PF03150	+	Di-haem cytochrome c peroxidase	0.542
PF03781	+	Sulfatase-modifying factor enzyme 1	0.511
PF08238	+	Sel1 repeat	0.495
PF13356	+	Domain of unknown function (DUF4102)	0.475
PF03435	+	Saccharopine dehydrogenase	0.474
PF02754	+	Cysteine-rich domain	0.438
PF05140	+	ResB-like family	0.403
PF03972	+	MmgE/PrpD family	0.369
PF04173	+	TQO small subunit DoxD	0.359
PF08003	+	Protein of unknown function (DUF1698)	0.354
PF05992	+	SbmA/BacA-like family	0.345
PF00016	+	Ribulose bisphosphate carboxylase large chain, catalytic domain	0.311
PF03824	+	High-affinity nickel-transport protein	0.305
PF08874	+	Domain of unknown function (DUF1835)	0.304
PF07732	+	Multicopper oxidase	0.300
PF03413	+	Peptidase propeptide and YPEB domain	0.297
PF05222	+	Alanine dehydrogenase/PNT, N-terminal domain	0.286
PF01262	+	Alanine dehydrogenase/PNT, C-terminal domain	0.279
PF02870	+	6-O-methylguanine DNA methyltransferase, ribonuclease-like domain	0.264

PF01865	+	Protein of unknown function DUF47	0.261
PF03831	+	PhnA protein	0.256
PF05170	-	AsmA family	0.241
PF09678	+	Cytochrome c oxidase caa3 assembly factor (Caa3_CtaG)	0.229
PF01734	+	Patatin-like phospholipase	0.227
PF00445	+	Ribonuclease T2 family	0.224
PF01841	+	Transglutaminase-like superfamily	0.218
PF11308	+	Glycosyl hydrolases related to GH101 family, GHL1-GHL3	0.213
PF14552	+	Tautomerase enzyme	0.191
PF13499	-	EF-hand domain pair	0.175
PF08867	+	FRG domain	0.151
PF05598	+	Transposase domain (DUF772)	0.142
PF04389	+	Peptidase family M28	0.129
PF05154	+	TM2 domain	0.126
PF13540	+	Regulator of chromosome condensation (RCC1) repeat	0.124
PF02436	+	Conserved carboxylase domain	0.120
PF01326	-	Pyruvate phosphate dikinase, PEP/pyruvate binding domain	0.099
PF03448	-	MgtE intracellular N domain	0.099
PF10412	+	Type IV secretion-system coupling protein DNA-binding domain	0.082
PF03848	+	Tellurite resistance protein TehB	0.061
PF00239	+	Resolvase, N terminal domain	0.048
PF01402	-	Ribbon-helix-helix protein, copG family	0.020
PF01558	-	Pyruvate ferredoxin/flavodoxin oxidoreductase	0.019
PF12832	-	MFS_1 like family	0.004
PF01425	-	Amidase	-0.002
PF13395	+	HNH endonuclease	-0.004
PF02796	+	Helix-turn-helix domain of resolvase	-0.011
PF04170	-	NlpE N-terminal domain	-0.039
PF13278	-	Putative amidotransferase	-0.063
PF01471	-	Putative peptidoglycan binding domain	-0.071
PF10722	-	Putative bacterial sensory transduction regulator	-0.077
PF13527	-	Acetyltransferase (GNAT) domain	-0.103
PF04343	-	Protein of unknown function, DUF488	-0.129
PF01321	-	Creatinase/Prolidase N-terminal domain	-0.183
PF05866	-	Endodeoxyribonuclease RusA	-0.184
PF01055	-	Glycosyl hydrolases family 31	-0.198
PF13749	-	ATP-dependent DNA helicase recG C-terminal	-0.200
PF04397	-	LytTr DNA-binding domain	-0.215
PF04326	-	Divergent AAA domain	-0.224
PF01515	-	Phosphate acetyl/butaryl transferase	-0.234
PF03729	-	Short repeat of unknown function (DUF308)	-0.236
PF04239	-	Protein of unknown function (DUF421)	-0.263
PF13740	-	ACT domain	-0.292
PF10371	-	Domain of unknown function	-0.296
PF04616	-	Glycosyl hydrolases family 43	-0.304
PF01895	-	PhoU domain	-0.322
PF02361	-	Cobalt transport protein	-0.362
PF01487	-	Type I 3-dehydroquinase	-0.371

PF00703	-	Glycosyl hydrolases family 2	-0.374
PF12822	-	Protein of unknown function (DUF3816)	-0.458
PF02457	-	DisA bacterial checkpoint controller nucleotide-binding	-0.495
PF00781	-	Diacylglycerol kinase catalytic domain	-0.500

Phenotype: Oxidase (Oxygen:Enzyme), Predictor: phypat+PGL

Pfam	class	Pfam_desc	cor
PF00032	+	Cytochrome b(C-terminal)/b6/petD	0.805
PF11614	+	IG-like fold at C-terminal of FixG, putative oxidoreductase	0.798
PF01329	+	Pterin 4 alpha carbinolamine dehydratase	0.655
PF01068	+	ATP dependent DNA ligase domain	0.612
PF01292	+	Prokaryotic cytochrome b561	0.575
PF00033	+	Cytochrome b(N-terminal)/b6/petB	0.572
PF02600	+	Disulfide bond formation protein DsbB	0.536
PF13386	+	Cytochrome C biogenesis protein transmembrane region	0.510
PF04820	+	Tryptophan halogenase	0.493
PF05157	+	Type II secretion system (T2SS), protein E, N-terminal domain	0.490
PF13356	+	Domain of unknown function (DUF4102)	0.475
PF03435	+	Saccharopine dehydrogenase	0.474
PF04982	+	HPP family	0.462
PF01220	+	Dehydroquinase class II	0.438
PF02754	+	Cysteine-rich domain	0.438
PF02190	+	ATP-dependent protease La (LON) domain	0.432
PF13505	+	Outer membrane protein beta-barrel domain	0.431
PF05726	+	Pirin C-terminal cupin domain	0.413
PF05618	+	Putative ATP-dependant zinc protease	0.408
PF10675	+	Protein of unknown function (DUF2489)	0.400
PF01078	+	Magnesium chelatase, subunit ChlI	0.385
PF08645	+	Polynucleotide kinase 3 phosphatase	0.376
PF00199	+	Catalase	0.364
PF10071	+	Zn-ribbon-containing, possibly nucleic-acid-binding protein (DUF2310)	0.363
PF13637	+	Ankyrin repeats (many copies)	0.360
PF04173	+	TQO small subunit DoxD	0.359
PF01124	+	MAPEG family	0.343
PF06472	+	ABC transporter transmembrane region 2	0.336
PF09835	+	Uncharacterized protein conserved in bacteria (DUF2062)	0.331
PF06628	+	Catalase-related immune-responsive	0.330
PF04191	+	Phospholipid methyltransferase	0.329
PF00394	+	Multicopper oxidase	0.326
PF11870	+	Domain of unknown function (DUF3390)	0.324
PF03899	+	ATP synthase I chain	0.297
PF03413	+	Peptidase propeptide and YPEB domain	0.297
PF09527	+	Putative F0F1-ATPase subunit (ATPase_gene1)	0.296
PF03737	+	Demethylmenaquinone methyltransferase	0.281
PF02696	+	Uncharacterized ACR, YdiU/UPF0061 family	0.281
PF14805	+	Tetrahydrodipicolinate N-succinyltransferase N-terminal	0.273
PF05954	-	Phage late control gene D protein (GPD)	0.272
PF04102	-	SlyX	0.265

PF01865	+	Protein of unknown function DUF47	0.261
PF13406	-	Transglycosylase SLT domain	0.260
PF00893	+	Small Multidrug Resistance protein	0.253
PF04632	-	Fusaric acid resistance protein family	0.245
PF00042	+	Globin	0.240
PF00764	+	Arginosuccinate synthase	0.229
PF05173	+	Dihydrodipicolinate reductase, C-terminus	0.228
PF00994	+	Probable molybdopterin binding domain	0.226
PF07726	+	ATPase family associated with various cellular activities (AAA)	0.218
PF00884	+	Sulfatase	0.213
PF07876	+	Stress responsive A/B Barrel Domain	0.205
PF00311	+	Phosphoenolpyruvate carboxylase	0.199
PF14464	-	Prokaryotic homologs of the JAB domain	0.196
PF10955	+	Protein of unknown function (DUF2757)	0.195
PF14069	+	Stage VI sporulation protein F	0.195
PF10628	+	Outer spore coat protein E (CotE)	0.195
PF11117	+	Protein of unknown function (DUF2626)	0.195
PF11808	-	Domain of unknown function (DUF3329)	0.175
PF13499	-	EF-hand domain pair	0.175
PF03788	+	LrgA family	0.174
PF00701	+	Dihydrodipicolinate synthetase family	0.172
PF04172	+	LrgB-like family	0.157
PF04030	+	D-arabinono-1,4-lactone oxidase	0.151
PF08212	-	Lipocalin-like domain	0.149
PF01455	+	HupF/HypC family	0.140
PF02698	+	DUF218 domain	0.139
PF01814	-	Hemerythrin HHE cation binding domain	0.133
PF13551	-	Winged helix-turn helix	0.126
PF09123	+	Domain of unknown function (DUF1931)	0.112
PF05067	+	Manganese containing catalase	0.073
PF08338	+	Domain of unknown function (DUF1731)	0.071
PF02661	+	Fic/DOC family	0.051
PF02639	-	Uncharacterized BCR, YaiI/YqxD family COG1671	0.051
PF05834	-	Lycopene cyclase protein	0.047
PF09278	-	MerR, DNA binding	0.041
PF05015	-	Plasmid maintenance system killer protein	0.040
PF13290	+	Chitobiase/beta-hexosaminidase C-terminal domain	0.038
PF04586	-	Caudovirus prohead protease	0.019
PF00395	+	S-layer homology domain	0.018
PF11700	-	Vacuole effluxer Atg22 like	0.014
PF00908	+	dTDP-4-dehydrorhamnose 3,5-epimerase	0.012
PF04261	-	Dyp-type peroxidase family	0.009
PF01555	+	DNA methylase	0.009
PF12832	-	MFS_1 like family	0.004
PF07885	-	Ion channel	-0.003
PF14691	-	Dihydroprymidine dehydrogenase domain II, 4Fe-4S cluster	-0.003
PF02498	-	BRO family, N-terminal domain	-0.019
PF03575	-	Peptidase family S51	-0.028

PF09669	-	Phage regulatory protein Rha (Phage_pRha)	-0.032
PF01427	-	D-ala-D-ala dipeptidase	-0.034
PF09831	-	Uncharacterized protein conserved in bacteria (DUF2058)	-0.038
PF04170	-	NlpE N-terminal domain	-0.039
PF03606	-	C4-dicarboxylate anaerobic carrier	-0.041
PF00708	+	Acylphosphatase	-0.050
PF07508	-	Recombinase	-0.066
PF13527	-	Acetyltransferase (GNAT) domain	-0.103
PF13632	-	Glycosyl transferase family group 2	-0.122
PF07664	-	Ferrous iron transport protein B C terminus	-0.124
PF09704	-	CRISPR-associated protein (Cas_Cas5)	-0.124
PF12849	-	PBP superfamily domain	-0.126
PF13514	-	AAA domain	-0.134
PF04883	-	Bacteriophage HK97-gp10, putative tail-component	-0.143
PF06210	-	Protein of unknown function (DUF1003)	-0.144
PF08840	-	BAAT / Acyl-CoA thioester hydrolase C terminal	-0.151
PF06580	-	Histidine kinase	-0.153
PF13495	-	Phage integrase, N-terminal SAM-like domain	-0.157
PF04493	-	Endonuclease V	-0.160
PF04961	+	Formiminotransferase-cyclodeaminase	-0.185
PF03610	-	PTS system fructose IIA component	-0.199
PF04397	-	LytTr DNA-binding domain	-0.215
PF01443	-	Viral (Superfamily 1) RNA helicase	-0.226
PF03747	-	ADP-ribosylglycohydrolase	-0.240
PF05135	-	Phage gp6-like head-tail connector protein	-0.252
PF13740	-	ACT domain	-0.292
PF01895	-	PhoU domain	-0.322
PF12464	-	Maltose acetyltransferase	-0.330
PF02361	-	Cobalt transport protein	-0.362
PF03611	-	PTS system sugar-specific permease component	-0.411
PF00874	-	PRD domain	-0.498
PF00781	-	Diacylglycerol kinase catalytic domain	-0.500

Phenotype: Hydrogen sulfide (Product), Predictor: phypat

Pfam	class	Pfam_desc	cor
PF13598	+	Domain of unknown function (DUF4139)	0.451
PF01930	+	Domain of unknown function DUF83	0.397
PF08753	+	NikR C terminal nickel binding domain	0.397
PF01155	+	Hydrogenase expression/synthesis hypA family	0.372
PF11761	+	Cobalamin biosynthesis central region	0.370
PF03956	+	Membrane protein of unknown function (DUF340)	0.295
PF10418	+	Iron-sulfur cluster binding domain of dihydroorotate dehydrogenase B	0.269
PF03577	+	Peptidase family C69	0.267
PF10589	+	NADH-ubiquinone oxidoreductase-F iron-sulfur binding region	0.237
PF08349	+	Protein of unknown function (DUF1722)	0.228
PF14526	+	Integron-associated effector binding protein	0.193
PF13289	+	SIR2-like domain	0.187
PF02369	+	Bacterial Ig-like domain (group 1)	0.187

PF06445	+	GyrI-like small molecule binding domain	0.182
PF03575	+	Peptidase family S51	0.158
PF10947	+	Protein of unknown function (DUF2628)	0.106
PF02423	+	Ornithine cyclodeaminase/mu-crystallin family	0.101
PF00145	-	C-5 cytosine-specific DNA methylase	-0.040
PF07669	-	Eco57I restriction-modification methylase	-0.127
PF00871	-	Acetokinase family	-0.127
PF05662	-	Coiled stalk of trimeric autotransporter adhesin	-0.142
PF12769	-	Domain of unknown function (DUF3814)	-0.142
PF14595	-	Thioredoxin	-0.147
PF05971	-	Protein of unknown function (DUF890)	-0.161
PF05137	-	Fimbrial assembly protein (PilN)	-0.169
PF13379	-	NMT1-like family	-0.172
PF04958	-	Arginine N-succinyltransferase beta subunit	-0.173
PF04995	-	Heme exporter protein D (CcmD)	-0.182
PF09084	-	NMT1/THI5 like	-0.190
PF01238	-	Phosphomannose isomerase type I	-0.192
PF13521	-	AAA domain	-0.192
PF00069	-	Protein kinase domain	-0.201
PF01425	-	Amidase	-0.208
PF01183	-	Glycosyl hydrolases family 25	-0.209
PF01055	-	Glycosyl hydrolases family 31	-0.214
PF02452	-	PemK-like protein	-0.219
PF13672	-	Protein phosphatase 2C	-0.232
PF03096	-	Ndr family	-0.237
PF07155	-	ECF-type riboflavin transporter, S component	-0.241
PF00468	-	Ribosomal protein L34	-0.247
PF14542	-	GCN5-related N-acetyl-transferase	-0.250
PF06277	-	Ethanolamine utilisation protein EutA	-0.256
PF04198	-	Putative sugar-binding domain	-0.261
PF01451	-	Low molecular weight phosphotyrosine protein phosphatase	-0.265
PF01575	-	MaoC like domain	-0.273
PF10282	-	Lactonase, 7-bladed beta-propeller	-0.275
PF00444	-	Ribosomal protein L36	-0.280
PF03070	-	TENA/THI-4/PQQC family	-0.284
PF00756	-	Putative esterase	-0.382
PF10410	-	DnaB-helicase binding domain of primase	-0.426

Phenotype: Hydrogen sulfide (Product), Predictor: phypat+PGL

Pfam	class	Pfam_desc	cor
PF13598	+	Domain of unknown function (DUF4139)	0.451
PF12914	+	SH3 domain of SH3b2 type	0.425
PF01930	+	Domain of unknown function DUF83	0.397
PF08753	+	NikR C terminal nickel binding domain	0.397
PF15580	+	Immunity protein 33	0.375
PF01155	+	Hydrogenase expression/synthesis hypA family	0.372
PF11761	+	Cobalamin biosynthesis central region	0.370
PF01314	+	Aldehyde ferredoxin oxidoreductase, domains 2 & 3	0.359

PF02256	+	Iron hydrogenase small subunit	0.359
PF09674	+	Protein of unknown function (DUF2400)	0.316
PF03956	+	Membrane protein of unknown function (DUF340)	0.295
PF11750	+	Protein of unknown function (DUF3307)	0.290
PF10418	+	Iron-sulfur cluster binding domain of dihydroorotate dehydrogenase B	0.269
PF03577	+	Peptidase family C69	0.267
PF03083	+	Sugar efflux transporter for intercellular exchange	0.265
PF13148	+	Protein of unknown function (DUF3987)	0.259
PF03102	+	NeuB family	0.241
PF09709	+	CRISPR-associated protein (Cas_Csd1)	0.237
PF03030	+	Inorganic H+ pyrophosphatase	0.230
PF14088	+	Domain of unknown function (DUF4268)	0.230
PF08349	+	Protein of unknown function (DUF1722)	0.228
PF14014	+	Protein of unknown function (DUF4230)	0.214
PF01867	+	CRISPR associated protein Cas1	0.214
PF13087	+	AAA domain	0.180
PF01643	+	Acyl-ACP thioesterase	0.174
PF06226	+	Protein of unknown function (DUF1007)	0.173
PF13310	+	Virulence protein RhuM family	0.173
PF13086	+	AAA domain	0.173
PF09821	+	C-terminal AAA-associated domain	0.162
PF11236	+	Protein of unknown function (DUF3037)	0.159
PF14281	+	PD-(D/E)XK nuclease superfamily	0.141
PF02592	+	Uncharacterized ACR, YhhQ family COG1738	0.123
PF03412	+	Peptidase C39 family	0.121
PF13650	+	Aspartyl protease	0.113
PF13711	+	Domain of unknown function (DUF4160)	0.108
PF02423	+	Ornithine cyclodeaminase/mu-crystallin family	0.101
PF08332	+	Calcium/calmodulin dependent protein kinase II Association	0.098
PF14487	+	Domain of unknown function (DUF4433)	0.096
PF03073	+	TspO/MBR family	0.079
PF13787	+	Protein of unknown function with HXXEE motif	0.049
PF09524	+	Conserved phage C-terminus (Phg_2220_C)	0.030
PF13740	-	ACT domain	-0.045
PF01094	+	Receptor family ligand binding region	-0.045
PF00704	+	Glycosyl hydrolases family 18	-0.060
PF00176	-	SNF2 family N-terminal domain	-0.098
PF05656	-	Protein of unknown function (DUF805)	-0.106
PF03706	-	Uncharacterised protein family (UPF0104)	-0.110
PF03895	-	YadA-like C-terminal region	-0.118
PF10076	-	Uncharacterized protein conserved in bacteria (DUF2313)	-0.119
PF04247	-	Invasion gene expression up-regulator, SirB	-0.142
PF07317	-	Flagellar regulator YcgR	-0.155
PF05971	-	Protein of unknown function (DUF890)	-0.161
PF13014	-	KH domain	-0.162
PF01741	-	Large-conductance mechanosensitive channel, MscL	-0.162
PF05239	-	PRC-barrel domain	-0.171
PF01593	-	Flavin containing amine oxidoreductase	-0.175

PF01557	-	Fumarylacetoacetate (FAA) hydrolase family	-0.180
PF05768	-	Glutaredoxin-like domain (DUF836)	-0.193
PF01183	-	Glycosyl hydrolases family 25	-0.209
PF00471	-	Ribosomal protein L33	-0.237
PF07155	-	ECF-type riboflavin transporter, S component	-0.241
PF00367	-	phosphotransferase system, EIIB	-0.250
PF11967	-	Recombination protein O N terminal	-0.267
PF00358	-	phosphoenolpyruvate-dependent sugar phosphotransferase system, EIIA 1	-0.275
PF00444	-	Ribosomal protein L36	-0.280
PF03483	-	B3/4 domain	-0.293

Phenotype: Casein hydrolysis (Proteolysis), Predictor: phypat

Pfam	class	Pfam_desc	cor
PF05922	+	Peptidase inhibitor I9	0.850
PF07070	+	SpoOM protein	0.850
PF00245	+	Alkaline phosphatase	0.747
PF01258	+	Prokaryotic dksA/traR C4-type zinc finger	0.717
PF02638	+	Glycosyl hydrolase like GH101	0.689
PF01699	+	Sodium/calcium exchanger protein	0.658
PF00873	+	AcrB/AcrD/AcrF family	0.644
PF00801	+	PKD domain	0.587
PF02522	+	Aminoglycoside 3-N-acetyltransferase	0.512
PF13464	+	Domain of unknown function (DUF4115)	0.479
PF13536	-	Multidrug resistance efflux transporter	-0.040
PF01261	-	Xylose isomerase-like TIM barrel	-0.040
PF00071	-	Ras family	-0.076
PF11798	-	IMS family HHH motif	-0.097
PF14501	-	GHKL domain	-0.142
PF13333	-	Integrase core domain	-0.143
PF03976	-	Polyphosphate kinase 2 (PPK2)	-0.168
PF00122	-	E1-E2 ATPase	-0.248
PF00707	-	Translation initiation factor IF-3, C-terminal domain	-0.251
PF00719	-	Inorganic pyrophosphatase	-0.275
PF01515	-	Phosphate acetyl/butaryl transferase	-0.281
PF04343	-	Protein of unknown function, DUF488	-0.319
PF14821	-	Threonine synthase N terminus	-0.330
PF07751	-	Abi-like protein	-0.330
PF07669	-	Eco57I restriction-modification methylase	-0.330
PF03729	-	Short repeat of unknown function (DUF308)	-0.400
PF02381	-	MraZ protein	-0.411
PF13338	-	Domain of unknown function (DUF4095)	-0.411
PF06738	-	Protein of unknown function (DUF1212)	-0.415
PF12821	-	Protein of unknown function (DUF3815)	-0.415
PF05167	-	Uncharacterised ACR (DUF711)	-0.444

Phenotype: Casein hydrolysis (Proteolysis), Predictor: phypat+PGL

Pfam	class	Pfam_desc	cor
PF00245	+	Alkaline phosphatase	0.747

PF04893	+	Yip1 domain	0.746
PF01258	+	Prokaryotic dksA/traR C4-type zinc finger	0.717
PF04134	+	Protein of unknown function, DUF393	0.705
PF01699	+	Sodium/calcium exchanger protein	0.658
PF02834	+	LigT like Phosphoesterase	0.657
PF13686	+	DsrE/DsrF/DrsH-like family	0.647
PF13712	+	Glycosyltransferase like family	0.647
PF00873	+	AcrB/AcrD/AcrF family	0.644
PF13638	+	PIN domain	0.632
PF05448	+	Acetyl xylan esterase (AXE1)	0.607
PF01903	+	CbiX	0.599
PF00704	+	Glycosyl hydrolases family 18	0.599
PF02635	+	DsrE/DsrF-like family	0.599
PF00801	+	PKD domain	0.587
PF01242	+	6-pyruvoyl tetrahydropterin synthase	0.572
PF03102	+	NeuB family	0.572
PF01175	+	Urocanase	0.539
PF01987	+	Mitochondrial biogenesis AIM24	0.539
PF01578	+	Cytochrome C assembly protein	0.509
PF13464	+	Domain of unknown function (DUF4115)	0.479
PF03806	+	AbgT putative transporter family	0.475
PF09992	+	Predicted periplasmic protein (DUF2233)	0.459
PF09685	+	Tic20-like protein	0.355
PF09614	+	CRISPR-associated protein (Cas_Csy2)	0.353
PF02300	+	Fumarate reductase subunit C	0.353
PF09822	+	ABC-type uncharacterized transport system	0.353
PF00265	+	Thymidine kinase	0.347
PF03259	+	Roadblock/LC7 domain	0.346
PF02274	+	Amidinotransferase	0.313
PF14103	+	Domain of unknown function (DUF4276)	0.281
PF04170	+	NlpE N-terminal domain	0.251
PF03308	+	ArgK protein	0.214
PF02366	+	Dolichyl-phosphate-mannose-protein mannosyltransferase	0.212
PF04018	+	Domain of unknown function (DUF368)	0.188
PF13375	+	RnfC Barrel sandwich hybrid domain	0.116
PF04228	+	Putative neutral zinc metallopeptidase	-0.029
PF05133	-	Phage portal protein, SPP1 Gp6-like	-0.034
PF13536	-	Multidrug resistance efflux transporter	-0.040
PF14501	-	GHKL domain	-0.142
PF13333	-	Integrase core domain	-0.143
PF07751	-	Abi-like protein	-0.330
PF14821	-	Threonine synthase N terminus	-0.330
PF07669	-	Eco57I restriction-modification methylase	-0.330
PF12821	-	Protein of unknown function (DUF3815)	-0.415
PF06738	-	Protein of unknown function (DUF1212)	-0.415

Phenotype: Gelatin hydrolysis (Proteolysis), Predictor: phypat

Pfam	class	Pfam_desc	cor

PF05922	+	Peptidase inhibitor I9	0.523
PF03174	+	Chitobiase/beta-hexosaminidase C-terminal domain	0.519
PF13638	+	PIN domain	0.478
PF04389	+	Peptidase family M28	0.474
PF08392	+	FAE1/Type III polyketide synthase-like protein	0.464
PF00801	+	PKD domain	0.464
PF13424	+	Tetratricopeptide repeat	0.462
PF01175	+	Urocanase	0.446
PF11308	+	Glycosyl hydrolases related to GH101 family, GHL1-GHL3	0.433
PF00704	+	Glycosyl hydrolases family 18	0.425
PF13385	+	Concanavalin A-like lectin/glucanases superfamily	0.422
PF07726	+	ATPase family associated with various cellular activities (AAA)	0.412
PF01841	+	Transglutaminase-like superfamily	0.408
PF12823	+	Domain of unknown function (DUF3817)	0.385
PF08327	+	Activator of Hsp90 ATPase homolog 1-like protein	0.376
PF08668	+	HDOD domain	0.376
PF00082	+	Subtilase family	0.371
PF05050	+	Methyltransferase FkbM domain	0.367
PF05067	+	Manganese containing catalase	0.364
PF04989	+	Cephalosporin hydroxylase	0.360
PF07691	+	PA14 domain	0.360
PF14470	+	Bacterial PH domain	0.353
PF03413	+	Peptidase propeptide and YPEB domain	0.323
PF07786	+	Protein of unknown function (DUF1624)	0.315
PF01037	+	AsnC family	0.313
PF09860	+	Uncharacterized protein conserved in bacteria (DUF2087)	0.285
PF04552	+	Sigma-54, DNA binding domain	0.280
PF05402	+	Coenzyme PQQ synthesis protein D (PqqD)	0.280
PF09423	+	PhoD-like phosphatase	0.269
PF05272	+	Virulence-associated protein E	0.258
PF13549	+	ATP-grasp domain	0.240
PF13250	+	Domain of unknown function (DUF4041)	0.233
PF04014	+	Antidote-toxin recognition MazE	0.205
PF01385	+	Probable transposase	0.173
PF07282	+	Putative transposase DNA-binding domain	0.173
PF02897	+	Prolyl oligopeptidase, N-terminal beta-propeller domain	0.142
PF04397	-	LytTr DNA-binding domain	0.043
PF00343	-	Carbohydrate phosphorylase	0.040
PF00145	-	C-5 cytosine-specific DNA methylase	-0.016
PF02574	-	Homocysteine S-methyltransferase	-0.029
PF07751	-	Abi-like protein	-0.031
PF00545	-	ribonuclease	-0.042
PF09290	-	Prokaryotic acetaldehyde dehydrogenase, dimerisation	-0.057
PF13401	-	AAA domain	-0.059
PF13566	-	Domain of unknown function (DUF4130	-0.062
PF02386	-	Cation transport protein	-0.063
PF01058	-	NADH ubiquinone oxidoreductase, 20 Kd subunit	-0.074
PF05198	-	Translation initiation factor IF-3, N-terminal domain	-0.077

PF00596	-	Class II Aldolase and Adducin N-terminal domain	-0.096
PF03354	-	Phage Terminase	-0.096
PF02744	-	Galactose-1-phosphate uridyl transferase, C-terminal domain	-0.110
PF10588	-	NADH-ubiquinone oxidoreductase-G iron-sulfur binding region	-0.114
PF13740	-	ACT domain	-0.117
PF06779	-	Protein of unknown function (DUF1228)	-0.117
PF02746	-	Mandelate racemase / muconate lactonizing enzyme, N-terminal domain	-0.122
PF01643	-	Acyl-ACP thioesterase	-0.125
PF10369	-	Small subunit of acetolactate synthase	-0.127
PF03960	-	ArsC family	-0.132
PF03051	-	Peptidase C1-like family	-0.135
PF13433	-	Periplasmic binding protein domain	-0.141
PF09704	-	CRISPR-associated protein (Cas_Cas5)	-0.142
PF01717	-	Cobalamin-independent synthase, Catalytic domain	-0.153
PF03480	-	Bacterial extracellular solute-binding protein, family 7	-0.153
PF02153	-	Prephenate dehydrogenase	-0.153
PF03601	-	Conserved hypothetical protein 698	-0.154
PF05145	-	Putative ammonia monooxygenase	-0.165
PF02934	-	GatB/GatE catalytic domain	-0.173
PF02575	-	YbaB/EbfC DNA-binding family	-0.180
PF00707	-	Translation initiation factor IF-3, C-terminal domain	-0.181
PF03951	-	Glutamine synthetase, beta-Grasp domain	-0.186
PF00742	-	Homoserine dehydrogenase	-0.186
PF00543	-	Nitrogen regulatory protein P-II	-0.188
PF10397	-	Adenylosuccinate lyase C-terminus	-0.188
PF03852	-	DNA mismatch endonuclease Vsr	-0.192
PF02686	-	Glu-tRNAGln amidotransferase C subunit	-0.201
PF05437	-	Branched-chain amino acid transport protein (AzlD)	-0.204
PF08223	-	PaaX-like protein C-terminal domain	-0.205
PF04290	-	Tripartite ATP-independent periplasmic transporters, DctQ component	-0.211
PF01425	-	Amidase	-0.212
PF02253	-	Phospholipase A1	-0.218
PF05336	-	Domain of unknown function (DUF718)	-0.225
PF03352	-	Methyladenine glycosylase	-0.231
PF12738	-	twin BRCT domain	-0.239
PF06472	-	ABC transporter transmembrane region 2	-0.244
PF05992	-	SbmA/BacA-like family	-0.244
PF03797	-	Autotransporter beta-domain	-0.272
PF04131	-	Putative N-acetylmannosamine-6-phosphate epimerase	-0.272
PF00171	-	Aldehyde dehydrogenase family	-0.275
PF02091	-	Glycyl-tRNA synthetase alpha subunit	-0.278
PF02092	-	Glycyl-tRNA synthetase beta subunit	-0.278
PF07972	-	NrdI Flavodoxin like	-0.288
PF01297	-	Periplasmic solute binding protein family	-0.291

Phenotype: Gelatin hydrolysis (Proteolysis), Predictor: phypat+PGL

Pfam	class	Pfam_desc	cor
PF05547	+	Immune inhibitor A peptidase M6	0.528

PF05922	+	Peptidase inhibitor I9	0.523
PF13638	+	PIN domain	0.478
PF04389	+	Peptidase family M28	0.474
PF08014	+	Domain of unknown function (DUF1704)	0.465
PF08392	+	FAE1/Type III polyketide synthase-like protein	0.464
PF00801	+	PKD domain	0.464
PF04209	+	homogentisate 1,2-dioxygenase	0.452
PF01175	+	Urocanase	0.446
PF00704	+	Glycosyl hydrolases family 18	0.425
PF13385	+	Concanavalin A-like lectin/glucanases superfamily	0.422
PF07726	+	ATPase family associated with various cellular activities (AAA)	0.412
PF01944	+	Integral membrane protein DUF95	0.410
PF01841	+	Transglutaminase-like superfamily	0.408
PF12823	+	Domain of unknown function (DUF3817)	0.385
PF08668	+	HDOD domain	0.376
PF08327	+	Activator of Hsp90 ATPase homolog 1-like protein	0.376
PF01329	+	Pterin 4 alpha carbinolamine dehydratase	0.371
PF09697	+	Protein of unknown function (Porph_ging)	0.369
PF05067	+	Manganese containing catalase	0.364
PF01987	+	Mitochondrial biogenesis AIM24	0.364
PF01483	+	Proprotein convertase P-domain	0.360
PF14470	+	Bacterial PH domain	0.353
PF13855	+	Leucine rich repeat	0.352
PF00932	+	Lamin Tail Domain	0.335
PF04072	+	Leucine carboxyl methyltransferase	0.331
PF13305	+	WHG domain	0.315
PF07786	+	Protein of unknown function (DUF1624)	0.315
PF13243	+	Prenyltransferase-like	0.314
PF12950	+	TaqI-like C-terminal specificity domain	0.301
PF13540	+	Regulator of chromosome condensation (RCC1) repeat	0.301
PF09860	+	Uncharacterized protein conserved in bacteria (DUF2087)	0.285
PF08331	+	Domain of unknown function (DUF1730)	0.284
PF05402	+	Coenzyme PQQ synthesis protein D (PqqD)	0.280
PF03190	+	Protein of unknown function, DUF255	0.280
PF01391	+	Collagen triple helix repeat (20 copies)	0.272
PF09423	+	PhoD-like phosphatase	0.269
PF13690	+	Chemotaxis phosphatase CheX	0.269
PF14259	+	RNA recognition motif (a.k.a. RRM, RBD, or RNP domain)	0.268
PF13893	+	RNA recognition motif. (a.k.a. RRM, RBD, or RNP domain)	0.268
PF00076	+	RNA recognition motif. (a.k.a. RRM, RBD, or RNP domain)	0.268
PF12732	+	YtxH-like protein	0.268
PF00725	+	3-hydroxyacyl-CoA dehydrogenase, C-terminal domain	0.260
PF00041	+	Fibronectin type III domain	0.258
PF03102	+	NeuB family	0.257
PF05088	+	Bacterial NAD-glutamate dehydrogenase	0.256
PF05567	+	Neisseria PilC beta-propeller domain	0.254
PF00891	+	O-methyltransferase	0.250
PF04012	+	PspA/IM30 family	0.249

PF13631	+	Cytochrome b(N-terminal)/b6/petB	0.249
PF07831	+	Pyrimidine nucleoside phosphorylase C-terminal domain	0.224
PF05708	+	Orthopoxvirus protein of unknown function (DUF830)	0.223
PF11992	+	Domain of unknown function (DUF3488)	0.219
PF09822	+	ABC-type uncharacterized transport system	0.219
PF01923	+	Cobalamin adenosyltransferase	0.217
PF10604	+	Polyketide cyclase / dehydrase and lipid transport	0.217
PF05036	+	Sporulation related domain	0.209
PF01258	+	Prokaryotic dksA/traR C4-type zinc finger	0.209
PF13469	-	Sulfotransferase family	0.201
PF01144	+	Coenzyme A transferase	0.199
PF02709	+	N-terminal domain of galactosyltransferase	0.196
PF13646	+	HEAT repeats	0.194
PF02086	+	D12 class N6 adenine-specific DNA methyltransferase	0.188
PF05195	+	Aminopeptidase P, N-terminal domain	0.165
PF00480	+	ROK family	0.165
PF02638	+	Glycosyl hydrolase like GH101	0.153
PF10263	+	SprT-like family	0.146
PF05656	+	Protein of unknown function (DUF805)	0.135
PF13452	-	N-terminal half of MaoC dehydratase	0.122
PF04324	-	BFD-like [2Fe-2S] binding domain	0.090
PF03853	+	YjeF-related protein N-terminus	0.088
PF01946	-	Thi4 family	0.075
PF05683	-	Fumarase C-terminus	0.069
PF05681	-	Fumarate hydratase (Fumerase)	0.069
PF11967	+	Recombination protein O N terminal	0.034
PF10947	-	Protein of unknown function (DUF2628)	0.031
PF03109	+	ABC1 family	0.031
PF05154	-	TM2 domain	0.021
PF01293	-	Phosphoenolpyruvate carboxykinase	0.004
PF00145	-	C-5 cytosine-specific DNA methylase	-0.016
PF10509	-	Galactokinase galactose-binding signature	-0.023
PF01915	-	Glycosyl hydrolase family 3 C-terminal domain	-0.027
PF06821	-	Serine hydrolase	-0.037
PF13449	-	Esterase-like activity of phytase	-0.042
PF14310	-	Fibronectin type III-like domain	-0.053
PF13089	-	Polyphosphate kinase N-terminal domain	-0.078
PF03459	-	TOBE domain	-0.083
PF13602	-	Zinc-binding dehydrogenase	-0.084
PF02503	-	Polyphosphate kinase middle domain	-0.087
PF12769	-	Domain of unknown function (DUF3814)	-0.091
PF01867	-	CRISPR associated protein Cas1	-0.095
PF00596	-	Class II Aldolase and Adducin N-terminal domain	-0.096
PF01960	-	ArgJ family	-0.100
PF08402	-	TOBE domain	-0.102
PF12682	-	Flavodoxin	-0.113
PF12974	-	ABC transporter, phosphonate, periplasmic substrate-binding protein	-0.116
PF13740	-	ACT domain	-0.117

PF01219	-	Prokaryotic diacylglycerol kinase	-0.119
PF00180	-	Isocitrate/isopropylmalate dehydrogenase	-0.122
PF02746	-	Mandelate racemase / muconate lactonizing enzyme, N-terminal domain	-0.122
PF01643	-	Acyl-ACP thioesterase	-0.125
PF10369	-	Small subunit of acetolactate synthase	-0.127
PF07702	-	UTRA domain	-0.127
PF13433	-	Periplasmic binding protein domain	-0.141
PF01361	-	Tautomerase enzyme	-0.146
PF02153	-	Prephenate dehydrogenase	-0.153
PF03601	-	Conserved hypothetical protein 698	-0.154
PF04174	-	A circularly permuted ATPgrasp	-0.158
PF02133	-	Permease for cytosine/purines, uracil, thiamine, allantoin	-0.160
PF02934	-	GatB/GatE catalytic domain	-0.173
PF02637	-	GatB domain	-0.173
PF00848	-	Ring hydroxylating alpha subunit (catalytic domain)	-0.178
PF03239	-	Iron permease FTR1 family	-0.181
PF00742	-	Homoserine dehydrogenase	-0.186
PF03609	-	PTS system sorbose-specific iic component	-0.188
PF00543	-	Nitrogen regulatory protein P-II	-0.188
PF10397	-	Adenylosuccinate lyase C-terminus	-0.188
PF03613	-	PTS system mannose/fructose/sorbose family IID component	-0.195
PF02686	-	Glu-tRNAGln amidotransferase C subunit	-0.201
PF05437	-	Branched-chain amino acid transport protein (AzlD)	-0.204
PF01425	-	Amidase	-0.212
